# Proteomic analysis of chromophobe renal cell carcinoma and benign renal oncocytoma biopsies reveals shared metabolic dysregulation

**DOI:** 10.1186/s12014-023-09443-8

**Published:** 2023-11-28

**Authors:** Luis B. Carvalho, Susana Jorge, Hugo López-Fernández, Carlos Lodeiro, Rajiv Dhir, Luis Campos Pinheiro, Mariana Medeiros, Hugo M. Santos, José L. Capelo

**Affiliations:** 1 BIOSCOPE Research Group, LAQV-REQUIMTE, Department of Chemistry, NOVA School of Science and Technology, FCT NOVA, Universidade NOVA de Lisboa, 2829-516 Caparica, Portugal; 2 PROTEOMASS Scientific Society, Departamental Building, FCT-NOVA, Caparica Campus, 2829-516 Caparica, Portugal; 3 CINBIO, Department of Computer Science, ESEI-Escuela Superior de Ingeniería Informática, Universidade de Vigo, 32004 Ourense, Spain; 4 SING Research Group, Galicia Sur Health Research Institute (IIS Galicia Sur), SERGAS-UVIGO, 36213 Vigo, Spain; 5 Department of Pathology, University of Pittsburgh Medical Center, Pittsburgh, PA USA; 6 Urology Department, Central Lisbon Hospital Center, Lisbon, Portugal; 7 NOVA Medical School, NOVA University of Lisbon, Lisbon, Portugal

**Keywords:** OCT-embedded tissues, Total Protein Approach, Label-free Quantification, Mass Spectrometry, Chromophobe Renal Cell Carcinoma, Renal Oncocytoma

## Abstract

**Background:**

This study investigates the proteomic landscapes of chromophobe renal cell carcinoma (chRCC) and renal oncocytomas (RO), two subtypes of renal cell carcinoma that together account for approximately 10% of all renal tumors. Despite their histological similarities and shared origins, chRCC is a malignant tumor necessitating aggressive intervention, while RO, a benign growth, is often subject to overtreatment due to difficulties in accurate differentiation.

**Methods:**

We conducted a label-free quantitative proteomic analysis on solid biopsies of chRCC (n = 5), RO (n = 5), and normal adjacent tissue (NAT, n = 5). The quantitative analysis was carried out by comparing protein abundances between tumor and NAT specimens. Our analysis identified a total of 1610 proteins across all samples, with 1379 (85.7%) of these proteins quantified in at least seven out of ten LC‒MS/MS runs for one renal tissue type (chRCC, RO, or NAT).

**Results:**

Our findings revealed significant similarities in the dysregulation of key metabolic pathways, including carbohydrate, lipid, and amino acid metabolism, in both chRCC and RO. Compared to NAT, both chRCC and RO showed a marked downregulation in gluconeogenesis proteins, but a significant upregulation of proteins integral to the citrate cycle. Interestingly, we observed a distinct divergence in the oxidative phosphorylation pathway, with RO showing a significant increase in the number and degree of alterations in proteins, surpassing that observed in chRCC.

**Conclusions:**

This study underscores the value of integrating high-resolution mass spectrometry protein quantification to effectively characterize and differentiate the proteomic landscapes of solid tumor biopsies diagnosed as chRCC and RO. The insights gained from this research offer valuable information for enhancing our understanding of these conditions and may aid in the development of improved diagnostic and therapeutic strategies.

**Supplementary Information:**

The online version contains supplementary material available at 10.1186/s12014-023-09443-8.

## Introduction

Renal cell carcinoma (RCC), a disease characterized by abnormal cell proliferation in the epithelial cells of the kidney, accounts for 400,000 new cases of adult kidney cancer globally each year [1]. There are several subtypes within the RCC classification, each displaying unique histological and cytological phenotypes [2]. Chromophobe renal cell carcinoma (chRCC) and renal oncocytomas (RO) are two RCC subtypes that represent approximately 10% of all renal tumors [3]. RO, a benign growth often found in the kidney collecting ducts, typically lends itself to conservative treatment approaches. Conversely, chRCC is a malignant tumor that, despite sharing a similar origin in distal kidney nephrons, requires more aggressive intervention to prevent dire consequences to the patient [3]. Given the strikingly similar anatomical origins and histological features of these two tumor types, their accurate differentiation poses a significant challenge [4]. Therefore, most patients undergo surgery, often involving a partial or total nephrectomy. This can lead to overtreatment in RO, as benign lesions may not require such invasive interventions if correctly identified beforehand. Thus, accurately distinguishing between these two conditions is essential not only to guide optimal treatment strategies but also to reduce the potential burden on patients and the healthcare system, thereby improving overall patient care [4, 5].

Building on our recent development of a highly efficient, ultrasonic-based methodology for extracting, identifying, and quantifying the proteome of solid renal biopsies embedded in optimal cutting temperature (OCT) compound [6], we sought further to leverage these technological advancements in our current study. In this context, we employed the total protein approach (TPA), a computational tool adept at converting spectral intensities into protein concentrations. The efficacy of the TPA methodology has been proven in various scenarios, such as evaluating metabolic pathways in slow and fast skeletal muscle [7], exploring biological processes associated with colorectal cancer [8], and assessing the impact on mitochondrial bioenergetics in disease-induced conditions [9]. Additionally, we previously investigated differentially expressed proteins in renal cancers using high-resolution MS combined with the TPA method [10]. In this work, we focused on the metabolic features associated with chRCC and RO versus NAT using the TPA method to identify undisclosed new features characterizing each tumor [11]. We compared our findings with the literature addressing the metabolic characteristics of these renal neoplasms [12, 13].

## Materials and methods

### Study design and sampling

In this manuscript, we have conscientiously reused a portion of the raw data from our previous by Jorge et al. [11]. The raw data in question forms the basis of a comprehensive dataset that was collected with significant effort and resources. This dataset is rich and multifaceted, allowing for various analyses and interpretations. While our previous publication focused on the identification of novel histochemical markers in renal neoplasm, the current manuscript focuses on the discovery of shared metabolic features between chromophobe renal cell carcinoma and renal oncocytoma. We believe that the use of this data, in this manner, is a responsible use of resources, avoiding unnecessary duplication of data collection efforts. These data comprises the use of high-resolution mass spectrometry to examine the proteomes of 15 human renal tissue biopsies that were flash-frozen and embedded in OCT. These included chromophobe renal cell carcinoma (chRCC, n = 5), renal oncocytoma (RO, n = 5) and normal adjacent renal tissue (NAT, n = 5). The University of Pittsburgh Biospecimen Core provided the human kidney tissue samples used in this study, which received approval from the University of Pittsburgh's Institutional Review Board (IRB # 02–077). All neoplasms in the study consisted of at least 85% tumor cells. Additional file 1: Table S1 summarizes the patient data involved in this research.

### Proteomic analysis

The processing of biopsies followed the methodology outlined in Jorge et al. [6] Briefly, tissues were first cleaned of OCT using an ultrasonic bath model TI-H-5 from Elma (Singen, Germany). Then, proteins were extracted in 8 M urea prepared in 25 mM ammonium bicarbonate using an ultrasonic probe (UP50H from Hielscher Ultrasonics, Teltow, Germany). Next, protein digestion was carried out with trypsin for four minutes using an ultrasonic microplate horn assembly device (QSonica, Newtown, CT, USA). The digested proteome extracts were subsequently analyzed by a label-free nanoLC-HR-MS/MS approach (UHR-QqTOF IMPACT HD from Bruker Daltonics, Bremen, Germany) as described before by Jorge et al. [11] A step-by-step protocol, including OCT Cleaning, proteome extraction, proteome digestion, and LC–MS/MS data acquisition is available in Additional file 2.

### Data analysis and statistics

The MaxQuant software V1.6.0.16 was utilized for relative label-free quantification. All raw files were processed in a single run using default settings [14, 15]. The Andromeda search engine was employed for database searches, using the UniProt-SwissProt Human database as a reference, along with a database of common contaminants.

For absolute protein quantification, the Total Protein Approach (TPA) was used to examine raw spectral intensities from the MaxQuant output [10, 11]. The calculation of protein concentration was performed as follows:

$$c \left(i\right)= \frac{MS\;signal\;(i)}{total \;MS \;signal \;x \;MW\; (i)} \left[\frac{mol}{g\;total\;protein}\right]$$

Data processing was performed using Perseus (version 1.6.15.0) with default settings [16, 17]. In brief, reverse hits and proteins only identified by site were removed from the protein list, and TPA-based concentrations were log_2_-transformed to reduce the effect of outliers. Protein groups were filtered based on a minimum presence of 70% in at least one group. Pearson correlation was performed on filtered values. Missing values were imputed from the total matrix with width = 0.5 and downshift = 1.8. PCA was performed on the filtered and imputed data. Log ratios were calculated as the difference in average log_2_ values between the two conditions tested in volcano plots (two-tailed Student’s t test, FDR = 0.01 and S0 = 0.1). Protein network analysis was integrated and visualized using the software platform Cytoscape V3.8.2 with the application StringApp V1.6.0 [18]. KEGG [19], Reactome [20] and GO terms [21] were used as ontology databases.

## Results

### Renal tumor profiling

**Fig. 1:**
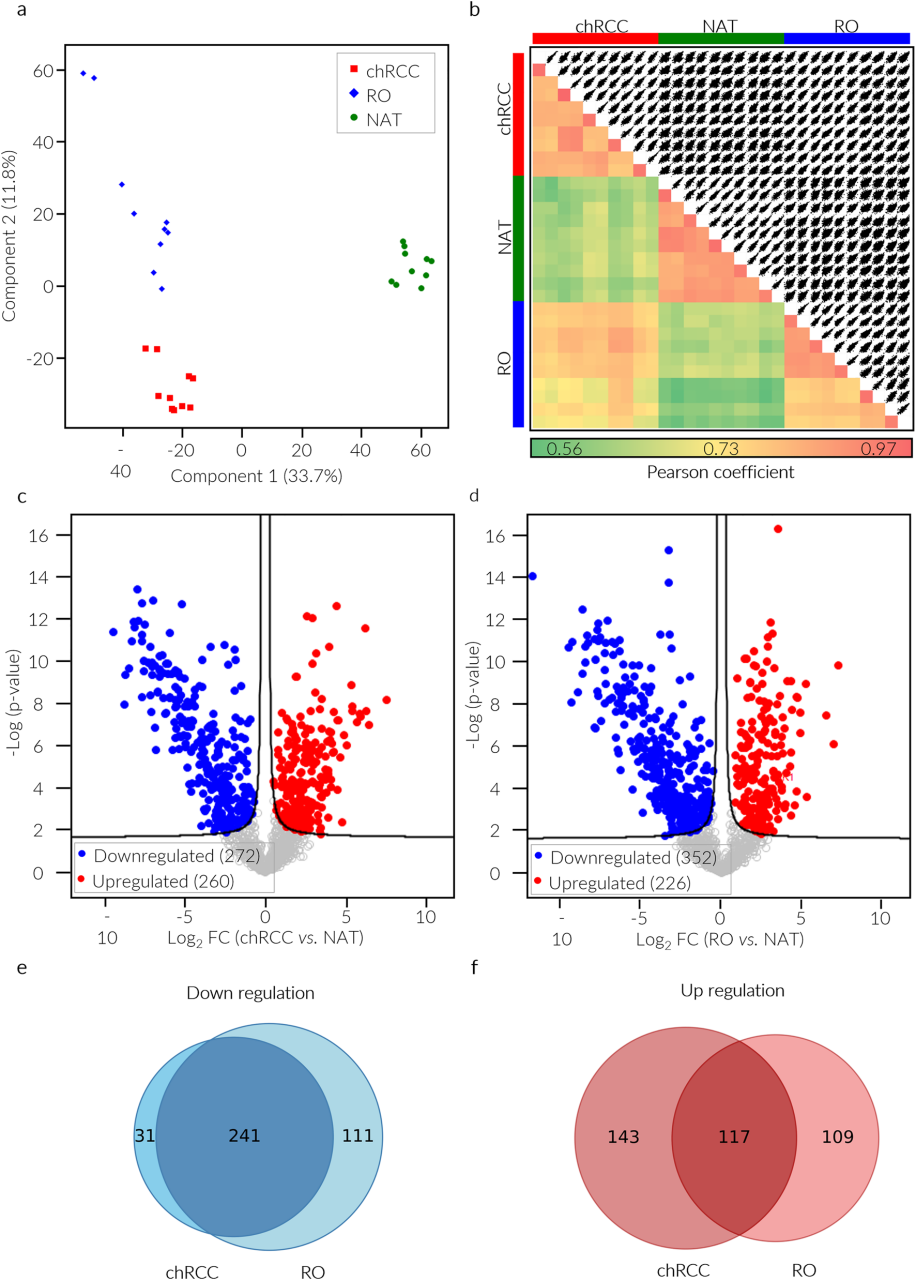
Classification of human renal tissue proteomes and protein expression profiles in tumors relative to NATs. Two instrumental replicates were run for each biopsy, resulting in 30 chromatograms that identified a total of 1610 proteins. Of these proteins, a total of 1379 were obtained from at least one tissue group (chRCC, RO, or NAT), with a reproducibility ranging from 70 to 100%. **a** Principal component analysis (PCA) of chRCC, RO, and NAT group samples. **b** Heatmap representation of Pearson correlation of biological (n = 5) and technical (n = 2, each sample) replicates (lower left) in combination with a scatterplot matrix protein of TPA-based quantification (upper right) demonstrating the reproducibility of the MS data. Volcano plots illustrate the significantly different (FDR = 0.01, S0 = 0.1) protein expression levels between (**c**) chRCC and NAT samples and (**d**) RO and NAT samples. Venn diagrams comparing proteomes of chRCC and RO in terms of the (**e**) downregulated proteins and (**f**) upregulated proteins

The tissue biopsies were analyzed in duplicate using liquid chromatography coupled to tandem mass spectrometry (nanoLC-ESI-HR-MS/MS). Raw spectral intensity values were converted into absolute concentrations using the TPA method [10]. A total of 1610 proteins were identified (1% FDR) across all samples, with 1379 (85.7%) of these proteins being quantified in at least seven out of the ten LC–MS/MS runs for one renal tissue type (chRCC, RO or NAT). Figure 1a displays the principal component analysis (PCA) of the quantified proteins, showing that each specimen class segregates into distinct clusters.

The reproducibility of the MS biological replicates was verified by comparing the Pearson correlation between all pairs of samples as well as visualizing pairwise scatter plots in a matrix (Fig. 1b). The calculated Pearson correlation coefficients spanned from 0.79 to 0.97 for chRCC, from 0.76 to 0.95 for RO, and from 0.83 to 0.95 for NAT. The Pearson coefficients were higher when tumors were compared to each other (0.68–0.86) than when compared to NAT (0.56–0.70). These results underline a marked deviation of both chRCC and RO from NAT. Furthermore, the data also demonstrate that there are discernible differences between chRCC and RO, highlighting the potential to differentiate these two neoplasms using this profiling approach.

### Dysregulated proteins between tumors and NATs

A two-tailed Student’s t test (FDR = 0.01 and S0 = 0.1) was used to distinguish the abundance of proteins between each tumor and NAT. In chRCC, 532 proteins exhibited significant differences compared to NAT, with 260 upregulated and 272 downregulated proteins (Fig. 1c). Similarly, in the case of RO versus NAT, 578 proteins showed significant differences, with 226 upregulated and 352 downregulated proteins (Fig. 1d). Comparing the upregulated and downregulated proteins between each tumor and NAT, 241 downregulated proteins (63%) and 117 upregulated proteins (32%) were found to be common (Fig. 1e, f).

**Fig. 2:**
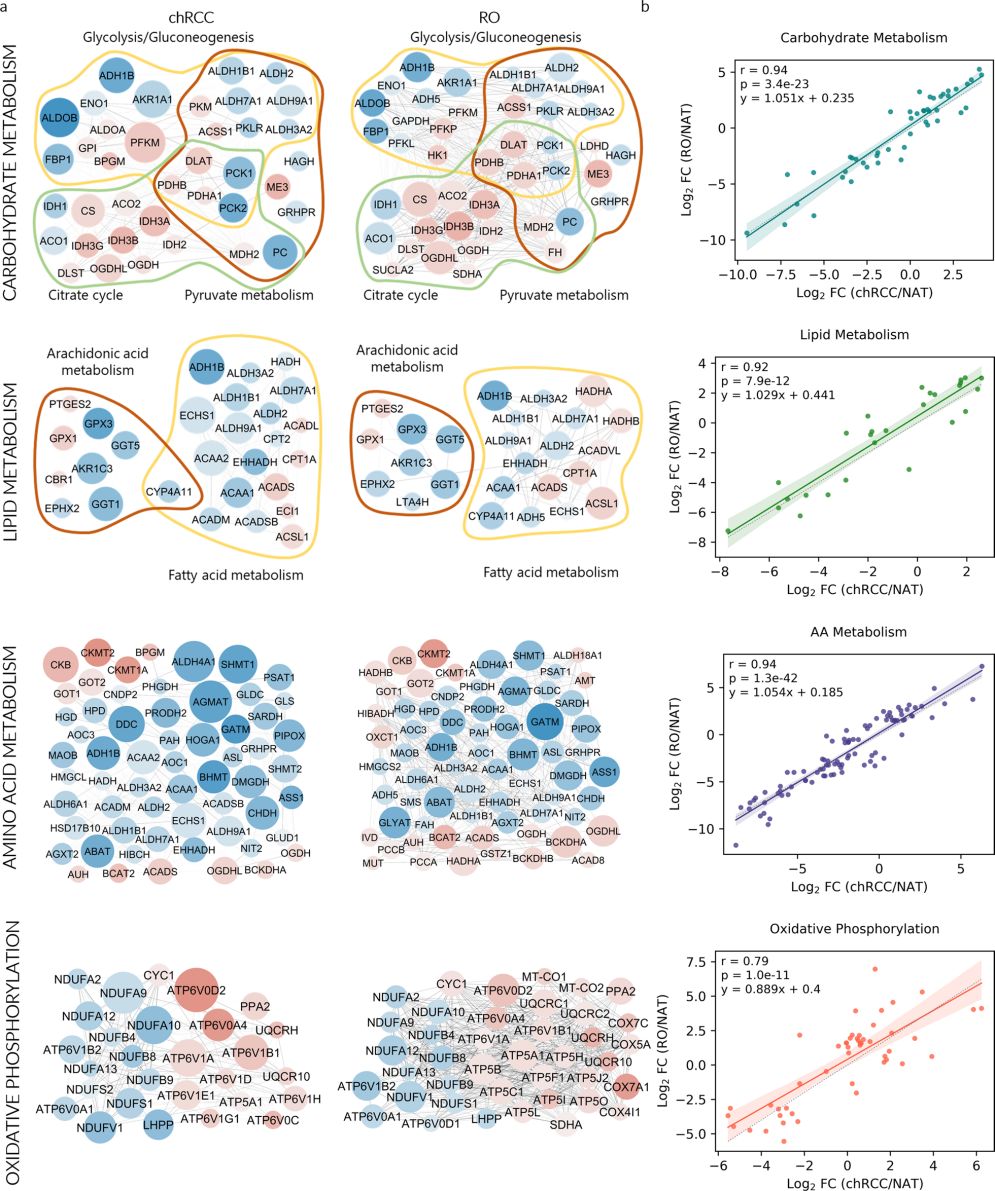
Metabolic pathway proteomes similarly affected in chRCC and RO tumors. **a** Network representation of a subset of pathways in four areas of metabolism in which proteins were differentially regulated in tumors. Significantly (FDR < 0.01) upregulated and downregulated proteins are shown in red and blue, respectively. Protein pathway analysis was performed by searching the KEGG database [19] using differentially expressed proteins between tumor and NAT biopsies. **b** Correlation between levels of dysregulation of individual proteins (log_2_ fold change (FC) in abundance in tumor vs NAT) in chRCC and RO for proteins in carbohydrate (n = 47), lipid (n = 27) and amino acid (n = 91) metabolism and oxidative phosphorylation (n = 47). The correlation coefficient r was calculated using the Pearson test. Shading areas represent the confidence of the interval, and p represents the p value of the test

To elucidate the biochemical processes driving the phenotypes of chRCC and RO, the differentially expressed proteins for each tissue type were used to interrogate comprehensive, functional proteomic databases. We found a common signature of metabolic dysregulation for chRCC and RO compared to NAT (Fig. 2a), affecting carbohydrate metabolism, lipid metabolism, amino acid metabolism and oxidative phosphorylation. Among KEGG protein pathways linked to carbohydrate metabolism, the most dysregulated were glycolysis/gluconeogenesis, pyruvate metabolism and the citric acid cycle (FDR < 8.94 × 10^–14^). In our analysis, we identified up to 23 proteins implicated in these pathways that exhibited dysregulation across both tumor subtypes.

The KEGG glycolysis/gluconeogenesis pathway includes processes involved in the degradation of glucose into pyruvate and the generation of glucose from noncarbohydrates. Based on the differential protein expression data, proteins involved in gluconeogenesis, such as fructose-1,6-bisphosphatase 1 (FBP1), pyruvate carboxylase (PC), and phosphoenolpyruvate carboxykinase 1 and 2 (PCK1 and PCK2), were significantly downregulated in tumors. In contrast, glycolytic proteins remained unchanged or upregulated, e.g., ATP-dependent 6-phosphofructokinase (PFKM). These data indicate that gluconeogenesis was downregulated in tumors compared to NATs. For instance, the results of FBP1 match well with its well-known inhibitory effects on glycolysis and tumor growth [22]. Furthermore, the downregulation of PC, which catalyzes the conversion of pyruvate to oxaloacetate in the first step of gluconeogenesis, suggests a shift in cellular metabolism toward the conversion of pyruvate into acetyl-CoA, as evidenced by the observed upregulation of proteins such as pyruvate dehydrogenase E1 component subunit alpha and beta (PDHA1 and PDHB) and the dihydrolipoyl lysine-residue acetyltransferase component of the pyruvate dehydrogenase complex (DLAT). Several TCA cycle proteins were upregulated, including citrate synthase (CS), aconitate hydratase (ACO2), isocitrate dehydrogenase [NADP] and [NAD] subunits alpha and beta (IDH2, IDH3A and IDH3B), dihydrolipoyl lysine-residue succinyltransferase component of the 2-oxoglutarate dehydrogenase complex (DLST), 2-oxoglutarate dehydrogenase (OGDH) and malate dehydrogenase (MDH2). The expression of proteins active in lipid metabolism was modified in both tumors, with most being downregulated relative to NAT controls. In the chRCC and RO samples, 91 downregulated proteins were linked to amino acid (AA) metabolism.

Pearson correlations were calculated for chRCC and RO based on the fold change in abundance of differentially expressed proteins in carbohydrate, lipid and AA metabolism pathways (Fig. 2b). Positive correlations were found for the four pathways compared, with the oxidative phosphorylation pathway showing the highest p and lowest Pearson correlation values.

### Main chRCC and RO features

The functional protein pathway analysis also revealed divergent features between chRCC and RO that may underlie differences in the tumor subtypes. Proteins involved in energy metabolism of chRCC were less dysregulated than RO. For instance, in chRCC, 28 proteins involved in the oxidative phosphorylation pathway were expressed at different levels compared to NAT, while in RO, 40 proteins belonging to this pathway were dysregulated (Fig. 2a). Comparing the variation between the proteins belonging to the oxidative phosphorylation pathway in both tumors versus NAT, a Pearson coefficient of 0.79 was obtained. The same comparison for carbohydrate, lipid, and AA metabolism resulted in Pearson coefficients of 0.94, 0.92 and 0.94, respectively (Fig. 2b).

**Fig. 3:**
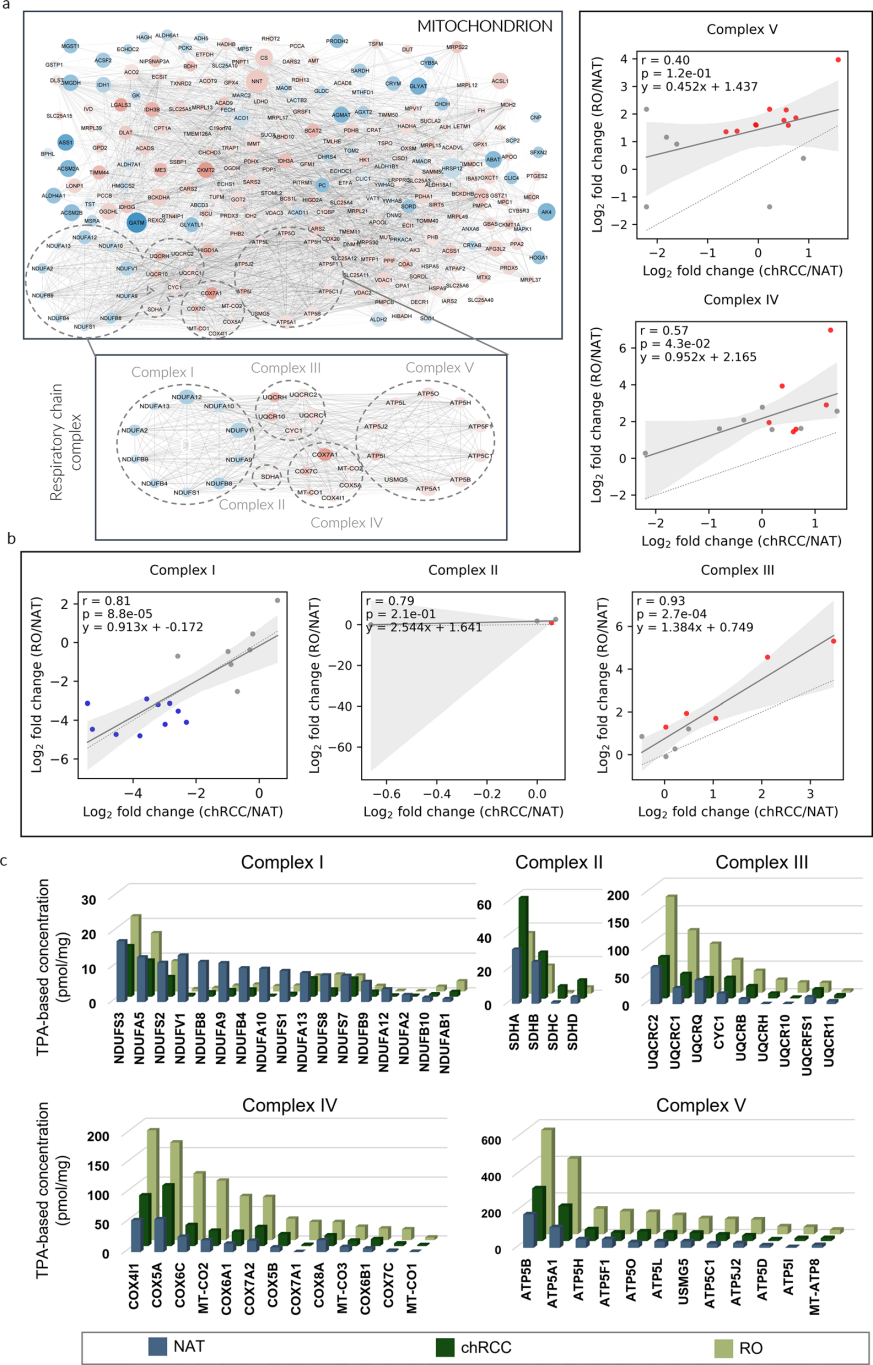
Differences in protein pathways underlying each tumor subtype. **a** Specific dysregulation of mitochondrial protein pathways in ROs, including those of respiratory chain complexes. Protein pathway analysis was performed by using the differentially expressed proteins in each tumor biopsy (relative to NAT) in searches against KEGG [19] and GO [21] databases. Significantly (FDR < 0.01) upregulated and downregulated proteins are shown in red and blue, respectively.** b** Comparison of the protein deregulation (log_2_ fold change (FC) in abundance in tumor relative to NAT) between chRCC and RO for the five respiratory chain complexes [Complex I, n = 44; Complex II, n = 4; Complex III, n = 10; Complex IV, n = 19; Complex V, n = 20]. Blue dots, downregulated proteins (RO vs NAT); red dots, upregulated proteins (RO vs NAT). The correlation coefficient r was calculated using the Pearson test. Shading areas represent the confidence of the interval, and p represents the p value of the test.** c** Absolute protein amount, expressed as pmol/mg of tissue, calculated through the TPA methodology

Mitochondrial KEGG pathways were dysregulated more extensively in RO biopsies, with 256 differentially expressed proteins (Fig. 3a) compared to 175 in chRCC (Additional file 3: Table S2).

The common chRCC versus NAT and RO versus NAT dysregulations of proteins related to respiratory chain complexes were evaluated and compared. The results depicted in Fig. 3b present Pearson correlation coefficients of 0.81, 0.79, 0.93, 0.57, and 0.40 for complexes I, II, III, IV and V, respectively. The TPA-based concentration values for the respiratory chain complex proteins are presented in Fig. 3c, Additional file 4: Figs. S1, 2.

## Discussion

In our research, we utilized the total protein approach to quantify the absolute protein amounts in chRCC, RO, and NAT tissues. These data enabled us to examine the unique protein expression patterns in chRCC and RO compared to NAT and to delve into potential biological implications. In this context, we believed that the most informative approach was to assess and compare the deviations from the normal state in both chRCC and RO. By contrasting these differences, we aimed to elucidate the unique dysregulation patterns associated with each malignancy, shedding light on their molecular characteristics.

PCA of the variance in protein abundance showed that the proteomes of the two tumor subtypes exhibited greater similarity to each other than to the proteomes of NAT samples. This similarity suggests a shared tumor phenotype between the two subtypes. However, PCA also highlighted discernible differences in the quantified proteomes of the two tumor subtypes, indicating unique characteristics associated with each subtype (Fig. 1a).

Comparing dysregulated pathways between chRCC and NAT with those between RO and NAT, as depicted in Fig. 2, reveals a similarity in dysregulation levels. This is evidenced by a Pearson coefficient of 0.92 or higher for carbohydrate metabolism, lipid metabolism, and amino acid metabolism, which strongly suggests parallel patterns of dysregulation in both chRCC and RO subtypes when compared to normal tissue (Fig. 2b). This observation aligns with the well-established hallmark of cancer, namely, the reprogramming of central metabolic pathways. It also corroborates the findings reported by Xiao et al*.* [13] who similarly identified decreased levels of proteins involved in gluconeogenesis and fatty acid metabolism in chRCC compared to NAT. Additionally, our data suggest that gluconeogenesis and lipid metabolism are also reduced in RO compared to NAT, which aligns with the findings reported by Kürschner et al*.* [23].

This collective evidence sheds light on the shared metabolic traits between chRCC and RO, thus confirming common signatures of metabolic dysregulation for chRCC and RO compared to NAT. The evident suppression of amino acid synthesis in both cancer types further highlights the possibility of amino acid auxotrophy as a defining feature of these conditions. The downregulation of argininosuccinate synthetase (ASS1), phosphoglycerate dehydrogenase (PHGDH), and glutamic-oxaloacetic transaminase 1 and 2 (GOT1 and GOT2) in both chRCC and RO tumors compared with NAT confirms this result [24]. Inhibition of this protein leads to decreased production of important amino acids such as arginine, serine and glycine, forcing cancer cells to depend on healthy tissue surrounding the tumor as a source of nutrients [24].

Additionally, the analysis of the oxidative phosphorylation pathway revealed a Pearson coefficient of 0.74 (Fig. 2b), suggesting the presence of distinct mechanisms of oxidative phosphorylation between the chRCC and RO subtypes. Notably, our findings align with previous studies, demonstrating significant alterations in mitochondrial pathways in both RO and chRCC when compared to NAT [25, 26]. It is interesting to note that the levels of dysregulated mitochondrial-related proteins were higher in RO than in chRCC (Fig. 3). Our findings agree with those reported by Joshi et al*.* [25], who suggest that mitochondrial activity acts as a barrier to tumorigenesis in RO, which means that normal mitochondrial activity can prevent or slow down the development of tumors. Reduced mitochondrial activity may contribute to the development of tumors by promoting a protumorigenic environment in cells. This is because normal mitochondrial activity is necessary to maintain healthy cellular metabolism, and altered mitochondrial activity in cancer cells can affect cellular metabolism and promote tumor growth. This suggests that targeting mitochondrial metabolism may be explored as a coadjutant therapeutic strategy for treating chRCC.

In addition to mitochondrial abnormalities, mutations in respiratory chain complex I genes are frequently observed in RO [23]. Our RO versus NAT proteomic data revealed downregulation of complex I-related proteins and predominant upregulation of proteins associated with complexes III, IV and V, as shown in Fig. 3b, c, Additional file 4: Figs. S1, 2. Our data agree with the work of Xiao et al*.* [12], who also reported reduced expression of complex I-related proteins and an increase in all other proteins related to respiratory chain complexes. In this way, the direct difference in the absolute levels of expression of these mitochondrial proteins demonstrates the utility of the TPA in discriminating between these two tumor subtypes.

## Conclusions

In summary, this work presents an in-depth exploration of the contrasting protein expression profiles found in chRCC and RO compared to their normal adjacent tissue. The study used the TPA-based method to determine the absolute amounts of proteins in each tissue type. The findings suggest that both chRCC and RO show dysregulation of carbohydrate, lipid, and amino acid metabolism, as well as mitochondrial abnormalities, when compared to NAT. The study found that the proteomes of the two tumor subtypes were more similar to each other than to NAT, reflecting their shared tumor phenotype. The observed decrease in argininosuccinate synthetase (ASS1) expression in both chRCC and RO tumors relative to adjacent healthy tissue indicates shared metabolic characteristics between these cancers. One such shared trait appears to be auxotrophy, which is prominently featured in both conditions. Targeting mitochondrial metabolism may be explored as a coadjutant therapeutic strategy for treating both chRCC and RO. The reduced number of samples per group used in this study must be considered in future studies.

### Supplementary Information

**Additional file 1: Table S1.** Description of human kidney biopsies used in the study.

**Additional file 2.** Step-by-step protocol, that includes OCT cleaning, proteome extraction, proteome digestion, and LC–MS/MS data acquisition.

**Additional file 3: Table S2.** Protein network analysis.

**Additional file 4: Figure S1.** TPA concentration values (pmol/mg of tissue) of respiratory chain complex I (CI), II (CII) and III (CIII)proteins. Statistical analysis was performed using pairwise Mann Whitney test (**p ≤ 0.001; ***p ≤ 0.0001; ****p ≤ 0.00001). **Figure S2.** TPA concentration values (pmol/mg of tissue) of respiratory chain complex IV (CIV) and V (CV) proteins. Statistical analysis was performed using pairwise Mann Whitney test (**p ≤ 0.001; ***p ≤ 0.0001;****p ≤ 0.00001).

## Data Availability

The mass spectrometry proteomics raw data have been deposited in the ProteomeXchange Consortium via the PRIDE [27] partner repository with the dataset identifier PXD022018.

## Abbreviations

chRCC: Chromophobe renal cell carcinoma
LFQ: Label-free quantification
nanoLC-HR-MS/MS: Nanoliquid chromatography-high resolution-tandem mass spectrometry
NAT: Normal adjacent tissue
OCT: Optimum cutting temperature
PCA: Principal component analysis
RCC: Renal cell carcinoma
RO: Benign renal oncocytoma
