# Supplementary material for: Proteomic analysis of chromophobe renal cell carcinoma and benign renal oncocytoma biopsies reveals shared metabolic dysregulation
Source: Clin Proteomics. 2023 Nov 28;20:54. doi: 10.1186/s12014-023-09443-8 (PMC10683195; doi:10.1186/s12014-023-09443-8)
Supplement: Supplementary file 1 — Additional file 1: Table S1. Description of human kidney biopsies used in the study. [file 12014_2023_9443_MOESM1_ESM.pdf]

Additional file 1: Table S1. Description of human kidney biopsies used in the study.

BIOPSY	AGE	GENDER	DIAGNOSIS*	SAMPLE TYPE*
**N1**	54	Male	RCC	NAT
**N2**	49	Female	Papillary	NAT
**N3**	58	Female	RCC	NAT
**N4**	72	Female	RCC	NAT
**N5**	70	Male	RCC	NAT
**C6**	73	Male	RCC	chRCC
**C7**	67	Female	RCC	chRCC
**C8**	71	Male	RCC	chRCC
**C9**	58	Female	RCC	chRCC
**C10**	81	Male	RCC	chRCC
**O11**	80	Male	RCC	RO
**O12**	69	Female	RCC	RO
**O13**	63	Male	RCC	RO
**O14**	62	Female	RCC	RO
**O15**	55	Female	RCC	RO

*RCC: renal cell carcinoma; NAT: normal adjacent tissue; chRCC: chromophobe renal cell carcinoma; RO: renal oncocytoma
