# Supplementary material for: Proteomic analysis of chromophobe renal cell carcinoma and benign renal oncocytoma biopsies reveals shared metabolic dysregulation
Source: Clin Proteomics. 2023 Nov 28;20:54. doi: 10.1186/s12014-023-09443-8 (PMC10683195; doi:10.1186/s12014-023-09443-8)
Supplement: Supplementary file 2 — Additional file 2. Step-by-step protocol, that includes OCT cleaning, proteome extraction, proteome digestion, and LC–MS/MS data acquisition. [file 12014_2023_9443_MOESM2_ESM.pdf]

Additional file 2: Material 1

Proteomic analysis of chromophobe renal cell carcinoma and benign renal oncocytoma biopsies reveals shared metabolic dysregulation.

Luis B. Carvalho[1,2 *], Susana Jorge[1,2 *], Hugo López-Fernández[3,4], Carlos Lodeiro[1,2], Rajiv Dhir[5], Luis Campos Pinheiro[6,7], Mariana Medeiros[6,7], Hugo M. Santos[1,2,5], José L. Capelo[1,2 **]

[1] BIOSCOPE Research Group, LAQV-REQUIMTE, Department of Chemistry, NOVA School of Science and Technology, FCT NOVA, Universidade NOVA de Lisboa, 2829-516 Caparica, Portugal.

[2] PROTEOMASS Scientific Society, Departamental Building, FCT-NOVA, Caparica Campus, 2829-516 Caparica, Portugal.

[3] CINBIO, Department of Computer Science, ESEI-Escuela Superior de Ingeniería Informática, Universidade de Vigo, Ourense, 32004, Spain.

[4] SING Research Group, Galicia Sur Health Research Institute (IIS Galicia Sur), SERGAS-UVIGO, Vigo, 36213, Spain

[5] Department of Pathology, University of Pittsburgh Medical Center, Pittsburgh, PA, United States.

[6] Urology Department, Central Lisbon Hospital Center, Lisbon, Portugal.

[7] NOVA Medical School, NOVA University of Lisbon, Lisbon, Portugal

Contents of Additional file 2: Material 1

Reagents. 2
Materials and equipment 2
Step-by-Step Protocol for OCT Cleaning of Human Kidney Tumors. 3
Step-by-Step Protocol for Proteome Extraction from Human Kidney Tumors. 4
Step-by-Step Protocol for Proteome Clean-up by Precipitation with DOC/TCA. 4
Step-by-Step Protocol for Proteome Digestion. 5
Nano-LC-ESI-MS/MS analysis. 5

Reagents.

All reagents used are from the highest purity available. Urea, Bradford reagent, iodoacetamide (IAA), sodium deoxycholate (DOC), acetone, and trichloroacetic acid (TCA) were purchased from Sigma-Aldrich (Basel, Switzerland). Ammonium bicarbonate (AmBic) was purchased from Fluka (Basel, Switzerland). Pierce™ Trypsin protease and trifluoroacetic acid (TFA) were purchased from Thermo Fischer Scientific (Waltham, MA, USA). Acetonitrile (ACN), formic acid (FA) and ethanol were purchased from Carlo Erba Reagents (Val de Reuil, France). Dithiothreitol (DTT) was purchased from Alfa Aesar (Karlsruhe, Germany), sodium hydroxide (NaOH) was from Panreac AppliChem (Barcelona, Spain).

Materials and equipment.

Protein digestion was performed in Eppendorf safe-lock tubes of 0.5mL volume (Hamburg, Germany). A vacuum concentrator centrifuge model UNIVAPO 150 ECH Speed Vac and a vacuum pump model UNIJET II (Munich, Germany) were used for sample drying and sample pre-concentration. A mini incubator from Labnet (New Jersey, USA) was used for protein reduction steps. Vortexer models ELMI CM70M-09 SkyLine (Southern California, USA), and Prism™ R Refrigerated Microcentrifuge and VX-200 Lab0020 Vortex Mixer from Labnet (New Jersey, USA), were used throughout the sample treatment. CLARIOstar® High Performance Monochromator Multimode from BMG LABTECH (Germany) was used for Bradford assays. An ultrasonic bath, model TI-H-5, from Elma (Singen, Germany) with control of temperature and amplitude was used to enhance OCT cleaning steps, an ultrasonic processor UP50H (50 W, 30 kHz, 1 mm diameter probe tip) from Hielscher Ultrasonics (Teltow, Germany) was utilized for tissue homogenization, and a Microplate Horn Assembly operating with the Q700 system (20 kHz) from QSonica (Newtown, CT, USA) was employed to accelerate enzymatic digestions. Mass spectrometry data was acquired using an UHR-QqTOF IMPACT HD from Bruker Daltonics (Bremen, Germany). Chromatographic separation of peptides was carried out using an Ultimate 3000 nLC nano-system equipped with a trap-column Acclaim PepMap100, 5 µm, 100 Å, 300 µm i.d. × 5 mm (Thermo Fisher Scientific) and an analytical column Acclaim™ PepMap™ 100 C18, 2 µm, 0.075 mm i.d x 150 mm (Thermo Fisher Scientific).

Step-by-Step Protocol for OCT Cleaning of Human Kidney Tumors.

Note: Adherence to established safety protocols, including the appropriate use of Personal Protection Equipment (PPE), is of utmost importance when conducting laboratory procedures, especially when handling biological samples. Always consult and comply with relevant safety documentation, institutional guidelines, and regulatory mandates to ensure laboratory activities are executed within a secure and responsible framework.

Step 1: Thawing the Samples

1.1 Remove the human kidney biopsies embedded in OCT from the -80°C freezer and place each sample in a sterile petri dish.

1.2 Allow them to thaw at room temperature until OCT is completely melted.

Step 2: Removing Excess OCT

2.1 Gently remove excess OCT from around the biopsies using a clean scalpel or a spatula. Note: Ensure that the biopsies are not damaged during the removal of OCT.

2.2 Transfer the tissue sample into clean 1.5 mL microtubes. *Note: Depending on the tissue size, larger Falcon tubes might be needed.*

Step 3: Ultrasonic Cleaning

3.1 Add enough 70% (v/v) ethanol, pre-chilled to 4 °C, to cover the tissue biopsies completely. Use 10 mL of 70% (v/v) ethanol per 1g of tissue sample, adjusting accordingly to the wet weight of your sample.

3.2 Place the samples into an Ultrasonic Bath model TI-H-5, from Elma (Singen, Germany) and sonicate the samples at 35 kHz for 2 min at 100% ultrasonic amplitude.

3.3 Centrifuge the samples at 4 °C for 2 minutes at 5,000×g.

3.4 Carefully remove the70% (v/v) ethanol supernatant.

3.5 Repeat steps 3.1 to 3.4 once more.

3.6 Add enough Milli-Q water, pre-chilled to 4 °C, to cover the tissue biopsies completely. Use 10 mL of Milli-Q water per 1g of tissue sample, adjusting accordingly to the wet weight of your sample.

3.7 Place the samples into an Ultrasonic Bath model TI-H-5, from Elma (Singen, Germany) and sonicate the samples at 35 kHz for 2 min at 100% ultrasonic amplitude.

3.8 Centrifuge the samples at 4 °C for 2 minutes at 5,000×g.

3.9 Carefully remove the Milli-Q water supernatant.

3.10 Repeat steps 3.6 to 3.9 five times.

Step-by-Step Protocol for Proteome Extraction from Human Kidney Tumors.

Ensure that the biopsies are handled with clean, sterilized tools to prevent any contamination during the freezing and powdering process.

1.1 Immediately freeze the biopsies using liquid nitrogen upon completing all cleaning steps. *Note: Ensure the kidney sample is fully submerged or adequately covered with liquid nitrogen to facilitate rapid freezing.*

1.2 Once the kidney sample is thoroughly frozen, utilize a mortar and pestle or a mechanical grinder to reduce the tissue sample to a powder. *Note: Ensure that the biopsies remain adequately frozen during the powdering process. Utilize liquid nitrogen as needed to prevent thawing.*

1.3 Add the appropriate volume of extraction buffer (8M Urea in 25 mM Ammonium Bicarbonate, 10 mM DTT) Use 10 mL of extraction buffer per 1g of tissue sample, adjusting accordingly to the wet weight of your sample. *Note: Always prepare fresh DTT.*

1.4 Vortex samples for 1 min.

1.5 Utilize an ultrasonic processor UP50H (50 W, 30 kHz, 1 mm diameter probe tip) for protein extraction. Operate the processor at 100% ultrasonic amplitude for 2 min in pulsed mode (10 sec on; 10 sec off).

1.6 Centrifuge the sample at 10,000×g for 10 min.

1.7 After centrifugation, collect the supernatant into new microtubes.

1.8 Repeat steps 1.3 and 1.7 two more times on the same sample. Combine the supernatants into the microtube prepared in step 1.6.

Step-by-Step Protocol for Proteome Clean-up by Precipitation with DOC/TCA.

1.1 Add 1 µL of 2% (w/v) Deoxycholate (DOC) to each 100 µL of proteome extract and incubate on ice for 30 min.

1.2 Add 25 µL of 100% (w/v) of Trichloroacetic acid (TCA) to the mixture and leave the samples on ice for an additional 20 min.

1.3 Centrifuge the samples at 16,000×g for 20 min at 4 °C.

1.4 Remove the supernatant, retaining the proteome pellet.

1.5 Wash the pellets with 200 µL of cold acetone (-20 ºC).

1.6 Centrifuge the samples at 16,000×g for 20 min at 4 °C.

1.7 Add 20 µL of 0.2 M NaOH to the protein pellet.

1.8 Incubate the pellet for 2 min at room temperature.

1.9 Add 80 µL of 6 M urea in 25 mM Ammonium Bicarbonate to the pellet.

1.10 Dissolve the protein pellet using the ultrasonic processor UP50H (50 W, 30 kHz, 1 mm diameter probe tip) operating at 50% ultrasonic amplitude. Perform four cycles of 10 sec of ultrasonic energy, with 5-second intervals between each cycle.

1.11 Determine the total protein content of each sample (n=3) using the Bradford protein assay.

Step-by-Step Protocol for Proteome Digestion.

1.1 Pipette 20 µL of the proteome extract from your previous experiment into new 0.5 mL low-bind microtubes. Add 2 µL of 110 mM DTT (Dithiothreitol) to the 20 µL of supernatant.

1.2 Vortex the mixture thoroughly to ensure uniform mixing.

1.3 Incubate the mixture for 30 minutes at 37 °C in a mini-incubator from Labnet (New Jersey, USA).

1.4 Add 2 µL of 600 mM IAA (Iodoacetamide) to the reduced protein sample. Vortex the sample thoroughly to ensure even distribution of IAA.

1.5 Incubate the sample for 30 minutes at room temperature, ensuring it is kept in the dark to prevent IAA degradation.

1.6 Dilute the alkylated sample to a final volume of 100 µL using 76 µL of 25 mM Ammonium Bicarbonate buffer. Mix thoroughly to ensure uniform dilution.

1.7 Measure and set 50 µg of total protein from your sample in new low-bind microtubes.

1.8 Add trypsin at a 1:20 (w/w) ratio. Typically, for 50 µg of total protein, use 2.5 µg of Trypsin MS-grade.

1.9 Perform trypsin digestion using a microplate horn assembly operating with the Q700 system (20 kHz) from QSonica (Newtown, CT, USA) using the following conditions: 25% ultrasonic amplitude, 4 minutes ultrasonic duty time, Pulsed mode: 30 seconds on, 15 seconds off

1.10 Add formic acid to the digested sample to achieve a final concentration of 0.1% (v/v).

1.11 Mix thoroughly to ensure an even distribution of formic acid.

1.12 Evaporate the samples to dryness using a vacuum concentrator centrifuge model UNIVAPO 150 ECH Speed Vac and a vacuum pump model UNIJET II (Munich, Germany).

Nano-LC-ESI-MS/MS analysis.

The LC-MS/MS analysis was conducted using an Ultimate 3000 nLC coupled to an UHR-QqTOF IMPACT HD (Bruker Daltonics) with a CaptiveSpray ion source (Bruker Daltonics). All samples were reconstituted to a final peptide concentration of 0.25 µg/µL in 3% ACN/0.1% (v/v) aqueous formic acid. Three µL of each sample was loaded into a trap column Acclaim PepMap100, 5 µm, 100 Å, 300 µm i.d. × 5 mm and desalted for 5 min with 3% B (B: 90% ACN/0.1% FA) at a flow rate of 15 µL/min. Chromatographic separation was carried out using an analytical column Acclaim™ PepMap™ 100 C18, 2 µm, 0.075 mm i.d x 150 mm with a linear gradient at 300 nL/min (mobile phase A: aqueous FA 0.1% (v/v); mobile phase B 90% (v/v) ACN and 0.08% (v/v) FA), 0-5 min with 3% of mobile phase B, 5-95 min from 3% to 35% of mobile phase B, 95-105 min linear gradient from 35% to 95% of mobile phase B, 105-115 with 95% of mobile phase B. For each sample, two replicate injections were performed. Chromatographic separation was carried out at 35 ºC. MS acquisition was set to cycles of MS (2 Hz), followed by MS/MS (8–32Hz), cycle time 3.0 seconds, with active exclusion (precursors were excluded from precursor selection for 0.5 min after acquisition of 1 MS/MS spectrum, intensity threshold for fragmentation of 2500 counts). Together with active exclusion set to 1, reconsider the precursor if the intensity of a precursor increases by a factor of 3, this mass will be taken from the temporary exclusion

list and fragmented again, ensuring that fragment spectra were taken near the peak maximum. All spectra were acquired in the range 150–2200 m/z.
