# Supplementary material for: Proteomic analysis of chromophobe renal cell carcinoma and benign renal oncocytoma biopsies reveals shared metabolic dysregulation
Source: Clin Proteomics. 2023 Nov 28;20:54. doi: 10.1186/s12014-023-09443-8 (PMC10683195; doi:10.1186/s12014-023-09443-8)
Supplement: Supplementary file 3 — Additional file 3: Table S2. Protein network analysis. [file 12014_2023_9443_MOESM3_ESM.pdf]

Additional file 3: Table S2. - Protein network analysis

<table>
<tr><th colspan="9">chRCC</th></tr>
<tr><th># background genes</th><th># genes</th><th>category</th><th>description</th><th>FDR value</th><th>genes</th><th>p-value</th><th>term name</th><th>transferred FDR value</th></tr>
<tr><td>1779</td><td>240</td><td>GO Process</td><td>small molecule metabolic process</td><td>1.4E-97</td><td>DCN|HSD17B10|NDUFB4|NANS|ACO2|GNPNAT1|MTHFD1|PCK2|AHCY|IDH3G|CRYM|ECH1|OGDH|PDHX|ACOT13|DNPH1|AGXT2|EHHADH|ACADL|ATP6V1B1|ENO1|AKR7A2|APOA1|TTR|GDA|ACADS|PEPD|GOT2|PMPCB|NDUFA2|NDUFA10|HRSP12|BHMT2|NARS|GGH|KHK|HEXB|ALDH2|DHTKD1|MECR|LRP2|ATP6V0A1|NNT|SCARB2|ACAD11|PRDX5|ALDH8A1|CPT1A|REXO2|NDUFA9|SORD|SLC27A2|BCKDHA|ABHD10|ATP6V1A|BHMT|ATP6V1B2|ERLIN2|DPYS|NDUFB9|GLUD1|HNMT|DLAT|QDPR|GPD1L|HGD|DDAH1|ACAA2|HPD|CBR1|FTCD|CRYL1|HPRT1|AMN|NDUFB8|CKB|IDH3A|GLYATL1|GPD1|PRODH2|ECI1|GUSB|SLC23A1|DCXR|HINT1|UGT2B7|GAA|ADH1B|ASL|PDHB|CYCS|ATP6V0A4|GPD2|ACO1|UQCRH|CYP4A11|AQP1|GBAS|GRHPR|MSRA|AHCYL2|ACSS1|CYC1|GLS|PIPOX|CES2|SHMT1|UGDH|PCK1|PKM|SLC7A8|UGT2B17|TALDO1|NDUFV1|BCAT2|DHRS4|MDH2|ACSM2B|NDUFA12|IDH2|UQCR10|ACAA1|SHMT2|BSG|DLST|GSTA1|PKLR|CYB5A|CS|PPA2|APOD|PTGES2|PFKM|ALDH3A2|UGT1A9|WARS|VCP|HIBCH|PNP|OPA1|MARC2|ATP1B1|MGST3|NDUFS2|ECHS1|LHPP|ACADSB|PHGDH|GSTO1|ABCD3|GOT1|HOGA1|ACADM|RBP4|CPT2|SCP2|ECHDC2|SARDH|CMPK1|AKR1A1|ASS1|PRPS1|OGDHL|HMGCL|ALDOB|ALDH4A1|VARS|AUH|AGMAT|PSAT1|ALDH1B1|CUBN|APRT|ACOT9|PDHA1|BPHL|AKR1C3|IDH3B|GLDC|BDH1|DPEP1|BPGM|NME2|CNP|PC|NIT2|DAK|ALDOA|AK4|QPRT|AMPD3|ABAT|GATM|MPST|HAGH|GGT5|ATP5A1|GGT1|ACY1|PTGR1|TST|ALDH7A1|SLC25A12|IDH1|TKT|NDUFS1|RHOA|GK|ACSF2|DDC|CKMT2|GPI|CKMT1A|GPX1|FBP1|LARS2|MME|ABHD14B|ACSL1|ETFDH|EPHX2|ECHDC1|ME3|MMAB|RAN|SLC25A10|PAH|ACSS3|ALDH6A1|NPC2|PTGR2|ACSM2A|GALK1|HADH|EPHX1|FMO1|PLCG2|GALE|HAO2|GLYAT</td><td>2.8E-101</td><td>GO.0044281</td><td>9.6844664</td></tr>
</table>

11238	495	GO Component	cytoplasm	3.2E-75	RALA|SYPL1|HEBP1|PSMA4|DCN|GNA11|HSD17B10|NDUFB4|EPDR1|NANS|VCL|NIPSNAP1|MYH9|ACO2|GNPNAT1|ATP6V1D|MTHFD1|PCK2|PYGB|AHCY|APMAP|TXNL1|MYL12A|IDH3G|F9|PSMD7|CRYM|CTSH|EHD4|ECH1|HNRNPL|PLIN3|CDC37|OGDH|FIS1|SFXN3|PPIF|PDHX|SLC25A3|ACOT13|DNPH1|AGXT2|LTF|EHHADH|REEP6|ACADL|ATP6V1B1|ENO1|AKR7A2|CTSD|APOA1|TTR|GDA|PFN2|PLBD1|TFG|ACADS|GOT2|CAPNS1|CANX|VIL1|HSPB1|PMPCB|RPS16|NDUFA2|ACTN4|NDUFA10|ATP6V1E1|NAPSA|LGALS3|GRSF1|HRSP12|DMGDH|BHMT2|SYNJ2BP|ARL8B|GSTM3|NARS|GGH|SQRDL|KHK|RAB21|LYZ|HEXB|PSMD11|ALDH2|CDH1|RAB11A|DNAJA3|SLC4A1|COTL1|CHCHD3|SLC9A3R1|RAB2A|PRDX1|DHTKD1|BBOX1|PACSIN2|NAPA|MECR|LRP2|CAPG|GRB14|RAB17|PSMD3|ATP6V0A1|NNT|ADD1|F13A1|SCARB2|ACAD11|RAB7A|ENPEP|VDAC1|CALB1|PRDX5|ALDH8A1|CPT1A|REXO2|NDUFA9|RBP5|SORD|SLC27A2|RHCG|IQGAP1|AFG3L2|ARHGDIA|BCKDHA|ECSIT|TINAGL1|PSMA5|ARL8A|NAT8|ABHD10|ATP6V1A|FTH1|BHMT|ATP6V1B2|ERLIN2|DPYS|NDUFB9|GLUD1|ENDOD1|LAMTOR1|HNMT|DLAT|QDPR|SLC25A4|GPD1L|HGD|DDAH1|UCHL1|ACAA2|CA2|ATP6V0D2|HPD|CBR1|PSMB4|FTCD|TBC1D24|AGL|CAPN2|RNPEP|FABP1|APEH|CMBL|SCIN|HSPA9|COL14A1|CFL2|CRYL1|HPRT1|AMN|NDUFB8|CKB|TPP1|IDH3A|ANPEP|GLYATL1|COL6A2|GPD1|PRODH2|ECI1|GUSB|SLC23A1|RPS9|TMED10|DCXR|HINT1|UGT2B7|GNB2|GAA|YWHAG|ADH1B|TMEM126A|ASL|PDHB|CYCS|ATP6V0A4|GPD2|ACO1|DPYSL2|UQCRH|RAB1B|CYP4A11|AQP1|RAB6A|AOC3|GBAS|HNRNPD|DAB2|GRHPR|TSFM|MSRA|TOLLIP|AHCYL2|TLN1|HSD11B2|SFXN1|ACSS1|CYC1|GLS|PIPOX|CES2|SHMT1|UGDH|PCK1|CHDH|PKM|SLC7A8|UGT2B17|TALDO1|RHOT2|NDUFV1|BCAT2|CNDP2|DHRS4|MDH2|EHD3|ACSM2B|AP2A2|KRT7|COPB2|ATP6V0C|NDUFA12|TMED9|IDH2|UQCR10|SERPINA5|LAMP1|RAB11B|ACAA1|SHMT2|BSG|PARVA|DLST|GSTA1|TRIM2|PKLR|CYB5A|CS|LAMTOR4|EPB41L3|PPA2|DHRS2|APOD|PTGES2|VAPA|CTSB|PFKM|ALDH3A2|ANXA6|SND1|UGT1A9|ALDH9A1|WARS|AGK|COL18A1|PLS3|GM2A|UBE2D3|PBLD|VCP|ATP6V1H|HIBCH|MSN|MYH10|COL4A2|DPP4|LONP1|MRPL37|MRPL24|PNP|MRPL21|OPA1|COA3|KIF21A|KRT19|COL6A1|AKR7A3|IBA57|MARC2|CYB5R1|MGST3|NDUFS2|TAGLN2|ECHS1|LHPP|ACADSB|PHGDH|GSTO1|FLNA|SFXN2|MYO6|ABCD3|RPL5|GOT1|HOGA1|ACADM|RBP4|CPT2|SCP2|ECHDC2|SLC25A5|SARDH|CMPK1|AKR1A1|ASS1|PRPS1|APOOL|GSN|RAB14|ATP6V1G1|OGDHL|CLIC4|HMGCL|HNRNPR|HSPG2|ALDOB|PSMB9|PSMB8|ALDH4A1|ASPN|VARS|AUH|CLIC1|COL4A1|AGMAT|PSAT1|TBC1D4|ALDH1B1|CUBN|SLC3A2|RSU1|CKAP4|MAOB|APRT|ACOT9|TXNDC5|PDHA1|MT1M|BPHL|NQO2|NAP1L4|AKR1C3|IDH3B|GLDC|SLC25A6|OCIAD1|ASAH1|GPNMB|CD9|FECH|KRT18|SACM1L|BDH1|PRKAR1A|BPGM|NME2|CNP|PC|NIT2|FHL1|ACTN1|PRKCD|DAK|PSAP|ALDOA|KTN1|AK4|QPRT|CD59|UMOD|MGST1|MYH11|AMPD3|ABAT|GATM|ILK|MPST|HAGH|ATP5A1|SEPT7|TXNRD2|ACY1|IFI30|ELAVL1|PDIA6|PTGR1|TST|RMDN1|ALDH7A1|IMMT|UBA52|SLC25A12|SCRN1|IDH1|TKT|NDUFS1|GDI1|HIGD1A|ANXA11|CRYZ|RHOA|GK|ACSF2|DDC|TANGO2|TMED4|CKMT2|GPI|CKMT1A|GPX1|FBP1|LAMP2|LARS2|TOMM40|AOC1|TOM1|SERPINA1|TGFBI|VWA1|MRPS22|MME|GFM1|FAM162A|PPIA|SSBP1|ABHD14B|GSTA2|FLNB|TMEM33|ACSL1|OCIAD2|NDUFA13|ETFDH|WDR1|KIF13B|EPHX2|CAPN1|TAGLN|ECHDC1|ME3|PHB2|ATP1A1|MMAB|RAN|SLC25A10|RPL18|MGAM|CD63|PAH|KR[illegible]	4.94E-78	GO.0005737	7.4493495
17	12	KEGG Pathways	2-Oxocarboxylic acid metabolism	6.9E-11	ACO2|IDH3G|GOT2|IDH3A|ACO1|BCAT2|IDH2|CS|GOT1|IDH3B|ACY1|IDH1	3.31E-12	hsa01210	1.01617808

854	156	GO Process	carboxylic acid metabolic process	1.8E-74	DCN|HSD17B10|ACO2|MTHFD1|AHCY|IDH3G|CRYM|ECH1|OGDH|PDHX|AGXT2|EHHADH|ACADL|ENO1|ACADS|PEPD|GOT2|HRSP12|BHMT2|NARS|GGH|HEXB|DHTKD1|MECR|NNT|SCARB2|ACAD11|ALDH8A1|CPT1A|SORD|SLC27A2|BCKDHA|ABHD10|BHMT|DPYS|GLUD1|HNMT|DLAT|QDPR|HGD|DDAH1|ACAA2|HPD|CBR1|FTCD|CRYL1|CKB|IDH3A|GLYATL1|PRODH2|ECI1|GUSB|SLC23A1|DCXR|UGT2B7|ASL|PDHB|ACO1|CYP4A11|GRHPR|MSRA|AHCYL2|ACSS1|GLS|PIPOX|CES2|SHMT1|UGDH|PCK1|PKM|SLC7A8|UGT2B17|BCAT2|MDH2|ACSM2B|IDH2|ACAA1|SHMT2|BSG|DLST|GSTA1|PKLR|CYB5A|CS|PPA2|PTGES2|PFKM|ALDH3A2|UGT1A9|WARS|HIBCH|MGST3|ECHS1|ACADSB|PHGDH|GSTO1|ABCD3|GOT1|HOGA1|ACADM|CPT2|SCP2|ECHDC2|SARDH|AKR1A1|ASS1|OGDHL|HMGCL|ALDOB|ALDH4A1|VARS|AUH|AGMAT|PSAT1|PDHA1|BPHL|AKR1C3|IDH3B|GLDC|DPEP1|BPGM|PC|NIT2|ALDOA|QPRT|ABAT|GATM|MPST|HAGH|GGT5|GGT1|ACY1|PTGR1|TST|ALDH7A1|IDH1|ACSF2|DDC|CKMT2|GPI|CKMT1A|GPX1|LARS2|ACSL1|ETFDH|EPHX2|ECHDC1|ME3|PAH|ALDH6A1|PTGR2|ACSM2A|GALK1|HADH|HAO2|GLYAT	6.92E-78	GO.0019752	7.3756962
959	161	GO Process	organic acid metabolic process	1.3E-72	DCN|HSD17B10|ACO2|MTHFD1|AHCY|IDH3G|CRYM|ECH1|OGDH|PDHX|AGXT2|EHHADH|ACADL|ENO1|ACADS|PEPD|GOT2|HRSP12|BHMT2|NARS|GGH|HEXB|DHTKD1|MECR|NNT|SCARB2|ACAD11|ALDH8A1|CPT1A|SORD|SLC27A2|BCKDHA|ABHD10|BHMT|DPYS|GLUD1|HNMT|DLAT|QDPR|HGD|DDAH1|ACAA2|HPD|CBR1|FTCD|CRYL1|CKB|IDH3A|GLYATL1|PRODH2|ECI1|GUSB|SLC23A1|DCXR|UGT2B7|ASL|PDHB|ACO1|CYP4A11|GRHPR|MSRA|AHCYL2|ACSS1|GLS|PIPOX|CES2|SHMT1|UGDH|PCK1|PKM|SLC7A8|UGT2B17|BCAT2|MDH2|ACSM2B|IDH2|ACAA1|SHMT2|BSG|DLST|GSTA1|PKLR|CYB5A|CS|PPA2|PTGES2|PFKM|ALDH3A2|UGT1A9|WARS|HIBCH|PNP|MARC2|MGST3|ECHS1|ACADSB|PHGDH|GSTO1|ABCD3|GOT1|HOGA1|ACADM|CPT2|SCP2|ECHDC2|SARDH|AKR1A1|ASS1|PRPS1|OGDHL|HMGCL|ALDOB|ALDH4A1|VARS|AUH|AGMAT|PSAT1|PDHA1|BPHL|AKR1C3|IDH3B|GLDC|DPEP1|BPGM|PC|NIT2|ALDOA|QPRT|ABAT|GATM|MPST|HAGH|GGT5|GGT1|ACY1|PTGR1|TST|ALDH7A1|IDH1|ACSF2|DDC|CKMT2|GPI|CKMT1A|GPX1|LARS2|ABHD14B|ACSL1|ETFDH|EPHX2|ECHDC1|ME3|PAH|ALDH6A1|PTGR2|ACSM2A|GALK1|HADH|FMO1|HAO2|GLYAT	7.47E-76	GO.0006082	7.18996295
943	158	GO Process	oxoacid metabolic process	4.4E-71	DCN|HSD17B10|ACO2|MTHFD1|AHCY|IDH3G|CRYM|ECH1|OGDH|PDHX|AGXT2|EHHADH|ACADL|ENO1|ACADS|PEPD|GOT2|HRSP12|BHMT2|NARS|GGH|HEXB|DHTKD1|MECR|NNT|SCARB2|ACAD11|ALDH8A1|CPT1A|SORD|SLC27A2|BCKDHA|ABHD10|BHMT|DPYS|GLUD1|HNMT|DLAT|QDPR|HGD|DDAH1|ACAA2|HPD|CBR1|FTCD|CRYL1|CKB|IDH3A|GLYATL1|PRODH2|ECI1|GUSB|SLC23A1|DCXR|UGT2B7|ASL|PDHB|ACO1|CYP4A11|GRHPR|MSRA|AHCYL2|ACSS1|GLS|PIPOX|CES2|SHMT1|UGDH|PCK1|PKM|SLC7A8|UGT2B17|BCAT2|MDH2|ACSM2B|IDH2|ACAA1|SHMT2|BSG|DLST|GSTA1|PKLR|CYB5A|CS|PPA2|PTGES2|PFKM|ALDH3A2|UGT1A9|WARS|HIBCH|MARC2|MGST3|ECHS1|ACADSB|PHGDH|GSTO1|ABCD3|GOT1|HOGA1|ACADM|CPT2|SCP2|ECHDC2|SARDH|AKR1A1|ASS1|OGDHL|HMGCL|ALDOB|ALDH4A1|VARS|AUH|AGMAT|PSAT1|PDHA1|BPHL|AKR1C3|IDH3B|GLDC|DPEP1|BPGM|PC|NIT2|ALDOA|QPRT|ABAT|GATM|MPST|HAGH|GGT5|GGT1|ACY1|PTGR1|TST|ALDH7A1|IDH1|ACSF2|DDC|CKMT2|GPI|CKMT1A|GPX1|LARS2|ABHD14B|ACSL1|ETFDH|EPHX2|ECHDC1|ME3|PAH|ALDH6A1|PTGR2|ACSM2A|GALK1|HADH|HAO2|GLYAT	3.49E-74	GO.0043436	7.03545777

1420	178	Reactome Pathways	Metabolism	6.3E-65	DCN|HSD17B10|CA12|MTHFD1|PCK2|PYGB|AHCY|CRYM|PLIN3|ACOT13|DNPH1|AGXT2|EHHADH|ACADL|ENO1|AKR7A2|TTR|GDA|PLBD1|ACADS|GOT2|HRSP12|DMGDH|BHMT2|GSTM3|SQRDL|KHK|HEXB|DHTKD1|BBOX1|MECR|LRP2|ACAD11|SORD|SLC27A2|IQGAP1|BCKDHA|ABHD10|BHMT|DPYS|GLUD1|HNMT|QDPR|GPD1L|HGD|DDAH1|ACAA2|HPD|CBR1|FTCD|AGL|FABP1|CMBL|CRYL1|HPRT1|AMN|CKB|GLYATL1|GPD1|PRODH2|ECI1|GUSB|SLC23A1|DCXR|UGT2B7|GAA|ADH1B|ASL|GPD2|CYP4A11|AOC3|GRHPR|HSD11B2|ACSS1|PIPOX|CES2|SHMT1|UGDH|CHDH|UGT2B17|BCAT2|CNDP2|ACSM2B|ACAA1|SHMT2|GSTA1|CYB5A|PPA2|PTGES2|PFKM|UGT1A9|ALDH9A1|AGK|GM2A|HIBCH|PNP|AKR7A3|MARC2|MGST3|ECHS1|LHPP|ACADSB|PHGDH|HOGA1|ACADM|RBP4|CPT2|SCP2|SLC25A5|SARDH|CMPK1|AKR1A1|ASS1|PRPS1|RAB14|HMGCL|HSPG2|ALDOB|ALDH4A1|AUH|AGMAT|PSAT1|ALDH1B1|CUBN|APRT|ACOT9|BPHL|NQO2|AKR1C3|GLDC|ASAH1|FECH|SACM1L|BDH1|DPEP1|BPGM|PC|DAK|AK4|QPRT|MGST1|AMPD3|GATM|MPST|GGT5|GGT1|ACY1|PTGR1|TST|ALDH7A1|IDH1|TKT|GK|ACSF2|DDC|CKMT2|GPI|CKMT1A|GPX1|FBP1|AOC1|ABHD14B|ACSL1|EPHX2|MMAB|RAN|PAH|ACSS3|ALDH6A1|PTGR2|ACSM2A|GALK1|HADH|EPHX1|FMO1|GALE|HAO2|GLYAT	1.06E-66	HSA-1430728	6.420412
5592	347	GO Function	catalytic activity	9.2E-65	RALA|PSMA4|GNA11|HSD17B10|CA12|NDUFB4|NANS|MYH9|ACO2|GNPNAT1|ATP6V1D|DHRS7|MTHFD1|PCK2|PYGB|AHCY|APMAP|TXNL1|IDH3G|F9|CRYM|CTSH|ECH1|CDC37|OGDH|ABHD11|PPIF|PDHX|ACOT13|DNPH1|AGXT2|LTF|EHHADH|FAHD2A|ACADL|ATP6V1B1|ENO1|AKR7A2|CTSD|GDA|HDHD3|PFN2|PLBD1|ACADS|PEPD|GOT2|CAPNS1|PMPCB|NDUFA2|NDUFA10|ATP6V1E1|NAPSA|HRSP12|DMGDH|BHMT2|ARL8B|GSTM3|NARS|TINAG|GGH|SQRDL|KHK|RAB21|LYZ|HEXB|ALDH2|RAB11A|RAB2A|PRDX1|DHTKD1|BBOX1|MECR|RAB17|ATP6V0A1|NNT|F13A1|ACAD11|RAB7A|ENPEP|PRDX5|ALDH8A1|CPT1A|REXO2|NDUFA9|SORD|SLC27A2|AFG3L2|BCKDHA|ECSIT|TINAGL1|PSMA5|ARL8A|NAT8|ABHD10|ATP6V1A|FTH1|BHMT|ATP6V1B2|ERLIN2|DPYS|NDUFB9|GLUD1|ENDOD1|HNMT|DLAT|QDPR|GPD1L|HGD|DDAH1|UCHL1|ACAA2|CA2|ATP6V0D2|HPD|CBR1|PSMB4|FTCD|AGL|CAPN2|RNPEP|APEH|CMBL|CRYL1|HPRT1|NDUFB8|CKB|TPP1|IDH3A|ANPEP|GLYATL1|GPD1|PRODH2|ECI1|GUSB|DCXR|HINT1|UGT2B7|GNB2|GAA|ADH1B|ASL|PDHB|CYCS|ATP6V0A4|GPD2|ACO1|DPYSL2|UQCRH|RAB1B|CYP4A11|RAB6A|AOC3|GRHPR|MSRA|AHCYL2|HSD11B2|ACSS1|CYC1|GLS|PIPOX|CES2|SHMT1|UGDH|PCK1|CHDH|PKM|UGT2B17|TALDO1|RHOT2|NDUFV1|BCAT2|CNDP2|DHRS4|MDH2|ACSM2B|ATP6V0C|NDUFA12|C11orf54|IDH2|UQCR10|RAB11B|ACAA1|SHMT2|DLST|GSTA1|TRIM2|PKLR|CYB5A|CS|PPA2|DHRS2|PTGES2|CTSB|PFKM|ALDH3A2|SND1|UGT1A9|ALDH9A1|WARS|AGK|GM2A|UBE2D3|PBLD|VCP|ATP6V1H|HIBCH|MYH10|DPP4|LONP1|PNP|OPA1|KIF21A|AKR7A3|IBA57|MARC2|CYB5R1|ATP1B1|MGST3|NDUFS2|ECHS1|LHPP|ACADSB|PHGDH|GSTO1|MYO6|ABCD3|GOT1|HOGA1|ACADM|XPNPEP2|CPT2|SCP2|ECHDC2|SARDH|CMPK1|AKR1A1|ASS1|PRPS1|RAB14|ATP6V1G1|OGDHL|HMGCL|ALDOB|PSMB9|PSMB8|ALDH4A1|VARS|AUH|AGMAT|PSAT1|ALDH1B1|SLC3A2|MAOB|APRT|ACOT9|TXNDC5|PDHA1|BPHL|NQO2|AKR1C3|IDH3B|GLDC|ASAH1|FECH|GPX3|SACM1L|BDH1|DPEP1|BPGM|NME2|CNP|PC|NIT2|PRKCD|DAK|PSAP|ALDOA|AK4|QPRT|MGST1|MYH11|AMPD3|ABAT|GATM|ILK|MPST|HAGH|GGT5|ATP5A1|SDR39U1|GGT1|TXNRD2|ACY1|IFI30|PDIA6|PTGR1|TST|ALDH7A1|SCRN1|IDH1|TKT|NDUFS1|CRYZ|RHOA|GK|ACSF2|DDC|CKMT2|GPI|CKMT1A|GPX1|FBP1|LARS2|AOC1|CFB|MME|GFM1|PPIA|ABHD14B|GSTA2|ACSL1|NDUFA13|ETFDH|KIF13B|EPHX2|CAPN1|ECHDC1|ME3|ATP1A1|MMAB|RAN|MGAM|PAH|ACSS3|ALDH6A1|PTGR2|ACSM2A|GALK1|HADH|DDX6|EPHX1|PSMC3|FMO1|PLCG2|GALE|HAO2|GLYAT	8.1E-68	GO.0003824	6.40366845

932	149	GO Process	oxidation-reduction process	4.1E-64	HSD17B10|NDUFB4|ACO2|DHRS7|MTHFD1|PYGB|TXNL1|IDH3G|CRYM|ECH1|OGDH|EHHADH|ACADL|ENO1|AKR7A2|APOA1|ACADS|PMPCB|NDUFA2|NDUFA10|DMGDH|SQRDL|ALDH2|PRDX1|DHTKD1|BBOX1|MECR|NNT|ACAD11|PRDX5|ALDH8A1|CPT1A|NDUFA9|SORD|SLC27A2|BCKDHA|ECSIT|FTH1|NDUFB9|GLUD1|DLAT|QDPR|GPD1L|HGD|ACAA2|HPD|CBR1|AGL|CRYL1|NDUFB8|IDH3A|GPD1|PRODH2|ECI1|DCXR|GAA|ADH1B|PDHB|CYCS|GPD2|ACO1|UQCRH|CYP4A11|AOC3|GRHPR|MSRA|HSD11B2|ACSS1|CYC1|PIPOX|UGDH|CHDH|PKM|TALDO1|NDUFV1|DHRS4|MDH2|NDUFA12|IDH2|UQCR10|ACAA1|DLST|GSTA1|PKLR|CYB5A|CS|DHRS2|PTGES2|PFKM|ALDH3A2|ALDH9A1|AKR7A3|MARC2|CYB5R1|MGST3|NDUFS2|ECHS1|ACADSB|PHGDH|GSTO1|ABCD3|ACADM|CPT2|SCP2|ECHDC2|SARDH|AKR1A1|OGDHL|ALDOB|ALDH4A1|AUH|ALDH1B1|MAOB|PDHA1|NQO2|AKR1C3|IDH3B|GLDC|GPX3|BDH1|BPGM|ALDOA|MGST1|SDR39U1|TXNRD2|IFI30|PDIA6|PTGR1|ALDH7A1|SLC25A12|IDH1|TKT|NDUFS1|HIGD1A|CRYZ|GPI|GPX1|AOC1|NDUFA13|ETFDH|ECHDC1|ME3|SLC25A10|PAH|ALDH6A1|PTGR2|HADH|FMO1|HAO2	4.04E-67	GO.0055114	6.33882767
46	11	GO Component	mitochondrial respiratory chain complex I	2.1E-06	NDUFB4|NDUFA2|NDUFA10|NDUFA9|NDUFB9|NDUFB8|NDUFV1|NDUFA12|NDUFS2|NDUFS1|NDUFA13	2.3E-07	GO.0005747	0.56716204
622	115	GO Process	drug metabolic process	1.1E-53	DCN|NDUFB4|ACO2|MTHFD1|AHCY|IDH3G|CTSH|OGDH|AGXT2|ATP6V1B1|ENO1|AKR7A2|PMPCB|NDUFA2|NDUFA10|HRSP12|BHMT2|SQRDL|ALDH2|PRDX1|DHTKD1|ATP6V0A1|NNT|PRDX5|ALDH8A1|NDUFA9|ATP6V1A|BHMT|ATP6V1B2|DPYS|NDUFB9|DLAT|QDPR|HGD|HPD|CBR1|FTCD|HPRT1|AMN|NDUFB8|CKB|IDH3A|ADH1B|PDHB|CYCS|ATP6V0A4|ACO1|UQCRH|GBAS|AHCYL2|ACSS1|CYC1|PIPOX|SHMT1|PKM|NDUFV1|MDH2|NDUFA12|IDH2|UQCR10|ACAA1|SHMT2|DLST|PKLR|CS|PFKM|ALDH3A2|VCP|ATP1B1|NDUFS2|PHGDH|SCP2|PRPS1|OGDHL|HMGCL|ALDOB|PSAT1|ALDH1B1|CUBN|MAOB|APRT|PDHA1|AKR1C3|IDH3B|GLDC|GPX3|BDH1|DPEP1|BPGM|PC|ALDOA|AK4|QPRT|AMPD3|GATM|MPST|GGT5|ATP5A1|GGT1|IDH1|NDUFS1|DDC|CKMT2|GPI|CKMT1A|GPX1|ACSL1|EPHX2|ME3|MMAB|PAH|ACSS3|GALK1|FMO1|GLYAT	1.25E-56	GO.0017144	5.29788107
716	113	GO Function	oxidoreductase activity	1.2E-46	HSD17B10|NDUFB4|DHRS7|MTHFD1|TXNL1|IDH3G|CRYM|OGDH|EHHADH|ACADL|AKR7A2|ACADS|NDUFA2|NDUFA10|DMGDH|SQRDL|ALDH2|PRDX1|DHTKD1|BBOX1|MECR|NNT|ACAD11|PRDX5|ALDH8A1|NDUFA9|SORD|BCKDHA|ECSIT|FTH1|NDUFB9|GLUD1|QDPR|GPD1L|HGD|HPD|CBR1|CRYL1|NDUFB8|IDH3A|GPD1|PRODH2|DCXR|ADH1B|PDHB|CYCS|GPD2|UQCRH|CYP4A11|AOC3|GRHPR|MSRA|HSD11B2|CYC1|PIPOX|UGDH|CHDH|NDUFV1|DHRS4|MDH2|NDUFA12|IDH2|UQCR10|ACAA1|GSTA1|CYB5A|DHRS2|PTGES2|ALDH3A2|ALDH9A1|AKR7A3|MARC2|CYB5R1|MGST3|NDUFS2|ACADSB|PHGDH|GSTO1|ACADM|SARDH|AKR1A1|OGDHL|ALDH4A1|ALDH1B1|MAOB|PDHA1|NQO2|AKR1C3|IDH3B|GLDC|GPX3|BDH1|MGST1|SDR39U1|TXNRD2|IFI30|PDIA6|PTGR1|ALDH7A1|IDH1|NDUFS1|CRYZ|GPX1|AOC1|NDUFA13|ETFDH|ME3|PAH|ALDH6A1|PTGR2|HADH|FMO1|HAO2	2.08E-49	GO.0016491	4.5928118
463	94	GO Component	mitochondrial matrix	1.2E-46	HSD17B10|ACO2|PCK2|IDH3G|OGDH|PPIF|PDHX|AGXT2|ACADL|ACADS|GOT2|PMPCB|NDUFA10|GRSF1|HRSP12|DMGDH|ALDH2|DNAJA3|DHTKD1|MECR|VDAC1|PRDX5|REXO2|NDUFA9|BCKDHA|ABHD10|GLUD1|DLAT|ACAA2|HSPA9|NDUFB8|IDH3A|ECI1|PDHB|TSFM|ACSS1|GLS|BCAT2|MDH2|ACSM2B|IDH2|SHMT2|DLST|CS|PPA2|DHRS2|HIBCH|LONP1|MRPL37|MRPL24|MRPL21|IBA57|NDUFS2|ECHS1|ACADSB|HOGA1|ACADM|SLC25A5|SARDH|OGDHL|HMGCL|ALDH4A1|AUH|ALDH1B1|ACOT9|PDHA1|IDH3B|GLDC|FECH|BDH1|PC|AK4|ABAT|MPST|HAGH|ATP5A1|TXNRD2|TST|ALDH7A1|NDUFS1|ACSF2|GPX1|LARS2|MRPS22|GFM1|SSBP1|ETFDH|ME3|MMAB|ACSS3|ALDH6A1|ACSM2A|HADH|GLYAT	5.62E-49	GO.0005759	4.59136402

388	88	GO Process	small molecule catabolic process	2.6E-46	HSD17B10|AHCY|CRYM|ECH1|AGXT2|EHHADH|ACADL|ENO1|ACADS|GOT2|HRSP12|KHK|HEXB|ALDH2|MECR|ACAD11|CPT1A|SORD|SLC27A2|BCKDHA|ABHD10|DPYS|GLUD1|HNMT|QDPR|HGD|DDAH1|ACAA2|HPD|FTCD|CRYL1|HPRT1|PRODH2|ECI1|GUSB|DCXR|GPD2|CYP4A11|GLS|PIPOX|SHMT1|PKM|BCAT2|ACAA1|DLST|PKLR|PFKM|ALDH3A2|HIBCH|PNP|ECHS1|ACADSB|ABCD3|GOT1|HOGA1|ACADM|CPT2|SCP2|ECHDC2|SARDH|AKR1A1|HMGCL|ALDOB|ALDH4A1|AUH|ALDH1B1|AKR1C3|GLDC|BDH1|BPGM|DAK|ALDOA|QPRT|ABAT|MPST|HAGH|TST|ALDH7A1|GK|GPI|ETFDH|ECHDC1|PAH|ALDH6A1|GALK1|HADH|GALE|HAO2	3.63E-49	GO.0044282	4.55800443
1646	160	GO Process	cellular catabolic process	8.7E-43	PSMA4|DCN|HSD17B10|PYGB|AHCY|PSMD7|CRYM|CTSH|ECH1|OGDH|FIS1|DNPH1|AGXT2|EHHADH|ACADL|ENO1|CTSD|APOA1|GDA|ACADS|GOT2|RPS16|NAPSA|HRSP12|DMGDH|GSTM3|LYZ|HEXB|PSMD11|ALDH2|PRDX1|DHTKD1|MECR|PSMD3|ACAD11|RAB7A|AMBP|ENPEP|VDAC1|PRDX5|CPT1A|SORD|SLC27A2|BCKDHA|PSMA5|ABHD10|BHMT|ERLIN2|DPYS|GLUD1|HNMT|QDPR|HGD|DDAH1|UCHL1|ACAA2|HPD|PSMB4|FTCD|AGL|CAPN2|RNPEP|FABP1|CRYL1|HPRT1|TPP1|ANPEP|PRODH2|ECI1|GUSB|RPS9|DCXR|HINT1|GAA|GPD2|RAB1B|CYP4A11|HNRNPD|TOLLIP|GLS|PIPOX|SHMT1|CHDH|PKM|BCAT2|AP2A2|ACAA1|DLST|PKLR|CTSB|PFKM|ALDH3A2|SND1|GM2A|UBE2D3|VCP|HIBCH|LONP1|PNP|ECHS1|ACADSB|GSTO1|ABCD3|RPL5|GOT1|HOGA1|ACADM|CPT2|SCP2|ECHDC2|SARDH|AKR1A1|OGDHL|HMGCL|ALDOB|PSMB9|PSMB8|ALDH4A1|AUH|ALDH1B1|MAOB|AKR1C3|GLDC|GPX3|BDH1|DPEP1|BPGM|CNP|ALDOA|QPRT|AMPD3|ABAT|MPST|HAGH|GGT5|GGT1|TST|ALDH7A1|UBA52|CRYZ|GK|GPI|GPX1|LAMP2|ACSL1|ETFDH|EPHX2|CAPN1|ECHDC1|RPL18|MGAM|PAH|ALDH6A1|GALK1|HADH|DDX6|EPHX1|PSMC3|PLCG2|HAO2	1.37E-45	GO.0044248	4.20604807
1859	169	GO Process	catabolic process	4.3E-42	PSMA4|DCN|HSD17B10|PYGB|AHCY|PSMD7|CRYM|CTSH|ECH1|OGDH|FIS1|DNPH1|AGXT2|EHHADH|ACADL|ENO1|CTSD|APOA1|GDA|PLBD1|ACADS|PEPD|GOT2|RPS16|NAPSA|HRSP12|DMGDH|GSTM3|KHK|LYZ|HEXB|PSMD11|ALDH2|PRDX1|DHTKD1|MECR|PSMD3|ACAD11|RAB7A|AMBP|ENPEP|VDAC1|PRDX5|CPT1A|SORD|SLC27A2|BCKDHA|PSMA5|ABHD10|BHMT|ERLIN2|DPYS|GLUD1|HNMT|QDPR|GPD1L|HGD|DDAH1|UCHL1|ACAA2|HPD|PSMB4|FTCD|AGL|CAPN2|RNPEP|FABP1|CRYL1|HPRT1|TPP1|ANPEP|GPD1|PRODH2|ECI1|GUSB|RPS9|DCXR|HINT1|GAA|GPD2|RAB1B|CYP4A11|HNRNPD|TOLLIP|GLS|PIPOX|CES2|SHMT1|CHDH|PKM|BCAT2|AP2A2|ACAA1|DLST|PKLR|CTSB|PFKM|ALDH3A2|SND1|GM2A|UBE2D3|VCP|HIBCH|LONP1|PNP|ECHS1|ACADSB|GSTO1|ABCD3|RPL5|GOT1|HOGA1|ACADM|CPT2|SCP2|ECHDC2|SARDH|AKR1A1|OGDHL|HMGCL|HSPG2|ALDOB|PSMB9|PSMB8|ALDH4A1|AUH|ALDH1B1|MAOB|AKR1C3|GLDC|GPX3|BDH1|DPEP1|BPGM|CNP|DAK|ALDOA|QPRT|AMPD3|ABAT|MPST|HAGH|GGT5|GGT1|TST|ALDH7A1|UBA52|CRYZ|GK|GPI|GPX1|LAMP2|ACSL1|ETFDH|EPHX2|CAPN1|ECHDC1|RPL18|MGAM|PAH|ALDH6A1|GALK1|HADH|DDX6|EPHX1|PSMC3|PLCG2|GALE|HAO2	7.54E-45	GO.0009056	4.13716111

<table>
<tr><td>1609</td><td>155</td><td>GO Process</td><td>organic substance catabolic process</td><td>7.8E-41</td><td>PSMA4|DCN|HSD17B10|PYGB|AHCY|PSMD7|CRYM|CTSH|ECH1|OGDH|DNPH1|AGXT2|EHHADH|ACADL|ENO1|CTSD|APOA1|GDA|PLBD1|ACADS|GOT2|RPS16|NAPSA|HRSP12|DMGDH|KHK|LYZ|HEXB|PSMD11|ALDH2|DHTKD1|MECR|PSMD3|ACAD11|RAB7A|AMBP|ENPEP|CPT1A|SORD|SLC27A2|BCKDHA|PSMA5|ABHD10|BHMT|ERLIN2|DPYS|GLUD1|HNMT|QDPR|GPD1L|HGD|DDAH1|UCHL1|ACAA2|HPD|PSMB4|FTCD|AGL|CAPN2|RNPEP|FABP1|CRYL1|HPRT1|TPP1|ANPEP|GPD1|PRODH2|ECI1|GUSB|RPS9|DCXR|HINT1|GAA|GPD2|CYP4A11|HNRNPD|TOLLIP|GLS|PIPOX|SHMT1|CHDH|PKM|BCAT2|AP2A2|ACAA1|DLST|PKLR|CTSB|PFKM|ALDH3A2|SND1|GM2A|UBE2D3|VCP|HIBCH|LONP1|PNP|ECHS1|ACADSB|ABCD3|RPL5|GOT1|HOGA1|ACADM|CPT2|SCP2|ECHDC2|SARDH|AKR1A1|OGDHL|HMGCL|HSPG2|ALDOB|PSMB9|PSMB8|ALDH4A1|AUH|ALDH1B1|MAOB|AKR1C3|GLDC|BDH1|BPGM|CNP|DAK|ALDOA|QPRT|AMPD3|ABAT|MPST|HAGH|GGT5|GGT1|TST|ALDH7A1|UBA52|GK|GPI|GPX1|LAMP2|ETFDH|EPHX2|CAPN1|ECHDC1|RPL18|MGAM|PAH|ALDH6A1|GALK1|HADH|DDX6|PSMC3|PLCG2|GALE|HAO2</td><td>1.53E-43</td><td>GO.1901575</td><td>4.0109579</td></tr>
</table>

14286	504	GO Component	intracellular	7.4E-39	RALA|SYPL1|HEBP1|PSMA4|DCN|GNA11|HSD17B10|NDUFB4|EPDR1|NANS|VCL|NIPSNAP1|MYH9|ACO2|GNPNAT1|ATP6V1D|MTHFD1|PCK2|PYGB|AHCY|APMAP|TXNL1|MYL12A|IDH3G|F9|PSMD7|CRYM|CTSH|EHD4|ECH1|HNRNPL|PLIN3|CDC37|OGDH|FIS1|SFXN3|PPIF|PDHX|SLC25A3|ACOT13|DNPH1|AGXT2|LTF|EHHADH|REEP6|ACADL|ATP6V1B1|ENO1|AKR7A2|CTSD|APOA1|TTR|GDA|PFN2|PLBD1|TFG|ACADS|PEPD|GOT2|CAPNS1|CANX|VIL1|HSPB1|PMPCB|RPS16|NDUFA2|ACTN4|NDUFA10|ATP6V1E1|NAPSA|LGALS3|GRSF1|HRSP12|DMGDH|BHMT2|SYNJ2BP|ARL8B|GSTM3|NARS|GGH|SQRDL|KHK|RAB21|LYZ|HEXB|PSMD11|ALDH2|CDH1|RAB11A|DNAJA3|SLC4A1|COTL1|CHCHD3|SLC9A3R1|RAB2A|PRDX1|DHTKD1|BBOX1|PACSIN2|NAPA|MECR|LRP2|CAPG|GRB14|RAB17|PSMD3|ATP6V0A1|NNT|ADD1|F13A1|SCARB2|ACAD11|RAB7A|AMBP|ENPEP|VDAC1|CALB1|PRDX5|ALDH8A1|CPT1A|REXO2|NDUFA9|RBP5|SORD|SLC27A2|RHCG|IQGAP1|AFG3L2|ARHGDIA|BCKDHA|ECSIT|TINAGL1|PSMA5|ARL8A|NAT8|ABHD10|ATP6V1A|FTH1|BHMT|ATP6V1B2|ERLIN2|DPYS|NDUFB9|GLUD1|ENDOD1|LAMTOR1|HNMT|DLAT|QDPR|SLC25A4|GPD1L|HGD|DDAH1|UCHL1|ACAA2|CA2|ATP6V0D2|HPD|CBR1|PSMB4|FTCD|TBC1D24|AGL|CAPN2|RNPEP|FABP1|APEH|CMBL|SCIN|HSPA9|COL14A1|CFL2|CRYL1|HPRT1|AMN|NDUFB8|CKB|TPP1|IDH3A|ANPEP|GLYATL1|COL6A2|GPD1|PRODH2|ECI1|GUSB|SLC23A1|RPS9|TMED10|DCXR|HINT1|UGT2B7|GNB2|GAA|YWHAG|ADH1B|TMEM126A|ASL|PDHB|CYCS|ATP6V0A4|GPD2|ACO1|DPYSL2|UQCRH|RAB1B|CSRP2|CYP4A11|AQP1|RAB6A|AOC3|GBAS|HNRNPD|DAB2|GRHPR|TSFM|MSRA|TOLLIP|AHCYL2|TLN1|HSD11B2|SFXN1|ACSS1|CYC1|GLS|PIPOX|CES2|SHMT1|UGDH|PCK1|CHDH|PKM|SLC7A8|UGT2B17|TALDO1|RHOT2|NDUFV1|BCAT2|HNRNPM|CNDP2|DHRS4|MDH2|EHD3|ACSM2B|AP2A2|KRT7|COPB2|ATP6V0C|NDUFA12|TMED9|C11orf54|IDH2|UQCR10|SERPINA5|LAMP1|RAB11B|ACAA1|SHMT2|BSG|PARVA|DLST|GSTA1|TRIM2|PKLR|CYB5A|CS|LAMTOR4|EPB41L3|PPA2|DHRS2|APOD|PTGES2|VAPA|CTSB|PFKM|ALDH3A2|ANXA6|SND1|UGT1A9|ALDH9A1|WARS|AGK|COL18A1|PLS3|GM2A|UBE2D3|PBLD|VCP|ATP6V1H|HIBCH|MSN|MYH10|COL4A2|DPP4|LONP1|MRPL37|MRPL24|PNP|MRPL21|OPA1|COA3|KIF21A|KRT19|COL6A1|AKR7A3|IBA57|MARC2|CYB5R1|CSRP1|ATP1B1|MGST3|NDUFS2|TAGLN2|ECHS1|LHPP|ACADSB|PHGDH|GSTO1|FLNA|SFXN2|MYO6|ABCD3|RPL5|GOT1|HOGA1|ACADM|RBP4|CPT2|SCP2|ECHDC2|SLC25A5|SARDH|CMPK1|AKR1A1|ASS1|PRPS1|APOOL|GSN|RAB14|ATP6V1G1|OGDHL|CLIC4|HMGCL|HNRNPR|HSPG2|ALDOB|PSMB9|PSMB8|ALDH4A1|ASPN|VARS|AUH|CLIC1|COL4A1|AGMAT|PSAT1|TBC1D4|ALDH1B1|HIST1H2AC|CUBN|SLC3A2|RSU1|CKAP4|MAOB|APRT|ACOT9|TXNDC5|PDHA1|MT1M|BPHL|NQO2|NAP1L4|AKR1C3|IDH3B|GLDC|SLC25A6|OCIAD1|ASAH1|GPNMB|CD9|FECH|KRT18|SACM1L|BDH1|PRKAR1A|DPEP1|BPGM|NME2|CNP|PC|NIT2|FHL1|ACTN1|PRKCD|DAK|PSAP|ALDOA|KTN1|AK4|QPRT|CD59|UMOD|MGST1|MYH11|AMPD3|ABAT|GATM|ILK|MPST|HAGH|ATP5A1|SEPT7|TXNRD2|ACY1|IFI30|ELAVL1|PDIA6|PTGR1|TST|RMDN1|ALDH7A1|IMMT|UBA52|SLC25A12|SCRN1|IDH1|TKT|NDUFS1|GDI1|HIGD1A|ANXA11|CRYZ|RHOA|GK|ACSF2|DDC|TANGO2|TMED4|CKMT2|GPI|CKMT1A|GPX1|FBP1|LAMP2|LARS2|TOMM40|AOC1|TOM1|SERPINA1|TGFBI|VWA1|MRPS22|MME|GFM1|FAM162A|PPIA|SSBP1|ABHD14B|GSTA2|FLNB|TMEM33|ACSL1|OCIAD2|NDUFA13|ETFDH|WDR1|KIF13B|EPHX2|CAPN1|TAGLN|EC[illegible]	4.58E-41	GO.0005622	3.81284271
237	66	GO Process	carboxylic acid catabolic process	7.9E-39	HSD17B10|AHCY|CRYM|ECH1|AGXT2|EHHADH|ACADL|ACADS|GOT2|HRSP12|HEXB|MECR|ACAD11|CPT1A|SORD|SLC27A2|BCKDHA|ABHD10|GLUD1|HNMT|QDPR|HGD|DDAH1|ACAA2|HPD|FTCD|CRYL1|PRODH2|ECI1|GUSB|DCXR|CYP4A11|GLS|PIPOX|SHMT1|BCAT2|ACAA1|DLST|ALDH3A2|HIBCH|ECHS1|ACADSB|ABCD3|GOT1|HOGA1|ACADM|CPT2|SCP2|ECHDC2|SARDH|AKR1A1|HMGCL|ALDH4A1|AUH|GLDC|QPRT|ABAT|MPST|TST|ALDH7A1|ETFDH|ECHDC1|PAH|ALDH6A1|HADH|HAO2	1.71E-41	GO.0046395	3.81034738

477	86	GO Process	monocarboxylic acid metabolic process	1.4E-38	DCN|ECH1|OGDH|PDHX|AGXT2|EHHADH|ACADL|ENO1|ACADS|GOT2|DHTKD1|MECR|SCARB2|ACAD11|ALDH8A1|CPT1A|SORD|SLC27A2|ABHD10|DLAT|ACAA2|CBR1|FTCD|CRYL1|PRODH2|ECI1|DCXR|UGT2B7|PDHB|CYP4A11|GRHPR|ACSS1|CES2|PCK1|PKM|UGT2B17|ACSM2B|IDH2|ACAA1|BSG|GSTA1|PKLR|PTGES2|PFKM|ALDH3A2|UGT1A9|ECHS1|ACADSB|PHGDH|ABCD3|HOGA1|ACADM|CPT2|SCP2|ECHDC2|AKR1A1|OGDHL|ALDOB|ALDH4A1|AUH|PDHA1|AKR1C3|BPGM|PC|NIT2|ALDOA|ABAT|HAGH|GGT5|GGT1|PTGR1|IDH1|ACSF2|GPI|GPX1|ACSL1|ETFDH|EPHX2|ECHDC1|ME3|PTGR2|ACSM2A|GALK1|HADH|HAO2|GLYAT	3.46E-41	GO.0032787	3.78696662
467	82	GO Process	cofactor metabolic process	6.1E-36	MTHFD1|AHCY|OGDH|PDHX|ACOT13|ENO1|AKR7A2|GOT2|BHMT2|GSTM3|GGH|PRDX1|DHTKD1|NNT|AMBP|PRDX5|NDUFA9|NAT8|DLAT|QDPR|GPD1L|FTCD|HSPA9|AMN|GPD1|SLC23A1|DCXR|PDHB|GPD2|AHCYL2|ACSS1|PIPOX|SHMT1|PKM|TALDO1|CNDP2|MDH2|ACSM2B|IDH2|SHMT2|DLST|GSTA1|PKLR|CYB5A|PFKM|VCP|PNP|IBA57|GSTO1|GOT1|AKR1A1|OGDHL|HMGCL|ALDOB|PSAT1|CUBN|MAOB|ACOT9|PDHA1|AKR1C3|FECH|GPX3|DPEP1|BPGM|PC|ALDOA|QPRT|HAGH|GGT5|GGT1|IDH1|TKT|ACSF2|GPI|GPX1|GSTA2|ACSL1|MMAB|ACSM2A|GALK1|FMO1|GLYAT	1.69E-38	GO.0051186	3.52139588
400	76	GO Process	generation of precursor metabolites and energy	3.4E-35	NDUFB4|ACO2|PYGB|IDH3G|OGDH|ENO1|AKR7A2|PMPCB|NDUFA2|NDUFA10|DMGDH|ALDH2|DHTKD1|NNT|NDUFA9|NDUFB9|DLAT|QDPR|SLC25A4|AGL|NDUFB8|IDH3A|GPD1|GAA|ADH1B|PDHB|CYCS|GPD2|ACO1|UQCRH|GBAS|ACSS1|CYC1|UGDH|PKM|TALDO1|NDUFV1|MDH2|NDUFA12|IDH2|UQCR10|DLST|PKLR|CYB5A|CS|PTGES2|PFKM|AKR7A3|NDUFS2|PHGDH|AKR1A1|OGDHL|HMGCL|ALDOB|ALDH4A1|ALDH1B1|MAOB|PDHA1|NQO2|IDH3B|GLDC|FECH|BDH1|BPGM|ALDOA|ATP5A1|TXNRD2|SLC25A12|IDH1|TKT|NDUFS1|GPI|ETFDH|ME3|ACSS3|GALK1	1.01E-37	GO.0006091	3.44659739
662	93	GO Process	nucleobase-containing small molecule metabolic process	3.4E-34	NDUFB4|NANS|GNPNAT1|MTHFD1|AHCY|OGDH|PDHX|ACOT13|DNPH1|ATP6V1B1|ENO1|GDA|PMPCB|NDUFA2|NDUFA10|DHTKD1|ATP6V0A1|NNT|PRDX5|REXO2|NDUFA9|ATP6V1A|ATP6V1B2|DPYS|NDUFB9|DLAT|GPD1L|HPRT1|NDUFB8|GPD1|DCXR|HINT1|PDHB|CYCS|ATP6V0A4|GPD2|UQCRH|AQP1|GBAS|AHCYL2|ACSS1|CYC1|PIPOX|SHMT1|UGDH|PKM|TALDO1|NDUFV1|MDH2|ACSM2B|NDUFA12|IDH2|UQCR10|DLST|PKLR|PFKM|VCP|PNP|OPA1|ATP1B1|NDUFS2|LHPP|CMPK1|PRPS1|OGDHL|HMGCL|ALDOB|APRT|ACOT9|PDHA1|BPGM|NME2|CNP|ALDOA|AK4|QPRT|AMPD3|ATP5A1|IDH1|TKT|NDUFS1|RHOA|ACSF2|GPI|GPX1|ABHD14B|ACSL1|RAN|ALDH6A1|ACSM2A|GALK1|FMO1|GLYAT	1.08E-36	GO.0055086	3.34672456
308	67	GO Process	cellular amino acid metabolic process	6.5E-34	HSD17B10|MTHFD1|AHCY|CRYM|AGXT2|PEPD|GOT2|HRSP12|BHMT2|NARS|BCKDHA|BHMT|DPYS|GLUD1|HNMT|QDPR|HGD|DDAH1|HPD|FTCD|CKB|GLYATL1|PRODH2|ASL|MSRA|AHCYL2|GLS|PIPOX|SHMT1|SLC7A8|BCAT2|SHMT2|DLST|PPA2|WARS|HIBCH|ACADSB|PHGDH|GOT1|HOGA1|SARDH|ASS1|HMGCL|ALDH4A1|VARS|AUH|AGMAT|PSAT1|BPHL|GLDC|DPEP1|NIT2|ABAT|GATM|MPST|GGT5|GGT1|ACY1|TST|ALDH7A1|DDC|CKMT2|CKMT1A|LARS2|PAH|ALDH6A1|GLYAT	2.19E-36	GO.0006520	3.31857524
581	86	GO Process	nucleoside phosphate metabolic process	6.1E-33	NDUFB4|MTHFD1|OGDH|PDHX|ACOT13|DNPH1|ATP6V1B1|ENO1|GDA|PMPCB|NDUFA2|NDUFA10|DHTKD1|ATP6V0A1|NNT|PRDX5|REXO2|NDUFA9|ATP6V1A|ATP6V1B2|NDUFB9|DLAT|GPD1L|HPRT1|NDUFB8|GPD1|DCXR|HINT1|PDHB|CYCS|ATP6V0A4|GPD2|UQCRH|AQP1|GBAS|ACSS1|CYC1|PIPOX|SHMT1|PKM|TALDO1|NDUFV1|MDH2|ACSM2B|NDUFA12|IDH2|UQCR10|DLST|PKLR|PFKM|VCP|PNP|OPA1|ATP1B1|NDUFS2|LHPP|CMPK1|PRPS1|OGDHL|HMGCL|ALDOB|APRT|ACOT9|PDHA1|BPGM|NME2|CNP|ALDOA|AK4|QPRT|AMPD3|ATP5A1|IDH1|TKT|NDUFS1|RHOA|ACSF2|GPI|GPX1|ABHD14B|ACSL1|RAN|ACSM2A|GALK1|FMO1|GLYAT	2.15E-35	GO.0006753	3.22175274

569	85	GO Process	small molecule biosynthetic process	8.2E-33	DCN|MTHFD1|PCK2|AHCY|OGDH|DNPH1|AGXT2|ENO1|APOA1|GDA|GOT2|BHMT2|DHTKD1|MECR|ALDH8A1|NDUFA9|SORD|SLC27A2|BHMT|GLUD1|QDPR|ACAA2|CBR1|HPRT1|GPD1|HINT1|ASL|GPD2|ACSS1|GLS|SHMT1|UGDH|PCK1|PKM|TALDO1|BCAT2|MDH2|ACSM2B|SHMT2|PKLR|PTGES2|PFKM|VCP|PNP|MGST3|PHGDH|ABCD3|GOT1|HOGA1|RBP4|SCP2|CMPK1|AKR1A1|ASS1|PRPS1|OGDHL|HMGCL|ALDOB|PSAT1|APRT|AKR1C3|BDH1|BPGM|NME2|CNP|PC|ALDOA|AMPD3|ABAT|GATM|GGT5|GGT1|SLC25A12|TKT|GPI|GPX1|FBP1|EPHX2|MMAB|SLC25A10|PAH|ACSS3|ACSM2A|GALK1|PLCG2	3.07E-35	GO.0044283	3.20867161
576	85	GO Process	nucleotide metabolic process	1.8E-32	NDUFB4|MTHFD1|OGDH|PDHX|ACOT13|DNPH1|ATP6V1B1|ENO1|GDA|PMPCB|NDUFA2|NDUFA10|DHTKD1|ATP6V0A1|NNT|PRDX5|REXO2|NDUFA9|ATP6V1A|ATP6V1B2|NDUFB9|DLAT|GPD1L|HPRT1|NDUFB8|GPD1|DCXR|HINT1|PDHB|CYCS|ATP6V0A4|GPD2|UQCRH|AQP1|GBAS|ACSS1|CYC1|PIPOX|SHMT1|PKM|TALDO1|NDUFV1|MDH2|ACSM2B|NDUFA12|IDH2|UQCR10|DLST|PKLR|PFKM|VCP|PNP|OPA1|ATP1B1|NDUFS2|CMPK1|PRPS1|OGDHL|HMGCL|ALDOB|APRT|ACOT9|PDHA1|BPGM|NME2|CNP|ALDOA|AK4|QPRT|AMPD3|ATP5A1|IDH1|TKT|NDUFS1|RHOA|ACSF2|GPI|GPX1|ABHD14B|ACSL1|RAN|ACSM2A|GALK1|FMO1|GLYAT	6.89E-35	GO.0009117	3.1756962
71	6	KEGG Pathways	Adherens junction	0.0499	VCL|ACTN4|CDH1|IQGAP1|ACTN1|RHOA	0.0158	hsa04520	0.13018995
481	75	GO Function	cofactor binding	1.4E-29	HEBP1|ACO2|PYGB|AHCY|IDH3G|CRYM|OGDH|AGXT2|ACADL|ACADS|GOT2|GSTM3|SQRDL|ALDH2|DHTKD1|NNT|ACAD11|AMBP|NDUFA9|SORD|GLUD1|QDPR|GPD1L|CRYL1|IDH3A|GPD1|PRODH2|CYCS|ACO1|CYP4A11|AOC3|GRHPR|HSD11B2|CYC1|SHMT1|UGDH|CHDH|NDUFV1|IDH2|SHMT2|CYB5A|PTGES2|MARC2|NDUFS2|ACADSB|PHGDH|GOT1|ACADM|SCP2|OGDHL|HMGCL|ALDH1B1|CUBN|MAOB|NQO2|IDH3B|GLDC|FECH|PC|MGST1|ABAT|TXNRD2|IDH1|TKT|NDUFS1|CRYZ|DDC|AOC1|ETFDH|ME3|MMAB|ALDH6A1|HADH|FMO1|HAO2	3.75E-32	GO.0048037	2.88477117

9569	390	GO Process	metabolic process	1.8E-28	PSMA4|DCN|HSD17B10|NDUFB4|NANS|MYH9|ACO2|GNPNAT1|DHRS7|MTHFD1|PCK2|PYGB|AHCY|APMAP|TXNL1|IDH3G|F9|PSMD7|CRYM|CTSH|ECH1|HNRNPL|CDC37|OGDH|FIS1|PPIF|PDHX|ACOT13|DNPH1|AGXT2|LTF|EHHADH|FAHD2A|ACADL|ATP6V1B1|ENO1|AKR7A2|CTSD|APOA1|TTR|GDA|HDHD3|PLBD1|ACADS|PEPD|GOT2|CAPNS1|PMPCB|RPS16|NDUFA2|NDUFA10|NAPSA|LGALS3|GRSF1|HRSP12|APCS|DMGDH|BHMT2|GSTM3|NARS|TINAG|GGH|SQRDL|KHK|LYZ|HEXB|PSMD11|ALDH2|RAB11A|DNAJA3|CHCHD3|RAB2A|PRDX1|DHTKD1|BBOX1|MECR|LRP2|PSMD3|ATP6V0A1|NNT|ADD1|F13A1|SCARB2|ACAD11|RAB7A|AMBP|ENPEP|VDAC1|CALB1|PRDX5|ALDH8A1|CPT1A|REXO2|NDUFA9|SORD|SLC27A2|AFG3L2|BCKDHA|ECSIT|TINAGL1|PSMA5|NAT8|ABHD10|ATP6V1A|FTH1|BHMT|ATP6V1B2|ERLIN2|DPYS|NDUFB9|GLUD1|ENDOD1|HNMT|DLAT|QDPR|SLC25A4|GPD1L|HGD|DDAH1|UCHL1|ACAA2|HPD|CBR1|PSMB4|FTCD|AGL|CAPN2|RNPEP|FABP1|APEH|CMBL|HSPA9|CRYL1|HPRT1|AMN|NDUFB8|CKB|TPP1|IDH3A|ANPEP|GLYATL1|GPD1|PRODH2|ECI1|GUSB|SLC23A1|RPS9|DCXR|HINT1|UGT2B7|GAA|ADH1B|ASL|PDHB|CYCS|ATP6V0A4|GPD2|ACO1|DPYSL2|UQCRH|RAB1B|CYP4A11|AQP1|RAB6A|AOC3|GBAS|HNRNPD|GRHPR|TSFM|MSRA|TOLLIP|AHCYL2|HSD11B2|ACSS1|CYC1|GLS|PIPOX|CES2|SHMT1|UGDH|PCK1|CHDH|PKM|SLC7A8|UGT2B17|TALDO1|NDUFV1|BCAT2|HNRNPM|CNDP2|DHRS4|MDH2|EHD3|ACSM2B|AP2A2|SLC5A2|NDUFA12|IDH2|UQCR10|RAB11B|ACAA1|SHMT2|BSG|DLST|GSTA1|TRIM2|PKLR|CYB5A|CS|PPA2|DHRS2|APOD|PTGES2|VAPA|CTSB|PFKM|ALDH3A2|SND1|UGT1A9|ALDH9A1|WARS|AGK|GM2A|UBE2D3|PBLD|VCP|HIBCH|COL4A2|DPP4|LONP1|MRPL37|MRPL24|PNP|MRPL21|OPA1|AKR7A3|IBA57|MARC2|CYB5R1|ATP1B1|MGST3|NDUFS2|ECHS1|LHPP|ACADSB|PHGDH|GSTO1|FLNA|ABCD3|RPL5|GOT1|HOGA1|ACADM|XPNPEP2|RBP4|CPT2|SCP2|ECHDC2|SARDH|CMPK1|AKR1A1|ASS1|PRPS1|GSN|RAB14|OGDHL|HMGCL|HNRNPR|HSPG2|ALDOB|PSMB9|PSMB8|ALDH4A1|VARS|AUH|AGMAT|PSAT1|ALDH1B1|CUBN|SLC3A2|CKAP4|MAOB|APRT|ACOT9|PDHA1|BPHL|NQO2|AKR1C3|IDH3B|GLDC|ASAH1|CD9|FECH|GPX3|SACM1L|BDH1|DPEP1|BPGM|NME2|CNP|PC|NIT2|PRKCD|DAK|PSAP|ALDOA|KTN1|AK4|QPRT|MGST1|AMPD3|ABAT|GATM|ILK|MPST|HAGH|GGT5|ATP5A1|SDR39U1|GGT1|TXNRD2|ACY1|IFI30|ELAVL1|PDIA6|PTGR1|TST|ALDH7A1|UBA52|SLC25A12|SCRN1|IDH1|TKT|NDUFS1|HIGD1A|CRYZ|RHOA|GK|ACSF2|DDC|CKMT2|GPI|CKMT1A|GPX1|FBP1|LAMP2|LARS2|AOC1|SERPINA1|TGFBI|CFB|VWA1|MRPS22|MME|GFM1|PPIA|SSBP1|ABHD14B|GSTA2|ACSL1|NDUFA13|ETFDH|EPHX2|CAPN1|ECHDC1|ME3|PHB2|ATP1A1|MMAB|RAN|SLC25A10|RPL18|MGAM|PAH|ACSS3|ALDH6A1|NPC2|PTGR2|ACSM2A|GALK1|HADH|DDX6|PHB|EPHX1|PSMC3|FMO1|PLCG2|GALE|HAO2|GLYAT	7.42E-31	GO.0008152	2.7747147
478	71	GO Process	purine-containing compound metabolic process	6.5E-27	NDUFB4|MTHFD1|AHCY|OGDH|PDHX|ACOT13|DNPH1|ATP6V1B1|ENO1|GDA|PMPCB|NDUFA2|NDUFA10|DHTKD1|ATP6V0A1|NDUFA9|ATP6V1A|ATP6V1B2|NDUFB9|DLAT|HPRT1|NDUFB8|HINT1|PDHB|CYCS|ATP6V0A4|UQCRH|AQP1|GBAS|AHCYL2|ACSS1|CYC1|PIPOX|SHMT1|PKM|NDUFV1|ACSM2B|NDUFA12|UQCR10|DLST|PKLR|PFKM|VCP|PNP|OPA1|ATP1B1|NDUFS2|PRPS1|OGDHL|HMGCL|ALDOB|APRT|ACOT9|PDHA1|BPGM|NME2|ALDOA|AK4|AMPD3|ATP5A1|NDUFS1|RHOA|ACSF2|GPI|GPX1|ABHD14B|ACSL1|RAN|ACSM2A|GALK1|GLYAT	2.81E-29	GO.0072521	2.6188425
274	56	GO Function	coenzyme binding	7.9E-27	PYGB|AHCY|IDH3G|CRYM|OGDH|AGXT2|ACADL|ACADS|GOT2|ALDH2|DHTKD1|NNT|ACAD11|NDUFA9|SORD|GLUD1|QDPR|GPD1L|CRYL1|IDH3A|GPD1|PRODH2|GRHPR|HSD11B2|SHMT1|UGDH|CHDH|NDUFV1|IDH2|SHMT2|MARC2|NDUFS2|ACADSB|PHGDH|GOT1|ACADM|SCP2|OGDHL|HMGCL|ALDH1B1|MAOB|NQO2|IDH3B|GLDC|PC|ABAT|TXNRD2|IDH1|CRYZ|DDC|ETFDH|ME3|ALDH6A1|HADH|FMO1|HAO2	2.8E-29	GO.0050662	2.61001795

691	84	GO Process	regulated exocytosis	1.1E-26	VCL|ATP6V1D|PYGB|PSMD7|CTSH|LTF|CTSD|APOA1|TTR|ACTN4|LGALS3|GGH|LYZ|HEXB|PSMD11|COTL1|PSMD3|ATP6V0A1|F13A1|RAB7A|SLC27A2|IQGAP1|PSMA5|ARL8A|FTH1|ENDOD1|LAMTOR1|AGL|APEH|SCIN|ANPEP|GUSB|TMED10|GAA|RAB6A|TOLLIP|TLN1|PKM|AP2A2|ATP6V0C|LAMP1|RAB11B|ACAA1|PTGES2|VAPA|CTSB|GM2A|VCP|PNP|CYB5R1|TAGLN2|FLNA|APOOL|GSN|RAB14|CKAP4|APRT|TXNDC5|ASAH1|CD9|NME2|NIT2|ACTN1|PRKCD|PSAP|ALDOA|CD59|MGST1|AMPD3|IDH1|RHOA|GPI|LAMP2|AOC1|TOM1|SERPINA1|MME|PPIA|WDR1|CAPN1|MGAM|CD63|NPC2|PSMC3	4.77E-29	GO.0045055	2.59788107
209	50	GO Process	alpha-amino acid metabolic process	1.3E-26	MTHFD1|AHCY|CRYM|AGXT2|GOT2|HRSP12|BHMT2|BHMT|GLUD1|HNMT|QDPR|HGD|DDAH1|HPD|FTCD|CKB|GLYATL1|PRODH2|ASL|MSRA|AHCYL2|GLS|PIPOX|SHMT1|BCAT2|SHMT2|DLST|HIBCH|PHGDH|GOT1|HOGA1|SARDH|ASS1|HMGCL|ALDH4A1|AUH|AGMAT|PSAT1|GLDC|DPEP1|NIT2|GATM|MPST|GGT1|ALDH7A1|CKMT2|CKMT1A|PAH|ALDH6A1|GLYAT	6.18E-29	GO.1901605	2.58860566
442	68	GO Process	purine nucleotide metabolic process	1.5E-26	NDUFB4|MTHFD1|OGDH|PDHX|ACOT13|DNPH1|ATP6V1B1|ENO1|GDA|PMPCB|NDUFA2|NDUFA10|DHTKD1|ATP6V0A1|NDUFA9|ATP6V1A|ATP6V1B2|NDUFB9|DLAT|HPRT1|NDUFB8|HINT1|PDHB|CYCS|ATP6V0A4|UQCRH|AQP1|GBAS|ACSS1|CYC1|PIPOX|PKM|NDUFV1|ACSM2B|NDUFA12|UQCR10|DLST|PKLR|PFKM|VCP|PNP|OPA1|ATP1B1|NDUFS2|PRPS1|OGDHL|HMGCL|ALDOB|APRT|ACOT9|PDHA1|BPGM|NME2|ALDOA|AK4|AMPD3|ATP5A1|NDUFS1|RHOA|ACSF2|GPI|GPX1|ABHD14B|ACSL1|RAN|ACSM2A|GALK1|GLYAT	7.43E-29	GO.0006163	2.58210231
1011	101	GO Process	organophosphate metabolic process	2.3E-26	NDUFB4|MTHFD1|OGDH|PDHX|ACOT13|DNPH1|ATP6V1B1|ENO1|APOA1|GDA|PLBD1|PMPCB|NDUFA2|NDUFA10|KHK|HEXB|DHTKD1|ATP6V0A1|NNT|PRDX5|REXO2|NDUFA9|SORD|ATP6V1A|ATP6V1B2|NDUFB9|DLAT|GPD1L|CRYL1|HPRT1|NDUFB8|GPD1|DCXR|HINT1|PDHB|CYCS|ATP6V0A4|GPD2|UQCRH|AQP1|GBAS|ACSS1|CYC1|PIPOX|SHMT1|PKM|TALDO1|NDUFV1|MDH2|ACSM2B|NDUFA12|IDH2|UQCR10|DLST|PKLR|PFKM|AGK|VCP|PNP|OPA1|ATP1B1|NDUFS2|LHPP|SCP2|CMPK1|AKR1A1|PRPS1|RAB14|OGDHL|HMGCL|ALDOB|APRT|ACOT9|PDHA1|SACM1L|BPGM|NME2|CNP|DAK|ALDOA|AK4|QPRT|AMPD3|ATP5A1|IDH1|TKT|NDUFS1|RHOA|GK|ACSF2|GPI|GPX1|FBP1|ABHD14B|ACSL1|RAN|ACSM2A|GALK1|FMO1|PLCG2|GLYAT	1.17E-28	GO.0019637	2.56401645
297	57	GO Process	coenzyme metabolic process	2.8E-26	MTHFD1|AHCY|OGDH|PDHX|ACOT13|ENO1|BHMT2|GGH|DHTKD1|NNT|PRDX5|NDUFA9|DLAT|QDPR|GPD1L|FTCD|GPD1|SLC23A1|DCXR|PDHB|GPD2|AHCYL2|ACSS1|PIPOX|SHMT1|PKM|TALDO1|MDH2|ACSM2B|IDH2|SHMT2|DLST|PKLR|CYB5A|PFKM|VCP|PNP|GSTO1|AKR1A1|OGDHL|HMGCL|ALDOB|ACOT9|PDHA1|BPGM|PC|ALDOA|QPRT|IDH1|TKT|ACSF2|GPI|ACSL1|ACSM2A|GALK1|FMO1|GLYAT	1.47E-28	GO.0006732	2.55575202
774	88	GO Process	exocytosis	2.9E-26	RALA|VCL|ATP6V1D|PYGB|PSMD7|CTSH|LTF|CTSD|APOA1|TTR|ACTN4|LGALS3|GGH|LYZ|HEXB|PSMD11|RAB11A|COTL1|PSMD3|ATP6V0A1|F13A1|RAB7A|SLC27A2|IQGAP1|PSMA5|ARL8A|FTH1|ENDOD1|LAMTOR1|AGL|APEH|SCIN|ANPEP|GUSB|TMED10|GAA|RAB6A|TOLLIP|TLN1|PKM|AP2A2|ATP6V0C|LAMP1|RAB11B|ACAA1|PTGES2|VAPA|CTSB|GM2A|VCP|MYH10|PNP|CYB5R1|TAGLN2|FLNA|APOOL|GSN|RAB14|CKAP4|APRT|TXNDC5|ASAH1|CD9|NME2|NIT2|ACTN1|PRKCD|PSAP|ALDOA|CD59|MGST1|AMPD3|SCRN1|IDH1|RHOA|GPI|LAMP2|AOC1|TOM1|SERPINA1|MME|PPIA|WDR1|CAPN1|MGAM|CD63|NPC2|PSMC3	1.58E-28	GO.0006887	2.5543634

5281	264	GO Process	organonitrogen compound metabolic process	3.3E-26	PSMA4|DCN|HSD17B10|NDUFB4|MYH9|MTHFD1|AHCY|F9|PSMD7|CRYM|CTSH|CDC37|OGDH|PPIF|PDHX|ACOT13|DNPH1|AGXT2|LTF|EHHADH|ACADL|ATP6V1B1|ENO1|CTSD|APOA1|TTR|GDA|PLBD1|PEPD|GOT2|CAPNS1|PMPCB|RPS16|NDUFA2|NDUFA10|NAPSA|HRSP12|APCS|DMGDH|BHMT2|GSTM3|NARS|TINAG|GGH|LYZ|HEXB|PSMD11|RAB11A|RAB2A|DHTKD1|BBOX1|PSMD3|ATP6V0A1|NNT|ADD1|F13A1|RAB7A|AMBP|ENPEP|CALB1|PRDX5|CPT1A|NDUFA9|AFG3L2|BCKDHA|TINAGL1|PSMA5|NAT8|ATP6V1A|BHMT|ATP6V1B2|ERLIN2|DPYS|NDUFB9|GLUD1|HNMT|DLAT|QDPR|GPD1L|HGD|DDAH1|UCHL1|HPD|PSMB4|FTCD|CAPN2|RNPEP|APEH|HPRT1|AMN|NDUFB8|CKB|TPP1|ANPEP|GLYATL1|GPD1|PRODH2|GUSB|RPS9|DCXR|HINT1|ASL|PDHB|CYCS|ATP6V0A4|GPD2|UQCRH|RAB1B|AQP1|RAB6A|AOC3|GBAS|TSFM|MSRA|TOLLIP|AHCYL2|ACSS1|CYC1|GLS|PIPOX|SHMT1|UGDH|PCK1|CHDH|PKM|SLC7A8|TALDO1|NDUFV1|BCAT2|CNDP2|MDH2|ACSM2B|AP2A2|NDUFA12|IDH2|UQCR10|RAB11B|SHMT2|DLST|GSTA1|TRIM2|PKLR|PPA2|VAPA|CTSB|PFKM|ALDH3A2|ALDH9A1|WARS|AGK|GM2A|UBE2D3|VCP|HIBCH|DPP4|LONP1|MRPL37|MRPL24|PNP|MRPL21|OPA1|IBA57|ATP1B1|MGST3|NDUFS2|LHPP|ACADSB|PHGDH|GSTO1|RPL5|GOT1|HOGA1|ACADM|XPNPEP2|SARDH|CMPK1|AKR1A1|ASS1|PRPS1|GSN|OGDHL|HMGCL|HSPG2|ALDOB|PSMB9|PSMB8|ALDH4A1|VARS|AUH|AGMAT|PSAT1|CUBN|CKAP4|MAOB|APRT|ACOT9|PDHA1|BPHL|GLDC|ASAH1|FECH|DPEP1|BPGM|NME2|PC|NIT2|PRKCD|PSAP|ALDOA|KTN1|AK4|QPRT|MGST1|AMPD3|ABAT|GATM|ILK|MPST|HAGH|GGT5|ATP5A1|GGT1|ACY1|PDIA6|TST|ALDH7A1|UBA52|SCRN1|IDH1|TKT|NDUFS1|RHOA|ACSF2|DDC|CKMT2|GPI|CKMT1A|GPX1|LAMP2|LARS2|AOC1|SERPINA1|TGFBI|CFB|VWA1|MRPS22|MME|GFM1|PPIA|ABHD14B|GSTA2|ACSL1|CAPN1|MMAB|RAN|RPL18|PAH|ALDH6A1|ACSM2A|GALK1|PHB|PSMC3|FMO1|GLYAT	1.86E-28	GO.1901564	2.54881166

16271	517	GO Component	cell	1.1E-25	RALA|SYPL1|HEBP1|PSMA4|DCN|GNA11|HSD17B10|CA12|NDUFB4|EPDR1|NANS|VCL|NIPSNAP1|MYH9|ACO2|GNPNAT1|ATP6V1D|MTHFD1|PCK2|PYGB|AHCY|APMAP|TXNL1|MYL12A|IDH3G|F9|PSMD7|CRYM|CTSH|EHD4|ECH1|HNRNPL|PLIN3|CDC37|OGDH|FIS1|SFXN3|PPIF|PDHX|SLC25A3|ACOT13|DNPH1|AGXT2|LTF|EHHADH|REEP6|ACADL|ATP6V1B1|ENO1|AKR7A2|CTSD|APOA1|TTR|GDA|PFN2|PLBD1|TFG|ACADS|PEPD|GOT2|CAPNS1|CANX|VIL1|HSPB1|PMPCB|RPS16|NDUFA2|ACTN4|NDUFA10|ATP6V1E1|NAPSA|LGALS3|GRSF1|HRSP12|DMGDH|BHMT2|SYNJ2BP|ARL8B|GSTM3|NARS|GGH|SQRDL|KHK|RAB21|LYZ|HEXB|PSMD11|ALDH2|CDH1|RAB11A|DNAJA3|SLC4A1|COTL1|CHCHD3|SLC9A3R1|RAB2A|PRDX1|DHTKD1|BBOX1|PACSIN2|NAPA|MECR|EPCAM|LRP2|CAPG|GRB14|RAB17|PSMD3|ATP6V0A1|NNT|ADD1|F13A1|SCARB2|ACAD11|RAB7A|AMBP|ENPEP|VDAC1|CALB1|PRDX5|ALDH8A1|CPT1A|REXO2|NDUFA9|RBP5|SORD|SLC27A2|RHCG|IQGAP1|AFG3L2|ARHGDIA|BCKDHA|BCAM|ECSIT|TINAGL1|PSMA5|ARL8A|NAT8|ABHD10|ATP6V1A|FTH1|BHMT|ATP6V1B2|ERLIN2|DPYS|NDUFB9|GLUD1|ENDOD1|LAMTOR1|HNMT|DLAT|QDPR|SLC25A4|GPD1L|HGD|DDAH1|UCHL1|ACAA2|CA2|ATP6V0D2|HPD|CBR1|PSMB4|FTCD|TBC1D24|AGL|CAPN2|RNPEP|FABP1|APEH|CMBL|SCIN|HSPA9|COL14A1|CFL2|CRYL1|HPRT1|AMN|NDUFB8|CKB|TPP1|IDH3A|CDH16|ANPEP|GLYATL1|COL6A2|GPD1|PRODH2|ECI1|GUSB|SLC23A1|RPS9|TMED10|DCXR|HINT1|UGT2B7|GNB2|GAA|YWHAG|ADH1B|TMEM126A|ASL|PDHB|CYCS|ATP6V0A4|GPD2|ACO1|DPYSL2|UQCRH|RAB1B|CSRP2|CYP4A11|AQP1|RAB6A|AOC3|GBAS|HNRNPD|DAB2|GRHPR|TSFM|MSRA|TOLLIP|AHCYL2|TLN1|HSD11B2|SFXN1|ACSS1|CYC1|GLS|PIPOX|CES2|SHMT1|UGDH|PCK1|CHDH|PKM|SLC7A8|UGT2B17|TALDO1|RHOT2|NDUFV1|BCAT2|HNRNPM|CNDP2|DHRS4|MDH2|EHD3|ACSM2B|AP2A2|SLC5A2|KRT7|COPB2|ATP6V0C|NDUFA12|TMED9|C11orf54|IDH2|UQCR10|SERPINA5|LAMP1|RAB11B|ACAA1|SHMT2|BSG|PARVA|DLST|GSTA1|TRIM2|PKLR|CYB5A|CS|LAMTOR4|EPB41L3|PPA2|DHRS2|APOD|PTGES2|VAPA|CTSB|PFKM|ALDH3A2|ANXA6|SND1|UGT1A9|ALDH9A1|WARS|AGK|COL18A1|PLS3|GM2A|UBE2D3|PBLD|VCP|ATP6V1H|HIBCH|MSN|MYH10|COL4A2|DPP4|LONP1|MRPL37|MRPL24|PNP|MRPL21|OPA1|COA3|KIF21A|KRT19|COL6A1|AKR7A3|IBA57|MARC2|CYB5R1|CSRP1|ATP1B1|MGST3|NDUFS2|TAGLN2|ECHS1|LHPP|ACADSB|PHGDH|GSTO1|FLNA|SFXN2|MYO6|ABCD3|RPL5|GOT1|HOGA1|ACADM|XPNPEP2|RBP4|CPT2|SCP2|ECHDC2|SLC25A5|SARDH|CMPK1|AKR1A1|ASS1|PRPS1|APOOL|GSN|RAB14|ATP6V1G1|OGDHL|CLIC4|HMGCL|HNRNPR|HSPG2|ALDOB|PSMB9|PSMB8|ALDH4A1|ASPN|VARS|AUH|CLIC1|COL4A1|AGMAT|PSAT1|KCTD12|TBC1D4|ALDH1B1|HIST1H2AC|CUBN|SLC3A2|RSU1|CKAP4|MAOB|APRT|ACOT9|TXNDC5|PDHA1|MT1M|BPHL|NQO2|NAP1L4|AKR1C3|IDH3B|GLDC|SLC25A6|OCIAD1|ASAH1|GPNMB|CD9|FECH|KRT18|SACM1L|BDH1|PRKAR1A|DPEP1|BPGM|NME2|CNP|PC|NIT2|FHL1|ACTN1|PRKCD|DAK|PSAP|ALDOA|KTN1|AK4|QPRT|CD59|UMOD|MGST1|MYH11|AMPD3|ABAT|GATM|ILK|MPST|HAGH|GGT5|ATP5A1|SEPT7|GGT1|TXNRD2|ACY1|IFI30|ELAVL1|PDIA6|PTGR1|TST|RMDN1|ALDH7A1|IMMT|UBA52|SLC25A12|SCRN1|IDH1|TKT|NDUFS1|SLC4A4|GDI1|PDZK1|HIGD1A|ANXA11|CRYZ|RHOA|GK|ACSF2|DDC|TANGO2|TMED4|CKMT2|GPI|CKMT1A|GPX1|FBP1|LAMP2|LARS2|TOMM40|AOC1|TOM1|SERPINA1|TGFBI|CFB|VWA1|MRPS22|MME|GFM1|FAM162A|PPIA|SSBP1|ABHD14B|GSTA2|F[illegible]	8.08E-28	GO.0005623	2.49788107
498	70	GO Process	neutrophil mediated immunity	2.4E-25	VCL|ATP6V1D|PYGB|PSMD7|CTSH|LTF|CTSD|TTR|LGALS3|GGH|LYZ|HEXB|PSMD11|COTL1|PSMD3|ATP6V0A1|RAB7A|SLC27A2|IQGAP1|PSMA5|ARL8A|FTH1|LAMTOR1|AGL|APEH|ANPEP|GUSB|GAA|RAB6A|TOLLIP|PKM|AP2A2|ATP6V0C|LAMP1|ACAA1|PTGES2|VAPA|CTSB|GM2A|VCP|PNP|GSN|RAB14|CKAP4|APRT|TXNDC5|ASAH1|NME2|NIT2|PRKCD|PSAP|ALDOA|CD59|MGST1|AMPD3|IDH1|RHOA|GPI|LAMP2|AOC1|TOM1|SERPINA1|MME|PPIA|WDR1|CAPN1|MGAM|CD63|NPC2|PSMC3	1.42E-27	GO.0002446	2.46197888

<table>
<tr><td>485</td><td>69</td><td>GO Process</td><td>neutrophil degranulation</td><td>3E-25</td><td>VCL|ATP6V1D|PYGB|PSMD7|CTSH|LTF|CTSD|TTR|LGALS3|GGH|LYZ|HEXB|PSMD11|COTL1|PSMD3|ATP6V0A1|RAB7A|SLC27A2|IQGAP1|PSMA5|ARL8A|FTH1|LAMTOR1|AGL|APEH|ANPEP|GUSB|GAA|RAB6A|TOLLIP|PKM|AP2A2|ATP6V0C|LAMP1|ACAA1|PTGES2|VAPA|CTSB|GM2A|VCP|PNP|GSN|RAB14|CKAP4|APRT|TXNDC5|ASAH1|NME2|NIT2|PRKCD|PSAP|ALDOA|CD59|MGST1|AMPD3|IDH1|RHOA|GPI|LAMP2|AOC1|TOM1|SERPINA1|MME|PPIA|CAPN1|MGAM|CD63|NPC2|PSMC3</td><td>1.84E-27</td><td>GO.0043312</td><td>2.45214335</td></tr>
<tr><td>8797</td><td>362</td><td>GO Process</td><td>cellular metabolic process</td><td>4.8E-25</td><td>PSMA4|DCN|HSD17B10|NDUFB4|NANS|ACO2|GNPNAT1|MTHFD1|PYGB|AHCY|IDH3G|PSMD7|CRYM|CTSH|ECH1|HNRNPL|CDC37|OGDH|FIS1|PPIF|PDHX|ACOT13|DNPH1|AGXT2|LTF|EHHADH|ACADL|ATP6V1B1|ENO1|AKR7A2|CTSD|APOA1|TTR|GDA|PLBD1|ACADS|PEPD|GOT2|PMPCB|RPS16|NDUFA2|NDUFA10|NAPSA|LGALS3|GRSF1|HRSP12|APCS|DMGDH|BHMT2|GSTM3|NARS|GGH|SQRDL|KHK|LYZ|HEXB|PSMD11|ALDH2|RAB11A|DNAJA3|CHCHD3|RAB2A|PRDX1|DHTKD1|BBOX1|MECR|LRP2|PSMD3|ATP6V0A1|NNT|ADD1|F13A1|SCARB2|ACAD11|RAB7A|AMBP|ENPEP|VDAC1|CALB1|PRDX5|ALDH8A1|CPT1A|REXO2|NDUFA9|SORD|SLC27A2|AFG3L2|BCKDHA|PSMA5|NAT8|ABHD10|ATP6V1A|BHMT|ATP6V1B2|ERLIN2|DPYS|NDUFB9|GLUD1|ENDOD1|HNMT|DLAT|QDPR|SLC25A4|GPD1L|HGD|DDAH1|UCHL1|ACAA2|HPD|CBR1|PSMB4|FTCD|AGL|CAPN2|RNPEP|FABP1|APEH|CMBL|HSPA9|CRYL1|HPRT1|AMN|NDUFB8|CKB|TPP1|IDH3A|ANPEP|GLYATL1|GPD1|PRODH2|ECI1|GUSB|SLC23A1|RPS9|DCXR|HINT1|UGT2B7|GAA|ADH1B|ASL|PDHB|CYCS|ATP6V0A4|GPD2|ACO1|DPYSL2|UQCRH|RAB1B|CYP4A11|AQP1|RAB6A|AOC3|GBAS|HNRNPD|GRHPR|TSFM|MSRA|TOLLIP|AHCYL2|ACSS1|CYC1|GLS|PIPOX|CES2|SHMT1|UGDH|PCK1|CHDH|PKM|SLC7A8|UGT2B17|TALDO1|NDUFV1|BCAT2|HNRNPM|CNDP2|DHRS4|MDH2|EHD3|ACSM2B|AP2A2|NDUFA12|IDH2|UQCR10|RAB11B|ACAA1|SHMT2|BSG|DLST|GSTA1|TRIM2|PKLR|CYB5A|CS|PPA2|DHRS2|PTGES2|VAPA|CTSB|PFKM|ALDH3A2|SND1|UGT1A9|ALDH9A1|WARS|AGK|GM2A|UBE2D3|VCP|HIBCH|COL4A2|LONP1|MRPL37|MRPL24|PNP|MRPL21|OPA1|AKR7A3|IBA57|MARC2|ATP1B1|MGST3|NDUFS2|ECHS1|LHPP|ACADSB|PHGDH|GSTO1|FLNA|ABCD3|RPL5|GOT1|HOGA1|ACADM|RBP4|CPT2|SCP2|ECHDC2|SARDH|CMPK1|AKR1A1|ASS1|PRPS1|GSN|RAB14|OGDHL|HMGCL|HNRNPR|HSPG2|ALDOB|PSMB9|PSMB8|ALDH4A1|VARS|AUH|AGMAT|PSAT1|ALDH1B1|CUBN|CKAP4|MAOB|APRT|ACOT9|PDHA1|BPHL|NQO2|AKR1C3|IDH3B|GLDC|ASAH1|CD9|FECH|GPX3|SACM1L|BDH1|DPEP1|BPGM|NME2|CNP|PC|NIT2|PRKCD|DAK|PSAP|ALDOA|KTN1|AK4|QPRT|MGST1|AMPD3|ABAT|GATM|ILK|MPST|HAGH|GGT5|ATP5A1|GGT1|TXNRD2|ACY1|ELAVL1|PDIA6|PTGR1|TST|ALDH7A1|UBA52|SLC25A12|IDH1|TKT|NDUFS1|CRYZ|RHOA|GK|ACSF2|DDC|CKMT2|GPI|CKMT1A|GPX1|FBP1|LAMP2|LARS2|AOC1|SERPINA1|TGFBI|VWA1|MRPS22|MME|GFM1|PPIA|SSBP1|ABHD14B|GSTA2|ACSL1|NDUFA13|ETFDH|EPHX2|CAPN1|ECHDC1|ME3|PHB2|ATP1A1|MMAB|RAN|SLC25A10|RPL18|MGAM|PAH|ACSS3|ALDH6A1|PTGR2|ACSM2A|GALK1|HADH|DDX6|PHB|EPHX1|PSMC3|FMO1|PLCG2|HAO2|GLYAT</td><td>3.13E-27</td><td>GO.0044237</td><td>2.43178549</td></tr>
<tr><td>679</td><td>80</td><td>GO Component</td><td>mitochondrial membrane</td><td>8.3E-25</td><td>NDUFB4|OGDH|FIS1|SFXN3|PPIF|SLC25A3|ACADL|GOT2|PMPCB|NDUFA2|NDUFA10|LGALS3|SYNJ2BP|SQRDL|CHCHD3|NNT|ACAD11|VDAC1|CPT1A|NDUFA9|SORD|AFG3L2|ECSIT|NDUFB9|SLC25A4|NDUFB8|PRODH2|TMEM126A|CYCS|GPD2|UQCRH|GBAS|SFXN1|CYC1|CHDH|RHOT2|NDUFV1|NDUFA12|UQCR10|SHMT2|CYB5A|AGK|MRPL37|MRPL24|MRPL21|OPA1|COA3|MARC2|NDUFS2|SFXN2|ACADM|CPT2|SLC25A5|ASS1|APOOL|MAOB|BPHL|SLC25A6|FECH|BDH1|CNP|MGST1|GATM|ATP5A1|IMMT|UBA52|SLC25A12|NDUFS1|HIGD1A|GK|CKMT2|CKMT1A|TOMM40|MRPS22|ACSL1|NDUFA13|ETFDH|PHB2|SLC25A10|PHB</td><td>7.69E-27</td><td>GO.0031966</td><td>2.40788339</td></tr>
</table>

2460	162	GO Function	small molecule binding	1.3E-24	RALA|GNA11|MYH9|GNPNAT1|MTHFD1|PCK2|PYGB|AHCY|IDH3G|CRYM|EHD4|OGDH|AGXT2|ACADL|ATP6V1B1|APOA1|ACADS|GOT2|ACTN4|HRSP12|ARL8B|NARS|TINAG|KHK|RAB21|ALDH2|RAB11A|DNAJA3|RAB2A|DHTKD1|RAB17|NNT|ACAD11|RAB7A|CALB1|RBP5|SORD|SLC27A2|AFG3L2|ARL8A|ATP6V1A|ATP6V1B2|ERLIN2|DPYS|GLUD1|QDPR|GPD1L|DDAH1|FTCD|FABP1|HSPA9|CRYL1|HPRT1|CKB|IDH3A|GPD1|PRODH2|HINT1|RAB1B|RAB6A|GRHPR|HSD11B2|ACSS1|SHMT1|UGDH|PCK1|CHDH|PKM|TALDO1|RHOT2|NDUFV1|EHD3|ACSM2B|IDH2|SERPINA5|RAB11B|SHMT2|BSG|PKLR|APOD|PFKM|ANXA6|UGT1A9|WARS|AGK|UBE2D3|VCP|MYH10|LONP1|PNP|OPA1|KIF21A|MARC2|NDUFS2|ACADSB|PHGDH|MYO6|ABCD3|GOT1|ACADM|RBP4|SCP2|CMPK1|ASS1|PRPS1|RAB14|OGDHL|HMGCL|ALDOB|VARS|ALDH1B1|CUBN|MAOB|APRT|NQO2|IDH3B|GLDC|PRKAR1A|NME2|CNP|PC|PRKCD|DAK|PSAP|ALDOA|AK4|MYH11|ABAT|ILK|ATP5A1|SEPT7|TXNRD2|IDH1|CRYZ|RHOA|GK|ACSF2|DDC|CKMT2|CKMT1A|FBP1|LARS2|GFM1|ACSL1|NDUFA13|ETFDH|KIF13B|ME3|ATP1A1|MMAB|RAN|TWF1|ACSS3|ALDH6A1|NPC2|ACSM2A|GALK1|HADH|DDX6|PSMC3|FMO1|HAO2	5.91E-27	GO.0036094	2.38728952
455	66	GO Process	ribose phosphate metabolic process	1.5E-24	NDUFB4|OGDH|PDHX|ACOT13|ATP6V1B1|ENO1|PMPCB|NDUFA2|NDUFA10|DHTKD1|ATP6V0A1|NDUFA9|ATP6V1A|ATP6V1B2|NDUFB9|DLAT|HPRT1|NDUFB8|HINT1|PDHB|CYCS|ATP6V0A4|UQCRH|AQP1|GBAS|ACSS1|CYC1|PIPOX|PKM|TALDO1|NDUFV1|ACSM2B|NDUFA12|UQCR10|DLST|PKLR|PFKM|VCP|OPA1|ATP1B1|NDUFS2|CMPK1|PRPS1|OGDHL|HMGCL|ALDOB|APRT|ACOT9|PDHA1|BPGM|NME2|ALDOA|AK4|AMPD3|ATP5A1|TKT|NDUFS1|RHOA|ACSF2|GPI|ABHD14B|ACSL1|RAN|ACSM2A|GALK1|GLYAT	1.04E-26	GO.0019693	2.38239087
722	81	GO Component	mitochondrial envelope	6.3E-24	NDUFB4|OGDH|FIS1|SFXN3|PPIF|SLC25A3|ACADL|GOT2|PMPCB|NDUFA2|NDUFA10|LGALS3|SYNJ2BP|SQRDL|CHCHD3|NNT|ACAD11|VDAC1|CPT1A|REXO2|NDUFA9|SORD|AFG3L2|ECSIT|NDUFB9|SLC25A4|NDUFB8|PRODH2|TMEM126A|CYCS|GPD2|UQCRH|GBAS|SFXN1|CYC1|CHDH|RHOT2|NDUFV1|NDUFA12|UQCR10|SHMT2|CYB5A|AGK|MRPL37|MRPL24|MRPL21|OPA1|COA3|MARC2|NDUFS2|SFXN2|ACADM|CPT2|SLC25A5|ASS1|APOOL|MAOB|BPHL|SLC25A6|FECH|BDH1|CNP|MGST1|GATM|ATP5A1|IMMT|UBA52|SLC25A12|NDUFS1|HIGD1A|GK|CKMT2|CKMT1A|TOMM40|MRPS22|ACSL1|NDUFA13|ETFDH|PHB2|SLC25A10|PHB	6.83E-26	GO.0005740	2.31979107
440	64	GO Process	ribonucleotide metabolic process	7.3E-24	NDUFB4|OGDH|PDHX|ACOT13|ATP6V1B1|ENO1|PMPCB|NDUFA2|NDUFA10|DHTKD1|ATP6V0A1|NDUFA9|ATP6V1A|ATP6V1B2|NDUFB9|DLAT|HPRT1|NDUFB8|HINT1|PDHB|CYCS|ATP6V0A4|UQCRH|AQP1|GBAS|ACSS1|CYC1|PIPOX|PKM|NDUFV1|ACSM2B|NDUFA12|UQCR10|DLST|PKLR|PFKM|VCP|OPA1|ATP1B1|NDUFS2|CMPK1|PRPS1|OGDHL|HMGCL|ALDOB|APRT|ACOT9|PDHA1|BPGM|NME2|ALDOA|AK4|AMPD3|ATP5A1|NDUFS1|RHOA|ACSF2|GPI|ABHD14B|ACSL1|RAN|ACSM2A|GALK1|GLYAT	5.65E-26	GO.0009259	2.31354889
425	63	GO Process	purine ribonucleotide metabolic process	7.3E-24	NDUFB4|OGDH|PDHX|ACOT13|ATP6V1B1|ENO1|PMPCB|NDUFA2|NDUFA10|DHTKD1|ATP6V0A1|NDUFA9|ATP6V1A|ATP6V1B2|NDUFB9|DLAT|HPRT1|NDUFB8|HINT1|PDHB|CYCS|ATP6V0A4|UQCRH|AQP1|GBAS|ACSS1|CYC1|PIPOX|PKM|NDUFV1|ACSM2B|NDUFA12|UQCR10|DLST|PKLR|PFKM|VCP|OPA1|ATP1B1|NDUFS2|PRPS1|OGDHL|HMGCL|ALDOB|APRT|ACOT9|PDHA1|BPGM|NME2|ALDOA|AK4|AMPD3|ATP5A1|NDUFS1|RHOA|ACSF2|GPI|ABHD14B|ACSL1|RAN|ACSM2A|GALK1|GLYAT	5.63E-26	GO.0009150	2.31354889
457	65	GO Process	carbohydrate metabolic process	8.4E-24	NANS|PCK2|PYGB|IDH3G|OGDH|ENO1|AKR7A2|GOT2|KHK|HEXB|ALDH2|DHTKD1|CPT1A|SORD|ABHD10|DLAT|GPD1L|AGL|CRYL1|IDH3A|GPD1|GUSB|SLC23A1|DCXR|UGT2B7|GAA|PDHB|GPD2|PCK1|PKM|UGT2B17|TALDO1|MDH2|SLC5A2|IDH2|PKLR|CYB5A|CS|APOD|PFKM|UGT1A9|GM2A|GSTO1|GOT1|RBP4|AKR1A1|OGDHL|ALDOB|ALDH1B1|SLC3A2|PDHA1|BPGM|PC|DAK|ALDOA|SLC25A12|IDH1|TKT|GK|GPI|FBP1|SLC25A10|MGAM|GALK1|GALE	6.93E-26	GO.0005975	2.30777937

<table>
<tr><td>12432</td><td>445</td><td>GO Component</td><td>organelle</td><td>1.1E-23</td><td>RALA|SYPL1|PSMA4|DCN|GNA11|HSD17B10|NDUFB4|EPDR1|VCL|NIPSNAP1|MYH9|ACO2|GNPNAT1|ATP6V1D|MTHFD1|PCK2|PYGB|AHCY|APMAP|TXNL1|MYL12A|IDH3G|F9|PSMD7|CRYM|CTSH|EHD4|ECH1|HNRNPL|PLIN3|OGDH|FIS1|SFXN3|PPIF|PDHX|SLC25A3|ACOT13|DNPH1|AGXT2|LTF|EHHADH|REEP6|ACADL|ATP6V1B1|ENO1|AKR7A2|CTSD|APOA1|TTR|PFN2|PLBD1|TFG|ACADS|PEPD|GOT2|CANX|VIL1|HSPB1|PMPCB|RPS16|NDUFA2|ACTN4|NDUFA10|ATP6V1E1|NAPSA|LGALS3|GRSF1|HRSP12|DMGDH|SYNJ2BP|ARL8B|GSTM3|GGH|SQRDL|RAB21|LYZ|HEXB|PSMD11|ALDH2|CDH1|RAB11A|DNAJA3|SLC4A1|COTL1|CHCHD3|SLC9A3R1|RAB2A|PRDX1|DHTKD1|PACSIN2|NAPA|MECR|LRP2|CAPG|GRB14|RAB17|PSMD3|ATP6V0A1|NNT|ADD1|F13A1|SCARB2|ACAD11|RAB7A|AMBP|ENPEP|VDAC1|CALB1|PRDX5|CPT1A|REXO2|NDUFA9|SORD|SLC27A2|RHCG|IQGAP1|AFG3L2|ARHGDIA|BCKDHA|ECSIT|PSMA5|ARL8A|NAT8|ABHD10|FTH1|BHMT|ATP6V1B2|ERLIN2|NDUFB9|GLUD1|LAMTOR1|DLAT|SLC25A4|UCHL1|ACAA2|ATP6V0D2|HPD|PSMB4|FTCD|AGL|CAPN2|RNPEP|FABP1|APEH|SCIN|HSPA9|COL14A1|CFL2|AMN|NDUFB8|CKB|TPP1|IDH3A|ANPEP|GLYATL1|COL6A2|PRODH2|ECI1|GUSB|SLC23A1|RPS9|TMED10|DCXR|HINT1|UGT2B7|GAA|YWHAG|ADH1B|TMEM126A|PDHB|CYCS|ATP6V0A4|GPD2|ACO1|DPYSL2|UQCRH|RAB1B|CSRP2|CYP4A11|AQP1|RAB6A|AOC3|GBAS|HNRNPD|DAB2|GRHPR|TSFM|MSRA|TOLLIP|AHCYL2|TLN1|HSD11B2|SFXN1|ACSS1|CYC1|GLS|PIPOX|CES2|SHMT1|UGDH|CHDH|PKM|UGT2B17|TALDO1|RHOT2|NDUFV1|BCAT2|HNRNPM|CNDP2|DHRS4|MDH2|EHD3|ACSM2B|AP2A2|KRT7|COPB2|ATP6V0C|NDUFA12|TMED9|C11orf54|IDH2|UQCR10|SERPINA5|LAMP1|RAB11B|ACAA1|SHMT2|BSG|PARVA|DLST|CYB5A|CS|LAMTOR4|EPB41L3|PPA2|DHRS2|APOD|PTGES2|VAPA|CTSB|PFKM|ALDH3A2|ANXA6|SND1|UGT1A9|WARS|AGK|COL18A1|PLS3|GM2A|UBE2D3|VCP|ATP6V1H|HIBCH|MSN|MYH10|COL4A2|DPP4|LONP1|MRPL37|MRPL24|PNP|MRPL21|OPA1|COA3|KIF21A|KRT19|COL6A1|IBA57|MARC2|CYB5R1|CSRP1|MGST3|NDUFS2|ECHS1|LHPP|ACADSB|FLNA|SFXN2|MYO6|ABCD3|RPL5|GOT1|HOGA1|ACADM|XPNPEP2|CPT2|SCP2|ECHDC2|SLC25A5|SARDH|CMPK1|ASS1|APOOL|GSN|RAB14|ATP6V1G1|OGDHL|CLIC4|HMGCL|HNRNPR|HSPG2|ALDOB|PSMB9|PSMB8|ALDH4A1|AUH|CLIC1|COL4A1|AGMAT|TBC1D4|ALDH1B1|HIST1H2AC|CUBN|SLC3A2|CKAP4|MAOB|APRT|ACOT9|TXNDC5|PDHA1|MT1M|BPHL|NQO2|NAP1L4|AKR1C3|IDH3B|GLDC|SLC25A6|OCIAD1|ASAH1|GPNMB|CD9|FECH|KRT18|SACM1L|BDH1|PRKAR1A|DPEP1|NME2|CNP|PC|NIT2|FHL1|ACTN1|PRKCD|PSAP|ALDOA|KTN1|AK4|CD59|UMOD|MGST1|MYH11|AMPD3|ABAT|GATM|ILK|MPST|HAGH|ATP5A1|SEPT7|TXNRD2|ACY1|IFI30|ELAVL1|PDIA6|TST|RMDN1|ALDH7A1|IMMT|UBA52|SLC25A12|SCRN1|IDH1|TKT|NDUFS1|GDI1|HIGD1A|ANXA11|RHOA|GK|ACSF2|DDC|TANGO2|TMED4|CKMT2|GPI|CKMT1A|GPX1|FBP1|LAMP2|LARS2|TOMM40|AOC1|TOM1|SERPINA1|TGFBI|VWA1|MRPS22|MME|GFM1|FAM162A|PPIA|SSBP1|ABHD14B|FLNB|TMEM33|ACSL1|OCIAD2|NDUFA13|ETFDH|WDR1|KIF13B|EPHX2|CAPN1|ECHDC1|ME3|PHB2|ATP1A1|MMAB|RAN|SLC25A10|RPL18|MGAM|CD63|KRT8|TWF1|ACSS3|ALDH6A1|NPC2|NDRG2|ACSM2A|CIRBP|EML2|HADH|DDX6|PHB|EPHX1|PDLIM5|VPS29|PSMC3|FMO1|HAO2|GLYAT</td><td>1.39E-25</td><td>GO.0043226</td><td>2.29469216</td></tr>
</table>

14652	489	GO Process	cellular process	1.3E-23	RALA|SYPL1|HEBP1|PSMA4|DCN|GNA11|HSD17B10|NDUFB4|NANS|VCL|MYH9|ACO2|GNPNAT1|ATP6V1D|MTHFD1|PYGB|AHCY|TXNL1|IDH3G|PSMD7|CRYM|CTSH|EHD4|ECH1|HNRNPL|CDC37|OGDH|FIS1|PPIF|PDHX|ACOT13|DNPH1|AGXT2|LTF|EHHADH|ACADL|ATP6V1B1|ENO1|AKR7A2|CTSD|APOA1|TTR|GDA|PFN2|PLBD1|TFG|ACADS|PEPD|GOT2|CAPNS1|CANX|VIL1|HSPB1|PMPCB|RPS16|NDUFA2|ACTN4|NDUFA10|LAMA5|ATP6V1E1|NAPSA|LGALS3|GRSF1|HRSP12|APCS|DMGDH|BHMT2|SYNJ2BP|ARL8B|GSTM3|NARS|GGH|SQRDL|KHK|RAB21|LYZ|HEXB|PSMD11|ALDH2|CDH1|RAB11A|DNAJA3|SLC4A1|COTL1|CHCHD3|SLC9A3R1|RAB2A|PRDX1|DHTKD1|BBOX1|PACSIN2|NAPA|MECR|EPCAM|LRP2|CAPG|GRB14|RAB17|PSMD3|ATP6V0A1|NNT|ADD1|F13A1|SCARB2|ACAD11|RAB7A|AMBP|ENPEP|VDAC1|CALB1|PRDX5|ALDH8A1|CPT1A|REXO2|NDUFA9|SORD|SLC27A2|RHCG|IQGAP1|AFG3L2|ARHGDIA|BCKDHA|BCAM|ECSIT|PSMA5|ARL8A|NAT8|ABHD10|ATP6V1A|FTH1|BHMT|ATP6V1B2|ERLIN2|DPYS|NDUFB9|GLUD1|ENDOD1|LAMTOR1|HNMT|DLAT|QDPR|SLC25A4|GPD1L|HGD|DDAH1|UCHL1|ACAA2|CA2|ATP6V0D2|HPD|CBR1|PSMB4|FTCD|TBC1D24|AGL|CAPN2|RNPEP|FABP1|APEH|CMBL|SCIN|HSPA9|COL14A1|CFL2|CRYL1|HPRT1|AMN|NDUFB8|CKB|TPP1|IDH3A|CDH16|ANPEP|GLYATL1|COL6A2|GPD1|PRODH2|ECI1|GUSB|SLC23A1|RPS9|TMED10|DCXR|HINT1|UGT2B7|GNB2|GAA|YWHAG|ADH1B|ASL|PDHB|CYCS|ATP6V0A4|GPD2|ACO1|DPYSL2|UQCRH|RAB1B|CSRP2|CYP4A11|AQP1|RAB6A|AOC3|GBAS|HNRNPD|DAB2|GRHPR|TSFM|MSRA|TOLLIP|AHCYL2|TLN1|SFXN1|ACSS1|CYC1|GLS|PIPOX|CES2|SHMT1|UGDH|PCK1|CHDH|PKM|SLC7A8|UGT2B17|TALDO1|RHOT2|NDUFV1|BCAT2|HNRNPM|CNDP2|DHRS4|MDH2|EHD3|ACSM2B|AP2A2|KRT7|ATP6V0C|NDUFA12|TMED9|IDH2|UQCR10|SERPINA5|LAMP1|RAB11B|ACAA1|SHMT2|BSG|PARVA|DLST|GSTA1|TRIM2|PKLR|CYB5A|CS|LAMTOR4|EPB41L3|PPA2|DHRS2|APOD|PTGES2|VAPA|CTSB|PFKM|ALDH3A2|ANXA6|SND1|UGT1A9|ALDH9A1|WARS|AGK|COL18A1|PLS3|GM2A|UBE2D3|VCP|ATP6V1H|HIBCH|MSN|MYH10|COL4A2|DPP4|LONP1|MRPL37|MRPL24|PNP|MRPL21|OPA1|COA3|KIF21A|KRT19|COL6A1|AKR7A3|IBA57|MARC2|CYB5R1|ATP1B1|DPT|MGST3|NDUFS2|TAGLN2|ECHS1|LHPP|ACADSB|PHGDH|GSTO1|FLNA|MYO6|ABCD3|RPL5|GOT1|HOGA1|ACADM|RBP4|CPT2|SCP2|ECHDC2|SLC25A5|SARDH|CMPK1|AKR1A1|ASS1|PRPS1|APOOL|GSN|RAB14|ATP6V1G1|OGDHL|CLIC4|HMGCL|HNRNPR|HSPG2|ALDOB|PSMB9|PSMB8|ALDH4A1|ASPN|VARS|AUH|CLIC1|COL4A1|AGMAT|PSAT1|KCTD12|TBC1D4|ALDH1B1|HIST1H2AC|CUBN|SLC3A2|RSU1|CKAP4|MAOB|APRT|ACOT9|TXNDC5|PDHA1|MT1M|BPHL|NQO2|NAP1L4|AKR1C3|IDH3B|GLDC|SLC25A6|ASAH1|GPNMB|CD9|FECH|GPX3|KRT18|SACM1L|BDH1|PRKAR1A|DPEP1|BPGM|NME2|CNP|PC|NIT2|FHL1|ACTN1|PRKCD|DAK|PSAP|ALDOA|KTN1|AK4|QPRT|CD59|UMOD|MGST1|MYH11|AMPD3|ABAT|GATM|ILK|MPST|HAGH|GGT5|ATP5A1|SEPT7|GGT1|TXNRD2|ACY1|IFI30|ELAVL1|PDIA6|PTGR1|TST|ALDH7A1|IMMT|UBA52|SLC25A12|SCRN1|IDH1|TKT|NDUFS1|SLC4A4|GDI1|HIGD1A|ANXA11|CRYZ|RHOA|GK|ACSF2|DDC|CKMT2|GPI|CKMT1A|GPX1|FBP1|LAMP2|LARS2|TOMM40|AOC1|TOM1|SERPINA1|TGFBI|VWA1|MRPS22|MME|GFM1|FAM162A|PPIA|SSBP1|ABHD14B|GSTA2|FLNB|TMEM33|ACSL1|NDUFA13|ETFDH|WDR1|KIF13B|EPHX2|CAPN1|TAGLN|ECHDC1|ME3|PHB2|ATP1A1|MMAB|RAN|SLC25A10|RPL18|MGAM|CD63|PAH|KRT8|TWF1|ACSS3|ALDH6A1|NPC2|NDRG[illegible]	1.12E-25	GO.0009987	2.28794261

<table>
<tr><td>4958</td><td>246</td><td>GO Component</td><td>cytosol</td><td>2E-23</td><td>PSMA4|NANS|VCL|MYH9|GNPNAT1|ATP6V1D|MTHFD1|AHCY|TXNL1|MYL12A|PSMD7|CRYM|CTSH|ECH1|HNRNPL|PLIN3|CDC37|ACOT13|DNPH1|EHHADH|ATP6V1B1|ENO1|AKR7A2|APOA1|GDA|PLBD1|TFG|CAPNS1|HSPB1|RPS16|ACTN4|ATP6V1E1|HRSP12|BHMT2|GSTM3|NARS|GGH|KHK|RAB21|PSMD11|RAB11A|DNAJA3|RAB2A|PRDX1|BBOX1|PACSIN2|NAPA|GRB14|PSMD3|ATP6V0A1|ADD1|RAB7A|CALB1|PRDX5|ALDH8A1|SORD|SLC27A2|IQGAP1|ARHGDIA|ECSIT|PSMA5|ABHD10|ATP6V1A|FTH1|BHMT|ATP6V1B2|ERLIN2|DPYS|ENDOD1|HNMT|QDPR|GPD1L|HGD|DDAH1|UCHL1|CA2|HPD|CBR1|PSMB4|FTCD|AGL|CAPN2|FABP1|APEH|CMBL|CRYL1|HPRT1|CKB|GPD1|RPS9|HINT1|GNB2|YWHAG|ADH1B|ASL|CYCS|ACO1|DPYSL2|RAB1B|RAB6A|HNRNPD|DAB2|GRHPR|MSRA|TOLLIP|AHCYL2|TLN1|GLS|PIPOX|SHMT1|UGDH|PCK1|PKM|TALDO1|RHOT2|NDUFV1|CNDP2|DHRS4|EHD3|AP2A2|KRT7|COPB2|NDUFA12|IDH2|LAMP1|RAB11B|ACAA1|PARVA|GSTA1|PKLR|CYB5A|EPB41L3|APOD|PTGES2|PFKM|SND1|ALDH9A1|WARS|AGK|PLS3|UBE2D3|VCP|ATP6V1H|MSN|MYH10|LONP1|PNP|OPA1|KIF21A|KRT19|AKR7A3|TAGLN2|LHPP|PHGDH|GSTO1|FLNA|MYO6|ABCD3|RPL5|GOT1|RBP4|SCP2|CMPK1|AKR1A1|ASS1|PRPS1|GSN|RAB14|ATP6V1G1|CLIC4|HMGCL|ALDOB|PSMB9|PSMB8|VARS|PSAT1|TBC1D4|CUBN|SLC3A2|RSU1|CKAP4|APRT|NQO2|AKR1C3|KRT18|PRKAR1A|BPGM|NME2|CNP|PC|NIT2|FHL1|ACTN1|PRKCD|DAK|ALDOA|QPRT|MYH11|AMPD3|ILK|MPST|HAGH|SEPT7|TXNRD2|ACY1|IFI30|ELAVL1|PDIA6|ALDH7A1|UBA52|IDH1|TKT|GDI1|ANXA11|CRYZ|RHOA|GK|DDC|GPI|GPX1|FBP1|TOMM40|TOM1|FAM162A|ABHD14B|GSTA2|FLNB|WDR1|KIF13B|EPHX2|CAPN1|ECHDC1|RAN|RPL18|PAH|KRT8|TWF1|NDRG2|GALK1|DDX6|PDLIM5|VPS29|PSMC3|PLCG2|GALE|HAO2</td><td>2.72E-25</td><td>GO.0005829</td><td>2.27055338</td></tr>
<tr><td>157</td><td>41</td><td>GO Component</td><td>myelin sheath</td><td>2.5E-23</td><td>RALA|ACO2|SLC25A3|GOT2|CANX|NDUFA10|PRDX1|NAPA|ATP6V1A|ATP6V1B2|DLAT|SLC25A4|UCHL1|CA2|HSPA9|CKB|IDH3A|GNB2|YWHAG|PKM|MDH2|EHD3|DLST|VCP|MSN|ATP1B1|PHGDH|SLC25A5|ASS1|GSN|PDHA1|CNP|ATP5A1|IMMT|SLC25A12|TKT|NDUFS1|GDI1|WDR1|ATP1A1|PHB</td><td>3.9E-25</td><td>GO.0043209</td><td>2.25968795</td></tr>
</table>

<table>
<tr><td>9135</td><td>366</td><td>GO Process</td><td>organic substance metabolic process</td><td>3.3E-23</td><td>PSMA4|DCN|HSD17B10|NDUFB4|NANS|MYH9|ACO2|GNPNAT1|MTHFD1|PCK2|PYGB|AHCY|IDH3G|F9|PSMD7|CRYM|CTSH|ECH1|HNRNPL|CDC37|OGDH|PPIF|PDHX|ACOT13|DNPH1|AGXT2|LTF|EHHADH|ACADL|ATP6V1B1|ENO1|AKR7A2|CTSD|APOA1|TTR|GDA|PLBD1|ACADS|PEPD|GOT2|CAPNS1|PMPCB|RPS16|NDUFA2|NDUFA10|NAPSA|LGALS3|GRSF1|HRSP12|APCS|DMGDH|BHMT2|GSTM3|NARS|TINAG|GGH|KHK|LYZ|HEXB|PSMD11|ALDH2|RAB11A|DNAJA3|CHCHD3|RAB2A|DHTKD1|BBOX1|MECR|LRP2|PSMD3|ATP6V0A1|NNT|ADD1|F13A1|SCARB2|ACAD11|RAB7A|AMBP|ENPEP|CALB1|PRDX5|ALDH8A1|CPT1A|REXO2|NDUFA9|SORD|SLC27A2|AFG3L2|BCKDHA|TINAGL1|PSMA5|NAT8|ABHD10|ATP6V1A|BHMT|ATP6V1B2|ERLIN2|DPYS|NDUFB9|GLUD1|ENDOD1|HNMT|DLAT|QDPR|GPD1L|HGD|DDAH1|UCHL1|ACAA2|HPD|CBR1|PSMB4|FTCD|AGL|CAPN2|RNPEP|FABP1|APEH|CRYL1|HPRT1|AMN|NDUFB8|CKB|TPP1|IDH3A|ANPEP|GLYATL1|GPD1|PRODH2|ECI1|GUSB|SLC23A1|RPS9|DCXR|HINT1|UGT2B7|GAA|ADH1B|ASL|PDHB|CYCS|ATP6V0A4|GPD2|ACO1|DPYSL2|UQCRH|RAB1B|CYP4A11|AQP1|RAB6A|AOC3|GBAS|HNRNPD|GRHPR|TSFM|MSRA|TOLLIP|AHCYL2|HSD11B2|ACSS1|CYC1|GLS|PIPOX|CES2|SHMT1|UGDH|PCK1|CHDH|PKM|SLC7A8|UGT2B17|TALDO1|NDUFV1|BCAT2|HNRNPM|CNDP2|DHRS4|MDH2|EHD3|ACSM2B|AP2A2|SLC5A2|NDUFA12|IDH2|UQCR10|RAB11B|ACAA1|SHMT2|BSG|DLST|GSTA1|TRIM2|PKLR|CYB5A|CS|PPA2|DHRS2|APOD|PTGES2|VAPA|CTSB|PFKM|ALDH3A2|SND1|UGT1A9|ALDH9A1|WARS|AGK|GM2A|UBE2D3|VCP|HIBCH|COL4A2|DPP4|LONP1|MRPL37|MRPL24|PNP|MRPL21|OPA1|AKR7A3|IBA57|MARC2|CYB5R1|ATP1B1|MGST3|NDUFS2|ECHS1|LHPP|ACADSB|PHGDH|GSTO1|FLNA|ABCD3|RPL5|GOT1|HOGA1|ACADM|XPNPEP2|RBP4|CPT2|SCP2|ECHDC2|SARDH|CMPK1|AKR1A1|ASS1|PRPS1|GSN|RAB14|OGDHL|HMGCL|HNRNPR|HSPG2|ALDOB|PSMB9|PSMB8|ALDH4A1|VARS|AUH|AGMAT|PSAT1|ALDH1B1|CUBN|SLC3A2|CKAP4|MAOB|APRT|ACOT9|PDHA1|BPHL|AKR1C3|IDH3B|GLDC|ASAH1|CD9|FECH|SACM1L|BDH1|DPEP1|BPGM|NME2|CNP|PC|NIT2|PRKCD|DAK|PSAP|ALDOA|KTN1|AK4|QPRT|MGST1|AMPD3|ABAT|GATM|ILK|MPST|HAGH|GGT5|ATP5A1|GGT1|ACY1|ELAVL1|PDIA6|PTGR1|TST|ALDH7A1|UBA52|SLC25A12|SCRN1|IDH1|TKT|NDUFS1|RHOA|GK|ACSF2|DDC|CKMT2|GPI|CKMT1A|GPX1|FBP1|LAMP2|LARS2|AOC1|SERPINA1|TGFBI|CFB|VWA1|MRPS22|MME|GFM1|PPIA|SSBP1|ABHD14B|GSTA2|ACSL1|ETFDH|EPHX2|CAPN1|ECHDC1|ME3|PHB2|MMAB|RAN|SLC25A10|RPL18|MGAM|PAH|ACSS3|ALDH6A1|NPC2|PTGR2|ACSM2A|GALK1|HADH|DDX6|PHB|EPHX1|PSMC3|FMO1|PLCG2|GALE|HAO2|GLYAT</td><td>2.9E-25</td><td>GO.0071704</td><td>2.24762535</td></tr>
</table>

12193	438	GO Component	intracellular organelle	5.8E-23	RALA|SYPL1|PSMA4|DCN|HSD17B10|NDUFB4|EPDR1|VCL|NIPSNAP1|MYH9|ACO2|GNPNAT1|ATP6V1D|MTHFD1|PCK2|PYGB|AHCY|APMAP|TXNL1|MYL12A|IDH3G|F9|PSMD7|CRYM|CTSH|EHD4|ECH1|HNRNPL|PLIN3|OGDH|FIS1|SFXN3|PPIF|PDHX|SLC25A3|ACOT13|DNPH1|AGXT2|LTF|EHHADH|REEP6|ACADL|ATP6V1B1|ENO1|AKR7A2|CTSD|APOA1|TTR|PFN2|PLBD1|TFG|ACADS|PEPD|GOT2|CANX|VIL1|HSPB1|PMPCB|RPS16|NDUFA2|ACTN4|NDUFA10|ATP6V1E1|NAPSA|LGALS3|GRSF1|HRSP12|DMGDH|SYNJ2BP|ARL8B|GGH|SQRDL|RAB21|LYZ|HEXB|PSMD11|ALDH2|CDH1|RAB11A|DNAJA3|SLC4A1|COTL1|CHCHD3|SLC9A3R1|RAB2A|PRDX1|DHTKD1|PACSIN2|NAPA|MECR|LRP2|CAPG|GRB14|RAB17|PSMD3|ATP6V0A1|NNT|ADD1|F13A1|SCARB2|ACAD11|RAB7A|AMBP|ENPEP|VDAC1|CALB1|PRDX5|CPT1A|REXO2|NDUFA9|SORD|SLC27A2|RHCG|IQGAP1|AFG3L2|ARHGDIA|BCKDHA|ECSIT|PSMA5|ARL8A|NAT8|ABHD10|FTH1|ATP6V1B2|ERLIN2|NDUFB9|GLUD1|LAMTOR1|DLAT|SLC25A4|UCHL1|ACAA2|ATP6V0D2|HPD|PSMB4|FTCD|AGL|CAPN2|RNPEP|FABP1|APEH|SCIN|HSPA9|COL14A1|CFL2|AMN|NDUFB8|CKB|TPP1|IDH3A|ANPEP|GLYATL1|COL6A2|PRODH2|ECI1|GUSB|SLC23A1|RPS9|TMED10|DCXR|HINT1|UGT2B7|GAA|YWHAG|ADH1B|TMEM126A|PDHB|CYCS|ATP6V0A4|GPD2|ACO1|DPYSL2|UQCRH|RAB1B|CSRP2|CYP4A11|AQP1|RAB6A|AOC3|GBAS|HNRNPD|DAB2|GRHPR|TSFM|MSRA|TOLLIP|AHCYL2|TLN1|HSD11B2|SFXN1|ACSS1|CYC1|GLS|PIPOX|CES2|SHMT1|UGDH|CHDH|PKM|UGT2B17|TALDO1|RHOT2|NDUFV1|BCAT2|HNRNPM|CNDP2|DHRS4|MDH2|EHD3|ACSM2B|AP2A2|KRT7|COPB2|ATP6V0C|NDUFA12|TMED9|C11orf54|IDH2|UQCR10|SERPINA5|LAMP1|RAB11B|ACAA1|SHMT2|BSG|PARVA|DLST|CYB5A|CS|LAMTOR4|EPB41L3|PPA2|DHRS2|APOD|PTGES2|VAPA|CTSB|PFKM|ALDH3A2|ANXA6|SND1|UGT1A9|WARS|AGK|COL18A1|PLS3|GM2A|UBE2D3|VCP|ATP6V1H|HIBCH|MSN|MYH10|COL4A2|DPP4|LONP1|MRPL37|MRPL24|PNP|MRPL21|OPA1|COA3|KIF21A|KRT19|COL6A1|IBA57|MARC2|CYB5R1|CSRP1|MGST3|NDUFS2|ECHS1|LHPP|ACADSB|FLNA|SFXN2|MYO6|ABCD3|RPL5|GOT1|HOGA1|ACADM|CPT2|SCP2|ECHDC2|SLC25A5|SARDH|CMPK1|ASS1|APOOL|GSN|RAB14|ATP6V1G1|OGDHL|CLIC4|HMGCL|HNRNPR|HSPG2|ALDOB|PSMB9|PSMB8|ALDH4A1|AUH|CLIC1|COL4A1|AGMAT|ALDH1B1|HIST1H2AC|CUBN|SLC3A2|CKAP4|MAOB|APRT|ACOT9|TXNDC5|PDHA1|MT1M|BPHL|NQO2|NAP1L4|AKR1C3|IDH3B|GLDC|SLC25A6|OCIAD1|ASAH1|GPNMB|CD9|FECH|KRT18|SACM1L|BDH1|DPEP1|NME2|CNP|PC|NIT2|FHL1|ACTN1|PRKCD|PSAP|ALDOA|KTN1|AK4|CD59|UMOD|MGST1|MYH11|AMPD3|ABAT|GATM|ILK|MPST|HAGH|ATP5A1|SEPT7|TXNRD2|IFI30|ELAVL1|PDIA6|TST|RMDN1|ALDH7A1|IMMT|UBA52|SLC25A12|SCRN1|IDH1|TKT|NDUFS1|GDI1|HIGD1A|ANXA11|RHOA|GK|ACSF2|DDC|TANGO2|TMED4|CKMT2|GPI|CKMT1A|GPX1|FBP1|LAMP2|LARS2|TOMM40|AOC1|TOM1|SERPINA1|TGFBI|VWA1|MRPS22|MME|GFM1|FAM162A|PPIA|SSBP1|ABHD14B|FLNB|TMEM33|ACSL1|OCIAD2|NDUFA13|ETFDH|WDR1|KIF13B|EPHX2|CAPN1|ECHDC1|ME3|PHB2|ATP1A1|MMAB|RAN|SLC25A10|RPL18|MGAM|CD63|KRT8|TWF1|ACSS3|ALDH6A1|NPC2|NDRG2|ACSM2A|CIRBP|EML2|HADH|DDX6|PHB|EPHX1|PDLIM5|VPS29|PSMC3|FMO1|HAO2|GLYAT	9.74E-25	GO.0043229	2.22395775

<table>
<tr><td>4130</td><td>218</td><td>GO Process</td><td>transport</td><td>6.3E-23</td><td>RALA|CA12|VCL|MYH9|ATP6V1D|PYGB|F9|PSMD7|CRYM|CTSH|EHD4|ECH1|PLIN3|CDC37|FIS1|SFXN3|PPIF|SLC25A3|LTF|EHHADH|ATP6V1B1|CTSD|APOA1|TTR|TFG|GOT2|CANX|VIL1|HSPB1|PMPCB|RPS16|ACTN4|ATP6V1E1|LGALS3|SYNJ2BP|TINAG|GGH|RAB21|LYZ|HEXB|PSMD11|RAB11A|SLC4A1|COTL1|SLC9A3R1|RAB2A|PACSIN2|NAPA|LRP2|RAB17|PSMD3|ATP6V0A1|NNT|ADD1|F13A1|SCARB2|RAB7A|AMBP|VDAC1|CPT1A|NDUFA9|SLC27A2|RHCG|IQGAP1|AFG3L2|TINAGL1|PSMA5|ARL8A|ATP6V1A|FTH1|ATP6V1B2|ERLIN2|ENDOD1|LAMTOR1|SLC25A4|UCHL1|ACAA2|CA2|ATP6V0D2|AGL|FABP1|APEH|SCIN|HSPA9|AMN|ANPEP|GUSB|SLC23A1|RPS9|TMED10|GAA|YWHAG|ATP6V0A4|ACO1|DPYSL2|RAB1B|AQP1|RAB6A|DAB2|TOLLIP|TLN1|SFXN1|CYC1|GLS|PIPOX|PKM|SLC7A8|RHOT2|DHRS4|EHD3|AP2A2|SLC5A2|COPB2|ATP6V0C|TMED9|SERPINA5|LAMP1|RAB11B|ACAA1|BSG|CYB5A|APOD|PTGES2|VAPA|CTSB|ANXA6|AGK|GM2A|UBE2D3|VCP|ATP6V1H|MYH10|PNP|OPA1|CYB5R1|ATP1B1|TAGLN2|FLNA|SFXN2|MYO6|ABCD3|RPL5|RBP4|CPT2|SCP2|SLC25A5|APOOL|GSN|RAB14|ATP6V1G1|CLIC4|HMGCL|HSPG2|CLIC1|TBC1D4|CUBN|SLC3A2|CKAP4|APRT|TXNDC5|AKR1C3|SLC25A6|ASAH1|CD9|KRT18|NME2|CNP|NIT2|ACTN1|PRKCD|PSAP|ALDOA|CD59|MGST1|AMPD3|ATP5A1|PDIA6|TST|UBA52|SLC25A12|SCRN1|IDH1|SLC4A4|GDI1|PDZK1|ANXA11|RHOA|DDC|TMED4|GPI|LAMP2|TOMM40|AOC1|TOM1|SERPINA1|MME|PPIA|TMEM33|ACSL1|NDUFA13|WDR1|KIF13B|EPHX2|CAPN1|PHB2|ATP1A1|RAN|SLC25A10|RPL18|MGAM|CD63|NPC2|SLC43A2|PHB|VPS29|PSMC3|PLCG2|HAO2</td><td>5.62E-25</td><td>GO.0006810</td><td>2.21985963</td></tr>
</table>

<table>
<tr><td>11244</td><td>416</td><td>GO Component</td><td>membrane-bounded organelle</td><td>9E-23</td><td>RALA|SYPL1|PSMA4|DCN|HSD17B10|NDUFB4|EPDR1|VCL|NIPSNAP1|MYH9|ACO2|GNPNAT1|ATP6V1D|MTHFD1|PCK2|PYGB|AHCY|APMAP|TXNL1|IDH3G|F9|PSMD7|CRYM|CTSH|EHD4|ECH1|HNRNPL|PLIN3|OGDH|FIS1|SFXN3|PPIF|PDHX|SLC25A3|ACOT13|DNPH1|AGXT2|LTF|EHHADH|REEP6|ACADL|ATP6V1B1|ENO1|AKR7A2|CTSD|APOA1|TTR|PLBD1|TFG|ACADS|PEPD|GOT2|CANX|HSPB1|PMPCB|RPS16|NDUFA2|ACTN4|NDUFA10|ATP6V1E1|NAPSA|LGALS3|GRSF1|HRSP12|DMGDH|SYNJ2BP|ARL8B|GGH|SQRDL|RAB21|LYZ|HEXB|PSMD11|ALDH2|CDH1|RAB11A|DNAJA3|COTL1|CHCHD3|RAB2A|PRDX1|DHTKD1|PACSIN2|NAPA|MECR|LRP2|CAPG|GRB14|RAB17|PSMD3|ATP6V0A1|NNT|ADD1|F13A1|SCARB2|ACAD11|RAB7A|AMBP|ENPEP|VDAC1|CALB1|PRDX5|CPT1A|REXO2|NDUFA9|SORD|SLC27A2|RHCG|IQGAP1|AFG3L2|BCKDHA|ECSIT|PSMA5|ARL8A|NAT8|ABHD10|FTH1|BHMT|ATP6V1B2|ERLIN2|NDUFB9|GLUD1|LAMTOR1|DLAT|SLC25A4|UCHL1|ACAA2|ATP6V0D2|HPD|PSMB4|FTCD|AGL|CAPN2|RNPEP|FABP1|APEH|HSPA9|COL14A1|CFL2|AMN|NDUFB8|CKB|TPP1|IDH3A|ANPEP|GLYATL1|COL6A2|PRODH2|ECI1|GUSB|RPS9|TMED10|HINT1|UGT2B7|GAA|YWHAG|ADH1B|TMEM126A|PDHB|CYCS|ATP6V0A4|GPD2|ACO1|UQCRH|RAB1B|CSRP2|CYP4A11|AQP1|RAB6A|AOC3|GBAS|HNRNPD|DAB2|GRHPR|TSFM|MSRA|TOLLIP|AHCYL2|HSD11B2|SFXN1|ACSS1|CYC1|GLS|PIPOX|CES2|SHMT1|UGDH|CHDH|PKM|UGT2B17|TALDO1|RHOT2|NDUFV1|BCAT2|HNRNPM|CNDP2|DHRS4|MDH2|EHD3|ACSM2B|AP2A2|COPB2|ATP6V0C|NDUFA12|TMED9|C11orf54|IDH2|UQCR10|SERPINA5|LAMP1|RAB11B|ACAA1|SHMT2|BSG|DLST|CYB5A|CS|LAMTOR4|PPA2|DHRS2|APOD|PTGES2|VAPA|CTSB|PFKM|ALDH3A2|ANXA6|SND1|UGT1A9|WARS|AGK|COL18A1|GM2A|UBE2D3|VCP|ATP6V1H|HIBCH|COL4A2|DPP4|LONP1|MRPL37|MRPL24|PNP|MRPL21|OPA1|COA3|COL6A1|IBA57|MARC2|CYB5R1|CSRP1|MGST3|NDUFS2|ECHS1|LHPP|ACADSB|FLNA|SFXN2|MYO6|ABCD3|RPL5|GOT1|HOGA1|ACADM|XPNPEP2|CPT2|SCP2|ECHDC2|SLC25A5|SARDH|CMPK1|ASS1|APOOL|GSN|RAB14|ATP6V1G1|OGDHL|CLIC4|HMGCL|HNRNPR|HSPG2|PSMB9|PSMB8|ALDH4A1|AUH|CLIC1|COL4A1|AGMAT|TBC1D4|ALDH1B1|HIST1H2AC|CUBN|SLC3A2|CKAP4|MAOB|APRT|ACOT9|TXNDC5|PDHA1|MT1M|BPHL|NQO2|NAP1L4|AKR1C3|IDH3B|GLDC|SLC25A6|OCIAD1|ASAH1|GPNMB|CD9|FECH|KRT18|SACM1L|BDH1|DPEP1|NME2|CNP|PC|NIT2|FHL1|ACTN1|PRKCD|PSAP|ALDOA|KTN1|AK4|CD59|UMOD|MGST1|MYH11|AMPD3|ABAT|GATM|ILK|MPST|HAGH|ATP5A1|SEPT7|TXNRD2|ACY1|IFI30|ELAVL1|PDIA6|TST|ALDH7A1|IMMT|UBA52|SLC25A12|SCRN1|IDH1|TKT|NDUFS1|GDI1|HIGD1A|ANXA11|RHOA|GK|ACSF2|DDC|TANGO2|TMED4|CKMT2|GPI|CKMT1A|GPX1|FBP1|LAMP2|LARS2|TOMM40|AOC1|TOM1|SERPINA1|TGFBI|VWA1|MRPS22|MME|GFM1|FAM162A|PPIA|SSBP1|ABHD14B|FLNB|TMEM33|ACSL1|OCIAD2|NDUFA13|ETFDH|EPHX2|CAPN1|ECHDC1|ME3|PHB2|ATP1A1|MMAB|RAN|SLC25A10|RPL18|MGAM|CD63|KRT8|ACSS3|ALDH6A1|NPC2|NDRG2|ACSM2A|CIRBP|HADH|DDX6|PHB|EPHX1|VPS29|PSMC3|FMO1|HAO2|GLYAT</td><td>1.66E-24</td><td>GO.0043227</td><td>2.20472076</td></tr>
<tr><td>574</td><td>70</td><td>GO Process</td><td>myeloid leukocyte activation</td><td>3E-22</td><td>VCL|ATP6V1D|PYGB|PSMD7|CTSH|LTF|CTSD|TTR|LGALS3|GGH|LYZ|HEXB|PSMD11|COTL1|PSMD3|ATP6V0A1|RAB7A|SLC27A2|IQGAP1|PSMA5|ARL8A|FTH1|LAMTOR1|AGL|APEH|ANPEP|GUSB|GAA|RAB6A|TOLLIP|PKM|AP2A2|ATP6V0C|LAMP1|ACAA1|DHRS2|PTGES2|VAPA|CTSB|GM2A|VCP|PNP|GSN|RAB14|CKAP4|APRT|TXNDC5|ASAH1|NME2|NIT2|PRKCD|PSAP|ALDOA|CD59|MGST1|AMPD3|IDH1|RHOA|GPI|LAMP2|AOC1|TOM1|SERPINA1|MME|PPIA|CAPN1|MGAM|CD63|NPC2|PSMC3</td><td>2.73E-24</td><td>GO.0002274</td><td>2.15214335</td></tr>
</table>

828	83	GO Component	secretory granule	7.3E-22	SYPL1|VCL|ATP6V1D|PYGB|PSMD7|CTSH|LTF|CTSD|APOA1|TTR|ACTN4|NAPSA|LGALS3|GGH|LYZ|HEXB|PSMD11|COTL1|PSMD3|ATP6V0A1|F13A1|RAB7A|SLC27A2|IQGAP1|PSMA5|ARL8A|FTH1|LAMTOR1|AGL|RNPEP|APEH|ANPEP|GUSB|TMED10|GAA|RAB6A|TOLLIP|PKM|AP2A2|ATP6V0C|SERPINA5|LAMP1|ACAA1|BSG|PTGES2|VAPA|CTSB|GM2A|VCP|PNP|CYB5R1|APOOL|GSN|RAB14|CKAP4|APRT|TXNDC5|ASAH1|CD9|NME2|NIT2|ACTN1|PRKCD|PSAP|ALDOA|CD59|MGST1|AMPD3|IDH1|ANXA11|RHOA|GPI|LAMP2|AOC1|TOM1|SERPINA1|MME|PPIA|CAPN1|MGAM|CD63|NPC2|PSMC3	1.46E-23	GO.0030141	2.11360826
959	90	GO Process	secretion by cell	1.1E-21	RALA|VCL|ATP6V1D|PYGB|PSMD7|CTSH|LTF|CTSD|APOA1|TTR|CANX|ACTN4|LGALS3|GGH|LYZ|HEXB|PSMD11|RAB11A|COTL1|PSMD3|ATP6V0A1|F13A1|RAB7A|SLC27A2|IQGAP1|PSMA5|ARL8A|FTH1|ENDOD1|LAMTOR1|AGL|APEH|SCIN|ANPEP|GUSB|TMED10|GAA|RAB6A|TOLLIP|TLN1|GLS|PKM|AP2A2|ATP6V0C|LAMP1|RAB11B|ACAA1|PTGES2|VAPA|CTSB|GM2A|VCP|MYH10|PNP|CYB5R1|TAGLN2|FLNA|APOOL|GSN|RAB14|CKAP4|APRT|TXNDC5|ASAH1|CD9|NME2|NIT2|ACTN1|PRKCD|PSAP|ALDOA|CD59|MGST1|AMPD3|SCRN1|IDH1|RHOA|GPI|LAMP2|AOC1|TOM1|SERPINA1|MME|PPIA|WDR1|CAPN1|MGAM|CD63|NPC2|PSMC3	1.03E-23	GO.0032940	2.0954677
198	43	GO Process	monosaccharide metabolic process	1.5E-21	PCK2|ENO1|GOT2|KHK|CPT1A|SORD|ABHD10|DLAT|CRYL1|GPD1|SLC23A1|DCXR|UGT2B7|GAA|PDHB|GPD2|PCK1|PKM|UGT2B17|TALDO1|MDH2|PKLR|CYB5A|APOD|PFKM|UGT1A9|GSTO1|GOT1|RBP4|AKR1A1|ALDOB|PDHA1|BPGM|PC|DAK|ALDOA|SLC25A12|TKT|GPI|FBP1|SLC25A10|GALK1|GALE	1.4E-23	GO.0005996	2.08297383
114	35	GO Process	cellular amino acid catabolic process	1.5E-21	HSD17B10|AHCY|CRYM|AGXT2|GOT2|HRSP12|BCKDHA|GLUD1|HNMT|QDPR|HGD|DDAH1|HPD|FTCD|PRODH2|GLS|PIPOX|SHMT1|BCAT2|DLST|HIBCH|ACADSB|GOT1|HOGA1|SARDH|HMGCL|ALDH4A1|AUH|GLDC|ABAT|MPST|TST|ALDH7A1|PAH|ALDH6A1	1.44E-23	GO.0009063	2.08268137
2318	148	GO Component	vesicle	2.1E-21	RALA|SYPL1|VCL|GNPNAT1|ATP6V1D|PYGB|AHCY|PSMD7|CTSH|EHD4|PLIN3|LTF|ATP6V1B1|CTSD|APOA1|TTR|CANX|ACTN4|ATP6V1E1|NAPSA|LGALS3|ARL8B|GGH|RAB21|LYZ|HEXB|PSMD11|CDH1|RAB11A|COTL1|RAB2A|PRDX1|PACSIN2|LRP2|CAPG|GRB14|RAB17|PSMD3|ATP6V0A1|F13A1|SCARB2|RAB7A|ENPEP|PRDX5|SORD|SLC27A2|RHCG|IQGAP1|PSMA5|ARL8A|FTH1|BHMT|ATP6V1B2|LAMTOR1|ATP6V0D2|AGL|RNPEP|APEH|AMN|TPP1|ANPEP|GUSB|TMED10|GAA|ATP6V0A4|RAB1B|AQP1|RAB6A|AOC3|DAB2|TOLLIP|PKM|EHD3|AP2A2|COPB2|ATP6V0C|TMED9|SERPINA5|LAMP1|RAB11B|ACAA1|BSG|LAMTOR4|PTGES2|VAPA|CTSB|ANXA6|SND1|GM2A|UBE2D3|VCP|DPP4|PNP|CYB5R1|MYO6|XPNPEP2|APOOL|GSN|RAB14|CLIC4|CLIC1|TBC1D4|CUBN|SLC3A2|CKAP4|APRT|TXNDC5|OCIAD1|ASAH1|GPNMB|CD9|NME2|CNP|NIT2|ACTN1|PRKCD|PSAP|ALDOA|CD59|MGST1|MYH11|AMPD3|ACY1|PDIA6|UBA52|IDH1|ANXA11|RHOA|DDC|GPI|LAMP2|AOC1|TOM1|SERPINA1|MME|PPIA|FLNB|TMEM33|OCIAD2|CAPN1|ATP1A1|RAN|MGAM|CD63|NPC2|PHB|VPS29|PSMC3	4.44E-23	GO.0031982	2.06861328
632	72	GO Process	leukocyte mediated immunity	2.2E-21	VCL|ATP6V1D|PYGB|PSMD7|CTSH|LTF|CTSD|TTR|LGALS3|GGH|LYZ|HEXB|PSMD11|COTL1|PRDX1|PSMD3|ATP6V0A1|RAB7A|SLC27A2|IQGAP1|PSMA5|ARL8A|FTH1|LAMTOR1|AGL|APEH|HPRT1|ANPEP|GUSB|GAA|RAB6A|TOLLIP|PKM|AP2A2|ATP6V0C|LAMP1|ACAA1|PTGES2|VAPA|CTSB|GM2A|VCP|PNP|GSN|RAB14|CKAP4|APRT|TXNDC5|ASAH1|NME2|NIT2|PRKCD|PSAP|ALDOA|CD59|MGST1|AMPD3|IDH1|RHOA|GPI|LAMP2|AOC1|TOM1|SERPINA1|MME|PPIA|WDR1|CAPN1|MGAM|CD63|NPC2|PSMC3	2.19E-23	GO.0002443	2.06575773

1083	95	GO Process	carbohydrate derivative metabolic process	4.3E-21	DCN|NDUFB4|NANS|GNPNAT1|AHCY|OGDH|PDHX|ACOT13|ATP6V1B1|ENO1|AKR7A2|PMPCB|NDUFA2|NDUFA10|KHK|HEXB|DHTKD1|ATP6V0A1|NDUFA9|SORD|ABHD10|ATP6V1A|ATP6V1B2|DPYS|NDUFB9|DLAT|GPD1L|CRYL1|HPRT1|NDUFB8|GPD1|GUSB|DCXR|HINT1|PDHB|CYCS|ATP6V0A4|GPD2|UQCRH|AQP1|GBAS|AHCYL2|ACSS1|CYC1|PIPOX|SHMT1|UGDH|PKM|TALDO1|NDUFV1|ACSM2B|NDUFA12|UQCR10|DLST|PKLR|PFKM|GM2A|VCP|PNP|OPA1|ATP1B1|NDUFS2|CMPK1|AKR1A1|PRPS1|OGDHL|HMGCL|HSPG2|ALDOB|APRT|ACOT9|PDHA1|AKR1C3|ASAH1|BPGM|NME2|DAK|PSAP|ALDOA|AK4|AMPD3|ATP5A1|TKT|NDUFS1|RHOA|GK|ACSF2|GPI|FBP1|ABHD14B|ACSL1|RAN|ACSM2A|GALK1|GLYAT	4.36E-23	GO.1901135	2.03716111
1070	94	GO Process	secretion	6.7E-21	RALA|VCL|ATP6V1D|PYGB|PSMD7|CTSH|LTF|CTSD|APOA1|TTR|GOT2|CANX|ACTN4|LGALS3|GGH|LYZ|HEXB|PSMD11|RAB11A|COTL1|SLC9A3R1|PSMD3|ATP6V0A1|F13A1|RAB7A|SLC27A2|IQGAP1|PSMA5|ARL8A|FTH1|ENDOD1|LAMTOR1|CA2|AGL|APEH|SCIN|ANPEP|GUSB|TMED10|GAA|AQP1|RAB6A|TOLLIP|TLN1|GLS|PKM|AP2A2|ATP6V0C|LAMP1|RAB11B|ACAA1|PTGES2|VAPA|CTSB|GM2A|VCP|MYH10|PNP|CYB5R1|TAGLN2|FLNA|APOOL|GSN|RAB14|CKAP4|APRT|TXNDC5|ASAH1|CD9|NME2|NIT2|ACTN1|PRKCD|PSAP|ALDOA|CD59|MGST1|AMPD3|SCRN1|IDH1|RHOA|GPI|LAMP2|AOC1|TOM1|SERPINA1|MME|PPIA|WDR1|CAPN1|MGAM|CD63|NPC2|PSMC3	6.98E-23	GO.0046903	2.01752235
616	70	GO Process	leukocyte activation involved in immune response	1E-20	VCL|ATP6V1D|PYGB|PSMD7|CTSH|LTF|CTSD|TTR|LGALS3|GGH|LYZ|HEXB|PSMD11|COTL1|PSMD3|ATP6V0A1|RAB7A|SLC27A2|IQGAP1|PSMA5|ARL8A|FTH1|LAMTOR1|AGL|APEH|ANPEP|GUSB|GAA|RAB6A|TOLLIP|PKM|AP2A2|ATP6V0C|LAMP1|ACAA1|PTGES2|VAPA|CTSB|GM2A|VCP|PNP|GSN|RAB14|CKAP4|APRT|TXNDC5|ASAH1|NME2|NIT2|PRKCD|PSAP|ALDOA|CD59|MGST1|AMPD3|IDH1|RHOA|GPI|LAMP2|AOC1|TOM1|SERPINA1|MME|PPIA|CAPN1|MGAM|CD63|NPC2|PSMC3|PLCG2	1.09E-22	GO.0002366	1.99913998
35	10	KEGG Pathways	Alanine, aspartate and glutamate metabolism	1.4E-06	AGXT2|GOT2|GLUD1|ASL|GLS|GOT1|ASS1|ALDH4A1|NIT2|ABAT	2.01E-07	hsa00250	0.58416375
1699	121	GO Process	vesicle-mediated transport	2.9E-20	RALA|VCL|MYH9|ATP6V1D|PYGB|F9|PSMD7|CTSH|EHD4|PLIN3|LTF|CTSD|APOA1|TTR|TFG|CANX|ACTN4|LGALS3|TINAG|GGH|LYZ|HEXB|PSMD11|RAB11A|COTL1|RAB2A|PACSIN2|NAPA|LRP2|RAB17|PSMD3|ATP6V0A1|F13A1|SCARB2|RAB7A|AMBP|SLC27A2|IQGAP1|TINAGL1|PSMA5|ARL8A|FTH1|ENDOD1|LAMTOR1|AGL|APEH|SCIN|AMN|ANPEP|GUSB|TMED10|GAA|DPYSL2|RAB1B|RAB6A|DAB2|TOLLIP|TLN1|PKM|EHD3|AP2A2|COPB2|ATP6V0C|TMED9|LAMP1|RAB11B|ACAA1|PTGES2|VAPA|CTSB|GM2A|VCP|ATP6V1H|MYH10|PNP|CYB5R1|TAGLN2|FLNA|MYO6|APOOL|GSN|RAB14|HSPG2|TBC1D4|CUBN|CKAP4|APRT|TXNDC5|ASAH1|CD9|KRT18|NME2|NIT2|ACTN1|PRKCD|PSAP|ALDOA|CD59|MGST1|AMPD3|PDIA6|UBA52|SCRN1|IDH1|ANXA11|RHOA|GPI|LAMP2|AOC1|TOM1|SERPINA1|MME|PPIA|WDR1|CAPN1|MGAM|CD63|NPC2|VPS29|PSMC3|PLCG2	3.22E-22	GO.0016192	1.95346171
311	50	GO Process	carboxylic acid biosynthetic process	3.5E-20	DCN|MTHFD1|AHCY|OGDH|AGXT2|ENO1|GOT2|BHMT2|DHTKD1|MECR|ALDH8A1|SLC27A2|BHMT|GLUD1|CBR1|ASL|ACSS1|GLS|SHMT1|UGDH|PKM|BCAT2|ACSM2B|SHMT2|PKLR|PTGES2|PFKM|MGST3|PHGDH|ABCD3|GOT1|HOGA1|SCP2|AKR1A1|ASS1|OGDHL|ALDOB|PSAT1|AKR1C3|BPGM|ALDOA|ABAT|GATM|GGT5|GGT1|GPI|EPHX2|PAH|ACSM2A|GALK1	3.94E-22	GO.0046394	1.9455932

<table>
<tr><td>948</td><td>86</td><td>GO Component</td><td>secretory vesicle</td><td>4E-20</td><td>SYPL1|VCL|ATP6V1D|PYGB|PSMD7|CTSH|LTF|ATP6V1B1|CTSD|APOA1|TTR|ACTN4|NAPSA|LGALS3|GGH|LYZ|HEXB|PSMD11|COTL1|PSMD3|ATP6V0A1|F13A1|RAB7A|SLC27A2|IQGAP1|PSMA5|ARL8A|FTH1|LAMTOR1|AGL|RNPEP|APEH|ANPEP|GUSB|TMED10|GAA|RAB6A|TOLLIP|PKM|AP2A2|ATP6V0C|SERPINA5|LAMP1|RAB11B|ACAA1|BSG|PTGES2|VAPA|CTSB|GM2A|VCP|PNP|CYB5R1|APOOL|GSN|RAB14|CKAP4|APRT|TXNDC5|ASAH1|CD9|NME2|NIT2|ACTN1|PRKCD|PSAP|ALDOA|CD59|MGST1|AMPD3|IDH1|ANXA11|RHOA|DDC|GPI|LAMP2|AOC1|TOM1|SERPINA1|MME|PPIA|CAPN1|MGAM|CD63|NPC2|PSMC3</td><td>9.3E-22</td><td>GO.0099503</td><td>1.9394695</td></tr>
<tr><td>185</td><td>40</td><td>GO Process</td><td>cellular modified amino acid metabolic process</td><td>5.2E-20</td><td>MTHFD1|AHCY|CRYM|ACADL|GOT2|DMGDH|BHMT2|GSTM3|GGH|BBOX1|CPT1A|NAT8|BHMT|FTCD|CKB|AHCYL2|PIPOX|SHMT1|CHDH|CNDP2|SHMT2|GSTA1|ALDH9A1|GSTO1|HOGA1|ACADM|SARDH|ASS1|ALDH4A1|DPEP1|GATM|HAGH|GGT5|GGT1|ALDH7A1|IDH1|CKMT2|CKMT1A|GPX1|GSTA2</td><td>6E-22</td><td>GO.0006575</td><td>1.92873503</td></tr>
<tr><td>2226</td><td>141</td><td>GO Component</td><td>cytoplasmic vesicle</td><td>5.8E-20</td><td>RALA|SYPL1|VCL|GNPNAT1|ATP6V1D|PYGB|AHCY|PSMD7|CTSH|EHD4|PLIN3|LTF|ATP6V1B1|CTSD|APOA1|TTR|CANX|ACTN4|ATP6V1E1|NAPSA|LGALS3|ARL8B|GGH|RAB21|LYZ|HEXB|PSMD11|CDH1|RAB11A|COTL1|RAB2A|PRDX1|PACSIN2|LRP2|CAPG|GRB14|RAB17|PSMD3|ATP6V0A1|F13A1|SCARB2|RAB7A|ENPEP|PRDX5|SLC27A2|RHCG|IQGAP1|PSMA5|ARL8A|FTH1|ATP6V1B2|LAMTOR1|ATP6V0D2|AGL|RNPEP|APEH|AMN|TPP1|ANPEP|GUSB|TMED10|GAA|ATP6V0A4|RAB1B|RAB6A|AOC3|DAB2|TOLLIP|PKM|EHD3|AP2A2|COPB2|ATP6V0C|TMED9|SERPINA5|LAMP1|RAB11B|ACAA1|BSG|LAMTOR4|PTGES2|VAPA|CTSB|ANXA6|SND1|GM2A|UBE2D3|VCP|DPP4|PNP|CYB5R1|MYO6|APOOL|GSN|RAB14|CLIC4|CUBN|SLC3A2|CKAP4|APRT|TXNDC5|OCIAD1|ASAH1|GPNMB|CD9|NME2|CNP|NIT2|ACTN1|PRKCD|PSAP|ALDOA|CD59|MGST1|MYH11|AMPD3|PDIA6|UBA52|IDH1|ANXA11|RHOA|DDC|GPI|LAMP2|AOC1|TOM1|SERPINA1|MME|PPIA|FLNB|TMEM33|OCIAD2|CAPN1|ATP1A1|RAN|MGAM|CD63|NPC2|PHB|VPS29|PSMC3</td><td>1.42E-21</td><td>GO.0031410</td><td>1.92380722</td></tr>
<tr><td>153</td><td>37</td><td>GO Process</td><td>cellular respiration</td><td>7E-20</td><td>NDUFB4|ACO2|IDH3G|OGDH|PMPCB|NDUFA2|NDUFA10|DHTKD1|NNT|NDUFA9|NDUFB9|DLAT|NDUFB8|IDH3A|GPD1|PDHB|CYCS|GPD2|ACO1|UQCRH|CYC1|NDUFV1|MDH2|NDUFA12|IDH2|UQCR10|DLST|CS|NDUFS2|OGDHL|PDHA1|IDH3B|SLC25A12|IDH1|NDUFS1|ETFDH|ME3</td><td>8.23E-22</td><td>GO.0045333</td><td>1.91580152</td></tr>
<tr><td>456</td><td>58</td><td>GO Component</td><td>mitochondrial inner membrane</td><td>1.8E-19</td><td>NDUFB4|PPIF|SLC25A3|GOT2|PMPCB|NDUFA2|NDUFA10|LGALS3|SQRDL|CHCHD3|NNT|ACAD11|NDUFA9|AFG3L2|ECSIT|NDUFB9|SLC25A4|NDUFB8|PRODH2|TMEM126A|CYCS|GPD2|UQCRH|CYC1|CHDH|NDUFV1|NDUFA12|UQCR10|SHMT2|AGK|MRPL37|MRPL24|MRPL21|OPA1|COA3|NDUFS2|CPT2|SLC25A5|APOOL|SLC25A6|FECH|BDH1|CNP|GATM|ATP5A1|IMMT|SLC25A12|NDUFS1|HIGD1A|CKMT2|CKMT1A|TOMM40|MRPS22|NDUFA13|ETFDH|PHB2|SLC25A10|PHB</td><td>5.09E-21</td><td>GO.0005743</td><td>1.87351822</td></tr>
<tr><td>135</td><td>35</td><td>GO Function</td><td>oxidoreductase activity, acting on CH-OH group of donors</td><td>3E-19</td><td>HSD17B10|IDH3G|EHHADH|AKR7A2|SORD|GPD1L|CBR1|CRYL1|IDH3A|GPD1|DCXR|ADH1B|GPD2|GRHPR|HSD11B2|UGDH|CHDH|DHRS4|MDH2|IDH2|CYB5A|DHRS2|ALDH3A2|AKR7A3|PHGDH|AKR1A1|AKR1C3|IDH3B|BDH1|PTGR1|IDH1|ME3|PTGR2|HADH|HAO2</td><td>1.56E-21</td><td>GO.0016614</td><td>1.8530178</td></tr>
<tr><td>221</td><td>42</td><td>GO Process</td><td>purine ribonucleoside triphosphate metabolic process</td><td>3.3E-19</td><td>NDUFB4|OGDH|ATP6V1B1|ENO1|PMPCB|NDUFA2|NDUFA10|DHTKD1|ATP6V0A1|NDUFA9|ATP6V1A|ATP6V1B2|NDUFB9|NDUFB8|CYCS|ATP6V0A4|UQCRH|GBAS|CYC1|PKM|NDUFV1|NDUFA12|UQCR10|PKLR|PFKM|VCP|OPA1|ATP1B1|NDUFS2|OGDHL|ALDOB|BPGM|NME2|ALDOA|AK4|AMPD3|ATP5A1|NDUFS1|RHOA|GPI|RAN|GALK1</td><td>3.91E-21</td><td>GO.0009205</td><td>1.84881166</td></tr>
<tr><td>262</td><td>45</td><td>GO Process</td><td>nucleoside monophosphate metabolic process</td><td>4.2E-19</td><td>NDUFB4|OGDH|DNPH1|ATP6V1B1|ENO1|PMPCB|NDUFA2|NDUFA10|DHTKD1|ATP6V0A1|NDUFA9|ATP6V1A|ATP6V1B2|NDUFB9|HPRT1|NDUFB8|CYCS|ATP6V0A4|UQCRH|GBAS|CYC1|SHMT1|PKM|NDUFV1|NDUFA12|UQCR10|PKLR|PFKM|VCP|ATP1B1|NDUFS2|LHPP|CMPK1|PRPS1|OGDHL|ALDOB|APRT|BPGM|ALDOA|AK4|AMPD3|ATP5A1|NDUFS1|GPI|GALK1</td><td>5.09E-21</td><td>GO.0009123</td><td>1.83809067</td></tr>
</table>

5162	240	GO Component	intracellular organelle lumen	4.9E-19	PSMA4|DCN|HSD17B10|VCL|ACO2|PCK2|PYGB|IDH3G|F9|PSMD7|CRYM|CTSH|ECH1|HNRNPL|OGDH|PPIF|PDHX|AGXT2|LTF|EHHADH|ACADL|CTSD|APOA1|TTR|ACADS|PEPD|GOT2|CANX|PMPCB|RPS16|ACTN4|NDUFA10|NAPSA|GRSF1|HRSP12|DMGDH|GGH|LYZ|HEXB|PSMD11|ALDH2|DNAJA3|COTL1|DHTKD1|PACSIN2|MECR|LRP2|CAPG|PSMD3|ATP6V0A1|ADD1|F13A1|SCARB2|VDAC1|PRDX5|REXO2|NDUFA9|SLC27A2|BCKDHA|ECSIT|PSMA5|ABHD10|FTH1|GLUD1|DLAT|UCHL1|ACAA2|PSMB4|AGL|FABP1|APEH|HSPA9|COL14A1|CFL2|NDUFB8|TPP1|IDH3A|COL6A2|ECI1|GUSB|RPS9|HINT1|GAA|ADH1B|PDHB|CYCS|HNRNPD|DAB2|GRHPR|TSFM|MSRA|TOLLIP|ACSS1|GLS|PIPOX|CES2|UGDH|PKM|TALDO1|BCAT2|HNRNPM|CNDP2|DHRS4|MDH2|ACSM2B|C11orf54|IDH2|ACAA1|SHMT2|DLST|CS|PPA2|DHRS2|PTGES2|CTSB|AGK|COL18A1|GM2A|UBE2D3|VCP|HIBCH|COL4A2|LONP1|MRPL37|MRPL24|PNP|MRPL21|OPA1|COL6A1|IBA57|NDUFS2|ECHS1|LHPP|ACADSB|FLNA|MYO6|ABCD3|RPL5|GOT1|HOGA1|ACADM|CPT2|SCP2|SLC25A5|SARDH|CMPK1|APOOL|GSN|OGDHL|CLIC4|HMGCL|HNRNPR|HSPG2|PSMB9|PSMB8|ALDH4A1|AUH|COL4A1|ALDH1B1|HIST1H2AC|CUBN|CKAP4|APRT|ACOT9|TXNDC5|PDHA1|NQO2|IDH3B|GLDC|ASAH1|FECH|KRT18|BDH1|NME2|CNP|PC|NIT2|ACTN1|PRKCD|PSAP|ALDOA|KTN1|AK4|UMOD|AMPD3|ABAT|GATM|ILK|MPST|HAGH|ATP5A1|TXNRD2|IFI30|ELAVL1|PDIA6|TST|ALDH7A1|UBA52|IDH1|TKT|NDUFS1|HIGD1A|ANXA11|ACSF2|GPI|GPX1|LAMP2|LARS2|AOC1|SERPINA1|VWA1|MRPS22|GFM1|PPIA|SSBP1|ABHD14B|NDUFA13|ETFDH|EPHX2|CAPN1|ME3|PHB2|MMAB|RAN|SLC25A10|RPL18|CD63|KRT8|ACSS3|ALDH6A1|NPC2|ACSM2A|CIRBP|HADH|DDX6|PHB|PSMC3|FMO1|HAO2|GLYAT	1.43E-20	GO.0070013	1.83106911
958	85	GO Process	organonitrogen compound catabolic process	5E-19	PSMA4|DCN|HSD17B10|AHCY|PSMD7|CRYM|CTSH|DNPH1|AGXT2|ACADL|CTSD|GDA|GOT2|NAPSA|HRSP12|DMGDH|HEXB|PSMD11|PSMD3|RAB7A|AMBP|ENPEP|BCKDHA|PSMA5|BHMT|ERLIN2|DPYS|GLUD1|HNMT|QDPR|HGD|DDAH1|UCHL1|HPD|PSMB4|FTCD|CAPN2|RNPEP|HPRT1|TPP1|ANPEP|PRODH2|GUSB|HINT1|TOLLIP|GLS|PIPOX|SHMT1|CHDH|BCAT2|AP2A2|DLST|CTSB|GM2A|UBE2D3|VCP|HIBCH|LONP1|PNP|ACADSB|GOT1|HOGA1|SARDH|HMGCL|HSPG2|PSMB9|PSMB8|ALDH4A1|AUH|MAOB|GLDC|QPRT|AMPD3|ABAT|MPST|GGT5|GGT1|TST|ALDH7A1|UBA52|GPX1|LAMP2|PAH|ALDH6A1|PSMC3	6.23E-21	GO.1901565	1.82992963
242	43	GO Process	ribonucleoside monophosphate metabolic process	9.7E-19	NDUFB4|OGDH|ATP6V1B1|ENO1|PMPCB|NDUFA2|NDUFA10|DHTKD1|ATP6V0A1|NDUFA9|ATP6V1A|ATP6V1B2|NDUFB9|HPRT1|NDUFB8|CYCS|ATP6V0A4|UQCRH|GBAS|CYC1|PKM|NDUFV1|NDUFA12|UQCR10|PKLR|PFKM|VCP|ATP1B1|NDUFS2|LHPP|CMPK1|PRPS1|OGDHL|ALDOB|APRT|BPGM|ALDOA|AK4|AMPD3|ATP5A1|NDUFS1|GPI|GALK1	1.26E-20	GO.0009161	1.80150229
186	38	GO Component	ficolin-1-rich granule	1.1E-18	VCL|PSMD7|CTSH|CTSD|LGALS3|PSMD11|COTL1|PSMD3|ATP6V0A1|PSMA5|ARL8A|FTH1|LAMTOR1|AGL|APEH|GUSB|GAA|PKM|AP2A2|ATP6V0C|LAMP1|CTSB|VCP|PNP|GSN|ASAH1|NME2|ALDOA|AMPD3|IDH1|RHOA|GPI|LAMP2|SERPINA1|PPIA|CAPN1|MGAM|PSMC3	3.62E-20	GO.0101002	1.79706162
217	41	GO Process	energy derivation by oxidation of organic compounds	1.1E-18	NDUFB4|ACO2|PYGB|IDH3G|OGDH|PMPCB|NDUFA2|NDUFA10|DHTKD1|NNT|NDUFA9|NDUFB9|DLAT|AGL|NDUFB8|IDH3A|GPD1|GAA|PDHB|CYCS|GPD2|ACO1|UQCRH|CYC1|NDUFV1|MDH2|NDUFA12|IDH2|UQCR10|DLST|CS|PFKM|NDUFS2|OGDHL|PDHA1|IDH3B|SLC25A12|IDH1|NDUFS1|ETFDH|ME3	1.42E-20	GO.0015980	1.79706162
230	42	GO Process	purine ribonucleoside monophosphate metabolic process	1.1E-18	NDUFB4|OGDH|ATP6V1B1|ENO1|PMPCB|NDUFA2|NDUFA10|DHTKD1|ATP6V0A1|NDUFA9|ATP6V1A|ATP6V1B2|NDUFB9|HPRT1|NDUFB8|CYCS|ATP6V0A4|UQCRH|GBAS|CYC1|PKM|NDUFV1|NDUFA12|UQCR10|PKLR|PFKM|VCP|ATP1B1|NDUFS2|LHPP|PRPS1|OGDHL|ALDOB|APRT|BPGM|ALDOA|AK4|AMPD3|ATP5A1|NDUFS1|GPI|GALK1	1.46E-20	GO.0009167	1.79625735

106	31	GO Process	monocarboxylic acid catabolic process	1.2E-18	ECH1|AGXT2|EHHADH|ACADL|ACADS|MECR|ACAD11|CPT1A|SORD|SLC27A2|ACAA2|CRYL1|ECI1|DCXR|CYP4A11|ACAA1|ALDH3A2|ECHS1|ABCD3|HOGA1|ACADM|CPT2|SCP2|ECHDC2|AKR1A1|AUH|ABAT|ETFDH|ECHDC1|HADH|HAO2	1.71E-20	GO.0072329	1.79065783
246	43	GO Process	nucleoside triphosphate metabolic process	1.6E-18	NDUFB4|OGDH|ATP6V1B1|ENO1|PMPCB|NDUFA2|NDUFA10|DHTKD1|ATP6V0A1|NDUFA9|ATP6V1A|ATP6V1B2|NDUFB9|NDUFB8|CYCS|ATP6V0A4|UQCRH|GBAS|CYC1|PKM|NDUFV1|NDUFA12|UQCR10|PKLR|PFKM|VCP|OPA1|ATP1B1|NDUFS2|CMPK1|OGDHL|ALDOB|BPGM|NME2|ALDOA|AK4|AMPD3|ATP5A1|NDUFS1|RHOA|GPI|RAN|GALK1	2.18E-20	GO.0009141	1.78068754
1146	92	GO Component	organelle envelope	1.8E-18	NDUFB4|OGDH|FIS1|SFXN3|PPIF|SLC25A3|ACADL|GOT2|PMPCB|NDUFA2|NDUFA10|LGALS3|SYNJ2BP|SQRDL|CHCHD3|NNT|ACAD11|VDAC1|CPT1A|REXO2|NDUFA9|SORD|AFG3L2|ECSIT|NDUFB9|SLC25A4|APEH|NDUFB8|PRODH2|TMEM126A|CYCS|GPD2|UQCRH|AQP1|GBAS|SFXN1|CYC1|CHDH|RHOT2|NDUFV1|NDUFA12|UQCR10|SHMT2|CYB5A|DHRS2|VAPA|AGK|MRPL37|MRPL24|MRPL21|OPA1|COA3|MARC2|MGST3|NDUFS2|SFXN2|MYO6|ACADM|CPT2|SLC25A5|ASS1|APOOL|CLIC1|MAOB|BPHL|SLC25A6|FECH|BDH1|CNP|MGST1|GATM|ATP5A1|IMMT|UBA52|SLC25A12|SCRN1|NDUFS1|HIGD1A|ANXA11|GK|CKMT2|CKMT1A|TOMM40|MRPS22|TMEM33|ACSL1|NDUFA13|ETFDH|PHB2|RAN|SLC25A10|PHB	6.44E-20	GO.0031967	1.77399286

10365	383	GO Component	intracellular membrane-bounded organelle	2E-18	PSMA4|DCN|HSD17B10|NDUFB4|EPDR1|NIPSNAP1|MYH9|ACO2|GNPNAT1|MTHFD1|PCK2|PYGB|AHCY|APMAP|TXNL1|IDH3G|F9|PSMD7|CRYM|CTSH|EHD4|ECH1|HNRNPL|PLIN3|OGDH|FIS1|SFXN3|PPIF|PDHX|SLC25A3|ACOT13|DNPH1|AGXT2|LTF|EHHADH|REEP6|ACADL|ATP6V1B1|ENO1|AKR7A2|CTSD|APOA1|TTR|PLBD1|TFG|ACADS|PEPD|GOT2|CANX|HSPB1|PMPCB|RPS16|NDUFA2|ACTN4|NDUFA10|NAPSA|LGALS3|GRSF1|HRSP12|DMGDH|SYNJ2BP|ARL8B|GGH|SQRDL|RAB21|LYZ|HEXB|PSMD11|ALDH2|CDH1|RAB11A|DNAJA3|COTL1|CHCHD3|RAB2A|PRDX1|DHTKD1|PACSIN2|NAPA|MECR|LRP2|CAPG|GRB14|PSMD3|ATP6V0A1|NNT|ADD1|SCARB2|ACAD11|RAB7A|AMBP|VDAC1|CALB1|PRDX5|CPT1A|REXO2|NDUFA9|SORD|SLC27A2|IQGAP1|AFG3L2|BCKDHA|ECSIT|PSMA5|ARL8A|NAT8|ABHD10|FTH1|ATP6V1B2|ERLIN2|NDUFB9|GLUD1|LAMTOR1|DLAT|SLC25A4|UCHL1|ACAA2|ATP6V0D2|HPD|PSMB4|FTCD|AGL|CAPN2|FABP1|APEH|HSPA9|COL14A1|CFL2|NDUFB8|CKB|TPP1|IDH3A|ANPEP|GLYATL1|COL6A2|PRODH2|ECI1|GUSB|RPS9|TMED10|HINT1|UGT2B7|GAA|YWHAG|ADH1B|TMEM126A|PDHB|CYCS|ATP6V0A4|GPD2|ACO1|UQCRH|RAB1B|CSRP2|CYP4A11|AQP1|RAB6A|AOC3|GBAS|HNRNPD|DAB2|GRHPR|TSFM|MSRA|TOLLIP|AHCYL2|HSD11B2|SFXN1|ACSS1|CYC1|GLS|PIPOX|CES2|SHMT1|UGDH|CHDH|PKM|UGT2B17|TALDO1|RHOT2|NDUFV1|BCAT2|HNRNPM|CNDP2|DHRS4|MDH2|EHD3|ACSM2B|AP2A2|COPB2|ATP6V0C|NDUFA12|TMED9|C11orf54|IDH2|UQCR10|SERPINA5|LAMP1|ACAA1|SHMT2|BSG|DLST|CYB5A|CS|LAMTOR4|PPA2|DHRS2|APOD|PTGES2|VAPA|CTSB|PFKM|ALDH3A2|ANXA6|SND1|UGT1A9|WARS|AGK|COL18A1|GM2A|UBE2D3|VCP|ATP6V1H|HIBCH|COL4A2|LONP1|MRPL37|MRPL24|PNP|MRPL21|OPA1|COA3|COL6A1|IBA57|MARC2|CYB5R1|CSRP1|MGST3|NDUFS2|ECHS1|LHPP|ACADSB|FLNA|SFXN2|MYO6|ABCD3|RPL5|GOT1|HOGA1|ACADM|CPT2|SCP2|ECHDC2|SLC25A5|SARDH|CMPK1|ASS1|APOOL|RAB14|ATP6V1G1|OGDHL|CLIC4|HMGCL|HNRNPR|HSPG2|PSMB9|PSMB8|ALDH4A1|AUH|CLIC1|COL4A1|AGMAT|ALDH1B1|HIST1H2AC|CUBN|SLC3A2|CKAP4|MAOB|APRT|ACOT9|TXNDC5|PDHA1|MT1M|BPHL|NQO2|NAP1L4|AKR1C3|IDH3B|GLDC|SLC25A6|ASAH1|FECH|KRT18|SACM1L|BDH1|DPEP1|CNP|PC|FHL1|PRKCD|PSAP|KTN1|AK4|CD59|UMOD|MGST1|ABAT|GATM|ILK|MPST|HAGH|ATP5A1|SEPT7|TXNRD2|IFI30|ELAVL1|PDIA6|TST|ALDH7A1|IMMT|UBA52|SLC25A12|SCRN1|IDH1|TKT|NDUFS1|GDI1|HIGD1A|ANXA11|RHOA|GK|ACSF2|TANGO2|TMED4|CKMT2|GPI|CKMT1A|GPX1|FBP1|LAMP2|LARS2|TOMM40|AOC1|TOM1|SERPINA1|TGFBI|VWA1|MRPS22|GFM1|FAM162A|SSBP1|ABHD14B|TMEM33|ACSL1|NDUFA13|ETFDH|EPHX2|CAPN1|ECHDC1|ME3|PHB2|ATP1A1|MMAB|RAN|SLC25A10|RPL18|CD63|KRT8|ACSS3|ALDH6A1|NPC2|NDRG2|ACSM2A|CIRBP|HADH|DDX6|PHB|EPHX1|VPS29|PSMC3|FMO1|HAO2|GLYAT	7.76E-20	GO.0043231	1.76946486
190	38	GO Process	ATP metabolic process	4.8E-18	NDUFB4|OGDH|ATP6V1B1|ENO1|PMPCB|NDUFA2|NDUFA10|DHTKD1|ATP6V0A1|NDUFA9|ATP6V1A|ATP6V1B2|NDUFB9|NDUFB8|CYCS|ATP6V0A4|UQCRH|GBAS|CYC1|PKM|NDUFV1|NDUFA12|UQCR10|PKLR|PFKM|VCP|ATP1B1|NDUFS2|OGDHL|ALDOB|BPGM|ALDOA|AK4|AMPD3|ATP5A1|NDUFS1|GPI|GALK1	6.86E-20	GO.0046034	1.73160529
113	31	GO Process	pyridine-containing compound metabolic process	5.7E-18	OGDH|PDHX|ENO1|DHTKD1|NNT|PRDX5|GPD1L|GPD1|DCXR|PDHB|GPD2|PKM|TALDO1|MDH2|IDH2|PKLR|PFKM|VCP|PNP|OGDHL|ALDOB|PSAT1|PDHA1|BPGM|ALDOA|QPRT|IDH1|TKT|GPI|GALK1|FMO1	8.2E-20	GO.0072524	1.72448877
107	30	GO Process	nicotinamide nucleotide metabolic process	1.3E-17	OGDH|PDHX|ENO1|DHTKD1|NNT|PRDX5|GPD1L|GPD1|DCXR|PDHB|GPD2|PKM|TALDO1|MDH2|IDH2|PKLR|PFKM|VCP|PNP|OGDHL|ALDOB|PDHA1|BPGM|ALDOA|QPRT|IDH1|TKT|GPI|GALK1|FMO1	1.94E-19	GO.0046496	1.68761484
98	29	GO Process	alpha-amino acid catabolic process	1.5E-17	AHCY|CRYM|AGXT2|GOT2|HRSP12|GLUD1|HNMT|QDPR|HGD|DDAH1|HPD|FTCD|PRODH2|GLS|PIPOX|SHMT1|BCAT2|DLST|HIBCH|GOT1|HOGA1|SARDH|HMGCL|ALDH4A1|AUH|GLDC|ALDH7A1|PAH|ALDH6A1	2.28E-19	GO.1901606	1.68181564

103	29	GO Component	oxidoreductase complex	1.8E-17	NDUFB4|OGDH|PDHX|PMPCB|NDUFA2|NDUFA10|DHTKD1|NDUFA9|BCKDHA|NDUFB9|DLAT|GPD1L|NDUFB8|GPD1|PDHB|GPD2|UQCRH|CYC1|NDUFV1|NDUFA12|UQCR10|DLST|NDUFS2|OGDHL|PDHA1|GLDC|NDUFS1|NDUFA13|ETFDH	7.12E-19	GO.1990204	1.674958
8808	341	GO Process	primary metabolic process	2.4E-17	PSMA4|DCN|HSD17B10|NDUFB4|NANS|MYH9|ACO2|GNPNAT1|MTHFD1|PCK2|PYGB|AHCY|IDH3G|F9|PSMD7|CRYM|CTSH|ECH1|HNRNPL|CDC37|OGDH|PPIF|PDHX|ACOT13|DNPH1|AGXT2|LTF|EHHADH|ACADL|ATP6V1B1|ENO1|AKR7A2|CTSD|APOA1|TTR|GDA|PLBD1|ACADS|PEPD|GOT2|CAPNS1|PMPCB|RPS16|NDUFA2|NDUFA10|NAPSA|LGALS3|GRSF1|HRSP12|APCS|BHMT2|NARS|TINAG|GGH|KHK|LYZ|HEXB|PSMD11|ALDH2|RAB11A|DNAJA3|CHCHD3|RAB2A|DHTKD1|MECR|LRP2|PSMD3|ATP6V0A1|NNT|ADD1|F13A1|SCARB2|ACAD11|RAB7A|AMBP|ENPEP|CALB1|PRDX5|ALDH8A1|CPT1A|REXO2|NDUFA9|SORD|SLC27A2|AFG3L2|BCKDHA|TINAGL1|PSMA5|NAT8|ABHD10|ATP6V1A|BHMT|ATP6V1B2|ERLIN2|DPYS|NDUFB9|GLUD1|ENDOD1|HNMT|DLAT|QDPR|GPD1L|HGD|DDAH1|UCHL1|ACAA2|HPD|CBR1|PSMB4|FTCD|AGL|CAPN2|RNPEP|FABP1|APEH|CRYL1|HPRT1|NDUFB8|CKB|TPP1|IDH3A|ANPEP|GLYATL1|GPD1|PRODH2|ECI1|GUSB|SLC23A1|RPS9|DCXR|HINT1|UGT2B7|GAA|ASL|PDHB|CYCS|ATP6V0A4|GPD2|ACO1|DPYSL2|UQCRH|RAB1B|CYP4A11|AQP1|RAB6A|GBAS|HNRNPD|TSFM|MSRA|TOLLIP|AHCYL2|HSD11B2|ACSS1|CYC1|GLS|PIPOX|CES2|SHMT1|UGDH|PCK1|PKM|SLC7A8|UGT2B17|TALDO1|NDUFV1|BCAT2|HNRNPM|CNDP2|DHRS4|MDH2|ACSM2B|AP2A2|SLC5A2|NDUFA12|IDH2|UQCR10|RAB11B|ACAA1|SHMT2|DLST|GSTA1|TRIM2|PKLR|CYB5A|CS|PPA2|DHRS2|APOD|PTGES2|VAPA|CTSB|PFKM|ALDH3A2|SND1|UGT1A9|WARS|AGK|GM2A|UBE2D3|VCP|HIBCH|COL4A2|DPP4|LONP1|MRPL37|MRPL24|PNP|MRPL21|OPA1|CYB5R1|ATP1B1|MGST3|NDUFS2|ECHS1|LHPP|ACADSB|PHGDH|GSTO1|FLNA|ABCD3|RPL5|GOT1|HOGA1|ACADM|XPNPEP2|RBP4|CPT2|SCP2|ECHDC2|SARDH|CMPK1|AKR1A1|ASS1|PRPS1|GSN|RAB14|OGDHL|HMGCL|HNRNPR|HSPG2|ALDOB|PSMB9|PSMB8|ALDH4A1|VARS|AUH|AGMAT|PSAT1|ALDH1B1|CUBN|SLC3A2|CKAP4|APRT|ACOT9|PDHA1|BPHL|AKR1C3|IDH3B|GLDC|ASAH1|SACM1L|DPEP1|BPGM|NME2|CNP|PC|NIT2|PRKCD|DAK|PSAP|ALDOA|KTN1|AK4|QPRT|AMPD3|ABAT|GATM|ILK|MPST|GGT5|ATP5A1|GGT1|ACY1|ELAVL1|PDIA6|PTGR1|TST|ALDH7A1|UBA52|SLC25A12|SCRN1|IDH1|TKT|NDUFS1|RHOA|GK|ACSF2|DDC|CKMT2|GPI|CKMT1A|GPX1|FBP1|LAMP2|LARS2|SERPINA1|TGFBI|CFB|VWA1|MRPS22|MME|GFM1|PPIA|SSBP1|ABHD14B|ACSL1|ETFDH|EPHX2|CAPN1|ECHDC1|ME3|PHB2|RAN|SLC25A10|RPL18|MGAM|PAH|ALDH6A1|NPC2|PTGR2|ACSM2A|GALK1|HADH|DDX6|PHB|PSMC3|FMO1|PLCG2|GALE|HAO2|GLYAT	3.61E-19	GO.0044238	1.6623423
105	29	GO Component	melanosome	2.6E-17	SYPL1|AHCY|CTSD|CANX|GGH|RAB2A|PRDX1|CAPG|RAB17|ATP6V0A1|RAB7A|ATP6V1B2|TPP1|TMED10|LAMP1|BSG|CTSB|ANXA6|SND1|SLC3A2|GPNMB|CNP|MYH11|PDIA6|ANXA11|TMEM33|ATP1A1|RAN|CD63	1.11E-18	GO.0042470	1.65900669
343	48	GO Process	sulfur compound metabolic process	3.1E-17	DCN|MTHFD1|AHCY|OGDH|PDHX|ACOT13|BHMT2|GSTM3|SQRDL|HEXB|NAT8|BHMT|DLAT|HSPA9|PDHB|MSRA|AHCYL2|ACSS1|PIPOX|CNDP2|ACSM2B|DLST|GSTA1|IBA57|MGST3|PHGDH|GSTO1|AKR1A1|HMGCL|ACOT9|PDHA1|DPEP1|PC|MGST1|MPST|HAGH|GGT5|GGT1|TST|IDH1|ACSF2|GPX1|ABHD14B|GSTA2|ACSL1|SLC25A10|ACSM2A|GLYAT	4.69E-19	GO.0006790	1.65157002
118	31	GO Function	oxidoreductase activity, acting on the CH-OH group of donors, NAD or NADP as acceptor	3.8E-17	HSD17B10|IDH3G|EHHADH|AKR7A2|SORD|GPD1L|CBR1|CRYL1|IDH3A|GPD1|DCXR|ADH1B|GRHPR|HSD11B2|UGDH|DHRS4|MDH2|IDH2|CYB5A|DHRS2|AKR7A3|PHGDH|AKR1A1|AKR1C3|IDH3B|BDH1|PTGR1|IDH1|ME3|PTGR2|HADH	2.37E-19	GO.0016616	1.64156688

3559	183	GO Process	regulation of biological quality	4E-17	DCN|GNA11|CA12|MYH9|MTHFD1|TXNL1|F9|CRYM|CTSH|CDC37|FIS1|SFXN3|PPIF|AGXT2|LTF|ACADL|ATP6V1B1|APOA1|TTR|PFN2|GOT2|VIL1|HSPB1|NAPSA|HRSP12|DMGDH|RAB21|HEXB|RAB11A|DNAJA3|SLC4A1|SLC9A3R1|PRDX1|NAPA|CAPG|RAB17|ATP6V0A1|NNT|ADD1|F13A1|SCARB2|RAB7A|ENPEP|CALB1|PRDX5|ALDH8A1|CPT1A|RHCG|AFG3L2|ATP6V1A|FTH1|BHMT|DPYS|GLUD1|LAMTOR1|HNMT|SLC25A4|GPD1L|DDAH1|ACAA2|CA2|ATP6V0D2|RNPEP|SCIN|HSPA9|CFL2|CKB|TPP1|ANPEP|RPS9|UGT2B7|GAA|YWHAG|ATP6V0A4|ACO1|DPYSL2|CYP4A11|AQP1|HNRNPD|TLN1|HSD11B2|SFXN1|SHMT1|PCK1|CHDH|SLC7A8|RHOT2|BCAT2|DHRS4|EHD3|ATP6V0C|SERPINA5|LAMP1|RAB11B|SHMT2|PARVA|LAMTOR4|EPB41L3|PPA2|DHRS2|PTGES2|PFKM|ANXA6|UGT1A9|ALDH9A1|GM2A|PBLD|VCP|ATP6V1H|MSN|MYH10|DPP4|OPA1|ATP1B1|PHGDH|GSTO1|FLNA|SFXN2|RPL5|GOT1|RBP4|SCP2|SLC25A5|SARDH|ASS1|GSN|ATP6V1G1|CLIC4|HNRNPR|VARS|CLIC1|CUBN|MAOB|APRT|TXNDC5|AKR1C3|GLDC|SLC25A6|CD9|PRKAR1A|BPGM|CNP|FHL1|PRKCD|ALDOA|CD59|UMOD|AMPD3|ABAT|ILK|SEPT7|TXNRD2|ELAVL1|PDIA6|ALDH7A1|IMMT|UBA52|NDUFS1|SLC4A4|GDI1|RHOA|DDC|GPI|GPX1|LAMP2|LARS2|SERPINA1|MME|FLNB|WDR1|EPHX2|PHB2|ATP1A1|PAH|TWF1|NPC2|ACSM2A|CIRBP|HADH|PHB|PDLIM5|PLCG2|GLYAT	6.18E-19	GO.0065008	1.64012095
3337	174	GO Component	organelle membrane	4.2E-17	RALA|SYPL1|NDUFB4|GNPNAT1|ATP6V1D|EHD4|PLIN3|OGDH|FIS1|SFXN3|PPIF|SLC25A3|REEP6|ACADL|ATP6V1B1|TFG|GOT2|CANX|PMPCB|NDUFA2|NDUFA10|LGALS3|SYNJ2BP|ARL8B|SQRDL|RAB21|RAB11A|CHCHD3|RAB2A|PACSIN2|NAPA|LRP2|GRB14|RAB17|ATP6V0A1|NNT|SCARB2|ACAD11|RAB7A|VDAC1|CPT1A|NDUFA9|SORD|SLC27A2|IQGAP1|AFG3L2|ECSIT|ARL8A|NAT8|ERLIN2|NDUFB9|LAMTOR1|SLC25A4|UCHL1|ATP6V0D2|HPD|FTCD|APEH|AMN|NDUFB8|ANPEP|PRODH2|TMED10|UGT2B7|GAA|TMEM126A|CYCS|ATP6V0A4|GPD2|UQCRH|RAB1B|CYP4A11|AQP1|RAB6A|GBAS|DAB2|HSD11B2|SFXN1|CYC1|CHDH|UGT2B17|RHOT2|NDUFV1|DHRS4|EHD3|AP2A2|COPB2|ATP6V0C|NDUFA12|TMED9|UQCR10|SERPINA5|LAMP1|RAB11B|SHMT2|BSG|CYB5A|LAMTOR4|PTGES2|VAPA|ALDH3A2|ANXA6|UGT1A9|AGK|UBE2D3|VCP|ATP6V1H|MRPL37|MRPL24|MRPL21|OPA1|COA3|MARC2|CYB5R1|MGST3|NDUFS2|SFXN2|MYO6|ACADM|CPT2|SLC25A5|ASS1|APOOL|RAB14|ATP6V1G1|CLIC4|CLIC1|CUBN|CKAP4|MAOB|BPHL|SLC25A6|CD9|FECH|SACM1L|BDH1|CNP|PSAP|KTN1|CD59|UMOD|MGST1|GATM|ATP5A1|PDIA6|IMMT|UBA52|SLC25A12|SCRN1|NDUFS1|HIGD1A|RHOA|GK|TMED4|CKMT2|CKMT1A|LAMP2|TOMM40|TOM1|SERPINA1|MRPS22|MME|TMEM33|ACSL1|NDUFA13|ETFDH|PHB2|SLC25A10|MGAM|CD63|PHB|EPHX1|VPS29|FMO1	1.93E-18	GO.0031090	1.63788237
894	78	GO Process	leukocyte activation	5E-17	VCL|MYH9|ATP6V1D|PYGB|PSMD7|CTSH|LTF|CTSD|TTR|LGALS3|GGH|LYZ|HEXB|PSMD11|DNAJA3|COTL1|PRDX1|PSMD3|ATP6V0A1|RAB7A|SLC27A2|IQGAP1|PSMA5|ARL8A|FTH1|LAMTOR1|AGL|APEH|HPRT1|ANPEP|GUSB|GAA|RAB6A|TOLLIP|PKM|AP2A2|ATP6V0C|LAMP1|ACAA1|DHRS2|PTGES2|VAPA|CTSB|GM2A|VCP|MSN|DPP4|PNP|GSN|RAB14|CKAP4|APRT|TXNDC5|ASAH1|NME2|NIT2|PRKCD|PSAP|ALDOA|CD59|MGST1|AMPD3|IDH1|RHOA|GPI|LAMP2|AOC1|TOM1|SERPINA1|MME|PPIA|KIF13B|CAPN1|MGAM|CD63|NPC2|PSMC3|PLCG2	7.93E-19	GO.0045321	1.63018995
169	35	GO Process	electron transport chain	5E-17	NDUFB4|AKR7A2|PMPCB|NDUFA2|NDUFA10|DMGDH|ALDH2|NDUFA9|NDUFB9|QDPR|NDUFB8|GPD1|CYCS|GPD2|UQCRH|CYC1|UGDH|NDUFV1|NDUFA12|UQCR10|CYB5A|PTGES2|AKR7A3|NDUFS2|PHGDH|AKR1A1|ALDH4A1|MAOB|NQO2|IDH3B|GLDC|TXNRD2|SLC25A12|NDUFS1|ETFDH	7.87E-19	GO.0022900	1.63018995
124	31	GO Process	antibiotic metabolic process	5E-17	ACO2|IDH3G|OGDH|AKR7A2|ALDH2|PRDX1|DHTKD1|NNT|PRDX5|DLAT|FTCD|IDH3A|ADH1B|PDHB|ACO1|ACSS1|MDH2|IDH2|DLST|CS|OGDHL|ALDH1B1|MAOB|PDHA1|AKR1C3|IDH3B|GPX3|DPEP1|IDH1|GPX1|ME3	8.02E-19	GO.0016999	1.63018995

<table>
<tr><td>124</td><td>31</td><td>GO Process</td><td>oxidoreduction coenzyme metabolic process</td><td>5E-17</td><td>OGDH|PDHX|ENO1|DHTKD1|NNT|PRDX5|NDUFA9|GPD1L|GPD1|DCXR|PDHB|GPD2|PKM|TALDO1|MDH2|IDH2|PKLR|PFKM|VCP|PNP|OGDHL|ALDOB|PDHA1|BPGM|ALDOA|QPRT|IDH1|TKT|GPI|GALK1|FMO1</td><td>8.02E-19</td><td>GO.0006733</td><td>1.63018995</td></tr>
<tr><td>5233</td><td>237</td><td>GO Process</td><td>localization</td><td>5.7E-17</td><td>RALA|CA12|VCL|MYH9|ATP6V1D|PYGB|F9|PSMD7|CRYM|CTSH|EHD4|ECH1|PLIN3|CDC37|OGDH|FIS1|SFXN3|PPIF|SLC25A3|LTF|EHHADH|ATP6V1B1|CTSD|APOA1|TTR|TFG|GOT2|CANX|VIL1|HSPB1|PMPCB|RPS16|ACTN4|LAMA5|ATP6V1E1|LGALS3|SYNJ2BP|ARL8B|TINAG|GGH|RAB21|LYZ|HEXB|PSMD11|CDH1|RAB11A|DNAJA3|SLC4A1|COTL1|SLC9A3R1|RAB2A|PACSIN2|NAPA|EPCAM|LRP2|GRB14|RAB17|PSMD3|ATP6V0A1|NNT|ADD1|F13A1|SCARB2|RAB7A|AMBP|ENPEP|VDAC1|CPT1A|NDUFA9|SORD|SLC27A2|RHCG|IQGAP1|AFG3L2|TINAGL1|PSMA5|ARL8A|ATP6V1A|FTH1|ATP6V1B2|ERLIN2|ENDOD1|LAMTOR1|SLC25A4|UCHL1|ACAA2|CA2|ATP6V0D2|AGL|FABP1|APEH|SCIN|HSPA9|AMN|ANPEP|GUSB|SLC23A1|RPS9|TMED10|GAA|YWHAG|ATP6V0A4|ACO1|DPYSL2|RAB1B|AQP1|RAB6A|DAB2|TOLLIP|TLN1|SFXN1|CYC1|GLS|PIPOX|PKM|SLC7A8|RHOT2|DHRS4|EHD3|AP2A2|SLC5A2|COPB2|ATP6V0C|TMED9|SERPINA5|LAMP1|RAB11B|ACAA1|BSG|PARVA|CYB5A|LAMTOR4|EPB41L3|APOD|PTGES2|VAPA|CTSB|ANXA6|AGK|GM2A|UBE2D3|VCP|ATP6V1H|MSN|MYH10|DPP4|PNP|OPA1|CYB5R1|ATP1B1|TAGLN2|FLNA|SFXN2|MYO6|ABCD3|RPL5|RBP4|CPT2|SCP2|SLC25A5|APOOL|GSN|RAB14|ATP6V1G1|CLIC4|HMGCL|HSPG2|CLIC1|TBC1D4|CUBN|SLC3A2|CKAP4|APRT|TXNDC5|AKR1C3|SLC25A6|ASAH1|CD9|KRT18|NME2|CNP|NIT2|ACTN1|PRKCD|PSAP|ALDOA|CD59|UMOD|MGST1|AMPD3|ILK|ATP5A1|PDIA6|TST|UBA52|SLC25A12|SCRN1|IDH1|SLC4A4|GDI1|PDZK1|ANXA11|RHOA|DDC|TMED4|GPI|GPX1|LAMP2|TOMM40|AOC1|TOM1|SERPINA1|MME|PPIA|FLNB|TMEM33|ACSL1|NDUFA13|WDR1|KIF13B|EPHX2|CAPN1|PHB2|ATP1A1|RAN|SLC25A10|RPL18|MGAM|CD63|TWF1|NPC2|SLC43A2|PHB|VPS29|PSMC3|PLCG2|HAO2</td><td>9.49E-19</td><td>GO.0051179</td><td>1.6242604</td></tr>
<tr><td>1370</td><td>99</td><td>GO Process</td><td>organonitrogen compound biosynthetic process</td><td>1.1E-16</td><td>DCN|MTHFD1|AHCY|OGDH|PDHX|AGXT2|ENO1|APOA1|GOT2|RPS16|BHMT2|GSTM3|NARS|DHTKD1|BBOX1|ATP6V0A1|BHMT|GLUD1|DLAT|QDPR|APEH|HPRT1|RPS9|ASL|PDHB|ATP6V0A4|AQP1|GBAS|TSFM|ACSS1|CYC1|GLS|SHMT1|UGDH|CHDH|PKM|BCAT2|CNDP2|IDH2|SHMT2|GSTA1|PKLR|PPA2|VAPA|PFKM|ALDH3A2|ALDH9A1|WARS|AGK|VCP|MRPL37|MRPL24|PNP|MRPL21|IBA57|MGST3|PHGDH|GSTO1|RPL5|GOT1|ACADM|CMPK1|AKR1A1|ASS1|PRPS1|OGDHL|HSPG2|ALDOB|VARS|AGMAT|PSAT1|APRT|PDHA1|FECH|BPGM|NME2|ALDOA|QPRT|MGST1|AMPD3|ABAT|GATM|HAGH|GGT5|ATP5A1|GGT1|ALDH7A1|UBA52|DDC|GPI|LARS2|MRPS22|GFM1|GSTA2|ACSL1|MMAB|RPL18|PAH|GALK1</td><td>1.87E-18</td><td>GO.1901566</td><td>1.5954677</td></tr>
<tr><td>168</td><td>20</td><td>KEGG Pathways</td><td>Alzheimer's disease</td><td>9.9E-07</td><td>HSD17B10|NDUFB4|NDUFA2|NDUFA10|NDUFA9|NDUFB9|CAPN2|NDUFB8|CYCS|UQCRH|CYC1|NDUFV1|NDUFA12|UQCR10|NDUFS2|ATP5A1|NDUFS1|MME|NDUFA13|CAPN1</td><td>1.26E-07</td><td>hsa05010</td><td>0.60052431</td></tr>
<tr><td>154</td><td>33</td><td>GO Process</td><td>hexose metabolic process</td><td>1.9E-16</td><td>PCK2|ENO1|GOT2|KHK|CPT1A|SORD|DLAT|GPD1|DCXR|GAA|PDHB|GPD2|PCK1|PKM|MDH2|PKLR|APOD|PFKM|GOT1|RBP4|AKR1A1|ALDOB|PDHA1|BPGM|PC|DAK|ALDOA|SLC25A12|GPI|FBP1|SLC25A10|GALK1|GALE</td><td>3.29E-18</td><td>GO.0019318</td><td>1.57121983</td></tr>
<tr><td>113</td><td>29</td><td>GO Process</td><td>glucose metabolic process</td><td>3.5E-16</td><td>PCK2|ENO1|GOT2|CPT1A|SORD|DLAT|GPD1|DCXR|GAA|PDHB|GPD2|PCK1|PKM|MDH2|PKLR|APOD|PFKM|GOT1|RBP4|AKR1A1|ALDOB|PDHA1|BPGM|PC|ALDOA|SLC25A12|GPI|FBP1|SLC25A10</td><td>5.97E-18</td><td>GO.0006006</td><td>1.54584208</td></tr>
<tr><td>104</td><td>28</td><td>GO Process</td><td>carbohydrate catabolic process</td><td>4.5E-16</td><td>PYGB|OGDH|ENO1|KHK|HEXB|DHTKD1|SORD|ABHD10|AGL|CRYL1|DCXR|GAA|GPD2|PKM|PKLR|PFKM|GM2A|AKR1A1|OGDHL|ALDOB|BPGM|DAK|ALDOA|GK|GPI|MGAM|GALK1|GALE</td><td>7.77E-18</td><td>GO.0016052</td><td>1.53496925</td></tr>
</table>

294	43	GO Process	fatty acid metabolic process	4.7E-16	ECH1|EHHADH|ACADL|ACADS|MECR|SCARB2|ACAD11|CPT1A|SLC27A2|ACAA2|CBR1|CRYL1|ECI1|CYP4A11|ACSS1|CES2|ACSM2B|ACAA1|GSTA1|PTGES2|ALDH3A2|ECHS1|ACADSB|ABCD3|ACADM|CPT2|SCP2|ECHDC2|AUH|AKR1C3|GGT5|GGT1|PTGR1|ACSF2|GPX1|ACSL1|ETFDH|EPHX2|ECHDC1|PTGR2|ACSM2A|HADH|HAO2	8.28E-18	GO.0006631	1.5326058
57	23	GO Function	NAD binding	6.7E-16	AHCY|IDH3G|ALDH2|NNT|SORD|GLUD1|QDPR|GPD1L|CRYL1|IDH3A|GPD1|GRHPR|HSD11B2|UGDH|NDUFV1|IDH2|NDUFS2|PHGDH|ALDH1B1|IDH3B|IDH1|ME3|HADH	4.73E-18	GO.0051287	1.51739252
40	20	GO Process	tricarboxylic acid metabolic process	1.5E-15	ACO2|IDH3G|OGDH|DHTKD1|NNT|GLUD1|DLAT|IDH3A|PDHB|ACO1|MDH2|IDH2|DLST|CS|ASS1|OGDHL|PDHA1|IDH3B|IDH1|ME3	2.73E-17	GO.0072350	1.48124793
1024	81	GO Process	cell activation	1.9E-15	GNA11|VCL|MYH9|ATP6V1D|PYGB|PSMD7|CTSH|LTF|CTSD|TTR|LGALS3|GGH|LYZ|HEXB|PSMD11|DNAJA3|COTL1|PRDX1|PSMD3|ATP6V0A1|RAB7A|SLC27A2|IQGAP1|PSMA5|ARL8A|FTH1|LAMTOR1|AGL|APEH|HPRT1|ANPEP|GUSB|GAA|RAB6A|TOLLIP|PKM|AP2A2|ATP6V0C|LAMP1|ACAA1|DHRS2|PTGES2|VAPA|CTSB|GM2A|VCP|MSN|DPP4|PNP|FLNA|GSN|RAB14|CKAP4|APRT|TXNDC5|ASAH1|CD9|NME2|NIT2|PRKCD|PSAP|ALDOA|CD59|MGST1|AMPD3|IDH1|RHOA|GPI|LAMP2|AOC1|TOM1|SERPINA1|MME|PPIA|KIF13B|CAPN1|MGAM|CD63|NPC2|PSMC3|PLCG2	3.36E-17	GO.0001775	1.47281584
2097	126	GO Function	nucleotide binding	3E-15	RALA|GNA11|MYH9|MTHFD1|PCK2|AHCY|IDH3G|CRYM|EHD4|ACADL|ATP6V1B1|ACADS|ARL8B|NARS|TINAG|KHK|RAB21|ALDH2|RAB11A|DNAJA3|RAB2A|RAB17|NNT|ACAD11|RAB7A|SORD|SLC27A2|AFG3L2|ARL8A|ATP6V1A|ATP6V1B2|GLUD1|QDPR|GPD1L|HSPA9|CRYL1|HPRT1|CKB|IDH3A|GPD1|PRODH2|HINT1|RAB1B|RAB6A|GRHPR|HSD11B2|ACSS1|UGDH|PCK1|CHDH|PKM|RHOT2|NDUFV1|EHD3|ACSM2B|IDH2|RAB11B|PKLR|PFKM|ANXA6|WARS|AGK|UBE2D3|VCP|MYH10|LONP1|OPA1|KIF21A|NDUFS2|ACADSB|PHGDH|MYO6|ABCD3|ACADM|SCP2|CMPK1|ASS1|PRPS1|RAB14|HMGCL|VARS|ALDH1B1|MAOB|APRT|NQO2|IDH3B|PRKAR1A|NME2|CNP|PC|PRKCD|DAK|AK4|MYH11|ILK|ATP5A1|SEPT7|TXNRD2|IDH1|CRYZ|RHOA|GK|ACSF2|CKMT2|CKMT1A|FBP1|LARS2|GFM1|ACSL1|NDUFA13|ETFDH|KIF13B|ME3|ATP1A1|MMAB|RAN|TWF1|ACSS3|ALDH6A1|ACSM2A|GALK1|HADH|DDX6|PSMC3|FMO1|HAO2	2.35E-17	GO.0000166	1.45287083
323	43	GO Component	secretory granule lumen	3.8E-15	VCL|PYGB|PSMD7|CTSH|LTF|CTSD|APOA1|TTR|ACTN4|GGH|LYZ|HEXB|PSMD11|COTL1|PSMD3|F13A1|PSMA5|AGL|GUSB|TOLLIP|PKM|ACAA1|PTGES2|GM2A|VCP|PNP|APOOL|GSN|APRT|TXNDC5|NME2|NIT2|ACTN1|PRKCD|ALDOA|AMPD3|IDH1|GPI|AOC1|SERPINA1|PPIA|NPC2|PSMC3	1.8E-16	GO.0034774	1.44225082
48	8	KEGG Pathways	Amino sugar and nucleotide sugar metabolism	0.00047	NANS|GNPNAT1|HEXB|UGDH|CYB5R1|GPI|GALK1|GALE	0.0001	hsa00520	0.33279021
94	9	KEGG Pathways	Amoebiasis	0.0064	GNA11|VCL|HSPB1|ACTN4|LAMA5|RAB7A|COL4A2|COL4A1|ACTN1	0.0016	hsa05146	0.219382
32	18	GO Process	tricarboxylic acid cycle	1.2E-14	ACO2|IDH3G|OGDH|DHTKD1|NNT|DLAT|IDH3A|PDHB|ACO1|MDH2|IDH2|DLST|CS|OGDHL|PDHA1|IDH3B|IDH1|ME3	2.22E-16	GO.0006099	1.39136402
55	21	GO Process	monosaccharide biosynthetic process	1.8E-14	PCK2|ENO1|GOT2|SORD|GPD1|GPD2|PCK1|TALDO1|MDH2|GOT1|RBP4|AKR1A1|ALDOB|BPGM|PC|ALDOA|SLC25A12|TKT|GPI|FBP1|SLC25A10	3.32E-16	GO.0046364	1.37423214
64	22	GO Process	cellular aldehyde metabolic process	2.1E-14	AGXT2|AKR7A2|GOT2|KHK|ALDH8A1|PRODH2|GRHPR|TALDO1|IDH2|ALDH3A2|ALDH9A1|AKR7A3|HOGA1|AKR1A1|ALDOB|ALDH4A1|AKR1C3|DAK|HAGH|ALDH7A1|IDH1|TKT	3.93E-16	GO.0006081	1.36736641
732	65	GO Process	cellular amide metabolic process	2.2E-14	MTHFD1|CTSH|OGDH|PDHX|ACOT13|RPS16|GSTM3|NARS|GGH|HEXB|ENPEP|NAT8|DLAT|FTCD|RNPEP|APEH|TPP1|ANPEP|RPS9|ASL|PDHB|TSFM|ACSS1|PIPOX|SHMT1|CNDP2|ACSM2B|SHMT2|DLST|GSTA1|PPA2|WARS|AGK|GM2A|MRPL37|MRPL24|MRPL21|GSTO1|RPL5|ASS1|HMGCL|VARS|ACOT9|PDHA1|ASAH1|DPEP1|PC|NIT2|HAGH|GGT5|GGT1|UBA52|IDH1|ACSF2|GPX1|LAMP2|LARS2|MRPS22|MME|GFM1|GSTA2|ACSL1|RPL18|ACSM2A|GLYAT	4.04E-16	GO.0043603	1.36655462

56	21	GO Process	fatty acid beta-oxidation	2.4E-14	ECH1|EHHADH|ACADL|ACADS|MECR|ACAD11|CPT1A|SLC27A2|ACAA2|ECI1|ACAA1|ECHS1|ABCD3|ACADM|CPT2|SCP2|ECHDC2|AUH|ETFDH|ECHDC1|HADH	4.45E-16	GO.0006635	1.36289321
84	24	GO Process	fatty acid catabolic process	3E-14	ECH1|EHHADH|ACADL|ACADS|MECR|ACAD11|CPT1A|SLC27A2|ACAA2|ECI1|CYP4A11|ACAA1|ALDH3A2|ECHS1|ABCD3|ACADM|CPT2|SCP2|ECHDC2|AUH|ETFDH|ECHDC1|HADH|HAO2	5.8E-16	GO.0009062	1.35185574
927	74	GO Process	immune effector process	3.1E-14	VCL|ATP6V1D|PYGB|PSMD7|CTSH|LTF|CTSD|TTR|LGALS3|GGH|LYZ|HEXB|PSMD11|COTL1|PRDX1|PSMD3|ATP6V0A1|RAB7A|SLC27A2|IQGAP1|PSMA5|ARL8A|FTH1|LAMTOR1|AGL|APEH|HPRT1|ANPEP|GUSB|GAA|RAB6A|TOLLIP|PKM|AP2A2|ATP6V0C|LAMP1|ACAA1|PTGES2|VAPA|CTSB|GM2A|VCP|PNP|GSN|RAB14|CKAP4|APRT|TXNDC5|ASAH1|NME2|NIT2|PRKCD|PSAP|ALDOA|CD59|MGST1|AMPD3|IDH1|RHOA|GPI|LAMP2|AOC1|TOM1|SERPINA1|CFB|MME|PPIA|WDR1|CAPN1|MGAM|CD63|NPC2|PSMC3|PLCG2	6.04E-16	GO.0002252	1.35044557
75	23	GO Process	fatty acid oxidation	3.4E-14	ECH1|EHHADH|ACADL|ACADS|MECR|ACAD11|CPT1A|SLC27A2|ACAA2|ECI1|ACAA1|ALDH3A2|ECHS1|ABCD3|ACADM|CPT2|SCP2|ECHDC2|AUH|ETFDH|ECHDC1|HADH|HAO2	6.56E-16	GO.0019395	1.34736607
66	22	GO Process	pyruvate metabolic process	3.4E-14	OGDH|PDHX|ENO1|DHTKD1|DLAT|PDHB|PCK1|PKM|BSG|PKLR|PFKM|HOGA1|OGDHL|ALDOB|PDHA1|BPGM|PC|ALDOA|HAGH|GPI|ME3|GALK1	6.68E-16	GO.0006090	1.34698003
120	9	KEGG Pathways	AMPK signaling pathway	0.0246	PCK2|RAB2A|CPT1A|PCK1|RAB11B|PFKM|RAB14|ELAVL1|FBP1	0.0071	hsa04152	0.16090649
61	9	KEGG Pathways	Arachidonic acid metabolism	0.00041	CBR1|CYP4A11|PTGES2|AKR1C3|GPX3|GGT5|GGT1|GPX1|EPHX2	8.73E-05	hsa00590	0.33872161
36	18	GO Process	citrate metabolic process	5.4E-14	ACO2|IDH3G|OGDH|DHTKD1|NNT|DLAT|IDH3A|PDHB|ACO1|MDH2|IDH2|DLST|CS|OGDHL|PDHA1|IDH3B|IDH1|ME3	1.08E-15	GO.0006101	1.32700257
125	27	GO Component	ficolin-1-rich granule lumen	6.4E-14	VCL|PSMD7|CTSH|CTSD|PSMD11|COTL1|PSMD3|PSMA5|FTH1|AGL|APEH|GUSB|PKM|CTSB|VCP|PNP|GSN|ASAH1|NME2|ALDOA|AMPD3|IDH1|GPI|SERPINA1|PPIA|CAPN1|PSMC3	3.34E-15	GO.1904813	1.31944991
48	18	KEGG Pathways	Arginine and proline metabolism	1.3E-12	GOT2|ALDH2|CKB|PRODH2|CNDP2|ALDH3A2|ALDH9A1|GOT1|HOGA1|ALDH4A1|AGMAT|ALDH1B1|MAOB|GATM|ALDH7A1|CKMT2|CKMT1A|AOC1	5.71E-14	hsa00330	1.18860566
2448	135	GO Function	hydrolase activity	1E-13	RALA|PSMA4|GNA11|MYH9|ATP6V1D|MTHFD1|AHCY|APMAP|F9|CTSH|ABHD11|ACOT13|DNPH1|LTF|FAHD2A|ATP6V1B1|AKR7A2|CTSD|GDA|HDHD3|PFN2|PLBD1|PEPD|CAPNS1|PMPCB|ATP6V1E1|NAPSA|HRSP12|ARL8B|TINAG|GGH|RAB21|LYZ|HEXB|RAB11A|RAB2A|RAB17|ATP6V0A1|RAB7A|ENPEP|REXO2|AFG3L2|TINAGL1|PSMA5|ARL8A|ABHD10|ATP6V1A|ATP6V1B2|DPYS|ENDOD1|DDAH1|UCHL1|CA2|ATP6V0D2|PSMB4|AGL|CAPN2|RNPEP|APEH|CMBL|TPP1|ANPEP|GUSB|HINT1|GNB2|GAA|ATP6V0A4|DPYSL2|RAB1B|RAB6A|AHCYL2|GLS|CES2|RHOT2|CNDP2|ATP6V0C|C11orf54|RAB11B|PPA2|CTSB|SND1|GM2A|VCP|ATP6V1H|HIBCH|MYH10|DPP4|LONP1|OPA1|KIF21A|ATP1B1|LHPP|MYO6|ABCD3|XPNPEP2|RAB14|ATP6V1G1|PSMB9|PSMB8|VARS|AGMAT|ACOT9|BPHL|ASAH1|SACM1L|DPEP1|BPGM|CNP|NIT2|PSAP|MYH11|AMPD3|HAGH|GGT5|ATP5A1|GGT1|ACY1|SCRN1|RHOA|FBP1|LARS2|CFB|MME|GFM1|ABHD14B|KIF13B|EPHX2|CAPN1|ATP1A1|RAN|MGAM|DDX6|EPHX1|PSMC3|PLCG2	9.73E-16	GO.0016787	1.3
71	22	GO Process	aerobic respiration	1.2E-13	ACO2|IDH3G|OGDH|PMPCB|DHTKD1|NNT|DLAT|IDH3A|PDHB|CYCS|ACO1|UQCRH|MDH2|IDH2|UQCR10|DLST|CS|OGDHL|PDHA1|IDH3B|IDH1|ME3	2.36E-15	GO.0009060	1.2935542
582	55	GO Component	lysosome	1.6E-13	DCN|EPDR1|PYGB|CTSH|CTSD|TTR|PLBD1|NAPSA|ARL8B|GGH|LYZ|HEXB|LRP2|ATP6V0A1|SCARB2|RAB7A|ARL8A|FTH1|LAMTOR1|ATP6V0D2|CAPN2|TPP1|GUSB|GAA|DAB2|TOLLIP|AP2A2|ATP6V0C|LAMP1|LAMTOR4|PTGES2|VAPA|CTSB|ANXA6|GM2A|VCP|MYO6|GOT1|ASS1|RAB14|HSPG2|CUBN|CKAP4|TXNDC5|ASAH1|PRKCD|PSAP|MGST1|IFI30|ANXA11|LAMP2|TOM1|CAPN1|CD63|NPC2	8.41E-15	GO.0005764	1.28068754

682	60	GO Component	vacuole	1.6E-13	DCN|EPDR1|PYGB|CTSH|ATP6V1B1|CTSD|TTR|PLBD1|NAPSA|ARL8B|GGH|LYZ|HEXB|NAPA|LRP2|ATP6V0A1|SCARB2|RAB7A|ARL8A|FTH1|LAMTOR1|ATP6V0D2|CAPN2|TPP1|GUSB|GAA|ATP6V0A4|DAB2|TOLLIP|AP2A2|ATP6V0C|LAMP1|LAMTOR4|PTGES2|VAPA|CTSB|ANXA6|GM2A|VCP|ATP6V1H|MYO6|GOT1|ASS1|RAB14|ATP6V1G1|HSPG2|CUBN|CKAP4|TXNDC5|ASAH1|PRKCD|PSAP|MGST1|IFI30|ANXA11|LAMP2|TOM1|CAPN1|CD63|NPC2	8.97E-15	GO.0005773	1.28013429
218	34	GO Process	cofactor biosynthetic process	2.3E-13	MTHFD1|OGDH|PDHX|ENO1|DHTKD1|NDUFA9|DLAT|QDPR|PDHB|ACSS1|PKM|CNDP2|IDH2|PKLR|PFKM|PNP|IBA57|AKR1A1|OGDHL|ALDOB|PSAT1|MAOB|PDHA1|FECH|BPGM|ALDOA|QPRT|HAGH|GGT5|GGT1|GPI|ACSL1|MMAB|GALK1	4.69E-15	GO.0051188	1.26401645
175	31	GO Function	lyase activity	3.4E-13	CA12|ACO2|PCK2|APMAP|EHHADH|ENO1|BCKDHA|CA2|FTCD|ECI1|ASL|ACO1|SHMT1|PCK1|SHMT2|PTGES2|MGST3|ECHS1|GOT1|HOGA1|ECHDC2|HMGCL|ALDOB|AUH|GLDC|FECH|DAK|ALDOA|DDC|ECHDC1|ME3	3.62E-15	GO.0016829	1.24659739
167	30	GO Process	cellular lipid catabolic process	3.5E-13	ECH1|EHHADH|ACADL|APOA1|ACADS|HEXB|MECR|ACAD11|CPT1A|SLC27A2|ACAA2|FABP1|ECI1|CYP4A11|ACAA1|ALDH3A2|GM2A|ECHS1|ABCD3|ACADM|CPT2|SCP2|ECHDC2|AUH|AKR1C3|ETFDH|ECHDC1|HADH|PLCG2|HAO2	7.33E-15	GO.0044242	1.24509967
2696	142	GO Function	anion binding	4.4E-13	RALA|GNA11|MYH9|MTHFD1|PCK2|PYGB|IDH3G|EHD4|OGDH|AGXT2|LTF|ACADL|ATP6V1B1|APOA1|PFN2|ACADS|GOT2|VIL1|HRSP12|ARL8B|GSTM3|NARS|KHK|RAB21|RAB11A|DNAJA3|RAB2A|DHTKD1|PACSIN2|RAB17|ACAD11|RAB7A|SLC27A2|IQGAP1|AFG3L2|ARL8A|ATP6V1A|ATP6V1B2|DPYS|GLUD1|QDPR|DDAH1|FTCD|FABP1|SCIN|HSPA9|CRYL1|CKB|PRODH2|RAB1B|RAB6A|GRHPR|TLN1|ACSS1|SHMT1|PCK1|CHDH|PKM|RHOT2|NDUFV1|EHD3|ACSM2B|SERPINA5|RAB11B|SHMT2|PKLR|PTGES2|PFKM|ANXA6|UGT1A9|WARS|AGK|UBE2D3|VCP|MYH10|LONP1|PNP|OPA1|KIF21A|MARC2|ACADSB|MYO6|ABCD3|GOT1|ACADM|SCP2|CMPK1|ASS1|PRPS1|RAB14|OGDHL|HMGCL|VARS|MAOB|APRT|NQO2|GLDC|GPNMB|PRKAR1A|DPEP1|NME2|PC|PRKCD|DAK|PSAP|AK4|MGST1|MYH11|ABAT|ILK|ATP5A1|SEPT7|TXNRD2|ANXA11|CRYZ|RHOA|GK|ACSF2|DDC|CKMT2|CKMT1A|FBP1|LARS2|AOC1|GFM1|ACSL1|NDUFA13|ETFDH|KIF13B|ATP1A1|MMAB|RAN|TWF1|ACSS3|ALDH6A1|ACSM2A|GALK1|HADH|DDX6|PSMC3|FMO1|HAO2	4.98E-15	GO.0043168	1.23615107
127	26	GO Component	peroxisome	5.6E-13	CRYM|ECH1|FIS1|EHHADH|HRSP12|ACAD11|PRDX5|SLC27A2|FABP1|GRHPR|PIPOX|DHRS4|IDH2|ACAA1|ALDH3A2|MARC2|ABCD3|SCP2|HMGCL|MGST1|IDH1|TKT|AOC1|ACSL1|EPHX2|HAO2	3.28E-14	GO.0005777	1.22510371

4153	190	GO Process	response to chemical	6.5E-13	RALA|DCN|GNA11|GNPNAT1|ATP6V1D|AHCY|TXNL1|CTSH|EHD4|HNRNPL|FIS1|PPIF|ATP6V1B1|AKR7A2|APOA1|GOT2|CANX|VIL1|HSPB1|LAMA5|ATP6V1E1|LGALS3|HRSP12|GSTM3|GGH|KHK|CDH1|DNAJA3|PRDX1|LRP2|ATP6V0A1|NNT|ADD1|F13A1|CALB1|PRDX5|CPT1A|SORD|IQGAP1|NAT8|ATP6V1A|ATP6V1B2|ERLIN2|LAMTOR1|QDPR|SLC25A4|ACAA2|CA2|ATP6V0D2|AGL|CAPN2|FABP1|CMBL|HSPA9|HPRT1|AMN|TPP1|COL6A2|GPD1|SLC23A1|TMED10|YWHAG|CYCS|ATP6V0A4|ACO1|DPYSL2|AQP1|AOC3|HNRNPD|TOLLIP|TLN1|HSD11B2|CYC1|CES2|SHMT1|PCK1|PKM|SLC7A8|TALDO1|BCAT2|HNRNPM|ACSM2B|ATP6V0C|RAB11B|SHMT2|BSG|PARVA|GSTA1|PKLR|CYB5A|LAMTOR4|DHRS2|APOD|CTSB|UGT1A9|COL18A1|UBE2D3|VCP|ATP6V1H|MSN|MYH10|COL4A2|LONP1|PNP|OPA1|COL6A1|AKR7A3|MARC2|MGST3|PHGDH|GSTO1|MYO6|ABCD3|GOT1|RBP4|SLC25A5|ASS1|GSN|RAB14|ATP6V1G1|CLIC4|HMGCL|PSMB8|ASPN|COL4A1|TBC1D4|CUBN|SLC3A2|MAOB|APRT|MT1M|BPHL|NQO2|AKR1C3|GLDC|SLC25A6|GPNMB|CD9|FECH|GPX3|KRT18|PRKAR1A|DPEP1|CNP|PRKCD|PSAP|AK4|MGST1|ABAT|ILK|MPST|GGT1|TXNRD2|ACY1|IFI30|PDIA6|PTGR1|UBA52|SLC25A12|IDH1|GDI1|PDZK1|HIGD1A|ANXA11|CRYZ|RHOA|DDC|GPX1|FBP1|AOC1|MME|FAM162A|PPIA|GSTA2|FLNB|ACSL1|NDUFA13|EPHX2|PHB2|ATP1A1|RAN|SLC25A10|KRT8|HADH|PHB|EPHX1|PSMC3|FMO1|PLCG2|GLYAT	1.35E-14	GO.0042221	1.21890957
20	7	KEGG Pathways	Arginine biosynthesis	2.7E-05	GOT2|GLUD1|ASL|GLS|GOT1|ASS1|ACY1	4.67E-06	hsa00220	0.45654311
27	9	KEGG Pathways	Ascorbate and aldarate metabolism	1.9E-06	ALDH2|UGT2B7|UGDH|UGT2B17|ALDH3A2|UGT1A9|ALDH9A1|ALDH1B1|ALDH7A1	2.79E-07	hsa00053	0.57121983
206	32	GO Process	purine-containing compound biosynthetic process	1.6E-12	MTHFD1|OGDH|PDHX|ENO1|DHTKD1|ATP6V0A1|DLAT|HPRT1|PDHB|ATP6V0A4|AQP1|GBAS|ACSS1|CYC1|SHMT1|PKM|PKLR|PFKM|PNP|PRPS1|OGDHL|ALDOB|APRT|PDHA1|BPGM|NME2|ALDOA|AMPD3|ATP5A1|GPI|ACSL1|GALK1	3.36E-14	GO.0072522	1.17986029
1192	82	GO Process	lipid metabolic process	1.6E-12	HSD17B10|ECH1|EHHADH|ACADL|APOA1|TTR|PLBD1|ACADS|HEXB|MECR|LRP2|SCARB2|ACAD11|RAB7A|ALDH8A1|CPT1A|SLC27A2|ERLIN2|GPD1L|ACAA2|CBR1|FABP1|CRYL1|TPP1|GPD1|ECI1|UGT2B7|CYP4A11|HSD11B2|ACSS1|CES2|PCK1|UGT2B17|DHRS4|ACSM2B|ACAA1|GSTA1|DHRS2|APOD|PTGES2|VAPA|ALDH3A2|UGT1A9|AGK|GM2A|CYB5R1|MGST3|ECHS1|ACADSB|ABCD3|ACADM|RBP4|CPT2|SCP2|ECHDC2|RAB14|HMGCL|HSPG2|AUH|CUBN|AKR1C3|ASAH1|SACM1L|PC|PSAP|GGT5|ATP5A1|GGT1|PTGR1|GK|ACSF2|GPX1|ACSL1|ETFDH|EPHX2|ECHDC1|NPC2|PTGR2|ACSM2A|HADH|PLCG2|HAO2	3.41E-14	GO.0006629	1.179588
103	24	GO Function	electron transfer activity	2.3E-12	AKR7A2|DMGDH|ALDH2|QDPR|CYCS|UQCRH|CYC1|UGDH|NDUFA12|UQCR10|CYB5A|PTGES2|AKR7A3|NDUFS2|PHGDH|AKR1A1|ALDH4A1|MAOB|NQO2|IDH3B|GLDC|TXNRD2|NDUFS1|ETFDH	2.78E-14	GO.0009055	1.16478175
2086	117	GO Process	phosphorus metabolic process	2.3E-12	NDUFB4|NANS|GNPNAT1|MTHFD1|CTSH|CDC37|OGDH|PDHX|ACOT13|DNPH1|ATP6V1B1|ENO1|APOA1|GDA|PLBD1|PMPCB|NDUFA2|NDUFA10|KHK|HEXB|DHTKD1|ATP6V0A1|NNT|PRDX5|REXO2|NDUFA9|SORD|ATP6V1A|ATP6V1B2|ERLIN2|NDUFB9|DLAT|GPD1L|CRYL1|HPRT1|NDUFB8|CKB|GPD1|DCXR|HINT1|PDHB|CYCS|ATP6V0A4|GPD2|UQCRH|AQP1|GBAS|TOLLIP|ACSS1|CYC1|PIPOX|SHMT1|UGDH|PKM|TALDO1|NDUFV1|MDH2|ACSM2B|NDUFA12|IDH2|UQCR10|DLST|PKLR|PPA2|PFKM|AGK|VCP|PNP|OPA1|ATP1B1|NDUFS2|LHPP|SCP2|CMPK1|AKR1A1|PRPS1|RAB14|OGDHL|HMGCL|ALDOB|APRT|ACOT9|PDHA1|SACM1L|BPGM|NME2|CNP|PRKCD|DAK|ALDOA|AK4|QPRT|AMPD3|ILK|ATP5A1|UBA52|IDH1|TKT|NDUFS1|RHOA|GK|ACSF2|CKMT2|GPI|CKMT1A|GPX1|FBP1|ABHD14B|ACSL1|EPHX2|ATP1A1|RAN|ACSM2A|GALK1|FMO1|PLCG2|GLYAT	4.93E-14	GO.0006793	1.16401645

108	24	GO Process	xenobiotic metabolic process	3.1E-12	AKR7A2|GSTM3|CMBL|AOC3|CES2|ACSM2B|UGT1A9|AKR7A3|MGST3|PHGDH|GSTO1|BPHL|NQO2|DPEP1|MGST1|GGT1|ACY1|CRYZ|AOC1|ACSL1|EPHX2|EPHX1|FMO1|GLYAT	6.82E-14	GO.0006805	1.15030704
97	23	GO Process	dicarboxylic acid metabolic process	3.2E-12	MTHFD1|OGDH|GOT2|GLUD1|FTCD|PRODH2|GRHPR|GLS|SHMT1|PCK1|MDH2|IDH2|SHMT2|DLST|GOT1|HOGA1|ASS1|ALDH4A1|NIT2|QPRT|GGT1|IDH1|ME3	7.01E-14	GO.0043648	1.149485
49	18	GO Process	hexose biosynthetic process	3.5E-12	PCK2|ENO1|GOT2|SORD|GPD1|GPD2|PCK1|MDH2|GOT1|RBP4|ALDOB|BPGM|PC|ALDOA|SLC25A12|GPI|FBP1|SLC25A10	7.61E-14	GO.0019319	1.14621809
293	37	GO Process	nucleoside phosphate biosynthetic process	4.8E-12	MTHFD1|OGDH|PDHX|ENO1|DHTKD1|ATP6V0A1|DLAT|HPRT1|PDHB|ATP6V0A4|AQP1|GBAS|ACSS1|CYC1|SHMT1|PKM|IDH2|PKLR|PFKM|PNP|LHPP|CMPK1|PRPS1|OGDHL|ALDOB|APRT|PDHA1|BPGM|NME2|ALDOA|AK4|QPRT|AMPD3|ATP5A1|GPI|ACSL1|GALK1	1.07E-13	GO.1901293	1.13178549
2040	114	GO Process	organic substance transport	6.5E-12	CA12|MYH9|ATP6V1D|ECH1|CDC37|FIS1|EHHADH|ATP6V1B1|APOA1|GOT2|CANX|VIL1|HSPB1|PMPCB|RPS16|ACTN4|ATP6V1E1|SYNJ2BP|RAB21|RAB11A|SLC4A1|SLC9A3R1|RAB2A|NAPA|LRP2|RAB17|ATP6V0A1|SCARB2|RAB7A|CPT1A|SLC27A2|RHCG|ATP6V1A|ATP6V1B2|SLC25A4|CA2|ATP6V0D2|FABP1|HSPA9|AMN|SLC23A1|RPS9|TMED10|YWHAG|ATP6V0A4|RAB1B|AQP1|RAB6A|DAB2|GLS|PIPOX|SLC7A8|DHRS4|EHD3|AP2A2|SLC5A2|COPB2|ATP6V0C|TMED9|SERPINA5|RAB11B|ACAA1|BSG|APOD|AGK|GM2A|UBE2D3|VCP|ATP6V1H|PNP|CYB5R1|ATP1B1|MYO6|ABCD3|RPL5|RBP4|CPT2|SCP2|SLC25A5|RAB14|ATP6V1G1|HMGCL|TBC1D4|CUBN|SLC3A2|AKR1C3|SLC25A6|KRT18|PSAP|TST|UBA52|SLC25A12|IDH1|SLC4A4|GDI1|PDZK1|DDC|TMED4|LAMP2|TOMM40|TOM1|ACSL1|NDUFA13|KIF13B|EPHX2|PHB2|RAN|SLC25A10|RPL18|CD63|NPC2|SLC43A2|VPS29|HAO2	1.46E-13	GO.0071702	1.11890957
193	30	GO Process	purine nucleotide biosynthetic process	9.4E-12	MTHFD1|OGDH|PDHX|ENO1|DHTKD1|ATP6V0A1|DLAT|HPRT1|PDHB|ATP6V0A4|AQP1|GBAS|ACSS1|CYC1|PKM|PKLR|PFKM|PRPS1|OGDHL|ALDOB|APRT|PDHA1|BPGM|NME2|ALDOA|AMPD3|ATP5A1|GPI|ACSL1|GALK1	2.13E-13	GO.0006164	1.10273344
577	52	GO Process	organophosphate biosynthetic process	1.2E-11	MTHFD1|OGDH|PDHX|ENO1|APOA1|HEXB|DHTKD1|ATP6V0A1|SORD|DLAT|GPD1L|CRYL1|HPRT1|GPD1|DCXR|PDHB|ATP6V0A4|AQP1|GBAS|ACSS1|CYC1|SHMT1|PKM|IDH2|PKLR|PFKM|AGK|PNP|LHPP|SCP2|CMPK1|AKR1A1|PRPS1|RAB14|OGDHL|ALDOB|APRT|PDHA1|SACM1L|BPGM|NME2|ALDOA|AK4|QPRT|AMPD3|ATP5A1|TKT|GK|GPI|ACSL1|GALK1|PLCG2	2.66E-13	GO.0090407	1.0935542
2065	114	GO Process	phosphate-containing compound metabolic process	1.4E-11	NDUFB4|MTHFD1|CTSH|CDC37|OGDH|PDHX|ACOT13|DNPH1|ATP6V1B1|ENO1|APOA1|GDA|PLBD1|PMPCB|NDUFA2|NDUFA10|KHK|HEXB|DHTKD1|ATP6V0A1|NNT|PRDX5|REXO2|NDUFA9|SORD|ATP6V1A|ATP6V1B2|ERLIN2|NDUFB9|DLAT|GPD1L|CRYL1|HPRT1|NDUFB8|CKB|GPD1|DCXR|HINT1|PDHB|CYCS|ATP6V0A4|GPD2|UQCRH|AQP1|GBAS|TOLLIP|ACSS1|CYC1|PIPOX|SHMT1|PKM|TALDO1|NDUFV1|MDH2|ACSM2B|NDUFA12|IDH2|UQCR10|DLST|PKLR|PPA2|PFKM|AGK|VCP|PNP|OPA1|ATP1B1|NDUFS2|LHPP|SCP2|CMPK1|AKR1A1|PRPS1|RAB14|OGDHL|HMGCL|ALDOB|APRT|ACOT9|PDHA1|SACM1L|BPGM|NME2|CNP|PRKCD|DAK|ALDOA|AK4|QPRT|AMPD3|ILK|ATP5A1|UBA52|IDH1|TKT|NDUFS1|RHOA|GK|ACSF2|CKMT2|GPI|CKMT1A|GPX1|FBP1|ABHD14B|ACSL1|EPHX2|ATP1A1|RAN|ACSM2A|GALK1|FMO1|PLCG2|GLYAT	3.16E-13	GO.0006796	1.08632794
251	33	GO Component	mitochondrial protein complex	1.4E-11	HSD17B10|NDUFB4|PPIF|PMPCB|NDUFA2|NDUFA10|CHCHD3|VDAC1|NDUFA9|AFG3L2|BCKDHA|NDUFB9|DLAT|NDUFB8|UQCRH|CYC1|NDUFV1|NDUFA12|UQCR10|AGK|MRPL37|MRPL24|MRPL21|NDUFS2|APOOL|SLC25A6|ATP5A1|IMMT|NDUFS1|TOMM40|MRPS22|NDUFA13|ETFDH	8.79E-13	GO.0098798	1.0844664
46	17	GO Process	gluconeogenesis	1.5E-11	PCK2|ENO1|GOT2|GPD1|GPD2|PCK1|MDH2|GOT1|RBP4|ALDOB|BPGM|PC|ALDOA|SLC25A12|GPI|FBP1|SLC25A10	3.49E-13	GO.0006094	1.08239087

291	36	GO Process	nucleotide biosynthetic process	1.7E-11	MTHFD1|OGDH|PDHX|ENO1|DHTKD1|ATP6V0A1|DLAT|HPRT1|PDHB|ATP6V0A4|AQP1|GBAS|ACSS1|CYC1|SHMT1|PKM|IDH2|PKLR|PFKM|PNP|CMPK1|PRPS1|OGDHL|ALDOB|APRT|PDHA1|BPGM|NME2|ALDOA|AK4|QPRT|AMPD3|ATP5A1|GPI|ACSL1|GALK1	4.05E-13	GO.0009165	1.07644716
157	27	GO Process	cellular response to xenobiotic stimulus	1.8E-11	AKR7A2|GSTM3|CMBL|AQP1|AOC3|CES2|ACSM2B|UGT1A9|AKR7A3|MGST3|PHGDH|GSTO1|ASS1|BPHL|NQO2|FECH|DPEP1|MGST1|GGT1|ACY1|CRYZ|AOC1|ACSL1|EPHX2|EPHX1|FMO1|GLYAT	4.17E-13	GO.0071466	1.07544873
119	24	GO Process	nucleoside monophosphate biosynthetic process	1.8E-11	OGDH|ENO1|DHTKD1|ATP6V0A1|HPRT1|ATP6V0A4|GBAS|CYC1|SHMT1|PKM|PKLR|PFKM|LHPP|CMPK1|PRPS1|OGDHL|ALDOB|APRT|BPGM|ALDOA|AMPD3|ATP5A1|GPI|GALK1	4.27E-13	GO.0009124	1.0747147
1554	93	GO Component	whole membrane	1.9E-11	SYPL1|VCL|GNPNAT1|ATP6V1D|EHD4|PLIN3|FIS1|ATP6V1B1|CTSD|LGALS3|SYNJ2BP|ARL8B|RAB21|CDH1|RAB11A|SLC9A3R1|PACSIN2|NAPA|LRP2|GRB14|RAB17|ATP6V0A1|SCARB2|RAB7A|VDAC1|CPT1A|SLC27A2|IQGAP1|ARL8A|ERLIN2|LAMTOR1|ATP6V0D2|CAPN2|AMN|ANPEP|TMED10|GAA|ATP6V0A4|RAB1B|RAB6A|GBAS|DAB2|RHOT2|EHD3|AP2A2|COPB2|ATP6V0C|SERPINA5|LAMP1|RAB11B|BSG|CYB5A|LAMTOR4|VAPA|ALDH3A2|ANXA6|AGK|UBE2D3|ATP6V1H|DPP4|OPA1|MARC2|CYB5R1|ATP1B1|MYO6|SLC25A5|ASS1|RAB14|ATP6V1G1|CUBN|CKAP4|MAOB|BPHL|CD9|PRKAR1A|CNP|PSAP|CD59|MGST1|UBA52|PDZK1|RHOA|GK|LAMP2|TOMM40|TOM1|MME|ACSL1|PHB2|ATP1A1|MGAM|CD63|VPS29	1.18E-12	GO.0098805	1.07281584
202	30	GO Process	ribonucleotide biosynthetic process	2.5E-11	OGDH|PDHX|ENO1|DHTKD1|ATP6V0A1|DLAT|HPRT1|PDHB|ATP6V0A4|AQP1|GBAS|ACSS1|CYC1|PKM|PKLR|PFKM|CMPK1|PRPS1|OGDHL|ALDOB|APRT|PDHA1|BPGM|NME2|ALDOA|AMPD3|ATP5A1|GPI|ACSL1|GALK1	6.09E-13	GO.0009260	1.05968795
98	22	GO Process	purine ribonucleoside monophosphate biosynthetic process	2.6E-11	OGDH|ENO1|DHTKD1|ATP6V0A1|HPRT1|ATP6V0A4|GBAS|CYC1|PKM|PKLR|PFKM|LHPP|PRPS1|OGDHL|ALDOB|APRT|BPGM|ALDOA|AMPD3|ATP5A1|GPI|GALK1	6.37E-13	GO.0009168	1.05800443
110	23	GO Process	ribonucleoside monophosphate biosynthetic process	2.8E-11	OGDH|ENO1|DHTKD1|ATP6V0A1|HPRT1|ATP6V0A4|GBAS|CYC1|PKM|PKLR|PFKM|LHPP|CMPK1|PRPS1|OGDHL|ALDOB|APRT|BPGM|ALDOA|AMPD3|ATP5A1|GPI|GALK1	6.87E-13	GO.0009156	1.05543958
189	29	GO Process	purine ribonucleotide biosynthetic process	2.9E-11	OGDH|PDHX|ENO1|DHTKD1|ATP6V0A1|DLAT|HPRT1|PDHB|ATP6V0A4|AQP1|GBAS|ACSS1|CYC1|PKM|PKLR|PFKM|PRPS1|OGDHL|ALDOB|APRT|PDHA1|BPGM|NME2|ALDOA|AMPD3|ATP5A1|GPI|ACSL1|GALK1	7.2E-13	GO.0009152	1.05391022
77	20	GO Process	cellular amino acid biosynthetic process	2.9E-11	MTHFD1|AHCY|AGXT2|GOT2|BHMT2|BHMT|GLUD1|ASL|GLS|SHMT1|BCAT2|SHMT2|PHGDH|GOT1|ASS1|PSAT1|ABAT|GATM|GGT1|PAH	7.19E-13	GO.0008652	1.05391022
155	26	GO Component	azurophil granule	2.9E-11	PYGB|TTR|GGH|LYZ|HEXB|ARL8A|LAMTOR1|GUSB|GAA|TOLLIP|ATP6V0C|LAMP1|PTGES2|VAPA|GM2A|VCP|CKAP4|TXNDC5|PRKCD|PSAP|MGST1|ANXA11|LAMP2|TOM1|CD63|NPC2	1.89E-12	GO.0042582	1.05346171
425	43	GO Process	organic hydroxy compound metabolic process	4.2E-11	ACO2|IDH3G|CRYM|AKR7A2|APOA1|TTR|ALDH2|LRP2|SORD|SLC27A2|ERLIN2|QDPR|ACAA2|HPRT1|GPD1|ADH1B|GPD2|CYP4A11|ACSS1|PCK1|DHRS4|IDH2|ACAA1|ALDH3A2|CYB5R1|GOT1|RBP4|SCP2|PSAT1|ALDH1B1|CUBN|MAOB|AKR1C3|IDH3B|DAK|HAGH|IDH1|GK|DDC|PAH|NPC2|GALK1|PLCG2	1.06E-12	GO.1901615	1.03809067
60	18	GO Process	alpha-amino acid biosynthetic process	5.2E-11	MTHFD1|AHCY|AGXT2|GOT2|BHMT2|BHMT|GLUD1|ASL|GLS|SHMT1|BCAT2|SHMT2|PHGDH|GOT1|ASS1|PSAT1|GATM|GGT1	1.32E-12	GO.1901607	1.02873503

2672	134	GO Process	cellular response to chemical stimulus	5.2E-11	RALA|DCN|GNA11|GNPNAT1|ATP6V1D|TXNL1|CTSH|EHD4|FIS1|PPIF|ATP6V1B1|AKR7A2|CANX|VIL1|HSPB1|LAMA5|ATP6V1E1|LGALS3|GSTM3|CDH1|PRDX1|ATP6V0A1|NNT|ADD1|F13A1|CALB1|PRDX5|CPT1A|IQGAP1|ATP6V1A|ATP6V1B2|LAMTOR1|QDPR|ACAA2|CA2|ATP6V0D2|CAPN2|FABP1|CMBL|HSPA9|TPP1|GPD1|YWHAG|CYCS|ATP6V0A4|AQP1|AOC3|HNRNPD|TOLLIP|TLN1|CES2|SHMT1|PCK1|PKM|TALDO1|BCAT2|HNRNPM|ACSM2B|ATP6V0C|RAB11B|PARVA|GSTA1|PKLR|LAMTOR4|DHRS2|CTSB|UGT1A9|UBE2D3|VCP|ATP6V1H|MSN|COL4A2|LONP1|OPA1|COL6A1|AKR7A3|MGST3|PHGDH|GSTO1|GOT1|SLC25A5|ASS1|GSN|RAB14|ATP6V1G1|CLIC4|PSMB8|ASPN|COL4A1|TBC1D4|APRT|MT1M|BPHL|NQO2|AKR1C3|GLDC|FECH|GPX3|KRT18|PRKAR1A|DPEP1|PRKCD|PSAP|MGST1|ILK|GGT1|TXNRD2|ACY1|IFI30|PDIA6|UBA52|HIGD1A|CRYZ|RHOA|DDC|GPX1|FBP1|AOC1|MME|FAM162A|PPIA|GSTA2|FLNB|ACSL1|NDUFA13|EPHX2|PHB2|ATP1A1|RAN|KRT8|PHB|EPHX1|FMO1|GLYAT	1.34E-12	GO.0070887	1.02848326
31	14	KEGG Pathways	beta-Alanine metabolism	9.6E-11	EHHADH|ALDH2|DPYS|AOC3|CNDP2|ALDH3A2|ALDH9A1|HIBCH|ECHS1|ACADM|ALDH1B1|ABAT|ALDH7A1|ALDH6A1	4.98E-12	hsa00410	1.00186345
158	26	Reactome Pathways	The citric acid (TCA) cycle and respiratory electron transport	8.2E-11	NDUFB4|OGDH|PDHX|NDUFA2|NDUFA10|NNT|NDUFA9|ECSIT|NDUFB9|DLAT|IDH3A|PDHB|UQCRH|NDUFV1|MDH2|NDUFA12|IDH2|UQCR10|DLST|NDUFS2|PDHA1|IDH3B|NDUFS1|NDUFA13|ETFDH|ME3	2.78E-12	HSA-1428517	1.00861861
71	6	KEGG Pathways	Bile secretion	0.0499	CA2|AQP1|ATP1B1|SLC4A4|ATP1A1|EPHX1	0.0158	hsa04976	0.13018995
120	23	GO Process	carbohydrate biosynthetic process	1.3E-10	NANS|PCK2|ENO1|GOT2|SORD|AGL|GPD1|GPD2|PCK1|TALDO1|MDH2|GOT1|RBP4|AKR1A1|ALDOB|BPGM|PC|ALDOA|SLC25A12|TKT|GPI|FBP1|SLC25A10	3.31E-12	GO.0016051	0.98961963
1754	100	GO Function	identical protein binding	1.7E-10	MYH9|GNPNAT1|AHCY|PSMD7|CRYM|DNPH1|AGXT2|ENO1|APOA1|TTR|TFG|GOT2|VIL1|HSPB1|ACTN4|HRSP12|APCS|GSTM3|LYZ|HEXB|CDH1|SLC4A1|PRDX1|BBOX1|PACSIN2|GRB14|ADD1|AMBP|VDAC1|CPT1A|SORD|RHCG|GLUD1|DLAT|QDPR|GPD1L|HGD|APEH|CRYL1|HPRT1|GPD1|DCXR|YWHAG|ASL|DPYSL2|AQP1|AOC3|GRHPR|SHMT1|PKM|SHMT2|PFKM|ALDH3A2|ANXA6|UGT1A9|WARS|COL18A1|PBLD|VCP|DPP4|AKR7A3|LHPP|FLNA|MYO6|ABCD3|HOGA1|ACADM|ASS1|PRPS1|HMGCL|ALDOB|ALDH4A1|KCTD12|CUBN|MAOB|NQO2|GLDC|PC|ACTN1|PSAP|ALDOA|QPRT|MGST1|ABAT|MPST|SEPT7|ACY1|ELAVL1|SLC25A12|IDH1|TKT|SLC4A4|FBP1|AOC1|SERPINA1|VWA1|FLNB|EPHX2|PSMC3|GALE	2.19E-12	GO.0042802	0.97798919
101	21	GO Process	nucleotide catabolic process	2.9E-10	OGDH|DNPH1|ENO1|GDA|DHTKD1|HPRT1|HINT1|PKM|PKLR|PFKM|VCP|PNP|OGDHL|ALDOB|BPGM|CNP|ALDOA|AMPD3|GPI|GPX1|GALK1	7.62E-12	GO.0009166	0.9536107
946	66	GO Process	cellular lipid metabolic process	3.2E-10	ECH1|EHHADH|ACADL|APOA1|TTR|PLBD1|ACADS|HEXB|MECR|LRP2|SCARB2|ACAD11|ALDH8A1|CPT1A|SLC27A2|GPD1L|ACAA2|CBR1|FABP1|CRYL1|GPD1|ECI1|CYP4A11|ACSS1|CES2|PCK1|DHRS4|ACSM2B|ACAA1|GSTA1|PTGES2|VAPA|ALDH3A2|UGT1A9|AGK|GM2A|ECHS1|ACADSB|ABCD3|ACADM|RBP4|CPT2|SCP2|ECHDC2|RAB14|HSPG2|AUH|AKR1C3|ASAH1|SACM1L|PSAP|GGT5|GGT1|PTGR1|GK|ACSF2|GPX1|ACSL1|ETFDH|EPHX2|ECHDC1|PTGR2|ACSM2A|HADH|PLCG2|HAO2	8.34E-12	GO.0044255	0.95003129
441	42	GO Process	cellular nitrogen compound catabolic process	4.4E-10	AHCY|OGDH|DNPH1|ACADL|ENO1|GDA|RPS16|DMGDH|DHTKD1|AMBP|BHMT|DPYS|HNMT|FTCD|HPRT1|RPS9|HINT1|HNRNPD|PKM|PKLR|PFKM|SND1|VCP|PNP|RPL5|OGDHL|ALDOB|DPEP1|BPGM|CNP|ALDOA|QPRT|AMPD3|MPST|TST|UBA52|GPI|GPX1|RPL18|ALDH6A1|GALK1|DDX6	1.18E-11	GO.0044270	0.93545777
34	14	GO Process	NADH metabolic process	5E-10	OGDH|ENO1|GPD1L|GPD1|GPD2|PKM|MDH2|PKLR|PFKM|VCP|ALDOB|BPGM|ALDOA|GPI	1.34E-11	GO.0006734	0.93018995

424	41	GO Process	mitochondrion organization	5E-10	HSD17B10|NDUFB4|FIS1|PPIF|PMPCB|NDUFA2|NDUFA10|SYNJ2BP|DNAJA3|CHCHD3|NDUFA9|AFG3L2|ECSIT|NDUFB9|SLC25A4|ACAA2|NDUFB8|YWHAG|CYCS|RHOT2|NDUFV1|NDUFA12|UQCR10|AGK|LONP1|OPA1|COA3|NDUFS2|SLC25A5|APOOL|HMGCL|SLC25A6|CNP|ATP5A1|IMMT|NDUFS1|TOMM40|SSBP1|NDUFA13|PHB2|PHB	1.35E-11	GO.0007005	0.930103
265	32	GO Process	lipid catabolic process	5.5E-10	ECH1|EHHADH|ACADL|APOA1|PLBD1|ACADS|HEXB|MECR|ACAD11|RAB7A|CPT1A|SLC27A2|ACAA2|FABP1|ECI1|CYP4A11|ACAA1|ALDH3A2|GM2A|ECHS1|ABCD3|ACADM|CPT2|SCP2|ECHDC2|AUH|AKR1C3|ETFDH|ECHDC1|HADH|PLCG2|HAO2	1.5E-11	GO.0016042	0.92596373
484	44	GO Process	organic cyclic compound catabolic process	5.6E-10	AHCY|OGDH|DNPH1|ENO1|GDA|RPS16|DHTKD1|AMBP|DPYS|HNMT|QDPR|HGD|HPD|FTCD|HPRT1|PRODH2|RPS9|HINT1|HNRNPD|PKM|PKLR|PFKM|SND1|VCP|PNP|RPL5|OGDHL|ALDOB|ALDH4A1|MAOB|BPGM|CNP|ALDOA|QPRT|AMPD3|UBA52|GPI|GPX1|EPHX2|RPL18|PAH|ALDH6A1|GALK1|DDX6	1.54E-11	GO.1901361	0.92494916
41	15	GO Function	oxidoreductase activity, acting on the aldehyde or oxo group of donors	6.9E-10	OGDH|ALDH2|DHTKD1|ALDH8A1|BCKDHA|PDHB|ALDH3A2|ALDH9A1|OGDHL|ALDH4A1|ALDH1B1|PDHA1|AKR1C3|ALDH7A1|ALDH6A1	9.73E-12	GO.0016903	0.91611509
128	22	GO Component	inner mitochondrial membrane protein complex	9.5E-10	NDUFB4|PMPCB|NDUFA2|NDUFA10|CHCHD3|NDUFA9|AFG3L2|NDUFB9|NDUFB8|UQCRH|CYC1|NDUFV1|NDUFA12|UQCR10|AGK|NDUFS2|APOOL|SLC25A6|ATP5A1|IMMT|NDUFS1|NDUFA13	6.4E-11	GO.0098800	0.90241089
149	24	GO Process	nucleobase-containing small molecule biosynthetic process	1E-09	OGDH|DNPH1|ENO1|GDA|DHTKD1|HPRT1|HINT1|PKM|PKLR|PFKM|VCP|PNP|CMPK1|OGDHL|ALDOB|APRT|BPGM|NME2|CNP|ALDOA|AMPD3|GPI|GPX1|GALK1	2.88E-11	GO.0034404	0.89829667
172	25	GO Component	vacuolar lumen	1.2E-09	DCN|PYGB|CTSD|TTR|GGH|LYZ|HEXB|SCARB2|TPP1|GUSB|GAA|TOLLIP|PTGES2|CTSB|GM2A|VCP|HSPG2|CUBN|TXNDC5|ASAH1|PRKCD|PSAP|IFI30|LAMP2|NPC2	7.98E-11	GO.0005775	0.89393022
4347	183	GO Component	endomembrane system	1.2E-09	SYPL1|DCN|NDUFB4|VCL|GNPNAT1|ATP6V1D|PYGB|APMAP|F9|PSMD7|CTSH|EHD4|PLIN3|FIS1|LTF|REEP6|ATP6V1B1|AKR7A2|CTSD|APOA1|TTR|TFG|CANX|ACTN4|ATP6V1E1|NAPSA|LGALS3|ARL8B|GGH|RAB21|LYZ|HEXB|PSMD11|CDH1|RAB11A|COTL1|SLC9A3R1|RAB2A|PACSIN2|NAPA|LRP2|GRB14|RAB17|PSMD3|ATP6V0A1|F13A1|SCARB2|RAB7A|SLC27A2|IQGAP1|PSMA5|ARL8A|NAT8|FTH1|ATP6V1B2|ERLIN2|LAMTOR1|UCHL1|ATP6V0D2|HPD|FTCD|AGL|CAPN2|RNPEP|APEH|COL14A1|AMN|NDUFB8|ANPEP|COL6A2|GUSB|TMED10|UGT2B7|GAA|ATP6V0A4|ACO1|RAB1B|CYP4A11|AQP1|RAB6A|AOC3|DAB2|TOLLIP|AHCYL2|HSD11B2|CES2|PKM|UGT2B17|DHRS4|EHD3|AP2A2|COPB2|ATP6V0C|TMED9|SERPINA5|LAMP1|RAB11B|ACAA1|BSG|CYB5A|LAMTOR4|DHRS2|APOD|PTGES2|VAPA|CTSB|ALDH3A2|ANXA6|UGT1A9|COL18A1|GM2A|UBE2D3|VCP|COL4A2|PNP|COL6A1|CYB5R1|MGST3|MYO6|RPL5|ASS1|APOOL|GSN|RAB14|HNRNPR|HSPG2|CLIC1|COL4A1|TBC1D4|CUBN|CKAP4|APRT|TXNDC5|OCIAD1|ASAH1|CD9|SACM1L|NME2|NIT2|ACTN1|PRKCD|PSAP|ALDOA|KTN1|CD59|UMOD|MGST1|AMPD3|PDIA6|UBA52|SCRN1|IDH1|GDI1|ANXA11|RHOA|DDC|TANGO2|TMED4|GPI|LAMP2|AOC1|TOM1|SERPINA1|TGFBI|VWA1|MME|PPIA|TMEM33|ACSL1|OCIAD2|CAPN1|ATP1A1|RAN|RPL18|MGAM|CD63|NPC2|NDRG2|PHB|EPHX1|VPS29|PSMC3|FMO1	8.72E-11	GO.0012505	0.89100949

7824	287	GO Process	response to stimulus	1.2E-09	RALA|HEBP1|DCN|GNA11|NDUFB4|VCL|MYH9|ACO2|GNPNAT1|ATP6V1D|PYGB|AHCY|TXNL1|F9|PSMD7|CTSH|EHD4|HNRNPL|CDC37|FIS1|PPIF|LTF|REEP6|ATP6V1B1|ENO1|AKR7A2|CTSD|APOA1|TTR|GOT2|CANX|VIL1|HSPB1|ACTN4|LAMA5|ATP6V1E1|LGALS3|HRSP12|APCS|SYNJ2BP|GSTM3|TINAG|GGH|KHK|LYZ|HEXB|PSMD11|CDH1|DNAJA3|COTL1|SLC9A3R1|PRDX1|EPCAM|LRP2|GRB14|RAB17|PSMD3|ATP6V0A1|NNT|ADD1|F13A1|SCARB2|RAB7A|CALB1|PRDX5|CPT1A|SORD|SLC27A2|IQGAP1|AFG3L2|ARHGDIA|BCAM|ECSIT|TINAGL1|PSMA5|ARL8A|NAT8|ATP6V1A|FTH1|ATP6V1B2|ERLIN2|LAMTOR1|QDPR|SLC25A4|DDAH1|UCHL1|ACAA2|CA2|ATP6V0D2|AGL|CAPN2|FABP1|APEH|CMBL|HSPA9|HPRT1|AMN|TPP1|ANPEP|COL6A2|GPD1|GUSB|SLC23A1|TMED10|HINT1|GNB2|GAA|YWHAG|CYCS|ATP6V0A4|ACO1|DPYSL2|AQP1|RAB6A|AOC3|HNRNPD|DAB2|MSRA|TOLLIP|TLN1|HSD11B2|CYC1|CES2|SHMT1|PCK1|PKM|SLC7A8|TALDO1|RHOT2|BCAT2|HNRNPM|EHD3|ACSM2B|AP2A2|ATP6V0C|NDUFA12|SERPINA5|LAMP1|RAB11B|ACAA1|SHMT2|BSG|PARVA|GSTA1|PKLR|CYB5A|LAMTOR4|DHRS2|APOD|PTGES2|VAPA|CTSB|ANXA6|UGT1A9|COL18A1|GM2A|UBE2D3|VCP|ATP6V1H|MSN|MYH10|COL4A2|DPP4|LONP1|PNP|OPA1|KRT19|COL6A1|AKR7A3|MARC2|ATP1B1|MGST3|NDUFS2|PHGDH|GSTO1|FLNA|MYO6|ABCD3|GOT1|RBP4|SLC25A5|ASS1|GSN|RAB14|ATP6V1G1|CLIC4|HMGCL|HSPG2|PSMB8|ASPN|CLIC1|COL4A1|TBC1D4|CUBN|SLC3A2|RSU1|CKAP4|MAOB|APRT|TXNDC5|MT1M|BPHL|NQO2|AKR1C3|GLDC|SLC25A6|ASAH1|GPNMB|CD9|FECH|GPX3|KRT18|PRKAR1A|DPEP1|NME2|CNP|NIT2|PRKCD|DAK|PSAP|ALDOA|AK4|CD59|UMOD|MGST1|AMPD3|ABAT|GATM|ILK|MPST|GGT5|GGT1|TXNRD2|ACY1|IFI30|PDIA6|PTGR1|UBA52|SLC25A12|IDH1|GDI1|PDZK1|HIGD1A|ANXA11|CRYZ|RHOA|DDC|GPI|GPX1|FBP1|LAMP2|AOC1|TOM1|SERPINA1|TGFBI|CFB|VWA1|MME|FAM162A|PPIA|GSTA2|FLNB|TMEM33|ACSL1|NDUFA13|ETFDH|KIF13B|EPHX2|CAPN1|PHB2|ATP1A1|RAN|SLC25A10|MGAM|CD63|KRT8|NPC2|NDRG2|CIRBP|HADH|PHB|EPHX1|VPS29|PSMC3|FMO1|PLCG2|GLYAT	3.45E-11	GO.0050896	0.89100949
55	16	GO Process	aspartate family amino acid metabolic process	1.2E-09	MTHFD1|AHCY|CRYM|GOT2|HRSP12|BHMT2|BHMT|MSRA|AHCYL2|PIPOX|DLST|PHGDH|GOT1|ASS1|NIT2|ALDH7A1	3.48E-11	GO.0009066	0.89100949
87	19	GO Process	neurotransmitter metabolic process	1.3E-09	AGXT2|HRSP12|DMGDH|BHMT|DPYS|HNMT|SHMT1|CHDH|SHMT2|ALDH9A1|PHGDH|SARDH|MAOB|GLDC|ABAT|ALDH7A1|DDC|PAH|GLYAT	3.66E-11	GO.0042133	0.88894103
152	24	GO Process	organophosphate catabolic process	1.5E-09	OGDH|DNPH1|ENO1|GDA|DHTKD1|GPD1L|HPRT1|GPD1|HINT1|PKM|PKLR|PFKM|VCP|PNP|OGDHL|ALDOB|BPGM|CNP|ALDOA|AMPD3|GPI|GPX1|GALK1|PLCG2	4.17E-11	GO.0046434	0.88356471
167	25	GO Process	coenzyme biosynthetic process	1.6E-09	MTHFD1|OGDH|PDHX|ENO1|DHTKD1|NDUFA9|DLAT|QDPR|PDHB|ACSS1|PKM|IDH2|PKLR|PFKM|PNP|AKR1A1|OGDHL|ALDOB|PDHA1|BPGM|ALDOA|QPRT|GPI|ACSL1|GALK1	4.54E-11	GO.0009108	0.88013429
262	31	GO Process	response to xenobiotic stimulus	1.7E-09	AKR7A2|GOT2|GSTM3|CMBL|HPRT1|AQP1|AOC3|CES2|ACSM2B|UGT1A9|AKR7A3|MGST3|PHGDH|GSTO1|ASS1|BPHL|NQO2|FECH|DPEP1|MGST1|ABAT|GGT1|ACY1|CRYZ|GPX1|AOC1|ACSL1|EPHX2|EPHX1|FMO1|GLYAT	5E-11	GO.0009410	0.87644716
72	29	KEGG Pathways	Biosynthesis of amino acids	1.7E-20	ACO2|IDH3G|ENO1|GOT2|IDH3A|ASL|ACO1|SHMT1|PKM|TALDO1|BCAT2|IDH2|SHMT2|PKLR|CS|PFKM|PHGDH|GOT1|ASS1|PRPS1|ALDOB|PSAT1|IDH3B|PC|ALDOA|ACY1|IDH1|TKT|PAH	2.07E-22	hsa01230	1.97644716
109	21	GO Function	oxidoreductase activity, acting on NAD(P)H	1.8E-09	NDUFB4|TXNL1|NDUFA2|NDUFA10|NNT|NDUFA9|ECSIT|NDUFB9|CBR1|NDUFB8|DCXR|NDUFV1|DHRS4|NDUFA12|CYB5R1|NDUFS2|AKR1C3|TXNRD2|NDUFS1|CRYZ|NDUFA13	2.68E-11	GO.0016651	0.8747147
200	27	GO Process	monocarboxylic acid biosynthetic process	2.2E-09	DCN|OGDH|ENO1|DHTKD1|MECR|ALDH8A1|SLC27A2|CBR1|ACSS1|PKM|ACSM2B|PKLR|PTGES2|PFKM|ABCD3|HOGA1|SCP2|OGDHL|ALDOB|AKR1C3|BPGM|ALDOA|ABAT|GPI|EPHX2|ACSM2A|GALK1	6.3E-11	GO.0072330	0.86655462

103	20	GO Process	nucleoside triphosphate biosynthetic process	2.4E-09	OGDH|ENO1|DHTKD1|ATP6V0A1|ATP6V0A4|GBAS|CYC1|PKM|PKLR|PFKM|CMPK1|OGDHL|ALDOB|BPGM|NME2|ALDOA|AK4|ATP5A1|GPI|GALK1	7.07E-11	GO.0009142	0.86197888
97	19	GO Component	brush border	2.6E-09	VCL|MYH9|VIL1|ACTN4|SLC9A3R1|LRP2|ENPEP|SCIN|AMN|DCXR|ATP6V0A4|AQP1|MYH10|CLIC1|CUBN|ACTN1|PDZK1|MME|FLNB	1.86E-10	GO.0005903	0.85900669
53	15	GO Component	peroxisomal matrix	2.7E-09	CRYM|ECH1|EHHADH|PRDX5|FABP1|GRHPR|PIPOX|DHRS4|ACAA1|ABCD3|SCP2|HMGCL|IDH1|EPHX2|HAO2	1.99E-10	GO.0005782	0.85702477
59	16	GO Process	monosaccharide catabolic process	2.9E-09	ENO1|KHK|SORD|CRYL1|DCXR|PKM|PKLR|PFKM|AKR1A1|ALDOB|BPGM|DAK|ALDOA|GPI|GALK1|GALE	8.44E-11	GO.0046365	0.85451551
453	41	GO Process	aromatic compound catabolic process	3E-09	AHCY|OGDH|DNPH1|ENO1|GDA|RPS16|DHTKD1|AMBP|DPYS|HNMT|QDPR|HGD|HPD|FTCD|HPRT1|RPS9|HINT1|HNRNPD|PKM|PKLR|PFKM|SND1|VCP|PNP|RPL5|OGDHL|ALDOB|MAOB|BPGM|CNP|ALDOA|AMPD3|UBA52|GPI|GPX1|EPHX2|RPL18|PAH|GALK1|DDX6|EPHX1	8.9E-11	GO.0019439	0.85243288
1690	93	GO Process	nitrogen compound transport	3.2E-09	MYH9|ATP6V1D|ECH1|CDC37|FIS1|EHHADH|ATP6V1B1|CANX|HSPB1|PMPCB|RPS16|ACTN4|ATP6V1E1|SYNJ2BP|RAB21|RAB11A|SLC9A3R1|RAB2A|NAPA|LRP2|RAB17|ATP6V0A1|SCARB2|RAB7A|SLC27A2|RHCG|ATP6V1A|ATP6V1B2|SLC25A4|ATP6V0D2|HSPA9|AMN|SLC23A1|RPS9|TMED10|YWHAG|ATP6V0A4|RAB1B|AQP1|RAB6A|DAB2|GLS|PIPOX|SLC7A8|DHRS4|EHD3|AP2A2|COPB2|ATP6V0C|TMED9|RAB11B|ACAA1|AGK|UBE2D3|VCP|ATP6V1H|PNP|ATP1B1|MYO6|RPL5|SCP2|SLC25A5|RAB14|ATP6V1G1|HMGCL|TBC1D4|CUBN|SLC3A2|AKR1C3|SLC25A6|KRT18|PSAP|TST|UBA52|SLC25A12|IDH1|GDI1|PDZK1|DDC|TMED4|LAMP2|TOMM40|TOM1|NDUFA13|KIF13B|EPHX2|PHB2|RAN|RPL18|CD63|SLC43A2|VPS29|HAO2	9.51E-11	GO.0071705	0.84989407
28	9	KEGG Pathways	Butanoate metabolism	2.4E-06	EHHADH|ACADS|ACSM2B|ECHS1|HMGCL|BDH1|ABAT|ACSM2A|HADH	3.6E-07	hsa00650	0.56143937
58	16	GO Function	oxidoreductase activity, acting on NAD(P)H, quinone or similar compound as acceptor	4.3E-09	NDUFB4|NDUFA2|NDUFA10|NDUFA9|NDUFB9|CBR1|NDUFB8|DCXR|NDUFV1|DHRS4|NDUFA12|NDUFS2|AKR1C3|NDUFS1|CRYZ|NDUFA13	6.8E-11	GO.0016655	0.83685562
96	19	GO Process	respiratory electron transport chain	5.3E-09	NDUFB4|PMPCB|NDUFA2|NDUFA10|NDUFA9|NDUFB9|NDUFB8|GPD1|CYCS|GPD2|UQCRH|CYC1|NDUFV1|NDUFA12|UQCR10|NDUFS2|SLC25A12|NDUFS1|ETFDH	1.59E-10	GO.0022904	0.82773661
116	45	KEGG Pathways	Carbon metabolism	1.7E-31	ACO2|IDH3G|OGDH|EHHADH|ENO1|ACADS|GOT2|GLUD1|DLAT|IDH3A|PDHB|ACO1|ACSS1|SHMT1|PKM|TALDO1|MDH2|IDH2|SHMT2|DLST|PKLR|CS|PFKM|HIBCH|ECHS1|PHGDH|GOT1|ACADM|PRPS1|OGDHL|ALDOB|PSAT1|PDHA1|IDH3B|GLDC|PC|DAK|ALDOA|IDH1|TKT|GPI|FBP1|ME3|ALDH6A1|HAO2	1.35E-33	hsa01200	3.07721133
2268	113	GO Process	macromolecule localization	6.6E-09	VCL|MYH9|ATP6V1D|ECH1|CDC37|FIS1|EHHADH|ATP6V1B1|APOA1|GOT2|CANX|HSPB1|PMPCB|RPS16|ACTN4|LAMA5|ATP6V1E1|SYNJ2BP|RAB21|HEXB|CDH1|RAB11A|DNAJA3|SLC9A3R1|RAB2A|PACSIN2|NAPA|LRP2|RAB17|ATP6V0A1|SCARB2|RAB7A|CPT1A|SLC27A2|ATP6V1A|ATP6V1B2|LAMTOR1|ATP6V0D2|FABP1|SCIN|HSPA9|AMN|RPS9|TMED10|YWHAG|ATP6V0A4|RAB1B|RAB6A|DAB2|TOLLIP|TLN1|PIPOX|DHRS4|EHD3|AP2A2|COPB2|ATP6V0C|TMED9|SERPINA5|LAMP1|RAB11B|ACAA1|BSG|LAMTOR4|EPB41L3|APOD|VAPA|AGK|GM2A|UBE2D3|VCP|ATP6V1H|PNP|ATP1B1|FLNA|MYO6|ABCD3|RPL5|RBP4|CPT2|SCP2|GSN|RAB14|ATP6V1G1|HMGCL|TBC1D4|CUBN|AKR1C3|SLC25A6|KRT18|PSAP|TST|UBA52|IDH1|GDI1|TMED4|LAMP2|TOMM40|TOM1|FLNB|TMEM33|ACSL1|NDUFA13|KIF13B|EPHX2|PHB2|RAN|RPL18|CD63|TWF1|NPC2|VPS29|HAO2	2E-10	GO.0033036	0.81817741

86	18	GO Process	purine ribonucleoside triphosphate biosynthetic process	7.2E-09	OGDH|ENO1|DHTKD1|ATP6V0A1|ATP6V0A4|GBAS|CYC1|PKM|PKLR|PFKM|OGDHL|ALDOB|BPGM|NME2|ALDOA|ATP5A1|GPI|GALK1	2.2E-10	GO.0009206	0.81438756
75	17	GO Process	ATP biosynthetic process	7.8E-09	OGDH|ENO1|DHTKD1|ATP6V0A1|ATP6V0A4|GBAS|CYC1|PKM|PKLR|PFKM|OGDHL|ALDOB|BPGM|ALDOA|ATP5A1|GPI|GALK1	2.4E-10	GO.0006754	0.81079054
93	18	GO Component	respirasome	8.6E-09	NDUFB4|PMPCB|NDUFA2|NDUFA10|NNT|NDUFA9|NDUFB9|NDUFB8|CYCS|UQCRH|CYC1|NDUFV1|NDUFA12|UQCR10|NDUFS2|NDUFS1|HIGD1A|NDUFA13	6.64E-10	GO.0070469	0.80639892
113	20	GO Process	drug catabolic process	9.7E-09	CTSH|HRSP12|ALDH2|PRDX1|PRDX5|DPYS|QDPR|HGD|HPD|PIPOX|DLST|ALDH1B1|MAOB|GLDC|GPX3|BDH1|QPRT|AMPD3|GPX1|PAH	3.01E-10	GO.0042737	0.80150229
370	35	GO Component	apical part of cell	9.7E-09	REEP6|ATP6V1B1|ATP6V1E1|SLC9A3R1|EPCAM|LRP2|RAB17|ENPEP|RHCG|ATP6V1A|CA2|ATP6V0D2|FABP1|AMN|SLC23A1|ATP6V0A4|CYP4A11|AQP1|PFKM|GM2A|MSN|DPP4|ATP1B1|MYO6|AKR1A1|CLIC4|CUBN|SLC3A2|CD9|DPEP1|UMOD|MGST1|PDZK1|ATP1A1|MGAM	7.62E-10	GO.0045177	0.80127808
164	23	GO Component	tertiary granule	1.1E-08	CTSH|LTF|CTSD|LGALS3|GGH|LYZ|ATP6V0A1|ARL8A|FTH1|LAMTOR1|GAA|AP2A2|ATP6V0C|LAMP1|RAB14|ASAH1|NIT2|ALDOA|CD59|IDH1|RHOA|LAMP2|MGAM	8.57E-10	GO.0070820	0.79706162
298	31	GO Component	secretory granule membrane	1.1E-08	ATP6V1D|LGALS3|ATP6V0A1|RAB7A|SLC27A2|IQGAP1|ARL8A|LAMTOR1|ANPEP|TMED10|GAA|RAB6A|AP2A2|ATP6V0C|SERPINA5|LAMP1|BSG|VAPA|CYB5R1|RAB14|CKAP4|CD9|PSAP|CD59|MGST1|RHOA|LAMP2|TOM1|MME|MGAM|CD63	9.07E-10	GO.0030667	0.7954677
66	16	GO Process	NAD metabolic process	1.1E-08	OGDH|ENO1|GPD1L|GPD1|GPD2|PKM|MDH2|PKLR|PFKM|VCP|PNP|ALDOB|BPGM|ALDOA|QPRT|GPI	3.48E-10	GO.0019674	0.7954677
65	6	KEGG Pathways	Central carbon metabolism in cancer	0.0362	PDHB|GLS|PKM|PFKM|PDHA1|IDH1	0.0108	hsa05230	0.14412914
56	15	GO Process	nucleoside diphosphate phosphorylation	1.2E-08	OGDH|ENO1|DHTKD1|PKM|PKLR|PFKM|CMPK1|OGDHL|ALDOB|BPGM|NME2|ALDOA|AK4|GPI|GALK1	3.81E-10	GO.0006165	0.79172146
76	12	KEGG Pathways	Chemical carcinogenesis	1.9E-05	GSTM3|CBR1|UGT2B7|ADH1B|UGT2B17|GSTA1|UGT1A9|MGST3|GSTO1|MGST1|GSTA2|EPHX1	3.2E-06	hsa05204	0.47212464
440	39	GO Process	heterocycle catabolic process	1.4E-08	AHCY|OGDH|DNPH1|ENO1|GDA|RPS16|DHTKD1|AMBP|DPYS|HNMT|FTCD|HPRT1|PRODH2|RPS9|HINT1|HNRNPD|PKM|PKLR|PFKM|SND1|VCP|PNP|RPL5|OGDHL|ALDOB|ALDH4A1|DPEP1|BPGM|CNP|ALDOA|QPRT|AMPD3|UBA52|GPI|GPX1|RPL18|ALDH6A1|GALK1|DDX6	4.56E-10	GO.0046700	0.7844664
1427	81	GO Process	response to oxygen-containing compound	1.5E-08	DCN|GNA11|ATP6V1D|CTSH|HNRNPL|FIS1|PPIF|ATP6V1B1|GOT2|ATP6V1E1|GGH|KHK|CDH1|PRDX1|ATP6V0A1|PRDX5|CPT1A|IQGAP1|ATP6V1A|ATP6V1B2|LAMTOR1|QDPR|CA2|ATP6V0D2|CAPN2|FABP1|COL6A2|GPD1|YWHAG|ATP6V0A4|AQP1|HNRNPD|HSD11B2|CYC1|SHMT1|PCK1|PKM|ATP6V0C|RAB11B|BSG|PKLR|LAMTOR4|APOD|COL18A1|ATP6V1H|MSN|OPA1|COL6A1|GOT1|RBP4|ASS1|GSN|ATP6V1G1|HMGCL|COL4A1|TBC1D4|MAOB|APRT|AKR1C3|GLDC|CD9|FECH|GPX3|PRKAR1A|DPEP1|CNP|PRKCD|MGST1|ABAT|TXNRD2|RHOA|GPX1|AOC1|ACSL1|NDUFA13|PHB2|ATP1A1|KRT8|HADH|FMO1|PLCG2	4.91E-10	GO.1901700	0.78124793
290	31	GO Process	alcohol metabolic process	1.5E-08	ACO2|IDH3G|AKR7A2|APOA1|TTR|ALDH2|SORD|ERLIN2|QDPR|ACAA2|GPD1|ADH1B|GPD2|ACSS1|PCK1|DHRS4|IDH2|ALDH3A2|GOT1|RBP4|SCP2|ALDH1B1|CUBN|AKR1C3|IDH3B|DAK|IDH1|GK|NPC2|GALK1|PLCG2	4.95E-10	GO.0006066	0.78124793

1560	86	GO Process	immune response	1.6E-08	VCL|ATP6V1D|PYGB|AHCY|PSMD7|CTSH|LTF|CTSD|TTR|LGALS3|APCS|TINAG|GGH|LYZ|HEXB|PSMD11|DNAJA3|COTL1|PRDX1|RAB17|PSMD3|ATP6V0A1|RAB7A|SLC27A2|IQGAP1|ECSIT|TINAGL1|PSMA5|ARL8A|FTH1|LAMTOR1|AGL|APEH|HPRT1|ANPEP|GUSB|GAA|RAB6A|TOLLIP|PKM|AP2A2|ATP6V0C|LAMP1|ACAA1|SHMT2|PTGES2|VAPA|CTSB|GM2A|VCP|PNP|ASS1|GSN|RAB14|PSMB8|CKAP4|APRT|TXNDC5|ASAH1|NME2|NIT2|PRKCD|DAK|PSAP|ALDOA|CD59|MGST1|AMPD3|IFI30|IDH1|RHOA|GPI|LAMP2|AOC1|TOM1|SERPINA1|CFB|MME|PPIA|FLNB|CAPN1|MGAM|CD63|NPC2|PSMC3|PLCG2	5.13E-10	GO.0006955	0.77986029
40	13	GO Process	serine family amino acid metabolic process	2.4E-08	MTHFD1|AHCY|AGXT2|HRSP12|AHCYL2|SHMT1|SHMT2|PHGDH|PSAT1|GLDC|MPST|GGT1|GLYAT	7.91E-10	GO.0009069	0.76197888
988	63	GO Process	response to nitrogen compound	2.5E-08	GNA11|ATP6V1D|HNRNPL|FIS1|ATP6V1B1|GOT2|ATP6V1E1|GSTM3|GGH|KHK|CDH1|ATP6V0A1|IQGAP1|ATP6V1A|ATP6V1B2|ERLIN2|LAMTOR1|QDPR|CA2|ATP6V0D2|CAPN2|HPRT1|GPD1|TMED10|YWHAG|ATP6V0A4|AQP1|HNRNPD|HSD11B2|CYC1|SHMT1|PCK1|PKM|ATP6V0C|BSG|PKLR|LAMTOR4|VCP|ATP6V1H|OPA1|COL6A1|MARC2|MGST3|GOT1|ASS1|GSN|ATP6V1G1|COL4A1|TBC1D4|SLC3A2|APRT|GLDC|CD9|PRKAR1A|DPEP1|PRKCD|MGST1|ABAT|RHOA|DDC|AOC1|HADH|PSMC3	8.17E-10	GO.1901698	0.76090649
17	10	GO Process	amino-acid betaine metabolic process	2.5E-08	ACADL|DMGDH|BBOX1|CPT1A|BHMT|SHMT1|CHDH|ALDH9A1|ACADM|ALDH7A1	8.14E-10	GO.0006577	0.76090649
72	16	GO Process	pyridine-containing compound biosynthetic process	3.1E-08	OGDH|ENO1|DHTKD1|PKM|IDH2|PKLR|PFKM|PNP|OGDHL|ALDOB|PSAT1|BPGM|ALDOA|QPRT|GPI|GALK1	1.04E-09	GO.0072525	0.75072396
876	58	GO Process	response to organonitrogen compound	3.3E-08	ATP6V1D|HNRNPL|FIS1|ATP6V1B1|GOT2|ATP6V1E1|GGH|KHK|CDH1|ATP6V0A1|IQGAP1|ATP6V1A|ATP6V1B2|ERLIN2|LAMTOR1|QDPR|CA2|ATP6V0D2|CAPN2|HPRT1|GPD1|TMED10|YWHAG|ATP6V0A4|AQP1|HNRNPD|HSD11B2|CYC1|SHMT1|PCK1|PKM|ATP6V0C|BSG|PKLR|LAMTOR4|VCP|ATP6V1H|OPA1|COL6A1|MGST3|GOT1|ASS1|GSN|ATP6V1G1|COL4A1|TBC1D4|APRT|GLDC|CD9|PRKAR1A|PRKCD|MGST1|ABAT|RHOA|DDC|AOC1|HADH|PSMC3	1.11E-09	GO.0010243	0.74814861
42	13	GO Process	pyruvate biosynthetic process	3.8E-08	OGDH|ENO1|DHTKD1|PKM|PKLR|PFKM|HOGA1|OGDHL|ALDOB|BPGM|ALDOA|GPI|GALK1	1.3E-09	GO.0042866	0.74168012
27	11	Reactome Pathways	ROS, RNS production in phagocytes	4.6E-08	ATP6V1D|ATP6V1B1|ATP6V1E1|ATP6V0A1|ATP6V1A|ATP6V1B2|ATP6V0D2|ATP6V0A4|ATP6V0C|ATP6V1H|ATP6V1G1	2.35E-09	HSA-1222556	0.7334419
1440	80	GO Process	amide transport	4.8E-08	MYH9|ATP6V1D|ECH1|CDC37|FIS1|EHHADH|ATP6V1B1|CANX|HSPB1|PMPCB|RPS16|ACTN4|ATP6V1E1|SYNJ2BP|RAB21|RAB11A|SLC9A3R1|RAB2A|NAPA|LRP2|RAB17|ATP6V0A1|SCARB2|RAB7A|SLC27A2|ATP6V1A|ATP6V1B2|ATP6V0D2|HSPA9|AMN|RPS9|TMED10|YWHAG|ATP6V0A4|RAB1B|RAB6A|DAB2|PIPOX|DHRS4|EHD3|AP2A2|COPB2|ATP6V0C|TMED9|RAB11B|ACAA1|AGK|UBE2D3|VCP|ATP6V1H|PNP|ATP1B1|MYO6|RPL5|SCP2|RAB14|ATP6V1G1|HMGCL|TBC1D4|CUBN|AKR1C3|SLC25A6|KRT18|PSAP|UBA52|IDH1|GDI1|TMED4|LAMP2|TOMM40|TOM1|NDUFA13|KIF13B|EPHX2|PHB2|RAN|RPL18|CD63|VPS29|HAO2	1.64E-09	GO.0042886	0.73178549

1416	79	GO Process	peptide transport	5.1E-08	MYH9|ATP6V1D|ECH1|CDC37|FIS1|EHHADH|ATP6V1B1|CANX|HSPB1|PMPCB|RPS16|ACTN4|ATP6V1E1|SYNJ2BP|RAB21|RAB11A|SLC9A3R1|RAB2A|NAPA|LRP2|RAB17|ATP6V0A1|SCARB2|RAB7A|SLC27A2|ATP6V1A|ATP6V1B2|ATP6V0D2|HSPA9|AMN|RPS9|TMED10|YWHAG|ATP6V0A4|RAB1B|RAB6A|DAB2|PIPOX|DHRS4|EHD3|AP2A2|COPB2|ATP6V0C|TMED9|RAB11B|ACAA1|AGK|UBE2D3|VCP|ATP6V1H|PNP|ATP1B1|MYO6|RPL5|SCP2|RAB14|ATP6V1G1|HMGCL|TBC1D4|CUBN|AKR1C3|SLC25A6|KRT18|UBA52|IDH1|GDI1|TMED4|LAMP2|TOMM40|TOM1|NDUFA13|KIF13B|EPHX2|PHB2|RAN|RPL18|CD63|VPS29|HAO2	1.76E-09	GO.0015833	0.72898826
1390	78	GO Process	intracellular transport	5.1E-08	F9|EHD4|ECH1|CDC37|FIS1|EHHADH|TFG|HSPB1|PMPCB|RPS16|ACTN4|SYNJ2BP|RAB21|RAB11A|SLC9A3R1|RAB2A|NAPA|RAB17|SCARB2|RAB7A|CPT1A|SLC27A2|TINAGL1|LAMTOR1|UCHL1|HSPA9|RPS9|TMED10|YWHAG|RAB1B|RAB6A|CYC1|PIPOX|RHOT2|DHRS4|EHD3|AP2A2|COPB2|TMED9|LAMP1|RAB11B|ACAA1|VAPA|AGK|UBE2D3|VCP|MYH10|OPA1|ATP1B1|MYO6|ABCD3|RPL5|CPT2|SCP2|RAB14|HMGCL|TBC1D4|AKR1C3|SLC25A6|CD59|ATP5A1|UBA52|IDH1|LAMP2|TOMM40|TOM1|SERPINA1|TMEM33|NDUFA13|KIF13B|EPHX2|PHB2|RAN|RPL18|CD63|NPC2|VPS29|HAO2	1.78E-09	GO.0046907	0.72890369
1391	78	GO Process	protein transport	5.3E-08	MYH9|ATP6V1D|ECH1|CDC37|FIS1|EHHADH|ATP6V1B1|CANX|HSPB1|PMPCB|RPS16|ACTN4|ATP6V1E1|SYNJ2BP|RAB21|RAB11A|RAB2A|NAPA|LRP2|RAB17|ATP6V0A1|SCARB2|RAB7A|SLC27A2|ATP6V1A|ATP6V1B2|ATP6V0D2|HSPA9|AMN|RPS9|TMED10|YWHAG|ATP6V0A4|RAB1B|RAB6A|DAB2|PIPOX|DHRS4|EHD3|AP2A2|COPB2|ATP6V0C|TMED9|RAB11B|ACAA1|AGK|UBE2D3|VCP|ATP6V1H|PNP|ATP1B1|MYO6|RPL5|SCP2|RAB14|ATP6V1G1|HMGCL|TBC1D4|CUBN|AKR1C3|SLC25A6|KRT18|UBA52|IDH1|GDI1|TMED4|LAMP2|TOMM40|TOM1|NDUFA13|KIF13B|EPHX2|PHB2|RAN|RPL18|CD63|VPS29|HAO2	1.83E-09	GO.0015031	0.72781894
172	23	GO Process	sulfur compound biosynthetic process	5.6E-08	DCN|MTHFD1|AHCY|PDHX|BHMT2|GSTM3|BHMT|DLAT|PDHB|ACSS1|CNDP2|GSTA1|MGST3|GSTO1|AKR1A1|PDHA1|MGST1|MPST|HAGH|GGT5|GGT1|GSTA2|ACSL1	1.96E-09	GO.0044272	0.72502637
94	17	GO Component	lysosomal lumen	5.9E-08	DCN|CTSD|HEXB|SCARB2|TPP1|GUSB|GAA|CTSB|GM2A|HSPG2|CUBN|TXNDC5|ASAH1|PSAP|IFI30|LAMP2|NPC2	4.89E-09	GO.0043202	0.72298847
44	13	GO Process	ADP metabolic process	6E-08	OGDH|ENO1|DHTKD1|PKM|PKLR|PFKM|OGDHL|ALDOB|BPGM|ALDOA|AMPD3|GPI|GALK1	2.09E-09	GO.0046031	0.72247537
30	18	KEGG Pathways	Citrate cycle (TCA cycle)	4.7E-15	ACO2|PCK2|IDH3G|OGDH|DLAT|IDH3A|PDHB|ACO1|PCK1|MDH2|IDH2|DLST|CS|OGDHL|PDHA1|IDH3B|PC|IDH1	9.44E-17	hsa00020	1.4326058
82	16	GO Component	microvillus	6.3E-08	ATP6V1B1|VIL1|ATP6V1E1|SLC9A3R1|ATP6V1A|ATP6V1B2|CA2|DCXR|AOC3|MSN|MYO6|CLIC4|DPEP1|CNP|PDZK1|KIF13B	5.36E-09	GO.0005902	0.71985963
25	12	KEGG Pathways	Collecting duct acid secretion	1.7E-09	ATP6V1D|ATP6V1B1|ATP6V1E1|SLC4A1|ATP6V0A1|ATP6V1A|ATP6V1B2|CA2|ATP6V0D2|ATP6V0A4|ATP6V0C|ATP6V1G1	9.75E-11	hsa04966	0.87594508
468	39	GO Process	response to toxic substance	6.5E-08	TXNL1|FIS1|PPIF|GOT2|GSTM3|GGH|CDH1|PRDX1|NNT|PRDX5|FABP1|HPRT1|SLC23A1|AQP1|SLC7A8|GSTA1|DHRS2|MARC2|MGST3|GSTO1|RBP4|ASS1|GSN|MAOB|BPHL|FECH|GPX3|CNP|PRKCD|MGST1|ABAT|MPST|TXNRD2|RHOA|DDC|GPX1|EPHX2|EPHX1|GLYAT	2.31E-09	GO.0009636	0.71844223
491	40	GO Process	response to inorganic substance	7.4E-08	FIS1|PPIF|GGH|KHK|CDH1|PRDX1|ADD1|SORD|IQGAP1|QDPR|CA2|FABP1|ACO1|AQP1|HNRNPD|BSG|PKLR|CYB5A|COL18A1|LONP1|GOT1|ASS1|GSN|CLIC4|MAOB|MT1M|AKR1C3|CD9|FECH|DPEP1|PRKCD|ABAT|TXNRD2|SLC25A12|GDI1|ANXA11|GPX1|FBP1|AOC1|KRT8	2.61E-09	GO.0010035	0.71337127
36	12	GO Process	transferrin transport	7.9E-08	ATP6V1D|ATP6V1B1|ATP6V1E1|ATP6V0A1|ATP6V1A|ATP6V1B2|ATP6V0D2|ATP6V0A4|ATP6V0C|RAB11B|ATP6V1H|ATP6V1G1	2.82E-09	GO.0033572	0.7102923

<table>
<tr><td>11878</td><td>387</td><td>GO Function</td><td>binding</td><td>8E-08</td><td>RALA|HEBP1|DCN|GNA11|HSD17B10|CA12|EPDR1|VCL|NIPSNAP1|MYH9|ACO2|GNPNAT1|MTHFD1|PCK2|PYGB|AHCY|MYL12A|IDH3G|F9|PSMD7|CRYM|CTSH|EHD4|ECH1|HNRNPL|CDC37|OGDH|FIS1|PPIF|SLC25A3|DNPH1|AGXT2|LTF|EHHADH|FAHD2A|ACADL|ATP6V1B1|ENO1|APOA1|TTR|GDA|PFN2|TFG|ACADS|PEPD|GOT2|CAPNS1|CANX|VIL1|HSPB1|PMPCB|RPS16|ACTN4|LAMA5|ATP6V1E1|LGALS3|GRSF1|HRSP12|APCS|BHMT2|SYNJ2BP|ARL8B|GSTM3|NARS|TINAG|SQRDL|KHK|RAB21|LYZ|HEXB|ALDH2|CDH1|RAB11A|DNAJA3|SLC4A1|COTL1|CHCHD3|SLC9A3R1|RAB2A|PRDX1|DHTKD1|BBOX1|PACSIN2|NAPA|EPCAM|LRP2|CAPG|GRB14|RAB17|ATP6V0A1|NNT|ADD1|F13A1|SCARB2|ACAD11|RAB7A|AMBP|ENPEP|VDAC1|CALB1|PRDX5|CPT1A|REXO2|NDUFA9|RBP5|SORD|SLC27A2|RHCG|IQGAP1|AFG3L2|BCKDHA|BCAM|TINAGL1|ARL8A|ATP6V1A|FTH1|BHMT|ATP6V1B2|ERLIN2|DPYS|GLUD1|ENDOD1|LAMTOR1|DLAT|QDPR|GPD1L|HGD|DDAH1|UCHL1|CA2|HPD|PSMB4|FTCD|AGL|CAPN2|RNPEP|FABP1|APEH|SCIN|HSPA9|COL14A1|CFL2|CRYL1|HPRT1|AMN|CKB|TPP1|IDH3A|CDH16|ANPEP|GPD1|PRODH2|GUSB|RPS9|TMED10|DCXR|HINT1|GNB2|GAA|YWHAG|ADH1B|ASL|CYCS|ATP6V0A4|GPD2|ACO1|DPYSL2|RAB1B|CSRP2|CYP4A11|AQP1|RAB6A|AOC3|HNRNPD|DAB2|GRHPR|TSFM|TOLLIP|TLN1|HSD11B2|ACSS1|CYC1|PIPOX|SHMT1|UGDH|PCK1|CHDH|PKM|SLC7A8|TALDO1|RHOT2|NDUFV1|HNRNPM|CNDP2|DHRS4|MDH2|EHD3|ACSM2B|AP2A2|ATP6V0C|TMED9|C11orf54|IDH2|SERPINA5|LAMP1|RAB11B|SHMT2|BSG|PARVA|TRIM2|PKLR|CYB5A|EPB41L3|PPA2|APOD|PTGES2|VAPA|CTSB|PFKM|ALDH3A2|ANXA6|UGT1A9|WARS|AGK|COL18A1|PLS3|UBE2D3|PBLD|VCP|MSN|MYH10|DPP4|LONP1|PNP|OPA1|KIF21A|KRT19|COL6A1|AKR7A3|MARC2|CSRP1|ATP1B1|NDUFS2|LHPP|ACADSB|PHGDH|FLNA|MYO6|ABCD3|RPL5|GOT1|HOGA1|ACADM|XPNPEP2|RBP4|SCP2|SLC25A5|CMPK1|ASS1|PRPS1|GSN|RAB14|ATP6V1G1|OGDHL|HMGCL|HNRNPR|HSPG2|ALDOB|ALDH4A1|ASPN|VARS|AUH|COL4A1|AGMAT|KCTD12|TBC1D4|ALDH1B1|HIST1H2AC|CUBN|SLC3A2|MAOB|APRT|MT1M|NQO2|NAP1L4|IDH3B|GLDC|GPNMB|CD9|FECH|GPX3|KRT18|PRKAR1A|DPEP1|NME2|CNP|PC|FHL1|ACTN1|PRKCD|DAK|PSAP|ALDOA|KTN1|AK4|QPRT|CD59|UMOD|MGST1|MYH11|AMPD3|ABAT|ILK|MPST|HAGH|ATP5A1|SEPT7|TXNRD2|ACY1|ELAVL1|TST|SLC25A12|IDH1|TKT|NDUFS1|SLC4A4|GDI1|PDZK1|ANXA11|CRYZ|RHOA|GK|ACSF2|DDC|CKMT2|GPI|CKMT1A|GPX1|FBP1|LAMP2|LARS2|AOC1|TOM1|SERPINA1|TGFBI|CFB|VWA1|MME|GFM1|PPIA|SSBP1|FLNB|ACSL1|NDUFA13|ETFDH|WDR1|KIF13B|EPHX2|CAPN1|TAGLN|ME3|PHB2|ATP1A1|MMAB|RAN|RPL18|MGAM|PAH|KRT8|TWF1|ACSS3|ALDH6A1|NPC2|ACSM2A|GALK1|CIRBP|EML2|HADH|DDX6|PHB|PDLIM5|VPS29|PSMC3|FMO1|PLCG2|GALE|HAO2</td><td>1.35E-09</td><td>GO.0005488</td><td>0.7094744</td></tr>
<tr><td>44</td><td>11</td><td>KEGG Pathways</td><td>Cysteine and methionine metabolism</td><td>1.2E-06</td><td>AHCY|AGXT2|GOT2|BHMT2|BHMT|AHCYL2|BCAT2|MDH2|GOT1|MPST|TST</td><td>1.56E-07</td><td>hsa00270</td><td>0.5928118</td></tr>
<tr><td>5</td><td>2</td><td>KEGG Pathways</td><td>D-Glutamine and D-glutamate metabolism</td><td>0.043</td><td>GLUD1|GLS</td><td>0.0133</td><td>hsa00471</td><td>0.13665315</td></tr>
<tr><td>85</td><td>16</td><td>GO Component</td><td>mitochondrial respirasome</td><td>9.8E-08</td><td>NDUFB4|PMPCB|NDUFA2|NDUFA10|NNT|NDUFA9|NDUFB9|NDUFB8|UQCRH|CYC1|NDUFV1|NDUFA12|UQCR10|NDUFS2|NDUFS1|NDUFA13</td><td>8.41E-09</td><td>GO.0005746</td><td>0.70105502</td></tr>
</table>

1467	80	GO Process	establishment of protein localization	1E-07	MYH9|ATP6V1D|ECH1|CDC37|FIS1|EHHADH|ATP6V1B1|CANX|HSPB1|PMPCB|RPS16|ACTN4|ATP6V1E1|SYNJ2BP|RAB21|RAB11A|RAB2A|NAPA|LRP2|RAB17|ATP6V0A1|SCARB2|RAB7A|SLC27A2|ATP6V1A|ATP6V1B2|ATP6V0D2|HSPA9|AMN|RPS9|TMED10|YWHAG|ATP6V0A4|RAB1B|RAB6A|DAB2|PIPOX|DHRS4|EHD3|AP2A2|COPB2|ATP6V0C|TMED9|LAMP1|RAB11B|ACAA1|AGK|UBE2D3|VCP|ATP6V1H|PNP|ATP1B1|FLNA|MYO6|RPL5|SCP2|RAB14|ATP6V1G1|HMGCL|TBC1D4|CUBN|AKR1C3|SLC25A6|KRT18|UBA52|IDH1|GDI1|TMED4|LAMP2|TOMM40|TOM1|NDUFA13|KIF13B|EPHX2|PHB2|RAN|RPL18|CD63|VPS29|HAO2	3.66E-09	GO.0045184	0.69913998
68	15	GO Process	nicotinamide nucleotide biosynthetic process	1.1E-07	OGDH|ENO1|DHTKD1|PKM|IDH2|PKLR|PFKM|PNP|OGDHL|ALDOB|BPGM|ALDOA|QPRT|GPI|GALK1	3.79E-09	GO.0019359	0.69788107
80	16	GO Process	nucleoside diphosphate metabolic process	1.1E-07	OGDH|ENO1|DHTKD1|PKM|PKLR|PFKM|CMPK1|OGDHL|ALDOB|BPGM|NME2|ALDOA|AK4|AMPD3|GPI|GALK1	3.93E-09	GO.0009132	0.69665762
9	8	GO Process	glyoxylate metabolic process	1.2E-07	AGXT2|GOT2|PRODH2|GRHPR|IDH2|HOGA1|ALDH4A1|IDH1	4.25E-09	GO.0046487	0.6935542
1966	98	GO Process	protein localization	1.2E-07	VCL|MYH9|ATP6V1D|ECH1|CDC37|FIS1|EHHADH|ATP6V1B1|CANX|HSPB1|PMPCB|RPS16|ACTN4|LAMA5|ATP6V1E1|SYNJ2BP|RAB21|CDH1|RAB11A|DNAJA3|SLC9A3R1|RAB2A|PACSIN2|NAPA|LRP2|RAB17|ATP6V0A1|SCARB2|RAB7A|SLC27A2|ATP6V1A|ATP6V1B2|LAMTOR1|ATP6V0D2|SCIN|HSPA9|AMN|RPS9|TMED10|YWHAG|ATP6V0A4|RAB1B|RAB6A|DAB2|TOLLIP|TLN1|PIPOX|DHRS4|EHD3|AP2A2|COPB2|ATP6V0C|TMED9|LAMP1|RAB11B|ACAA1|BSG|LAMTOR4|EPB41L3|VAPA|AGK|UBE2D3|VCP|ATP6V1H|PNP|ATP1B1|FLNA|MYO6|RPL5|SCP2|GSN|RAB14|ATP6V1G1|HMGCL|TBC1D4|CUBN|AKR1C3|SLC25A6|KRT18|UBA52|IDH1|GDI1|TMED4|LAMP2|TOMM40|TOM1|FLNB|TMEM33|NDUFA13|KIF13B|EPHX2|PHB2|RAN|RPL18|CD63|TWF1|VPS29|HAO2	4.27E-09	GO.0008104	0.6935542
69	15	GO Process	glutamine family amino acid metabolic process	1.2E-07	GOT2|GLUD1|DDAH1|FTCD|GLYATL1|PRODH2|ASL|GLS|PHGDH|GOT1|ASS1|ALDH4A1|AGMAT|NIT2|GGT1	4.51E-09	GO.0009064	0.69136402
38	12	GO Process	glycolytic process	1.3E-07	OGDH|ENO1|DHTKD1|PKM|PKLR|PFKM|OGDHL|ALDOB|BPGM|ALDOA|GPI|GALK1	4.68E-09	GO.0006096	0.68996295
66	12	KEGG Pathways	Drug metabolism - cytochrome P450	5.7E-06	GSTM3|UGT2B7|ADH1B|UGT2B17|GSTA1|UGT1A9|MGST3|GSTO1|MAOB|MGST1|GSTA2|FMO1	8.59E-07	hsa00982	0.52479516
39	12	GO Process	trivalent inorganic cation transport	1.6E-07	ATP6V1D|ATP6V1B1|ATP6V1E1|ATP6V0A1|ATP6V1A|ATP6V1B2|ATP6V0D2|ATP6V0A4|ATP6V0C|RAB11B|ATP6V1H|ATP6V1G1	5.98E-09	GO.0072512	0.67986029
2180	105	GO Process	cellular localization	1.6E-07	RALA|VCL|MYH9|ATP6V1D|F9|EHD4|ECH1|CDC37|FIS1|LTF|EHHADH|TFG|CANX|HSPB1|PMPCB|RPS16|ACTN4|LAMA5|SYNJ2BP|ARL8B|RAB21|CDH1|RAB11A|DNAJA3|SLC9A3R1|RAB2A|PACSIN2|NAPA|RAB17|SCARB2|RAB7A|CPT1A|SLC27A2|TINAGL1|FTH1|LAMTOR1|UCHL1|SCIN|HSPA9|AMN|RPS9|TMED10|GAA|YWHAG|RAB1B|RAB6A|TOLLIP|TLN1|CYC1|PIPOX|RHOT2|DHRS4|EHD3|AP2A2|COPB2|TMED9|LAMP1|RAB11B|ACAA1|BSG|LAMTOR4|EPB41L3|VAPA|AGK|UBE2D3|VCP|MYH10|OPA1|ATP1B1|FLNA|MYO6|ABCD3|RPL5|CPT2|SCP2|GSN|RAB14|HMGCL|TBC1D4|AKR1C3|SLC25A6|KRT18|CD59|ATP5A1|UBA52|IDH1|DDC|LAMP2|TOMM40|TOM1|SERPINA1|FLNB|TMEM33|NDUFA13|KIF13B|EPHX2|PHB2|RAN|RPL18|CD63|TWF1|NPC2|VPS29|PLCG2|HAO2	6.15E-09	GO.0051641	0.6790485
71	15	GO Process	iron ion transport	1.7E-07	ATP6V1D|LTF|ATP6V1B1|ATP6V1E1|ATP6V0A1|ATP6V1A|FTH1|ATP6V1B2|ATP6V0D2|ATP6V0A4|SFXN1|ATP6V0C|RAB11B|ATP6V1H|ATP6V1G1	6.32E-09	GO.0006826	0.67798919
25	10	GO Component	proton-transporting V-type ATPase complex	1.7E-07	ATP6V1D|ATP6V1B1|ATP6V0A1|ATP6V1A|ATP6V1B2|ATP6V0D2|ATP6V0A4|ATP6V0C|ATP6V1H|ATP6V1G1	1.47E-08	GO.0033176	0.67772835

64	14	Reactome Pathways	Mitochondrial protein import	2E-07	ACO2|IDH3G|PMPCB|CHCHD3|VDAC1|SLC25A4|HSPA9|NDUFB8|CYC1|CS|SLC25A6|ATP5A1|SLC25A12|TOMM40	1.38E-08	HSA-1268020	0.66903698
2815	126	GO Process	response to organic substance	2.3E-07	RALA|DCN|GNA11|GNPNAT1|ATP6V1D|CTSH|EHD4|HNRNPL|FIS1|ATP6V1B1|GOT2|CANX|VIL1|HSPB1|LAMA5|ATP6V1E1|HRSP12|GGH|KHK|CDH1|DNAJA3|ATP6V0A1|ADD1|F13A1|CALB1|CPT1A|SORD|IQGAP1|ATP6V1A|ATP6V1B2|ERLIN2|LAMTOR1|QDPR|CA2|ATP6V0D2|AGL|CAPN2|HSPA9|HPRT1|TPP1|COL6A2|GPD1|TMED10|YWHAG|ATP6V0A4|AQP1|HNRNPD|TOLLIP|TLN1|HSD11B2|CYC1|SHMT1|PCK1|PKM|TALDO1|BCAT2|HNRNPM|ATP6V0C|RAB11B|SHMT2|BSG|PKLR|LAMTOR4|CTSB|UBE2D3|VCP|ATP6V1H|MSN|COL4A2|LONP1|OPA1|COL6A1|MGST3|GSTO1|ABCD3|GOT1|RBP4|SLC25A5|ASS1|GSN|RAB14|ATP6V1G1|HMGCL|PSMB8|ASPN|COL4A1|TBC1D4|SLC3A2|MAOB|APRT|AKR1C3|GLDC|CD9|FECH|GPX3|KRT18|PRKAR1A|CNP|PRKCD|PSAP|MGST1|ABAT|ILK|IFI30|PDIA6|UBA52|IDH1|RHOA|DDC|AOC1|MME|PPIA|GSTA2|FLNB|ACSL1|NDUFA13|PHB2|ATP1A1|RAN|KRT8|HADH|PHB|EPHX1|PSMC3|FMO1|PLCG2	8.95E-09	GO.0010033	0.66307841
79	15	GO Component	respiratory chain complex	2.5E-07	NDUFB4|PMPCB|NDUFA2|NDUFA10|NDUFA9|NDUFB9|NDUFB8|UQCRH|CYC1|NDUFV1|NDUFA12|UQCR10|NDUFS2|NDUFS1|NDUFA13	2.22E-08	GO.0098803	0.66038007
41	12	GO Process	cellular modified amino acid biosynthetic process	2.5E-07	MTHFD1|BBOX1|SHMT1|CHDH|CNDP2|ALDH9A1|ACADM|GATM|HAGH|GGT5|GGT1|ALDH7A1	9.58E-09	GO.0042398	0.66038007
307	29	GO Component	apical plasma membrane	2.5E-07	ATP6V1B1|ATP6V1E1|SLC9A3R1|EPCAM|LRP2|RAB17|ENPEP|RHCG|ATP6V1A|ATP6V0D2|AMN|SLC23A1|ATP6V0A4|CYP4A11|AQP1|PFKM|GM2A|MSN|DPP4|ATP1B1|AKR1A1|CUBN|SLC3A2|CD9|DPEP1|UMOD|PDZK1|ATP1A1|MGAM	2.3E-08	GO.0016324	0.65968795
135	20	GO Function	vitamin binding	2.7E-07	PYGB|OGDH|AGXT2|GOT2|DHTKD1|CALB1|RBP5|FTCD|SHMT1|SHMT2|MARC2|GOT1|RBP4|OGDHL|CUBN|GLDC|PC|ABAT|DDC|MMAB	4.71E-09	GO.0019842	0.65734887
80	15	GO Component	extracellular exosome	2.8E-07	RAB11A|SORD|BHMT|ANPEP|AQP1|SERPINA5|XPNPEP2|CLIC1|CUBN|CD9|ALDOA|ACY1|LAMP2|AOC1|CD63	2.58E-08	GO.0070062	0.65543958
100	17	GO Process	oxidative phosphorylation	2.9E-07	NDUFB4|PMPCB|NDUFA2|NDUFA10|NDUFA9|NDUFB9|NDUFB8|CYCS|UQCRH|GBAS|CYC1|NDUFV1|NDUFA12|UQCR10|NDUFS2|ATP5A1|NDUFS1	1.11E-08	GO.0006119	0.65421181
88	16	GO Process	acyl-CoA metabolic process	3.3E-07	OGDH|PDHX|ACOT13|DLAT|PDHB|ACSS1|PIPOX|ACSM2B|DLST|HMGCL|ACOT9|PDHA1|ACSF2|ACSL1|ACSM2A|GLYAT	1.3E-08	GO.0006637	0.64762535
318	30	GO Process	protein targeting	3.4E-07	ECH1|CDC37|FIS1|EHHADH|PMPCB|RPS16|SYNJ2BP|SCARB2|RAB7A|SLC27A2|RPS9|YWHAG|PIPOX|DHRS4|ACAA1|AGK|UBE2D3|RPL5|SCP2|HMGCL|SLC25A6|UBA52|IDH1|LAMP2|TOMM40|NDUFA13|KIF13B|EPHX2|RPL18|HAO2	1.35E-08	GO.0006605	0.64647059
76	14	KEGG Pathways	Drug metabolism - other enzymes	7.9E-07	GSTM3|DPYS|HPRT1|GUSB|UGT2B7|CES2|UGT2B17|GSTA1|UGT1A9|MGST3|GSTO1|NME2|MGST1|GSTA2	9.13E-08	hsa00983	0.61040253
245	26	GO Process	lipid modification	3.7E-07	ECH1|EHHADH|ACADL|ACADS|MECR|ACAD11|CPT1A|SLC27A2|ACAA2|ECI1|ACAA1|ALDH3A2|AGK|ECHS1|ABCD3|ACADM|CPT2|SCP2|ECHDC2|AUH|SACM1L|ETFDH|EPHX2|ECHDC1|HADH|HAO2	1.45E-08	GO.0030258	0.64353339
47	5	KEGG Pathways	Endocrine and other factor-regulated calcium reabsorption	0.0381	RAB11A|CALB1|AP2A2|ATP1B1|ATP1A1	0.0116	hsa04961	0.1419075
339	31	GO Process	response to metal ion	3.8E-07	PPIF|GGH|KHK|CDH1|ADD1|SORD|IQGAP1|QDPR|CA2|ACO1|AQP1|HNRNPD|BSG|PKLR|CYB5A|LONP1|GOT1|ASS1|GSN|CLIC4|MAOB|MT1M|AKR1C3|FECH|DPEP1|ABAT|SLC25A12|GDI1|ANXA11|FBP1|AOC1	1.5E-08	GO.0010038	0.64213608

1491	79	GO Process	homeostatic process	3.9E-07	DCN|CA12|MTHFD1|TXNL1|CTSH|FIS1|SFXN3|LTF|ACADL|ATP6V1B1|APOA1|NAPSA|HEXB|RAB11A|DNAJA3|SLC4A1|SLC9A3R1|PRDX1|ATP6V0A1|NNT|ADD1|RAB7A|CALB1|PRDX5|RHCG|AFG3L2|ATP6V1A|FTH1|LAMTOR1|GPD1L|CA2|ATP6V0D2|HSPA9|CFL2|CKB|TPP1|GAA|ATP6V0A4|ACO1|CYP4A11|AQP1|SFXN1|PCK1|SLC7A8|RHOT2|ATP6V0C|RAB11B|PTGES2|PFKM|ANXA6|PBLD|ATP6V1H|OPA1|ATP1B1|GSTO1|SFXN2|GOT1|RBP4|ATP6V1G1|CLIC4|CUBN|TXNDC5|PRKAR1A|BPGM|FHL1|ALDOA|UMOD|AMPD3|TXNRD2|PDIA6|IMMT|SLC4A4|GPX1|LAMP2|EPHX2|ATP1A1|NPC2|ACSM2A|PLCG2	1.55E-08	GO.0042592	0.64089354
8349	290	GO Process	nitrogen compound metabolic process	3.9E-07	PSMA4|DCN|HSD17B10|NDUFB4|NANS|MYH9|GNPNAT1|MTHFD1|AHCY|F9|PSMD7|CRYM|CTSH|HNRNPL|CDC37|OGDH|PPIF|PDHX|ACOT13|DNPH1|AGXT2|LTF|EHHADH|ACADL|ATP6V1B1|ENO1|AKR7A2|CTSD|APOA1|TTR|GDA|PLBD1|PEPD|GOT2|CAPNS1|PMPCB|RPS16|NDUFA2|NDUFA10|NAPSA|LGALS3|GRSF1|HRSP12|APCS|DMGDH|BHMT2|GSTM3|NARS|TINAG|GGH|LYZ|HEXB|PSMD11|RAB11A|DNAJA3|CHCHD3|RAB2A|DHTKD1|BBOX1|PSMD3|ATP6V0A1|NNT|ADD1|F13A1|RAB7A|AMBP|ENPEP|CALB1|PRDX5|CPT1A|REXO2|NDUFA9|AFG3L2|BCKDHA|TINAGL1|PSMA5|NAT8|ATP6V1A|BHMT|ATP6V1B2|ERLIN2|DPYS|NDUFB9|GLUD1|ENDOD1|HNMT|DLAT|QDPR|GPD1L|HGD|DDAH1|UCHL1|HPD|PSMB4|FTCD|CAPN2|RNPEP|APEH|HPRT1|AMN|NDUFB8|CKB|TPP1|ANPEP|GLYATL1|GPD1|PRODH2|GUSB|SLC23A1|RPS9|DCXR|HINT1|ASL|PDHB|CYCS|ATP6V0A4|GPD2|DPYSL2|UQCRH|RAB1B|AQP1|RAB6A|AOC3|GBAS|HNRNPD|GRHPR|TSFM|MSRA|TOLLIP|AHCYL2|ACSS1|CYC1|GLS|PIPOX|SHMT1|UGDH|PCK1|CHDH|PKM|SLC7A8|TALDO1|NDUFV1|BCAT2|HNRNPM|CNDP2|MDH2|ACSM2B|AP2A2|NDUFA12|IDH2|UQCR10|RAB11B|SHMT2|DLST|GSTA1|TRIM2|PKLR|PPA2|VAPA|CTSB|PFKM|ALDH3A2|SND1|ALDH9A1|WARS|AGK|GM2A|UBE2D3|VCP|HIBCH|COL4A2|DPP4|LONP1|MRPL37|MRPL24|PNP|MRPL21|OPA1|IBA57|MARC2|ATP1B1|MGST3|NDUFS2|LHPP|ACADSB|PHGDH|GSTO1|FLNA|RPL5|GOT1|HOGA1|ACADM|XPNPEP2|SARDH|CMPK1|AKR1A1|ASS1|PRPS1|GSN|OGDHL|HMGCL|HNRNPR|HSPG2|ALDOB|PSMB9|PSMB8|ALDH4A1|VARS|AUH|AGMAT|PSAT1|CUBN|CKAP4|MAOB|APRT|ACOT9|PDHA1|BPHL|AKR1C3|GLDC|ASAH1|FECH|DPEP1|BPGM|NME2|CNP|PC|NIT2|PRKCD|PSAP|ALDOA|KTN1|AK4|QPRT|MGST1|AMPD3|ABAT|GATM|ILK|MPST|HAGH|GGT5|ATP5A1|GGT1|ACY1|ELAVL1|PDIA6|TST|ALDH7A1|UBA52|SCRN1|IDH1|TKT|NDUFS1|RHOA|ACSF2|DDC|CKMT2|GPI|CKMT1A|GPX1|LAMP2|LARS2|AOC1|SERPINA1|TGFBI|CFB|VWA1|MRPS22|MME|GFM1|PPIA|SSBP1|ABHD14B|GSTA2|ACSL1|CAPN1|PHB2|MMAB|RAN|RPL18|PAH|ALDH6A1|ACSM2A|GALK1|DDX6|PHB|PSMC3|FMO1|GLYAT	1.57E-08	GO.0006807	0.64078232
66	12	KEGG Pathways	Epithelial cell signaling in Helicobacter pylori infection	5.7E-06	ATP6V1D|ATP6V1B1|ATP6V1E1|ATP6V0A1|ATP6V1A|ATP6V1B2|ATP6V0D2|ATP6V0A4|ATP6V0C|ATP6V1H|ATP6V1G1|PLCG2	8.59E-07	hsa05120	0.52479516
44	20	KEGG Pathways	Fatty acid degradation	4.8E-15	EHHADH|ACADL|ACADS|ALDH2|CPT1A|ACAA2|ECI1|ADH1B|CYP4A11|ACAA1|ALDH3A2|ALDH9A1|ECHS1|ACADSB|ACADM|CPT2|ALDH1B1|ALDH7A1|ACSL1|HADH	1.16E-16	hsa00071	1.43160529
31	11	GO Function	oxidoreductase activity, acting on the aldehyde or oxo group of donors, NAD or NADP as acceptor	4.1E-07	OGDH|ALDH2|ALDH8A1|ALDH3A2|ALDH9A1|ALDH4A1|ALDH1B1|PDHA1|AKR1C3|ALDH7A1|ALDH6A1	7.61E-09	GO.0016620	0.63861582
78	15	GO Process	mitochondrial ATP synthesis coupled electron transport	4.8E-07	NDUFB4|PMPCB|NDUFA2|NDUFA10|NDUFA9|NDUFB9|NDUFB8|CYCS|UQCRH|CYC1|NDUFV1|NDUFA12|UQCR10|NDUFS2|NDUFS1	1.91E-08	GO.0042775	0.6322393

51	13	GO Function	carbon-carbon lyase activity	4.9E-07	PCK2|BCKDHA|SHMT1|PCK1|SHMT2|GOT1|HOGA1|HMGCL|ALDOB|ALDOA|DDC|ECHDC1|ME3	9.49E-09	GO.0016830	0.63106911
25	4	KEGG Pathways	Fatty acid elongation	0.0234	MECR|ACAA2|ECHS1|HADH	0.0066	hsa00062	0.16307841
49	12	GO Component	proton-transporting two-sector ATPase complex	5.5E-07	ATP6V1D|ATP6V1B1|ATP6V1E1|ATP6V0A1|ATP6V1A|ATP6V1B2|ATP6V0D2|ATP6V0A4|ATP6V0C|ATP6V1H|ATP6V1G1|ATP5A1	5.17E-08	GO.0016469	0.62596373
79	15	GO Process	peroxisome organization	5.5E-07	ECH1|FIS1|EHHADH|SLC27A2|PIPOX|DHRS4|ACAA1|UBE2D3|ABCD3|SCP2|HMGCL|UBA52|IDH1|EPHX2|HAO2	2.22E-08	GO.0007031	0.62596373
1616	83	GO Process	establishment of localization in cell	5.9E-07	MYH9|F9|EHD4|ECH1|CDC37|FIS1|LTF|EHHADH|TFG|CANX|HSPB1|PMPCB|RPS16|ACTN4|SYNJ2BP|RAB21|RAB11A|SLC9A3R1|RAB2A|NAPA|RAB17|SCARB2|RAB7A|CPT1A|SLC27A2|TINAGL1|LAMTOR1|UCHL1|HSPA9|RPS9|TMED10|YWHAG|RAB1B|RAB6A|CYC1|PIPOX|RHOT2|DHRS4|EHD3|AP2A2|COPB2|TMED9|LAMP1|RAB11B|ACAA1|VAPA|AGK|UBE2D3|VCP|MYH10|OPA1|ATP1B1|MYO6|ABCD3|RPL5|CPT2|SCP2|RAB14|HMGCL|TBC1D4|AKR1C3|SLC25A6|CD59|ATP5A1|UBA52|IDH1|DDC|LAMP2|TOMM40|TOM1|SERPINA1|TMEM33|NDUFA13|KIF13B|EPHX2|PHB2|RAN|RPL18|CD63|NPC2|VPS29|PLCG2|HAO2	2.39E-08	GO.0051649	0.62328441
2370	109	GO Process	immune system process	7.8E-07	VCL|MYH9|ATP6V1D|MTHFD1|PYGB|AHCY|PSMD7|CTSH|LTF|CTSD|TTR|CANX|LGALS3|APCS|TINAG|GGH|LYZ|HEXB|PSMD11|DNAJA3|COTL1|PRDX1|DHTKD1|EPCAM|GRB14|RAB17|PSMD3|ATP6V0A1|ADD1|RAB7A|SLC27A2|IQGAP1|ECSIT|TINAGL1|PSMA5|ARL8A|FTH1|LAMTOR1|AGL|APEH|HSPA9|HPRT1|ANPEP|GUSB|GAA|RAB6A|TOLLIP|SFXN1|PKM|SLC7A8|AP2A2|ATP6V0C|LAMP1|ACAA1|SHMT2|BSG|DHRS2|PTGES2|VAPA|CTSB|GM2A|VCP|MSN|DPP4|PNP|ATP1B1|ASS1|GSN|RAB14|PSMB9|PSMB8|SLC3A2|CKAP4|APRT|TXNDC5|ASAH1|BPGM|NME2|NIT2|ACTN1|PRKCD|DAK|PSAP|ALDOA|CD59|UMOD|MGST1|AMPD3|IFI30|UBA52|IDH1|RHOA|GPI|LAMP2|AOC1|TOM1|SERPINA1|CFB|MME|PPIA|FLNB|WDR1|KIF13B|CAPN1|MGAM|CD63|NPC2|PSMC3|PLCG2	3.2E-08	GO.0002376	0.6107349
48	13	KEGG Pathways	Fatty acid metabolism	6.4E-08	EHHADH|ACADL|ACADS|MECR|CPT1A|ACAA2|ACAA1|ECHS1|ACADSB|ACADM|CPT2|ACSL1|HADH	5.1E-09	hsa01212	0.71958606
197	16	KEGG Pathways	Focal adhesion	0.00077	VCL|MYL12A|ACTN4|LAMA5|CAPN2|COL6A2|TLN1|PARVA|COL4A2|COL6A1|FLNA|COL4A1|ACTN1|ILK|RHOA|FLNB	0.00017	hsa04510	0.31135093
26	5	KEGG Pathways	Folate biosynthesis	0.005	GGH|QDPR|CBR1|AKR1C3|PAH	0.0012	hsa00790	0.230103
724	47	GO Component	cytoplasmic vesicle membrane	8.4E-07	RALA|SYPL1|ATP6V1D|ATP6V1B1|LGALS3|RAB21|RAB11A|PACSIN2|LRP2|ATP6V0A1|SCARB2|RAB7A|SLC27A2|IQGAP1|ARL8A|LAMTOR1|ATP6V0D2|ANPEP|TMED10|GAA|ATP6V0A4|RAB6A|DAB2|AP2A2|COPB2|ATP6V0C|SERPINA5|LAMP1|RAB11B|BSG|VAPA|CYB5R1|MYO6|RAB14|CLIC4|CKAP4|CD9|PSAP|CD59|MGST1|UBA52|RHOA|LAMP2|TOM1|MME|MGAM|CD63	8.17E-08	GO.0030659	0.60741724
28	10	GO Process	cellular modified amino acid catabolic process	8.5E-07	AHCY|ACADL|GOT2|DMGDH|BHMT|HOGA1|SARDH|ALDH4A1|GGT5|GGT1	3.52E-08	GO.0042219	0.6069051
70	14	GO Process	peroxisomal transport	8.9E-07	ECH1|EHHADH|SLC27A2|PIPOX|DHRS4|ACAA1|UBE2D3|ABCD3|SCP2|HMGCL|UBA52|IDH1|EPHX2|HAO2	3.7E-08	GO.0043574	0.60525663
33	7	KEGG Pathways	Fructose and mannose metabolism	0.00035	KHK|SORD|PFKM|ALDOB|DAK|ALDOA|FBP1	0.000073	hsa00051	0.3455932

1367	73	GO Process	cellular protein localization	1.1E-06	VCL|ATP6V1D|ECH1|CDC37|FIS1|EHHADH|HSPB1|PMPCB|RPS16|LAMA5|SYNJ2BP|CDH1|RAB11A|DNAJA3|SLC9A3R1|PACSIN2|NAPA|SCARB2|RAB7A|SLC27A2|LAMTOR1|SCIN|HSPA9|AMN|RPS9|TMED10|YWHAG|RAB6A|TOLLIP|TLN1|PIPOX|DHRS4|EHD3|AP2A2|COPB2|LAMP1|RAB11B|ACAA1|BSG|LAMTOR4|EPB41L3|VAPA|AGK|UBE2D3|VCP|ATP1B1|FLNA|MYO6|RPL5|SCP2|GSN|HMGCL|TBC1D4|AKR1C3|SLC25A6|KRT18|UBA52|IDH1|LAMP2|TOMM40|TOM1|FLNB|TMEM33|NDUFA13|KIF13B|EPHX2|PHB2|RAN|RPL18|CD63|TWF1|VPS29|HAO2	4.4E-08	GO.0034613	0.59788107
21	9	GO Process	neurotransmitter catabolic process	1.2E-06	DMGDH|BHMT|HNMT|CHDH|SARDH|MAOB|GLDC|ABAT|ALDH7A1	4.88E-08	GO.0042135	0.5935542
106	17	GO Function	exopeptidase activity	1.2E-06	CTSH|PEPD|GGH|ENPEP|UCHL1|RNPEP|APEH|TPP1|ANPEP|CNDP2|DPP4|XPNPEP2|DPEP1|GGT5|GGT1|SCRN1|MME	2.39E-08	GO.0008238	0.5928118
31	5	KEGG Pathways	Galactose metabolism	0.0095	GAA|PFKM|MGAM|GALK1|GALE	0.0024	hsa00052	0.20222764
100	8	KEGG Pathways	Glucagon signaling pathway	0.0262	PCK2|PYGB|CPT1A|PDHB|PCK1|PKM|PDHA1|FBP1	0.0077	hsa04922	0.15816987
49	12	GO Process	hexose catabolic process	1.2E-06	ENO1|KHK|PKM|PKLR|PFKM|ALDOB|BPGM|DAK|ALDOA|GPI|GALK1|GALE	5.17E-08	GO.0019320	0.59172146
362	31	GO Process	response to peptide hormone	1.4E-06	ATP6V1D|ATP6V1B1|GOT2|ATP6V1E1|GGH|KHK|ATP6V0A1|IQGAP1|ATP6V1A|ATP6V1B2|QDPR|CA2|ATP6V0D2|YWHAG|ATP6V0A4|HSD11B2|CYC1|PCK1|PKM|ATP6V0C|BSG|PKLR|ATP6V1H|GOT1|ASS1|ATP6V1G1|TBC1D4|APRT|PRKAR1A|PRKCD|HADH	6E-08	GO.0043434	0.58569852
50	14	KEGG Pathways	Glutathione metabolism	1.3E-08	GSTM3|NAT8|ANPEP|IDH2|GSTA1|MGST3|GSTO1|GPX3|MGST1|GGT5|GGT1|IDH1|GPX1|GSTA2	9.04E-10	hsa00480	0.78996295
129	18	GO Process	platelet degranulation	1.5E-06	VCL|APOA1|ACTN4|F13A1|ENDOD1|TLN1|CYB5R1|TAGLN2|FLNA|APOOL|CD9|ACTN1|PSAP|ALDOA|LAMP2|SERPINA1|WDR1|CD63	6.31E-08	GO.0002576	0.58356471
625	43	GO Process	carbohydrate derivative biosynthetic process	1.5E-06	DCN|NANS|GNPNAT1|OGDH|PDHX|ENO1|DHTKD1|ATP6V0A1|SORD|DLAT|CRYL1|HPRT1|DCXR|PDHB|ATP6V0A4|AQP1|GBAS|ACSS1|CYC1|SHMT1|UGDH|PKM|PKLR|PFKM|VCP|CMPK1|AKR1A1|PRPS1|OGDHL|HSPG2|ALDOB|APRT|PDHA1|BPGM|NME2|ALDOA|AMPD3|ATP5A1|TKT|GK|GPI|ACSL1|GALK1	6.63E-08	GO.1901137	0.58153086
67	13	GO Component	integral component of mitochondrial membrane	1.6E-06	FIS1|SYNJ2BP|CHCHD3|CPT1A|AFG3L2|SLC25A4|RHOT2|AGK|COA3|APOOL|IMMT|TOMM40|ETFDH	1.55E-07	GO.0032592	0.58096683
213	23	GO Process	response to insulin	1.7E-06	ATP6V1D|ATP6V1B1|GOT2|ATP6V1E1|GGH|KHK|ATP6V0A1|ATP6V1A|ATP6V1B2|ATP6V0D2|YWHAG|ATP6V0A4|HSD11B2|PCK1|PKM|ATP6V0C|PKLR|ATP6V1H|GOT1|ATP6V1G1|TBC1D4|APRT|HADH	7.42E-08	GO.0032868	0.57695511
59	9	KEGG Pathways	Glycerolipid metabolism	0.00034	ALDH2|ALDH3A2|ALDH9A1|AGK|AKR1A1|ALDH1B1|DAK|ALDH7A1|GK	6.93E-05	hsa00561	0.34685211
57	12	GO Component	azurophil granule membrane	2E-06	ARL8A|LAMTOR1|GAA|ATP6V0C|LAMP1|VAPA|CKAP4|PSAP|MGST1|LAMP2|TOM1|CD63	2.16E-07	GO.0035577	0.5692504
52	12	GO Process	glutathione metabolic process	2.1E-06	GSTM3|NAT8|CNDP2|GSTA1|GSTO1|DPEP1|HAGH|GGT5|GGT1|IDH1|GPX1|GSTA2	9.07E-08	GO.0006749	0.56840297
13	4	GO Component	mitochondrial respiratory chain complex III	0.0041	PMPCB|UQCRH|CYC1|UQCR10	0.00085	GO.0005750	0.23872161
39	17	KEGG Pathways	Glycine, serine and threonine metabolism	1E-12	AGXT2|DMGDH|BHMT|AOC3|GRHPR|PIPOX|SHMT1|CHDH|SHMT2|PHGDH|SARDH|PSAT1|MAOB|GLDC|BPGM|GATM|ALDH7A1	4E-14	hsa00260	1.2

1292	69	GO Process	ion transport	2.5E-06	CA12|ATP6V1D|SFXN3|SLC25A3|LTF|ATP6V1B1|APOA1|GOT2|PMPCB|ATP6V1E1|SLC4A1|SLC9A3R1|LRP2|ATP6V0A1|NNT|VDAC1|CPT1A|NDUFA9|SLC27A2|RHCG|AFG3L2|ATP6V1A|FTH1|ATP6V1B2|SLC25A4|CA2|ATP6V0D2|FABP1|SLC23A1|ATP6V0A4|AQP1|SFXN1|CYC1|GLS|SLC7A8|SLC5A2|ATP6V0C|RAB11B|BSG|CYB5A|ANXA6|ATP6V1H|OPA1|CYB5R1|ATP1B1|SFXN2|ABCD3|CPT2|SCP2|SLC25A5|ATP6V1G1|CLIC4|CLIC1|SLC3A2|SLC25A6|PSAP|ATP5A1|SLC25A12|SLC4A4|PDZK1|TOMM40|ACSL1|PHB2|ATP1A1|SLC25A10|NPC2|SLC43A2|PHB|PLCG2	1.08E-07	GO.0006811	0.56090649
6066	223	GO Function	ion binding	2.5E-06	RALA|GNA11|CA12|EPDR1|MYH9|ACO2|MTHFD1|PCK2|PYGB|MYL12A|IDH3G|F9|EHD4|OGDH|AGXT2|LTF|FAHD2A|ACADL|ATP6V1B1|ENO1|APOA1|GDA|PFN2|ACADS|PEPD|GOT2|CAPNS1|CANX|VIL1|PMPCB|ACTN4|HRSP12|APCS|BHMT2|ARL8B|GSTM3|NARS|KHK|RAB21|CDH1|RAB11A|DNAJA3|RAB2A|DHTKD1|BBOX1|PACSIN2|LRP2|RAB17|F13A1|ACAD11|RAB7A|ENPEP|CALB1|SORD|SLC27A2|IQGAP1|AFG3L2|BCKDHA|ARL8A|ATP6V1A|FTH1|BHMT|ATP6V1B2|DPYS|GLUD1|ENDOD1|QDPR|HGD|DDAH1|CA2|HPD|FTCD|CAPN2|RNPEP|FABP1|SCIN|HSPA9|CRYL1|HPRT1|CKB|TPP1|IDH3A|CDH16|ANPEP|PRODH2|ADH1B|CYCS|GPD2|ACO1|RAB1B|CSRP2|CYP4A11|RAB6A|AOC3|GRHPR|TLN1|ACSS1|CYC1|SHMT1|PCK1|CHDH|PKM|RHOT2|NDUFV1|CNDP2|EHD3|ACSM2B|C11orf54|IDH2|SERPINA5|RAB11B|SHMT2|TRIM2|PKLR|CYB5A|PPA2|PTGES2|PFKM|ANXA6|UGT1A9|WARS|AGK|COL18A1|PLS3|UBE2D3|VCP|MYH10|LONP1|PNP|OPA1|KIF21A|MARC2|CSRP1|NDUFS2|LHPP|ACADSB|MYO6|ABCD3|GOT1|ACADM|XPNPEP2|SCP2|CMPK1|ASS1|PRPS1|GSN|RAB14|OGDHL|HMGCL|HSPG2|ASPN|VARS|AGMAT|CUBN|MAOB|APRT|MT1M|NQO2|IDH3B|GLDC|GPNMB|FECH|PRKAR1A|DPEP1|NME2|PC|FHL1|ACTN1|PRKCD|DAK|PSAP|AK4|UMOD|MGST1|MYH11|AMPD3|ABAT|ILK|HAGH|ATP5A1|SEPT7|TXNRD2|ACY1|SLC25A12|IDH1|TKT|NDUFS1|ANXA11|CRYZ|RHOA|GK|ACSF2|DDC|CKMT2|CKMT1A|FBP1|LARS2|AOC1|MME|GFM1|ACSL1|NDUFA13|ETFDH|KIF13B|EPHX2|CAPN1|ME3|ATP1A1|MMAB|RAN|PAH|TWF1|ACSS3|ALDH6A1|ACSM2A|GALK1|HADH|DDX6|PDLIM5|VPS29|PSMC3|FMO1|HAO2	5.32E-08	GO.0043167	0.55985995
2163	101	GO Function	carbohydrate derivative binding	2.6E-06	RALA|DCN|GNA11|MYH9|MTHFD1|PCK2|IDH3G|EHD4|LTF|ACADL|ATP6V1B1|VIL1|ACTN4|ARL8B|NARS|KHK|RAB21|RAB11A|DNAJA3|RAB2A|RAB17|RAB7A|SLC27A2|AFG3L2|ARL8A|ATP6V1A|ATP6V1B2|GLUD1|PSMB4|HSPA9|CKB|RAB1B|RAB6A|ACSS1|PCK1|PKM|RHOT2|NDUFV1|EHD3|ACSM2B|SERPINA5|RAB11B|PKLR|CTSB|PFKM|ANXA6|WARS|AGK|UBE2D3|VCP|MYH10|LONP1|PNP|OPA1|KIF21A|MYO6|ABCD3|SCP2|CMPK1|ASS1|PRPS1|RAB14|HMGCL|VARS|APRT|GPNMB|PRKAR1A|DPEP1|NME2|PC|PRKCD|DAK|PSAP|AK4|MYH11|ILK|ATP5A1|SEPT7|RHOA|GK|ACSF2|CKMT2|CKMT1A|FBP1|LARS2|AOC1|GFM1|ACSL1|NDUFA13|KIF13B|ATP1A1|MMAB|RAN|TWF1|ACSS3|ALDH6A1|ACSM2A|GALK1|DDX6|PSMC3|HAO2	5.77E-08	GO.0097367	0.55816987
78	14	GO Process	protein homotetramerization	2.8E-06	HSD17B10|ACOT13|ACADL|DPYS|HPRT1|DCXR|GLS|SHMT1|PKM|SHMT2|GPX3|ALDOA|CRYZ|FBP1	1.21E-07	GO.0051289	0.55606673
1950	91	GO Component	bounding membrane of organelle	2.8E-06	SYPL1|GNPNAT1|ATP6V1D|EHD4|PLIN3|FIS1|ATP6V1B1|TFG|LGALS3|SYNJ2BP|ARL8B|RAB21|RAB11A|RAB2A|PACSIN2|NAPA|LRP2|GRB14|RAB17|ATP6V0A1|SCARB2|RAB7A|VDAC1|CPT1A|SLC27A2|IQGAP1|ARL8A|NAT8|LAMTOR1|ATP6V0D2|HPD|FTCD|AMN|ANPEP|TMED10|GAA|ATP6V0A4|RAB1B|RAB6A|GBAS|DAB2|RHOT2|EHD3|AP2A2|COPB2|ATP6V0C|TMED9|SERPINA5|LAMP1|RAB11B|BSG|CYB5A|LAMTOR4|PTGES2|VAPA|ALDH3A2|ANXA6|AGK|UBE2D3|ATP6V1H|OPA1|MARC2|CYB5R1|MYO6|ASS1|RAB14|ATP6V1G1|CUBN|CKAP4|MAOB|BPHL|CD9|SACM1L|CNP|PSAP|CD59|UMOD|MGST1|UBA52|RHOA|GK|LAMP2|TOMM40|TOM1|SERPINA1|MME|ACSL1|PHB2|MGAM|CD63|VPS29	3.12E-07	GO.0098588	0.55559552

311	27	GO Component	vacuolar membrane	3E-06	ATP6V1B1|ARL8B|NAPA|ATP6V0A1|RAB7A|ARL8A|LAMTOR1|ATP6V0D2|GAA|ATP6V0A4|DAB2|AP2A2|ATP6V0C|LAMP1|LAMTOR4|VAPA|ANXA6|ATP6V1H|MYO6|ATP6V1G1|CUBN|CKAP4|PSAP|MGST1|LAMP2|TOM1|CD63	3.36E-07	GO.0005774	0.5530178
181	20	GO Component	mitochondrial outer membrane	3.3E-06	FIS1|SYNJ2BP|VDAC1|CPT1A|GBAS|RHOT2|CYB5A|AGK|OPA1|MARC2|ASS1|MAOB|BPHL|CNP|MGST1|UBA52|GK|TOMM40|ACSL1|PHB2	3.77E-07	GO.0005741	0.54867824
485	36	GO Process	cellular response to organonitrogen compound	3.3E-06	ATP6V1D|FIS1|ATP6V1B1|ATP6V1E1|CDH1|ATP6V0A1|ATP6V1A|ATP6V1B2|LAMTOR1|CA2|ATP6V0D2|CAPN2|GPD1|YWHAG|ATP6V0A4|AQP1|HNRNPD|SHMT1|PCK1|PKM|ATP6V0C|PKLR|LAMTOR4|ATP6V1H|OPA1|COL6A1|GOT1|ASS1|ATP6V1G1|COL4A1|TBC1D4|APRT|PRKAR1A|PRKCD|DDC|AOC1	1.47E-07	GO.0071417	0.54814861
223	23	GO Process	mitochondrial transport	3.6E-06	FIS1|PPIF|SLC25A3|PMPCB|CPT1A|AFG3L2|SLC25A4|ACAA2|YWHAG|CYC1|RHOT2|AGK|OPA1|CPT2|SLC25A5|SLC25A6|CNP|ATP5A1|TST|SLC25A12|TOMM40|NDUFA13|SLC25A10	1.58E-07	GO.0006839	0.54497716
275	25	GO Component	endocytic vesicle	3.6E-06	LTF|APOA1|RAB11A|LRP2|RAB17|ATP6V0A1|RAB7A|ATP6V0D2|AMN|ATP6V0A4|EHD3|AP2A2|ATP6V0C|LAMP1|RAB11B|DPP4|MYO6|GSN|RAB14|CUBN|CD9|UBA52|ANXA11|LAMP2|FLNB	4.18E-07	GO.0030139	0.54473318
123	17	GO Process	purine nucleoside bisphosphate metabolic process	3.7E-06	OGDH|PDHX|ACOT13|DLAT|PDHB|ACSS1|PIPOX|ACSM2B|DLST|HMGCL|ACOT9|PDHA1|ACSF2|ABHD14B|ACSL1|ACSM2A|GLYAT	1.66E-07	GO.0034032	0.54317983
123	17	GO Process	ribonucleoside bisphosphate metabolic process	3.7E-06	OGDH|PDHX|ACOT13|DLAT|PDHB|ACSS1|PIPOX|ACSM2B|DLST|HMGCL|ACOT9|PDHA1|ACSF2|ABHD14B|ACSL1|ACSM2A|GLYAT	1.66E-07	GO.0033875	0.54317983
23	9	GO Function	proton-exporting ATPase activity, phosphorylative mechanism	4E-06	ATP6V1E1|ATP6V0A1|ATP6V1A|ATP6V1B2|ATP6V0D2|ATP6V0A4|ATP6V0C|ATP6V1H|ATP6V1G1	9.13E-08	GO.0008553	0.54001169
68	13	GO Process	protein targeting to peroxisome	4E-06	ECH1|EHHADH|SLC27A2|PIPOX|DHRS4|ACAA1|UBE2D3|SCP2|HMGCL|UBA52|IDH1|EPHX2|HAO2	1.8E-07	GO.0006625	0.54001169
26	9	GO Process	glycolytic process through glucose-6-phosphate	4.7E-06	ENO1|PKM|PKLR|PFKM|ALDOB|BPGM|ALDOA|GPI|GALK1	2.14E-07	GO.0061620	0.53316141
26	9	GO Process	cellular carbohydrate catabolic process	4.7E-06	PYGB|SORD|ABHD10|AGL|GAA|GPD2|PFKM|GK|MGAM	2.14E-07	GO.0044275	0.53316141
18	8	GO Process	branched-chain amino acid catabolic process	4.8E-06	HSD17B10|BCKDHA|BCAT2|HIBCH|ACADSB|HMGCL|AUH|ALDH6A1	2.22E-07	GO.0009083	0.53214816
830	51	GO Function	protein homodimerization activity	4.8E-06	MYH9|PSMD7|CRYM|DNPH1|ENO1|GOT2|VIL1|HSPB1|ACTN4|HRSP12|GSTM3|HEXB|SLC4A1|GRB14|ADD1|AMBP|QDPR|GPD1L|CRYL1|HPRT1|GPD1|AOC3|GRHPR|SHMT1|PFKM|ALDH3A2|ANXA6|UGT1A9|WARS|DPP4|LHPP|FLNA|ABCD3|HOGA1|PRPS1|HMGCL|CUBN|MAOB|NQO2|GLDC|ACTN1|PSAP|QPRT|MGST1|ABAT|ELAVL1|IDH1|TKT|AOC1|EPHX2|GALE	1.14E-07	GO.0042803	0.53196645
64	12	GO Component	proteasome complex	5.4E-06	PSMA4|TXNL1|PSMD7|HSPB1|PSMD11|PSMD3|PSMA5|PSMB4|VCP|PSMB9|PSMB8|PSMC3	6.43E-07	GO.0000502	0.52652002
431	33	GO Process	response to peptide	5.4E-06	ATP6V1D|HNRNPL|FIS1|ATP6V1B1|GOT2|ATP6V1E1|GGH|KHK|ATP6V0A1|IQGAP1|ATP6V1A|ATP6V1B2|QDPR|CA2|ATP6V0D2|YWHAG|ATP6V0A4|HSD11B2|CYC1|PCK1|PKM|ATP6V0C|BSG|PKLR|ATP6V1H|GOT1|ASS1|ATP6V1G1|TBC1D4|APRT|PRKAR1A|PRKCD|HADH	2.54E-07	GO.1901652	0.52644011

497	36	GO Process	peptide metabolic process	5.4E-06	CTSH|RPS16|GSTM3|NARS|ENPEP|NAT8|RNPEP|APEH|TPP1|ANPEP|RPS9|TSFM|CNDP2|GSTA1|PPA2|WARS|MRPL37|MRPL24|MRPL21|GSTO1|RPL5|VARS|DPEP1|HAGH|GGT5|GGT1|UBA52|IDH1|GPX1|LAMP2|LARS2|MRPS22|MME|GFM1|GSTA2|RPL18	2.55E-07	GO.0006518	0.52644011
68	22	KEGG Pathways	Glycolysis / Gluconeogenesis	4E-14	PCK2|ENO1|ALDH2|DLAT|ADH1B|PDHB|ACSS1|PCK1|PKM|PKLR|PFKM|ALDH3A2|ALDH9A1|AKR1A1|ALDOB|ALDH1B1|PDHA1|BPGM|ALDOA|ALDH7A1|GPI|FBP1	1.12E-15	hsa00010	1.339794
28	10	KEGG Pathways	Glyoxylate and dicarboxylate metabolism	3.7E-07	ACO2|ACO1|GRHPR|SHMT1|MDH2|SHMT2|CS|HOGA1|GLDC|HAO2	3.52E-08	hsa00630	0.64365189
806	49	GO Process	cellular homeostasis	5.9E-06	TXNL1|FIS1|LTF|HEXB|SLC4A1|SLC9A3R1|PRDX1|ATP6V0A1|NNT|ADD1|RAB7A|CALB1|PRDX5|RHCG|AFG3L2|ATP6V1A|FTH1|CA2|ATP6V0D2|CFL2|CKB|GAA|ATP6V0A4|ACO1|AQP1|RHOT2|ATP6V0C|RAB11B|PTGES2|PFKM|ANXA6|ATP6V1H|OPA1|ATP1B1|GSTO1|GOT1|ATP6V1G1|CLIC4|TXNDC5|ALDOA|TXNRD2|PDIA6|IMMT|SLC4A4|GPX1|LAMP2|EPHX2|ATP1A1|PLCG2	2.77E-07	GO.0019725	0.52313619
187	21	GO Function	carboxylic acid binding	6.1E-06	GOT2|HRSP12|DPYS|GLUD1|DDAH1|FTCD|FABP1|GRHPR|SHMT1|PCK1|SERPINA5|SHMT2|UGT1A9|GOT1|SCP2|ASS1|HMGCL|GLDC|PC|PSAP|DDC	1.51E-07	GO.0031406	0.52146702
179	20	GO Process	ammonium ion metabolic process	6.8E-06	ACADL|AKR7A2|APOA1|PLBD1|DMGDH|BBOX1|CPT1A|BHMT|HNMT|HPRT1|SHMT1|CHDH|ALDH9A1|ACADM|SARDH|AGMAT|MAOB|AKR1C3|ALDH7A1|DDC	3.2E-07	GO.0097164	0.51694113
1868	89	GO Function	ribonucleotide binding	6.9E-06	RALA|GNA11|MYH9|MTHFD1|PCK2|IDH3G|EHD4|ACADL|ATP6V1B1|ARL8B|NARS|KHK|RAB21|RAB11A|DNAJA3|RAB2A|RAB17|RAB7A|SLC27A2|AFG3L2|ARL8A|ATP6V1A|ATP6V1B2|GLUD1|HSPA9|CKB|RAB1B|RAB6A|ACSS1|PCK1|PKM|RHOT2|NDUFV1|EHD3|ACSM2B|RAB11B|PKLR|PFKM|ANXA6|WARS|AGK|UBE2D3|VCP|MYH10|LONP1|OPA1|KIF21A|MYO6|ABCD3|SCP2|CMPK1|ASS1|PRPS1|RAB14|HMGCL|VARS|APRT|PRKAR1A|NME2|PC|PRKCD|DAK|AK4|MYH11|ILK|ATP5A1|SEPT7|RHOA|GK|ACSF2|CKMT2|CKMT1A|FBP1|LARS2|GFM1|ACSL1|NDUFA13|KIF13B|ATP1A1|MMAB|RAN|TWF1|ACSS3|ALDH6A1|ACSM2A|GALK1|DDX6|PSMC3|HAO2	1.82E-07	GO.0032553	0.51592668
45	11	GO Function	NADH dehydrogenase (ubiquinone) activity	7E-06	NDUFB4|NDUFA2|NDUFA10|NDUFA9|NDUFB9|NDUFB8|NDUFV1|NDUFA12|NDUFS2|NDUFS1|NDUFA13	1.9E-07	GO.0008137	0.51580152
394	31	GO Process	nucleobase-containing compound catabolic process	7.2E-06	AHCY|OGDH|DNPH1|ENO1|GDA|RPS16|DHTKD1|DPYS|HPRT1|RPS9|HINT1|HNRNPD|PKM|PKLR|PFKM|SND1|VCP|PNP|RPL5|OGDHL|ALDOB|BPGM|CNP|ALDOA|AMPD3|UBA52|GPI|GPX1|RPL18|GALK1|DDX6	3.41E-07	GO.0034655	0.51438756
396	31	GO Process	establishment of protein localization to organelle	7.9E-06	ECH1|FIS1|EHHADH|PMPCB|RPS16|RAB11A|SCARB2|RAB7A|SLC27A2|RPS9|PIPOX|DHRS4|LAMP1|ACAA1|AGK|UBE2D3|RPL5|SCP2|HMGCL|AKR1C3|SLC25A6|UBA52|IDH1|LAMP2|TOMM40|NDUFA13|EPHX2|PHB2|RAN|RPL18|HAO2	3.78E-07	GO.0072594	0.51012748
148	18	GO Process	fatty acid derivative metabolic process	8.4E-06	CBR1|CYP4A11|CES2|PTGES2|MGST3|HMGCL|AKR1C3|BDH1|DPEP1|GGT5|GGT1|PTGR1|GPX1|ACSL1|EPHX2|ACSS3|PTGR2|ACSM2A	4.04E-07	GO.1901568	0.50741724
995	56	GO Process	chemical homeostasis	8.4E-06	DCN|CA12|CTSH|FIS1|SFXN3|LTF|ATP6V1B1|APOA1|NAPSA|HEXB|RAB11A|SLC4A1|SLC9A3R1|ATP6V0A1|NNT|RAB7A|CALB1|RHCG|AFG3L2|ATP6V1A|FTH1|LAMTOR1|GPD1L|CA2|ATP6V0D2|CKB|ATP6V0A4|ACO1|CYP4A11|AQP1|SFXN1|PCK1|SLC7A8|ATP6V0C|RAB11B|PFKM|ANXA6|ATP6V1H|OPA1|ATP1B1|GSTO1|SFXN2|GOT1|RBP4|ATP6V1G1|CLIC4|PRKAR1A|FHL1|UMOD|IMMT|SLC4A4|EPHX2|ATP1A1|NPC2|ACSM2A|PLCG2	4.04E-07	GO.0048878	0.50741724
23	10	KEGG Pathways	Histidine metabolism	8.9E-08	ALDH2|HNMT|FTCD|CNDP2|ALDH3A2|ALDH9A1|ALDH1B1|MAOB|ALDH7A1|AOC1	7.75E-09	hsa00340	0.70520764

27	9	GO Function	oxidoreductase activity, acting on the CH-NH group of donors	9.5E-06	MTHFD1|CRYM|DMGDH|QDPR|PRODH2|PIPOX|SARDH|ALDH4A1|ETFDH	2.79E-07	GO.0016645	0.50218195
29	9	GO Process	energy coupled proton transmembrane transport, against electrochemical gradient	9.5E-06	ATP6V1B1|ATP6V1E1|ATP6V0A1|ATP6V1A|ATP6V1B2|ATP6V0D2|ATP6V0A4|ATP6V0C|ATP6V1H	4.61E-07	GO.0015988	0.50204516
7	6	GO Process	amino-acid betaine biosynthetic process	9.7E-06	BBOX1|SHMT1|CHDH|ALDH9A1|ACADM|ALDH7A1	4.69E-07	GO.0006578	0.50145735
39	10	GO Process	sulfur amino acid metabolic process	9.7E-06	MTHFD1|AHCY|BHMT2|BHMT|MSRA|AHCYL2|DPEP1|MPST|GGT1|TST	4.72E-07	GO.0000096	0.50141246
59	12	GO Function	GDP binding	9.7E-06	RALA|ARL8B|RAB21|RAB2A|RAB17|RAB7A|PCK1|RAB11B|PRPS1|RAB14|RHOA|RAN	2.99E-07	GO.0019003	0.50136762
1865	88	GO Function	purine nucleotide binding	1E-05	RALA|GNA11|MYH9|MTHFD1|PCK2|AHCY|IDH3G|EHD4|ACADL|ATP6V1B1|ARL8B|NARS|KHK|RAB21|RAB11A|DNAJA3|RAB2A|RAB17|RAB7A|SLC27A2|AFG3L2|ARL8A|ATP6V1A|ATP6V1B2|GLUD1|HSPA9|CKB|RAB1B|RAB6A|ACSS1|PCK1|PKM|RHOT2|EHD3|ACSM2B|RAB11B|PKLR|PFKM|ANXA6|WARS|AGK|UBE2D3|VCP|MYH10|LONP1|OPA1|KIF21A|MYO6|ABCD3|SCP2|CMPK1|ASS1|PRPS1|RAB14|HMGCL|VARS|APRT|PRKAR1A|NME2|PC|PRKCD|DAK|AK4|MYH11|ILK|ATP5A1|SEPT7|RHOA|GK|ACSF2|CKMT2|CKMT1A|FBP1|LARS2|GFM1|ACSL1|NDUFA13|KIF13B|ATP1A1|MMAB|RAN|TWF1|ACSS3|ALDH6A1|ACSM2A|GALK1|DDX6|PSMC3	3.24E-07	GO.0017076	0.49913998
729	45	GO Process	membrane organization	1.2E-05	RALA|MYH9|FIS1|PPIF|TFG|RAB11A|DNAJA3|CHCHD3|PACSIN2|NAPA|LRP2|SCARB2|RAB7A|AFG3L2|SLC25A4|ACAA2|TMED10|YWHAG|RAB1B|DAB2|RHOT2|AP2A2|TMED9|SERPINA5|EPB41L3|VAPA|AGK|MYH10|OPA1|ATP1B1|SLC25A5|APOOL|GSN|SLC25A6|CD9|CNP|PRKCD|CD59|ATP5A1|IMMT|UBA52|RHOA|SERPINA1|PPIA|NDUFA13	5.87E-07	GO.0061024	0.4924453
61	12	GO Function	cation-transporting ATPase activity	1.3E-05	ATP6V1E1|ATP6V0A1|ATP6V1A|ATP6V1B2|ATP6V0D2|ATP6V0A4|ATP6V0C|ATP6V1H|ATP1B1|ATP6V1G1|ATP5A1|ATP1A1	4.09E-07	GO.0019829	0.490309
153	18	GO Process	cellular response to insulin stimulus	1.3E-05	ATP6V1D|ATP6V1B1|ATP6V1E1|ATP6V0A1|ATP6V1A|ATP6V1B2|ATP6V0D2|YWHAG|ATP6V0A4|PCK1|PKM|ATP6V0C|PKLR|ATP6V1H|GOT1|ATP6V1G1|TBC1D4|APRT	6.29E-07	GO.0032869	0.48961963
1853	87	GO Function	purine ribonucleotide binding	1.4E-05	RALA|GNA11|MYH9|MTHFD1|PCK2|IDH3G|EHD4|ACADL|ATP6V1B1|ARL8B|NARS|KHK|RAB21|RAB11A|DNAJA3|RAB2A|RAB17|RAB7A|SLC27A2|AFG3L2|ARL8A|ATP6V1A|ATP6V1B2|GLUD1|HSPA9|CKB|RAB1B|RAB6A|ACSS1|PCK1|PKM|RHOT2|EHD3|ACSM2B|RAB11B|PKLR|PFKM|ANXA6|WARS|AGK|UBE2D3|VCP|MYH10|LONP1|OPA1|KIF21A|MYO6|ABCD3|SCP2|CMPK1|ASS1|PRPS1|RAB14|HMGCL|VARS|APRT|PRKAR1A|NME2|PC|PRKCD|DAK|AK4|MYH11|ILK|ATP5A1|SEPT7|RHOA|GK|ACSF2|CKMT2|CKMT1A|FBP1|LARS2|GFM1|ACSL1|NDUFA13|KIF13B|ATP1A1|MMAB|RAN|TWF1|ACSS3|ALDH6A1|ACSM2A|GALK1|DDX6|PSMC3	4.68E-07	GO.0032555	0.4853872
65	12	GO Process	mitochondrial respiratory chain complex I assembly	1.5E-05	NDUFB4|NDUFA2|NDUFA10|NDUFA9|ECSIT|NDUFB9|NDUFB8|NDUFV1|NDUFA12|NDUFS2|NDUFS1|NDUFA13	7.44E-07	GO.0032981	0.48239087

1047	56	GO Component	membrane protein complex	1.5E-05	GNA11|NDUFB4|ATP6V1D|PPIF|ATP6V1B1|PMPCB|NDUFA2|NDUFA10|ATP6V1E1|CDH1|CHCHD3|NAPA|ATP6V0A1|VDAC1|NDUFA9|AFG3L2|ATP6V1A|ATP6V1B2|NDUFB9|LAMTOR1|ATP6V0D2|NDUFB8|TMED10|ATP6V0A4|UQCRH|TOLLIP|CYC1|NDUFV1|AP2A2|COPB2|ATP6V0C|NDUFA12|UQCR10|LAMTOR4|AGK|VCP|ATP6V1H|KRT19|ATP1B1|NDUFS2|APOOL|ATP6V1G1|CLIC4|HSPG2|CLIC1|SLC25A6|SACM1L|ATP5A1|IMMT|NDUFS1|LAMP2|TOMM40|NDUFA13|ATP1A1|KRT8|VPS29	1.83E-06	GO.0098796	0.48210231
568	38	GO Process	cellular response to nitrogen compound	1.5E-05	GNA11|ATP6V1D|FIS1|ATP6V1B1|ATP6V1E1|CDH1|ATP6V0A1|ATP6V1A|ATP6V1B2|LAMTOR1|CA2|ATP6V0D2|CAPN2|GPD1|YWHAG|ATP6V0A4|AQP1|HNRNPD|SHMT1|PCK1|PKM|ATP6V0C|PKLR|LAMTOR4|ATP6V1H|OPA1|COL6A1|GOT1|ASS1|ATP6V1G1|COL4A1|TBC1D4|APRT|PRKAR1A|DPEP1|PRKCD|DDC|AOC1	7.53E-07	GO.1901699	0.48210231
21	8	GO Function	proton-transporting ATPase activity, rotational mechanism	1.6E-05	ATP6V1E1|ATP6V0A1|ATP6V1A|ATP6V1B2|ATP6V0D2|ATP6V0A4|ATP6V0C|ATP6V1H	5.69E-07	GO.0046961	0.48041003
30	9	GO Function	ATPase activity, coupled to transmembrane movement of ions, rotational mechanism	1.6E-05	ATP6V1E1|ATP6V0A1|ATP6V1A|ATP6V1B2|ATP6V0D2|ATP6V0A4|ATP6V0C|ATP6V1H|ATP5A1	5.86E-07	GO.0044769	0.48013429
123	16	GO Process	mitochondrial membrane organization	1.6E-05	PPIF|CHCHD3|AFG3L2|SLC25A4|ACAA2|YWHAG|RHOT2|AGK|OPA1|SLC25A5|APOOL|SLC25A6|CNP|ATP5A1|IMMT|NDUFA13	7.95E-07	GO.0007006	0.47986029
882	51	GO Function	cytoskeletal protein binding	1.7E-05	RALA|VCL|MYH9|PFN2|VIL1|ACTN4|ARL8B|CDH1|RAB11A|SLC4A1|COTL1|SLC9A3R1|PACSIN2|CAPG|ADD1|RHCG|ARL8A|FTCD|CAPN2|SCIN|CFL2|DPYSL2|RAB6A|TLN1|RAB11B|PARVA|EPB41L3|VAPA|PLS3|MSN|MYH10|OPA1|KIF21A|FLNA|MYO6|GSN|RAB14|ALDOB|ACTN1|ALDOA|KTN1|MYH11|RHOA|FLNB|WDR1|KIF13B|TAGLN|ATP1A1|TWF1|EML2|PDLIM5	6.51E-07	GO.0008092	0.47644716
87	13	GO Component	peptidase complex	1.7E-05	PSMA4|TXNL1|PSMD7|HSPB1|PSMD11|PSMD3|AFG3L2|PSMA5|PSMB4|VCP|PSMB9|PSMB8|PSMC3	2.17E-06	GO.1905368	0.47594508
157	18	GO Process	regulation of macroautophagy	1.8E-05	DCN|ATP6V1D|CDC37|ATP6V1B1|CAPNS1|ATP6V1E1|ATP6V0A1|ATP6V1A|ATP6V1B2|LAMTOR1|UCHL1|ATP6V0D2|RAB1B|ATP6V0C|LAMTOR4|ATP6V1H|ATP6V1G1|CAPN1	8.84E-07	GO.0016241	0.47544873

<table>
<tr><td>6607</td><td>233</td><td>GO Function</td><td>protein binding</td><td>1.8E-05</td><td>RALA|DCN|GNA11|VCL|MYH9|GNPNAT1|AHCY|PSMD7|CRYM|ECH1|CDC37|OGDH|FIS1|DNPH1|AGXT2|EHHADH|ENO1|APOA1|TTR|PFN2|TFG|GOT2|CANX|VIL1|HSPB1|ACTN4|LAMA5|ATP6V1E1|LGALS3|HRSP12|APCS|SYNJ2BP|ARL8B|GSTM3|LYZ|HEXB|CDH1|RAB11A|DNAJA3|SLC4A1|COTL1|CHCHD3|SLC9A3R1|PRDX1|BBOX1|PACSIN2|NAPA|EPCAM|LRP2|CAPG|GRB14|ATP6V0A1|ADD1|SCARB2|RAB7A|AMBP|VDAC1|PRDX5|CPT1A|SORD|SLC27A2|RHCG|IQGAP1|AFG3L2|BCAM|TINAGL1|ARL8A|ERLIN2|DPYS|GLUD1|LAMTOR1|DLAT|QDPR|GPD1L|HGD|UCHL1|FTCD|AGL|CAPN2|APEH|SCIN|HSPA9|COL14A1|CFL2|CRYL1|HPRT1|AMN|CKB|GPD1|GUSB|TMED10|DCXR|HINT1|GNB2|YWHAG|ASL|ATP6V0A4|DPYSL2|AQP1|RAB6A|AOC3|HNRNPD|DAB2|GRHPR|TOLLIP|TLN1|PIPOX|SHMT1|PKM|HNRNPM|DHRS4|MDH2|AP2A2|ATP6V0C|TMED9|SERPINA5|LAMP1|RAB11B|SHMT2|PARVA|CYB5A|EPB41L3|VAPA|CTSB|PFKM|ALDH3A2|ANXA6|UGT1A9|WARS|COL18A1|PLS3|PBLD|VCP|MSN|MYH10|DPP4|LONP1|OPA1|KIF21A|COL6A1|AKR7A3|ATP1B1|NDUFS2|LHPP|FLNA|MYO6|ABCD3|RPL5|HOGA1|ACADM|RBP4|SCP2|SLC25A5|ASS1|PRPS1|GSN|RAB14|ATP6V1G1|HMGCL|HSPG2|ALDOB|ALDH4A1|COL4A1|KCTD12|TBC1D4|HIST1H2AC|CUBN|MAOB|NQO2|NAP1L4|GLDC|GPNMB|CD9|GPX3|KRT18|PRKAR1A|PC|FHL1|ACTN1|PRKCD|PSAP|ALDOA|KTN1|QPRT|CD59|MGST1|MYH11|ABAT|ILK|MPST|ATP5A1|SEPT7|ACY1|ELAVL1|SLC25A12|IDH1|TKT|SLC4A4|GDI1|PDZK1|ANXA11|RHOA|DDC|GPI|GPX1|FBP1|LAMP2|AOC1|TOM1|SERPINA1|TGFBI|CFB|VWA1|PPIA|FLNB|WDR1|KIF13B|EPHX2|TAGLN|PHB2|ATP1A1|RAN|KRT8|TWF1|NPC2|EML2|DDX6|PHB|PDLIM5|PSMC3|PLCG2|GALE|HAO2</td><td>7.03E-07</td><td>GO.0005515</td><td>0.47423214</td></tr>
<tr><td>193</td><td>23</td><td>KEGG Pathways</td><td>Huntington's disease</td><td>1.5E-07</td><td>NDUFB4|PPIF|NDUFA2|NDUFA10|VDAC1|NDUFA9|NDUFB9|SLC25A4|NDUFB8|CYCS|UQCRH|CYC1|NDUFV1|AP2A2|NDUFA12|UQCR10|NDUFS2|SLC25A5|SLC25A6|ATP5A1|NDUFS1|GPX1|NDUFA13</td><td>1.42E-08</td><td>hsa05016</td><td>0.68124793</td></tr>
<tr><td>59</td><td>13</td><td>KEGG Pathways</td><td>Lysine degradation</td><td>4.1E-07</td><td>OGDH|EHHADH|ALDH2|BBOX1|PIPOX|DLST|ALDH3A2|ALDH9A1|ECHS1|OGDHL|ALDH1B1|ALDH7A1|HADH</td><td>4.23E-08</td><td>hsa00310</td><td>0.6391474</td></tr>
<tr><td>8</td><td>6</td><td>GO Function</td><td>oxidoreductase activity, acting on the aldehyde or oxo group of donors, disulfide as acceptor</td><td>2E-05</td><td>OGDH|DHTKD1|BCKDHA|PDHB|OGDHL|PDHA1</td><td>8.03E-07</td><td>GO.0016624</td><td>0.46946486</td></tr>
<tr><td>8</td><td>6</td><td>GO Function</td><td>enoyl-CoA hydratase activity</td><td>2E-05</td><td>EHHADH|ECI1|ECHS1|ECHDC2|AUH|ECHDC1</td><td>8.03E-07</td><td>GO.0004300</td><td>0.46946486</td></tr>
<tr><td>61</td><td>11</td><td>GO Component</td><td>ficolin-1-rich granule membrane</td><td>2.1E-05</td><td>LGALS3|ATP6V0A1|ARL8A|LAMTOR1|GAA|AP2A2|ATP6V0C|LAMP1|RHOA|LAMP2|MGAM</td><td>2.64E-06</td><td>GO.0101003</td><td>0.46840297</td></tr>
<tr><td>1353</td><td>68</td><td>GO Process</td><td>response to endogenous stimulus</td><td>2.2E-05</td><td>GNA11|ATP6V1D|CTSH|FIS1|ATP6V1B1|GOT2|VIL1|ATP6V1E1|GGH|KHK|CDH1|ATP6V0A1|SORD|IQGAP1|ATP6V1A|ATP6V1B2|LAMTOR1|QDPR|CA2|ATP6V0D2|AGL|CAPN2|GPD1|YWHAG|ATP6V0A4|AQP1|HNRNPD|HSD11B2|CYC1|SHMT1|PCK1|PKM|HNRNPM|ATP6V0C|BSG|PKLR|LAMTOR4|CTSB|UBE2D3|ATP6V1H|MSN|COL4A2|LONP1|OPA1|COL6A1|GOT1|ASS1|RAB14|ATP6V1G1|COL4A1|TBC1D4|MAOB|APRT|AKR1C3|CD9|FECH|PRKAR1A|PRKCD|UBA52|IDH1|RHOA|DDC|AOC1|ACSL1|ATP1A1|RAN|HADH|PHB</td><td>1.1E-06</td><td>GO.0009719</td><td>0.46615435</td></tr>
<tr><td>143</td><td>17</td><td>GO Process</td><td>glycosyl compound metabolic process</td><td>2.2E-05</td><td>AHCY|AKR7A2|ABHD10|DPYS|HPRT1|AHCYL2|PNP|OPA1|CMPK1|PRPS1|APRT|AKR1C3|NME2|AK4|AMPD3|RHOA|RAN</td><td>1.13E-06</td><td>GO.1901657</td><td>0.4653647</td></tr>
<tr><td>650</td><td>41</td><td>GO Process</td><td>symbiont process</td><td>2.2E-05</td><td>RALA|PSMA4|LTF|APCS|CDH1|SCARB2|RAB7A|AMBP|VDAC1|SLC25A4|PSMB4|ANPEP|RAB1B|AQP1|RAB6A|TLN1|AP2A2|KRT7|ATP6V0C|LAMP1|VAPA|CTSB|SND1|VCP|ATP6V1H|DPP4|KRT19|SLC25A5|PSMB9|PSMB8|SLC25A6|KRT18|PC|UBA52|RHOA|GPX1|PPIA|RAN|KRT8|DDX6|PSMC3</td><td>1.14E-06</td><td>GO.0044403</td><td>0.46516951</td></tr>
</table>

96	14	GO Process	detoxification	2.2E-05	TXNL1|GSTM3|PRDX1|NNT|PRDX5|FABP1|GSTA1|MARC2|MGST3|GSTO1|GPX3|MGST1|TXNRD2|GPX1	1.15E-06	GO.0098754	0.4649752
900	51	GO Process	response to drug	2.2E-05	GNA11|PPIF|APOA1|GOT2|GGH|CDH1|LRP2|CPT1A|SORD|NAT8|QDPR|SLC25A4|CA2|FABP1|HPRT1|AMN|AQP1|HNRNPD|HSD11B2|PKLR|APOD|COL18A1|PNP|OPA1|MYO6|ABCD3|RBP4|SLC25A5|ASS1|GSN|CUBN|MAOB|SLC25A6|FECH|DPEP1|PRKCD|AK4|MGST1|ABAT|PTGR1|SLC25A12|PDZK1|RHOA|DDC|GPX1|FBP1|AOC1|ACSL1|ATP1A1|SLC25A10|HADH	1.14E-06	GO.0042493	0.4649752
144	17	GO Process	cellular carbohydrate metabolic process	2.4E-05	PYGB|KHK|SORD|ABHD10|AGL|GAA|GPD2|PCK1|IDH2|PFKM|GOT1|DAK|IDH1|GK|FBP1|MGAM|GALK1	1.23E-06	GO.0044262	0.46216021
512	35	GO Process	protein complex oligomerization	2.5E-05	HSD17B10|EHD4|FIS1|ACOT13|ACADL|CPT1A|DPYS|HPRT1|COL6A2|TMED10|DCXR|GRHPR|GLS|SHMT1|PKM|DHRS4|EHD3|SHMT2|PFKM|ANXA6|VCP|LONP1|OPA1|COL6A1|HMGCL|KCTD12|GPX3|ALDOA|QPRT|MGST1|ILK|SEPT7|ELAVL1|CRYZ|FBP1	1.32E-06	GO.0051259	0.45985995
69	12	GO Process	primary alcohol metabolic process	2.5E-05	AKR7A2|TTR|ALDH2|GPD1|ADH1B|GPD2|ACSS1|DHRS4|ALDH3A2|RBP4|ALDH1B1|AKR1C3	1.3E-06	GO.0034308	0.45985995
24	8	GO Process	glucuronate metabolic process	2.5E-05	SORD|ABHD10|CRYL1|DCXR|UGT2B7|UGT2B17|UGT1A9|AKR1A1	1.3E-06	GO.0019585	0.45985995
854	49	GO Process	response to hormone	2.6E-05	GNA11|ATP6V1D|CTSH|ATP6V1B1|GOT2|ATP6V1E1|GGH|KHK|ATP6V0A1|SORD|IQGAP1|ATP6V1A|ATP6V1B2|QDPR|CA2|ATP6V0D2|AGL|YWHAG|ATP6V0A4|AQP1|HNRNPD|HSD11B2|CYC1|PCK1|PKM|ATP6V0C|BSG|PKLR|CTSB|ATP6V1H|MSN|LONP1|GOT1|ASS1|ATP6V1G1|TBC1D4|MAOB|APRT|AKR1C3|FECH|PRKAR1A|PRKCD|IDH1|RHOA|ACSL1|ATP1A1|RAN|HADH|PHB	1.35E-06	GO.0009725	0.45900669
55	11	GO Function	hydro-lyase activity	2.6E-05	CA12|ACO2|EHHADH|ENO1|CA2|ECI1|ACO1|ECHS1|ECHDC2|AUH|ECHDC1	1.08E-06	GO.0016836	0.45833595
16	7	GO Process	glycine metabolic process	2.7E-05	AGXT2|HRSP12|SHMT1|SHMT2|PHGDH|GLDC|GLYAT	1.41E-06	GO.0006544	0.45718652
33	9	GO Function	ADP binding	2.7E-05	MYH9|GLUD1|PKM|VCP|MYH10|LONP1|MYO6|PRPS1|ATP1A1	1.15E-06	GO.0043531	0.45670307
98	14	GO Process	mitochondrial respiratory chain complex assembly	2.7E-05	NDUFB4|NDUFA2|NDUFA10|NDUFA9|ECSIT|NDUFB9|NDUFB8|NDUFV1|NDUFA12|UQCR10|COA3|NDUFS2|NDUFS1|NDUFA13	1.43E-06	GO.0033108	0.45670307
123	21	KEGG Pathways	Lysosome	3.2E-09	CTSH|CTSD|NAPSA|HEXB|ATP6V0A1|SCARB2|ATP6V0D2|TPP1|GUSB|GAA|ATP6V0A4|ATP6V0C|LAMP1|CTSB|GM2A|ATP6V1H|ASAH1|PSAP|LAMP2|CD63|NPC2	1.95E-10	hsa04142	0.8489455
92	13	GO Component	azurophil granule lumen	2.9E-05	PYGB|TTR|GGH|LYZ|HEXB|GUSB|TOLLIP|PTGES2|GM2A|VCP|TXNDC5|PRKCD|NPC2	3.78E-06	GO.0035578	0.45346171
252	22	GO Component	lysosomal membrane	2.9E-05	ARL8B|ATP6V0A1|RAB7A|ARL8A|LAMTOR1|ATP6V0D2|GAA|DAB2|AP2A2|ATP6V0C|LAMP1|LAMTOR4|VAPA|ANXA6|MYO6|CUBN|CKAP4|PSAP|MGST1|LAMP2|TOM1|CD63	3.79E-06	GO.0005765	0.45346171

<table>
<tr><th></th><th></th><th></th><th></th><th></th><th></th><th></th><th></th><th></th></tr>
<tr><td>1250</td><td>181</td><td>KEGG Pathways</td><td>Metabolic pathways</td><td>1.2E-74</td><td>HSD17B10|NDUFB4|NANS|ACO2|ATP6V1D|MTHFD1|PCK2|PYGB|AHCY|IDH3G|OGDH|PDHX|AGXT2|EHHADH|ACADL|ATP6V1B1|ENO1|GDA|ACADS|GOT2|NDUFA2|NDUFA10|LAMA5|ATP6V1E1|DMGDH|BHMT2|KHK|HEXB|ALDH2|MECR|ATP6V0A1|NNT|NDUFA9|SORD|BCKDHA|ATP6V1A|BHMT|ATP6V1B2|DPYS|NDUFB9|GLUD1|DLAT|QDPR|HGD|ACAA2|ATP6V0D2|HPD|CBR1|FTCD|AGL|CMBL|CRYL1|HPRT1|NDUFB8|CKB|IDH3A|ANPEP|PRODH2|GUSB|DCXR|UGT2B7|GAA|ADH1B|ASL|PDHB|CYCS|ATP6V0A4|ACO1|UQCRH|CYP4A11|AOC3|GRHPR|AHCYL2|ACSS1|CYC1|GLS|PIPOX|SHMT1|UGDH|PCK1|CHDH|PKM|UGT2B17|TALDO1|NDUFV1|BCAT2|CNDP2|DHRS4|MDH2|ACSM2B|ATP6V0C|NDUFA12|IDH2|UQCR10|ACAA1|SHMT2|DLST|PKLR|CS|PTGES2|PFKM|ALDH3A2|UGT1A9|ALDH9A1|AGK|ATP6V1H|HIBCH|PNP|NDUFS2|ECHS1|ACADSB|PHGDH|GOT1|HOGA1|ACADM|SCP2|SARDH|AKR1A1|ASS1|PRPS1|ATP6V1G1|OGDHL|HMGCL|ALDOB|ALDH4A1|AUH|AGMAT|PSAT1|ALDH1B1|MAOB|APRT|PDHA1|AKR1C3|IDH3B|GLDC|ASAH1|FECH|SACM1L|BDH1|BPGM|NME2|PC|DAK|ALDOA|AK4|QPRT|AMPD3|ABAT|GATM|MPST|GGT5|ATP5A1|GGT1|ACY1|TST|ALDH7A1|IDH1|TKT|NDUFS1|GK|DDC|CKMT2|GPI|CKMT1A|FBP1|AOC1|ACSL1|NDUFA13|EPHX2|ME3|MMAB|MGAM|PAH|ACSS3|ALDH6A1|ACSM2A|GALK1|HADH|PLCG2|GALE|HAO2</td><td>4.83E-77</td><td>hsa01100</td><td>7.39172146</td></tr>
<tr><td>25</td><td>8</td><td>GO Process</td><td>canonical glycolysis</td><td>3.2E-05</td><td>ENO1|PKM|PKLR|PFKM|ALDOB|BPGM|ALDOA|GPI</td><td>1.68E-06</td><td>GO.0061621</td><td>0.45003129</td></tr>
<tr><td>25</td><td>8</td><td>GO Process</td><td>NADH regeneration</td><td>3.2E-05</td><td>ENO1|PKM|PKLR|PFKM|ALDOB|BPGM|ALDOA|GPI</td><td>1.68E-06</td><td>GO.0006735</td><td>0.45003129</td></tr>
<tr><td>1770</td><td>82</td><td>GO Process</td><td>protein-containing complex subunit organization</td><td>3.3E-05</td><td>HSD17B10|NDUFB4|EHD4|FIS1|ACOT13|ACADL|APOA1|TFG|VIL1|NDUFA2|NDUFA10|APCS|PSMD11|SLC9A3R1|NAPA|CAPG|ATP6V0A1|CPT1A|NDUFA9|ECSIT|DPYS|NDUFB9|APEH|COL14A1|CFL2|HPRT1|NDUFB8|COL6A2|TMED10|DCXR|ATP6V0A4|RAB1B|GRHPR|TLN1|GLS|SHMT1|PKM|NDUFV1|DHRS4|EHD3|NDUFA12|UQCR10|SHMT2|PFKM|ANXA6|VCP|LONP1|MRPL37|MRPL24|MRPL21|OPA1|COA3|COL6A1|DPT|NDUFS2|RPL5|GSN|HMGCL|ALDOB|KCTD12|NAP1L4|GPX3|ALDOA|QPRT|CD59|MGST1|MYH11|ILK|SEPT7|ELAVL1|UBA52|NDUFS1|CRYZ|FBP1|LAMP2|SERPINA1|MRPS22|TMEM33|NDUFA13|WDR1|CIRBP|DDX6</td><td>1.76E-06</td><td>GO.0043933</td><td>0.44828041</td></tr>
<tr><td>381</td><td>29</td><td>GO Function</td><td>nucleoside binding</td><td>3.5E-05</td><td>RALA|GNA11|PCK2|EHD4|ACTN4|ARL8B|RAB21|RAB11A|RAB2A|RAB17|RAB7A|ARL8A|GLUD1|RAB1B|RAB6A|PCK1|RHOT2|EHD3|RAB11B|ANXA6|PNP|OPA1|PRPS1|RAB14|AK4|SEPT7|RHOA|GFM1|RAN</td><td>1.52E-06</td><td>GO.0001882</td><td>0.44522253</td></tr>
<tr><td>17</td><td>7</td><td>GO Process</td><td>serine family amino acid biosynthetic process</td><td>3.6E-05</td><td>MTHFD1|AGXT2|SHMT1|SHMT2|PHGDH|PSAT1|GGT1</td><td>1.95E-06</td><td>GO.0009070</td><td>0.44412914</td></tr>
<tr><td>1710</td><td>80</td><td>GO Function</td><td>drug binding</td><td>3.8E-05</td><td>MYH9|MTHFD1|PYGB|IDH3G|EHD4|PPIF|AGXT2|ATP6V1B1|GOT2|NARS|KHK|DNAJA3|LRP2|SLC27A2|AFG3L2|ATP6V1A|ATP6V1B2|DPYS|GLUD1|FTCD|FABP1|HSPA9|CKB|ACSS1|SHMT1|PKM|EHD3|ACSM2B|SHMT2|PKLR|PFKM|WARS|AGK|UBE2D3|VCP|MYH10|LONP1|PNP|KIF21A|MARC2|MYO6|ABCD3|GOT1|CMPK1|ASS1|PRPS1|VARS|CUBN|APRT|NQO2|GLDC|NME2|PC|PRKCD|DAK|AK4|MYH11|ABAT|ILK|ATP5A1|GK|ACSF2|DDC|CKMT2|CKMT1A|FBP1|LARS2|AOC1|PPIA|ACSL1|NDUFA13|KIF13B|ATP1A1|MMAB|TWF1|ACSS3|ACSM2A|GALK1|DDX6|PSMC3</td><td>1.68E-06</td><td>GO.0008144</td><td>0.4419075</td></tr>
<tr><td>323</td><td>26</td><td>GO Process</td><td>response to acid chemical</td><td>3.8E-05</td><td>CTSH|FIS1|CPT1A|LAMTOR1|CAPN2|AQP1|HNRNPD|SHMT1|PCK1|LAMTOR4|COL18A1|OPA1|COL6A1|RBP4|ASS1|GSN|HMGCL|COL4A1|AKR1C3|GLDC|CD9|RHOA|ACSL1|NDUFA13|PHB2|KRT8</td><td>2.06E-06</td><td>GO.0001101</td><td>0.4419075</td></tr>
<tr><td>896</td><td>50</td><td>GO Process</td><td>cellular response to oxygen-containing compound</td><td>4.1E-05</td><td>GNA11|ATP6V1D|FIS1|PPIF|ATP6V1B1|ATP6V1E1|CDH1|PRDX1|ATP6V0A1|PRDX5|CPT1A|ATP6V1A|ATP6V1B2|LAMTOR1|CA2|ATP6V0D2|CAPN2|FABP1|GPD1|YWHAG|ATP6V0A4|AQP1|HNRNPD|SHMT1|PCK1|PKM|ATP6V0C|RAB11B|PKLR|LAMTOR4|ATP6V1H|MSN|OPA1|COL6A1|GOT1|ASS1|ATP6V1G1|COL4A1|TBC1D4|APRT|AKR1C3|FECH|PRKAR1A|DPEP1|PRKCD|MGST1|RHOA|AOC1|NDUFA13|PHB2</td><td>2.22E-06</td><td>GO.1901701</td><td>0.43882767</td></tr>
</table>

1061	55	GO Component	plasma membrane region	4.1E-05	RALA|NIPSNAP1|MYH9|ATP6V1B1|ATP6V1E1|RAB21|CDH1|RAB11A|DNAJA3|SLC4A1|SLC9A3R1|PACSIN2|EPCAM|LRP2|RAB17|ENPEP|RHCG|ATP6V1A|CA2|ATP6V0D2|AMN|CDH16|SLC23A1|ATP6V0A4|CYP4A11|AQP1|TLN1|SLC7A8|EHD3|EPB41L3|PFKM|GM2A|MSN|MYH10|DPP4|KRT19|ATP1B1|MYO6|AKR1A1|KCTD12|CUBN|SLC3A2|CD9|PRKAR1A|DPEP1|UMOD|SEPT7|SLC4A4|PDZK1|RHOA|ATP1A1|MGAM|KRT8|TWF1|PDLIM5	5.51E-06	GO.0098590	0.43861582
70	14	KEGG Pathways	Metabolism of xenobiotics by cytochrome P450	3.7E-07	AKR7A2|GSTM3|CBR1|UGT2B7|ADH1B|UGT2B17|GSTA1|UGT1A9|AKR7A3|MGST3|GSTO1|MGST1|GSTA2|EPHX1	3.7E-08	hsa00980	0.64317983
148	11	KEGG Pathways	mTOR signaling pathway	0.0128	ATP6V1D|ATP6V1B1|ATP6V1E1|ATP6V1A|ATP6V1B2|LAMTOR1|LAMTOR4|ATP6V1H|ATP6V1G1|SLC3A2|RHOA	0.0033	hsa04150	0.189279
432	30	GO Component	actin cytoskeleton	4.2E-05	VCL|MYH9|MYL12A|VIL1|ACTN4|CDH1|SLC9A3R1|CAPG|ADD1|IQGAP1|CAPN2|SCIN|CFL2|MSRA|PLS3|MYH10|KRT19|FLNA|MYO6|GSN|CLIC4|ACTN1|ALDOA|MYH11|ILK|SEPT7|FLNB|WDR1|TWF1|PDLIM5	5.66E-06	GO.0015629	0.43788237
457	31	GO Component	endosome membrane	4.5E-05	GNPNAT1|EHD4|PLIN3|ARL8B|RAB21|RAB11A|PACSIN2|GRB14|RAB17|ATP6V0A1|SCARB2|RAB7A|ARL8A|LAMTOR1|ATP6V0D2|AMN|ATP6V0A4|EHD3|AP2A2|ATP6V0C|LAMP1|RAB11B|LAMTOR4|ANXA6|UBE2D3|RAB14|CUBN|UBA52|LAMP2|CD63|VPS29	6.17E-06	GO.0010008	0.43467875
245	22	GO Process	cellular response to peptide hormone stimulus	4.6E-05	ATP6V1D|ATP6V1B1|ATP6V1E1|ATP6V0A1|ATP6V1A|ATP6V1B2|CA2|ATP6V0D2|YWHAG|ATP6V0A4|PCK1|PKM|ATP6V0C|PKLR|ATP6V1H|GOT1|ASS1|ATP6V1G1|TBC1D4|APRT|PRKAR1A|PRKCD	2.48E-06	GO.0071375	0.43410352
119	15	GO Process	monovalent inorganic cation homeostasis	4.6E-05	ATP6V1B1|SLC4A1|ATP6V0A1|RAB7A|RHCG|CA2|ATP6V0D2|ATP6V0A4|CYP4A11|ATP6V0C|ATP6V1H|ATP1B1|CLIC4|SLC4A4|ATP1A1	2.49E-06	GO.0055067	0.43400838
414	30	GO Process	organic anion transport	4.7E-05	CA12|APOA1|GOT2|SLC4A1|SLC9A3R1|LRP2|CPT1A|SLC27A2|SLC25A4|CA2|FABP1|SLC23A1|AQP1|GLS|SLC7A8|BSG|CYB5R1|ABCD3|CPT2|SCP2|SLC25A5|SLC3A2|SLC25A6|PSAP|SLC25A12|SLC4A4|ACSL1|SLC25A10|NPC2|SLC43A2	2.56E-06	GO.0015711	0.43306831
724	43	GO Process	interspecies interaction between organisms	4.9E-05	RALA|PSMA4|LTF|LGALS3|APCS|LYZ|CDH1|SCARB2|RAB7A|AMBP|VDAC1|SLC25A4|PSMB4|ANPEP|RAB1B|AQP1|RAB6A|TLN1|AP2A2|KRT7|ATP6V0C|LAMP1|VAPA|CTSB|SND1|VCP|ATP6V1H|DPP4|KRT19|SLC25A5|PSMB9|PSMB8|SLC25A6|KRT18|PC|UBA52|RHOA|GPX1|PPIA|RAN|KRT8|DDX6|PSMC3	2.7E-06	GO.0044419	0.43089185
5	5	GO Process	isocitrate metabolic process	5E-05	ACO2|IDH3G|IDH2|IDH3B|IDH1	2.78E-06	GO.0006102	0.4298432
55	10	GO Component	tertiary granule lumen	5.1E-05	CTSH|LTF|CTSD|GGH|LYZ|FTH1|ASAH1|NIT2|ALDOA|IDH1	7.05E-06	GO.1904724	0.42932822
90	13	GO Process	cellular detoxification	5.5E-05	TXNL1|GSTM3|PRDX1|NNT|PRDX5|FABP1|GSTA1|MGST3|GSTO1|GPX3|MGST1|TXNRD2|GPX1	3.04E-06	GO.1990748	0.42628074
38	9	GO Process	nucleobase metabolic process	5.6E-05	MTHFD1|GDA|DPYS|HPRT1|SHMT1|CMPK1|PRPS1|APRT|ALDH6A1	3.12E-06	GO.0009112	0.4251812
161	17	Reactome Pathways	Apoptosis	5.8E-05	PSMA4|PSMD7|PSMD11|CDH1|PSMD3|ADD1|PSMA5|PSMB4|YWHAG|CYCS|OPA1|GSN|PSMB9|PSMB8|PRKCD|UBA52|PSMC3	4.9E-06	HSA-109581	0.42380722
5	5	GO Function	glyceraldehyde-3-phosphate dehydrogenase (NAD+) (non-phosphorylating) activity	6.2E-05	ALDH2|ALDH3A2|ALDH9A1|ALDH1B1|ALDH7A1	2.78E-06	GO.0043878	0.42090115

5	5	GO Function	isocitrate dehydrogenase activity	6.2E-05	IDH3G|IDH3A|IDH2|IDH3B|IDH1	2.78E-06	GO.0004448	0.42090115
1514	72	GO Process	protein-containing complex assembly	6.2E-05	HSD17B10|NDUFB4|EHD4|FIS1|ACOT13|ACADL|APOA1|TFG|VIL1|NDUFA2|NDUFA10|APCS|PSMD11|SLC9A3R1|NAPA|CAPG|ATP6V0A1|CPT1A|NDUFA9|ECSIT|DPYS|NDUFB9|HPRT1|NDUFB8|COL6A2|TMED10|DCXR|ATP6V0A4|RAB1B|GRHPR|TLN1|GLS|SHMT1|PKM|NDUFV1|DHRS4|EHD3|NDUFA12|UQCR10|SHMT2|PFKM|ANXA6|VCP|LONP1|OPA1|COA3|COL6A1|NDUFS2|RPL5|GSN|HMGCL|ALDOB|KCTD12|NAP1L4|GPX3|ALDOA|QPRT|CD59|MGST1|MYH11|ILK|SEPT7|ELAVL1|UBA52|NDUFS1|CRYZ|FBP1|SERPINA1|TMEM33|NDUFA13|CIRBP|DDX6	3.47E-06	GO.0065003	0.42083094
708	42	GO Process	ion homeostasis	6.5E-05	DCN|CA12|FIS1|SFXN3|LTF|ATP6V1B1|APOA1|HEXB|SLC4A1|SLC9A3R1|ATP6V0A1|RAB7A|CALB1|RHCG|AFG3L2|ATP6V1A|FTH1|GPD1L|CA2|ATP6V0D2|CKB|ATP6V0A4|ACO1|CYP4A11|SFXN1|SLC7A8|ATP6V0C|ANXA6|ATP6V1H|ATP1B1|GSTO1|SFXN2|GOT1|ATP6V1G1|CLIC4|FHL1|UMOD|IMMT|SLC4A4|EPHX2|ATP1A1|PLCG2	3.65E-06	GO.0050801	0.41877553
76	12	GO Function	flavin adenine dinucleotide binding	6.9E-05	ACADL|ACADS|ACAD11|PRODH2|CHDH|ACADSB|ACADM|MAOB|NQO2|TXNRD2|ETFDH|FMO1	3.2E-06	GO.0050660	0.41643094
51	10	GO Process	NAD biosynthetic process	6.9E-05	ENO1|PKM|PKLR|PFKM|PNP|ALDOB|BPGM|ALDOA|QPRT|GPI	3.9E-06	GO.0009435	0.4160522
840	47	GO Process	regulation of catabolic process	7.5E-05	DCN|ATP6V1D|CDC37|ATP6V1B1|APOA1|CAPNS1|HSPB1|ATP6V1E1|LRP2|PSMD3|ATP6V0A1|NNT|RAB7A|VDAC1|CPT1A|ATP6V1A|ATP6V1B2|LAMTOR1|UCHL1|ATP6V0D2|FABP1|GPD1|RAB1B|HNRNPD|DAB2|ATP6V0C|LAMTOR4|VCP|ATP6V1H|MSN|FLNA|RPL5|ATP6V1G1|HNRNPR|BPGM|PRKCD|PSAP|ELAVL1|UBA52|IDH1|GPX1|FBP1|NDUFA13|CAPN1|CIRBP|PHB|PSMC3	4.24E-06	GO.0009894	0.41255182
158	17	GO Function	actin filament binding	8.2E-05	MYH9|VIL1|ACTN4|CAPG|ADD1|SCIN|CFL2|TLN1|PLS3|MYH10|FLNA|MYO6|GSN|ACTN1|MYH11|WDR1|TAGLN	3.89E-06	GO.0051015	0.40877779
20	7	GO Process	alditol metabolic process	8.2E-05	SORD|GPD2|PCK1|GOT1|DAK|GK|GALK1	4.67E-06	GO.0019400	0.40861861
6	5	GO Process	choline catabolic process	8.7E-05	DMGDH|BHMT|CHDH|SARDH|ALDH7A1	4.98E-06	GO.0042426	0.40599818
155	11	KEGG Pathways	Necroptosis	0.0167	PYGB|VDAC1|FTH1|GLUD1|SLC25A4|CAPN2|SLC25A5|HIST1H2AC|SLC25A6|PPIA|CAPN1	0.0045	hsa04217	0.17772835
571	36	GO Process	viral process	9.1E-05	RALA|PSMA4|SCARB2|RAB7A|AMBP|VDAC1|SLC25A4|PSMB4|ANPEP|RAB1B|RAB6A|TLN1|AP2A2|KRT7|ATP6V0C|LAMP1|VAPA|CTSB|SND1|VCP|ATP6V1H|DPP4|KRT19|SLC25A5|PSMB9|PSMB8|SLC25A6|KRT18|PC|UBA52|RHOA|PPIA|RAN|KRT8|DDX6|PSMC3	5.25E-06	GO.0016032	0.40390538
30	8	GO Process	NADP metabolic process	9.1E-05	NNT|PRDX5|DCXR|TALDO1|IDH2|IDH1|TKT|FMO1	5.26E-06	GO.0006739	0.40390538
30	8	GO Process	glutamate metabolic process	9.1E-05	GOT2|GLUD1|FTCD|PRODH2|GLS|GOT1|ALDH4A1|GGT1	5.26E-06	GO.0006536	0.40390538
81	12	GO Process	water-soluble vitamin metabolic process	9.9E-05	MTHFD1|AMN|SLC23A1|SHMT1|SHMT2|CYB5A|GSTO1|AKR1A1|PSAT1|CUBN|PC|MMAB	5.76E-06	GO.0006767	0.40026136
122	14	GO Component	phagocytic vesicle	0.0001	LTF|RAB11A|ATP6V0A1|RAB7A|ATP6V0D2|ATP6V0A4|ATP6V0C|LAMP1|RAB11B|GSN|RAB14|ANXA11|LAMP2|FLNB	1.42E-05	GO.0045335	0.4

819	46	GO Function	pyrophosphatase activity	0.0001	RALA|GNA11|MYH9|ATP6V1D|PFN2|ATP6V1E1|ARL8B|RAB21|RAB11A|RAB2A|RAB17|ATP6V0A1|RAB7A|ARL8A|ATP6V1A|ATP6V1B2|ATP6V0D2|GNB2|ATP6V0A4|RAB1B|RAB6A|RHOT2|ATP6V0C|RAB11B|PPA2|VCP|ATP6V1H|MYH10|LONP1|OPA1|KIF21A|ATP1B1|LHPP|MYO6|ABCD3|RAB14|ATP6V1G1|MYH11|ATP5A1|RHOA|GFM1|KIF13B|ATP1A1|RAN|DDX6|PSMC3	4.91E-06	GO.0016462	0.4
21	7	GO Process	glutathione derivative biosynthetic process	0.0001	GSTM3|GSTA1|MGST3|GSTO1|AKR1A1|MGST1|GSTA2	6.09E-06	GO.1901687	0.4
5126	185	GO Process	cellular nitrogen compound metabolic process	0.0001	HSD17B10|NDUFB4|NANS|GNPNAT1|MTHFD1|AHCY|CTSH|HNRNPL|OGDH|PDHX|ACOT13|DNPH1|LTF|ACADL|ATP6V1B1|ENO1|GDA|PMPCB|RPS16|NDUFA2|NDUFA10|LGALS3|GRSF1|HRSP12|DMGDH|GSTM3|NARS|GGH|HEXB|DNAJA3|CHCHD3|DHTKD1|BBOX1|ATP6V0A1|NNT|AMBP|ENPEP|PRDX5|CPT1A|REXO2|NDUFA9|NAT8|ATP6V1A|BHMT|ATP6V1B2|DPYS|NDUFB9|ENDOD1|HNMT|DLAT|QDPR|GPD1L|FTCD|RNPEP|APEH|HPRT1|NDUFB8|TPP1|ANPEP|GPD1|SLC23A1|RPS9|DCXR|HINT1|ASL|PDHB|CYCS|ATP6V0A4|GPD2|DPYSL2|UQCRH|AQP1|GBAS|HNRNPD|GRHPR|TSFM|AHCYL2|ACSS1|CYC1|PIPOX|SHMT1|UGDH|CHDH|PKM|TALDO1|NDUFV1|HNRNPM|CNDP2|MDH2|ACSM2B|NDUFA12|IDH2|UQCR10|SHMT2|DLST|GSTA1|PKLR|PPA2|PFKM|SND1|ALDH9A1|WARS|AGK|GM2A|UBE2D3|VCP|COL4A2|LONP1|MRPL37|MRPL24|PNP|MRPL21|OPA1|IBA57|ATP1B1|NDUFS2|LHPP|GSTO1|FLNA|RPL5|ACADM|SARDH|CMPK1|ASS1|PRPS1|OGDHL|HMGCL|HNRNPR|ALDOB|VARS|AGMAT|PSAT1|APRT|ACOT9|PDHA1|ASAH1|FECH|DPEP1|BPGM|NME2|CNP|PC|NIT2|ALDOA|AK4|QPRT|AMPD3|MPST|HAGH|GGT5|ATP5A1|GGT1|ELAVL1|TST|ALDH7A1|UBA52|IDH1|TKT|NDUFS1|RHOA|ACSF2|DDC|GPI|GPX1|LAMP2|LARS2|MRPS22|MME|GFM1|PPIA|SSBP1|ABHD14B|GSTA2|ACSL1|PHB2|MMAB|RAN|RPL18|ALDH6A1|ACSM2A|GALK1|DDX6|PHB|FMO1|GLYAT	6.09E-06	GO.0034641	0.4
21	7	GO Process	aspartate family amino acid catabolic process	0.0001	CRYM|GOT2|HRSP12|PIPOX|DLST|GOT1|ALDH7A1	6.09E-06	GO.0009068	0.4
159	16	GO Component	specific granule	0.00011	VCL|ATP6V1D|LTF|CTSD|GGH|LYZ|SLC27A2|LAMTOR1|TOLLIP|ACAA1|CKAP4|NIT2|CD59|ANXA11|AOC1|TOM1	1.61E-05	GO.0042581	0.39586073
53	10	GO Function	pyridoxal phosphate binding	0.00011	PYGB|AGXT2|GOT2|SHMT1|SHMT2|MARC2|GOT1|GLDC|ABAT|DDC	5.28E-06	GO.0030170	0.39586073
17	3	KEGG Pathways	Nitrogen metabolism	0.0473	CA12|GLUD1|CA2	0.0147	hsa00910	0.13251389
13	6	GO Process	carnitine metabolic process	0.00011	ACADL|BBOX1|CPT1A|SHMT1|ALDH9A1|ACADM	6.49E-06	GO.0009437	0.39586073
16	6	GO Component	vacuolar proton-transporting V-type ATPase complex	0.00012	ATP6V1B1|ATP6V0A1|ATP6V0D2|ATP6V0A4|ATP6V1H|ATP6V1G1	1.67E-05	GO.0016471	0.39208188
603	37	GO Function	peptidase activity, acting on L-amino acid peptides	0.00013	PSMA4|F9|CTSH|LTF|CTSD|PEPD|CAPNS1|PMPCB|NAPSA|TINAG|GGH|ENPEP|AFG3L2|TINAGL1|PSMA5|UCHL1|PSMB4|CAPN2|RNPEP|APEH|TPP1|ANPEP|CNDP2|CTSB|DPP4|LONP1|XPNPEP2|PSMB9|PSMB8|DPEP1|GGT5|GGT1|ACY1|SCRN1|CFB|MME|CAPN1	6.91E-06	GO.0070011	0.38860566
370	27	GO Function	purine ribonucleoside binding	0.00013	RALA|GNA11|PCK2|EHD4|ARL8B|RAB21|RAB11A|RAB2A|RAB17|RAB7A|ARL8A|GLUD1|RAB1B|RAB6A|PCK1|RHOT2|EHD3|RAB11B|ANXA6|OPA1|PRPS1|RAB14|AK4|SEPT7|RHOA|GFM1|RAN	7.21E-06	GO.0032550	0.38860566

778	44	GO Function	nucleoside-triphosphatase activity	0.00013	RALA|GNA11|MYH9|ATP6V1D|PFN2|ATP6V1E1|ARL8B|RAB21|RAB11A|RAB2A|RAB17|ATP6V0A1|RAB7A|ARL8A|ATP6V1A|ATP6V1B2|ATP6V0D2|GNB2|ATP6V0A4|RAB1B|RAB6A|RHOT2|ATP6V0C|RAB11B|VCP|ATP6V1H|MYH10|LONP1|OPA1|KIF21A|ATP1B1|MYO6|ABCD3|RAB14|ATP6V1G1|MYH11|ATP5A1|RHOA|GFM1|KIF13B|ATP1A1|RAN|DDX6|PSMC3	6.74E-06	GO.0017111	0.38860566
99	13	GO Process	icosanoid metabolic process	0.00013	CBR1|CYP4A11|CES2|PTGES2|MGST3|AKR1C3|DPEP1|GGT5|GGT1|PTGR1|GPX1|EPHX2|PTGR2	7.78E-06	GO.0006690	0.38860566
2505	102	GO Component	extracellular region	0.00014	HEBP1|EPDR1|VCL|PYGB|F9|PSMD7|CTSH|LTF|CTSD|APOA1|TTR|PLBD1|ACTN4|LAMA5|NAPSA|LGALS3|APCS|GSTM3|TINAG|GGH|LYZ|HEXB|PSMD11|CDH1|RAB11A|COTL1|PSMD3|F13A1|AMBP|SORD|TINAGL1|PSMA5|FTH1|BHMT|ENDOD1|AGL|APEH|COL14A1|AMN|CKB|ANPEP|COL6A2|GUSB|AQP1|TOLLIP|TLN1|PKM|SERPINA5|ACAA1|APOD|PTGES2|CTSB|COL18A1|GM2A|VCP|COL4A2|DPP4|PNP|COL6A1|TAGLN2|FLNA|XPNPEP2|RBP4|APOOL|GSN|HSPG2|ASPN|CLIC1|COL4A1|CUBN|APRT|TXNDC5|ASAH1|CD9|GPX3|DPEP1|NME2|CNP|NIT2|ACTN1|PRKCD|PSAP|ALDOA|CD59|AMPD3|ACY1|IFI30|PDIA6|IDH1|GPI|LAMP2|AOC1|SERPINA1|TGFBI|CFB|VWA1|PPIA|WDR1|CAPN1|CD63|NPC2|PSMC3	2.01E-05	GO.0005576	0.3853872
70	11	GO Process	purine ribonucleoside metabolic process	0.00014	AHCY|HPRT1|AHCYL2|PNP|OPA1|APRT|NME2|AK4|AMPD3|RHOA|RAN	8.55E-06	GO.0046128	0.3853872
836	46	GO Process	intracellular protein transport	0.00014	ECH1|CDC37|FIS1|EHHADH|HSPB1|PMPCB|RPS16|SYNJ2BP|RAB11A|NAPA|SCARB2|RAB7A|SLC27A2|HSPA9|RPS9|TMED10|YWHAG|PIPOX|DHRS4|AP2A2|COPB2|ACAA1|AGK|UBE2D3|VCP|ATP1B1|MYO6|RPL5|SCP2|HMGCL|TBC1D4|AKR1C3|SLC25A6|UBA52|IDH1|LAMP2|TOMM40|TOM1|NDUFA13|KIF13B|EPHX2|PHB2|RAN|RPL18|VPS29|HAO2	8.12E-06	GO.0006886	0.3853872
17	6	GO Component	proton-transporting two-sector ATPase complex, catalytic domain	0.00015	ATP6V1B1|ATP6V1E1|ATP6V1A|ATP6V1B2|ATP6V1H|ATP5A1	2.21E-05	GO.0033178	0.38239087
117	14	GO Process	vitamin metabolic process	0.00015	MTHFD1|LRP2|CBR1|AMN|SLC23A1|SHMT1|SHMT2|CYB5A|GSTO1|AKR1A1|PSAT1|CUBN|PC|MMAB	9.25E-06	GO.0006766	0.38239087
44	9	GO Process	mitochondrial electron transport, NADH to ubiquinone	0.00015	NDUFB4|NDUFA2|NDUFA10|NDUFA9|NDUFB9|NDUFB8|NDUFV1|NDUFS2|NDUFS1	8.83E-06	GO.0006120	0.38239087
1794	80	GO Function	purine ribonucleoside triphosphate binding	0.00016	RALA|GNA11|MYH9|MTHFD1|PCK2|IDH3G|EHD4|ATP6V1B1|ARL8B|NARS|KHK|RAB21|RAB11A|DNAJA3|RAB2A|RAB17|RAB7A|SLC27A2|AFG3L2|ARL8A|ATP6V1A|ATP6V1B2|GLUD1|HSPA9|CKB|RAB1B|RAB6A|ACSS1|PCK1|PKM|RHOT2|EHD3|ACSM2B|RAB11B|PKLR|PFKM|ANXA6|WARS|AGK|UBE2D3|VCP|MYH10|LONP1|OPA1|KIF21A|MYO6|ABCD3|CMPK1|ASS1|PRPS1|RAB14|VARS|NME2|PC|PRKCD|DAK|AK4|MYH11|ILK|ATP5A1|SEPT7|RHOA|GK|ACSF2|CKMT2|CKMT1A|LARS2|GFM1|ACSL1|NDUFA13|KIF13B|ATP1A1|MMAB|RAN|TWF1|ACSS3|ACSM2A|GALK1|DDX6|PSMC3	9.41E-06	GO.0035639	0.379588
149	16	KEGG Pathways	Non-alcoholic fatty liver disease (NAFLD)	4.2E-05	NDUFB4|NDUFA2|NDUFA10|NDUFA9|NDUFB9|NDUFB8|CYCS|UQCRH|CYC1|NDUFV1|NDUFA12|UQCR10|PKLR|NDUFS2|NDUFS1|NDUFA13	7.64E-06	hsa04932	0.43798639
289	23	GO Process	cellular response to peptide	0.00016	ATP6V1D|FIS1|ATP6V1B1|ATP6V1E1|ATP6V0A1|ATP6V1A|ATP6V1B2|CA2|ATP6V0D2|YWHAG|ATP6V0A4|PCK1|PKM|ATP6V0C|PKLR|ATP6V1H|GOT1|ASS1|ATP6V1G1|TBC1D4|APRT|PRKAR1A|PRKCD	9.45E-06	GO.1901653	0.379588
86	12	GO Process	cellular oxidant detoxification	0.00016	TXNL1|PRDX1|NNT|PRDX5|FABP1|GSTA1|MGST3|GSTO1|GPX3|MGST1|TXNRD2|GPX1	9.98E-06	GO.0098869	0.379588

5163	185	GO Process	cellular component organization	0.00016	RALA|DCN|HSD17B10|NDUFB4|VCL|MYH9|ATP6V1D|EHD4|ECH1|FIS1|PPIF|ACOT13|EHHADH|ACADL|APOA1|TTR|PFN2|TFG|VIL1|PMPCB|NDUFA2|ACTN4|NDUFA10|LAMA5|APCS|SYNJ2BP|RAB21|HEXB|PSMD11|CDH1|RAB11A|DNAJA3|CHCHD3|SLC9A3R1|RAB2A|PACSIN2|NAPA|LRP2|CAPG|RAB17|ATP6V0A1|ADD1|SCARB2|RAB7A|CPT1A|NDUFA9|SLC27A2|IQGAP1|AFG3L2|ECSIT|DPYS|NDUFB9|LAMTOR1|SLC25A4|UCHL1|ACAA2|TBC1D24|APEH|SCIN|HSPA9|COL14A1|CFL2|HPRT1|NDUFB8|TPP1|COL6A2|TMED10|DCXR|GAA|YWHAG|CYCS|ATP6V0A4|DPYSL2|RAB1B|AQP1|DAB2|GRHPR|TLN1|GLS|PIPOX|SHMT1|PKM|RHOT2|NDUFV1|DHRS4|EHD3|AP2A2|ATP6V0C|NDUFA12|TMED9|UQCR10|SERPINA5|ACAA1|SHMT2|BSG|PARVA|LAMTOR4|EPB41L3|APOD|VAPA|PFKM|ANXA6|AGK|COL18A1|PLS3|UBE2D3|VCP|MSN|MYH10|COL4A2|LONP1|MRPL37|MRPL24|MRPL21|OPA1|COA3|KRT19|COL6A1|IBA57|ATP1B1|DPT|NDUFS2|PHGDH|FLNA|ABCD3|RPL5|SCP2|SLC25A5|APOOL|GSN|RAB14|CLIC4|HMGCL|HSPG2|ALDOB|COL4A1|KCTD12|HIST1H2AC|NAP1L4|SLC25A6|CD9|GPX3|KRT18|PRKAR1A|CNP|ACTN1|PRKCD|ALDOA|QPRT|CD59|MGST1|MYH11|ILK|ATP5A1|SEPT7|ELAVL1|IMMT|UBA52|IDH1|NDUFS1|GDI1|CRYZ|RHOA|FBP1|LAMP2|TOMM40|SERPINA1|TGFBI|VWA1|MRPS22|PPIA|SSBP1|FLNB|TMEM33|NDUFA13|WDR1|EPHX2|PHB2|RAN|KRT8|TWF1|CIRBP|DDX6|PHB|HAO2	9.6E-06	GO.0016043	0.379588
876	46	GO Component	endosome	0.00017	GNPNAT1|CTSH|EHD4|PLIN3|APOA1|ATP6V1E1|NAPSA|ARL8B|RAB21|CDH1|RAB11A|PACSIN2|LRP2|GRB14|RAB17|ATP6V0A1|SCARB2|RAB7A|ARL8A|LAMTOR1|ATP6V0D2|AMN|ATP6V0A4|AOC3|EHD3|AP2A2|ATP6V0C|LAMP1|RAB11B|LAMTOR4|CTSB|ANXA6|UBE2D3|RAB14|CUBN|OCIAD1|UBA52|RHOA|LAMP2|TOM1|OCIAD2|ATP1A1|RAN|CD63|PHB|VPS29	2.48E-05	GO.0005768	0.37695511
118	14	GO Function	ATPase-coupled transmembrane transporter activity	0.00017	ATP6V1D|ATP6V1E1|ATP6V0A1|ATP6V1A|ATP6V1B2|ATP6V0D2|ATP6V0A4|ATP6V0C|ATP6V1H|ATP1B1|ABCD3|ATP6V1G1|ATP5A1|ATP1A1	1.01E-05	GO.0042626	0.37695511
23	7	GO Function	transferase activity, transferring nitrogenous groups	0.00017	AGXT2|GOT2|BCAT2|GOT1|PSAT1|ABAT|GATM	0.00001	GO.0016769	0.37695511
312	24	GO Process	protein homooligomerization	0.00017	HSD17B10|EHD4|FIS1|ACOT13|ACADL|CPT1A|DPYS|HPRT1|DCXR|GLS|SHMT1|PKM|EHD3|SHMT2|ANXA6|VCP|LONP1|KCTD12|GPX3|ALDOA|MGST1|ELAVL1|CRYZ|FBP1	1.03E-05	GO.0051260	0.37695511
58	10	GO Function	oxidoreductase activity, acting on the CH-CH group of donors	0.00018	ACADL|ACADS|MECR|ACAD11|ACAA1|ACADSB|ACADM|AKR1C3|PTGR1|PTGR2	1.07E-05	GO.0016627	0.37447275
743	42	GO Process	regulation of cellular catabolic process	0.00018	DCN|ATP6V1D|CDC37|ATP6V1B1|APOA1|CAPNS1|HSPB1|ATP6V1E1|LRP2|ATP6V0A1|NNT|VDAC1|CPT1A|ATP6V1A|ATP6V1B2|LAMTOR1|UCHL1|ATP6V0D2|FABP1|GPD1|RAB1B|HNRNPD|DAB2|ATP6V0C|LAMTOR4|VCP|ATP6V1H|MSN|RPL5|ATP6V1G1|HNRNPR|BPGM|PRKCD|PSAP|ELAVL1|UBA52|IDH1|GPX1|FBP1|CAPN1|CIRBP|PSMC3	1.11E-05	GO.0031329	0.37447275
2343	98	GO Process	cellular component assembly	0.00018	HSD17B10|NDUFB4|VCL|ATP6V1D|EHD4|FIS1|ACOT13|ACADL|APOA1|TFG|VIL1|NDUFA2|ACTN4|NDUFA10|LAMA5|APCS|PSMD11|CDH1|RAB11A|SLC9A3R1|PACSIN2|NAPA|CAPG|RAB17|ATP6V0A1|ADD1|RAB7A|CPT1A|NDUFA9|ECSIT|DPYS|NDUFB9|HSPA9|CFL2|HPRT1|NDUFB8|COL6A2|TMED10|DCXR|YWHAG|ATP6V0A4|RAB1B|GRHPR|TLN1|GLS|SHMT1|PKM|NDUFV1|DHRS4|EHD3|NDUFA12|UQCR10|SHMT2|PARVA|EPB41L3|PFKM|ANXA6|PLS3|VCP|MYH10|LONP1|OPA1|COA3|KRT19|COL6A1|IBA57|NDUFS2|FLNA|RPL5|GSN|HMGCL|ALDOB|KCTD12|NAP1L4|CD9|GPX3|PRKAR1A|ACTN1|ALDOA|QPRT|CD59|MGST1|MYH11|ILK|SEPT7|ELAVL1|UBA52|NDUFS1|CRYZ|RHOA|FBP1|SERPINA1|TMEM33|NDUFA13|WDR1|KRT8|CIRBP|DDX6	1.07E-05	GO.0022607	0.37447275

120	14	GO Function	proton transmembrane transporter activity	0.00019	SLC25A3|ATP6V1B1|ATP6V1E1|ATP6V0A1|NNT|ATP6V1A|ATP6V1B2|ATP6V0D2|ATP6V0A4|ATP6V0C|CYB5A|ATP6V1H|ATP6V1G1|ATP5A1	0.000012	GO.0015078	0.37212464
137	15	GO Process	proton transmembrane transport	0.00019	SLC25A3|ATP6V1B1|ATP6V1E1|ATP6V0A1|NNT|ATP6V1A|ATP6V1B2|ATP6V0D2|ATP6V0A4|CYC1|ATP6V0C|CYB5A|ATP6V1H|ATP6V1G1|ATP5A1	1.18E-05	GO.1902600	0.37212464
34	8	GO Process	pH reduction	0.00019	ATP6V1B1|ATP6V0A1|RAB7A|ATP6V0D2|ATP6V0A4|ATP6V0C|ATP6V1H|CLIC4	1.16E-05	GO.0045851	0.37212464
120	14	GO Process	nucleoside metabolic process	0.00019	AHCY|DPYS|HPRT1|AHCYL2|PNP|OPA1|CMPK1|PRPS1|APRT|NME2|AK4|AMPD3|RHOA|RAN	0.000012	GO.0009116	0.37212464
10	5	GO Component	dihydrolipoyl dehydrogenase complex	0.0002	OGDH|DHTKD1|BCKDHA|DLST|OGDHL	2.97E-05	GO.0045240	0.369897
15	6	GO Function	aldehyde dehydrogenase (NAD) activity	0.0002	ALDH2|ALDH3A2|ALDH9A1|ALDH4A1|ALDH1B1|ALDH7A1	1.24E-05	GO.0004029	0.369897
20	4	KEGG Pathways	One carbon pool by folate	0.0128	MTHFD1|FTCD|SHMT1|SHMT2	0.0033	hsa00670	0.189279
15	6	GO Process	fructose metabolic process	0.0002	KHK|SORD|ALDOB|DAK|ALDOA|FBP1	1.24E-05	GO.0006000	0.369897
384	27	GO Function	guanyl ribonucleotide binding	0.00021	RALA|GNA11|PCK2|EHD4|ARL8B|RAB21|RAB11A|RAB2A|RAB17|RAB7A|ARL8A|GLUD1|RAB1B|RAB6A|PCK1|RHOT2|EHD3|RAB11B|ANXA6|OPA1|PRPS1|RAB14|AK4|SEPT7|RHOA|GFM1|RAN	1.35E-05	GO.0032561	0.36777807
1235	60	GO Process	transmembrane transport	0.00021	ATP6V1D|SFXN3|SLC25A3|ATP6V1B1|PMPCB|ATP6V1E1|SLC4A1|LRP2|ATP6V0A1|NNT|ADD1|VDAC1|CPT1A|SLC27A2|RHCG|AFG3L2|ATP6V1A|ATP6V1B2|ERLIN2|SLC25A4|ATP6V0D2|SLC23A1|ATP6V0A4|AQP1|SFXN1|CYC1|SLC7A8|SLC5A2|ATP6V0C|BSG|CYB5A|ANXA6|AGK|VCP|ATP6V1H|OPA1|ATP1B1|SFXN2|ABCD3|RBP4|CPT2|SLC25A5|ATP6V1G1|CLIC4|CLIC1|SLC3A2|SLC25A6|ATP5A1|TST|UBA52|SLC25A12|SLC4A4|TOMM40|NDUFA13|PHB2|ATP1A1|SLC25A10|SLC43A2|PHB|PLCG2	1.34E-05	GO.0055085	0.36777807
524	33	GO Process	anion transport	0.00021	CA12|APOA1|GOT2|SLC4A1|SLC9A3R1|LRP2|VDAC1|CPT1A|SLC27A2|SLC25A4|CA2|FABP1|SLC23A1|AQP1|GLS|SLC7A8|BSG|CYB5R1|ABCD3|CPT2|SCP2|SLC25A5|CLIC4|CLIC1|SLC3A2|SLC25A6|PSAP|SLC25A12|SLC4A4|ACSL1|SLC25A10|NPC2|SLC43A2	1.33E-05	GO.0006820	0.36777807
295	23	GO Process	regulation of neurotransmitter levels	0.00021	AGXT2|PFN2|HRSP12|DMGDH|NAPA|BHMT|DPYS|HNMT|DDAH1|SHMT1|CHDH|SHMT2|ALDH9A1|PHGDH|SARDH|ASS1|MAOB|GLDC|ABAT|ALDH7A1|DDC|PAH|GLYAT	1.29E-05	GO.0001505	0.36777807
157	16	GO Process	protein tetramerization	0.00022	HSD17B10|ACOT13|ACADL|DPYS|HPRT1|DCXR|GLS|SHMT1|PKM|DHRS4|SHMT2|HMGCL|GPX3|ALDOA|CRYZ|FBP1	1.39E-05	GO.0051262	0.36575773
60	10	GO Process	amine metabolic process	0.00022	DMGDH|BHMT|HNMT|AOC3|CHDH|SARDH|AGMAT|ALDH7A1|DDC|AOC1	1.39E-05	GO.0009308	0.36575773
90	12	GO Process	unsaturated fatty acid metabolic process	0.00024	CBR1|CYP4A11|CES2|ACAA1|GSTA1|PTGES2|SCP2|AKR1C3|PTGR1|ACSL1|EPHX2|PTGR2	1.51E-05	GO.0033559	0.36197888
591	35	Reactome Pathways	Hemostasis	0.00024	GNA11|VCL|F9|APOA1|ACTN4|EPCAM|GRB14|F13A1|ENDOD1|GNB2|TLN1|SLC7A8|EHD3|SERPINA5|BSG|KIF21A|CYB5R1|ATP1B1|TAGLN2|FLNA|APOOL|SLC3A2|CD9|PRKAR1A|ACTN1|PSAP|ALDOA|RHOA|LAMP2|SERPINA1|PPIA|WDR1|KIF13B|CD63|PLCG2	2.47E-05	HSA-109582	0.36197888

<table>
<tr><td>968</td><td>50</td><td>GO Function</td><td>protein-containing complex binding</td><td>0.00025</td><td>DCN|GNA11|MYH9|FIS1|SLC25A3|ATP6V1B1|APOA1|VIL1|ACTN4|LAMA5|LGALS3|SLC9A3R1|NAPA|EPCAM|CAPG|ADD1|RAB7A|AMBP|NDUFA9|IQGAP1|SCIN|COL14A1|CFL2|TMED10|GNB2|TLN1|CTSB|PLS3|VCP|MYH10|KRT19|FLNA|MYO6|GSN|HSPG2|ASPN|NAP1L4|GPNMB|CD9|ACTN1|UMOD|MYH11|ILK|PDZK1|AOC1|TGFBI|WDR1|TAGLN|KRT8|TWF1</td><td>1.65E-05</td><td>GO.0044877</td><td>0.360206</td></tr>
<tr><td>124</td><td>14</td><td>GO Function</td><td>ATPase activity, coupled to movement of substances</td><td>0.00025</td><td>ATP6V1D|ATP6V1E1|ATP6V0A1|ATP6V1A|ATP6V1B2|ATP6V0D2|ATP6V0A4|ATP6V0C|ATP6V1H|ATP1B1|ABCD3|ATP6V1G1|ATP5A1|ATP1A1</td><td>1.68E-05</td><td>GO.0043492</td><td>0.360206</td></tr>
<tr><td>366</td><td>26</td><td>GO Function</td><td>GTP binding</td><td>0.00025</td><td>RALA|GNA11|PCK2|EHD4|ARL8B|RAB21|RAB11A|RAB2A|RAB17|RAB7A|ARL8A|GLUD1|RAB1B|RAB6A|PCK1|RHOT2|EHD3|RAB11B|ANXA6|OPA1|RAB14|AK4|SEPT7|RHOA|GFM1|RAN</td><td>1.65E-05</td><td>GO.0005525</td><td>0.360206</td></tr>
<tr><td>995</td><td>51</td><td>GO Process</td><td>ion transmembrane transport</td><td>0.00026</td><td>ATP6V1D|SFXN3|SLC25A3|ATP6V1B1|PMPCB|ATP6V1E1|SLC4A1|LRP2|ATP6V0A1|NNT|VDAC1|CPT1A|SLC27A2|RHCG|AFG3L2|ATP6V1A|ATP6V1B2|SLC25A4|ATP6V0D2|SLC23A1|ATP6V0A4|AQP1|SFXN1|CYC1|SLC7A8|SLC5A2|ATP6V0C|BSG|CYB5A|ANXA6|ATP6V1H|OPA1|ATP1B1|SFXN2|ABCD3|CPT2|SLC25A5|ATP6V1G1|CLIC4|CLIC1|SLC3A2|SLC25A6|ATP5A1|SLC25A12|SLC4A4|PHB2|ATP1A1|SLC25A10|SLC43A2|PHB|PLCG2</td><td>1.64E-05</td><td>GO.0034220</td><td>0.35850267</td></tr>
<tr><td>91</td><td>12</td><td>GO Process</td><td>regulation of pH</td><td>0.00026</td><td>ATP6V1B1|SLC4A1|ATP6V0A1|RAB7A|RHCG|CA2|ATP6V0D2|ATP6V0A4|ATP6V0C|ATP6V1H|CLIC4|SLC4A4</td><td>1.67E-05</td><td>GO.0006885</td><td>0.35850267</td></tr>
<tr><td>192</td><td>17</td><td>GO Component</td><td>actin-based cell projection</td><td>0.00027</td><td>ATP6V1B1|VIL1|ATP6V1E1|SLC9A3R1|ATP6V1A|ATP6V1B2|CA2|DCXR|AOC3|MSN|MYO6|CLIC4|DPEP1|CNP|PDZK1|KIF13B|TWF1</td><td>4.01E-05</td><td>GO.0098858</td><td>0.35686362</td></tr>
<tr><td>26</td><td>7</td><td>GO Function</td><td>CoA-ligase activity</td><td>0.00028</td><td>SLC27A2|ACSS1|ACSM2B|ACSF2|ACSL1|ACSS3|ACSM2A</td><td>1.96E-05</td><td>GO.0016405</td><td>0.3552842</td></tr>
<tr><td>9</td><td>5</td><td>GO Function</td><td>aldehyde-lyase activity</td><td>0.00029</td><td>SHMT1|SHMT2|HOGA1|ALDOB|ALDOA</td><td>2.02E-05</td><td>GO.0016832</td><td>0.3537602</td></tr>
<tr><td>585</td><td>35</td><td>GO Process</td><td>cellular response to hormone stimulus</td><td>0.00032</td><td>GNA11|ATP6V1D|CTSH|ATP6V1B1|ATP6V1E1|ATP6V0A1|ATP6V1A|ATP6V1B2|CA2|ATP6V0D2|YWHAG|ATP6V0A4|AQP1|HNRNPD|PCK1|PKM|ATP6V0C|PKLR|CTSB|ATP6V1H|MSN|GOT1|ASS1|ATP6V1G1|TBC1D4|APRT|AKR1C3|FECH|PRKAR1A|PRKCD|RHOA|ACSL1|ATP1A1|RAN|PHB</td><td>2.03E-05</td><td>GO.0032870</td><td>0.349485</td></tr>
<tr><td>43</td><td>8</td><td>GO Component</td><td>mitochondrial nucleoid</td><td>0.00033</td><td>GRSF1|DNAJA3|VDAC1|HSPA9|SHMT2|LONP1|SLC25A5|SSBP1</td><td>5.08E-05</td><td>GO.0042645</td><td>0.34814861</td></tr>
<tr><td>79</td><td>11</td><td>GO Function</td><td>antioxidant activity</td><td>0.00033</td><td>TXNL1|PRDX1|PRDX5|FABP1|GSTA1|MGST3|GSTO1|GPX3|MGST1|TXNRD2|GPX1</td><td>2.37E-05</td><td>GO.0016209</td><td>0.34814861</td></tr>
<tr><td>131</td><td>28</td><td>KEGG Pathways</td><td>Oxidative phosphorylation</td><td>4E-14</td><td>NDUFB4|ATP6V1D|ATP6V1B1|NDUFA2|NDUFA10|ATP6V1E1|ATP6V0A1|NDUFA9|ATP6V1A|ATP6V1B2|NDUFB9|ATP6V0D2|NDUFB8|ATP6V0A4|UQCRH|CYC1|NDUFV1|ATP6V0C|NDUFA12|UQCR10|PPA2|ATP6V1H|NDUFS2|LHPP|ATP6V1G1|ATP5A1|NDUFS1|NDUFA13</td><td>1.29E-15</td><td>hsa00190</td><td>1.33957739</td></tr>
<tr><td>57</td><td>9</td><td>GO Component</td><td>actin filament bundle</td><td>0.00035</td><td>MYH9|VIL1|ACTN4|PLS3|MYH10|ACTN1|ILK|SEPT7|FLNB</td><td>5.45E-05</td><td>GO.0032432</td><td>0.3455932</td></tr>
<tr><td>142</td><td>22</td><td>KEGG Pathways</td><td>Parkinson's disease</td><td>5.9E-09</td><td>NDUFB4|PPIF|NDUFA2|NDUFA10|VDAC1|NDUFA9|NDUFB9|SLC25A4|UCHL1|NDUFB8|CYCS|UQCRH|CYC1|NDUFV1|NDUFA12|UQCR10|NDUFS2|SLC25A5|SLC25A6|ATP5A1|NDUFS1|NDUFA13</td><td>3.74E-10</td><td>hsa05012</td><td>0.82328441</td></tr>
<tr><td>354</td><td>25</td><td>GO Function</td><td>active transmembrane transporter activity</td><td>0.00036</td><td>ATP6V1D|SLC25A3|ATP6V1E1|SLC4A1|ATP6V0A1|ATP6V1A|ATP6V1B2|SLC25A4|ATP6V0D2|SLC23A1|ATP6V0A4|SLC7A8|SLC5A2|ATP6V0C|ATP6V1H|ATP1B1|ABCD3|SLC25A5|ATP6V1G1|SLC3A2|SLC25A6|ATP5A1|SLC4A4|ATP1A1|SLC25A10</td><td>2.62E-05</td><td>GO.0022804</td><td>0.34436975</td></tr>
<tr><td>27</td><td>7</td><td>GO Process</td><td>glutamine family amino acid catabolic process</td><td>0.00038</td><td>GOT2|GLUD1|DDAH1|PRODH2|GLS|GOT1|ALDH4A1</td><td>2.41E-05</td><td>GO.0009065</td><td>0.34202164</td></tr>
<tr><td>27</td><td>7</td><td>GO Process</td><td>acetyl-CoA metabolic process</td><td>0.00038</td><td>PDHX|DLAT|PDHB|ACSS1|PIPOX|DLST|PDHA1</td><td>2.41E-05</td><td>GO.0006084</td><td>0.34202164</td></tr>
</table>

643	37	GO Process	inorganic ion homeostasis	0.0004	CA12|FIS1|SFXN3|LTF|ATP6V1B1|HEXB|SLC4A1|SLC9A3R1|ATP6V0A1|RAB7A|CALB1|RHCG|AFG3L2|ATP6V1A|FTH1|CA2|ATP6V0D2|CKB|ATP6V0A4|ACO1|CYP4A11|SFXN1|SLC7A8|ATP6V0C|ANXA6|ATP6V1H|ATP1B1|GSTO1|SFXN2|GOT1|ATP6V1G1|CLIC4|IMMT|SLC4A4|EPHX2|ATP1A1|PLCG2	2.61E-05	GO.0098771	0.339794
10	5	GO Function	hydrolase activity, acting on ether bonds	0.00041	AHCY|AKR7A2|RNPEP|EPHX2|EPHX1	2.97E-05	GO.0016801	0.33872161
34	9	KEGG Pathways	Pentose and glucuronate interconversions	8.9E-06	SORD|CRYL1|GUSB|DCXR|UGT2B7|UGDH|UGT2B17|UGT1A9|AKR1A1	1.42E-06	hsa00040	0.50520764
495	31	GO Process	amide biosynthetic process	0.00041	MTHFD1|PDHX|RPS16|NARS|DLAT|APEH|RPS9|ASL|PDHB|TSFM|ACSS1|CNDP2|PPA2|WARS|AGK|MRPL37|MRPL24|MRPL21|RPL5|ASS1|VARS|PDHA1|HAGH|GGT5|GGT1|UBA52|LARS2|MRPS22|GFM1|ACSL1|RPL18	2.68E-05	GO.0043604	0.33872161
52	9	GO Process	cofactor catabolic process	0.00044	AHCY|PRDX1|AMBP|PRDX5|VCP|GPX3|GGT5|GGT1|GPX1	2.87E-05	GO.0051187	0.33565473
18	6	GO Process	nucleobase biosynthetic process	0.00044	MTHFD1|HPRT1|SHMT1|CMPK1|PRPS1|APRT	2.88E-05	GO.0046112	0.33565473
18	6	GO Process	2-oxoglutarate metabolic process	0.00044	OGDH|GOT2|IDH2|DLST|GOT1|IDH1	2.88E-05	GO.0006103	0.33565473
30	8	KEGG Pathways	Pentose phosphate pathway	3E-05	TALDO1|PFKM|PRPS1|ALDOB|ALDOA|TKT|GPI|FBP1	5.26E-06	hsa00030	0.45243288
649	37	GO Process	protein localization to organelle	0.00048	ATP6V1D|ECH1|FIS1|EHHADH|PMPCB|RPS16|RAB11A|PACSIN2|SCARB2|RAB7A|SLC27A2|RPS9|RAB6A|TOLLIP|PIPOX|DHRS4|LAMP1|ACAA1|LAMTOR4|VAPA|AGK|UBE2D3|RPL5|SCP2|HMGCL|AKR1C3|SLC25A6|UBA52|IDH1|LAMP2|TOMM40|NDUFA13|EPHX2|PHB2|RAN|RPL18|HAO2	3.15E-05	GO.0033365	0.33187588
1106	54	GO Process	cellular response to endogenous stimulus	0.00049	GNA11|ATP6V1D|CTSH|FIS1|ATP6V1B1|VIL1|ATP6V1E1|CDH1|ATP6V0A1|IQGAP1|ATP6V1A|ATP6V1B2|LAMTOR1|CA2|ATP6V0D2|CAPN2|GPD1|YWHAG|ATP6V0A4|AQP1|HNRNPD|SHMT1|PCK1|PKM|HNRNPM|ATP6V0C|PKLR|LAMTOR4|CTSB|UBE2D3|ATP6V1H|MSN|COL4A2|OPA1|COL6A1|GOT1|ASS1|RAB14|ATP6V1G1|COL4A1|TBC1D4|APRT|AKR1C3|FECH|PRKAR1A|PRKCD|UBA52|RHOA|DDC|AOC1|ACSL1|ATP1A1|RAN|PHB	3.29E-05	GO.0071495	0.33098039
98	12	GO Process	cellular monovalent inorganic cation homeostasis	0.00049	SLC4A1|ATP6V0A1|RAB7A|CA2|ATP6V0D2|ATP6V0A4|ATP6V0C|ATP6V1H|ATP1B1|CLIC4|SLC4A4|ATP1A1	3.25E-05	GO.0030004	0.33098039
98	12	GO Process	ribonucleoside metabolic process	0.00049	AHCY|HPRT1|AHCYL2|PNP|OPA1|CMPK1|APRT|NME2|AK4|AMPD3|RHOA|RAN	3.25E-05	GO.0009119	0.33098039
82	11	GO Process	insulin receptor signaling pathway	0.00049	ATP6V1D|ATP6V1B1|ATP6V1E1|ATP6V0A1|ATP6V1A|ATP6V1B2|ATP6V0D2|ATP6V0A4|ATP6V0C|ATP6V1H|ATP6V1G1	3.24E-05	GO.0008286	0.33098039

<table>
<tr><td>5342</td><td>187</td><td>GO Process</td><td>cellular component organization or biogenesis</td><td>0.00052</td><td>RALA|DCN|HSD17B10|NDUFB4|VCL|MYH9|ATP6V1D|EHD4|ECH1|FIS1|PPIF|ACOT13|EHHADH|ACADL|APOA1|TTR|PFN2|TFG|VIL1|PMPCB|RPS16|NDUFA2|ACTN4|NDUFA10|LAMA5|APCS|SYNJ2BP|RAB21|HEXB|PSMD11|CDH1|RAB11A|DNAJA3|CHCHD3|SLC9A3R1|RAB2A|PACSIN2|NAPA|LRP2|CAPG|RAB17|ATP6V0A1|ADD1|SCARB2|RAB7A|CPT1A|NDUFA9|SLC27A2|IQGAP1|AFG3L2|ECSIT|DPYS|NDUFB9|LAMTOR1|SLC25A4|UCHL1|ACAA2|TBC1D24|APEH|SCIN|HSPA9|COL14A1|CFL2|HPRT1|NDUFB8|TPP1|COL6A2|RPS9|TMED10|DCXR|GAA|YWHAG|CYCS|ATP6V0A4|DPYSL2|RAB1B|AQP1|DAB2|GRHPR|TLN1|GLS|PIPOX|SHMT1|PKM|RHOT2|NDUFV1|DHRS4|EHD3|AP2A2|ATP6V0C|NDUFA12|TMED9|UQCR10|SERPINA5|ACAA1|SHMT2|BSG|PARVA|LAMTOR4|EPB41L3|APOD|VAPA|PFKM|ANXA6|AGK|COL18A1|PLS3|UBE2D3|VCP|MSN|MYH10|COL4A2|LONP1|MRPL37|MRPL24|MRPL21|OPA1|COA3|KRT19|COL6A1|IBA57|ATP1B1|DPT|NDUFS2|PHGDH|FLNA|ABCD3|RPL5|SCP2|SLC25A5|APOOL|GSN|RAB14|CLIC4|HMGCL|HSPG2|ALDOB|COL4A1|KCTD12|HIST1H2AC|NAP1L4|SLC25A6|CD9|GPX3|KRT18|PRKAR1A|CNP|ACTN1|PRKCD|ALDOA|QPRT|CD59|MGST1|MYH11|ILK|ATP5A1|SEPT7|ELAVL1|IMMT|UBA52|IDH1|NDUFS1|GDI1|CRYZ|RHOA|FBP1|LAMP2|TOMM40|SERPINA1|TGFBI|VWA1|MRPS22|PPIA|SSBP1|FLNB|TMEM33|NDUFA13|WDR1|EPHX2|PHB2|RAN|KRT8|TWF1|CIRBP|DDX6|PHB|HAO2</td><td>3.49E-05</td><td>GO.0071840</td><td>0.32839967</td></tr>
<tr><td>212</td><td>18</td><td>GO Function</td><td>transferase activity, transferring acyl groups</td><td>0.00054</td><td>GNPNAT1|PDHX|F13A1|CPT1A|NAT8|DLAT|ACAA2|GLYATL1|ACSM2B|ACAA1|DLST|CS|CPT2|SCP2|GGT5|GGT1|ACSM2A|GLYAT</td><td>0.00004</td><td>GO.0016746</td><td>0.32676062</td></tr>
<tr><td>83</td><td>11</td><td>GO Process</td><td>mitochondrial transmembrane transport</td><td>0.00054</td><td>CPT1A|AFG3L2|CYC1|AGK|OPA1|CPT2|ATP5A1|TST|SLC25A12|TOMM40|NDUFA13</td><td>3.58E-05</td><td>GO.1990542</td><td>0.32676062</td></tr>
<tr><td>6</td><td>4</td><td>GO Component</td><td>oxoglutarate dehydrogenase complex</td><td>0.00055</td><td>OGDH|DHTKD1|DLST|OGDHL</td><td>8.73E-05</td><td>GO.0045252</td><td>0.32596373</td></tr>
<tr><td>41</td><td>8</td><td>GO Process</td><td>inner mitochondrial membrane organization</td><td>0.00056</td><td>CHCHD3|AFG3L2|AGK|OPA1|APOOL|ATP5A1|IMMT|NDUFA13</td><td>3.77E-05</td><td>GO.0007007</td><td>0.3251812</td></tr>
<tr><td>30</td><td>7</td><td>GO Function</td><td>acid-thiol ligase activity</td><td>0.00058</td><td>SLC27A2|ACSS1|ACSM2B|ACSF2|ACSL1|ACSS3|ACSM2A</td><td>4.32E-05</td><td>GO.0016878</td><td>0.3236572</td></tr>
<tr><td>155</td><td>15</td><td>GO Function</td><td>ligase activity</td><td>0.00059</td><td>MTHFD1|NARS|SLC27A2|UCHL1|ACSS1|ACSM2B|WARS|ASS1|VARS|PC|ACSF2|LARS2|ACSL1|ACSS3|ACSM2A</td><td>4.46E-05</td><td>GO.0016874</td><td>0.3229148</td></tr>
<tr><td>413</td><td>27</td><td>GO Function</td><td>actin binding</td><td>0.00059</td><td>VCL|MYH9|PFN2|VIL1|ACTN4|COTL1|CAPG|ADD1|SCIN|CFL2|TLN1|PARVA|EPB41L3|PLS3|MSN|MYH10|FLNA|MYO6|GSN|ACTN1|ALDOA|MYH11|FLNB|WDR1|TAGLN|TWF1|PDLIM5</td><td>4.45E-05</td><td>GO.0003779</td><td>0.3229148</td></tr>
<tr><td>62</td><td>9</td><td>GO Component</td><td>specific granule lumen</td><td>0.00061</td><td>VCL|LTF|CTSD|GGH|LYZ|TOLLIP|ACAA1|NIT2|AOC1</td><td>9.77E-05</td><td>GO.0035580</td><td>0.32146702</td></tr>
<tr><td>11</td><td>5</td><td>GO Process</td><td>oxaloacetate metabolic process</td><td>0.00063</td><td>GOT2|PCK1|MDH2|GOT1|NIT2</td><td>4.22E-05</td><td>GO.0006107</td><td>0.32006595</td></tr>
</table>

4792	168	GO Component	protein-containing complex	0.00065	PSMA4|DCN|GNA11|HSD17B10|NDUFB4|VCL|MYH9|ATP6V1D|TXNL1|MYL12A|PSMD7|CTSH|HNRNPL|CDC37|OGDH|FIS1|PPIF|PDHX|LTF|ATP6V1B1|ENO1|APOA1|TTR|GOT2|CANX|HSPB1|PMPCB|RPS16|NDUFA2|ACTN4|NDUFA10|LAMA5|ATP6V1E1|LGALS3|GRSF1|PSMD11|CDH1|RAB11A|CHCHD3|DHTKD1|NAPA|LRP2|CAPG|PSMD3|ATP6V0A1|ADD1|VDAC1|NDUFA9|IQGAP1|AFG3L2|BCKDHA|PSMA5|ATP6V1A|FTH1|ATP6V1B2|ERLIN2|NDUFB9|LAMTOR1|DLAT|GPD1L|ATP6V0D2|PSMB4|AGL|FABP1|SCIN|COL14A1|AMN|NDUFB8|COL6A2|GPD1|RPS9|TMED10|HINT1|GNB2|PDHB|CYCS|ATP6V0A4|GPD2|UQCRH|HNRNPD|TOLLIP|CYC1|PKM|NDUFV1|HNRNPM|AP2A2|COPB2|ATP6V0C|NDUFA12|UQCR10|SERPINA5|SHMT2|DLST|LAMTOR4|APOD|PFKM|SND1|WARS|AGK|COL18A1|VCP|ATP6V1H|MYH10|COL4A2|MRPL37|MRPL24|MRPL21|KIF21A|KRT19|COL6A1|ATP1B1|NDUFS2|FLNA|MYO6|RPL5|RBP4|SCP2|SLC25A5|PRPS1|APOOL|GSN|ATP6V1G1|OGDHL|CLIC4|HNRNPR|HSPG2|PSMB9|PSMB8|CLIC1|COL4A1|HIST1H2AC|CKAP4|PDHA1|GLDC|SLC25A6|SACM1L|PRKAR1A|MYH11|ABAT|ILK|ATP5A1|SEPT7|ELAVL1|PDIA6|IMMT|UBA52|NDUFS1|GDI1|HIGD1A|LAMP2|TOMM40|MRPS22|PPIA|TMEM33|NDUFA13|ETFDH|WDR1|KIF13B|PHB2|ATP1A1|RAN|RPL18|KRT8|CIRBP|EML2|DDX6|VPS29|PSMC3	0.00011	GO.0032991	0.31870866
196	17	GO Function	magnesium ion binding	0.00066	IDH3G|ENO1|HPRT1|IDH3A|PCK1|PKM|IDH2|PKLR|PPA2|OPA1|LHPP|PRPS1|HMGCL|IDH3B|IDH1|EPHX2|RAN	5.09E-05	GO.0000287	0.31804561
321	23	GO Process	response to oxygen levels	0.00066	AHCY|FIS1|ACTN4|ATP6V1A|ACAA2|CAPN2|FABP1|AQP1|HSD11B2|PCK1|PKM|PKLR|DPP4|LONP1|OPA1|ATP1B1|ATP6V1G1|ABAT|UBA52|HIGD1A|RHOA|FAM162A|PHB2	4.44E-05	GO.0070482	0.31804561
57	9	GO Function	amino acid binding	0.00069	GOT2|DPYS|GLUD1|DDAH1|SHMT1|SHMT2|ASS1|GLDC|DDC	5.45E-05	GO.0016597	0.31611509
20	6	GO Process	methionine metabolic process	0.0007	MTHFD1|AHCY|BHMT2|BHMT|MSRA|AHCYL2	4.71E-05	GO.0006555	0.3154902
195	17	GO Process	cellular response to toxic substance	0.00071	TXNL1|FIS1|PPIF|GSTM3|PRDX1|NNT|PRDX5|FABP1|AQP1|GSTA1|MGST3|GSTO1|GPX3|PRKCD|MGST1|TXNRD2|GPX1	0.000048	GO.0097237	0.31487417
2219	91	GO Process	cellular response to organic substance	0.00071	RALA|DCN|GNA11|GNPNAT1|ATP6V1D|CTSH|EHD4|FIS1|ATP6V1B1|CANX|VIL1|HSPB1|LAMA5|ATP6V1E1|CDH1|ATP6V0A1|ADD1|F13A1|CALB1|CPT1A|IQGAP1|ATP6V1A|ATP6V1B2|LAMTOR1|CA2|ATP6V0D2|CAPN2|HSPA9|TPP1|GPD1|YWHAG|ATP6V0A4|AQP1|HNRNPD|TOLLIP|TLN1|SHMT1|PCK1|PKM|TALDO1|BCAT2|HNRNPM|ATP6V0C|RAB11B|PKLR|LAMTOR4|CTSB|UBE2D3|VCP|ATP6V1H|MSN|COL4A2|OPA1|COL6A1|GSTO1|GOT1|SLC25A5|ASS1|GSN|RAB14|ATP6V1G1|PSMB8|ASPN|COL4A1|TBC1D4|APRT|AKR1C3|GLDC|FECH|KRT18|PRKAR1A|PRKCD|PSAP|ILK|IFI30|PDIA6|UBA52|RHOA|DDC|AOC1|MME|PPIA|GSTA2|FLNB|ACSL1|NDUFA13|PHB2|ATP1A1|RAN|KRT8|PHB	4.83E-05	GO.0071310	0.31487417
36	7	GO Component	integral component of mitochondrial inner membrane	0.00072	CHCHD3|AFG3L2|AGK|COA3|APOOL|IMMT|ETFDH	0.00012	GO.0031305	0.31426675
21	6	GO Function	oxidoreductase activity, acting on the CH-NH2 group of donors	0.00074	CRYM|GLUD1|AOC3|MAOB|GLDC|AOC1	5.92E-05	GO.0016638	0.31307683
21	6	GO Function	transaminase activity	0.00074	AGXT2|GOT2|BCAT2|GOT1|PSAT1|ABAT	5.92E-05	GO.0008483	0.31307683
44	8	GO Function	aminopeptidase activity	0.00074	CTSH|PEPD|ENPEP|RNPEP|ANPEP|DPP4|XPNPEP2|DPEP1	5.86E-05	GO.0004177	0.31307683
31	7	GO Process	peptide catabolic process	0.00076	CTSH|ENPEP|RNPEP|TPP1|ANPEP|GGT5|GGT1	5.17E-05	GO.0043171	0.31191864
81	16	KEGG Pathways	Peroxisome	6.1E-08	ECH1|EHHADH|PRDX1|PRDX5|SLC27A2|PIPOX|DHRS4|IDH2|ACAA1|ABCD3|SCP2|HMGCL|IDH1|ACSL1|EPHX2|HAO2	4.59E-09	hsa04146	0.72182446

5	4	GO Process	carnitine biosynthetic process	0.00078	BBOX1|SHMT1|ALDH9A1|ACADM	5.35E-05	GO.0045329	0.31079054
418	27	GO Process	actin cytoskeleton organization	0.00078	RALA|MYH9|PFN2|VIL1|ACTN4|SLC9A3R1|PACSIN2|ADD1|SCIN|CFL2|AQP1|TLN1|PARVA|EPB41L3|PLS3|MYH10|KRT19|FLNA|GSN|PRKAR1A|ACTN1|ALDOA|MYH11|RHOA|FLNB|WDR1|KRT8	5.39E-05	GO.0030036	0.31079054
5	4	GO Process	glucuronate catabolic process to xylulose 5-phosphate	0.00078	SORD|CRYL1|DCXR|AKR1A1	5.35E-05	GO.0019640	0.31079054
104	12	GO Process	long-chain fatty acid metabolic process	0.00079	ACADL|CPT1A|SLC27A2|CBR1|CYP4A11|ACAA1|GSTA1|PTGES2|SCP2|AKR1C3|ACSL1|EPHX2	5.51E-05	GO.0001676	0.31023729
7	4	GO Component	alveolar lamellar body	0.00081	CTSH|NAPSA|RAB7A|LAMP1	0.00013	GO.0097208	0.3091515
493	30	GO Process	actin filament-based process	0.00084	RALA|MYH9|PFN2|VIL1|ACTN4|SLC9A3R1|PACSIN2|ADD1|GPD1L|SCIN|CFL2|AQP1|TLN1|PARVA|EPB41L3|PLS3|MYH10|KRT19|FLNA|MYO6|GSN|PRKAR1A|ACTN1|ALDOA|MYH11|RHOA|FLNB|WDR1|ATP1A1|KRT8	5.91E-05	GO.0030029	0.30757207
12	5	GO Process	purine nucleobase biosynthetic process	0.00084	MTHFD1|HPRT1|SHMT1|PRPS1|APRT	5.85E-05	GO.0009113	0.30757207
21	6	GO Process	purine nucleobase metabolic process	0.00084	MTHFD1|GDA|HPRT1|SHMT1|PRPS1|APRT	5.92E-05	GO.0006144	0.30757207
12	5	GO Process	mitochondrial electron transport, ubiquinol to cytochrome c	0.00084	PMPCB|CYCS|UQCRH|CYC1|UQCR10	5.85E-05	GO.0006122	0.30757207
32	7	GO Process	intracellular pH reduction	0.00087	ATP6V0A1|RAB7A|ATP6V0D2|ATP6V0A4|ATP6V0C|ATP6V1H|CLIC4	6.16E-05	GO.0051452	0.30604807
58	9	GO Process	negative regulation of protein polymerization	0.00087	PFN2|VIL1|CAPG|ADD1|SCIN|GSN|PRKCD|TWF1|EML2	6.15E-05	GO.0032272	0.30604807
45	8	GO Process	negative regulation of actin filament polymerization	0.00095	PFN2|VIL1|CAPG|ADD1|SCIN|GSN|PRKCD|TWF1	6.75E-05	GO.0030837	0.30222764
1295	58	GO Component	catalytic complex	0.00096	PSMA4|GNA11|HSD17B10|NDUFB4|TXNL1|PSMD7|OGDH|PDHX|ENO1|HSPB1|PMPCB|NDUFA2|NDUFA10|PSMD11|DHTKD1|PSMD3|NDUFA9|AFG3L2|BCKDHA|PSMA5|NDUFB9|DLAT|GPD1L|PSMB4|AGL|NDUFB8|GPD1|HINT1|PDHB|CYCS|GPD2|UQCRH|CYC1|PKM|NDUFV1|HNRNPM|NDUFA12|UQCR10|DLST|PFKM|VCP|ATP1B1|NDUFS2|MYO6|PRPS1|OGDHL|HNRNPR|PSMB9|PSMB8|PDHA1|GLDC|PRKAR1A|ABAT|NDUFS1|NDUFA13|ETFDH|ATP1A1|PSMC3	0.00016	GO.1902494	0.30177288
649	36	GO Process	epithelial cell differentiation	0.00096	DNPH1|CAPNS1|VIL1|LAMA5|LGALS3|GSTM3|SLC9A3R1|VDAC1|CPT1A|RHCG|IQGAP1|CBR1|TPP1|DAB2|TOLLIP|KRT7|GSTA1|CTSB|COL18A1|MSN|KRT19|TAGLN2|FLNA|CLIC4|COL4A1|AKR1C3|KRT18|PSAP|TST|RHOA|GPX1|GSTA2|FLNB|CAPN1|TAGLN|KRT8	6.84E-05	GO.0030855	0.30177288
286	21	GO Process	regulation of autophagy	0.00096	DCN|ATP6V1D|CDC37|ATP6V1B1|CAPNS1|HSPB1|ATP6V1E1|ATP6V0A1|VDAC1|ATP6V1A|ATP6V1B2|LAMTOR1|UCHL1|ATP6V0D2|RAB1B|ATP6V0C|LAMTOR4|ATP6V1H|ATP6V1G1|PSAP|CAPN1	6.82E-05	GO.0010506	0.30177288
13	5	GO Function	omega peptidase activity	0.00097	GGH|UCHL1|APEH|GGT5|GGT1	7.93E-05	GO.0008242	0.30132283

2556	101	GO Process	cellular component biogenesis	0.00099	HSD17B10|NDUFB4|VCL|ATP6V1D|EHD4|FIS1|ACOT13|ACADL|APOA1|TFG|VIL1|RPS16|NDUFA2|ACTN4|NDUFA10|LAMA5|APCS|PSMD11|CDH1|RAB11A|SLC9A3R1|PACSIN2|NAPA|CAPG|RAB17|ATP6V0A1|ADD1|RAB7A|CPT1A|NDUFA9|ECSIT|DPYS|NDUFB9|HSPA9|CFL2|HPRT1|NDUFB8|COL6A2|RPS9|TMED10|DCXR|YWHAG|ATP6V0A4|RAB1B|GRHPR|TLN1|GLS|SHMT1|PKM|NDUFV1|DHRS4|EHD3|NDUFA12|UQCR10|SHMT2|PARVA|EPB41L3|PFKM|ANXA6|PLS3|VCP|MYH10|LONP1|OPA1|COA3|KRT19|COL6A1|IBA57|NDUFS2|FLNA|RPL5|GSN|HMGCL|ALDOB|KCTD12|NAP1L4|CD9|GPX3|PRKAR1A|ACTN1|ALDOA|QPRT|CD59|MGST1|MYH11|ILK|SEPT7|ELAVL1|UBA52|NDUFS1|CRYZ|RHOA|FBP1|SERPINA1|TMEM33|NDUFA13|WDR1|RAN|KRT8|CIRBP|DDX6	7.07E-05	GO.0044085	0.30043648
1900	78	GO Component	plasma membrane bounded cell projection	0.001	GNA11|MYH9|AHCY|REEP6|ATP6V1B1|PFN2|CANX|VIL1|HSPB1|ACTN4|ATP6V1E1|GSTM3|RAB21|CDH1|RAB11A|SLC9A3R1|PACSIN2|LRP2|RAB17|CALB1|SORD|IQGAP1|ATP6V1A|ATP6V1B2|HNMT|QDPR|UCHL1|CA2|TBC1D24|CAPN2|AMN|CKB|DCXR|ATP6V0A4|AQP1|AOC3|AHCYL2|TLN1|PKM|EHD3|LAMP1|EPB41L3|APOD|PFKM|MSN|MYH10|DPP4|OPA1|FLNA|MYO6|GOT1|ACADM|ASS1|GSN|CLIC4|HNRNPR|CUBN|PRKAR1A|DPEP1|NME2|CNP|ACTN1|UMOD|ABAT|ILK|MPST|SEPT7|GDI1|PDZK1|RHOA|DDC|MME|KIF13B|PHB2|TWF1|NDRG2|DDX6|PDLIM5	0.00018	GO.0120025	0.3
34	7	GO Function	carboxy-lyase activity	0.001	PCK2|BCKDHA|PCK1|GOT1|DDC|ECHDC1|ME3	8.61E-05	GO.0016831	0.3
6	4	GO Function	epoxide hydrolase activity	0.001	AKR7A2|RNPEP|EPHX2|EPHX1	8.73E-05	GO.0004301	0.3
33	7	GO Process	pteridine-containing compound metabolic process	0.001	MTHFD1|GGH|QDPR|FTCD|PIPOX|SHMT1|SHMT2	0.000073	GO.0042558	0.3
22	6	GO Process	vacuolar acidification	0.001	ATP6V0A1|ATP6V0D2|ATP6V0A4|ATP6V0C|ATP6V1H|CLIC4	7.37E-05	GO.0007035	0.3
288	21	GO Process	response to hypoxia	0.001	AHCY|FIS1|ACTN4|ACAA2|CAPN2|FABP1|AQP1|HSD11B2|PCK1|PKM|PKLR|DPP4|LONP1|OPA1|ATP1B1|ABAT|UBA52|HIGD1A|RHOA|FAM162A|PHB2	7.49E-05	GO.0001666	0.3
8	4	GO Component	pyruvate dehydrogenase complex	0.0011	PDHX|DLAT|PDHB|PDHA1	0.0002	GO.0045254	0.29586073
1969	80	GO Component	cell projection	0.0011	GNA11|MYH9|AHCY|REEP6|ATP6V1B1|PFN2|CANX|VIL1|HSPB1|ACTN4|ATP6V1E1|GSTM3|RAB21|CDH1|RAB11A|SLC9A3R1|PACSIN2|LRP2|RAB17|CALB1|SORD|IQGAP1|ATP6V1A|ATP6V1B2|HNMT|QDPR|UCHL1|CA2|TBC1D24|CAPN2|SCIN|AMN|CKB|DCXR|ATP6V0A4|AQP1|AOC3|AHCYL2|TLN1|PKM|EHD3|LAMP1|EPB41L3|APOD|PFKM|MSN|MYH10|DPP4|OPA1|FLNA|MYO6|GOT1|ACADM|ASS1|GSN|CLIC4|HNRNPR|CUBN|PRKAR1A|DPEP1|NME2|CNP|ACTN1|UMOD|ABAT|ILK|MPST|SEPT7|GDI1|PDZK1|RHOA|DDC|MME|WDR1|KIF13B|PHB2|TWF1|NDRG2|DDX6|PDLIM5	0.0002	GO.0042995	0.29586073

8420	269	GO Component	membrane	0.0011	RALA|SYPL1|GNA11|HSD17B10|CA12|NDUFB4|VCL|NIPSNAP1|MYH9|GNPNAT1|ATP6V1D|APMAP|F9|EHD4|PLIN3|OGDH|FIS1|SFXN3|PPIF|SLC25A3|REEP6|ACADL|ATP6V1B1|ENO1|CTSD|APOA1|TFG|GOT2|CAPNS1|CANX|VIL1|HSPB1|PMPCB|NDUFA2|NDUFA10|ATP6V1E1|LGALS3|SYNJ2BP|ARL8B|SQRDL|RAB21|CDH1|RAB11A|DNAJA3|SLC4A1|CHCHD3|SLC9A3R1|RAB2A|PACSIN2|NAPA|EPCAM|LRP2|GRB14|RAB17|ATP6V0A1|NNT|ADD1|SCARB2|ACAD11|RAB7A|AMBP|ENPEP|VDAC1|CPT1A|NDUFA9|SORD|SLC27A2|RHCG|IQGAP1|AFG3L2|BCAM|ECSIT|ARL8A|NAT8|ATP6V1A|ATP6V1B2|ERLIN2|NDUFB9|LAMTOR1|SLC25A4|GPD1L|UCHL1|CA2|ATP6V0D2|HPD|FTCD|TBC1D24|CAPN2|RNPEP|APEH|SCIN|AMN|NDUFB8|CDH16|ANPEP|COL6A2|PRODH2|SLC23A1|TMED10|DCXR|HINT1|UGT2B7|GNB2|GAA|ADH1B|TMEM126A|CYCS|ATP6V0A4|GPD2|DPYSL2|UQCRH|RAB1B|CYP4A11|AQP1|RAB6A|AOC3|GBAS|DAB2|MSRA|TOLLIP|TLN1|HSD11B2|SFXN1|CYC1|CHDH|SLC7A8|UGT2B17|RHOT2|NDUFV1|DHRS4|MDH2|EHD3|AP2A2|SLC5A2|COPB2|ATP6V0C|NDUFA12|TMED9|UQCR10|SERPINA5|LAMP1|RAB11B|SHMT2|BSG|PARVA|CYB5A|LAMTOR4|EPB41L3|PTGES2|VAPA|PFKM|ALDH3A2|ANXA6|UGT1A9|AGK|PLS3|GM2A|UBE2D3|VCP|ATP6V1H|MSN|MYH10|DPP4|MRPL37|MRPL24|MRPL21|OPA1|COA3|KIF21A|KRT19|COL6A1|MARC2|CYB5R1|ATP1B1|MGST3|NDUFS2|FLNA|SFXN2|MYO6|ABCD3|ACADM|XPNPEP2|CPT2|SLC25A5|AKR1A1|ASS1|APOOL|GSN|RAB14|ATP6V1G1|CLIC4|HSPG2|CLIC1|KCTD12|CUBN|SLC3A2|CKAP4|MAOB|BPHL|GLDC|SLC25A6|GPNMB|CD9|FECH|SACM1L|BDH1|PRKAR1A|DPEP1|CNP|FHL1|ACTN1|PRKCD|PSAP|KTN1|CD59|UMOD|MGST1|GATM|ILK|GGT5|ATP5A1|SEPT7|GGT1|PDIA6|IMMT|UBA52|SLC25A12|SCRN1|NDUFS1|SLC4A4|PDZK1|HIGD1A|RHOA|GK|TMED4|CKMT2|GPI|CKMT1A|LAMP2|TOMM40|AOC1|TOM1|SERPINA1|TGFBI|CFB|MRPS22|MME|FAM162A|FLNB|TMEM33|ACSL1|NDUFA13|ETFDH|WDR1|CAPN1|PHB2|ATP1A1|SLC25A10|MGAM|CD63|KRT8|TWF1|SLC43A2|PHB|EPHX1|PDLIM5|VPS29|FMO1|PLCG2	0.00018	GO.0016020	0.29586073
13	5	GO Process	dicarboxylic acid biosynthetic process	0.0011	MTHFD1|GOT2|GLUD1|GLS|GOT1	7.93E-05	GO.0043650	0.29586073
13	5	GO Process	glucan catabolic process	0.0011	PYGB|AGL|GAA|PFKM|MGAM	7.93E-05	GO.0009251	0.29586073
13	5	GO Process	acetyl-CoA biosynthetic process	0.0011	PDHX|DLAT|PDHB|ACSS1|PDHA1	7.93E-05	GO.0006085	0.29586073
23	6	GO Process	cellular biogenic amine catabolic process	0.0012	DMGDH|BHMT|HNMT|CHDH|SARDH|ALDH7A1	9.08E-05	GO.0042402	0.29208188
17	5	GO Component	pseudopodium	0.0013	ACTN4|CAPN2|MSN|CNP|ACTN1	0.00022	GO.0031143	0.28860566
1796	74	GO Component	endoplasmic reticulum	0.0013	APMAP|F9|EHD4|FIS1|REEP6|APOA1|TFG|CANX|RAB21|RAB2A|LRP2|SCARB2|SLC27A2|NAT8|ERLIN2|UCHL1|HPD|FTCD|AGL|CAPN2|COL14A1|NDUFB8|COL6A2|TMED10|UGT2B7|ACO1|RAB1B|CYP4A11|RAB6A|AOC3|AHCYL2|HSD11B2|CES2|UGT2B17|DHRS4|COPB2|TMED9|CYB5A|APOD|VAPA|ALDH3A2|UGT1A9|COL18A1|VCP|COL4A2|COL6A1|CYB5R1|MGST3|RPL5|ASS1|RAB14|HNRNPR|COL4A1|CUBN|CKAP4|TXNDC5|SACM1L|PRKCD|KTN1|CD59|MGST1|PDIA6|UBA52|RHOA|TMED4|SERPINA1|VWA1|TMEM33|ACSL1|ATP1A1|RPL18|NPC2|EPHX1|FMO1	0.00024	GO.0005783	0.28860566
62	9	GO Process	pigment metabolic process	0.0013	MTHFD1|AMBP|HPRT1|SHMT1|UGT1A9|IBA57|PRPS1|APRT|FECH	9.77E-05	GO.0042440	0.28860566
14	5	GO Process	barbed-end actin filament capping	0.0014	VIL1|CAPG|ADD1|GSN|TWF1	0.00011	GO.0051016	0.2853872

14	5	GO Process	cellular polysaccharide catabolic process	0.0014	PYGB|AGL|GAA|PFKM|MGAM	0.00011	GO.0044247	0.2853872
14	5	GO Process	cysteine metabolic process	0.0014	MTHFD1|AHCY|AHCYL2|MPST|GGT1	0.00011	GO.0006534	0.2853872
79	10	GO Process	regulation of intracellular pH	0.0015	SLC4A1|ATP6V0A1|RAB7A|CA2|ATP6V0D2|ATP6V0A4|ATP6V0C|ATP6V1H|CLIC4|SLC4A4	0.00011	GO.0051453	0.28239087
49	8	GO Process	regulation of actin filament depolymerization	0.0015	VIL1|CAPG|ADD1|SCIN|CFL2|GSN|WDR1|TWF1	0.00011	GO.0030834	0.28239087
24	6	GO Process	one-carbon metabolic process	0.0015	MTHFD1|AHCY|FTCD|AHCYL2|SHMT1|SHMT2	0.00011	GO.0006730	0.28239087
18	5	GO Component	lamellar body	0.0016	CTSH|NAPSA|RAB7A|LAMP1|CKAP4	0.00028	GO.0042599	0.279588
9	4	GO Component	proton-transporting V-type ATPase, V1 domain	0.0016	ATP6V1B1|ATP6V1A|ATP6V1B2|ATP6V1H	0.00028	GO.0033180	0.279588
7	4	GO Function	thyroid hormone binding	0.0016	CRYM|CTSH|TTR|PKM	0.00013	GO.0070324	0.279588
1524	66	GO Function	adenyl nucleotide binding	0.0016	MYH9|MTHFD1|AHCY|IDH3G|EHD4|ACADL|ATP6V1B1|NARS|KHK|DNAJA3|SLC27A2|AFG3L2|ATP6V1A|ATP6V1B2|GLUD1|HSPA9|CKB|ACSS1|PKM|EHD3|ACSM2B|PKLR|PFKM|WARS|AGK|UBE2D3|VCP|MYH10|LONP1|KIF21A|MYO6|ABCD3|SCP2|CMPK1|ASS1|PRPS1|HMGCL|VARS|APRT|PRKAR1A|NME2|PC|PRKCD|DAK|AK4|MYH11|ILK|ATP5A1|GK|ACSF2|CKMT2|CKMT1A|FBP1|LARS2|ACSL1|NDUFA13|KIF13B|ATP1A1|MMAB|TWF1|ACSS3|ALDH6A1|ACSM2A|GALK1|DDX6|PSMC3	0.00014	GO.0030554	0.279588
25	6	GO Function	glutathione transferase activity	0.0016	GSTM3|GSTA1|MGST3|GSTO1|MGST1|GSTA2	0.00013	GO.0004364	0.279588
25	6	GO Function	aldo-keto reductase (NADP) activity	0.0016	AKR7A2|DHRS4|CYB5A|AKR7A3|AKR1A1|AKR1C3	0.00013	GO.0004033	0.279588
230	17	GO Component	cell cortex	0.0017	VCL|MYH9|ENO1|ACTN4|SLC4A1|CAPN2|FABP1|SCIN|MYH10|KRT19|FLNA|MYO6|GSN|SEPT7|RHOA|FLNB|WDR1	0.00031	GO.0005938	0.27695511
145	15	KEGG Pathways	Phagosome	0.00011	ATP6V1D|ATP6V1B1|CANX|ATP6V1E1|ATP6V0A1|RAB7A|ATP6V1A|ATP6V1B2|ATP6V0D2|ATP6V0A4|ATP6V0C|LAMP1|ATP6V1H|ATP6V1G1|LAMP2	2.19E-05	hsa04145	0.39586073
1055	50	GO Process	epithelium development	0.0017	RALA|VCL|MTHFD1|CTSH|DNPH1|CAPNS1|VIL1|LAMA5|LGALS3|GRSF1|GSTM3|SLC9A3R1|EPCAM|LRP2|VDAC1|CALB1|CPT1A|RHCG|IQGAP1|CA2|CBR1|TPP1|DAB2|TOLLIP|KRT7|GSTA1|CTSB|COL18A1|MSN|KRT19|TAGLN2|PHGDH|FLNA|CLIC4|COL4A1|AKR1C3|KRT18|PSAP|UMOD|ILK|TST|RHOA|GPX1|GSTA2|FLNB|WDR1|CAPN1|TAGLN|PHB2|KRT8	0.00013	GO.0060429	0.27695511
50	8	GO Process	cellular biogenic amine metabolic process	0.0017	DMGDH|BHMT|HNMT|CHDH|SARDH|AGMAT|ALDH7A1|DDC	0.00013	GO.0006576	0.27695511
15	5	GO Process	fatty acid beta-oxidation using acyl-CoA dehydrogenase	0.0018	ACADL|ACADS|ACAD11|ACADM|ETFDH	0.00014	GO.0033539	0.27447275
7	4	GO Process	glycerol-3-phosphate metabolic process	0.0018	GPD1L|GPD1|GPD2|GK	0.00013	GO.0006072	0.27447275

3	3	GO Component	glycerol-3-phosphate dehydrogenase complex	0.0019	GPD1L|GPD1|GPD2	0.00034	GO.0009331	0.27212464
51	8	GO Process	purine-containing compound catabolic process	0.0019	AHCY|DNPH1|GDA|HPRT1|HINT1|PNP|AMPD3|GPX1	0.00015	GO.0072523	0.27212464
373	24	GO Process	response to oxidative stress	0.0019	NDUFB4|TXNL1|PPIF|PRDX1|PRDX5|FABP1|CYCS|AQP1|HNRNPD|MSRA|NDUFA12|DHRS2|APOD|LONP1|NDUFS2|AKR1C3|GPX3|DPEP1|PRKCD|MGST1|TXNRD2|IDH1|GPX1|ETFDH	0.00015	GO.0006979	0.27212464
16	5	GO Function	quinone binding	0.002	SQRDL|AOC3|NDUFS2|AOC1|ETFDH	0.00018	GO.0048038	0.269897
1301	58	GO Function	protein dimerization activity	0.002	MYH9|PSMD7|CRYM|DNPH1|ENO1|TTR|GOT2|VIL1|HSPB1|ACTN4|HRSP12|GSTM3|HEXB|SLC4A1|GRB14|ADD1|AMBP|PRDX5|QDPR|GPD1L|CAPN2|CRYL1|HPRT1|GPD1|AOC3|GRHPR|SHMT1|VAPA|PFKM|ALDH3A2|ANXA6|UGT1A9|WARS|DPP4|LHPP|FLNA|ABCD3|HOGA1|RBP4|PRPS1|HMGCL|HIST1H2AC|CUBN|MAOB|NQO2|GLDC|ACTN1|PSAP|QPRT|MGST1|ABAT|ELAVL1|IDH1|TKT|AOC1|EPHX2|RAN|GALE	0.00018	GO.0046983	0.269897
16	5	GO Function	acyl-CoA dehydrogenase activity	0.002	ACADL|ACADS|ACAD11|ACADSB|ACADM	0.00018	GO.0003995	0.269897
82	10	GO Process	fatty acid derivative biosynthetic process	0.002	CBR1|PTGES2|MGST3|HMGCL|AKR1C3|BDH1|GGT5|GGT1|ACSL1|ACSS3	0.00015	GO.1901570	0.269897
10	4	GO Component	proton-transporting V-type ATPase, V0 domain	0.0021	ATP6V0A1|ATP6V0D2|ATP6V0A4|ATP6V0C	0.00038	GO.0033179	0.26777807
213	16	GO Component	basolateral plasma membrane	0.0021	ATP6V1B1|SLC4A1|EPCAM|RAB17|RHCG|CA2|CDH16|SLC23A1|AQP1|SLC7A8|GM2A|MSN|ATP1B1|UMOD|SLC4A4|ATP1A1	0.00039	GO.0016323	0.26777807
402	24	GO Component	cell-cell junction	0.0022	VCL|MYH9|ACTN4|CDH1|PACSIN2|EPCAM|ADD1|IQGAP1|EPB41L3|VAPA|DPP4|ATP1B1|FLNA|CLIC4|ACTN1|PRKCD|ILK|RHOA|AOC1|WDR1|ATP1A1|KRT8|TWF1|DDX6	0.00041	GO.0005911	0.26575773
1514	65	GO Function	adenyl ribonucleotide binding	0.0022	MYH9|MTHFD1|IDH3G|EHD4|ACADL|ATP6V1B1|NARS|KHK|DNAJA3|SLC27A2|AFG3L2|ATP6V1A|ATP6V1B2|GLUD1|HSPA9|CKB|ACSS1|PKM|EHD3|ACSM2B|PKLR|PFKM|WARS|AGK|UBE2D3|VCP|MYH10|LONP1|KIF21A|MYO6|ABCD3|SCP2|CMPK1|ASS1|PRPS1|HMGCL|VARS|APRT|PRKAR1A|NME2|PC|PRKCD|DAK|AK4|MYH11|ILK|ATP5A1|GK|ACSF2|CKMT2|CKMT1A|FBP1|LARS2|ACSL1|NDUFA13|KIF13B|ATP1A1|MMAB|TWF1|ACSS3|ALDH6A1|ACSM2A|GALK1|DDX6|PSMC3	0.0002	GO.0032559	0.26575773
8	4	GO Function	nucleobase transmembrane transporter activity	0.0022	SLC25A4|SLC23A1|SLC25A5|SLC25A6	0.0002	GO.0015205	0.26575773
629	34	GO Process	cation homeostasis	0.0022	FIS1|SFXN3|LTF|ATP6V1B1|HEXB|SLC4A1|ATP6V0A1|RAB7A|CALB1|RHCG|AFG3L2|ATP6V1A|FTH1|CA2|ATP6V0D2|ATP6V0A4|ACO1|CYP4A11|SFXN1|SLC7A8|ATP6V0C|ANXA6|ATP6V1H|ATP1B1|GSTO1|SFXN2|GOT1|ATP6V1G1|CLIC4|IMMT|SLC4A4|EPHX2|ATP1A1|PLCG2	0.00017	GO.0055080	0.26575773
77	9	GO Component	cortical cytoskeleton	0.0023	VCL|MYH9|ACTN4|SLC4A1|CAPN2|KRT19|FLNA|GSN|WDR1	0.00043	GO.0030863	0.26382722
113	11	GO Component	endoplasmic reticulum-Golgi intermediate compartment	0.0023	GNPNAT1|RAB2A|NAT8|FTCD|ANPEP|TMED10|RAB1B|TMED9|CD59|PDIA6|SERPINA1	0.00043	GO.0005793	0.26382722
40	7	GO Function	peroxidase activity	0.0023	PRDX1|PRDX5|GSTA1|MGST3|GPX3|MGST1|GPX1	0.00021	GO.0004601	0.26382722

<table>
<tr><td>68</td><td>9</td><td>GO Process</td><td>cell redox homeostasis</td><td>0.0024</td><td>TXNL1|PRDX1|NNT|PRDX5|PTGES2|TXNDC5|TXNRD2|PDIA6|GPX1</td><td>0.00018</td><td>GO.0045454</td><td>0.26197888</td></tr>
<tr><td>39</td><td>7</td><td>GO Process</td><td>aromatic amino acid family metabolic process</td><td>0.0024</td><td>MTHFD1|HNMT|QDPR|HGD|HPD|FTCD|PAH</td><td>0.00018</td><td>GO.0009072</td><td>0.26197888</td></tr>
<tr><td>55</td><td>8</td><td>GO Function</td><td>transferase activity, transferring alkyl or aryl (other than methyl) groups</td><td>0.0025</td><td>NANS|GSTM3|GSTA1|MGST3|GSTO1|MGST1|GSTA2|MMAB</td><td>0.00023</td><td>GO.0016765</td><td>0.260206</td></tr>
<tr><td>4714</td><td>164</td><td>GO Process</td><td>biosynthetic process</td><td>0.0025</td><td>DCN|NANS|GNPNAT1|MTHFD1|PCK2|AHCY|APMAP|OGDH|PDHX|DNPH1|AGXT2|LTF|ENO1|APOA1|GDA|GOT2|RPS16|BHMT2|GSTM3|NARS|HEXB|DNAJA3|CHCHD3|DHTKD1|BBOX1|MECR|ATP6V0A1|ALDH8A1|NDUFA9|SORD|SLC27A2|BHMT|GLUD1|DLAT|QDPR|GPD1L|ACAA2|CBR1|AGL|APEH|CRYL1|HPRT1|GPD1|RPS9|DCXR|HINT1|ASL|PDHB|ATP6V0A4|GPD2|AQP1|GBAS|HNRNPD|TSFM|HSD11B2|ACSS1|CYC1|GLS|SHMT1|UGDH|PCK1|CHDH|PKM|TALDO1|BCAT2|CNDP2|MDH2|ACSM2B|IDH2|SHMT2|GSTA1|PKLR|PPA2|PTGES2|VAPA|PFKM|ALDH3A2|SND1|ALDH9A1|WARS|AGK|PBLD|VCP|COL4A2|MRPL37|MRPL24|PNP|MRPL21|IBA57|CYB5R1|MGST3|LHPP|PHGDH|GSTO1|FLNA|ABCD3|RPL5|GOT1|HOGA1|ACADM|RBP4|SCP2|CMPK1|AKR1A1|ASS1|PRPS1|RAB14|OGDHL|HMGCL|HSPG2|ALDOB|VARS|AGMAT|PSAT1|MAOB|APRT|PDHA1|AKR1C3|FECH|SACM1L|BDH1|BPGM|NME2|CNP|PC|ALDOA|AK4|QPRT|MGST1|AMPD3|ABAT|GATM|MPST|HAGH|GGT5|ATP5A1|GGT1|ALDH7A1|UBA52|SLC25A12|TKT|GK|DDC|GPI|GPX1|FBP1|LARS2|MRPS22|GFM1|PPIA|SSBP1|GSTA2|ACSL1|EPHX2|PHB2|MMAB|SLC25A10|RPL18|PAH|ACSS3|ACSM2A|GALK1|PHB|PLCG2</td><td>0.0002</td><td>GO.0009058</td><td>0.260206</td></tr>
<tr><td>27</td><td>6</td><td>GO Process</td><td>folic acid-containing compound metabolic process</td><td>0.0025</td><td>MTHFD1|GGH|FTCD|PIPOX|SHMT1|SHMT2</td><td>0.00019</td><td>GO.0006760</td><td>0.260206</td></tr>
<tr><td>8</td><td>4</td><td>GO Process</td><td>acetyl-CoA biosynthetic process from pyruvate</td><td>0.0025</td><td>PDHX|DLAT|PDHB|PDHA1</td><td>0.0002</td><td>GO.0006086</td><td>0.260206</td></tr>
<tr><td>609</td><td>33</td><td>GO Process</td><td>import into cell</td><td>0.0026</td><td>MYH9|EHD4|LTF|APOA1|CANX|TINAG|PACSIN2|LRP2|SCARB2|RAB7A|AMBP|SLC27A2|TINAGL1|AMN|DPYSL2|DAB2|AP2A2|ATP6V1H|ATP1B1|MYO6|GSN|HSPG2|CUBN|SLC3A2|TXNDC5|CD9|PRKCD|PDIA6|ANXA11|TOM1|ACSL1|ATP1A1|PLCG2</td><td>0.00021</td><td>GO.0098657</td><td>0.25850267</td></tr>
<tr><td>69</td><td>9</td><td>GO Process</td><td>cellular ketone metabolic process</td><td>0.0026</td><td>AKR7A2|GOT2|NDUFA9|GPD1|GPD2|DHRS4|SCP2|AKR1C3|HAGH</td><td>0.0002</td><td>GO.0042180</td><td>0.25850267</td></tr>
<tr><td>222</td><td>17</td><td>GO Process</td><td>cellular response to oxidative stress</td><td>0.0026</td><td>TXNL1|PPIF|PRDX1|PRDX5|FABP1|CYCS|AQP1|HNRNPD|DHRS2|LONP1|AKR1C3|GPX3|DPEP1|PRKCD|MGST1|TXNRD2|GPX1</td><td>0.00021</td><td>GO.0034599</td><td>0.25850267</td></tr>
<tr><td>21</td><td>5</td><td>GO Component</td><td>proteasome core complex</td><td>0.0027</td><td>PSMA4|PSMA5|PSMB4|PSMB9|PSMB8</td><td>0.00051</td><td>GO.0005839</td><td>0.25686362</td></tr>
<tr><td>63</td><td>8</td><td>GO Component</td><td>actomyosin</td><td>0.0028</td><td>MYH9|ACTN4|MYH10|ACTN1|ILK|SEPT7|FLNB|WDR1</td><td>0.00054</td><td>GO.0042641</td><td>0.2552842</td></tr>
<tr><td>17</td><td>5</td><td>GO Process</td><td>glycerol metabolic process</td><td>0.0028</td><td>GPD2|PCK1|GOT1|DAK|GK</td><td>0.00022</td><td>GO.0006071</td><td>0.2552842</td></tr>
<tr><td>17</td><td>5</td><td>GO Process</td><td>sulfur amino acid biosynthetic process</td><td>0.0028</td><td>MTHFD1|AHCY|BHMT2|BHMT|GGT1</td><td>0.00022</td><td>GO.0000097</td><td>0.2552842</td></tr>
<tr><td>9</td><td>4</td><td>GO Function</td><td>3-hydroxyacyl-CoA dehydrogenase activity</td><td>0.0029</td><td>HSD17B10|EHHADH|CRYL1|HADH</td><td>0.00028</td><td>GO.0003857</td><td>0.2537602</td></tr>
</table>

28	6	GO Process	aromatic amino acid family catabolic process	0.0029	HNMT|QDPR|HGD|HPD|FTCD|PAH	0.00023	GO.0009074	0.2537602
321	21	GO Function	amide binding	0.0031	PPIF|ACADL|APOA1|GSTM3|ENPEP|FTCD|RNPEP|TPP1|ANPEP|SLC7A8|PTGES2|SCP2|HMGCL|NQO2|PC|PSAP|MGST1|MME|PPIA|PHB2|ALDH6A1	0.0003	GO.0033218	0.25086383
5382	182	GO Function	organic cyclic compound binding	0.0033	RALA|HEBP1|GNA11|HSD17B10|MYH9|MTHFD1|PCK2|PYGB|AHCY|IDH3G|CRYM|EHD4|HNRNPL|OGDH|AGXT2|LTF|ACADL|ATP6V1B1|ENO1|APOA1|ACADS|GOT2|RPS16|ACTN4|GRSF1|ARL8B|NARS|TINAG|KHK|RAB21|ALDH2|RAB11A|DNAJA3|RAB2A|DHTKD1|RAB17|NNT|ACAD11|RAB7A|AMBP|CALB1|PRDX5|REXO2|SORD|SLC27A2|AFG3L2|ARL8A|ATP6V1A|ATP6V1B2|ERLIN2|DPYS|GLUD1|ENDOD1|QDPR|GPD1L|FTCD|HSPA9|CRYL1|HPRT1|CKB|IDH3A|GPD1|PRODH2|RPS9|HINT1|CYCS|ACO1|RAB1B|CYP4A11|RAB6A|HNRNPD|GRHPR|TSFM|HSD11B2|ACSS1|CYC1|SHMT1|UGDH|PCK1|CHDH|PKM|RHOT2|NDUFV1|EHD3|ACSM2B|IDH2|RAB11B|SHMT2|PKLR|CYB5A|APOD|PTGES2|PFKM|ANXA6|WARS|AGK|UBE2D3|VCP|MSN|MYH10|LONP1|PNP|OPA1|KIF21A|MARC2|NDUFS2|ACADSB|PHGDH|MYO6|ABCD3|RPL5|GOT1|ACADM|SCP2|CMPK1|ASS1|PRPS1|RAB14|OGDHL|HMGCL|HNRNPR|VARS|AUH|ALDH1B1|HIST1H2AC|CUBN|SLC3A2|MAOB|APRT|NQO2|IDH3B|GLDC|PRKAR1A|NME2|CNP|PC|ACTN1|PRKCD|DAK|AK4|MYH11|ABAT|ILK|ATP5A1|SEPT7|TXNRD2|ELAVL1|TST|IDH1|CRYZ|RHOA|GK|ACSF2|DDC|CKMT2|CKMT1A|FBP1|LARS2|GFM1|SSBP1|ACSL1|NDUFA13|ETFDH|KIF13B|ME3|ATP1A1|MMAB|RAN|RPL18|TWF1|ACSS3|ALDH6A1|NPC2|ACSM2A|GALK1|CIRBP|HADH|DDX6|PHB|PSMC3|FMO1|HAO2	0.00032	GO.0097159	0.24814861
3	3	GO Function	adenine transmembrane transporter activity	0.0034	SLC25A4|SLC25A5|SLC25A6	0.00034	GO.0015207	0.24685211
3	3	GO Function	ATP:ADP antiporter activity	0.0034	SLC25A4|SLC25A5|SLC25A6	0.00034	GO.0005471	0.24685211
19	5	GO Function	glutathione peroxidase activity	0.0034	GSTA1|MGST3|GPX3|MGST1|GPX1	0.00035	GO.0004602	0.24685211
3	3	GO Function	oxoglutarate dehydrogenase (succinyl-transferring) activity	0.0034	OGDH|DHTKD1|OGDHL	0.00034	GO.0004591	0.24685211
3	3	GO Function	isocitrate dehydrogenase (NAD+) activity	0.0034	IDH3G|IDH3A|IDH3B	0.00034	GO.0004449	0.24685211
42	7	GO Process	neurotransmitter biosynthetic process	0.0034	AGXT2|SHMT1|SHMT2|ALDH9A1|ABAT|DDC|PAH	0.00027	GO.0042136	0.24685211
42	7	GO Process	antibiotic catabolic process	0.0034	ALDH2|PRDX1|PRDX5|ALDH1B1|AKR1C3|GPX3|GPX1	0.00027	GO.0017001	0.24685211
29	6	GO Process	prostaglandin metabolic process	0.0034	CBR1|CES2|PTGES2|AKR1C3|PTGR1|PTGR2	0.00027	GO.0006693	0.24685211
29	6	GO Process	leukotriene metabolic process	0.0034	CYP4A11|MGST3|DPEP1|GGT5|GGT1|PTGR1	0.00027	GO.0006691	0.24685211
18	5	GO Process	muscle cell cellular homeostasis	0.0035	CFL2|GAA|PFKM|ALDOA|LAMP2	0.00028	GO.0046716	0.2455932
18	5	GO Process	tetrahydrofolate metabolic process	0.0035	MTHFD1|FTCD|PIPOX|SHMT1|SHMT2	0.00028	GO.0046653	0.2455932

9	4	GO Process	tetrahydrofolate interconversion	0.0035	MTHFD1|FTCD|SHMT1|SHMT2	0.00028	GO.0035999	0.2455932
9	4	GO Process	nucleobase transport	0.0035	SLC25A4|SLC23A1|SLC25A5|SLC25A6	0.00028	GO.0015851	0.2455932
50	7	GO Component	stress fiber	0.0036	MYH9|ACTN4|MYH10|ACTN1|ILK|SEPT7|FLNB	0.0007	GO.0001725	0.24436975
399	24	GO Function	endopeptidase activity	0.0036	PSMA4|F9|CTSH|LTF|CTSD|CAPNS1|PMPCB|NAPSA|TINAG|AFG3L2|PSMA5|UCHL1|PSMB4|CAPN2|APEH|TPP1|CTSB|DPP4|LONP1|PSMB9|PSMB8|CFB|MME|CAPN1	0.00037	GO.0004175	0.24436975
186	15	GO Process	hormone metabolic process	0.0036	CRYM|APOA1|TTR|ENPEP|ALDH8A1|UGT2B7|HSD11B2|DHRS4|DHRS2|UGT1A9|ALDH9A1|RBP4|SCP2|AKR1C3|MME	0.00029	GO.0042445	0.24436975
1626	68	GO Process	tissue development	0.0036	RALA|DCN|VCL|MTHFD1|CTSH|DNPH1|CAPNS1|VIL1|LAMA5|LGALS3|GRSF1|GSTM3|SLC9A3R1|EPCAM|LRP2|VDAC1|CALB1|CPT1A|RHCG|IQGAP1|CA2|CBR1|CFL2|TPP1|GAA|DAB2|TOLLIP|PKM|KRT7|BSG|GSTA1|APOD|CTSB|ALDH3A2|COL18A1|MSN|MYH10|COL4A2|KRT19|COL6A1|TAGLN2|PHGDH|FLNA|RBP4|GSN|CLIC4|ASPN|COL4A1|AKR1C3|GPNMB|KRT18|PRKAR1A|PSAP|UMOD|MYH11|ILK|TST|RHOA|GPX1|TGFBI|GSTA2|FLNB|WDR1|CAPN1|TAGLN|PHB2|KRT8|PDLIM5	0.00029	GO.0009888	0.24436975
23	5	GO Component	integral component of mitochondrial outer membrane	0.0037	FIS1|SYNJ2BP|CPT1A|RHOT2|TOMM40	0.00073	GO.0031307	0.24317983
252	18	GO Process	carboxylic acid transport	0.0037	GOT2|SLC9A3R1|LRP2|CPT1A|SLC27A2|FABP1|SLC23A1|GLS|SLC7A8|BSG|ABCD3|CPT2|SLC3A2|PSAP|SLC25A12|ACSL1|SLC25A10|SLC43A2	0.0003	GO.0046942	0.24317983
228	16	GO Component	contractile fiber	0.0038	VCL|ENO1|HSPB1|ACTN4|SLC4A1|CFL2|PARVA|KRT19|FLNA|ACTN1|ALDOA|MYH11|ILK|FLNB|KRT8|TWF1	0.00077	GO.0043292	0.24202164
4963	170	GO Process	organic cyclic compound metabolic process	0.0038	HSD17B10|NDUFB4|NANS|GNPNAT1|MTHFD1|AHCY|CRYM|HNRNPL|OGDH|PDHX|ACOT13|DNPH1|LTF|ATP6V1B1|ENO1|AKR7A2|APOA1|GDA|GOT2|PMPCB|RPS16|NDUFA2|NDUFA10|LGALS3|GRSF1|HRSP12|GSTM3|NARS|GGH|DNAJA3|CHCHD3|DHTKD1|LRP2|ATP6V0A1|NNT|AMBP|PRDX5|REXO2|NDUFA9|SLC27A2|ATP6V1A|ATP6V1B2|ERLIN2|DPYS|NDUFB9|ENDOD1|HNMT|DLAT|QDPR|GPD1L|HGD|ACAA2|HPD|FTCD|HPRT1|AMN|NDUFB8|GPD1|PRODH2|SLC23A1|RPS9|DCXR|HINT1|UGT2B7|PDHB|CYCS|ATP6V0A4|GPD2|DPYSL2|UQCRH|AQP1|GBAS|HNRNPD|AHCYL2|HSD11B2|ACSS1|CYC1|PIPOX|SHMT1|UGDH|PKM|UGT2B17|TALDO1|NDUFV1|HNRNPM|DHRS4|MDH2|ACSM2B|NDUFA12|IDH2|UQCR10|ACAA1|SHMT2|DLST|PKLR|PPA2|DHRS2|PFKM|SND1|WARS|UBE2D3|VCP|COL4A2|LONP1|PNP|OPA1|IBA57|CYB5R1|ATP1B1|NDUFS2|LHPP|FLNA|RPL5|SCP2|CMPK1|PRPS1|OGDHL|HMGCL|HNRNPR|ALDOB|ALDH4A1|VARS|PSAT1|CUBN|MAOB|APRT|ACOT9|PDHA1|AKR1C3|FECH|BPGM|NME2|CNP|PC|ALDOA|AK4|QPRT|AMPD3|ATP5A1|ELAVL1|UBA52|IDH1|TKT|NDUFS1|RHOA|ACSF2|DDC|GPI|GPX1|LARS2|MME|PPIA|SSBP1|ABHD14B|ACSL1|EPHX2|PHB2|MMAB|RAN|RPL18|PAH|ALDH6A1|NPC2|ACSM2A|GALK1|DDX6|PHB|EPHX1|FMO1|GLYAT	0.00031	GO.1901360	0.24202164
122	11	GO Component	sarcolemma	0.0039	VCL|GOT2|COL6A2|AQP1|LAMP1|BSG|KRT19|COL6A1|ATP1B1|ATP1A1|KRT8	0.00078	GO.0042383	0.24089354
20	5	GO Function	C-acyltransferase activity	0.004	ACAA2|ACSM2B|ACAA1|SCP2|ACSM2A	0.00042	GO.0016408	0.239794
17	8	KEGG Pathways	Phenylalanine metabolism	1.2E-06	GOT2|HPD|AOC3|GOT1|MAOB|DDC|PAH|GLYAT	1.57E-07	hsa00360	0.5928118
300	19	GO Component	membrane raft	0.0041	VCL|CTSD|CDH1|SLC9A3R1|PACSIN2|VDAC1|IQGAP1|ERLIN2|LAMTOR1|CAPN2|BSG|LAMTOR4|DPP4|ATP1B1|SLC25A5|PRKAR1A|PDZK1|LAMP2|ATP1A1	0.00084	GO.0045121	0.23872161
52	7	GO Component	cleavage furrow	0.0041	RALA|MYH9|RAB21|RAB11A|MYH10|SEPT7|RHOA	0.00087	GO.0032154	0.23872161

52	7	GO Component	brush border membrane	0.0041	SLC9A3R1|LRP2|AMN|ATP6V0A4|AQP1|CUBN|PDZK1	0.00087	GO.0031526	0.23872161
1531	175	GO Component	mitochondrion	2.1E-57	HSD17B10|NDUFB4|NIPSNAP1|ACO2|MTHFD1|PCK2|IDH3G|CRYM|ECH1|OGDH|FIS1|SFXN3|PPIF|PDHX|SLC25A3|ACOT13|AGXT2|ACADL|ACADS|GOT2|PMPCB|NDUFA2|NDUFA10|LGALS3|GRSF1|HRSP12|DMGDH|SYNJ2BP|SQRDL|ALDH2|DNAJA3|CHCHD3|DHTKD1|MECR|NNT|ACAD11|VDAC1|PRDX5|CPT1A|REXO2|NDUFA9|SORD|AFG3L2|BCKDHA|ECSIT|ABHD10|NDUFB9|GLUD1|DLAT|SLC25A4|ACAA2|HSPA9|NDUFB8|IDH3A|GLYATL1|PRODH2|ECI1|YWHAG|TMEM126A|PDHB|CYCS|GPD2|ACO1|UQCRH|GBAS|TSFM|MSRA|SFXN1|ACSS1|CYC1|GLS|CHDH|RHOT2|NDUFV1|BCAT2|DHRS4|MDH2|ACSM2B|NDUFA12|IDH2|UQCR10|SHMT2|DLST|CYB5A|CS|PPA2|DHRS2|PTGES2|ANXA6|AGK|HIBCH|LONP1|MRPL37|MRPL24|MRPL21|OPA1|COA3|IBA57|MARC2|NDUFS2|ECHS1|ACADSB|SFXN2|ABCD3|HOGA1|ACADM|CPT2|SCP2|ECHDC2|SLC25A5|SARDH|ASS1|APOOL|OGDHL|CLIC4|HMGCL|ALDH4A1|AUH|CLIC1|AGMAT|ALDH1B1|MAOB|ACOT9|PDHA1|BPHL|IDH3B|GLDC|SLC25A6|FECH|BDH1|CNP|PC|AK4|MGST1|ABAT|GATM|MPST|HAGH|ATP5A1|TXNRD2|TST|ALDH7A1|IMMT|UBA52|SLC25A12|IDH1|NDUFS1|HIGD1A|GK|ACSF2|CKMT2|CKMT1A|GPX1|LARS2|TOMM40|MRPS22|GFM1|FAM162A|SSBP1|ACSL1|NDUFA13|ETFDH|CAPN1|ECHDC1|ME3|PHB2|MMAB|SLC25A10|ACSS3|ALDH6A1|ACSM2A|HADH|DDX6|PHB|GLYAT	6.33E-60	GO.0005739	5.66861328
164	13	GO Component	ruffle	0.0041	MYH9|VIL1|SLC9A3R1|PACSIN2|IQGAP1|ATP6V1B2|TLN1|MYO6|GSN|NME2|ACTN1|RHOA|TWF1	0.00084	GO.0001726	0.23872161
61	8	GO Function	oxidoreductase activity, acting on a sulfur group of donors	0.0041	TXNL1|SQRDL|MSRA|PTGES2|GSTO1|TXNRD2|IFI30|PDIA6	0.00044	GO.0016667	0.23872161
283	19	GO Function	GTPase activity	0.0041	RALA|GNA11|ARL8B|RAB21|RAB11A|RAB2A|RAB17|RAB7A|ARL8A|GNB2|RAB1B|RAB6A|RHOT2|RAB11B|OPA1|RAB14|RHOA|GFM1|RAN	0.00043	GO.0003924	0.23872161
3	3	GO Process	amino-acid betaine catabolic process	0.0041	ACADL|DMGDH|BHMT	0.00034	GO.0006579	0.23872161
3	3	GO Process	NADH oxidation	0.0041	GPD1|GPD2|ALDOB	0.00034	GO.0006116	0.23872161
46	7	GO Function	NADP binding	0.0042	CRYM|NNT|QDPR|GRHPR|IDH1|CRYZ|FMO1	0.00045	GO.0050661	0.23767507
19	5	GO Process	glyceraldehyde-3-phosphate metabolic process	0.0042	KHK|TALDO1|ALDOB|DAK|TKT	0.00035	GO.0019682	0.23767507
92	10	GO Process	cellular component assembly involved in morphogenesis	0.0043	CFL2|EPB41L3|MYH10|KRT19|CD9|PRKAR1A|MYH11|ILK|WDR1|KRT8	0.00035	GO.0010927	0.23665315
44	7	GO Process	purine nucleotide catabolic process	0.0043	DNPH1|GDA|HPRT1|HINT1|PNP|AMPD3|GPX1	0.00035	GO.0006195	0.23665315

3131	115	GO Process	organelle organization	0.0044	RALA|HSD17B10|NDUFB4|MYH9|ATP6V1D|ECH1|FIS1|PPIF|EHHADH|PFN2|TFG|VIL1|PMPCB|NDUFA2|ACTN4|NDUFA10|LAMA5|SYNJ2BP|HEXB|RAB11A|DNAJA3|CHCHD3|SLC9A3R1|RAB2A|PACSIN2|NAPA|RAB17|ADD1|RAB7A|NDUFA9|SLC27A2|AFG3L2|ECSIT|NDUFB9|LAMTOR1|SLC25A4|ACAA2|SCIN|CFL2|NDUFB8|TPP1|TMED10|GAA|YWHAG|CYCS|DPYSL2|RAB1B|AQP1|TLN1|PIPOX|RHOT2|NDUFV1|DHRS4|EHD3|ATP6V0C|NDUFA12|TMED9|UQCR10|ACAA1|PARVA|EPB41L3|VAPA|AGK|PLS3|UBE2D3|MSN|MYH10|LONP1|OPA1|COA3|KRT19|NDUFS2|FLNA|ABCD3|RPL5|SCP2|SLC25A5|APOOL|GSN|RAB14|HMGCL|HIST1H2AC|NAP1L4|SLC25A6|KRT18|PRKAR1A|CNP|ACTN1|PRKCD|ALDOA|CD59|MYH11|ATP5A1|SEPT7|IMMT|UBA52|IDH1|NDUFS1|RHOA|TOMM40|SERPINA1|PPIA|SSBP1|FLNB|TMEM33|NDUFA13|WDR1|EPHX2|PHB2|RAN|KRT8|CIRBP|DDX6|PHB|HAO2	0.00037	GO.0006996	0.23565473
62	8	GO Function	iron-sulfur cluster binding	0.0045	ACO2|ACO1|NDUFV1|NDUFS2|FECH|ABAT|NDUFS1|ETFDH	0.00049	GO.0051536	0.23467875
62	8	GO Function	myosin binding	0.0045	RALA|RAB11A|SLC9A3R1|RAB6A|RAB11B|GSN|RAB14|RHOA	0.00049	GO.0017022	0.23467875
670	33	GO Component	perinuclear region of cytoplasm	0.0046	EHD4|HNRNPL|ACTN4|CDH1|RAB11A|SLC9A3R1|PRDX5|CAPN2|GNB2|TOLLIP|EHD3|LAMP1|APOD|PTGES2|CTSB|ANXA6|VCP|MSN|FLNA|MYO6|GSN|RAB14|CLIC4|CLIC1|CKAP4|MT1M|KRT18|CNP|PRKCD|LAMP2|TWF1|NDRG2|DDX6	0.001	GO.0048471	0.23372422
234	16	GO Component	late endosome	0.0046	GNPNAT1|CTSH|NAPSA|ARL8B|RAB11A|SCARB2|RAB7A|ARL8A|LAMTOR1|LAMP1|LAMTOR4|ANXA6|RAB14|LAMP2|CD63|VPS29	0.001	GO.0005770	0.23372422
11	4	GO Function	AMP binding	0.0046	ACSS1|PRPS1|APRT|FBP1	0.00051	GO.0016208	0.23372422
21	5	GO Function	threonine-type endopeptidase activity	0.0046	PSMA4|PSMA5|PSMB4|PSMB9|PSMB8	0.00051	GO.0004298	0.23372422
10	4	GO Process	aldehyde catabolic process	0.0046	AGXT2|HOGA1|AKR1A1|HAGH	0.00038	GO.0046185	0.23372422
93	10	GO Process	diterpenoid metabolic process	0.0046	APOA1|TTR|LRP2|ALDH8A1|DHRS4|ALDH3A2|UGT1A9|RBP4|HSPG2|AKR1C3	0.00038	GO.0016101	0.23372422
10	4	GO Process	lysine catabolic process	0.0046	CRYM|PIPOX|DLST|ALDH7A1	0.00038	GO.0006554	0.23372422
330	20	GO Component	intrinsic component of organelle membrane	0.0047	SYPL1|FIS1|CANX|SYNJ2BP|CHCHD3|CPT1A|SLC27A2|AFG3L2|SLC25A4|RHOT2|RAB11B|AGK|COA3|APOOL|SACM1L|IMMT|LAMP2|TOMM40|TMEM33|ETFDH	0.001	GO.0031300	0.23279021
63	8	GO Function	mRNA 3'-UTR binding	0.0047	HNRNPL|HNRNPD|RPL5|HNRNPR|AUH|ELAVL1|CRYZ|CIRBP	0.00054	GO.0003730	0.23279021
5	3	KEGG Pathways	Phenylalanine, tyrosine and tryptophan biosynthesis	0.004	GOT2|GOT1|PAH	0.00092	hsa00400	0.239794
60	8	GO Process	cellular response to amino acid stimulus	0.0047	LAMTOR1|CAPN2|HNRNPD|LAMTOR4|OPA1|COL6A1|ASS1|COL4A1	0.0004	GO.0071230	0.23279021
71	8	GO Component	phagocytic vesicle membrane	0.005	ATP6V0A1|RAB7A|ATP6V0D2|ATP6V0A4|ATP6V0C|LAMP1|RAB11B|LAMP2	0.0011	GO.0030670	0.230103
70	7	KEGG Pathways	Platinum drug resistance	0.0153	GSTM3|CYCS|GSTA1|MGST3|GSTO1|MGST1|GSTA2	0.0041	hsa01524	0.18153086
4	3	GO Function	carbonyl reductase (NADPH) activity	0.0051	CBR1|DHRS4|DHRS2	0.00058	GO.0004090	0.22924298
4	3	GO Function	nucleobase binding	0.0051	DPYS|PNP|APRT	0.00058	GO.0002054	0.22924298

32	6	GO Process	actin filament capping	0.0051	VIL1|CAPG|ADD1|SCIN|GSN|TWF1	0.00043	GO.0051693	0.22924298
32	6	GO Process	GTP metabolic process	0.0051	OPA1|NME2|AK4|AMPD3|RHOA|RAN	0.00043	GO.0046039	0.22924298
4656	160	GO Process	organic substance biosynthetic process	0.0052	DCN|NANS|GNPNAT1|MTHFD1|PCK2|AHCY|OGDH|PDHX|DNPH1|AGXT2|LTF|ENO1|APOA1|GDA|GOT2|RPS16|BHMT2|GSTM3|NARS|HEXB|DNAJA3|CHCHD3|DHTKD1|BBOX1|MECR|ATP6V0A1|ALDH8A1|NDUFA9|SORD|SLC27A2|BHMT|GLUD1|DLAT|QDPR|GPD1L|ACAA2|CBR1|AGL|APEH|CRYL1|HPRT1|GPD1|RPS9|DCXR|HINT1|ASL|PDHB|ATP6V0A4|GPD2|AQP1|GBAS|HNRNPD|TSFM|HSD11B2|ACSS1|CYC1|GLS|SHMT1|UGDH|PCK1|CHDH|PKM|TALDO1|BCAT2|CNDP2|MDH2|ACSM2B|IDH2|SHMT2|GSTA1|PKLR|PPA2|PTGES2|VAPA|PFKM|ALDH3A2|SND1|ALDH9A1|WARS|AGK|VCP|COL4A2|MRPL37|MRPL24|PNP|MRPL21|IBA57|CYB5R1|MGST3|LHPP|PHGDH|GSTO1|FLNA|ABCD3|RPL5|GOT1|HOGA1|ACADM|RBP4|SCP2|CMPK1|AKR1A1|ASS1|PRPS1|RAB14|OGDHL|HMGCL|HSPG2|ALDOB|VARS|AGMAT|PSAT1|APRT|PDHA1|AKR1C3|FECH|SACM1L|BDH1|BPGM|NME2|CNP|PC|ALDOA|AK4|QPRT|MGST1|AMPD3|ABAT|GATM|HAGH|GGT5|ATP5A1|GGT1|ALDH7A1|UBA52|SLC25A12|TKT|GK|DDC|GPI|GPX1|FBP1|LARS2|MRPS22|GFM1|PPIA|SSBP1|GSTA2|ACSL1|EPHX2|PHB2|MMAB|SLC25A10|RPL18|PAH|ACSS3|ACSM2A|GALK1|PHB|PLCG2	0.00044	GO.1901576	0.22839967
310	19	GO Component	integral component of organelle membrane	0.0054	SYPL1|FIS1|CANX|SYNJ2BP|CHCHD3|CPT1A|SLC27A2|AFG3L2|SLC25A4|RHOT2|AGK|COA3|APOOL|SACM1L|IMMT|LAMP2|TOMM40|TMEM33|ETFDH	0.0012	GO.0031301	0.22676062
216	15	GO Component	myofibril	0.0055	VCL|ENO1|HSPB1|ACTN4|SLC4A1|CFL2|PARVA|KRT19|FLNA|ACTN1|ALDOA|ILK|FLNB|KRT8|TWF1	0.0012	GO.0030016	0.22596373
91	9	GO Component	platelet alpha granule	0.0056	ACTN4|F13A1|SERPINA5|CYB5R1|APOOL|CD9|ACTN1|ALDOA|SERPINA1	0.0013	GO.0031091	0.2251812
270	18	GO Function	carbohydrate binding	0.0056	GNPNAT1|CANX|LGALS3|APCS|TINAG|SORD|TINAGL1|AGL|GAA|TALDO1|BSG|PFKM|PRPS1|ALDOB|ALDOA|FBP1|MGAM|GALK1	0.00065	GO.0030246	0.2251812
1223	53	GO Function	transporter activity	0.0056	ATP6V1D|SFXN3|SLC25A3|ATP6V1B1|APOA1|ATP6V1E1|SLC4A1|PACSIN2|LRP2|ATP6V0A1|NNT|VDAC1|SLC27A2|RHCG|ATP6V1A|ATP6V1B2|SLC25A4|ATP6V0D2|FABP1|SLC23A1|ATP6V0A4|AQP1|SFXN1|SLC7A8|SLC5A2|ATP6V0C|BSG|CYB5A|APOD|ANXA6|GM2A|ATP6V1H|ATP1B1|SFXN2|ABCD3|RBP4|SCP2|SLC25A5|ATP6V1G1|CLIC4|CLIC1|CUBN|SLC3A2|SLC25A6|ATP5A1|SLC25A12|SLC4A4|PDZK1|TOMM40|ATP1A1|SLC25A10|NPC2|SLC43A2	0.00065	GO.0005215	0.2251812
392	23	GO Function	ATPase activity	0.0057	MYH9|ATP6V1D|PFN2|ATP6V1E1|ATP6V0A1|ATP6V1A|ATP6V1B2|ATP6V0D2|ATP6V0A4|ATP6V0C|VCP|ATP6V1H|MYH10|LONP1|KIF21A|ATP1B1|ABCD3|ATP6V1G1|ATP5A1|KIF13B|ATP1A1|DDX6|PSMC3	0.00068	GO.0016887	0.22441251
62	8	GO Process	organic hydroxy compound catabolic process	0.0057	ALDH2|SORD|GPD2|CYP4A11|ALDH1B1|MAOB|AKR1C3|GK	0.00049	GO.1901616	0.22441251
196	15	GO Process	cellular response to acid chemical	0.0057	CPT1A|LAMTOR1|CAPN2|AQP1|HNRNPD|SHMT1|PCK1|LAMTOR4|OPA1|COL6A1|ASS1|COL4A1|AKR1C3|NDUFA13|PHB2	0.00048	GO.0071229	0.22441251
175	14	GO Process	carbohydrate derivative catabolic process	0.0058	DCN|AHCY|HEXB|ABHD10|DPYS|GPD1L|HPRT1|GPD1|GUSB|HINT1|GM2A|PNP|HSPG2|AMPD3	0.00049	GO.1901136	0.2236572
6	3	GO Component	vacuolar proton-transporting V-type ATPase, V0 domain	0.0059	ATP6V0A1|ATP6V0A4|ATP6V0C	0.0013	GO.0000220	0.2229148

11	4	GO Process	erythrose 4-phosphate/phosphoenolpyruvate family amino acid metabolic process	0.0059	QDPR|HGD|HPD|PAH	0.00051	GO.1902221	0.2229148
47	7	GO Process	pigment biosynthetic process	0.0059	MTHFD1|HPRT1|SHMT1|IBA57|PRPS1|APRT|FECH	0.00051	GO.0046148	0.2229148
11	4	GO Process	xenobiotic catabolic process	0.0059	GSTM3|GSTO1|CRYZ|ACSL1	0.00051	GO.0042178	0.2229148
47	7	GO Process	interleukin-12-mediated signaling pathway	0.0059	RALA|HSPA9|TALDO1|MSN|GSTO1|PPIA|GSTA2	0.00051	GO.0035722	0.2229148
97	10	GO Process	actomyosin structure organization	0.0059	MYH9|CFL2|EPB41L3|MYH10|KRT19|PRKAR1A|MYH11|RHOA|WDR1|KRT8	0.00052	GO.0031032	0.2229148
11	4	GO Process	L-ascorbic acid metabolic process	0.0059	SLC23A1|CYB5A|GSTO1|AKR1A1	0.00051	GO.0019852	0.2229148
21	5	GO Process	mitochondrial calcium ion transmembrane transport	0.0059	PMPCB|AFG3L2|OPA1|PHB2|PHB	0.00051	GO.0006851	0.2229148
11	4	GO Process	creatine metabolic process	0.0059	CKB|GATM|CKMT2|CKMT1A	0.00051	GO.0006600	0.2229148
11	4	GO Process	L-serine metabolic process	0.0059	SHMT1|SHMT2|PHGDH|PSAT1	0.00051	GO.0006563	0.2229148
11	4	GO Process	L-phenylalanine catabolic process	0.0059	QDPR|HGD|HPD|PAH	0.00051	GO.0006559	0.2229148
11	4	GO Process	L-phenylalanine metabolic process	0.0059	QDPR|HGD|HPD|PAH	0.00051	GO.0006558	0.2229148
66	8	GO Function	monosaccharide binding	0.006	GNPNAT1|TALDO1|BSG|PFKM|ALDOB|ALDOA|FBP1|GALK1	0.00071	GO.0048029	0.22218487
866	41	GO Process	cation transport	0.0061	ATP6V1D|SLC25A3|LTF|ATP6V1B1|PMPCB|ATP6V1E1|SLC4A1|SLC9A3R1|LRP2|ATP6V0A1|NNT|NDUFA9|RHCG|AFG3L2|ATP6V1A|FTH1|ATP6V1B2|ATP6V0D2|SLC23A1|ATP6V0A4|AQP1|SFXN1|CYC1|SLC7A8|SLC5A2|ATP6V0C|RAB11B|CYB5A|ANXA6|ATP6V1H|OPA1|ATP1B1|ATP6V1G1|SLC3A2|ATP5A1|SLC4A4|PDZK1|PHB2|ATP1A1|PHB|PLCG2	0.00054	GO.0006812	0.22146702
243	17	GO Process	regulation of cysteine-type endopeptidase activity	0.0062	CTSH|FIS1|LTF|VIL1|DNAJA3|PRDX5|FABP1|CYCS|AQP1|VCP|GSN|PSMB9|DPEP1|RHOA|GPX1|FAM162A|NDUFA13	0.00055	GO.2000116	0.22076083
42	6	KEGG Pathways	Porphyrin and chlorophyll metabolism	0.0064	GUSB|UGT2B7|UGT2B17|UGT1A9|FECH|MMAB	0.0015	hsa00860	0.219382
72	15	KEGG Pathways	PPAR signaling pathway	8.9E-08	PCK2|EHHADH|ACADL|APOA1|CPT1A|SLC27A2|FABP1|PCK1|ACAA1|ACADM|CPT2|SCP2|ILK|GK|ACSL1	7.45E-09	hsa03320	0.70520764
48	7	GO Process	guanosine-containing compound metabolic process	0.0064	HPRT1|OPA1|NME2|AK4|AMPD3|RHOA|RAN	0.00057	GO.1901068	0.219382

437	25	GO Process	monovalent inorganic cation transport	0.0064	SLC25A3|ATP6V1B1|ATP6V1E1|SLC4A1|SLC9A3R1|ATP6V0A1|NNT|NDUFA9|ATP6V1A|ATP6V1B2|ATP6V0D2|SLC23A1|ATP6V0A4|AQP1|CYC1|SLC5A2|ATP6V0C|CYB5A|ATP6V1H|ATP1B1|ATP6V1G1|SLC3A2|ATP5A1|SLC4A4|ATP1A1	0.00057	GO.0015672	0.219382
4	3	GO Process	adenine transport	0.0066	SLC25A4|SLC25A5|SLC25A6	0.00058	GO.0015853	0.21804561
4	3	GO Process	glycine biosynthetic process	0.0066	AGXT2|SHMT1|SHMT2	0.00058	GO.0006545	0.21804561
4	3	GO Process	xylulose metabolic process	0.0066	DCXR|TALDO1|TKT	0.00058	GO.0005997	0.21804561
5305	177	GO Function	heterocyclic compound binding	0.0067	RALA|HEBP1|GNA11|HSD17B10|MYH9|MTHFD1|PCK2|PYGB|AHCY|IDH3G|CRYM|EHD4|HNRNPL|OGDH|AGXT2|LTF|ACADL|ATP6V1B1|ENO1|ACADS|GOT2|RPS16|ACTN4|GRSF1|ARL8B|NARS|TINAG|KHK|RAB21|ALDH2|RAB11A|DNAJA3|RAB2A|DHTKD1|RAB17|NNT|ACAD11|RAB7A|AMBP|PRDX5|REXO2|SORD|SLC27A2|AFG3L2|ARL8A|ATP6V1A|ATP6V1B2|DPYS|GLUD1|ENDOD1|QDPR|GPD1L|FTCD|HSPA9|CRYL1|HPRT1|CKB|IDH3A|GPD1|PRODH2|RPS9|HINT1|CYCS|ACO1|RAB1B|CYP4A11|RAB6A|HNRNPD|GRHPR|TSFM|HSD11B2|ACSS1|CYC1|SHMT1|UGDH|PCK1|CHDH|PKM|RHOT2|NDUFV1|EHD3|ACSM2B|IDH2|RAB11B|SHMT2|PKLR|CYB5A|PTGES2|PFKM|ANXA6|WARS|AGK|UBE2D3|VCP|MSN|MYH10|LONP1|PNP|OPA1|KIF21A|MARC2|NDUFS2|ACADSB|PHGDH|MYO6|ABCD3|RPL5|GOT1|ACADM|SCP2|CMPK1|ASS1|PRPS1|RAB14|OGDHL|HMGCL|HNRNPR|VARS|AUH|ALDH1B1|HIST1H2AC|CUBN|SLC3A2|MAOB|APRT|NQO2|IDH3B|GLDC|PRKAR1A|NME2|CNP|PC|ACTN1|PRKCD|DAK|AK4|MYH11|ABAT|ILK|ATP5A1|SEPT7|TXNRD2|ELAVL1|TST|IDH1|CRYZ|RHOA|GK|ACSF2|DDC|CKMT2|CKMT1A|FBP1|LARS2|GFM1|SSBP1|ACSL1|NDUFA13|ETFDH|KIF13B|ME3|ATP1A1|MMAB|RAN|RPL18|TWF1|ACSS3|ALDH6A1|ACSM2A|GALK1|CIRBP|HADH|DDX6|PHB|PSMC3|FMO1|HAO2	0.0008	GO.1901363	0.21739252
22	5	GO Process	toxin metabolic process	0.0069	SQRDL|GSTO1|MPST|DDC|FMO1	0.00061	GO.0009404	0.21611509
22	5	GO Process	aspartate family amino acid biosynthetic process	0.0069	MTHFD1|GOT2|BHMT2|BHMT|GOT1	0.00061	GO.0009067	0.21611509
12	4	GO Process	AMP metabolic process	0.0074	PRPS1|APRT|AK4|AMPD3	0.00067	GO.0046033	0.21307683
12	4	GO Process	ethanol oxidation	0.0074	ALDH2|ADH1B|ACSS1|ALDH1B1	0.00067	GO.0006069	0.21307683
12	4	GO Process	glycogen catabolic process	0.0074	PYGB|AGL|GAA|PFKM	0.00067	GO.0005980	0.21307683
5	3	GO Function	fructose binding	0.0076	PFKM|ALDOB|ALDOA	0.00092	GO.0070061	0.21191864
5	3	GO Function	thiamine pyrophosphate binding	0.0076	OGDH|DHTKD1|OGDHL	0.00092	GO.0030976	0.21191864
136	11	GO Component	I band	0.0077	HSPB1|ACTN4|SLC4A1|CFL2|PARVA|KRT19|FLNA|ACTN1|ALDOA|FLNB|KRT8	0.0018	GO.0031674	0.21135093
161	13	GO Process	organic hydroxy compound biosynthetic process	0.0078	APOA1|SLC27A2|QDPR|ACAA2|PCK1|CYB5R1|GOT1|SCP2|PSAT1|AKR1C3|DDC|PAH|PLCG2	0.00071	GO.1901617	0.21079054
23	5	GO Process	glutamine metabolic process	0.008	GLUD1|GLYATL1|GLS|PHGDH|NIT2	0.00073	GO.0006541	0.209691
121	11	GO Process	striated muscle cell development	0.0081	AFG3L2|UCHL1|CFL2|MYH10|KRT19|PRKAR1A|MYH11|GPX1|WDR1|KRT8|PDLIM5	0.00074	GO.0055002	0.2091515

<table>
<tr><td>102</td><td>10</td><td>GO Process</td><td>polyol metabolic process</td><td>0.0081</td><td>SORD|QDPR|GPD2|PCK1|GOT1|SCP2|DAK|GK|GALK1|PLCG2</td><td>0.00074</td><td>GO.0019751</td><td>0.2091515</td></tr>
<tr><td>7</td><td>3</td><td>GO Component</td><td>MICOS complex</td><td>0.0082</td><td>CHCHD3|APOOL|IMMT</td><td>0.0019</td><td>GO.0061617</td><td>0.20861861</td></tr>
<tr><td>36</td><td>6</td><td>GO Process</td><td>cellular metabolic compound salvage</td><td>0.0082</td><td>BHMT2|BHMT|HPRT1|PNP|APRT|AMPD3</td><td>0.00075</td><td>GO.0043094</td><td>0.20861861</td></tr>
<tr><td>299</td><td>18</td><td>GO Component</td><td>endoplasmic reticulum lumen</td><td>0.0083</td><td>F9|APOA1|CANX|SLC27A2|COL14A1|COL6A2|CES2|COL18A1|COL4A2|COL6A1|COL4A1|CKAP4|TXNDC5|KTN1|PDIA6|SERPINA1|VWA1|FMO1</td><td>0.0019</td><td>GO.0005788</td><td>0.20809219</td></tr>
<tr><td>147</td><td>12</td><td>GO Function</td><td>isomerase activity</td><td>0.0084</td><td>ECH1|PPIF|EHHADH|ECI1|PTGES2|PBLD|TXNDC5|BPGM|PDIA6|GPI|PPIA|GALE</td><td>0.001</td><td>GO.0016853</td><td>0.20757207</td></tr>
<tr><td>88</td><td>9</td><td>GO Function</td><td>steroid binding</td><td>0.0084</td><td>APOA1|CALB1|ERLIN2|HSD11B2|APOD|ANXA6|SCP2|ATP1A1|NPC2</td><td>0.001</td><td>GO.0005496</td><td>0.20757207</td></tr>
<tr><td>402</td><td>22</td><td>GO Component</td><td>cytoplasmic region</td><td>0.0086</td><td>VCL|MYH9|ENO1|CANX|HSPB1|ACTN4|RAB21|SLC4A1|UCHL1|CAPN2|FABP1|SCIN|MYH10|OPA1|KRT19|FLNA|MYO6|GSN|SEPT7|RHOA|FLNB|WDR1</td><td>0.002</td><td>GO.0099568</td><td>0.20655015</td></tr>
<tr><td>103</td><td>10</td><td>GO Process</td><td>response to amino acid</td><td>0.0086</td><td>LAMTOR1|CAPN2|HNRNPD|PCK1|LAMTOR4|OPA1|COL6A1|ASS1|COL4A1|RHOA</td><td>0.0008</td><td>GO.0043200</td><td>0.20655015</td></tr>
<tr><td>970</td><td>44</td><td>GO Process</td><td>macromolecule catabolic process</td><td>0.0086</td><td>PSMA4|DCN|PYGB|PSMD7|CTSH|CTSD|RPS16|NAPSA|LYZ|HEXB|PSMD11|PSMD3|RAB7A|AMBP|PSMA5|ERLIN2|UCHL1|PSMB4|AGL|CAPN2|TPP1|GUSB|RPS9|GAA|HNRNPD|TOLLIP|AP2A2|CTSB|PFKM|SND1|UBE2D3|VCP|LONP1|RPL5|HSPG2|PSMB9|PSMB8|UBA52|LAMP2|CAPN1|RPL18|MGAM|DDX6|PSMC3</td><td>0.00079</td><td>GO.0009057</td><td>0.20655015</td></tr>
<tr><td>14</td><td>4</td><td>GO Function</td><td>phosphotransferase activity, nitrogenous group as acceptor</td><td>0.0087</td><td>CKB|NME2|CKMT2|CKMT1A</td><td>0.0011</td><td>GO.0016775</td><td>0.20604807</td></tr>
<tr><td>185</td><td>14</td><td>GO Process</td><td>endosomal transport</td><td>0.0089</td><td>EHD4|RAB11A|RAB17|RAB7A|TINAGL1|LAMTOR1|RAB6A|EHD3|RAB11B|VCP|RAB14|UBA52|TOM1|VPS29</td><td>0.00082</td><td>GO.0016197</td><td>0.205061</td></tr>
<tr><td>13</td><td>4</td><td>GO Process</td><td>homocysteine metabolic process</td><td>0.0092</td><td>MTHFD1|AHCY|DPEP1|MPST</td><td>0.00085</td><td>GO.0050667</td><td>0.20362122</td></tr>
<tr><td>664</td><td>33</td><td>GO Process</td><td>metal ion transport</td><td>0.0093</td><td>ATP6V1D|LTF|ATP6V1B1|PMPCB|ATP6V1E1|SLC4A1|SLC9A3R1|LRP2|ATP6V0A1|NDUFA9|AFG3L2|ATP6V1A|FTH1|ATP6V1B2|ATP6V0D2|SLC23A1|ATP6V0A4|AQP1|SFXN1|SLC5A2|ATP6V0C|RAB11B|ANXA6|ATP6V1H|OPA1|ATP1B1|ATP6V1G1|SLC3A2|SLC4A4|PHB2|ATP1A1|PHB|PLCG2</td><td>0.00087</td><td>GO.0030001</td><td>0.20315171</td></tr>
<tr><td>52</td><td>7</td><td>GO Process</td><td>secondary metabolic process</td><td>0.0093</td><td>AKR7A2|SQRDL|GSTO1|AKR1C3|MPST|DDC|FMO1</td><td>0.00087</td><td>GO.0019748</td><td>0.20315171</td></tr>
<tr><td>144</td><td>12</td><td>GO Process</td><td>sensory perception of sound</td><td>0.0094</td><td>CRYM|ATP6V1B1|HEXB|SLC9A3R1|LRP2|NDUFB9|ATP6V0A4|MYO6|ALDH7A1|GPX1|WDR1|EML2</td><td>0.00088</td><td>GO.0007605</td><td>0.20268721</td></tr>
<tr><td>32</td><td>10</td><td>KEGG Pathways</td><td>Propanoate metabolism</td><td>8E-07</td><td>EHHADH|BCKDHA|ACSS1|HIBCH|ECHS1|ACADM|ABAT|ECHDC1|ACSS3|ALDH6A1</td><td>9.96E-08</td><td>hsa00640</td><td>0.60952845</td></tr>
<tr><td>209</td><td>15</td><td>GO Process</td><td>regulation of cysteine-type endopeptidase activity involved in apoptotic process</td><td>0.0096</td><td>CTSH|FIS1|VIL1|DNAJA3|PRDX5|FABP1|CYCS|AQP1|VCP|GSN|DPEP1|RHOA|GPX1|FAM162A|NDUFA13</td><td>0.0009</td><td>GO.0043281</td><td>0.20177288</td></tr>
<tr><td>584</td><td>30</td><td>GO Process</td><td>cellular ion homeostasis</td><td>0.0096</td><td>FIS1|LTF|HEXB|SLC4A1|SLC9A3R1|ATP6V0A1|RAB7A|CALB1|RHCG|AFG3L2|ATP6V1A|FTH1|CA2|ATP6V0D2|CKB|ATP6V0A4|ACO1|ATP6V0C|ANXA6|ATP6V1H|ATP1B1|GSTO1|GOT1|ATP6V1G1|CLIC4|IMMT|SLC4A4|EPHX2|ATP1A1|PLCG2</td><td>0.0009</td><td>GO.0006873</td><td>0.20177288</td></tr>
</table>

5	3	GO Process	fructose catabolic process to hydroxyacetone phosphate and glyceraldehyde-3-phosphate	0.0097	KHK|ALDOB|DAK	0.00092	GO.0061624	0.20132283
5	3	GO Process	4-hydroxyproline catabolic process	0.0097	GOT2|HOGA1|ALDH4A1	0.00092	GO.0019470	0.20132283
5	3	GO Process	response to aluminum ion	0.0097	QDPR|LONP1|MAOB	0.00092	GO.0010044	0.20132283
166	13	GO Process	viral life cycle	0.0098	SCARB2|RAB7A|ANPEP|RAB1B|LAMP1|CTSB|VCP|DPP4|PC|UBA52|PPIA|RAN|DDX6	0.00093	GO.0019058	0.20087739
53	7	GO Process	sulfur compound catabolic process	0.0101	DCN|AHCY|HEXB|MPST|GGT5|GGT1|TST	0.00096	GO.0044273	0.19956786
87	9	GO Process	retinoid metabolic process	0.0101	APOA1|TTR|LRP2|ALDH8A1|DHRS4|UGT1A9|RBP4|HSPG2|AKR1C3	0.00095	GO.0001523	0.19956786
38	6	GO Process	vitamin transport	0.0102	APOA1|LRP2|AMN|SLC23A1|RBP4|CUBN	0.00097	GO.0051180	0.19913998
6	3	GO Function	alpha-glucosidase activity	0.0107	AGL|GAA|MGAM	0.0013	GO.0090599	0.19706162
41	6	GO Function	4 iron, 4 sulfur cluster binding	0.0107	ACO2|ACO1|NDUFV1|NDUFS2|NDUFS1|ETFDH	0.0014	GO.0051539	0.19706162
15	4	GO Function	dipeptidase activity	0.0107	PEPD|CNDP2|DPEP1|SCRN1	0.0013	GO.0016805	0.19706162
6	3	GO Function	primary amine oxidase activity	0.0107	AOC3|MAOB|AOC1	0.0013	GO.0008131	0.19706162
6	3	GO Function	creatine kinase activity	0.0107	CKB|CKMT2|CKMT1A	0.0013	GO.0004111	0.19706162
165	12	GO Component	midbody	0.0108	RALA|ARL8B|CAPG|IQGAP1|ARL8A|MYH10|CLIC4|SEPT7|GDI1|ANXA11|RHOA|RAN	0.0026	GO.0030496	0.19665762
122	10	GO Component	Z disc	0.0108	HSPB1|ACTN4|SLC4A1|CFL2|PARVA|KRT19|FLNA|ACTN1|FLNB|KRT8	0.0026	GO.0030018	0.19665762
57	7	GO Function	monocarboxylic acid binding	0.0108	HRSP12|FABP1|SERPINA5|UGT1A9|SCP2|PC|PSAP	0.0014	GO.0033293	0.19665762
390	22	GO Function	monovalent inorganic cation transmembrane transporter activity	0.0108	SLC25A3|ATP6V1B1|ATP6V1E1|SLC4A1|ATP6V0A1|NNT|ATP6V1A|ATP6V1B2|ATP6V0D2|SLC23A1|ATP6V0A4|AQP1|SLC5A2|ATP6V0C|CYB5A|ATP6V1H|ATP1B1|ATP6V1G1|SLC3A2|ATP5A1|SLC4A4|ATP1A1	0.0014	GO.0015077	0.19665762
510	27	GO Process	endocytosis	0.0111	MYH9|EHD4|APOA1|CANX|TINAG|PACSIN2|LRP2|SCARB2|RAB7A|AMBP|TINAGL1|AMN|DPYSL2|DAB2|AP2A2|ATP6V1H|MYO6|GSN|HSPG2|CUBN|TXNDC5|CD9|PRKCD|PDIA6|ANXA11|TOM1|PLCG2	0.0011	GO.0006897	0.1954677
14	4	GO Process	glutathione biosynthetic process	0.0112	CNDP2|HAGH|GGT5|GGT1	0.0011	GO.0006750	0.1950782
511	27	GO Process	regulation of hormone levels	0.0113	CRYM|APOA1|TTR|ENPEP|ALDH8A1|CPT1A|GLUD1|SLC25A4|UGT2B7|HSD11B2|BCAT2|DHRS4|RAB11B|DHRS2|PFKM|UGT1A9|ALDH9A1|DPP4|RBP4|SCP2|SLC25A5|AKR1C3|SLC25A6|ABAT|MME|ATP1A1|HADH	0.0011	GO.0010817	0.19469216
71	8	GO Process	regulation of mitochondrial membrane permeability	0.0114	PPIF|SLC25A4|ACAA2|YWHAG|RHOT2|SLC25A5|SLC25A6|CNP	0.0011	GO.0046902	0.19430951

19	4	GO Component	costamere	0.0117	VCL|KRT19|ILK|KRT8	0.0028	GO.0043034	0.19318141
333	20	GO Process	regulation of cellular component size	0.0118	PFN2|VIL1|RAB21|RAB11A|SLC9A3R1|CAPG|ADD1|SCIN|CFL2|DPYSL2|AQP1|LAMTOR4|MSN|GSN|PRKCD|ILK|GDI1|RHOA|WDR1|TWF1	0.0011	GO.0032535	0.1928118
43	9	KEGG Pathways	Proteasome	4.2E-05	PSMA4|PSMD7|PSMD11|PSMD3|PSMA5|PSMB4|PSMB9|PSMB8|PSMC3	7.51E-06	hsa03050	0.43798639
16	4	GO Function	alcohol dehydrogenase (NADP+) activity	0.0124	AKR7A2|DHRS4|AKR1A1|AKR1C3	0.0016	GO.0008106	0.19065783
66	7	GO Component	endoplasmic reticulum-Golgi intermediate compartment membrane	0.0126	RAB2A|NAT8|TMED10|RAB1B|TMED9|CD59|SERPINA1	0.003	GO.0033116	0.18996295
150	12	GO Process	maintenance of location	0.0126	APOA1|HEXB|FTH1|SCIN|GAA|TLN1|EPB41L3|GM2A|FLNA|GSN|FLNB|TWF1	0.0012	GO.0051235	0.18996295
90	13	KEGG Pathways	Protein digestion and absorption	1.9E-05	COL14A1|COL6A2|SLC7A8|COL18A1|COL4A2|DPP4|COL6A1|ATP1B1|XPNPEP2|COL4A1|SLC3A2|MME|ATP1A1	3.04E-06	hsa04974	0.47328283
23	9	KEGG Pathways	Proximal tubule bicarbonate reclamation	7.9E-07	PCK2|GLUD1|CA2|AQP1|GLS|PCK1|ATP1B1|SLC4A4|ATP1A1	9.13E-08	hsa04964	0.61040253
179	13	GO Function	transferase activity, transferring acyl groups other than amino-acyl groups	0.0132	GNPNAT1|CPT1A|NAT8|DLAT|ACAA2|GLYATL1|ACSM2B|ACAA1|DLST|CPT2|SCP2|ACSM2A|GLYAT	0.0018	GO.0016747	0.18794261
39	18	KEGG Pathways	Pyruvate metabolism	8.9E-14	PCK2|ALDH2|DLAT|PDHB|GRHPR|ACSS1|PCK1|PKM|MDH2|PKLR|ALDH3A2|ALDH9A1|ALDH1B1|PDHA1|PC|HAGH|ALDH7A1|ME3	3.22E-15	hsa00620	1.30486625
9	3	GO Component	autolysosome	0.0137	FTH1|LAMP1|LAMP2	0.0033	GO.0044754	0.18632794
34	5	GO Component	endosome lumen	0.0138	CTSH|NAPSA|LRP2|CTSB|CD63	0.0034	GO.0031904	0.18601209
15	4	GO Process	NADPH regeneration	0.0138	NNT|TALDO1|IDH1|TKT	0.0013	GO.0006740	0.18601209
6	3	GO Process	purine nucleobase transmembrane transport	0.0139	SLC25A4|SLC25A5|SLC25A6	0.0013	GO.1904823	0.18569852
6	3	GO Process	mitochondrial acetyl-CoA biosynthetic process from pyruvate	0.0139	PDHX|PDHB|PDHA1	0.0013	GO.0061732	0.18569852
6	3	GO Process	nucleobase catabolic process	0.0139	GDA|DPYS|ALDH6A1	0.0013	GO.0046113	0.18569852
6	3	GO Process	positive regulation of dopamine metabolic process	0.0139	HPRT1|MAOB|ABAT	0.0013	GO.0045964	0.18569852
6	3	GO Process	sequestering of actin monomers	0.0139	SCIN|GSN|TWF1	0.0013	GO.0042989	0.18569852

6	3	GO Process	alditol catabolic process	0.0139	SORD|GPD2|GK	0.0013	GO.0019405	0.18569852
41	6	GO Process	acyl-CoA biosynthetic process	0.014	PDHX|DLAT|PDHB|ACSS1|PDHA1|ACSL1	0.0014	GO.0071616	0.1853872
41	6	GO Process	icosanoid biosynthetic process	0.014	CBR1|PTGES2|MGST3|AKR1C3|GGT5|GGT1	0.0014	GO.0046456	0.1853872
317	18	GO Component	cell projection membrane	0.0141	SLC9A3R1|PACSIN2|LRP2|AMN|ATP6V0A4|AQP1|TLN1|EHD3|EPB41L3|MSN|DPP4|MYO6|CUBN|DPEP1|UMOD|PDZK1|RHOA|TWF1	0.0035	GO.0031253	0.18507809
7	3	GO Function	alditol:NADP+ 1-oxidoreductase activity	0.0141	AKR7A2|AKR1A1|AKR1C3	0.0019	GO.0004032	0.18507809
195	13	GO Component	sarcomere	0.0142	ENO1|HSPB1|ACTN4|SLC4A1|CFL2|PARVA|KRT19|FLNA|ACTN1|ALDOA|ILK|FLNB|KRT8	0.0035	GO.0030017	0.18477117
442	24	GO Process	regulation of cell morphogenesis	0.0145	MYH9|APOA1|VIL1|ACTN4|RAB21|HEXB|RAB11A|SLC9A3R1|ARHGDIA|DPYSL2|PARVA|EPB41L3|MSN|MYH10|OPA1|FLNA|ALDOA|ILK|SEPT7|GDI1|RHOA|WDR1|KIF13B|PDLIM5	0.0014	GO.0022604	0.1838632
17	4	GO Function	myosin V binding	0.0147	RAB11A|RAB6A|RAB11B|RAB14	0.002	GO.0031489	0.18326827
17	4	GO Function	oxidoreductase activity, acting on the CH-NH group of donors, NAD or NADP as acceptor	0.0147	MTHFD1|CRYM|QDPR|ALDH4A1	0.002	GO.0016646	0.18326827
17	4	GO Function	fatty-acyl-CoA binding	0.0147	ACADL|SCP2|HMGCL|ALDH6A1	0.002	GO.0000062	0.18326827
267	17	GO Process	renal system development	0.0147	DCN|CTSH|LAMA5|HRSP12|EPCAM|ENPEP|CALB1|IQGAP1|CA2|TMED10|RBP4|ASS1|COL4A1|UMOD|ILK|MPST|MME	0.0015	GO.0072001	0.18326827
272	17	Reactome Pathways	Innate Immune System	0.0148	LGALS3|GGH|LYZ|COTL1|ARL8A|FTH1|APEH|ANPEP|PKM|LAMP1|CKAP4|TXNDC5|NIT2|TOM1|MME|MGAM|NPC2	0.0018	HSA-168249	0.18297383
1732	68	GO Process	regulation of transport	0.0149	RALA|EHD4|FIS1|PPIF|REEP6|APOA1|PFN2|ACTN4|LGALS3|SYNJ2BP|RAB21|CDH1|RAB11A|SLC9A3R1|PACSIN2|NAPA|RAB17|RAB7A|CPT1A|GLUD1|LAMTOR1|SLC25A4|GPD1L|ACAA2|CA2|SCIN|YWHAG|CYP4A11|AQP1|GBAS|DAB2|EHD3|IDH2|LAMP1|RAB11B|APOD|PFKM|MSN|MYH10|DPP4|OPA1|ATP1B1|GSTO1|FLNA|MYO6|RBP4|SCP2|SLC25A5|RAB14|CLIC4|CLIC1|TBC1D4|MAOB|SLC25A6|FHL1|PRKCD|ABAT|GDI1|PDZK1|RHOA|GPI|PPIA|WDR1|CAPN1|ATP1A1|CD63|HADH|PLCG2	0.0015	GO.0051049	0.18268137
75	8	GO Process	Golgi vesicle budding	0.0152	TFG|NAPA|TMED10|RAB1B|TMED9|VAPA|CD59|SERPINA1	0.0015	GO.0048194	0.18181564
205	13	KEGG Pathways	Regulation of actin cytoskeleton	0.0186	VCL|MYH9|MYL12A|PFN2|ACTN4|IQGAP1|SCIN|CFL2|MSN|MYH10|GSN|ACTN1|RHOA	0.0052	hsa04810	0.17304871
21	4	GO Component	proteasome regulatory particle	0.0154	PSMD7|PSMD11|PSMD3|PSMC3	0.0038	GO.0005838	0.18124793
45	6	GO Function	cholesterol binding	0.0154	APOA1|ERLIN2|APOD|ANXA6|SCP2|NPC2	0.0021	GO.0015485	0.18124793
152	11	GO Component	endocytic vesicle membrane	0.0157	ATP6V0A1|RAB7A|ATP6V0D2|ATP6V0A4|AP2A2|ATP6V0C|LAMP1|RAB11B|CD9|UBA52|LAMP2	0.0039	GO.0030666	0.18041003
80	8	GO Function	alcohol binding	0.0158	APOA1|RBP5|ERLIN2|APOD|ANXA6|RBP4|SCP2|NPC2	0.0022	GO.0043178	0.18013429
99	9	GO Function	hydrolase activity, hydrolyzing O-glycosyl compounds	0.0158	LYZ|HEXB|ABHD10|AGL|GUSB|GAA|GM2A|PSAP|MGAM	0.0022	GO.0004553	0.18013429

141	11	GO Function	hydrolase activity, acting on carbon-nitrogen (but not peptide) bonds	0.0164	MTHFD1|GDA|DPYS|DDAH1|DPYSL2|GLS|AGMAT|ASAH1|NIT2|AMPD3|ACY1	0.0023	GO.0016810	0.17851562
16	4	GO Process	purine-containing compound salvage	0.0164	HPRT1|PNP|APRT|AMPD3	0.0016	GO.0043101	0.17851562
16	4	GO Process	mitochondrial fusion	0.0164	FIS1|CHCHD3|AFG3L2|OPA1	0.0016	GO.0008053	0.17851562
156	12	GO Process	protein localization to plasma membrane	0.0165	LAMA5|CDH1|RAB11A|SLC9A3R1|AMN|EHD3|BSG|EPB41L3|ATP1B1|FLNA|SCP2|KRT18	0.0017	GO.0072659	0.17825161
36	5	GO Component	other organism cell	0.0167	LTF|LGALS3|AQP1|UBA52|RAN	0.0042	GO.0044216	0.17772835
36	5	GO Component	other organism	0.0167	LTF|LGALS3|AQP1|UBA52|RAN	0.0042	GO.0044215	0.17772835
62	6	KEGG Pathways	Retinol metabolism	0.0299	UGT2B7|ADH1B|CYP4A11|UGT2B17|DHRS4|UGT1A9	0.0089	hsa00830	0.15243288
135	11	GO Process	cell junction assembly	0.0167	VCL|ACTN4|LAMA5|TLN1|EPB41L3|FLNA|CD9|ACTN1|ILK|RHOA|WDR1	0.0017	GO.0034329	0.17772835
665	32	GO Process	cellular chemical homeostasis	0.0168	FIS1|LTF|HEXB|SLC4A1|SLC9A3R1|ATP6V0A1|RAB7A|CALB1|RHCG|AFG3L2|ATP6V1A|FTH1|CA2|ATP6V0D2|CKB|ATP6V0A4|ACO1|ATP6V0C|RAB11B|ANXA6|ATP6V1H|OPA1|ATP1B1|GSTO1|GOT1|ATP6V1G1|CLIC4|IMMT|SLC4A4|EPHX2|ATP1A1|PLCG2	0.0017	GO.0055082	0.17746907
59	7	GO Process	myofibril assembly	0.0168	CFL2|MYH10|KRT19|PRKAR1A|MYH11|WDR1|KRT8	0.0017	GO.0030239	0.17746907
115	10	GO Process	negative regulation of supramolecular fiber organization	0.017	PFN2|VIL1|CAPG|ADD1|SCIN|GSN|HSPG2|PRKCD|TWF1|EML2	0.0017	GO.1902904	0.17695511
43	6	GO Process	establishment or maintenance of apical/basal cell polarity	0.017	SLC9A3R1|MSN|CLIC4|ILK|RHOA|WDR1	0.0017	GO.0035088	0.17695511
248	16	GO Process	steroid metabolic process	0.0172	APOA1|LRP2|SLC27A2|ERLIN2|ACAA2|UGT2B7|HSD11B2|UGT2B17|DHRS4|ACAA1|DHRS2|CYB5R1|SCP2|CUBN|AKR1C3|NPC2	0.0018	GO.0008202	0.17644716
53	6	GO Component	cortical actin cytoskeleton	0.0173	VCL|MYH9|CAPN2|KRT19|GSN|WDR1	0.0044	GO.0030864	0.17619539
884	39	GO Function	ion transmembrane transporter activity	0.0176	SFXN3|SLC25A3|ATP6V1B1|ATP6V1E1|SLC4A1|ATP6V0A1|NNT|VDAC1|SLC27A2|RHCG|ATP6V1A|ATP6V1B2|SLC25A4|ATP6V0D2|SLC23A1|ATP6V0A4|AQP1|SFXN1|SLC7A8|SLC5A2|ATP6V0C|BSG|CYB5A|ANXA6|ATP6V1H|ATP1B1|SFXN2|SLC25A5|ATP6V1G1|CLIC4|CLIC1|SLC3A2|SLC25A6|ATP5A1|SLC25A12|SLC4A4|ATP1A1|SLC25A10|SLC43A2	0.0025	GO.0015075	0.17544873
91	8	GO Component	basement membrane	0.0177	LAMA5|TINAG|COL18A1|COL4A2|HSPG2|COL4A1|TGFBI|VWA1	0.0046	GO.0005604	0.17520267
234	15	GO Function	sulfur compound binding	0.0179	OGDH|LTF|ACADL|GSTM3|DHTKD1|SERPINA5|PTGES2|SCP2|OGDHL|HMGCL|GPNMB|PC|MGST1|AOC1|ALDH6A1	0.0025	GO.1901681	0.1747147
47	6	GO Function	intramolecular oxidoreductase activity	0.0179	EHHADH|ECI1|PTGES2|TXNDC5|PDIA6|GPI	0.0026	GO.0016860	0.1747147
8	3	GO Function	hydroxymethyl-, formyl- and related transferase activity	0.0179	FTCD|SHMT1|SHMT2	0.0025	GO.0016742	0.1747147

8	3	GO Function	malate dehydrogenase activity	0.0179	MDH2|PHGDH|ME3	0.0025	GO.0016615	0.1747147
8	3	GO Function	acyl-CoA ligase activity	0.0179	ACSM2B|ACSF2|ACSM2A	0.0025	GO.0003996	0.1747147
373	21	GO Process	regulation of lipid metabolic process	0.018	ACADL|APOA1|CPT1A|ERLIN2|LAMTOR1|FABP1|CYP4A11|APOD|UGT1A9|ACADM|CPT2|SCP2|AKR1C3|PRKCD|PSAP|IDH1|ACSL1|EPHX2|ATP1A1|RAN|NPC2	0.0018	GO.0019216	0.17447275
1462	58	GO Function	ATP binding	0.0182	MYH9|MTHFD1|IDH3G|EHD4|ATP6V1B1|NARS|KHK|DNAJA3|SLC27A2|AFG3L2|ATP6V1A|ATP6V1B2|GLUD1|HSPA9|CKB|ACSS1|PKM|EHD3|ACSM2B|PKLR|PFKM|WARS|AGK|UBE2D3|VCP|MYH10|LONP1|KIF21A|MYO6|ABCD3|CMPK1|ASS1|PRPS1|VARS|NME2|PC|PRKCD|DAK|AK4|MYH11|ILK|ATP5A1|GK|ACSF2|CKMT2|CKMT1A|LARS2|ACSL1|NDUFA13|KIF13B|ATP1A1|MMAB|TWF1|ACSS3|ACSM2A|GALK1|DDX6|PSMC3	0.0027	GO.0005524	0.17399286
148	12	KEGG Pathways	Retrograde endocannabinoid signaling	0.0047	NDUFB4|NDUFA2|NDUFA10|NDUFA9|NDUFB9|NDUFB8|GNB2|NDUFV1|NDUFA12|NDUFS2|NDUFS1|NDUFA13	0.0011	hsa04723	0.23279021
7	3	GO Process	purine nucleotide salvage	0.0184	HPRT1|APRT|AMPD3	0.0019	GO.0032261	0.17351822
7	3	GO Process	fructose 1,6-bisphosphate metabolic process	0.0184	ALDOB|ALDOA|FBP1	0.0019	GO.0030388	0.17351822
44	6	GO Process	bicarbonate transport	0.0184	CA12|SLC4A1|CA2|AQP1|CYB5R1|SLC4A4	0.0019	GO.0015701	0.17351822
7	3	GO Process	L-serine biosynthetic process	0.0184	SHMT2|PHGDH|PSAT1	0.0019	GO.0006564	0.17351822
7	3	GO Process	leucine metabolic process	0.0184	BCAT2|HMGCL|AUH	0.0019	GO.0006551	0.17351822
123	10	GO Function	hydrolase activity, acting on glycosyl bonds	0.0185	DNPH1|LYZ|HEXB|ABHD10|AGL|GUSB|GAA|GM2A|PSAP|MGAM	0.0027	GO.0016798	0.17328283
84	11	KEGG Pathways	Rheumatoid arthritis	0.0002	ATP6V1D|ATP6V1B1|ATP6V1E1|ATP6V0A1|ATP6V1A|ATP6V1B2|ATP6V0D2|ATP6V0A4|ATP6V0C|ATP6V1H|ATP6V1G1	3.96E-05	hsa05323	0.369897
299	18	GO Process	urogenital system development	0.0186	DCN|CTSH|LAMA5|HRSP12|EPCAM|ENPEP|CALB1|IQGAP1|CA2|TMED10|RBP4|ASS1|COL4A1|PSAP|UMOD|ILK|MPST|MME	0.0019	GO.0001655	0.17304871
19	4	GO Function	ankyrin binding	0.0189	CDH1|SLC4A1|RHCG|ATP1A1	0.0028	GO.0030506	0.17235382
17	4	GO Process	arginine metabolic process	0.019	DDAH1|ASL|ASS1|AGMAT	0.002	GO.0006525	0.17212464
17	4	GO Process	regulation of oxidative phosphorylation	0.019	PPIF|SHMT2|VCP|RHOA	0.002	GO.0002082	0.17212464
182	13	GO Process	striated muscle cell differentiation	0.0193	MYH9|AFG3L2|UCHL1|CAPN2|CFL2|MYH10|KRT19|PRKAR1A|MYH11|GPX1|WDR1|KRT8|PDLIM5	0.002	GO.0051146	0.17144427
23	4	GO Component	microvillus membrane	0.0197	SLC9A3R1|MSN|DPEP1|PDZK1	0.0051	GO.0031528	0.17055338
11	3	GO Component	proteasome core complex, beta-subunit complex	0.0201	PSMB4|PSMB9|PSMB8	0.0053	GO.0019774	0.16968039
74	7	GO Component	recycling endosome membrane	0.0205	EHD4|RAB11A|PACSIN2|RAB17|EHD3|RAB11B|RAB14	0.0054	GO.0055038	0.16882461

119	10	GO Process	secondary alcohol metabolic process	0.0208	ACO2|IDH3G|APOA1|ERLIN2|ACAA2|IDH2|CUBN|IDH3B|IDH1|NPC2	0.0022	GO.1902652	0.16819367
353	20	GO Process	anion transmembrane transport	0.0208	SLC4A1|LRP2|VDAC1|CPT1A|SLC27A2|SLC25A4|SLC23A1|SLC7A8|BSG|ABCD3|CPT2|SLC25A5|CLIC4|CLIC1|SLC3A2|SLC25A6|SLC25A12|SLC4A4|SLC25A10|SLC43A2	0.0022	GO.0098656	0.16819367
85	8	GO Function	cysteine-type endopeptidase activity	0.0209	CTSH|CTSD|CAPNS1|TINAG|UCHL1|CAPN2|CTSB|CAPN1	0.0031	GO.0004197	0.16798537
62	7	GO Process	regulation of ATP metabolic process	0.0209	PPIF|ENO1|GPD1|SHMT2|VCP|RHOA|FBP1	0.0022	GO.1903578	0.16798537
162	12	GO Process	cellular response to metal ion	0.0212	PPIF|CDH1|ADD1|IQGAP1|AQP1|GSN|CLIC4|MT1M|AKR1C3|DPEP1|FBP1|AOC1	0.0022	GO.0071248	0.16736641
39	5	GO Component	multivesicular body	0.0216	CTSH|NAPSA|RAB11A|LAMP1|CD63	0.0057	GO.0005771	0.16655462
255	16	GO Process	aging	0.0216	DCN|CANX|DNAJA3|PCK1|APOD|COL4A2|LONP1|OPA1|ASS1|GSN|CNP|PRKCD|ABAT|ILK|DDC|MME	0.0023	GO.0007568	0.16655462
185	13	GO Process	cellular response to inorganic substance	0.0218	PPIF|CDH1|ADD1|IQGAP1|AQP1|HNRNPD|GSN|CLIC4|MT1M|AKR1C3|DPEP1|FBP1|AOC1	0.0023	GO.0071241	0.16615435
20	4	GO Function	metalloaminopeptidase activity	0.022	ENPEP|RNPEP|ANPEP|XPNPEP2	0.0033	GO.0070006	0.16575773
20	4	GO Function	complement binding	0.022	APCS|CD59|CFB|PHB	0.0033	GO.0001848	0.16575773
46	6	GO Process	alcohol catabolic process	0.0221	ALDH2|SORD|GPD2|ALDH1B1|AKR1C3|GK	0.0023	GO.0046164	0.16556077
18	4	GO Process	dicarboxylic acid catabolic process	0.0223	GOT2|GLUD1|GOT1|QPRT	0.0024	GO.0043649	0.16516951
31	5	GO Process	hydrogen peroxide metabolic process	0.0223	PRDX1|PRDX5|MAOB|GPX3|GPX1	0.0024	GO.0042743	0.16516951
31	5	GO Process	cristae formation	0.0223	CHCHD3|AFG3L2|APOOL|ATP5A1|IMMT	0.0024	GO.0042407	0.16516951
18	4	GO Process	glutamine family amino acid biosynthetic process	0.0223	GLUD1|ASL|GLS|ASS1	0.0024	GO.0009084	0.16516951
700	32	GO Function	calcium ion binding	0.0232	EPDR1|MYL12A|F9|EHD4|CAPNS1|CANX|VIL1|ACTN4|APCS|CDH1|LRP2|CALB1|IQGAP1|CAPN2|SCIN|CDH16|GPD2|AOC3|RHOT2|EHD3|ANXA6|PLS3|GSN|HSPG2|ASPN|CUBN|ACTN1|UMOD|SLC25A12|ANXA11|AOC1|CAPN1	0.0036	GO.0005509	0.1634512
383	21	GO Process	supramolecular fiber organization	0.0232	VIL1|ACTN4|RAB11A|ADD1|SCIN|COL14A1|CFL2|PLS3|MYH10|KRT19|DPT|FLNA|GSN|PRKAR1A|ACTN1|ALDOA|MYH11|ILK|RHOA|WDR1|KRT8	0.0025	GO.0097435	0.1634512
33	5	KEGG Pathways	Starch and sucrose metabolism	0.012	PYGB|AGL|GAA|GPI|MGAM	0.003	hsa00500	0.19208188
8	3	GO Process	positive regulation of early endosome to late endosome transport	0.0237	RAB21|DAB2|MSN	0.0025	GO.2000643	0.16252517
8	3	GO Process	actin filament severing	0.0237	VIL1|SCIN|GSN	0.0025	GO.0051014	0.16252517
8	3	GO Process	purine ribonucleoside catabolic process	0.0237	AHCY|HPRT1|PNP	0.0025	GO.0046130	0.16252517

8	3	GO Process	actin filament depolymerization	0.0237	VIL1|CFL2|WDR1	0.0025	GO.0030042	0.16252517
143	11	GO Process	regulation of cell shape	0.0237	MYH9|VIL1|HEXB|SLC9A3R1|PARVA|EPB41L3|MSN|MYH10|ALDOA|SEPT7|WDR1	0.0026	GO.0008360	0.16252517
673	31	GO Function	lipid binding	0.0238	LTF|APOA1|PFN2|GOT2|VIL1|HRSP12|PACSIN2|CALB1|RBP5|IQGAP1|ERLIN2|PSMB4|FABP1|SCIN|TLN1|HSD11B2|AP2A2|SERPINA5|APOD|ANXA6|UGT1A9|VCP|OPA1|RBP4|SCP2|DPEP1|PSAP|ANXA11|ATP1A1|TWF1|NPC2	0.0037	GO.0008289	0.1623423
122	10	GO Process	negative regulation of protein complex assembly	0.0238	PFN2|VIL1|CAPG|ADD1|SCIN|GSN|PRKCD|LAMP2|TWF1|EML2	0.0026	GO.0031333	0.1623423
3	2	GO Component	myosin II filament	0.0244	MYH9|MYH10	0.0065	GO.0097513	0.16126102
530	25	GO Component	axon	0.0244	PFN2|CANX|HSPB1|RAB21|RAB11A|LRP2|CALB1|IQGAP1|UCHL1|CA2|TBC1D24|EPB41L3|MYH10|OPA1|GOT1|ACADM|HNRNPR|ILK|GDI1|RHOA|DDC|MME|KIF13B|PHB2|NDRG2	0.0066	GO.0030424	0.16126102
3	2	GO Component	collagen type VI trimer	0.0244	DCN|COL6A1	0.0065	GO.0005589	0.16126102
21	4	GO Function	fatty acid ligase activity	0.0246	SLC27A2|ACSM2B|ACSL1|ACSM2A	0.0038	GO.0015645	0.16090649
10	4	KEGG Pathways	Sulfur metabolism	0.0017	SQRDL|CYCS|MPST|TST	0.00038	hsa00920	0.27695511
166	12	GO Process	protein stabilization	0.0248	CDC37|APOA1|PFN2|DNAJA3|LAMP1|ATP1B1|FLNA|RPL5|PRKCD|LAMP2|PHB2|PHB	0.0027	GO.0050821	0.16055483
83	8	GO Process	iron ion homeostasis	0.0249	SFXN3|LTF|ATP6V1A|FTH1|ACO1|SFXN1|SFXN2|ATP6V1G1	0.0027	GO.0055072	0.16038007
83	8	GO Process	maintenance of location in cell	0.0249	FTH1|SCIN|GAA|TLN1|EPB41L3|GSN|FLNB|TWF1	0.0027	GO.0051651	0.16038007
386	21	GO Process	peptide biosynthetic process	0.0249	RPS16|NARS|APEH|RPS9|TSFM|CNDP2|PPA2|WARS|MRPL37|MRPL24|MRPL21|RPL5|VARS|HAGH|GGT5|GGT1|UBA52|LARS2|MRPS22|GFM1|RPL18	0.0027	GO.0043043	0.16038007
123	10	GO Process	liver development	0.0249	ACO2|GNPNAT1|RPS16|QDPR|HNRNPD|PKM|ASS1|HMGCL|AK4|MPST	0.0027	GO.0001889	0.16038007
575	28	GO Process	lipid biosynthetic process	0.025	APOA1|HEXB|MECR|ALDH8A1|SLC27A2|GPD1L|ACAA2|CBR1|GPD1|HSD11B2|ACSS1|PCK1|ACSM2B|PTGES2|VAPA|ALDH3A2|AGK|CYB5R1|ABCD3|SCP2|RAB14|AKR1C3|SACM1L|GK|ACSL1|EPHX2|ACSM2A|PLCG2	0.0027	GO.0008610	0.160206
2	2	GO Function	purine phosphoribosyltransferase activity	0.0254	HPRT1|APRT	0.004	GO.0106130	0.15951663
2	2	GO Function	oxidoreductase activity, acting on the CH-NH group of donors, flavin as acceptor	0.0254	DMGDH|SARDH	0.004	GO.0046997	0.15951663
2	2	GO Function	electron transporter, transferring electrons from CoQH2-cytochrome c reductase complex and cytochrome c oxidase complex activity	0.0254	CYCS|CYC1	0.004	GO.0045155	0.15951663

2	2	GO Function	L-allo-threonine aldolase activity	0.0254	SHMT1|SHMT2	0.004	GO.0008732	0.15951663
2	2	GO Function	pyruvate kinase activity	0.0254	PKM|PKLR	0.004	GO.0004743	0.15951663
2	2	GO Function	phosphoenolpyruvate carboxykinase (GTP) activity	0.0254	PCK2|PCK1	0.004	GO.0004613	0.15951663
2	2	GO Function	isocitrate dehydrogenase (NADP+) activity	0.0254	IDH2|IDH1	0.004	GO.0004450	0.15951663
2	2	GO Function	glycine hydroxymethyltransferase activity	0.0254	SHMT1|SHMT2	0.004	GO.0004372	0.15951663
2	2	GO Function	glycerol-3-phosphate dehydrogenase (quinone) activity	0.0254	GPD1|GPD2	0.004	GO.0004368	0.15951663
2	2	GO Function	glycerol-3-phosphate dehydrogenase [NAD+] activity	0.0254	GPD1L|GPD1	0.004	GO.0004367	0.15951663
2	2	GO Function	dihydropyrimidinase activity	0.0254	DPYS|DPYSL2	0.004	GO.0004157	0.15951663
2	2	GO Function	aconitate hydratase activity	0.0254	ACO2|ACO1	0.004	GO.0003994	0.15951663
2	2	GO Function	leukotriene-C(4) hydrolase	0.0254	GGT5|GGT1	0.004	GO.0002951	0.15951663
2	2	GO Function	peptidyltransferase activity	0.0254	GGT5|GGT1	0.004	GO.0000048	0.15951663
103	9	GO Process	reactive oxygen species metabolic process	0.0255	PRDX1|NNT|PRDX5|MAOB|GPX3|NDUFS1|GPX1|NDUFA13|EPHX2	0.0028	GO.0072593	0.15934598
19	4	GO Process	cellular glucuronidation	0.0255	ABHD10|UGT2B7|UGT2B17|UGT1A9	0.0028	GO.0052695	0.15934598
65	7	GO Process	vesicle coating	0.0255	TFG|NAPA|TMED10|RAB1B|TMED9|CD59|SERPINA1	0.0028	GO.0006901	0.15934598
71	7	GO Function	modified amino acid binding	0.0256	GSTM3|FTCD|SCIN|TLN1|PTGES2|DPEP1|MGST1	0.0044	GO.0072341	0.159176

<table>
<tr><td>5254</td><td>168</td><td>GO Component</td><td>cell periphery</td><td>0.0257</td><td>RALA|SYPL1|GNA11|HSD17B10|CA12|VCL|NIPSNAP1|MYH9|ATP6V1D|F9|EHD4|SLC25A3|ATP6V1B1|ENO1|APOA1|GOT2|CAPNS1|VIL1|HSPB1|ACTN4|ATP6V1E1|LGALS3|RAB21|CDH1|RAB11A|DNAJA3|SLC4A1|SLC9A3R1|PACSIN2|NAPA|EPCAM|LRP2|GRB14|RAB17|ATP6V0A1|ADD1|SCARB2|RAB7A|AMBP|ENPEP|VDAC1|SLC27A2|RHCG|IQGAP1|BCAM|ARL8A|ATP6V1A|ATP6V1B2|ERLIN2|LAMTOR1|SLC25A4|GPD1L|CA2|ATP6V0D2|FTCD|TBC1D24|CAPN2|RNPEP|FABP1|SCIN|AMN|CDH16|ANPEP|COL6A2|SLC23A1|TMED10|DCXR|HINT1|GNB2|GAA|ADH1B|ATP6V0A4|DPYSL2|CYP4A11|AQP1|RAB6A|AOC3|DAB2|TOLLIP|TLN1|SLC7A8|RHOT2|EHD3|AP2A2|SLC5A2|ATP6V0C|SERPINA5|LAMP1|BSG|PARVA|EPB41L3|VAPA|PFKM|PLS3|GM2A|UBE2D3|ATP6V1H|MSN|MYH10|DPP4|KIF21A|KRT19|COL6A1|CYB5R1|ATP1B1|FLNA|MYO6|XPNPEP2|SLC25A5|AKR1A1|GSN|RAB14|ATP6V1G1|CLIC4|HSPG2|CLIC1|KCTD12|CUBN|SLC3A2|CKAP4|GLDC|GPNMB|CD9|KRT18|SACM1L|PRKAR1A|DPEP1|NME2|CNP|FHL1|ACTN1|PRKCD|PSAP|KTN1|CD59|UMOD|MGST1|ILK|GGT5|ATP5A1|SEPT7|GGT1|PDIA6|UBA52|SLC4A4|PDZK1|RHOA|GPI|LAMP2|AOC1|TOM1|TGFBI|CFB|MME|FLNB|ACSL1|WDR1|CAPN1|PHB2|ATP1A1|MGAM|CD63|KRT8|TWF1|SLC43A2|PHB|PDLIM5|PLCG2</td><td>0.0071</td><td>GO.0071944</td><td>0.15900669</td></tr>
<tr><td>61</td><td>13</td><td>KEGG Pathways</td><td>Synaptic vesicle cycle</td><td>5.3E-07</td><td>ATP6V1D|ATP6V1B1|ATP6V1E1|NAPA|ATP6V0A1|ATP6V1A|ATP6V1B2|ATP6V0D2|ATP6V0A4|AP2A2|ATP6V0C|ATP6V1H|ATP6V1G1</td><td>5.95E-08</td><td>hsa04721</td><td>0.62749055</td></tr>
<tr><td>104</td><td>9</td><td>GO Process</td><td>fatty acid biosynthetic process</td><td>0.0269</td><td>MECR|CBR1|ACSS1|ACSM2B|PTGES2|ABCD3|AKR1C3|EPHX2|ACSM2A</td><td>0.003</td><td>GO.0006633</td><td>0.15702477</td></tr>
<tr><td>79</td><td>7</td><td>GO Component</td><td>mitochondrial intermembrane space</td><td>0.027</td><td>REXO2|CYCS|SHMT2|AGK|OPA1|GATM|NDUFS1</td><td>0.0075</td><td>GO.0005758</td><td>0.15686362</td></tr>
<tr><td>37</td><td>5</td><td>GO Function</td><td>ATPase activity, coupled to transmembrane movement of ions, phosphorylative mechanism</td><td>0.0271</td><td>ATP6V1E1|ATP6V0D2|ATP1B1|ATP6V1G1|ATP1A1</td><td>0.0047</td><td>GO.0015662</td><td>0.15670307</td></tr>
<tr><td>720</td><td>33</td><td>GO Process</td><td>cellular component morphogenesis</td><td>0.0271</td><td>VCL|MYH9|FIS1|LAMA5|SLC9A3R1|PACSIN2|LRP2|ADD1|IQGAP1|AFG3L2|UCHL1|CFL2|HPRT1|DPYSL2|PARVA|EPB41L3|COL18A1|MYH10|OPA1|KRT19|FLNA|CLIC4|CD9|PRKAR1A|CNP|ACTN1|MYH11|ILK|RHOA|SSBP1|FLNB|WDR1|KRT8</td><td>0.003</td><td>GO.0032989</td><td>0.15670307</td></tr>
<tr><td>1043</td><td>42</td><td>GO Component</td><td>nuclear outer membrane-endoplasmic reticulum membrane network</td><td>0.0285</td><td>REEP6|CANX|RAB21|RAB2A|SCARB2|SLC27A2|NAT8|ERLIN2|UCHL1|HPD|FTCD|TMED10|UGT2B7|RAB1B|CYP4A11|RAB6A|HSD11B2|UGT2B17|DHRS4|COPB2|TMED9|CYB5A|VAPA|ALDH3A2|UGT1A9|VCP|CYB5R1|MGST3|RAB14|CKAP4|SACM1L|KTN1|CD59|MGST1|PDIA6|UBA52|RHOA|TMED4|TMEM33|ACSL1|EPHX1|FMO1</td><td>0.0079</td><td>GO.0042175</td><td>0.15451551</td></tr>
<tr><td>170</td><td>12</td><td>GO Process</td><td>carboxylic acid transmembrane transport</td><td>0.029</td><td>LRP2|CPT1A|SLC27A2|SLC23A1|SLC7A8|BSG|ABCD3|CPT2|SLC3A2|SLC25A12|SLC25A10|SLC43A2</td><td>0.0032</td><td>GO.1905039</td><td>0.1537602</td></tr>
<tr><td>148</td><td>11</td><td>GO Process</td><td>regulation of mitochondrion organization</td><td>0.0293</td><td>DCN|CDC37|FIS1|PPIF|VDAC1|ACAA2|YWHAG|OPA1|SLC25A5|GPX1|FAM162A</td><td>0.0033</td><td>GO.0010821</td><td>0.15331324</td></tr>
<tr><td>393</td><td>21</td><td>GO Process</td><td>regulation of endopeptidase activity</td><td>0.0295</td><td>CTSH|FIS1|LTF|VIL1|DNAJA3|AMBP|PRDX5|FABP1|CYCS|AQP1|SERPINA5|VCP|GSN|PSMB9|PSMB8|DPEP1|RHOA|GPX1|SERPINA1|FAM162A|NDUFA13</td><td>0.0033</td><td>GO.0052548</td><td>0.1530178</td></tr>
<tr><td>20</td><td>4</td><td>GO Process</td><td>hydrogen peroxide catabolic process</td><td>0.0295</td><td>PRDX1|PRDX5|GPX3|GPX1</td><td>0.0033</td><td>GO.0042744</td><td>0.1530178</td></tr>
</table>

20	4	GO Process	response to hydroperoxide	0.0295	GPX3|PRKCD|MGST1|GPX1	0.0033	GO.0033194	0.1530178
9	3	GO Process	ketone body biosynthetic process	0.0296	HMGCL|BDH1|ACSS3	0.0033	GO.0046951	0.15287083
9	3	GO Process	negative regulation by host of viral process	0.0296	LTF|APCS|VAPA	0.0033	GO.0044793	0.15287083
9	3	GO Process	water-soluble vitamin biosynthetic process	0.0296	AKR1A1|PSAT1|MMAB	0.0033	GO.0042364	0.15287083
9	3	GO Process	high-density lipoprotein particle clearance	0.0296	APOA1|AMN|CUBN	0.0033	GO.0034384	0.15287083
9	3	GO Process	ADP transport	0.0296	SLC25A4|SLC25A5|SLC25A6	0.0033	GO.0015866	0.15287083
9	3	GO Process	valine metabolic process	0.0296	BCAT2|HIBCH|ALDH6A1	0.0033	GO.0006573	0.15287083
9	3	GO Process	glutamate catabolic process	0.0296	GOT2|GLUD1|GOT1	0.0033	GO.0006538	0.15287083
9	3	GO Process	fructose 6-phosphate metabolic process	0.0296	TALDO1|PFKM|FBP1	0.0033	GO.0006002	0.15287083
228	19	KEGG Pathways	Thermogenesis	0.00016	NDUFB4|NDUFA2|NDUFA10|CPT1A|NDUFA9|NDUFB9|NDUFB8|UQCRH|CYC1|NDUFV1|NDUFA12|UQCR10|COA3|NDUFS2|CPT2|ATP5A1|NDUFS1|ACSL1|NDUFA13	3.12E-05	hsa04714	0.379588
11	3	GO Function	glutathione binding	0.0303	GSTM3|PTGES2|MGST1	0.0053	GO.0043295	0.15185574
395	21	GO Process	gland development	0.0305	ACO2|GNPNAT1|APOA1|GOT2|RPS16|LAMA5|CDH1|SLC9A3R1|QDPR|HNRNPD|PKM|SERPINA5|MSN|ASS1|HMGCL|APRT|PSAP|AK4|MPST|CAPN1|PHB2	0.0035	GO.0048732	0.15157002
1203	49	GO Process	proteolysis	0.0305	PSMA4|MYH9|F9|PSMD7|CTSH|LTF|CTSD|PEPD|CAPNS1|PMPCB|NAPSA|TINAG|GGH|PSMD11|PSMD3|ENPEP|AFG3L2|TINAGL1|PSMA5|ERLIN2|UCHL1|PSMB4|CAPN2|RNPEP|APEH|TPP1|ANPEP|TOLLIP|CNDP2|SHMT2|CTSB|UBE2D3|VCP|DPP4|LONP1|XPNPEP2|PSMB9|PSMB8|DPEP1|GGT5|GGT1|ACY1|UBA52|SCRN1|RHOA|CFB|MME|CAPN1|PSMC3	0.0035	GO.0006508	0.15157002
68	7	GO Process	COPII-coated vesicle budding	0.0308	TFG|NAPA|TMED10|RAB1B|VAPA|CD59|SERPINA1	0.0035	GO.0090114	0.15114493
68	7	GO Process	regulation of mitochondrial membrane potential	0.0308	DCN|NNT|PPA2|VCP|GOT1|CLIC1|NDUFS1	0.0035	GO.0051881	0.15114493
68	7	GO Process	vesicle targeting, to, from or within Golgi	0.0308	TFG|NAPA|TMED10|RAB1B|TMED9|CD59|SERPINA1	0.0035	GO.0048199	0.15114493
107	9	GO Process	renal system process	0.0308	RAB11A|SLC9A3R1|CYP4A11|AQP1|HSD11B2|GSN|AKR1C3|PRKAR1A|RHOA	0.0035	GO.0003014	0.15114493
1049	43	GO Function	transmembrane transporter activity	0.0311	ATP6V1D|SFXN3|SLC25A3|ATP6V1B1|ATP6V1E1|SLC4A1|ATP6V0A1|NNT|VDAC1|SLC27A2|RHCG|ATP6V1A|ATP6V1B2|SLC25A4|ATP6V0D2|SLC23A1|ATP6V0A4|AQP1|SFXN1|SLC7A8|SLC5A2|ATP6V0C|BSG|CYB5A|ANXA6|ATP6V1H|ATP1B1|SFXN2|ABCD3|RBP4|SLC25A5|ATP6V1G1|CLIC4|CLIC1|SLC3A2|SLC25A6|ATP5A1|SLC25A12|SLC4A4|TOMM40|ATP1A1|SLC25A10|SLC43A2	0.0055	GO.0022857	0.15072396
10	3	Reactome Pathways	Erythrocytes take up carbon dioxide and release oxygen	0.0313	SLC4A1|CA2|AQP1	0.0042	HSA-1237044	0.15044557

125	9	GO Component	late endosome membrane	0.0321	ARL8B|SCARB2|RAB7A|ARL8A|LAMTOR1|LAMTOR4|ANXA6|LAMP2|CD63	0.009	GO.0031902	0.1493495
129	10	GO Process	response to calcium ion	0.0324	PPIF|ADD1|IQGAP1|HNRNPD|CLIC4|AKR1C3|DPEP1|SLC25A12|GDI1|ANXA11	0.0037	GO.0051592	0.1489455
1022	41	GO Component	endoplasmic reticulum membrane	0.0326	REEP6|CANX|RAB21|RAB2A|SCARB2|SLC27A2|NAT8|ERLIN2|UCHL1|HPD|FTCD|TMED10|UGT2B7|RAB1B|CYP4A11|RAB6A|HSD11B2|UGT2B17|DHRS4|COPB2|TMED9|CYB5A|VAPA|ALDH3A2|UGT1A9|VCP|CYB5R1|MGST3|CKAP4|SACM1L|KTN1|CD59|MGST1|PDIA6|UBA52|RHOA|TMED4|TMEM33|ACSL1|EPHX1|FMO1	0.0092	GO.0005789	0.14867824
69	7	GO Process	fatty acid transport	0.0329	GOT2|CPT1A|SLC27A2|FABP1|ABCD3|CPT2|ACSL1	0.0038	GO.0015908	0.14828041
69	7	GO Process	triglyceride metabolic process	0.0329	APOA1|CPT1A|FABP1|PCK1|GK|GPX1|ACSL1	0.0038	GO.0006641	0.14828041
245	15	GO Process	negative regulation of cell adhesion	0.033	APOA1|ACTN4|LGALS3|CDH1|EPCAM|ARHGDIA|APOD|ASS1|GPNMB|CD9|PRKAR1A|PRKCD|ABAT|RHOA|TGFBI	0.0038	GO.0007162	0.14814861
618	29	GO Process	inorganic cation transmembrane transport	0.0331	SLC25A3|ATP6V1B1|PMPCB|ATP6V1E1|SLC4A1|ATP6V0A1|NNT|AFG3L2|ATP6V1A|ATP6V1B2|ATP6V0D2|SLC23A1|ATP6V0A4|AQP1|CYC1|SLC5A2|ATP6V0C|CYB5A|ATP6V1H|OPA1|ATP1B1|ATP6V1G1|SLC3A2|ATP5A1|SLC4A4|PHB2|ATP1A1|PHB|PLCG2	0.0038	GO.0098662	0.1480172
63	6	GO Component	clathrin-coated pit	0.0333	LRP2|AMN|DAB2|AP2A2|MYO6|CUBN	0.0095	GO.0005905	0.14775558
4	2	GO Component	terminal web	0.0336	VCL|KRT19	0.0096	GO.1990357	0.14736607
1025	41	GO Component	endoplasmic reticulum subcompartment	0.0336	REEP6|CANX|RAB21|RAB2A|SCARB2|SLC27A2|NAT8|ERLIN2|UCHL1|HPD|FTCD|TMED10|UGT2B7|RAB1B|CYP4A11|RAB6A|HSD11B2|UGT2B17|DHRS4|COPB2|TMED9|CYB5A|VAPA|ALDH3A2|UGT1A9|VCP|CYB5R1|MGST3|CKAP4|SACM1L|KTN1|CD59|MGST1|PDIA6|UBA52|RHOA|TMED4|TMEM33|ACSL1|EPHX1|FMO1	0.0096	GO.0098827	0.14736607
4	2	GO Component	mitochondrial permeability transition pore complex	0.0336	PPIF|VDAC1	0.0096	GO.0005757	0.14736607
109	9	GO Process	cellular hormone metabolic process	0.034	TTR|ALDH8A1|UGT2B7|DHRS4|DHRS2|UGT1A9|RBP4|SCP2|AKR1C3	0.004	GO.0034754	0.14685211
2	2	GO Process	positive regulation of protein processing in phagocytic vesicle	0.0342	MYH9|GSN	0.004	GO.1903923	0.14659739
2	2	GO Process	protein localization to bicellular tight junction	0.0342	ACTN4|FLNA	0.004	GO.1902396	0.14659739
707	32	GO Process	inorganic ion transmembrane transport	0.0342	SLC25A3|ATP6V1B1|PMPCB|ATP6V1E1|SLC4A1|ATP6V0A1|NNT|AFG3L2|ATP6V1A|ATP6V1B2|ATP6V0D2|SLC23A1|ATP6V0A4|AQP1|CYC1|SLC5A2|ATP6V0C|CYB5A|ATP6V1H|OPA1|ATP1B1|ATP6V1G1|CLIC4|CLIC1|SLC3A2|ATP5A1|SLC4A4|PHB2|ATP1A1|SLC25A10|PHB|PLCG2	0.0041	GO.0098660	0.14659739
2	2	GO Process	glycolytic process through glucose-1-phosphate	0.0342	PFKM|GALK1	0.004	GO.0061622	0.14659739
2	2	GO Process	glycerol-3-phosphate catabolic process	0.0342	GPD1L|GPD1	0.004	GO.0046168	0.14659739
2	2	GO Process	urate biosynthetic process	0.0342	PNP|PRPS1	0.004	GO.0034418	0.14659739

2	2	GO Process	regulation of isoprenoid metabolic process	0.0342	AKR1C3|NPC2	0.004	GO.0019747	0.14659739
2	2	GO Process	glutamate catabolic process to 2-oxoglutarate	0.0342	GOT2|GOT1	0.004	GO.0019551	0.14659739
2	2	GO Process	glutamate catabolic process to aspartate	0.0342	GOT2|GOT1	0.004	GO.0019550	0.14659739
2	2	GO Process	glycine betaine biosynthetic process from choline	0.0342	CHDH|ALDH7A1	0.004	GO.0019285	0.14659739
2	2	GO Process	glycine biosynthetic process from serine	0.0342	SHMT1|SHMT2	0.004	GO.0019264	0.14659739
2	2	GO Process	viral RNA genome packaging	0.0342	PC|DDX6	0.004	GO.0019074	0.14659739
2	2	GO Process	cyanate catabolic process	0.0342	MPST|TST	0.004	GO.0009440	0.14659739
175	12	GO Process	endoplasmic reticulum to Golgi vesicle-mediated transport	0.0342	F9|TFG|RAB2A|NAPA|TMED10|RAB1B|COPB2|TMED9|VAPA|VCP|CD59|SERPINA1	0.004	GO.0006888	0.14659739
2	2	GO Process	adenine salvage	0.0342	HPRT1|APRT	0.004	GO.0006168	0.14659739
2	2	GO Process	glycerophosphate shuttle	0.0342	GPD1|GPD2	0.004	GO.0006127	0.14659739
2	2	GO Process	glyoxylate cycle	0.0342	IDH2|IDH1	0.004	GO.0006097	0.14659739
2	2	GO Process	xylulose biosynthetic process	0.0342	TALDO1|TKT	0.004	GO.0005999	0.14659739
57	6	GO Function	metalloexopeptidase activity	0.0347	PEPD|ENPEP|RNPEP|ANPEP|XPNPEP2|DPEP1	0.0061	GO.0008235	0.14596705
11	3	Reactome Pathways	Cytosolic tRNA aminoacylation	0.0347	NARS|WARS|VARS	0.0053	HSA-379716	0.14596705
10	3	GO Process	vacuolar proton-transporting V-type ATPase complex assembly	0.0348	ATP6V0A1|ATP6V0A4|ALDOB	0.0042	GO.0070072	0.14584208
10	3	GO Process	histidine metabolic process	0.0348	MTHFD1|HNMT|FTCD	0.0042	GO.0006547	0.14584208
90	8	GO Process	regulation of nucleotide metabolic process	0.0351	PPIF|ENO1|GPD1|SHMT2|VCP|BPGM|RHOA|FBP1	0.0043	GO.0006140	0.14546929
73	7	KEGG Pathways	Thyroid hormone synthesis	0.0183	TTR|CANX|LRP2|ATP1B1|GPX3|GPX1|ATP1A1	0.0051	hsa04918	0.17375489
325	18	GO Process	Golgi vesicle transport	0.0362	F9|TFG|RAB2A|NAPA|AMN|TMED10|RAB1B|RAB6A|EHD3|COPB2|TMED9|LAMP1|VAPA|VCP|RAB14|KRT18|CD59|SERPINA1	0.0044	GO.0048193	0.14412914
22	4	GO Process	mitochondrial genome maintenance	0.0363	DNAJA3|SLC25A4|LONP1|OPA1	0.0045	GO.0000002	0.14400934

3	2	GO Function	medium-chain-acyl-CoA dehydrogenase activity	0.0366	ACAD11|ACADM	0.0065	GO.0070991	0.14365189
3	2	GO Function	3 iron, 4 sulfur cluster binding	0.0366	ACO2|ACO1	0.0065	GO.0051538	0.14365189
3	2	GO Function	15-oxoprostaglandin 13-oxidase activity	0.0366	PTGR1|PTGR2	0.0065	GO.0047522	0.14365189
3	2	GO Function	13-prostaglandin reductase activity	0.0366	PTGR1|PTGR2	0.0065	GO.0036132	0.14365189
3	2	GO Function	maltose alpha-glucosidase activity	0.0366	GAA|MGAM	0.0065	GO.0032450	0.14365189
3	2	GO Function	oxidoreductase activity, acting on the CH-OH group of donors, oxygen as acceptor	0.0366	ALDH3A2|HAO2	0.0065	GO.0016899	0.14365189
3	2	GO Function	oxidoreductase activity, acting on a sulfur group of donors, quinone or similar compound as acceptor	0.0366	SQRDL|GSTO1	0.0065	GO.0016672	0.14365189
3	2	GO Function	oxidoreductase activity, acting on the CH-NH2 group of donors, NAD or NADP as acceptor	0.0366	CRYM|GLUD1	0.0065	GO.0016639	0.14365189
3	2	GO Function	palmitoyl-CoA oxidase activity	0.0366	ACADL|ACAA1	0.0065	GO.0016401	0.14365189
3	2	GO Function	protein kinase C inhibitor activity	0.0366	HSPB1|YWHAG	0.0065	GO.0008426	0.14365189
3	2	GO Function	thiosulfate sulfurtransferase activity	0.0366	MPST|TST	0.0065	GO.0004792	0.14365189
3	2	GO Function	pyruvate dehydrogenase (acetyl-transferring) activity	0.0366	PDHB|PDHA1	0.0065	GO.0004739	0.14365189
3	2	GO Function	fructose-bisphosphate aldolase activity	0.0366	ALDOB|ALDOA	0.0065	GO.0004332	0.14365189
3	2	GO Function	dodecenoyl-CoA delta-isomerase activity	0.0366	EHHADH|ECI1	0.0065	GO.0004165	0.14365189

3	2	GO Function	L-aspartate:2-oxoglutarate aminotransferase activity	0.0366	GOT2|GOT1	0.0065	GO.0004069	0.14365189
3	2	GO Function	acetate-CoA ligase activity	0.0366	ACSS1|ACSS3	0.0065	GO.0003987	0.14365189
3	2	GO Function	purine nucleobase binding	0.0366	PNP|APRT	0.0065	GO.0002060	0.14365189
155	11	GO Process	drug transport	0.0369	LRP2|SLC25A4|CA2|AMN|AQP1|SLC25A5|CUBN|SLC25A6|SLC25A12|PDZK1|SLC25A10	0.0045	GO.0015893	0.14329736
405	21	GO Process	protein localization to membrane	0.0372	RPS16|LAMA5|CDH1|RAB11A|DNAJA3|SLC9A3R1|AMN|RPS9|EHD3|RAB11B|BSG|EPB41L3|AGK|ATP1B1|FLNA|RPL5|SCP2|KRT18|UBA52|NDUFA13|RPL18	0.0046	GO.0072657	0.14294571
570	27	GO Process	cellular cation homeostasis	0.0374	FIS1|LTF|HEXB|SLC4A1|ATP6V0A1|RAB7A|CALB1|AFG3L2|ATP6V1A|FTH1|CA2|ATP6V0D2|ATP6V0A4|ACO1|ATP6V0C|ANXA6|ATP6V1H|ATP1B1|GSTO1|GOT1|ATP6V1G1|CLIC4|IMMT|SLC4A4|EPHX2|ATP1A1|PLCG2	0.0046	GO.0030003	0.14271284
37	5	GO Process	response to glucagon	0.0379	QDPR|CYC1|PCK1|ASS1|PRKAR1A	0.0047	GO.0033762	0.14213608
112	9	GO Process	regulation of steroid metabolic process	0.0379	ACADL|APOA1|ERLIN2|LAMTOR1|SCP2|AKR1C3|EPHX2|ATP1A1|RAN	0.0047	GO.0019218	0.14213608
37	5	GO Process	virion assembly	0.0379	RAB1B|PC|UBA52|PPIA|DDX6	0.0047	GO.0019068	0.14213608
37	5	GO Process	morphogenesis of a polarized epithelium	0.0379	LAMA5|SLC9A3R1|MSN|RHOA|WDR1	0.0047	GO.0001738	0.14213608
251	15	GO Process	kidney development	0.038	DCN|CTSH|LAMA5|HRSP12|EPCAM|ENPEP|CALB1|IQGAP1|CA2|TMED10|ASS1|UMOD|ILK|MPST|MME	0.0047	GO.0001822	0.14202164
40	13	KEGG Pathways	Tryptophan metabolism	1.2E-08	OGDH|EHHADH|ALDH2|ALDH3A2|ALDH9A1|ECHS1|OGDHL|ALDH1B1|MAOB|ALDH7A1|DDC|AOC1|HADH	7.91E-10	hsa00380	0.7935542
26	4	GO Function	oxidoreductase activity, acting on the CH-CH group of donors, NAD or NADP as acceptor	0.0384	MECR|AKR1C3|PTGR1|PTGR2	0.0075	GO.0016628	0.14156688
54	6	GO Process	long-chain fatty acid transport	0.0387	CPT1A|SLC27A2|FABP1|ABCD3|CPT2|ACSL1	0.0048	GO.0015909	0.1412289
13	3	GO Function	NADPH binding	0.0397	QDPR|GRHPR|CRYZ	0.0078	GO.0070402	0.14012095
80	7	GO Function	ATPase binding	0.0397	RALA|ATP6V1E1|ATP6V0A1|ATP6V0A4|ATP1B1|ATP6V1G1|ALDOB	0.008	GO.0051117	0.14012095
13	3	GO Function	potassium ion binding	0.0397	PKM|PKLR|ATP1A1	0.0078	GO.0030955	0.14012095
13	3	GO Function	carbon-nitrogen lyase activity	0.0397	APMAP|FTCD|ASL	0.0078	GO.0016840	0.14012095
122	9	GO Function	integrin binding	0.0397	ACTN4|LAMA5|TLN1|HSPG2|GPNMB|CD9|ACTN1|ILK|TGFBI	0.0078	GO.0005178	0.14012095
30	4	GO Component	podosome	0.0403	VCL|SCIN|GSN|WDR1	0.0117	GO.0002102	0.1394695
43	5	GO Function	disulfide oxidoreductase activity	0.0409	TXNL1|PTGES2|GSTO1|TXNRD2|PDIA6	0.0083	GO.0015036	0.13882767
23	4	GO Process	mitochondrial calcium ion homeostasis	0.0411	FIS1|AFG3L2|ANXA6|IMMT	0.0051	GO.0051560	0.13861582

23	4	GO Process	positive regulation of lipid catabolic process	0.0411	APOA1|CPT1A|FABP1|PRKCD	0.0051	GO.0050996	0.13861582
23	4	GO Process	modulation by host of viral process	0.0411	LTF|APCS|VAPA|PC	0.0051	GO.0044788	0.13861582
38	5	GO Process	glycosyl compound catabolic process	0.0414	AHCY|ABHD10|DPYS|HPRT1|PNP	0.0052	GO.1901658	0.13829997
38	5	GO Process	positive regulation of calcium ion transmembrane transporter activity	0.0414	GBAS|EHD3|ATP1B1|GSTO1|PLCG2	0.0052	GO.1901021	0.13829997
38	5	GO Process	sarcomere organization	0.0414	CFL2|KRT19|PRKAR1A|WDR1|KRT8	0.0052	GO.0045214	0.13829997
55	6	GO Process	lysosome organization	0.0415	HEXB|RAB7A|LAMTOR1|TPP1|GAA|ATP6V0C	0.0052	GO.0007040	0.13819519
305	17	GO Process	response to antibiotic	0.0417	PPIF|GOT2|GGH|FABP1|AQP1|AOC3|HNRNPD|OPA1|RBP4|GSN|MAOB|FECH|PRKCD|ABAT|RHOA|GPX1|AOC1	0.0053	GO.0046677	0.13798639
11	3	GO Process	fatty acid transmembrane transport	0.0418	CPT1A|ABCD3|CPT2	0.0053	GO.1902001	0.13788237
11	3	GO Process	hepatocyte apoptotic process	0.0418	GSN|KRT18|KRT8	0.0053	GO.0097284	0.13788237
11	3	GO Process	negative regulation of platelet aggregation	0.0418	CD9|PRKCD|ABAT	0.0053	GO.0090331	0.13788237
11	3	GO Process	cyclooxygenase pathway	0.0418	CBR1|PTGES2|AKR1C3	0.0053	GO.0019371	0.13788237
11	3	GO Process	ATP transport	0.0418	SLC25A4|SLC25A5|SLC25A6	0.0053	GO.0015867	0.13788237
11	3	GO Process	nucleoside monophosphate catabolic process	0.0418	DNPH1|HPRT1|AMPD3	0.0053	GO.0009125	0.13788237
11	3	GO Process	aspartate metabolic process	0.0418	GOT2|GOT1|ASS1	0.0053	GO.0006531	0.13788237
11	3	GO Process	disaccharide metabolic process	0.0418	GAA|FBP1|MGAM	0.0053	GO.0005984	0.13788237
159	11	GO Process	regulation of cell size	0.0421	RAB21|RAB11A|SLC9A3R1|ADD1|DPYSL2|AQP1|LAMTOR4|MSN|ILK|GDI1|RHOA	0.0054	GO.0008361	0.13757179
332	18	GO Process	regulation of small molecule metabolic process	0.0424	PPIF|ACADL|ENO1|APOA1|CPT1A|BHMT|ERLIN2|FABP1|GPD1|SHMT2|UGT1A9|VCP|AKR1C3|BPGM|RHOA|FBP1|EPHX2|RAN	0.0055	GO.0062012	0.13726341

5159	163	GO Component	plasma membrane	0.0429	RALA|SYPL1|GNA11|HSD17B10|CA12|VCL|NIPSNAP1|MYH9|ATP6V1D|F9|EHD4|SLC25A3|ATP6V1B1|ENO1|APOA1|GOT2|CAPNS1|VIL1|HSPB1|ATP6V1E1|LGALS3|RAB21|CDH1|RAB11A|DNAJA3|SLC4A1|SLC9A3R1|PACSIN2|NAPA|EPCAM|LRP2|GRB14|RAB17|ATP6V0A1|ADD1|SCARB2|RAB7A|AMBP|ENPEP|VDAC1|SLC27A2|RHCG|IQGAP1|BCAM|ARL8A|ATP6V1A|ATP6V1B2|ERLIN2|LAMTOR1|SLC25A4|GPD1L|CA2|ATP6V0D2|FTCD|TBC1D24|CAPN2|RNPEP|SCIN|AMN|CDH16|ANPEP|COL6A2|SLC23A1|TMED10|DCXR|HINT1|GNB2|GAA|ADH1B|ATP6V0A4|DPYSL2|CYP4A11|AQP1|RAB6A|AOC3|DAB2|TOLLIP|TLN1|SLC7A8|RHOT2|EHD3|AP2A2|SLC5A2|ATP6V0C|SERPINA5|LAMP1|BSG|PARVA|EPB41L3|VAPA|PFKM|PLS3|GM2A|UBE2D3|ATP6V1H|MSN|MYH10|DPP4|KIF21A|KRT19|COL6A1|CYB5R1|ATP1B1|FLNA|MYO6|XPNPEP2|SLC25A5|AKR1A1|GSN|RAB14|ATP6V1G1|CLIC4|HSPG2|CLIC1|KCTD12|CUBN|SLC3A2|CKAP4|GLDC|GPNMB|CD9|SACM1L|PRKAR1A|DPEP1|CNP|FHL1|ACTN1|PRKCD|PSAP|KTN1|CD59|UMOD|MGST1|ILK|GGT5|ATP5A1|SEPT7|GGT1|PDIA6|UBA52|SLC4A4|PDZK1|RHOA|GPI|LAMP2|AOC1|TOM1|TGFBI|CFB|MME|FLNB|ACSL1|WDR1|CAPN1|ATP1A1|MGAM|CD63|KRT8|TWF1|SLC43A2|PHB|PDLIM5|PLCG2	0.0127	GO.0005886	0.13675427
88	7	GO Component	collagen trimer	0.0429	DCN|COL14A1|COL6A2|COL18A1|COL4A2|COL6A1|COL4A1	0.0126	GO.0005581	0.13675427
172	12	KEGG Pathways	Tuberculosis	0.0133	CTSD|ATP6V0A1|RAB7A|ATP6V0D2|HSPA9|CYCS|ATP6V0A4|ATP6V0C|LAMP1|ATP6V1H|RHOA|LAMP2	0.0035	hsa05152	0.18761484
220	13	GO Function	organic anion transmembrane transporter activity	0.0438	SLC4A1|SLC27A2|SLC25A4|SLC23A1|SLC7A8|BSG|SLC25A5|SLC3A2|SLC25A6|SLC25A12|SLC4A4|SLC25A10|SLC43A2	0.0089	GO.0008514	0.13585259
160	11	GO Process	regulation of actin polymerization or depolymerization	0.0438	PFN2|VIL1|CAPG|ADD1|SCIN|CFL2|GSN|PRKCD|RHOA|WDR1|TWF1	0.0056	GO.0008064	0.13585259
82	7	GO Function	antiporter activity	0.0439	SLC4A1|SLC25A4|SLC7A8|SLC25A5|SLC3A2|SLC25A6|SLC25A10	0.009	GO.0015297	0.13575355
68	6	GO Component	platelet alpha granule lumen	0.0443	ACTN4|F13A1|APOOL|ACTN1|ALDOA|SERPINA1	0.0131	GO.0031093	0.13535963
39	5	GO Process	establishment or maintenance of epithelial cell apical/basal polarity	0.0443	SLC9A3R1|MSN|ILK|RHOA|WDR1	0.0057	GO.0045197	0.13535963
5	2	GO Component	spermatoproteasome complex	0.0444	PSMB9|PSMB8	0.0133	GO.1990111	0.1352617
5	2	GO Component	Ragulator complex	0.0444	LAMTOR1|LAMTOR4	0.0133	GO.0071986	0.1352617
75	7	GO Process	interaction with symbiont	0.0446	LTF|APCS|AQP1|VAPA|PC|GPX1|PSMC3	0.0058	GO.0051702	0.13506651
283	16	GO Process	multicellular organismal homeostasis	0.0453	CTSH|ACADL|NAPSA|RAB11A|ADD1|RAB7A|PRDX5|TPP1|CYP4A11|AQP1|PBLD|RBP4|CUBN|PRKAR1A|AMPD3|GPX1	0.0059	GO.0048871	0.13439018
362	19	GO Process	translation	0.0463	RPS16|NARS|APEH|RPS9|TSFM|PPA2|WARS|MRPL37|MRPL24|MRPL21|RPL5|VARS|GGT5|GGT1|UBA52|LARS2|MRPS22|GFM1|RPL18	0.006	GO.0006412	0.1334419
209	13	GO Process	receptor-mediated endocytosis	0.0464	APOA1|CANX|TINAG|LRP2|SCARB2|AMBP|TINAGL1|AMN|DAB2|AP2A2|HSPG2|CUBN|CD9	0.0061	GO.0006898	0.1333482

4	2	GO Function	leukotriene C4 gamma-glutamyl transferase activity	0.0469	GGT5|GGT1	0.0096	GO.0103068	0.13288272
4	2	GO Function	hypoglycin A gamma-glutamyl transpeptidase activity	0.0469	GGT5|GGT1	0.0096	GO.0102953	0.13288272
4	2	GO Function	glycine N-acyltransferase activity	0.0469	GLYATL1|GLYAT	0.0096	GO.0047961	0.13288272
4	2	GO Function	L-malate dehydrogenase activity	0.0469	MDH2|PHGDH	0.0096	GO.0030060	0.13288272
4	2	GO Function	transferase activity, transferring aldehyde or ketonic groups	0.0469	TALDO1|TKT	0.0096	GO.0016744	0.13288272
4	2	GO Function	inorganic diphosphatase activity	0.0469	PPA2|LHPP	0.0096	GO.0004427	0.13288272
4	2	GO Function	carnitine O-palmitoyltransferase activity	0.0469	CPT1A|CPT2	0.0096	GO.0004095	0.13288272
198	12	GO Function	mRNA binding	0.0469	HNRNPL|GRSF1|ACO1|HNRNPD|SHMT1|MYH10|RPL5|HNRNPR|AUH|ELAVL1|CRYZ|CIRBP	0.0098	GO.0003729	0.13288272
57	6	GO Process	tetrapyrrole metabolic process	0.0469	AMBP|AMN|IBA57|CUBN|FECH|MMAB	0.0061	GO.0033013	0.13288272
57	6	GO Process	actin cytoskeleton reorganization	0.0469	RALA|MYH9|PARVA|FLNA|GSN|RHOA	0.0061	GO.0031532	0.13288272
76	7	GO Process	cell-cell junction assembly	0.0471	VCL|ACTN4|TLN1|EPB41L3|CD9|RHOA|WDR1	0.0062	GO.0007043	0.13269791
36	8	KEGG Pathways	Tyrosine metabolism	8.8E-05	GOT2|HGD|HPD|ADH1B|AOC3|GOT1|MAOB|DDC	1.66E-05	hsa00350	0.40540393
40	5	GO Process	plasma lipoprotein particle clearance	0.048	APOA1|AMN|AP2A2|CUBN|NPC2	0.0063	GO.0034381	0.13187588
106	8	GO Function	unfolded protein binding	0.049	CDC37|CANX|APCS|DNAJA3|AFG3L2|HSPA9|NAP1L4|PPIA	0.0105	GO.0051082	0.13098039
200	12	GO Function	cell adhesion molecule binding	0.0492	ACTN4|LAMA5|CDH1|EPCAM|TLN1|MSN|HSPG2|GPNMB|CD9|ACTN1|ILK|TGFBI	0.0105	GO.0050839	0.13080349
164	11	GO Process	cellular response to oxygen levels	0.0492	ATP6V1A|ACAA2|FABP1|AQP1|PCK1|OPA1|ATP6V1G1|UBA52|HIGD1A|FAM162A|PHB2	0.0067	GO.0071453	0.13080349
12	3	GO Process	actin crosslink formation	0.0492	PLS3|FLNA|ACTN1	0.0065	GO.0051764	0.13080349
3	2	GO Process	guanine metabolic process	0.0492	GDA|HPRT1	0.0065	GO.0046098	0.13080349
365	19	GO Process	post-translational protein modification	0.0492	PSMA4|PSMD7|APOA1|PSMD11|RAB11A|RAB2A|PSMD3|PSMA5|PSMB4|RAB1B|RAB11B|PSMB9|PSMB8|CKAP4|KTN1|PDIA6|SERPINA1|VWA1|PSMC3	0.0065	GO.0043687	0.13080349

3	2	GO Process	inositol trisphosphate biosynthetic process	0.0492	SCP2|PLCG2	0.0065	GO.0032959	0.13080349
25	4	GO Process	intracellular lipid transport	0.0492	CPT1A|ABCD3|CPT2|NPC2	0.0066	GO.0032365	0.13080349
3	2	GO Process	thymine metabolic process	0.0492	DPYS|ALDH6A1	0.0065	GO.0019859	0.13080349
3	2	GO Process	carnitine metabolic process, CoA-linked	0.0492	ACADL|ACADM	0.0065	GO.0019254	0.13080349
25	4	GO Process	positive regulation of protein processing	0.0492	MYH9|ENO1|GSN|PHB	0.0066	GO.0010954	0.13080349
3	2	GO Process	glyoxylate catabolic process	0.0492	AGXT2|HOGA1	0.0065	GO.0009436	0.13080349
12	3	GO Process	methionine biosynthetic process	0.0492	MTHFD1|BHMT2|BHMT	0.0065	GO.0009086	0.13080349
3	2	GO Process	response to lipid hydroperoxide	0.0492	GPX3|MGST1	0.0065	GO.0006982	0.13080349
3	2	GO Process	sesquiterpenoid metabolic process	0.0492	ALDH3A2|AKR1C3	0.0065	GO.0006714	0.13080349
58	6	GO Process	protein targeting to mitochondrion	0.0492	FIS1|PMPCB|AGK|SLC25A6|TOMM40|NDUFA13	0.0066	GO.0006626	0.13080349
3	2	GO Process	aspartate biosynthetic process	0.0492	GOT2|GOT1	0.0065	GO.0006532	0.13080349
3	2	GO Process	thymine catabolic process	0.0492	DPYS|ALDH6A1	0.0065	GO.0006210	0.13080349
3	2	GO Process	hexitol metabolic process	0.0492	SORD|GALK1	0.0065	GO.0006059	0.13080349
3	2	GO Process	sucrose metabolic process	0.0492	GAA|FBP1	0.0065	GO.0005985	0.13080349
3	2	GO Process	maltose metabolic process	0.0492	GAA|MGAM	0.0065	GO.0000023	0.13080349
48	22	KEGG Pathways	Valine, leucine and isoleucine degradation	1.8E-16	HSD17B10|AGXT2|EHHADH|ACADS|ALDH2|BCKDHA|ACAA2|BCAT2|ACAA1|ALDH3A2|ALDH9A1|HIBCH|ECHS1|ACADSB|ACADM|HMGCL|AUH|ALDH1B1|ABAT|ALDH7A1|ALDH6A1|HADH	2.94E-18	hsa00280	1.57375489
48	12	KEGG Pathways	Vibrio cholerae infection	4.1E-07	ATP6V1D|ATP6V1B1|ATP6V1E1|ATP6V0A1|ATP6V1A|ATP6V1B2|ATP6V0D2|ATP6V0A4|ATP6V0C|ATP6V1H|ATP6V1G1|PLCG2	4.25E-08	hsa05110	0.6391474

RO

# background genes	# genes	category	description	FDR value	genes	p-value	term name	transferred FDR value

<table>
<tr><td>1779</td><td>280</td><td>GO Process</td><td>small molecule metabolic process</td><td>4E-122</td><td>DCN|NDUFB4|OXCT1|UQCRC1|NANS|MAPK1|ACO2|GSTZ1|MTHFD1|PCK2|AHCY|IDH3G|CRYM|DECR1|OGDH|PNPO|SLC25A11|PDHX|LTA4H|GAPDH|DNPH1|AGXT2|EHHADH|ATP6V1B1|SPR|ENO1|AKR7A2|TTR|GDA|ACADS|PEPD|GOT2|PMPCB|NDUFA2|NDUFA10|HRSP12|BHMT2|CARS2|GGH|KHK|ALDH2|ATP5B|MECR|LRP2|ATP6V0A1|NNT|SDHA|ACAD11|VCAN|HIBADH|PRDX5|ALDH8A1|CPT1A|REXO2|NDUFA9|LUM|SORD|SLC27A2|UQCRC2|PFKL|BCKDHA|ABHD10|ATP6V1A|ALDH1L1|AMT|BHMT|MUT|ATP6V1B2|ERLIN2|DPYS|NDUFB9|HNMT|DLAT|OXSM|ACAD8|QDPR|GPD1L|HGD|DDAH1|HPD|ATP5O|FTCD|ATP5J2|ADH5|BDH2|ALDH1A1|CRYL1|HPRT1|AMN|NDUFB8|CKB|IDH3A|GLYATL1|ATP5L|GPD1|PRODH2|ATP5H|ECI1|SLC23A1|DCXR|HINT1|UGT2B7|GAA|ATP5I|ADH1B|GLB1|ASL|PDHB|CYCS|ATP6V0A4|GPD2|ACO1|UQCRH|ISCU|CYP4A11|AQP1|ACAD9|GBAS|GRHPR|MSRA|CRAT|ACSS1|CYC1|PIPOX|COX5A|CES2|BCKDHB|SHMT1|UGDH|PCK1|SLC7A8|UGT2B17|NDUFV1|BCAT2|HADHB|FBN1|DHRS4|MDH2|BGN|ACSM2B|P4HB|NDUFA12|IDH2|UQCR10|ACAA1|DLST|GSTA1|PKLR|CYB5A|CS|SLC25A15|PPA2|PTGES2|PFKM|ALDH3A2|GPX4|UGT1A9|STOML2|ATP5C1|ITIH2|MPC1|CYB5R3|MT-CO1|PNP|OPA1|MT-CO2|DARS2|FH|MARC2|IARS2|ATP1B1|MGST3|ECHS1|LHPP|HMGCS2|PHGDH|GSTO1|ATP5F1|AHCYL1|ABCD3|GOT1|HOGA1|ALDH18A1|RBP4|SCP2|ECHDC2|SARDH|CMPK1|AKR1A1|ASS1|OGDHL|ALDOB|ALDH4A1|VARS|AUH|AGMAT|PCCA|PSAT1|ALDH1B1|CUBN|SUCLA2|ESD|ACOT9|PDHA1|GLRX|BPHL|AKR1C3|HADHA|IDH3B|DUT|PFKP|GLDC|AK3|AMACR|BDH1|DPEP1|NME2|CNP|PC|SUOX|NIT2|DAK|AK4|QPRT|AMPD3|ABAT|GATM|MPST|HAGH|GGT5|GSTP1|ATP5A1|GGT1|ACY1|FAH|SMS|PTGR1|TST|ALDH7A1|PCYOX1|SLC25A12|IDH1|RDH13|TKT|NDUFS1|NAPRT|GK|ACSF2|HK1|DDC|CKMT2|CKMT1A|GPX1|FBP1|LARS2|IVD|MME|PCCB|ABHD14B|ACSL1|COX7C|ETFDH|EPHX2|ECHDC1|ACADVL|ME3|SLC25A10|PAH|ALDH6A1|NPC2|ETFA|COX4I1|ACSM2A|GALK1|SARS2|EPHX1|FMO1|PLCG2|HAO2|GLYAT</td><td>6.8E-126</td><td>GO.0044281</td><td>12.1452225</td></tr>
</table>

<table>
<tr><td>16271</td><td>565</td><td>GO Component</td><td>cell</td><td>1.1E-28</td><td>RALA|DCN|NDUFB4|OXCT1|UQCRC1|NANS|VCL|MAPK1|LGALS1|RTCB|MYH9|ACO2|NID2|GSTZ1|MTHFD1|PCK2|AHCY|IDH3G|PGRMC1|F9|CRYM|CTSH|DECR1|OGDH|PLGRKT|PPIF|PNPO|PFN1|SLC25A11|C1QBP|NAGLU|VTN|CTSC|PDHX|SLC25A3|MLEC|LTA4H|GAPDH|RAB35|DNPH1|AGXT2|SKP1|LTF|EHHADH|REEP6|ATP6V1B1|SPR|ENO1|AKR7A2|CTSD|TTR|GDA|ACADS|PEPD|GOT2|DSTN|TRAP1|CANX|TUBA4A|VIL1|HSPB1|PMPCB|MTX2|NDUFA2|ACTN4|NDUFA10|NAPSA|LGALS3|GRSF1|HRSP12|DMGDH|BHMT2|GSTM3|CARS2|LAMC1|MRPL15|GGH|SQRDL|ADAM10|KHK|LRPPRC|LYZ|PEBP1|PSMD11|ALDH2|ERP29|ATP5B|COTL1|CHCHD3|SLC9A3R1|PRDX1|BBOX1|EHD2|NAPA|CD81|MECR|LRP2|PSMD3|ATP6V0A1|NNT|ADD1|F13A1|SDHA|ACAD11|VCAN|AMBP|ENPEP|VDAC1|HIBADH|CALB1|PRDX5|ALDH8A1|CPT1A|EIF4H|REXO2|MTFP1|NDUFA9|RBP5|LUM|SORD|SLC27A2|RHCG|UQCRC2|AFG3L2|PFKL|BCKDHA|SOD1|BCAM|ECSIT|TIMM44|TINAGL1|NAT8|ABHD10|ATP6V1A|ALDH1L1|AMT|BHMT|IQGAP2|HIGD2A|MUT|ATP6V1B2|ERLIN2|LACTB2|DPYS|NDUFB9|MRPL49|HNMT|CCT5|DLAT|OXSM|ACAD8|QDPR|SLC25A4|GPD1L|HGD|DDAH1|UCHL1|ATP6V0D2|MRPL17|HPD|ATP5O|ACTC1|ATP6V0D1|FTCD|ATP5J2|COX7A1|CAPN2|RNPEP|FABP1|ADH5|BDH2|CMBL|SCIN|HSPA9|COL1A2|ALDH1A1|COL14A1|CRYL1|PRDX3|HPRT1|AMN|NDUFB8|CKB|CCT2|TPP1|IDH3A|CDH16|HSP90B1|PPIB|LDHD|ANPEP|GLYATL1|PDIA3|ATP5L|CA4|TUBA1C|GPD1|PRODH2|ATP5H|ECI1|SLC23A1|DCXR|HINT1|UGT2B7|LETM1|MRPL39|GAA|ATP5I|YWHAG|MRPL13|ADH1B|TMEM126A|GLB1|ASL|PDHB|CYCS|ATP6V0A4|GPD2|ACO1|DPYSL2|C19orf70|UQCRH|PRKACA|RAB1B|ISCU|CSRP2|CYP4A11|AQP1|AOC3|ACAD9|GBAS|DAB2|GRHPR|TSFM|MSRA|CRAT|TLN1|ACSS1|CYC1|PIPOX|COX5A|CES2|BCKDHB|SHMT1|UGDH|PCK1|CHDH|TMEM11|SLC7A8|UGT2B17|CALR|RHOT2|TUFM|NDUFV1|BCAT2|PLEC|HSPA5|HADHB|FBN1|CNDP2|DHRS4|MDH2|EHD3|BGN|ACSM2B|AP2A2|P4HB|SLC5A2|NDUFA12|C11orf54|IDH2|UQCR10|SERPINA5|RAB11B|ACAA1|MRPL12|PARVA|HSP90AA1|TMLHE|DLST|GSTA1|PLS1|PKLR|TUBB4B|CYB5A|RPS2|PRDX6|CS|SLC25A15|GNAI1|EPB41L3|PPA2|SLC25A40|TPM4|PTGES2|SAMM50|CTSB|PFKM|ALDH3A2|ANXA2|GPX4|ANXA6|UGT1A9|ALDH9A1|AGK|COL18A1|VAT1|RAC1|STOML2|PIGR|ATP5C1|GM2A|ITIH2|PBLD|MSN|MYH10|COL4A2|DPP3|DPP4|LONP1|MRPL37|MPC1|CYB5R3|MT-CO1|PNP|MRPL21|OPA1|COA3|MT-CO2|DARS2|KRT19|TGM2|AKR7A3|FH|IBA57|MARC2|IARS2|C4BPA|CSRP1|ATP1B1|MGST3|ECHS1|S100A10|LHPP|RTN4IP1|HMGCS2|PHGDH|GSTO1|ATP5F1|AHCYL1|USMG5|FLNA|SFXN2|MYO6|ABCD3|GOT1|HOGA1|GNAS|XPNPEP2|ALDH18A1|RBP4|SCP2|ECHDC2|SLC25A5|PMPCA|SARDH|CMPK1|AKR1A1|ASS1|VDAC2|YWHAB|APOOL|RAB14|CISD1|OGDHL|CLIC4|HSPG2|NIPSNAP3A|ALDOB|ALDH4A1|ASPN|VARS|AUH|CLIC1|COL4A1|AGMAT|PCCA|FLOT1|PSAT1|KCTD12|ALDH1B1|CUBN|SLC3A2|RSU1|ACTR2|MAOB|SUCLA2|ESD|APOO|ACOT9|POSTN|TXNDC5|PDHA1|MT1M|GLRX|MPV17|HBD|BPHL|NQO2|AKR1C3|HADHA|IDH3B|DUT|PITRM1|PFKP|GLDC|SLC25A6|AK3|AMACR|CD9|FECH|KRT18|SACM1L|DNM2|BDH1|DPEP1|NME2|PRCP|CNP|CORO1B|JUP|PC|SUOX|NIT2|FHL1|ACTN1|DAK|FLOT2|AK4|MVP|QPRT|SNTB1|UMOD|PDP1|MGST1|TSPO|MYH11|AMPD3|ABAT|GATM|ILK|MPST|HAGH|GGT5|GSTP1|ATP5A1|SEPT7|FKBP1A|CDC42|GGT1|TXNRD2|RDX|ACY1|IFI30|FAH|PDIA6|SMS|PTGR1|TST|TF|RMDN1|ALAD|SSB|ALDH7A1|IMMT|PCYOX1|[illegible]</td><td>3.03E-30</td><td>GO.0005623</td><td>2.79746941</td></tr>
</table>

14286	552	GO Component	intracellular	6.3E-44	RALA|DCN|NDUFB4|OXCT1|UQCRC1|NANS|VCL|MAPK1|LGALS1|RTCB|MYH9|ACO2|GSTZ1|MTHFD1|PCK2|AHCY|IDH3G|PGRMC1|F9|CRYM|CTSH|DECR1|OGDH|PPIF|PNPO|PFN1|SLC25A11|C1QBP|NAGLU|VTN|CTSC|PDHX|SLC25A3|MLEC|LTA4H|GAPDH|RAB35|DNPH1|AGXT2|SKP1|LTF|EHHADH|REEP6|ATP6V1B1|SPR|ENO1|AKR7A2|CTSD|TTR|GDA|ACADS|PEPD|GOT2|DSTN|TRAP1|CANX|TUBA4A|VIL1|HSPB1|PMPCB|MTX2|NDUFA2|ACTN4|NDUFA10|NAPSA|LGALS3|GRSF1|HRSP12|DMGDH|BHMT2|GSTM3|CARS2|LAMC1|MRPL15|GGH|SQRDL|ADAM10|KHK|LRPPRC|LYZ|PEBP1|PSMD11|ALDH2|ERP29|ATP5B|COTL1|CHCHD3|SLC9A3R1|PRDX1|BBOX1|EHD2|NAPA|MECR|LRP2|PSMD3|ATP6V0A1|NNT|ADD1|F13A1|SDHA|ACAD11|VCAN|AMBP|ENPEP|VDAC1|HIBADH|CALB1|PRDX5|ALDH8A1|CPT1A|EIF4H|REXO2|MTFP1|NDUFA9|RBP5|LUM|SORD|SLC27A2|RHCG|UQCRC2|AFG3L2|PFKL|BCKDHA|SOD1|ECSIT|TIMM44|TINAGL1|NAT8|ABHD10|ATP6V1A|ALDH1L1|AMT|BHMT|IQGAP2|HIGD2A|MUT|ATP6V1B2|ERLIN2|LACTB2|DPYS|NDUFB9|MRPL49|HNMT|CCT5|DLAT|OXSM|ACAD8|QDPR|SLC25A4|GPD1L|HGD|DDAH1|UCHL1|ATP6V0D2|MRPL17|HPD|ATP5O|ACTC1|ATP6V0D1|FTCD|ATP5J2|COX7A1|CAPN2|RNPEP|FABP1|ADH5|BDH2|CMBL|SCIN|HSPA9|COL1A2|ALDH1A1|COL14A1|CRYL1|PRDX3|HPRT1|AMN|NDUFB8|CKB|CCT2|TPP1|IDH3A|HSP90B1|PPIB|LDHD|ANPEP|GLYATL1|PDIA3|ATP5L|CA4|TUBA1C|GPD1|PRODH2|ATP5H|ECI1|SLC23A1|DCXR|HINT1|UGT2B7|LETM1|MRPL39|GAA|ATP5I|YWHAG|MRPL13|ADH1B|TMEM126A|GLB1|ASL|PDHB|CYCS|ATP6V0A4|GPD2|ACO1|DPYSL2|C19orf70|UQCRH|PRKACA|RAB1B|ISCU|CSRP2|CYP4A11|AQP1|AOC3|ACAD9|GBAS|DAB2|GRHPR|TSFM|MSRA|CRAT|TLN1|ACSS1|CYC1|PIPOX|COX5A|CES2|BCKDHB|SHMT1|UGDH|PCK1|CHDH|TMEM11|SLC7A8|UGT2B17|CALR|RHOT2|TUFM|NDUFV1|BCAT2|PLEC|HSPA5|HADHB|FBN1|CNDP2|DHRS4|MDH2|EHD3|BGN|ACSM2B|AP2A2|P4HB|NDUFA12|C11orf54|IDH2|UQCR10|SERPINA5|RAB11B|ACAA1|MRPL12|PARVA|HSP90AA1|TMLHE|DLST|GSTA1|PLS1|PKLR|TUBB4B|CYB5A|RPS2|PRDX6|CS|SLC25A15|GNAI1|EPB41L3|PPA2|SLC25A40|TPM4|PTGES2|SAMM50|CTSB|PFKM|ALDH3A2|ANXA2|GPX4|ANXA6|UGT1A9|ALDH9A1|AGK|COL18A1|VAT1|RAC1|STOML2|PIGR|ATP5C1|GM2A|ITIH2|PBLD|MSN|MYH10|COL4A2|DPP3|DPP4|LONP1|MRPL37|MPC1|CYB5R3|MT-CO1|PNP|MRPL21|OPA1|COA3|MT-CO2|DARS2|KRT19|TGM2|AKR7A3|FH|IBA57|MARC2|IARS2|CSRP1|ATP1B1|MGST3|ECHS1|S100A10|LHPP|RTN4IP1|HMGCS2|PHGDH|GSTO1|ATP5F1|AHCYL1|USMG5|FLNA|SFXN2|MYO6|ABCD3|GOT1|HOGA1|GNAS|ALDH18A1|RBP4|SCP2|ECHDC2|SLC25A5|PMPCA|SARDH|CMPK1|AKR1A1|ASS1|VDAC2|YWHAB|APOOL|RAB14|CISD1|OGDHL|CLIC4|HSPG2|NIPSNAP3A|ALDOB|ALDH4A1|ASPN|VARS|AUH|CLIC1|COL4A1|AGMAT|PCCA|FLOT1|PSAT1|ALDH1B1|CUBN|SLC3A2|RSU1|ACTR2|MAOB|SUCLA2|ESD|APOO|ACOT9|POSTN|TXNDC5|PDHA1|MT1M|GLRX|MPV17|HBD|BPHL|NQO2|AKR1C3|HADHA|IDH3B|DUT|PITRM1|PFKP|GLDC|SLC25A6|AK3|AMACR|CD9|FECH|KRT18|SACM1L|DNM2|BDH1|DPEP1|NME2|PRCP|CNP|CORO1B|JUP|PC|SUOX|NIT2|FHL1|ACTN1|DAK|FLOT2|AK4|MVP|QPRT|SNTB1|UMOD|PDP1|MGST1|TSPO|MYH11|AMPD3|ABAT|GATM|ILK|MPST|HAGH|GSTP1|ATP5A1|SEPT7|FKBP1A|CDC42|TXNRD2|RDX|ACY1|IFI30|FAH|PDIA6|SMS|PTGR1|TST|TF|RMDN1|ALAD|SSB|ALDH7A1|IMMT|PCYOX1|SLC25A12|SCRN1|ARPC1B|IDH1|RDH1[illegible]	9.73E-46	GO.0005622	4.32034257

1250	203	KEGG Pathways	Metabolic pathways	1.5E-85	NDUFB4|UQCRC1|NANS|ACO2|GSTZ1|MTHFD1|PCK2|AHCY|IDH3G|OGDH|PNPO|NAGLU|PDHX|LTA4H|GAPDH|AGXT2|EHHADH|ATP6V1B1|SPR|ENO1|GDA|ACADS|GOT2|NDUFA2|NDUFA10|LAMA5|DMGDH|BHMT2|KHK|ALDH2|ATP5B|MECR|ATP6V0A1|NNT|SDHA|HIBADH|NDUFA9|SORD|UQCRC2|PFKL|BCKDHA|ATP6V1A|AMT|BHMT|MUT|ATP6V1B2|DPYS|NDUFB9|DLAT|OXSM|ACAD8|QDPR|HGD|ATP6V0D2|HPD|ATP5O|ATP6V0D1|FTCD|ATP5J2|ADH5|BDH2|CMBL|ALDH1A1|CRYL1|HPRT1|NDUFB8|CKB|IDH3A|ANPEP|ATP5L|PRODH2|ATP5H|DCXR|UGT2B7|GAA|ATP5I|ADH1B|GLB1|ASL|PDHB|CYCS|ATP6V0A4|ACO1|UQCRH|CYP4A11|AOC3|GRHPR|ACSS1|CYC1|PIPOX|COX5A|BCKDHB|SHMT1|UGDH|PCK1|CHDH|UGT2B17|NDUFV1|BCAT2|HADHB|CNDP2|DHRS4|MDH2|ACSM2B|NDUFA12|IDH2|UQCR10|ACAA1|DLST|PKLR|PRDX6|CS|PTGES2|PFKM|ALDH3A2|UGT1A9|ALDH9A1|AGK|ATP5C1|MT-CO1|PNP|MT-CO2|FH|ECHS1|HMGCS2|PHGDH|ATP5F1|AHCYL1|GOT1|HOGA1|ALDH18A1|SCP2|SARDH|AKR1A1|ASS1|OGDHL|ALDOB|ALDH4A1|AUH|AGMAT|PCCA|PSAT1|ALDH1B1|MAOB|SUCLA2|PDHA1|AKR1C3|HADHA|IDH3B|DUT|PFKP|GLDC|AMACR|FECH|SACM1L|BDH1|NME2|PC|SUOX|DAK|AK4|QPRT|AMPD3|ABAT|GATM|MPST|GGT5|ATP5A1|GGT1|ACY1|FAH|SMS|TST|ALAD|ALDH7A1|IDH1|TKT|NDUFS1|NAPRT|GK|HK1|DDC|CKMT2|CKMT1A|FBP1|GBE1|AOC1|IVD|PCCB|ACSL1|NDUFA13|COX7C|EPHX2|ACADVL|ME3|MGAM|PAH|ALDH6A1|COX4I1|ACSM2A|GALK1|PLCG2|HAO2	5.19E-88	hsa01100	8.48326827
854	174	GO Process	carboxylic acid metabolic process	2.8E-84	DCN|ACO2|GSTZ1|MTHFD1|AHCY|IDH3G|CRYM|DECR1|OGDH|PDHX|LTA4H|GAPDH|AGXT2|EHHADH|ENO1|ACADS|PEPD|GOT2|HRSP12|BHMT2|CARS2|GGH|MECR|NNT|SDHA|ACAD11|VCAN|HIBADH|ALDH8A1|CPT1A|SORD|SLC27A2|PFKL|BCKDHA|ABHD10|ALDH1L1|AMT|BHMT|MUT|DPYS|HNMT|DLAT|OXSM|ACAD8|QDPR|HGD|DDAH1|HPD|FTCD|BDH2|CRYL1|CKB|IDH3A|GLYATL1|PRODH2|ECI1|SLC23A1|DCXR|UGT2B7|ASL|PDHB|ACO1|CYP4A11|ACAD9|GRHPR|MSRA|CRAT|ACSS1|PIPOX|CES2|BCKDHB|SHMT1|UGDH|PCK1|SLC7A8|UGT2B17|BCAT2|HADHB|MDH2|BGN|ACSM2B|P4HB|IDH2|ACAA1|DLST|GSTA1|PKLR|CYB5A|CS|PPA2|PTGES2|PFKM|ALDH3A2|GPX4|UGT1A9|ITIH2|MPC1|CYB5R3|DARS2|FH|IARS2|MGST3|ECHS1|PHGDH|GSTO1|AHCYL1|ABCD3|GOT1|HOGA1|ALDH18A1|SCP2|ECHDC2|SARDH|AKR1A1|ASS1|OGDHL|ALDOB|ALDH4A1|VARS|AUH|AGMAT|PCCA|PSAT1|SUCLA2|PDHA1|BPHL|AKR1C3|HADHA|IDH3B|PFKP|GLDC|AMACR|DPEP1|PC|NIT2|QPRT|ABAT|GATM|MPST|HAGH|GGT5|GSTP1|GGT1|ACY1|FAH|SMS|PTGR1|TST|ALDH7A1|PCYOX1|IDH1|ACSF2|HK1|DDC|CKMT2|CKMT1A|GPX1|LARS2|IVD|PCCB|ACSL1|ETFDH|EPHX2|ECHDC1|ACADVL|ME3|PAH|ALDH6A1|ETFA|ACSM2A|GALK1|SARS2|HAO2|GLYAT	1.06E-87	GO.0019752	8.35606673

959	181	GO Process	organic acid metabolic process	1.6E-83	DCN|ACO2|GSTZ1|MTHFD1|AHCY|IDH3G|CRYM|DECR1|OGDH|PDHX|LTA4H|GAPDH|AGXT2|EHHADH|ENO1|ACADS|PEPD|GOT2|HRSP12|BHMT2|CARS2|GGH|MECR|NNT|SDHA|ACAD11|VCAN|HIBADH|ALDH8A1|CPT1A|LUM|SORD|SLC27A2|PFKL|BCKDHA|ABHD10|ALDH1L1|AMT|BHMT|MUT|DPYS|HNMT|DLAT|OXSM|ACAD8|QDPR|HGD|DDAH1|HPD|FTCD|BDH2|CRYL1|CKB|IDH3A|GLYATL1|PRODH2|ECI1|SLC23A1|DCXR|UGT2B7|GLB1|ASL|PDHB|ACO1|CYP4A11|ACAD9|GRHPR|MSRA|CRAT|ACSS1|PIPOX|CES2|BCKDHB|SHMT1|UGDH|PCK1|SLC7A8|UGT2B17|BCAT2|HADHB|MDH2|BGN|ACSM2B|P4HB|IDH2|ACAA1|DLST|GSTA1|PKLR|CYB5A|CS|PPA2|PTGES2|PFKM|ALDH3A2|GPX4|UGT1A9|ITIH2|MPC1|CYB5R3|PNP|DARS2|FH|MARC2|IARS2|MGST3|ECHS1|PHGDH|GSTO1|AHCYL1|ABCD3|GOT1|HOGA1|ALDH18A1|SCP2|ECHDC2|SARDH|AKR1A1|ASS1|OGDHL|ALDOB|ALDH4A1|VARS|AUH|AGMAT|PCCA|PSAT1|SUCLA2|PDHA1|BPHL|AKR1C3|HADHA|IDH3B|PFKP|GLDC|AMACR|DPEP1|PC|SUOX|NIT2|QPRT|ABAT|GATM|MPST|HAGH|GGT5|GSTP1|GGT1|ACY1|FAH|SMS|PTGR1|TST|ALDH7A1|PCYOX1|IDH1|ACSF2|HK1|DDC|CKMT2|CKMT1A|GPX1|LARS2|IVD|PCCB|ABHD14B|ACSL1|ETFDH|EPHX2|ECHDC1|ACADVL|ME3|PAH|ALDH6A1|ETFA|ACSM2A|GALK1|SARS2|FMO1|HAO2|GLYAT	9.24E-87	GO.0006082	8.279588
943	179	GO Process	oxoacid metabolic process	6.1E-83	DCN|ACO2|GSTZ1|MTHFD1|AHCY|IDH3G|CRYM|DECR1|OGDH|PDHX|LTA4H|GAPDH|AGXT2|EHHADH|ENO1|ACADS|PEPD|GOT2|HRSP12|BHMT2|CARS2|GGH|MECR|NNT|SDHA|ACAD11|VCAN|HIBADH|ALDH8A1|CPT1A|LUM|SORD|SLC27A2|PFKL|BCKDHA|ABHD10|ALDH1L1|AMT|BHMT|MUT|DPYS|HNMT|DLAT|OXSM|ACAD8|QDPR|HGD|DDAH1|HPD|FTCD|BDH2|CRYL1|CKB|IDH3A|GLYATL1|PRODH2|ECI1|SLC23A1|DCXR|UGT2B7|GLB1|ASL|PDHB|ACO1|CYP4A11|ACAD9|GRHPR|MSRA|CRAT|ACSS1|PIPOX|CES2|BCKDHB|SHMT1|UGDH|PCK1|SLC7A8|UGT2B17|BCAT2|HADHB|MDH2|BGN|ACSM2B|P4HB|IDH2|ACAA1|DLST|GSTA1|PKLR|CYB5A|CS|PPA2|PTGES2|PFKM|ALDH3A2|GPX4|UGT1A9|ITIH2|MPC1|CYB5R3|DARS2|FH|MARC2|IARS2|MGST3|ECHS1|PHGDH|GSTO1|AHCYL1|ABCD3|GOT1|HOGA1|ALDH18A1|SCP2|ECHDC2|SARDH|AKR1A1|ASS1|OGDHL|ALDOB|ALDH4A1|VARS|AUH|AGMAT|PCCA|PSAT1|SUCLA2|PDHA1|BPHL|AKR1C3|HADHA|IDH3B|PFKP|GLDC|AMACR|DPEP1|PC|SUOX|NIT2|QPRT|ABAT|GATM|MPST|HAGH|GGT5|GSTP1|GGT1|ACY1|FAH|SMS|PTGR1|TST|ALDH7A1|PCYOX1|IDH1|ACSF2|HK1|DDC|CKMT2|CKMT1A|GPX1|LARS2|IVD|PCCB|ABHD14B|ACSL1|ETFDH|EPHX2|ECHDC1|ACADVL|ME3|PAH|ALDH6A1|ETFA|ACSM2A|GALK1|SARS2|HAO2|GLYAT	4.71E-86	GO.0043436	8.22139588
932	177	GO Process	oxidation-reduction process	5.3E-82	NDUFB4|UQCRC1|ACO2|GSTZ1|MTHFD1|IDH3G|CRYM|DECR1|OGDH|PNPO|GAPDH|EHHADH|SPR|ENO1|AKR7A2|ACADS|PMPCB|NDUFA2|NDUFA10|DMGDH|SQRDL|ALDH2|PRDX1|BBOX1|MECR|NNT|SDHA|ACAD11|HIBADH|PRDX5|ALDH8A1|CPT1A|NDUFA9|SORD|SLC27A2|UQCRC2|PFKL|BCKDHA|SOD1|ECSIT|ALDH1L1|HIGD2A|NDUFB9|DLAT|ACAD8|QDPR|GPD1L|HGD|HPD|COX7A1|ADH5|BDH2|ALDH1A1|CRYL1|PRDX3|NDUFB8|IDH3A|LDHD|PDIA3|GPD1|PRODH2|ECI1|DCXR|GAA|ADH1B|PDHB|CYCS|GPD2|ACO1|UQCRH|CYP4A11|AOC3|ACAD9|GRHPR|MSRA|CRAT|ACSS1|CYC1|PIPOX|COX5A|BCKDHB|UGDH|CHDH|NDUFV1|HADHB|DHRS4|MDH2|P4HB|NDUFA12|IDH2|UQCR10|ACAA1|TMLHE|DLST|GSTA1|PKLR|CYB5A|PRDX6|CS|PTGES2|PFKM|ALDH3A2|GPX4|ALDH9A1|VAT1|CYB5R3|MT-CO1|MT-CO2|AKR7A3|FH|MARC2|MGST3|ECHS1|RTN4IP1|PHGDH|GSTO1|ABCD3|GNAS|ALDH18A1|SCP2|ECHDC2|SARDH|AKR1A1|OGDHL|ALDOB|ALDH4A1|AUH|ALDH1B1|MAOB|SUCLA2|PDHA1|GLRX|NQO2|AKR1C3|HADHA|IDH3B|PFKP|GLDC|AMACR|GPX3|BDH1|SUOX|MGST1|GSTP1|TXNRD2|IFI30|PDIA6|PTGR1|ALDH7A1|PCYOX1|SLC25A12|IDH1|RDH13|TKT|NDUFS1|SELENBP1|HIGD1A|CRYZ|HK1|COX20|GPX1|GBE1|AOC1|IVD|NDUFA13|COX7C|ETFDH|ECHDC1|ACADVL|ME3|SLC25A10|PAH|ALDH6A1|ETFA|COX4I1|FMO1|HAO2	5.11E-85	GO.0055114	8.12757241

622	147	GO Process	drug metabolic process	1.2E-77	DCN|NDUFB4|OXCT1|UQCRC1|ACO2|GSTZ1|MTHFD1|AHCY|IDH3G|CTSH|OGDH|PNPO|GAPDH|AGXT2|ATP6V1B1|SPR|ENO1|AKR7A2|PMPCB|NDUFA2|NDUFA10|HRSP12|BHMT2|SQRDL|ALDH2|ATP5B|PRDX1|ATP6V0A1|NNT|SDHA|VCAN|PRDX5|ALDH8A1|NDUFA9|UQCRC2|PFKL|SOD1|ATP6V1A|ALDH1L1|AMT|BHMT|MUT|ATP6V1B2|DPYS|NDUFB9|DLAT|QDPR|HGD|HPD|ATP5O|FTCD|ATP5J2|ADH5|BDH2|ALDH1A1|PRDX3|HPRT1|AMN|NDUFB8|CKB|IDH3A|ATP5L|ATP5H|ATP5I|ADH1B|PDHB|CYCS|ATP6V0A4|ACO1|UQCRH|GBAS|ACSS1|CYC1|PIPOX|COX5A|SHMT1|NDUFV1|MDH2|BGN|NDUFA12|IDH2|UQCR10|ACAA1|DLST|PKLR|PRDX6|CS|PFKM|ALDH3A2|STOML2|ATP5C1|MT-CO1|MT-CO2|FH|ATP1B1|HMGCS2|PHGDH|ATP5F1|AHCYL1|ALDH18A1|SCP2|OGDHL|ALDOB|PCCA|PSAT1|ALDH1B1|CUBN|MAOB|SUCLA2|ESD|PDHA1|AKR1C3|IDH3B|PFKP|GLDC|AK3|GPX3|BDH1|DPEP1|PC|AK4|QPRT|AMPD3|GATM|MPST|GGT5|ATP5A1|GGT1|FAH|SMS|IDH1|NDUFS1|HK1|DDC|CKMT2|CKMT1A|GPX1|PCCB|ACSL1|COX7C|EPHX2|ME3|PAH|COX4I1|GALK1|FMO1|GLYAT	1.38E-80	GO.0017144	7.6924453
5592	382	GO Function	catalytic activity	1.6E-72	RALA|SPATA20|NDUFB4|OXCT1|UQCRC1|NANS|MAPK1|RTCB|MYH9|ACO2|GSTZ1|MTHFD1|PCK2|AHCY|IDH3G|F9|CRYM|CTSH|DECR1|OGDH|ABHD11|PPIF|PNPO|NAGLU|CTSC|PDHX|LTA4H|GAPDH|RAB35|DNPH1|AGXT2|SKP1|LTF|EHHADH|ATP6V1B1|SPR|ENO1|AKR7A2|CTSD|GDA|HDHD3|ACADS|PEPD|GOT2|TUBA4A|PMPCB|NDUFA2|NDUFA10|NAPSA|HRSP12|DMGDH|BHMT2|GSTM3|CARS2|TINAG|GGH|SQRDL|ADAM10|KHK|LRPPRC|LYZ|ALDH2|ATP5B|PRDX1|BBOX1|EHD2|MECR|ATP6V0A1|NNT|F13A1|SDHA|ACAD11|ENPEP|HIBADH|PRDX5|ALDH8A1|CPT1A|EIF4H|REXO2|NDUFA9|SORD|SLC27A2|UQCRC2|AFG3L2|PFKL|BCKDHA|SOD1|ECSIT|TINAGL1|NAT8|ABHD10|ATP6V1A|ALDH1L1|AMT|BHMT|MUT|ATP6V1B2|ERLIN2|LACTB2|DPYS|NDUFB9|HNMT|DLAT|OXSM|ACAD8|QDPR|GPD1L|HGD|DDAH1|UCHL1|ATP6V0D2|HPD|ATP5O|ACTC1|ATP6V0D1|FTCD|COX7A1|CAPN2|RNPEP|ADH5|BDH2|CMBL|ALDH1A1|CRYL1|PRDX3|HPRT1|NDUFB8|CKB|TPP1|IDH3A|PPIB|LDHD|ANPEP|GLYATL1|PDIA3|CA4|TUBA1C|GPD1|PRODH2|ECI1|DCXR|HINT1|UGT2B7|GAA|ADH1B|GLB1|ASL|PDHB|CYCS|ATP6V0A4|GPD2|ACO1|DPYSL2|UQCRH|PRKACA|RAB1B|ISCU|CYP4A11|AOC3|ACAD9|GRHPR|MSRA|CRAT|ACSS1|CYC1|PIPOX|COX5A|CES2|BCKDHB|SHMT1|UGDH|PCK1|CHDH|UGT2B17|RHOT2|TUFM|NDUFV1|BCAT2|HSPA5|HADHB|CNDP2|DHRS4|MDH2|ACSM2B|P4HB|NDUFA12|C11orf54|IDH2|UQCR10|RAB11B|ACAA1|HSP90AA1|TMLHE|DLST|GSTA1|PKLR|TUBB4B|CYB5A|PRDX6|CS|GNAI1|TPSAB1|PPA2|PTGES2|CTSB|PFKM|ALDH3A2|GPX4|UGT1A9|ALDH9A1|AGK|VAT1|RAC1|ATP5C1|GM2A|PBLD|MYH10|DPP3|DPP4|LONP1|CYB5R3|MT-CO1|PNP|OPA1|MT-CO2|DARS2|TGM2|AKR7A3|FH|IBA57|MARC2|IARS2|ATP1B1|MGST3|ECHS1|LHPP|RTN4IP1|HMGCS2|PHGDH|GSTO1|MYO6|ABCD3|GOT1|HOGA1|GNAS|XPNPEP2|ALDH18A1|SCP2|ECHDC2|PMPCA|SARDH|CMPK1|AKR1A1|ASS1|COMTD1|RAB14|OGDHL|ALDOB|ALDH4A1|VARS|AUH|AGMAT|PCCA|PSAT1|ALDH1B1|SLC3A2|MAOB|SUCLA2|ESD|ACOT9|TXNDC5|PDHA1|GLRX|BPHL|NQO2|AKR1C3|HADHA|IDH3B|DUT|PITRM1|PFKP|GLDC|AK3|AMACR|FECH|GPX3|SACM1L|DNM2|BDH1|DPEP1|NME2|PRCP|CNP|PC|SUOX|NIT2|DAK|AK4|QPRT|PDP1|MGST1|MYH11|AMPD3|ABAT|GATM|ILK|MPST|HAGH|GGT5|GSTP1|ATP5A1|FKBP1A|CDC42|GGT1|TXNRD2|ACY1|IFI30|FAH|PDIA6|SMS|PTGR1|TST|ALAD|ALDH7A1|CPVL|PCYOX1|SCRN1|IDH1|RDH13|TKT|NDUFS1|SELENBP1|CRYZ|PNPT1|NAPRT|GK|ACSF2|HK1|DDC|CKMT2|CKMT1A|GPX1|FBP1|LARS2|GBE1|AOC1|IVD|MME|PCCB|GFM1|ABHD14B|GSTA2|ACSL1|NDUFA13|COX7C|ETFDH|EPHX2|RAP1GAP|ECHDC1|ACADVL|ME3|ATP1A1|TIMM50|MYL6|MGAM|PAH|METTL7A|DNM1L|ALDH6A1|ETFA|ITGB3|COX4I1|ACSM2A|GALK1|TTN|SARS2|SIRT5|EPHX1|FMO1|PLCG2|HAO2|GLYAT	1.35E-75	GO.0003824	7.17851562

11238	545	GO Component	cytoplasm	1E-86	RALA|DCN|NDUFB4|OXCT1|UQCRC1|NANS|VCL|MAPK1|LGALS1|RTCB|MYH9|ACO2|GSTZ1|MTHFD1|PCK2|AHCY|IDH3G|PGRMC1|F9|CRYM|CTSH|DECR1|OGDH|PPIF|PNPO|PFN1|SLC25A11|C1QBP|NAGLU|VTN|CTSC|PDHX|SLC25A3|MLEC|LTA4H|GAPDH|RAB35|DNPH1|AGXT2|SKP1|LTF|EHHADH|REEP6|ATP6V1B1|SPR|ENO1|AKR7A2|CTSD|TTR|GDA|ACADS|GOT2|DSTN|TRAP1|CANX|TUBA4A|VIL1|HSPB1|PMPCB|MTX2|NDUFA2|ACTN4|NDUFA10|NAPSA|LGALS3|GRSF1|HRSP12|DMGDH|BHMT2|GSTM3|CARS2|LAMC1|MRPL15|GGH|SQRDL|ADAM10|KHK|LRPPRC|LYZ|PEBP1|PSMD11|ALDH2|ERP29|ATP5B|COTL1|CHCHD3|SLC9A3R1|PRDX1|BBOX1|EHD2|NAPA|MECR|LRP2|PSMD3|ATP6V0A1|NNT|ADD1|F13A1|SDHA|ACAD11|VCAN|ENPEP|VDAC1|HIBADH|CALB1|PRDX5|ALDH8A1|CPT1A|EIF4H|REXO2|MTFP1|NDUFA9|RBP5|LUM|SORD|SLC27A2|RHCG|UQCRC2|AFG3L2|PFKL|BCKDHA|SOD1|ECSIT|TIMM44|TINAGL1|NAT8|ABHD10|ATP6V1A|ALDH1L1|AMT|BHMT|IQGAP2|HIGD2A|MUT|ATP6V1B2|ERLIN2|LACTB2|DPYS|NDUFB9|MRPL49|HNMT|CCT5|DLAT|OXSM|ACAD8|QDPR|SLC25A4|GPD1L|HGD|DDAH1|UCHL1|ATP6V0D2|MRPL17|HPD|ATP5O|ACTC1|ATP6V0D1|FTCD|ATP5J2|COX7A1|CAPN2|RNPEP|FABP1|ADH5|BDH2|CMBL|SCIN|HSPA9|COL1A2|ALDH1A1|COL14A1|CRYL1|PRDX3|HPRT1|AMN|NDUFB8|CKB|CCT2|TPP1|IDH3A|HSP90B1|PPIB|LDHD|ANPEP|GLYATL1|PDIA3|ATP5L|CA4|TUBA1C|GPD1|PRODH2|ATP5H|ECI1|SLC23A1|DCXR|HINT1|UGT2B7|LETM1|MRPL39|GAA|ATP5I|YWHAG|MRPL13|ADH1B|TMEM126A|GLB1|ASL|PDHB|CYCS|ATP6V0A4|GPD2|ACO1|DPYSL2|C19orf70|UQCRH|PRKACA|RAB1B|ISCU|CYP4A11|AQP1|AOC3|ACAD9|GBAS|DAB2|GRHPR|TSFM|MSRA|CRAT|TLN1|ACSS1|CYC1|PIPOX|COX5A|CES2|BCKDHB|SHMT1|UGDH|PCK1|CHDH|TMEM11|SLC7A8|UGT2B17|CALR|RHOT2|TUFM|NDUFV1|BCAT2|PLEC|HSPA5|HADHB|FBN1|CNDP2|DHRS4|MDH2|EHD3|BGN|ACSM2B|AP2A2|P4HB|NDUFA12|IDH2|UQCR10|SERPINA5|RAB11B|ACAA1|MRPL12|PARVA|HSP90AA1|TMLHE|DLST|GSTA1|PLS1|PKLR|TUBB4B|CYB5A|RPS2|PRDX6|CS|SLC25A15|GNAI1|EPB41L3|PPA2|SLC25A40|TPM4|PTGES2|SAMM50|CTSB|PFKM|ALDH3A2|ANXA2|GPX4|ANXA6|UGT1A9|ALDH9A1|AGK|COL18A1|VAT1|RAC1|STOML2|PIGR|ATP5C1|GM2A|ITIH2|PBLD|MSN|MYH10|COL4A2|DPP3|DPP4|LONP1|MRPL37|MPC1|CYB5R3|MT-CO1|PNP|MRPL21|OPA1|COA3|MT-CO2|DARS2|KRT19|TGM2|AKR7A3|FH|IBA57|MARC2|IARS2|MGST3|ECHS1|S100A10|LHPP|RTN4IP1|HMGCS2|PHGDH|GSTO1|ATP5F1|AHCYL1|USMG5|FLNA|SFXN2|MYO6|ABCD3|GOT1|HOGA1|GNAS|ALDH18A1|RBP4|SCP2|ECHDC2|SLC25A5|PMPCA|SARDH|CMPK1|AKR1A1|ASS1|VDAC2|YWHAB|APOOL|RAB14|CISD1|OGDHL|CLIC4|HSPG2|NIPSNAP3A|ALDOB|ALDH4A1|ASPN|VARS|AUH|CLIC1|COL4A1|AGMAT|PCCA|FLOT1|PSAT1|ALDH1B1|CUBN|SLC3A2|RSU1|ACTR2|MAOB|SUCLA2|ESD|APOO|ACOT9|POSTN|TXNDC5|PDHA1|MT1M|GLRX|MPV17|HBD|BPHL|NQO2|AKR1C3|HADHA|IDH3B|DUT|PITRM1|PFKP|GLDC|SLC25A6|AK3|AMACR|CD9|FECH|KRT18|SACM1L|DNM2|BDH1|NME2|PRCP|CNP|CORO1B|JUP|PC|SUOX|NIT2|FHL1|ACTN1|DAK|FLOT2|AK4|MVP|QPRT|SNTB1|UMOD|PDP1|MGST1|TSPO|MYH11|AMPD3|ABAT|GATM|ILK|MPST|HAGH|GSTP1|ATP5A1|SEPT7|FKBP1A|CDC42|TXNRD2|RDX|ACY1|IFI30|FAH|PDIA6|SMS|PTGR1|TST|TF|RMDN1|ALAD|SSB|ALDH7A1|IMMT|PCYOX1|SLC25A12|SCRN1|ARPC1B|IDH1|RDH13|TKT|NDUFS1|AHSG|SELENBP1|HIGD1A|CRYZ|PNPT1|NAPRT|GK|ACSF2|HK1|ACTA2|DDC|CKMT2|NDRG1|COX20[illegible]	3.16E-89	GO.0005737	8.59913998

<table>
<tr><td>1420</td><td>192</td><td>Reactome Pathways</td><td>Metabolism</td><td>1E-68</td><td>DCN|OXCT1|MTHFD1|PCK2|AHCY|CRYM|DECR1|PNPO|SLC25A11|NAGLU|LTA4H|GAPDH|DNPH1|AGXT2|EHHADH|SPR|ENO1|AKR7A2|TTR|GDA|ACADS|GOT2|HRSP12|DMGDH|BHMT2|GSTM3|SQRDL|KHK|BBOX1|MECR|LRP2|ACAD11|VCAN|HIBADH|LUM|SORD|SLC27A2|PFKL|BCKDHA|ABHD10|ALDH1L1|AMT|BHMT|MUT|DPYS|HNMT|ACAD8|QDPR|GPD1L|HGD|DDAH1|HPD|FTCD|FABP1|ADH5|BDH2|CMBL|ALDH1A1|CRYL1|HPRT1|AMN|CKB|GLYATL1|GPD1|PRODH2|ECI1|SLC23A1|DCXR|UGT2B7|GAA|ADH1B|GLB1|ASL|GPD2|CYP4A11|AOC3|GRHPR|CRAT|ACSS1|PIPOX|CES2|BCKDHB|SHMT1|UGDH|CHDH|UGT2B17|BCAT2|HADHB|CNDP2|BGN|ACSM2B|ACAA1|TMLHE|GSTA1|CYB5A|SLC25A15|PPA2|PTGES2|PFKM|GPX4|UGT1A9|ALDH9A1|AGK|GM2A|CYB5R3|PNP|AKR7A3|MARC2|MGST3|ECHS1|LHPP|HMGCS2|PHGDH|HOGA1|RBP4|SCP2|SLC25A5|SARDH|CMPK1|AKR1A1|ASS1|RAB14|HSPG2|ALDOB|ALDH4A1|AUH|AGMAT|PCCA|PSAT1|ALDH1B1|CUBN|ESD|ACOT9|GLRX|BPHL|NQO2|AKR1C3|HADHA|PFKP|GLDC|AMACR|FECH|SACM1L|BDH1|DPEP1|PC|SUOX|DAK|AK4|QPRT|MGST1|TSPO|AMPD3|GATM|MPST|GGT5|GSTP1|GGT1|ACY1|FAH|SMS|PTGR1|TST|ALAD|ALDH7A1|IDH1|TKT|NAPRT|GK|ACSF2|HK1|DDC|CKMT2|CKMT1A|GPX1|FBP1|GBE1|AOC1|IVD|PCCB|ABHD14B|ACSL1|EPHX2|ACADVL|PAH|ALDH6A1|ACSM2A|GALK1|EPHX1|FMO1|HAO2|GLYAT</td><td>1.67E-70</td><td>HSA-1430728</td><td>6.8</td></tr>
<tr><td>716</td><td>140</td><td>GO Function</td><td>oxidoreductase activity</td><td>1.7E-64</td><td>NDUFB4|UQCRC1|GSTZ1|MTHFD1|IDH3G|CRYM|DECR1|OGDH|PNPO|GAPDH|EHHADH|SPR|AKR7A2|ACADS|NDUFA2|NDUFA10|DMGDH|SQRDL|ALDH2|PRDX1|BBOX1|MECR|NNT|SDHA|ACAD11|HIBADH|PRDX5|ALDH8A1|NDUFA9|SORD|BCKDHA|SOD1|ECSIT|ALDH1L1|NDUFB9|ACAD8|QDPR|GPD1L|HGD|HPD|COX7A1|ADH5|BDH2|ALDH1A1|CRYL1|PRDX3|NDUFB8|IDH3A|LDHD|PDIA3|GPD1|PRODH2|DCXR|ADH1B|PDHB|CYCS|GPD2|UQCRH|CYP4A11|AOC3|ACAD9|GRHPR|MSRA|CYC1|PIPOX|COX5A|BCKDHB|UGDH|CHDH|NDUFV1|HADHB|DHRS4|MDH2|P4HB|NDUFA12|IDH2|UQCR10|ACAA1|TMLHE|GSTA1|CYB5A|PRDX6|PTGES2|ALDH3A2|GPX4|ALDH9A1|VAT1|CYB5R3|MT-CO1|MT-CO2|AKR7A3|MARC2|MGST3|RTN4IP1|PHGDH|GSTO1|ALDH18A1|SARDH|AKR1A1|OGDHL|ALDH4A1|ALDH1B1|MAOB|PDHA1|GLRX|NQO2|AKR1C3|HADHA|IDH3B|GLDC|GPX3|BDH1|SUOX|MGST1|GSTP1|TXNRD2|IFI30|PDIA6|PTGR1|ALDH7A1|PCYOX1|IDH1|RDH13|NDUFS1|SELENBP1|CRYZ|GPX1|AOC1|IVD|NDUFA13|COX7C|ETFDH|ACADVL|ME3|PAH|ALDH6A1|ETFA|COX4I1|FMO1|HAO2</td><td>2.8E-67</td><td>GO.0016491</td><td>6.37695511</td></tr>
</table>

12432	499	GO Component	organelle	1.4E-32	RALA|DCN|NDUFB4|OXCT1|UQCRC1|VCL|MAPK1|LGALS1|RTCB|MYH9|ACO2|GSTZ1|MTHFD1|PCK2|AHCY|IDH3G|PGRMC1|F9|CRYM|CTSH|DECR1|OGDH|PPIF|PNPO|PFN1|SLC25A11|C1QBP|NAGLU|VTN|CTSC|PDHX|SLC25A3|MLEC|LTA4H|GAPDH|RAB35|DNPH1|AGXT2|SKP1|LTF|EHHADH|REEP6|ATP6V1B1|SPR|ENO1|AKR7A2|CTSD|TTR|ACADS|PEPD|GOT2|DSTN|TRAP1|CANX|TUBA4A|VIL1|HSPB1|PMPCB|MTX2|NDUFA2|ACTN4|NDUFA10|NAPSA|LGALS3|GRSF1|HRSP12|DMGDH|GSTM3|CARS2|LAMC1|MRPL15|GGH|SQRDL|ADAM10|LRPPRC|LYZ|PSMD11|ALDH2|ERP29|ATP5B|COTL1|CHCHD3|SLC9A3R1|PRDX1|EHD2|NAPA|CD81|MECR|LRP2|PSMD3|ATP6V0A1|NNT|ADD1|F13A1|SDHA|ACAD11|VCAN|AMBP|ENPEP|VDAC1|HIBADH|CALB1|PRDX5|CPT1A|REXO2|MTFP1|NDUFA9|LUM|SORD|SLC27A2|RHCG|UQCRC2|AFG3L2|PFKL|BCKDHA|SOD1|ECSIT|TIMM44|NAT8|ABHD10|AMT|BHMT|IQGAP2|HIGD2A|MUT|ATP6V1B2|ERLIN2|LACTB2|NDUFB9|MRPL49|CCT5|DLAT|OXSM|ACAD8|SLC25A4|UCHL1|ATP6V0D2|MRPL17|HPD|ATP5O|ACTC1|ATP6V0D1|FTCD|ATP5J2|COX7A1|CAPN2|RNPEP|FABP1|ADH5|SCIN|HSPA9|COL1A2|COL14A1|PRDX3|AMN|NDUFB8|CKB|CCT2|TPP1|IDH3A|HSP90B1|PPIB|LDHD|ANPEP|GLYATL1|PDIA3|ATP5L|CA4|TUBA1C|PRODH2|ATP5H|ECI1|SLC23A1|DCXR|HINT1|UGT2B7|LETM1|MRPL39|GAA|ATP5I|YWHAG|MRPL13|ADH1B|TMEM126A|GLB1|PDHB|CYCS|ATP6V0A4|GPD2|ACO1|DPYSL2|C19orf70|UQCRH|PRKACA|RAB1B|ISCU|CSRP2|CYP4A11|AQP1|AOC3|ACAD9|GBAS|DAB2|GRHPR|TSFM|MSRA|CRAT|TLN1|ACSS1|CYC1|PIPOX|COX5A|CES2|BCKDHB|SHMT1|UGDH|CHDH|TMEM11|UGT2B17|CALR|RHOT2|TUFM|NDUFV1|BCAT2|PLEC|HSPA5|HADHB|FBN1|CNDP2|DHRS4|MDH2|EHD3|BGN|ACSM2B|AP2A2|P4HB|NDUFA12|C11orf54|IDH2|UQCR10|SERPINA5|RAB11B|ACAA1|MRPL12|PARVA|HSP90AA1|TMLHE|DLST|PLS1|TUBB4B|CYB5A|RPS2|PRDX6|CS|SLC25A15|GNAI1|EPB41L3|PPA2|SLC25A40|TPM4|PTGES2|SAMM50|CTSB|PFKM|ALDH3A2|ANXA2|GPX4|ANXA6|UGT1A9|AGK|COL18A1|VAT1|RAC1|STOML2|PIGR|ATP5C1|GM2A|ITIH2|MSN|MYH10|COL4A2|DPP3|DPP4|LONP1|MRPL37|MPC1|CYB5R3|MT-CO1|PNP|MRPL21|OPA1|COA3|MT-CO2|DARS2|KRT19|TGM2|FH|IBA57|MARC2|IARS2|CSRP1|MGST3|ECHS1|S100A10|LHPP|RTN4IP1|HMGCS2|ATP5F1|AHCYL1|USMG5|FLNA|SFXN2|MYO6|ABCD3|GOT1|HOGA1|GNAS|XPNPEP2|ALDH18A1|SCP2|ECHDC2|SLC25A5|PMPCA|SARDH|CMPK1|ASS1|VDAC2|YWHAB|APOOL|RAB14|CISD1|OGDHL|CLIC4|HSPG2|NIPSNAP3A|ALDOB|ALDH4A1|AUH|CLIC1|COL4A1|AGMAT|PCCA|FLOT1|ALDH1B1|CUBN|SLC3A2|ACTR2|MAOB|SUCLA2|ESD|APOO|ACOT9|POSTN|TXNDC5|PDHA1|MT1M|GLRX|MPV17|BPHL|NQO2|AKR1C3|HADHA|IDH3B|DUT|PITRM1|GLDC|SLC25A6|AK3|AMACR|CD9|FECH|KRT18|SACM1L|DNM2|BDH1|DPEP1|NME2|PRCP|CNP|CORO1B|JUP|PC|SUOX|NIT2|FHL1|ACTN1|FLOT2|AK4|MVP|SNTB1|UMOD|PDP1|MGST1|TSPO|MYH11|AMPD3|ABAT|GATM|ILK|MPST|HAGH|GSTP1|ATP5A1|SEPT7|FKBP1A|CDC42|TXNRD2|RDX|ACY1|IFI30|PDIA6|TST|TF|RMDN1|ALAD|SSB|ALDH7A1|IMMT|PCYOX1|SLC25A12|SCRN1|ARPC1B|IDH1|RDH13|TKT|NDUFS1|AHSG|SELENBP1|HIGD1A|PNPT1|NAPRT|GK|ACSF2|HK1|ACTA2|DDC|CKMT2|NDRG1|COX20|CKMT1A|GPX1|FBP1|LARS2|TOMM40|AOC1|BCS1L|SERPINA1|TGFBI|VWA1|ATPAF2|MRPS22|IVD|MME|TIMMDC1|PCCB|GFM1|SSBP1|ABHD14B|ACSL1|OCIAD2|NDUFA13|MRPS30|COX7C|ETFDH|VDAC3|EPHX2|CRYAB|RAP1GAP|ECHDC1|IST1|ACADVL|ME3|P[illegible]	2.8E-34	GO.0043226	3.18601209

12193	494	GO Component	intracellular organelle	8.9E-33	RALA|DCN|NDUFB4|OXCT1|UQCRC1|VCL|MAPK1|LGALS1|RTCB|MYH9|ACO2|GSTZ1|MTHFD1|PCK2|AHCY|IDH3G|PGRMC1|F9|CRYM|CTSH|DECR1|OGDH|PPIF|PNPO|PFN1|SLC25A11|C1QBP|NAGLU|VTN|CTSC|PDHX|SLC25A3|MLEC|LTA4H|GAPDH|RAB35|DNPH1|AGXT2|SKP1|LTF|EHHADH|REEP6|ATP6V1B1|SPR|ENO1|AKR7A2|CTSD|TTR|ACADS|PEPD|GOT2|DSTN|TRAP1|CANX|TUBA4A|VIL1|HSPB1|PMPCB|MTX2|NDUFA2|ACTN4|NDUFA10|NAPSA|LGALS3|GRSF1|HRSP12|DMGDH|CARS2|LAMC1|MRPL15|GGH|SQRDL|ADAM10|LRPPRC|LYZ|PSMD11|ALDH2|ERP29|ATP5B|COTL1|CHCHD3|SLC9A3R1|PRDX1|EHD2|NAPA|MECR|LRP2|PSMD3|ATP6V0A1|NNT|ADD1|F13A1|SDHA|ACAD11|VCAN|AMBP|ENPEP|VDAC1|HIBADH|CALB1|PRDX5|CPT1A|REXO2|MTFP1|NDUFA9|LUM|SORD|SLC27A2|RHCG|UQCRC2|AFG3L2|PFKL|BCKDHA|SOD1|ECSIT|TIMM44|NAT8|ABHD10|AMT|IQGAP2|HIGD2A|MUT|ATP6V1B2|ERLIN2|LACTB2|NDUFB9|MRPL49|CCT5|DLAT|OXSM|ACAD8|SLC25A4|UCHL1|ATP6V0D2|MRPL17|HPD|ATP5O|ACTC1|ATP6V0D1|FTCD|ATP5J2|COX7A1|CAPN2|RNPEP|FABP1|ADH5|SCIN|HSPA9|COL1A2|COL14A1|PRDX3|AMN|NDUFB8|CKB|CCT2|TPP1|IDH3A|HSP90B1|PPIB|LDHD|ANPEP|GLYATL1|PDIA3|ATP5L|CA4|TUBA1C|PRODH2|ATP5H|ECI1|SLC23A1|DCXR|HINT1|UGT2B7|LETM1|MRPL39|GAA|ATP5I|YWHAG|MRPL13|ADH1B|TMEM126A|GLB1|PDHB|CYCS|ATP6V0A4|GPD2|ACO1|DPYSL2|C19orf70|UQCRH|PRKACA|RAB1B|ISCU|CSRP2|CYP4A11|AQP1|AOC3|ACAD9|GBAS|DAB2|GRHPR|TSFM|MSRA|CRAT|TLN1|ACSS1|CYC1|PIPOX|COX5A|CES2|BCKDHB|SHMT1|UGDH|CHDH|TMEM11|UGT2B17|CALR|RHOT2|TUFM|NDUFV1|BCAT2|PLEC|HSPA5|HADHB|FBN1|CNDP2|DHRS4|MDH2|EHD3|BGN|ACSM2B|AP2A2|P4HB|NDUFA12|C11orf54|IDH2|UQCR10|SERPINA5|RAB11B|ACAA1|MRPL12|PARVA|HSP90AA1|TMLHE|DLST|PLS1|TUBB4B|CYB5A|RPS2|PRDX6|CS|SLC25A15|GNAI1|EPB41L3|PPA2|SLC25A40|TPM4|PTGES2|SAMM50|CTSB|PFKM|ALDH3A2|ANXA2|GPX4|ANXA6|UGT1A9|AGK|COL18A1|VAT1|RAC1|STOML2|PIGR|ATP5C1|GM2A|ITIH2|MSN|MYH10|COL4A2|DPP3|DPP4|LONP1|MRPL37|MPC1|CYB5R3|MT-CO1|PNP|MRPL21|OPA1|COA3|MT-CO2|DARS2|KRT19|TGM2|FH|IBA57|MARC2|IARS2|CSRP1|MGST3|ECHS1|S100A10|LHPP|RTN4IP1|HMGCS2|ATP5F1|AHCYL1|USMG5|FLNA|SFXN2|MYO6|ABCD3|GOT1|HOGA1|GNAS|ALDH18A1|SCP2|ECHDC2|SLC25A5|PMPCA|SARDH|CMPK1|ASS1|VDAC2|YWHAB|APOOL|RAB14|CISD1|OGDHL|CLIC4|HSPG2|NIPSNAP3A|ALDOB|ALDH4A1|AUH|CLIC1|COL4A1|AGMAT|PCCA|FLOT1|ALDH1B1|CUBN|SLC3A2|ACTR2|MAOB|SUCLA2|ESD|APOO|ACOT9|POSTN|TXNDC5|PDHA1|MT1M|GLRX|MPV17|BPHL|NQO2|AKR1C3|HADHA|IDH3B|DUT|PITRM1|GLDC|SLC25A6|AK3|AMACR|CD9|FECH|KRT18|SACM1L|DNM2|BDH1|DPEP1|NME2|PRCP|CNP|CORO1B|JUP|PC|SUOX|NIT2|FHL1|ACTN1|FLOT2|AK4|MVP|SNTB1|UMOD|PDP1|MGST1|TSPO|MYH11|AMPD3|ABAT|GATM|ILK|MPST|HAGH|GSTP1|ATP5A1|SEPT7|FKBP1A|CDC42|TXNRD2|RDX|IFI30|PDIA6|TST|TF|RMDN1|ALAD|SSB|ALDH7A1|IMMT|PCYOX1|SLC25A12|SCRN1|ARPC1B|IDH1|RDH13|TKT|NDUFS1|AHSG|SELENBP1|HIGD1A|PNPT1|NAPRT|GK|ACSF2|HK1|ACTA2|DDC|CKMT2|NDRG1|COX20|CKMT1A|GPX1|FBP1|LARS2|TOMM40|AOC1|BCS1L|SERPINA1|TGFBI|VWA1|ATPAF2|MRPS22|IVD|MME|TIMMDC1|PCCB|GFM1|SSBP1|ABHD14B|ACSL1|OCIAD2|NDUFA13|MRPS30|COX7C|ETFDH|VDAC3|EPHX2|CRYAB|RAP1GAP|ECHDC1|IST1|ACADVL|ME3|PHB2|ATP1A1|TI[illegible]	1.66E-34	GO.0043229	3.20496351

<table>
<tr><td>11244</td><td>471</td><td>GO Component</td><td>membrane-bounded organelle</td><td>1.5E-32</td><td>RALA|DCN|NDUFB4|OXCT1|UQCRC1|VCL|MAPK1|LGALS1|RTCB|MYH9|ACO2|GSTZ1|MTHFD1|PCK2|AHCY|IDH3G|PGRMC1|F9|CRYM|CTSH|DECR1|OGDH|PPIF|PNPO|PFN1|SLC25A11|C1QBP|NAGLU|VTN|CTSC|PDHX|SLC25A3|MLEC|LTA4H|GAPDH|RAB35|DNPH1|AGXT2|SKP1|LTF|EHHADH|REEP6|ATP6V1B1|SPR|ENO1|AKR7A2|CTSD|TTR|ACADS|PEPD|GOT2|TRAP1|CANX|HSPB1|PMPCB|MTX2|NDUFA2|ACTN4|NDUFA10|NAPSA|LGALS3|GRSF1|HRSP12|DMGDH|CARS2|LAMC1|MRPL15|GGH|SQRDL|ADAM10|LRPPRC|LYZ|PSMD11|ALDH2|ERP29|ATP5B|COTL1|CHCHD3|PRDX1|EHD2|NAPA|CD81|MECR|LRP2|PSMD3|ATP6V0A1|NNT|ADD1|F13A1|SDHA|ACAD11|VCAN|AMBP|ENPEP|VDAC1|HIBADH|CALB1|PRDX5|CPT1A|REXO2|MTFP1|NDUFA9|LUM|SORD|SLC27A2|RHCG|UQCRC2|AFG3L2|PFKL|BCKDHA|SOD1|ECSIT|TIMM44|NAT8|ABHD10|AMT|BHMT|IQGAP2|HIGD2A|MUT|ATP6V1B2|ERLIN2|LACTB2|NDUFB9|MRPL49|CCT5|DLAT|OXSM|ACAD8|SLC25A4|UCHL1|ATP6V0D2|MRPL17|HPD|ATP5O|ATP6V0D1|FTCD|ATP5J2|COX7A1|CAPN2|RNPEP|FABP1|ADH5|HSPA9|COL1A2|COL14A1|PRDX3|AMN|NDUFB8|CKB|CCT2|TPP1|IDH3A|HSP90B1|PPIB|LDHD|ANPEP|GLYATL1|PDIA3|ATP5L|CA4|TUBA1C|PRODH2|ATP5H|ECI1|HINT1|UGT2B7|LETM1|MRPL39|GAA|ATP5I|YWHAG|MRPL13|ADH1B|TMEM126A|GLB1|PDHB|CYCS|ATP6V0A4|GPD2|ACO1|C19orf70|UQCRH|PRKACA|RAB1B|ISCU|CSRP2|CYP4A11|AQP1|AOC3|ACAD9|GBAS|DAB2|GRHPR|TSFM|MSRA|CRAT|ACSS1|CYC1|PIPOX|COX5A|CES2|BCKDHB|SHMT1|UGDH|CHDH|TMEM11|UGT2B17|CALR|RHOT2|TUFM|NDUFV1|BCAT2|HSPA5|HADHB|FBN1|CNDP2|DHRS4|MDH2|EHD3|BGN|ACSM2B|AP2A2|P4HB|NDUFA12|C11orf54|IDH2|UQCR10|SERPINA5|RAB11B|ACAA1|MRPL12|HSP90AA1|TMLHE|DLST|TUBB4B|CYB5A|RPS2|PRDX6|CS|SLC25A15|GNAI1|PPA2|SLC25A40|PTGES2|SAMM50|CTSB|PFKM|ALDH3A2|ANXA2|GPX4|ANXA6|UGT1A9|AGK|COL18A1|VAT1|RAC1|STOML2|PIGR|ATP5C1|GM2A|ITIH2|COL4A2|DPP3|DPP4|LONP1|MRPL37|MPC1|CYB5R3|MT-CO1|PNP|MRPL21|OPA1|COA3|MT-CO2|DARS2|TGM2|FH|IBA57|MARC2|IARS2|CSRP1|MGST3|ECHS1|S100A10|LHPP|RTN4IP1|HMGCS2|ATP5F1|AHCYL1|USMG5|FLNA|SFXN2|MYO6|ABCD3|GOT1|HOGA1|GNAS|XPNPEP2|ALDH18A1|SCP2|ECHDC2|SLC25A5|PMPCA|SARDH|CMPK1|ASS1|VDAC2|YWHAB|APOOL|RAB14|CISD1|OGDHL|CLIC4|HSPG2|NIPSNAP3A|ALDH4A1|AUH|CLIC1|COL4A1|AGMAT|PCCA|FLOT1|ALDH1B1|CUBN|SLC3A2|ACTR2|MAOB|SUCLA2|ESD|APOO|ACOT9|POSTN|TXNDC5|PDHA1|MT1M|GLRX|MPV17|BPHL|NQO2|AKR1C3|HADHA|IDH3B|DUT|PITRM1|GLDC|SLC25A6|AK3|AMACR|CD9|FECH|KRT18|SACM1L|DNM2|BDH1|DPEP1|NME2|PRCP|CNP|JUP|PC|SUOX|NIT2|FHL1|ACTN1|FLOT2|AK4|MVP|UMOD|PDP1|MGST1|TSPO|MYH11|AMPD3|ABAT|GATM|ILK|MPST|HAGH|GSTP1|ATP5A1|SEPT7|FKBP1A|CDC42|TXNRD2|ACY1|IFI30|PDIA6|TST|TF|ALAD|SSB|ALDH7A1|IMMT|PCYOX1|SLC25A12|SCRN1|IDH1|RDH13|TKT|NDUFS1|AHSG|SELENBP1|HIGD1A|PNPT1|NAPRT|GK|ACSF2|HK1|DDC|CKMT2|NDRG1|COX20|CKMT1A|GPX1|FBP1|LARS2|TOMM40|AOC1|BCS1L|SERPINA1|TGFBI|VWA1|ATPAF2|MRPS22|IVD|MME|TIMMDC1|PCCB|GFM1|SSBP1|ABHD14B|ACSL1|OCIAD2|NDUFA13|MRPS30|COX7C|ETFDH|VDAC3|EPHX2|CRYAB|RAP1GAP|ECHDC1|IST1|ACADVL|ME3|PHB2|ATP1A1|TIMM50|VIM|SLC25A10|MGAM|METTL7A|DNM1L|ALDH6A1|NPC2|ETFA|ITGB3|COX4I1|ACTG1|ACSM2A|RBM8A|TTN|SARS2|SIRT5|PHB|EPHX1|FMO1|AP2B1|HAO2|GLYAT</td><td>3.21E-34</td><td>GO.0043227</td><td>3.18326827</td></tr>
</table>

400	100	GO Process	generation of precursor metabolites and energy	7.1E-53	NDUFB4|OXCT1|UQCRC1|ACO2|IDH3G|OGDH|GAPDH|ENO1|AKR7A2|PMPCB|NDUFA2|NDUFA10|DMGDH|ALDH2|ATP5B|NNT|SDHA|NDUFA9|UQCRC2|PFKL|NDUFB9|DLAT|QDPR|SLC25A4|ATP5O|ATP5J2|COX7A1|ADH5|BDH2|ALDH1A1|NDUFB8|IDH3A|ATP5L|GPD1|ATP5H|GAA|ATP5I|ADH1B|PDHB|CYCS|GPD2|ACO1|UQCRH|GBAS|ACSS1|CYC1|COX5A|UGDH|NDUFV1|MDH2|NDUFA12|IDH2|UQCR10|DLST|PKLR|CYB5A|CS|PTGES2|PFKM|STOML2|ATP5C1|MT-CO1|MT-CO2|AKR7A3|FH|HMGCS2|PHGDH|ATP5F1|GNAS|AKR1A1|OGDHL|ALDOB|ALDH4A1|ALDH1B1|MAOB|SUCLA2|PDHA1|GLRX|NQO2|IDH3B|PFKP|GLDC|FECH|BDH1|ATP5A1|TXNRD2|SLC25A12|IDH1|TKT|NDUFS1|HK1|COX20|GBE1|COX7C|ETFDH|ACADVL|ME3|ETFA|COX4I1|GALK1	9.54E-56	GO.0006091	5.21511953

<table>
<tr><td>10365</td><td>441</td><td>GO Component</td><td>intracellular membrane-bounded organelle</td><td>5.9E-29</td><td>DCN|NDUFB4|OXCT1|UQCRC1|MAPK1|LGALS1|RTCB|MYH9|ACO2|GSTZ1|MTHFD1|PCK2|AHCY|IDH3G|PGRMC1|F9|CRYM|CTSH|DECR1|OGDH|PPIF|PNPO|PFN1|SLC25A11|C1QBP|NAGLU|VTN|CTSC|PDHX|SLC25A3|MLEC|LTA4H|GAPDH|DNPH1|AGXT2|SKP1|LTF|EHHADH|REEP6|ATP6V1B1|SPR|ENO1|AKR7A2|CTSD|TTR|ACADS|PEPD|GOT2|TRAP1|CANX|HSPB1|PMPCB|MTX2|NDUFA2|ACTN4|NDUFA10|NAPSA|LGALS3|GRSF1|HRSP12|DMGDH|CARS2|LAMC1|MRPL15|GGH|SQRDL|ADAM10|LRPPRC|LYZ|PSMD11|ALDH2|ERP29|ATP5B|COTL1|CHCHD3|PRDX1|EHD2|NAPA|MECR|LRP2|PSMD3|ATP6V0A1|NNT|ADD1|SDHA|ACAD11|VCAN|AMBP|VDAC1|HIBADH|CALB1|PRDX5|CPT1A|REXO2|MTFP1|NDUFA9|LUM|SORD|SLC27A2|UQCRC2|AFG3L2|BCKDHA|SOD1|ECSIT|TIMM44|NAT8|ABHD10|AMT|HIGD2A|MUT|ATP6V1B2|ERLIN2|LACTB2|NDUFB9|MRPL49|CCT5|DLAT|OXSM|ACAD8|SLC25A4|UCHL1|ATP6V0D2|MRPL17|HPD|ATP5O|ATP6V0D1|FTCD|ATP5J2|COX7A1|CAPN2|FABP1|ADH5|HSPA9|COL1A2|COL14A1|PRDX3|NDUFB8|CKB|CCT2|TPP1|IDH3A|HSP90B1|PPIB|LDHD|ANPEP|GLYATL1|PDIA3|ATP5L|CA4|TUBA1C|PRODH2|ATP5H|ECI1|HINT1|UGT2B7|LETM1|MRPL39|GAA|ATP5I|YWHAG|MRPL13|ADH1B|TMEM126A|GLB1|PDHB|CYCS|ATP6V0A4|GPD2|ACO1|C19orf70|UQCRH|PRKACA|RAB1B|ISCU|CSRP2|CYP4A11|AQP1|AOC3|ACAD9|GBAS|DAB2|GRHPR|TSFM|MSRA|CRAT|ACSS1|CYC1|PIPOX|COX5A|CES2|BCKDHB|SHMT1|UGDH|CHDH|TMEM11|UGT2B17|CALR|RHOT2|TUFM|NDUFV1|BCAT2|HSPA5|HADHB|FBN1|CNDP2|DHRS4|MDH2|EHD3|BGN|ACSM2B|AP2A2|P4HB|NDUFA12|C11orf54|IDH2|UQCR10|SERPINA5|ACAA1|MRPL12|HSP90AA1|TMLHE|DLST|TUBB4B|CYB5A|RPS2|PRDX6|CS|SLC25A15|GNAI1|PPA2|SLC25A40|PTGES2|SAMM50|CTSB|PFKM|ALDH3A2|ANXA2|GPX4|ANXA6|UGT1A9|AGK|COL18A1|VAT1|RAC1|STOML2|PIGR|ATP5C1|GM2A|ITIH2|COL4A2|DPP3|LONP1|MRPL37|MPC1|CYB5R3|MT-CO1|PNP|MRPL21|OPA1|COA3|MT-CO2|DARS2|TGM2|FH|IBA57|MARC2|IARS2|CSRP1|MGST3|ECHS1|S100A10|LHPP|RTN4IP1|HMGCS2|ATP5F1|AHCYL1|USMG5|FLNA|SFXN2|MYO6|ABCD3|GOT1|HOGA1|GNAS|ALDH18A1|SCP2|ECHDC2|SLC25A5|PMPCA|SARDH|CMPK1|ASS1|VDAC2|YWHAB|APOOL|RAB14|CISD1|OGDHL|CLIC4|HSPG2|NIPSNAP3A|ALDH4A1|AUH|CLIC1|COL4A1|AGMAT|PCCA|ALDH1B1|CUBN|SLC3A2|ACTR2|MAOB|SUCLA2|ESD|APOO|ACOT9|POSTN|TXNDC5|PDHA1|MT1M|GLRX|MPV17|BPHL|NQO2|AKR1C3|HADHA|IDH3B|DUT|PITRM1|GLDC|SLC25A6|AK3|AMACR|FECH|KRT18|SACM1L|DNM2|BDH1|DPEP1|PRCP|CNP|JUP|PC|SUOX|FHL1|AK4|MVP|UMOD|PDP1|MGST1|TSPO|ABAT|GATM|ILK|MPST|HAGH|GSTP1|ATP5A1|SEPT7|FKBP1A|CDC42|TXNRD2|IFI30|PDIA6|TST|TF|SSB|ALDH7A1|IMMT|PCYOX1|SLC25A12|SCRN1|IDH1|RDH13|TKT|NDUFS1|AHSG|SELENBP1|HIGD1A|PNPT1|NAPRT|GK|ACSF2|HK1|CKMT2|NDRG1|COX20|CKMT1A|GPX1|FBP1|LARS2|TOMM40|AOC1|BCS1L|SERPINA1|TGFBI|VWA1|ATPAF2|MRPS22|IVD|TIMMDC1|PCCB|GFM1|SSBP1|ABHD14B|ACSL1|NDUFA13|MRPS30|COX7C|ETFDH|VDAC3|EPHX2|CRYAB|RAP1GAP|ECHDC1|IST1|ACADVL|ME3|PHB2|ATP1A1|TIMM50|VIM|SLC25A10|METTL7A|DNM1L|ALDH6A1|NPC2|ETFA|ITGB3|COX4I1|ACSM2A|RBM8A|TTN|SARS2|SIRT5|PHB|EPHX1|FMO1|AP2B1|HAO2|GLYAT</td><td>1.46E-30</td><td>GO.0043231</td><td>2.82306227</td></tr>
</table>

<table>
<tr><td>388</td><td>96</td><td>GO Process</td><td>small molecule catabolic process</td><td>2.6E-50</td><td>OXCT1|GSTZ1|AHCY|CRYM|DECR1|GAPDH|AGXT2|EHHADH|ENO1|ACADS|GOT2|HRSP12|KHK|ALDH2|MECR|ACAD11|HIBADH|CPT1A|SORD|SLC27A2|PFKL|BCKDHA|ABHD10|ALDH1L1|AMT|DPYS|HNMT|ACAD8|QDPR|HGD|DDAH1|HPD|FTCD|ADH5|BDH2|ALDH1A1|CRYL1|HPRT1|PRODH2|ECI1|DCXR|GLB1|GPD2|CYP4A11|CRAT|PIPOX|BCKDHB|SHMT1|BCAT2|HADHB|ACAA1|DLST|PKLR|PFKM|ALDH3A2|PNP|ECHS1|ABCD3|GOT1|HOGA1|SCP2|ECHDC2|SARDH|AKR1A1|ALDOB|ALDH4A1|AUH|ALDH1B1|ESD|AKR1C3|HADHA|PFKP|GLDC|AMACR|BDH1|DAK|QPRT|ABAT|MPST|HAGH|FAH|TST|ALDH7A1|PCYOX1|GK|HK1|IVD|PCCB|ETFDH|ECHDC1|ACADVL|PAH|ALDH6A1|ETFA|GALK1|HAO2</td><td>4.05E-53</td><td>GO.0044282</td><td>4.95816987</td></tr>
<tr><td>8420</td><td>306</td><td>GO Component</td><td>membrane</td><td>9.7E-06</td><td>RALA|NDUFB4|UQCRC1|VCL|MAPK1|RTCB|MYH9|NID2|PGRMC1|F9|OGDH|PLGRKT|PPIF|SLC25A11|C1QBP|VTN|CTSC|SLC25A3|MLEC|GAPDH|RAB35|REEP6|ATP6V1B1|ENO1|CTSD|GOT2|TRAP1|CANX|VIL1|HSPB1|PMPCB|MTX2|NDUFA2|NDUFA10|LGALS3|MRPL15|SQRDL|ADAM10|LRPPRC|ATP5B|CHCHD3|SLC9A3R1|EHD2|NAPA|CD81|LRP2|ATP6V0A1|NNT|ADD1|SDHA|ACAD11|AMBP|ENPEP|VDAC1|CPT1A|MTFP1|NDUFA9|SORD|SLC27A2|RHCG|UQCRC2|AFG3L2|BCAM|ECSIT|TIMM44|NAT8|ATP6V1A|IQGAP2|HIGD2A|ATP6V1B2|ERLIN2|NDUFB9|MRPL49|SLC25A4|GPD1L|UCHL1|ATP6V0D2|MRPL17|HPD|ATP5O|ATP6V0D1|FTCD|ATP5J2|COX7A1|CAPN2|RNPEP|SCIN|AMN|NDUFB8|CDH16|HSP90B1|LDHD|ANPEP|PDIA3|ATP5L|CA4|PRODH2|ATP5H|SLC23A1|DCXR|HINT1|UGT2B7|LETM1|MRPL39|GAA|ATP5I|MRPL13|ADH1B|TMEM126A|CYCS|ATP6V0A4|GPD2|DPYSL2|C19orf70|UQCRH|PRKACA|RAB1B|CYP4A11|AQP1|AOC3|ACAD9|GBAS|DAB2|MSRA|CRAT|TLN1|CYC1|COX5A|CHDH|TMEM11|SLC7A8|UGT2B17|CALR|RHOT2|NDUFV1|PLEC|HSPA5|HADHB|DHRS4|MDH2|EHD3|BGN|AP2A2|P4HB|SLC5A2|NDUFA12|UQCR10|SERPINA5|RAB11B|MRPL12|PARVA|HSP90AA1|CYB5A|SLC25A15|GNAI1|EPB41L3|SLC25A40|PTGES2|SAMM50|PFKM|ALDH3A2|ANXA2|ANXA6|UGT1A9|AGK|VAT1|RAC1|STOML2|PIGR|ATP5C1|GM2A|MSN|MYH10|DPP3|DPP4|MRPL37|MPC1|CYB5R3|MT-CO1|MRPL21|OPA1|COA3|MT-CO2|KRT19|TGM2|MARC2|C4BPA|ATP1B1|MGST3|S100A10|RTN4IP1|ATP5F1|AHCYL1|USMG5|FLNA|SFXN2|MYO6|ABCD3|GNAS|XPNPEP2|ALDH18A1|SLC25A5|PMPCA|AKR1A1|ASS1|COMTD1|VDAC2|APOOL|RAB14|CISD1|CLIC4|HSPG2|CLIC1|FLOT1|KCTD12|CUBN|SLC3A2|MAOB|APOO|MPV17|BPHL|HADHA|GLDC|SLC25A6|AMACR|CD9|FECH|SACM1L|DNM2|BDH1|DPEP1|PRCP|CNP|CORO1B|JUP|FHL1|ACTN1|FLOT2|SNTB1|UMOD|MGST1|TSPO|GATM|ILK|GGT5|GSTP1|ATP5A1|SEPT7|FKBP1A|CDC42|GGT1|RDX|PDIA6|TF|IMMT|PCYOX1|SLC25A12|SCRN1|RDH13|NDUFS1|SLC4A4|PDZK1|SELENBP1|HIGD1A|PNPT1|GK|HK1|CKMT2|NDRG1|COX20|CKMT1A|TOMM40|AOC1|BCS1L|SERPINA1|TGFBI|MRPS22|MME|TIMMDC1|ACSL1|NDUFA13|MRPS30|COX7C|ETFDH|VDAC3|CRYAB|RAP1GAP|ACADVL|PHB2|ATP1A1|TIMM50|VIM|SLC25A10|MGAM|METTL7A|DNM1L|ITGB3|COX4I1|ACTG1|SIRT5|PHB|EPHX1|PDLIM5|FMO1|PLCG2|AP2B1</td><td>1.24E-06</td><td>GO.0016020</td><td>0.50136762</td></tr>
<tr><td>467</td><td>96</td><td>GO Process</td><td>cofactor metabolic process</td><td>3.1E-44</td><td>GSTZ1|MTHFD1|AHCY|OGDH|PNPO|PDHX|GAPDH|SPR|ENO1|AKR7A2|GOT2|BHMT2|GSTM3|GGH|PRDX1|NNT|AMBP|PRDX5|NDUFA9|PFKL|SOD1|NAT8|ALDH1L1|MUT|DLAT|OXSM|QDPR|GPD1L|FTCD|BDH2|HSPA9|PRDX3|AMN|GPD1|SLC23A1|DCXR|PDHB|GPD2|ISCU|ACSS1|PIPOX|SHMT1|CNDP2|MDH2|ACSM2B|IDH2|HSP90AA1|DLST|GSTA1|PKLR|CYB5A|PRDX6|PFKM|MPC1|CYB5R3|PNP|IBA57|GSTO1|AHCYL1|GOT1|AKR1A1|OGDHL|ALDOB|PCCA|PSAT1|CUBN|MAOB|SUCLA2|ACOT9|PDHA1|AKR1C3|PFKP|FECH|GPX3|DPEP1|PC|QPRT|TSPO|HAGH|GGT5|GSTP1|GGT1|ALAD|IDH1|TKT|NAPRT|ACSF2|HK1|GPX1|PCCB|GSTA2|ACSL1|ACSM2A|GALK1|FMO1|GLYAT</td><td>5.31E-47</td><td>GO.0051186</td><td>4.35142786</td></tr>
</table>

<table>
<tr><td>5162</td><td>283</td><td>GO Component</td><td>intracellular organelle lumen</td><td>1.1E-28</td><td>DCN|OXCT1|VCL|MAPK1|LGALS1|RTCB|ACO2|GSTZ1|PCK2|IDH3G|F9|CRYM|CTSH|DECR1|OGDH|PPIF|PNPO|C1QBP|NAGLU|VTN|CTSC|PDHX|LTA4H|AGXT2|SKP1|LTF|EHHADH|SPR|CTSD|TTR|ACADS|PEPD|GOT2|TRAP1|CANX|PMPCB|MTX2|ACTN4|NDUFA10|NAPSA|GRSF1|HRSP12|DMGDH|CARS2|LAMC1|MRPL15|GGH|ADAM10|LRPPRC|LYZ|PSMD11|ALDH2|ERP29|ATP5B|COTL1|MECR|LRP2|PSMD3|ATP6V0A1|ADD1|F13A1|SDHA|VCAN|VDAC1|HIBADH|PRDX5|REXO2|NDUFA9|LUM|SLC27A2|UQCRC2|PFKL|BCKDHA|SOD1|ECSIT|TIMM44|ABHD10|AMT|MUT|LACTB2|MRPL49|CCT5|DLAT|ACAD8|UCHL1|MRPL17|FABP1|HSPA9|COL1A2|COL14A1|PRDX3|NDUFB8|CCT2|TPP1|IDH3A|HSP90B1|PPIB|PDIA3|ATP5H|ECI1|HINT1|MRPL39|GAA|MRPL13|ADH1B|GLB1|PDHB|CYCS|C19orf70|PRKACA|ISCU|DAB2|GRHPR|TSFM|MSRA|CRAT|ACSS1|PIPOX|CES2|BCKDHB|UGDH|CALR|TUFM|BCAT2|HSPA5|HADHB|FBN1|CNDP2|DHRS4|MDH2|BGN|ACSM2B|P4HB|C11orf54|IDH2|ACAA1|MRPL12|HSP90AA1|TMLHE|DLST|TUBB4B|RPS2|PRDX6|CS|GNAI1|PPA2|PTGES2|CTSB|ANXA2|GPX4|AGK|COL18A1|VAT1|STOML2|ATP5C1|GM2A|ITIH2|COL4A2|DPP3|LONP1|MRPL37|CYB5R3|PNP|MRPL21|OPA1|DARS2|FH|IBA57|IARS2|ECHS1|LHPP|HMGCS2|ATP5F1|FLNA|MYO6|ABCD3|GOT1|HOGA1|SCP2|SLC25A5|PMPCA|SARDH|CMPK1|VDAC2|APOOL|OGDHL|CLIC4|HSPG2|ALDH4A1|AUH|COL4A1|PCCA|ALDH1B1|CUBN|ACTR2|SUCLA2|ESD|ACOT9|TXNDC5|PDHA1|NQO2|HADHA|IDH3B|DUT|PITRM1|GLDC|AK3|AMACR|FECH|KRT18|BDH1|NME2|CNP|JUP|PC|SUOX|NIT2|ACTN1|AK4|MVP|UMOD|PDP1|AMPD3|ABAT|GATM|ILK|MPST|HAGH|GSTP1|ATP5A1|TXNRD2|IFI30|PDIA6|TST|TF|ALAD|ALDH7A1|IDH1|TKT|NDUFS1|AHSG|SELENBP1|HIGD1A|PNPT1|NAPRT|ACSF2|GPX1|LARS2|AOC1|SERPINA1|VWA1|ATPAF2|MRPS22|IVD|TIMMDC1|PCCB|GFM1|SSBP1|ABHD14B|NDUFA13|ETFDH|EPHX2|CRYAB|IST1|ACADVL|ME3|PHB2|TIMM50|VIM|SLC25A10|METTL7A|ALDH6A1|NPC2|ETFA|ACSM2A|RBM8A|TTN|SARS2|SIRT5|PHB|FMO1|HAO2|GLYAT</td><td>2.81E-30</td><td>GO.0070013</td><td>2.79746941</td></tr>
<tr><td>662</td><td>107</td><td>GO Process</td><td>nucleobase-containing small molecule metabolic process</td><td>4.2E-41</td><td>NDUFB4|UQCRC1|NANS|MAPK1|MTHFD1|AHCY|OGDH|PDHX|GAPDH|DNPH1|ATP6V1B1|ENO1|GDA|PMPCB|NDUFA2|NDUFA10|ATP5B|ATP6V0A1|NNT|SDHA|PRDX5|REXO2|NDUFA9|UQCRC2|PFKL|ATP6V1A|ATP6V1B2|DPYS|NDUFB9|DLAT|OXSM|GPD1L|ATP5O|ATP5J2|HPRT1|NDUFB8|ATP5L|GPD1|ATP5H|DCXR|HINT1|ATP5I|PDHB|CYCS|ATP6V0A4|GPD2|UQCRH|AQP1|GBAS|ACSS1|CYC1|PIPOX|COX5A|SHMT1|UGDH|NDUFV1|MDH2|ACSM2B|NDUFA12|IDH2|UQCR10|DLST|PKLR|PFKM|STOML2|ATP5C1|MPC1|MT-CO1|PNP|OPA1|MT-CO2|ATP1B1|LHPP|ATP5F1|AHCYL1|CMPK1|OGDHL|ALDOB|SUCLA2|ACOT9|PDHA1|GLRX|DUT|PFKP|AK3|NME2|CNP|AK4|QPRT|AMPD3|ATP5A1|IDH1|TKT|NDUFS1|NAPRT|ACSF2|HK1|GPX1|ABHD14B|ACSL1|COX7C|ALDH6A1|COX4I1|ACSM2A|GALK1|FMO1|GLYAT</td><td>8.77E-44</td><td>GO.0055086</td><td>4.0377786</td></tr>
<tr><td>477</td><td>93</td><td>GO Process</td><td>monocarboxylic acid metabolic process</td><td>4.2E-41</td><td>DCN|DECR1|OGDH|PDHX|LTA4H|GAPDH|AGXT2|EHHADH|ENO1|ACADS|GOT2|MECR|ACAD11|VCAN|ALDH8A1|CPT1A|SORD|SLC27A2|PFKL|ABHD10|DLAT|OXSM|FTCD|BDH2|CRYL1|PRODH2|ECI1|DCXR|UGT2B7|PDHB|CYP4A11|ACAD9|GRHPR|CRAT|ACSS1|CES2|PCK1|UGT2B17|HADHB|BGN|ACSM2B|IDH2|ACAA1|GSTA1|PKLR|PTGES2|PFKM|ALDH3A2|GPX4|UGT1A9|MPC1|ECHS1|PHGDH|ABCD3|HOGA1|SCP2|ECHDC2|AKR1A1|OGDHL|ALDOB|ALDH4A1|AUH|PCCA|PDHA1|AKR1C3|HADHA|PFKP|AMACR|PC|NIT2|ABAT|HAGH|GGT5|GSTP1|GGT1|PTGR1|IDH1|ACSF2|HK1|GPX1|IVD|PCCB|ACSL1|ETFDH|EPHX2|ECHDC1|ACADVL|ME3|ETFA|ACSM2A|GALK1|HAO2|GLYAT</td><td>8.09E-44</td><td>GO.0032787</td><td>4.0377786</td></tr>
</table>

<table>
<tr><td>5281</td><td>316</td><td>GO Process</td><td>organonitrogen compound metabolic process</td><td>7.6E-41</td><td>DCN|NDUFB4|UQCRC1|MAPK1|LGALS1|MYH9|GSTZ1|MTHFD1|AHCY|F9|CRYM|CTSH|OGDH|PPIF|PNPO|C1QBP|NAGLU|CTSC|PDHX|LTA4H|GAPDH|DNPH1|AGXT2|SKP1|LTF|EHHADH|ATP6V1B1|SPR|ENO1|CTSD|TTR|GDA|PEPD|GOT2|PMPCB|NDUFA2|NDUFA10|NAPSA|HRSP12|APCS|DMGDH|BHMT2|GSTM3|CARS2|LAMC1|TINAG|MRPL15|GGH|ADAM10|LYZ|PEBP1|PSMD11|ATP5B|BBOX1|PSMD3|ATP6V0A1|NNT|ADD1|F13A1|SDHA|VCAN|AMBP|ENPEP|HIBADH|CALB1|PRDX5|CPT1A|EIF4H|NDUFA9|LUM|UQCRC2|AFG3L2|PFKL|BCKDHA|SOD1|TINAGL1|NAT8|ATP6V1A|ALDH1L1|AMT|BHMT|MUT|ATP6V1B2|ERLIN2|DPYS|NDUFB9|MRPL49|HNMT|DLAT|OXSM|ACAD8|QDPR|GPD1L|HGD|DDAH1|UCHL1|MRPL17|HPD|ATP5O|FTCD|ATP5J2|CAPN2|RNPEP|ADH5|BDH2|PRDX3|HPRT1|AMN|NDUFB8|CKB|TPP1|HSP90B1|PPIB|ANPEP|GLYATL1|PDIA3|ATP5L|GPD1|PRODH2|ATP5H|DCXR|HINT1|MRPL39|ATP5I|MRPL13|GLB1|ASL|PDHB|CYCS|ATP6V0A4|GPD2|UQCRH|PRKACA|RAB1B|ISCU|AQP1|AOC3|GBAS|TSFM|MSRA|CRAT|ACSS1|CYC1|PIPOX|COX5A|BCKDHB|SHMT1|UGDH|PCK1|CHDH|SLC7A8|CALR|TUFM|NDUFV1|BCAT2|HSPA5|FBN1|CNDP2|MDH2|BGN|ACSM2B|AP2A2|P4HB|NDUFA12|IDH2|UQCR10|RAB11B|MRPL12|HSP90AA1|TMLHE|DLST|GSTA1|PKLR|RPS2|SLC25A15|TPSAB1|PPA2|CTSB|PFKM|ALDH3A2|ANXA2|ALDH9A1|AGK|RAC1|STOML2|ATP5C1|GM2A|ITIH2|DPP3|DPP4|LONP1|MRPL37|MPC1|MT-CO1|PNP|MRPL21|OPA1|MT-CO2|DARS2|TGM2|IBA57|IARS2|C4BPA|ATP1B1|MGST3|LHPP|PHGDH|GSTO1|ATP5F1|AHCYL1|GOT1|HOGA1|XPNPEP2|ALDH18A1|PMPCA|SARDH|CMPK1|AKR1A1|ASS1|YWHAB|OGDHL|HSPG2|ALDOB|ALDH4A1|VARS|AUH|AGMAT|PCCA|PSAT1|CUBN|MAOB|SUCLA2|ESD|ACOT9|PDHA1|GLRX|BPHL|DUT|PITRM1|PFKP|GLDC|AK3|FECH|DPEP1|NME2|PRCP|PC|NIT2|AK4|QPRT|PDP1|MGST1|TSPO|AMPD3|ABAT|GATM|ILK|MPST|HAGH|GGT5|GSTP1|ATP5A1|FKBP1A|CDC42|GGT1|ACY1|FAH|PDIA6|SMS|TST|TF|ALAD|ALDH7A1|CPVL|PCYOX1|SCRN1|IDH1|TKT|NDUFS1|AHSG|NAPRT|ACSF2|HK1|DDC|CKMT2|CKMT1A|GPX1|LARS2|AOC1|SERPINA1|TGFBI|VWA1|MRPS22|IVD|MME|PCCB|GFM1|ABHD14B|GSTA2|ACSL1|MRPS30|COX7C|CRYAB|TIMM50|PAH|ALDH6A1|COX4I1|ACSM2A|GALK1|TTN|SARS2|SIRT5|PHB|FMO1|AP2B1|GLYAT</td><td>1.76E-43</td><td>GO.1901564</td><td>4.0118045</td></tr>
</table>

9569	446	GO Process	metabolic process	9.8E-41	DCN|NDUFB4|OXCT1|UQCRC1|NANS|MAPK1|LGALS1|RTCB|MYH9|ACO2|GSTZ1|MTHFD1|PCK2|AHCY|IDH3G|F9|CRYM|CTSH|DECR1|OGDH|PPIF|PNPO|SLC25A11|C1QBP|NAGLU|CTSC|PDHX|MLEC|LTA4H|GAPDH|DNPH1|AGXT2|SKP1|LTF|EHHADH|ATP6V1B1|SPR|ENO1|AKR7A2|CTSD|TTR|GDA|HDHD3|ACADS|PEPD|GOT2|PMPCB|NDUFA2|NDUFA10|NAPSA|LGALS3|GRSF1|HRSP12|APCS|DMGDH|BHMT2|GSTM3|CARS2|LAMC1|TINAG|MRPL15|GGH|SQRDL|ADAM10|KHK|LRPPRC|LYZ|PEBP1|PSMD11|ALDH2|ATP5B|CHCHD3|PRDX1|BBOX1|CD81|MECR|LRP2|PSMD3|ATP6V0A1|NNT|ADD1|F13A1|SDHA|ACAD11|VCAN|AMBP|ENPEP|VDAC1|HIBADH|CALB1|PRDX5|ALDH8A1|CPT1A|EIF4H|REXO2|NDUFA9|LUM|SORD|SLC27A2|UQCRC2|AFG3L2|PFKL|BCKDHA|SOD1|ECSIT|TINAGL1|NAT8|ABHD10|ATP6V1A|ALDH1L1|AMT|BHMT|HIGD2A|MUT|ATP6V1B2|ERLIN2|LACTB2|DPYS|NDUFB9|MRPL49|HNMT|DLAT|OXSM|ACAD8|QDPR|SLC25A4|GPD1L|HGD|DDAH1|UCHL1|MRPL17|HPD|ATP5O|FTCD|ATP5J2|COX7A1|CAPN2|RNPEP|FABP1|ADH5|BDH2|CMBL|HSPA9|ALDH1A1|CRYL1|PRDX3|HPRT1|AMN|NDUFB8|CKB|TPP1|IDH3A|HSP90B1|PPIB|LDHD|ANPEP|GLYATL1|PDIA3|ATP5L|GPD1|PRODH2|ATP5H|ECI1|SLC23A1|DCXR|HINT1|UGT2B7|MRPL39|GAA|ATP5I|MRPL13|ADH1B|GLB1|ASL|PDHB|CYCS|ATP6V0A4|GPD2|ACO1|DPYSL2|UQCRH|PRKACA|RAB1B|ISCU|CYP4A11|AQP1|AOC3|ACAD9|GBAS|GRHPR|TSFM|MSRA|CRAT|ACSS1|CYC1|PIPOX|COX5A|CES2|BCKDHB|SHMT1|UGDH|PCK1|CHDH|SLC7A8|UGT2B17|CALR|TUFM|NDUFV1|BCAT2|HSPA5|HADHB|FBN1|CNDP2|DHRS4|MDH2|EHD3|BGN|ACSM2B|AP2A2|P4HB|SLC5A2|NDUFA12|IDH2|UQCR10|RAB11B|ACAA1|MRPL12|HSP90AA1|TMLHE|DLST|GSTA1|PKLR|CYB5A|RPS2|PRDX6|CS|SLC25A15|TPSAB1|PPA2|PTGES2|CTSB|PFKM|ALDH3A2|ANXA2|GPX4|UGT1A9|ALDH9A1|AGK|VAT1|RAC1|STOML2|ATP5C1|GM2A|ITIH2|PBLD|COL4A2|DPP3|DPP4|LONP1|MRPL37|MPC1|CYB5R3|MT-CO1|PNP|MRPL21|OPA1|MT-CO2|DARS2|TGM2|AKR7A3|FH|IBA57|MARC2|IARS2|C4BPA|ATP1B1|MGST3|ECHS1|LHPP|RTN4IP1|HMGCS2|PHGDH|GSTO1|ATP5F1|AHCYL1|FLNA|ABCD3|GOT1|HOGA1|GNAS|XPNPEP2|ALDH18A1|RBP4|SCP2|ECHDC2|PMPCA|SARDH|CMPK1|AKR1A1|ASS1|COMTD1|YWHAB|RAB14|OGDHL|HSPG2|ALDOB|ALDH4A1|VARS|AUH|AGMAT|PCCA|PSAT1|ALDH1B1|CUBN|SLC3A2|MAOB|SUCLA2|ESD|ACOT9|PDHA1|GLRX|BPHL|NQO2|AKR1C3|HADHA|IDH3B|DUT|PITRM1|PFKP|GLDC|AK3|AMACR|CD9|FECH|GPX3|SACM1L|DNM2|BDH1|DPEP1|NME2|PRCP|CNP|PC|SUOX|NIT2|DAK|AK4|QPRT|PDP1|MGST1|TSPO|AMPD3|ABAT|GATM|ILK|MPST|HAGH|GGT5|GSTP1|ATP5A1|FKBP1A|CDC42|GGT1|TXNRD2|ACY1|IFI30|FAH|PDIA6|SMS|PTGR1|TST|TF|ALAD|SSB|ALDH7A1|CPVL|PCYOX1|SLC25A12|SCRN1|IDH1|RDH13|TKT|NDUFS1|AHSG|SELENBP1|HIGD1A|CRYZ|PNPT1|NAPRT|GK|ACSF2|HK1|DDC|CKMT2|COX20|CKMT1A|GPX1|FBP1|LARS2|GBE1|AOC1|SERPINA1|TGFBI|VWA1|MRPS22|IVD|MME|PCCB|GFM1|SSBP1|ABHD14B|GSTA2|ACSL1|NDUFA13|MRPS30|COX7C|ETFDH|EPHX2|CRYAB|ECHDC1|ACADVL|ME3|PHB2|ATP1A1|TIMM50|SLC25A10|MGAM|PAH|METTL7A|ALDH6A1|NPC2|ETFA|COX4I1|ACSM2A|RBM8A|GALK1|TTN|SARS2|SIRT5|PHB|EPHX1|FMO1|PLCG2|AP2B1|HAO2|GLYAT	2.45E-43	GO.0008152	4.00101054
581	100	GO Process	nucleoside phosphate metabolic process	2.2E-40	NDUFB4|UQCRC1|MAPK1|MTHFD1|OGDH|PDHX|GAPDH|DNPH1|ATP6V1B1|ENO1|GDA|PMPCB|NDUFA2|NDUFA10|ATP5B|ATP6V0A1|NNT|SDHA|PRDX5|REXO2|NDUFA9|UQCRC2|PFKL|ATP6V1A|ATP6V1B2|NDUFB9|DLAT|OXSM|GPD1L|ATP5O|ATP5J2|HPRT1|NDUFB8|ATP5L|GPD1|ATP5H|DCXR|HINT1|ATP5I|PDHB|CYCS|ATP6V0A4|GPD2|UQCRH|AQP1|GBAS|ACSS1|CYC1|PIPOX|COX5A|SHMT1|NDUFV1|MDH2|ACSM2B|NDUFA12|IDH2|UQCR10|DLST|PKLR|PFKM|STOML2|ATP5C1|MPC1|MT-CO1|PNP|OPA1|MT-CO2|ATP1B1|LHPP|ATP5F1|CMPK1|OGDHL|ALDOB|SUCLA2|ACOT9|PDHA1|DUT|PFKP|AK3|NME2|CNP|AK4|QPRT|AMPD3|ATP5A1|IDH1|TKT|NDUFS1|NAPRT|ACSF2|HK1|GPX1|ABHD14B|ACSL1|COX7C|COX4I1|ACSM2A|GALK1|FMO1|GLYAT	5.92E-43	GO.0006753	3.96595559

237	70	GO Process	carboxylic acid catabolic process	2.5E-40	GSTZ1|AHCY|CRYM|DECR1|AGXT2|EHHADH|ACADS|GOT2|HRSP12|MECR|ACAD11|HIBADH|CPT1A|SORD|SLC27A2|BCKDHA|ABHD10|ALDH1L1|AMT|HNMT|ACAD8|QDPR|HGD|DDAH1|HPD|FTCD|BDH2|CRYL1|PRODH2|ECI1|DCXR|CYP4A11|CRAT|PIPOX|BCKDHB|SHMT1|BCAT2|HADHB|ACAA1|DLST|ALDH3A2|ECHS1|ABCD3|GOT1|HOGA1|SCP2|ECHDC2|SARDH|AKR1A1|ALDH4A1|AUH|HADHA|GLDC|AMACR|QPRT|ABAT|MPST|FAH|TST|ALDH7A1|PCYOX1|IVD|PCCB|ETFDH|ECHDC1|ACADVL|PAH|ALDH6A1|ETFA|HAO2	7.15E-43	GO.0046395	3.9607303
576	99	GO Process	nucleotide metabolic process	5.6E-40	NDUFB4|UQCRC1|MAPK1|MTHFD1|OGDH|PDHX|GAPDH|DNPH1|ATP6V1B1|ENO1|GDA|PMPCB|NDUFA2|NDUFA10|ATP5B|ATP6V0A1|NNT|SDHA|PRDX5|REXO2|NDUFA9|UQCRC2|PFKL|ATP6V1A|ATP6V1B2|NDUFB9|DLAT|OXSM|GPD1L|ATP5O|ATP5J2|HPRT1|NDUFB8|ATP5L|GPD1|ATP5H|DCXR|HINT1|ATP5I|PDHB|CYCS|ATP6V0A4|GPD2|UQCRH|AQP1|GBAS|ACSS1|CYC1|PIPOX|COX5A|SHMT1|NDUFV1|MDH2|ACSM2B|NDUFA12|IDH2|UQCR10|DLST|PKLR|PFKM|STOML2|ATP5C1|MPC1|MT-CO1|PNP|OPA1|MT-CO2|ATP1B1|ATP5F1|CMPK1|OGDHL|ALDOB|SUCLA2|ACOT9|PDHA1|DUT|PFKP|AK3|NME2|CNP|AK4|QPRT|AMPD3|ATP5A1|IDH1|TKT|NDUFS1|NAPRT|ACSF2|HK1|GPX1|ABHD14B|ACSL1|COX7C|COX4I1|ACSM2A|GALK1|FMO1|GLYAT	1.82E-42	GO.0009117	3.92549252
1531	256	GO Component	mitochondrion	2E-115	NDUFB4|OXCT1|UQCRC1|MAPK1|ACO2|GSTZ1|MTHFD1|PCK2|IDH3G|CRYM|DECR1|OGDH|PPIF|SLC25A11|C1QBP|PDHX|SLC25A3|AGXT2|ACADS|GOT2|TRAP1|PMPCB|MTX2|NDUFA2|NDUFA10|LGALS3|GRSF1|HRSP12|DMGDH|CARS2|MRPL15|SQRDL|LRPPRC|ALDH2|ATP5B|CHCHD3|MECR|NNT|SDHA|ACAD11|VDAC1|HIBADH|PRDX5|CPT1A|REXO2|MTFP1|NDUFA9|SORD|UQCRC2|AFG3L2|BCKDHA|SOD1|ECSIT|TIMM44|ABHD10|AMT|HIGD2A|MUT|LACTB2|NDUFB9|MRPL49|DLAT|OXSM|ACAD8|SLC25A4|MRPL17|ATP5O|ATP5J2|COX7A1|ADH5|HSPA9|PRDX3|NDUFB8|IDH3A|LDHD|GLYATL1|ATP5L|PRODH2|ATP5H|ECI1|LETM1|MRPL39|ATP5I|YWHAG|MRPL13|TMEM126A|PDHB|CYCS|GPD2|ACO1|C19orf70|UQCRH|PRKACA|ISCU|ACAD9|GBAS|TSFM|MSRA|CRAT|ACSS1|CYC1|COX5A|BCKDHB|CHDH|TMEM11|RHOT2|TUFM|NDUFV1|BCAT2|HSPA5|HADHB|DHRS4|MDH2|ACSM2B|NDUFA12|IDH2|UQCR10|MRPL12|TMLHE|DLST|CYB5A|CS|SLC25A15|PPA2|SLC25A40|PTGES2|SAMM50|GPX4|ANXA6|AGK|VAT1|STOML2|ATP5C1|LONP1|MRPL37|MPC1|CYB5R3|MT-CO1|MRPL21|OPA1|COA3|MT-CO2|DARS2|TGM2|FH|IBA57|MARC2|IARS2|ECHS1|RTN4IP1|HMGCS2|ATP5F1|USMG5|SFXN2|ABCD3|HOGA1|ALDH18A1|SCP2|ECHDC2|SLC25A5|PMPCA|SARDH|ASS1|VDAC2|YWHAB|APOOL|CISD1|OGDHL|CLIC4|NIPSNAP3A|ALDH4A1|AUH|CLIC1|AGMAT|PCCA|ALDH1B1|MAOB|SUCLA2|APOO|ACOT9|PDHA1|MPV17|BPHL|HADHA|IDH3B|DUT|PITRM1|GLDC|SLC25A6|AK3|AMACR|FECH|DNM2|BDH1|CNP|PC|SUOX|AK4|PDP1|MGST1|TSPO|ABAT|GATM|MPST|HAGH|GSTP1|ATP5A1|TXNRD2|TST|ALDH7A1|IMMT|SLC25A12|IDH1|RDH13|NDUFS1|HIGD1A|PNPT1|GK|ACSF2|HK1|CKMT2|COX20|CKMT1A|GPX1|LARS2|TOMM40|BCS1L|ATPAF2|MRPS22|IVD|TIMMDC1|PCCB|GFM1|SSBP1|ACSL1|NDUFA13|MRPS30|COX7C|ETFDH|VDAC3|CRYAB|ECHDC1|ACADVL|ME3|PHB2|TIMM50|SLC25A10|DNM1L|ALDH6A1|ETFA|COX4I1|ACSM2A|SARS2|SIRT5|PHB|GLYAT	2.9E-118	GO.0005739	11.4725842

<table>
<tr><th></th><th></th><th></th><th></th><th></th><th></th><th></th><th></th><th></th></tr>
<tr><td>1646</td><td>160</td><td>GO Process</td><td>cellular catabolic process</td><td>7.7E-38</td><td>DCN|OXCT1|GSTZ1|AHCY|CRYM|CTSH|DECR1|OGDH|CTSC|LTA4H|GAPDH|DNPH1|AGXT2|SKP1|EHHADH|ENO1|CTSD|GDA|ACADS|GOT2|NAPSA|HRSP12|DMGDH|GSTM3|LYZ|PSMD11|ALDH2|PRDX1|MECR|PSMD3|ACAD11|VCAN|AMBP|ENPEP|VDAC1|HIBADH|PRDX5|CPT1A|LUM|SORD|SLC27A2|PFKL|BCKDHA|ABHD10|ALDH1L1|AMT|BHMT|ERLIN2|DPYS|HNMT|ACAD8|QDPR|HGD|DDAH1|UCHL1|HPD|FTCD|CAPN2|RNPEP|FABP1|ADH5|BDH2|CRYL1|PRDX3|HPRT1|TPP1|HSP90B1|ANPEP|PRODH2|ECI1|DCXR|HINT1|GAA|GLB1|GPD2|RAB1B|CYP4A11|CRAT|PIPOX|BCKDHB|SHMT1|CHDH|BCAT2|HSPA5|HADHB|BGN|AP2A2|ACAA1|HSP90AA1|DLST|PKLR|RPS2|PRDX6|CTSB|PFKM|ALDH3A2|GM2A|LONP1|PNP|ECHS1|GSTO1|ABCD3|GOT1|HOGA1|SCP2|ECHDC2|SARDH|AKR1A1|OGDHL|ALDOB|ALDH4A1|AUH|ALDH1B1|MAOB|ESD|AKR1C3|HADHA|DUT|PFKP|GLDC|AMACR|GPX3|BDH1|DPEP1|CNP|QPRT|AMPD3|ABAT|MPST|HAGH|GGT5|GGT1|FAH|TST|SSB|ALDH7A1|CPVL|PCYOX1|CRYZ|PNPT1|GK|HK1|GPX1|IVD|PCCB|ACSL1|ETFDH|EPHX2|ECHDC1|ACADVL|MGAM|PAH|ALDH6A1|ETFA|RBM8A|GALK1|EPHX1|PLCG2|AP2B1|HAO2</td><td>2.67E-40</td><td>GO.0044248</td><td>3.71140737</td></tr>
<tr><td>308</td><td>74</td><td>GO Process</td><td>cellular amino acid metabolic process</td><td>1.2E-37</td><td>GSTZ1|MTHFD1|AHCY|CRYM|AGXT2|PEPD|GOT2|HRSP12|BHMT2|CARS2|HIBADH|BCKDHA|AMT|BHMT|MUT|DPYS|HNMT|ACAD8|QDPR|HGD|DDAH1|HPD|FTCD|CKB|GLYATL1|PRODH2|ASL|MSRA|PIPOX|BCKDHB|SHMT1|SLC7A8|BCAT2|P4HB|DLST|PPA2|DARS2|IARS2|PHGDH|AHCYL1|GOT1|HOGA1|ALDH18A1|SARDH|ASS1|ALDH4A1|VARS|AUH|AGMAT|PSAT1|BPHL|GLDC|DPEP1|NIT2|ABAT|GATM|MPST|GGT5|GGT1|ACY1|FAH|SMS|TST|ALDH7A1|PCYOX1|DDC|CKMT2|CKMT1A|LARS2|IVD|PAH|ALDH6A1|SARS2|GLYAT</td><td>4.43E-40</td><td>GO.0006520</td><td>3.69172146</td></tr>
<tr><td>1859</td><td>170</td><td>GO Process</td><td>catabolic process</td><td>1.8E-37</td><td>DCN|OXCT1|GSTZ1|AHCY|CRYM|CTSH|DECR1|OGDH|NAGLU|CTSC|LTA4H|GAPDH|DNPH1|AGXT2|SKP1|EHHADH|ENO1|CTSD|GDA|ACADS|PEPD|GOT2|NAPSA|HRSP12|DMGDH|GSTM3|KHK|LYZ|PSMD11|ALDH2|PRDX1|MECR|PSMD3|ACAD11|VCAN|AMBP|ENPEP|VDAC1|HIBADH|PRDX5|CPT1A|LUM|SORD|SLC27A2|PFKL|BCKDHA|ABHD10|ALDH1L1|AMT|BHMT|ERLIN2|DPYS|HNMT|ACAD8|QDPR|GPD1L|HGD|DDAH1|UCHL1|HPD|FTCD|CAPN2|RNPEP|FABP1|ADH5|BDH2|ALDH1A1|CRYL1|PRDX3|HPRT1|TPP1|HSP90B1|ANPEP|GPD1|PRODH2|ECI1|DCXR|HINT1|GAA|GLB1|GPD2|RAB1B|CYP4A11|CRAT|PIPOX|CES2|BCKDHB|SHMT1|CHDH|BCAT2|HSPA5|HADHB|BGN|AP2A2|ACAA1|HSP90AA1|DLST|PKLR|RPS2|PRDX6|CTSB|PFKM|ALDH3A2|ANXA2|GM2A|LONP1|PNP|ECHS1|GSTO1|ABCD3|GOT1|HOGA1|SCP2|ECHDC2|SARDH|AKR1A1|OGDHL|HSPG2|ALDOB|ALDH4A1|AUH|ALDH1B1|MAOB|ESD|AKR1C3|HADHA|DUT|PFKP|GLDC|AMACR|GPX3|BDH1|DPEP1|CNP|DAK|QPRT|AMPD3|ABAT|MPST|HAGH|GGT5|GGT1|FAH|TST|SSB|ALDH7A1|CPVL|PCYOX1|CRYZ|PNPT1|GK|HK1|GPX1|IVD|PCCB|ACSL1|ETFDH|EPHX2|ECHDC1|ACADVL|MGAM|PAH|ALDH6A1|ETFA|RBM8A|GALK1|EPHX1|PLCG2|AP2B1|HAO2</td><td>6.77E-40</td><td>GO.0009056</td><td>3.6756962</td></tr>
<tr><td>481</td><td>88</td><td>GO Function</td><td>cofactor binding</td><td>6.5E-37</td><td>ACO2|AHCY|IDH3G|PGRMC1|CRYM|DECR1|OGDH|PNPO|GAPDH|AGXT2|SPR|ACADS|GOT2|GSTM3|SQRDL|ALDH2|NNT|SDHA|ACAD11|AMBP|HIBADH|NDUFA9|SORD|MUT|ACAD8|QDPR|GPD1L|BDH2|ALDH1A1|CRYL1|IDH3A|LDHD|GPD1|PRODH2|CYCS|ACO1|ISCU|CYP4A11|AOC3|ACAD9|GRHPR|CYC1|SHMT1|UGDH|CHDH|NDUFV1|IDH2|CYB5A|PTGES2|CYB5R3|MT-CO1|MARC2|PHGDH|GOT1|SCP2|CISD1|OGDHL|PCCA|ALDH1B1|CUBN|MAOB|HBD|NQO2|HADHA|IDH3B|GLDC|FECH|PC|SUOX|MGST1|ABAT|GSTP1|TXNRD2|IDH1|TKT|NDUFS1|CRYZ|DDC|AOC1|IVD|ETFDH|ACADVL|ME3|ALDH6A1|ETFA|SIRT5|FMO1|HAO2</td><td>1.61E-39</td><td>GO.0048037</td><td>3.61877553</td></tr>
</table>

1609	156	GO Process	organic substance catabolic process	1.2E-36	DCN|OXCT1|GSTZ1|AHCY|CRYM|CTSH|DECR1|OGDH|NAGLU|CTSC|LTA4H|GAPDH|DNPH1|AGXT2|SKP1|EHHADH|ENO1|CTSD|GDA|ACADS|GOT2|NAPSA|HRSP12|DMGDH|KHK|LYZ|PSMD11|ALDH2|MECR|PSMD3|ACAD11|VCAN|AMBP|ENPEP|HIBADH|CPT1A|LUM|SORD|SLC27A2|PFKL|BCKDHA|ABHD10|ALDH1L1|AMT|BHMT|ERLIN2|DPYS|HNMT|ACAD8|QDPR|GPD1L|HGD|DDAH1|UCHL1|HPD|FTCD|CAPN2|RNPEP|FABP1|ADH5|BDH2|ALDH1A1|CRYL1|HPRT1|TPP1|HSP90B1|ANPEP|GPD1|PRODH2|ECI1|DCXR|HINT1|GAA|GLB1|GPD2|CYP4A11|CRAT|PIPOX|BCKDHB|SHMT1|CHDH|BCAT2|HSPA5|HADHB|BGN|AP2A2|ACAA1|HSP90AA1|DLST|PKLR|RPS2|PRDX6|CTSB|PFKM|ALDH3A2|ANXA2|GM2A|LONP1|PNP|ECHS1|ABCD3|GOT1|HOGA1|SCP2|ECHDC2|SARDH|AKR1A1|OGDHL|HSPG2|ALDOB|ALDH4A1|AUH|ALDH1B1|MAOB|ESD|AKR1C3|HADHA|DUT|PFKP|GLDC|AMACR|BDH1|CNP|DAK|QPRT|AMPD3|ABAT|MPST|HAGH|GGT5|GGT1|FAH|TST|SSB|ALDH7A1|CPVL|PCYOX1|PNPT1|GK|HK1|GPX1|IVD|PCCB|ETFDH|EPHX2|ECHDC1|ACADVL|MGAM|PAH|ALDH6A1|ETFA|RBM8A|GALK1|PLCG2|AP2B1|HAO2	4.65E-39	GO.1901575	3.59393022
116	50	KEGG Pathways	Carbon metabolism	5.1E-35	ACO2|IDH3G|OGDH|GAPDH|EHHADH|ENO1|ACADS|GOT2|SDHA|PFKL|AMT|MUT|DLAT|ADH5|IDH3A|PDHB|ACO1|ACSS1|SHMT1|MDH2|IDH2|DLST|PKLR|CS|PFKM|FH|ECHS1|PHGDH|GOT1|OGDHL|ALDOB|PCCA|PSAT1|SUCLA2|ESD|PDHA1|HADHA|IDH3B|PFKP|GLDC|PC|DAK|IDH1|TKT|HK1|FBP1|PCCB|ME3|ALDH6A1|HAO2	3.59E-37	hsa01200	3.42941363

<table>
<tr><th></th><th></th><th></th><th></th><th></th><th></th><th></th><th></th><th></th></tr>
<tr><td>8797</td><td>412</td><td>GO Process</td><td>cellular metabolic process</td><td>1.9E-34</td><td>DCN|NDUFB4|OXCT1|UQCRC1|NANS|MAPK1|LGALS1|RTCB|ACO2|GSTZ1|MTHFD1|AHCY|IDH3G|CRYM|CTSH|DECR1|OGDH|PPIF|PNPO|C1QBP|CTSC|PDHX|LTA4H|GAPDH|DNPH1|AGXT2|SKP1|LTF|EHHADH|ATP6V1B1|SPR|ENO1|AKR7A2|CTSD|TTR|GDA|ACADS|PEPD|GOT2|PMPCB|NDUFA2|NDUFA10|NAPSA|LGALS3|GRSF1|HRSP12|APCS|DMGDH|BHMT2|GSTM3|CARS2|LAMC1|MRPL15|GGH|SQRDL|ADAM10|KHK|LRPPRC|LYZ|PEBP1|PSMD11|ALDH2|ATP5B|CHCHD3|PRDX1|BBOX1|CD81|MECR|LRP2|PSMD3|ATP6V0A1|NNT|ADD1|F13A1|SDHA|ACAD11|VCAN|AMBP|ENPEP|VDAC1|HIBADH|CALB1|PRDX5|ALDH8A1|CPT1A|EIF4H|REXO2|NDUFA9|LUM|SORD|SLC27A2|UQCRC2|AFG3L2|PFKL|BCKDHA|SOD1|NAT8|ABHD10|ATP6V1A|ALDH1L1|AMT|BHMT|MUT|ATP6V1B2|ERLIN2|LACTB2|DPYS|NDUFB9|MRPL49|HNMT|DLAT|OXSM|ACAD8|QDPR|SLC25A4|GPD1L|HGD|DDAH1|UCHL1|MRPL17|HPD|ATP5O|FTCD|ATP5J2|COX7A1|CAPN2|RNPEP|FABP1|ADH5|BDH2|CMBL|HSPA9|ALDH1A1|CRYL1|PRDX3|HPRT1|AMN|NDUFB8|CKB|TPP1|IDH3A|HSP90B1|PPIB|ANPEP|GLYATL1|ATP5L|GPD1|PRODH2|ATP5H|ECI1|SLC23A1|DCXR|HINT1|UGT2B7|MRPL39|GAA|ATP5I|MRPL13|ADH1B|GLB1|ASL|PDHB|CYCS|ATP6V0A4|GPD2|ACO1|DPYSL2|UQCRH|PRKACA|RAB1B|ISCU|CYP4A11|AQP1|AOC3|ACAD9|GBAS|GRHPR|TSFM|MSRA|CRAT|ACSS1|CYC1|PIPOX|COX5A|CES2|BCKDHB|SHMT1|UGDH|PCK1|CHDH|SLC7A8|UGT2B17|TUFM|NDUFV1|BCAT2|HSPA5|HADHB|FBN1|CNDP2|DHRS4|MDH2|EHD3|BGN|ACSM2B|AP2A2|P4HB|NDUFA12|IDH2|UQCR10|RAB11B|ACAA1|MRPL12|HSP90AA1|TMLHE|DLST|GSTA1|PKLR|CYB5A|RPS2|PRDX6|CS|SLC25A15|PPA2|PTGES2|CTSB|PFKM|ALDH3A2|GPX4|UGT1A9|ALDH9A1|AGK|RAC1|STOML2|ATP5C1|GM2A|ITIH2|COL4A2|LONP1|MRPL37|MPC1|CYB5R3|MT-CO1|PNP|MRPL21|OPA1|MT-CO2|DARS2|TGM2|AKR7A3|FH|IBA57|MARC2|IARS2|ATP1B1|MGST3|ECHS1|LHPP|HMGCS2|PHGDH|GSTO1|ATP5F1|AHCYL1|FLNA|ABCD3|GOT1|HOGA1|GNAS|ALDH18A1|RBP4|SCP2|ECHDC2|PMPCA|SARDH|CMPK1|AKR1A1|ASS1|YWHAB|RAB14|OGDHL|HSPG2|ALDOB|ALDH4A1|VARS|AUH|AGMAT|PCCA|PSAT1|ALDH1B1|CUBN|MAOB|SUCLA2|ESD|ACOT9|PDHA1|GLRX|BPHL|NQO2|AKR1C3|HADHA|IDH3B|DUT|PFKP|GLDC|AK3|AMACR|CD9|FECH|GPX3|SACM1L|DNM2|BDH1|DPEP1|NME2|CNP|PC|SUOX|NIT2|DAK|AK4|QPRT|PDP1|MGST1|TSPO|AMPD3|ABAT|GATM|ILK|MPST|HAGH|GGT5|GSTP1|ATP5A1|FKBP1A|CDC42|GGT1|TXNRD2|ACY1|FAH|PDIA6|SMS|PTGR1|TST|TF|ALAD|SSB|ALDH7A1|CPVL|PCYOX1|SLC25A12|IDH1|RDH13|TKT|NDUFS1|AHSG|CRYZ|PNPT1|NAPRT|GK|ACSF2|HK1|DDC|CKMT2|COX20|CKMT1A|GPX1|FBP1|LARS2|GBE1|AOC1|SERPINA1|TGFBI|VWA1|MRPS22|IVD|MME|PCCB|GFM1|SSBP1|ABHD14B|GSTA2|ACSL1|NDUFA13|MRPS30|COX7C|ETFDH|EPHX2|CRYAB|ECHDC1|ACADVL|ME3|PHB2|ATP1A1|TIMM50|SLC25A10|MGAM|PAH|ALDH6A1|ETFA|COX4I1|ACSM2A|RBM8A|GALK1|TTN|SARS2|SIRT5|PHB|EPHX1|FMO1|PLCG2|AP2B1|HAO2|GLYAT</td><td>8.13E-37</td><td>GO.0044237</td><td>3.37189666</td></tr>
</table>

<table>
<tr><th></th><th></th><th></th><th></th><th></th><th></th><th></th><th></th><th></th></tr>
<tr><td>9135</td><td>420</td><td>GO Process</td><td>organic substance metabolic process</td><td>8.7E-34</td><td>DCN|NDUFB4|OXCT1|UQCRC1|NANS|MAPK1|LGALS1|RTCB|MYH9|ACO2|GSTZ1|MTHFD1|PCK2|AHCY|IDH3G|F9|CRYM|CTSH|DECR1|OGDH|PPIF|PNPO|SLC25A11|C1QBP|NAGLU|CTSC|PDHX|MLEC|LTA4H|GAPDH|DNPH1|AGXT2|SKP1|LTF|EHHADH|ATP6V1B1|SPR|ENO1|AKR7A2|CTSD|TTR|GDA|ACADS|PEPD|GOT2|PMPCB|NDUFA2|NDUFA10|NAPSA|LGALS3|GRSF1|HRSP12|APCS|DMGDH|BHMT2|GSTM3|CARS2|LAMC1|TINAG|MRPL15|GGH|ADAM10|KHK|LRPPRC|LYZ|PEBP1|PSMD11|ALDH2|ATP5B|CHCHD3|BBOX1|CD81|MECR|LRP2|PSMD3|ATP6V0A1|NNT|ADD1|F13A1|SDHA|ACAD11|VCAN|AMBP|ENPEP|HIBADH|CALB1|PRDX5|ALDH8A1|CPT1A|EIF4H|REXO2|NDUFA9|LUM|SORD|SLC27A2|UQCRC2|AFG3L2|PFKL|BCKDHA|SOD1|TINAGL1|NAT8|ABHD10|ATP6V1A|ALDH1L1|AMT|BHMT|MUT|ATP6V1B2|ERLIN2|LACTB2|DPYS|NDUFB9|MRPL49|HNMT|DLAT|OXSM|ACAD8|QDPR|GPD1L|HGD|DDAH1|UCHL1|MRPL17|HPD|ATP5O|FTCD|ATP5J2|CAPN2|RNPEP|FABP1|ADH5|BDH2|ALDH1A1|CRYL1|PRDX3|HPRT1|AMN|NDUFB8|CKB|TPP1|IDH3A|HSP90B1|PPIB|ANPEP|GLYATL1|PDIA3|ATP5L|GPD1|PRODH2|ATP5H|ECI1|SLC23A1|DCXR|HINT1|UGT2B7|MRPL39|GAA|ATP5I|MRPL13|ADH1B|GLB1|ASL|PDHB|CYCS|ATP6V0A4|GPD2|ACO1|DPYSL2|UQCRH|PRKACA|RAB1B|ISCU|CYP4A11|AQP1|AOC3|ACAD9|GBAS|GRHPR|TSFM|MSRA|CRAT|ACSS1|CYC1|PIPOX|COX5A|CES2|BCKDHB|SHMT1|UGDH|PCK1|CHDH|SLC7A8|UGT2B17|CALR|TUFM|NDUFV1|BCAT2|HSPA5|HADHB|FBN1|CNDP2|DHRS4|MDH2|EHD3|BGN|ACSM2B|AP2A2|P4HB|SLC5A2|NDUFA12|IDH2|UQCR10|RAB11B|ACAA1|MRPL12|HSP90AA1|TMLHE|DLST|GSTA1|PKLR|CYB5A|RPS2|PRDX6|CS|SLC25A15|TPSAB1|PPA2|PTGES2|CTSB|PFKM|ALDH3A2|ANXA2|GPX4|UGT1A9|ALDH9A1|AGK|RAC1|STOML2|ATP5C1|GM2A|ITIH2|COL4A2|DPP3|DPP4|LONP1|MRPL37|MPC1|CYB5R3|MT-CO1|PNP|MRPL21|OPA1|MT-CO2|DARS2|TGM2|AKR7A3|FH|IBA57|MARC2|IARS2|C4BPA|ATP1B1|MGST3|ECHS1|LHPP|HMGCS2|PHGDH|GSTO1|ATP5F1|AHCYL1|FLNA|ABCD3|GOT1|HOGA1|GNAS|XPNPEP2|ALDH18A1|RBP4|SCP2|ECHDC2|PMPCA|SARDH|CMPK1|AKR1A1|ASS1|YWHAB|RAB14|OGDHL|HSPG2|ALDOB|ALDH4A1|VARS|AUH|AGMAT|PCCA|PSAT1|ALDH1B1|CUBN|SLC3A2|MAOB|SUCLA2|ESD|ACOT9|PDHA1|GLRX|BPHL|AKR1C3|HADHA|IDH3B|DUT|PITRM1|PFKP|GLDC|AK3|AMACR|CD9|FECH|SACM1L|DNM2|BDH1|DPEP1|NME2|PRCP|CNP|PC|SUOX|NIT2|DAK|AK4|QPRT|PDP1|MGST1|TSPO|AMPD3|ABAT|GATM|ILK|MPST|HAGH|GGT5|GSTP1|ATP5A1|FKBP1A|CDC42|GGT1|ACY1|FAH|PDIA6|SMS|PTGR1|TST|TF|ALAD|SSB|ALDH7A1|CPVL|PCYOX1|SLC25A12|SCRN1|IDH1|RDH13|TKT|NDUFS1|AHSG|PNPT1|NAPRT|GK|ACSF2|HK1|DDC|CKMT2|CKMT1A|GPX1|FBP1|LARS2|GBE1|AOC1|SERPINA1|TGFBI|VWA1|MRPS22|IVD|MME|PCCB|GFM1|SSBP1|ABHD14B|GSTA2|ACSL1|MRPS30|COX7C|ETFDH|EPHX2|CRYAB|ECHDC1|ACADVL|ME3|PHB2|TIMM50|SLC25A10|MGAM|PAH|ALDH6A1|NPC2|ETFA|COX4I1|ACSM2A|RBM8A|GALK1|TTN|SARS2|SIRT5|PHB|EPHX1|FMO1|PLCG2|AP2B1|HAO2|GLYAT</td><td>3.86E-36</td><td>GO.0071704</td><td>3.30604807</td></tr>
<tr><td>478</td><td>83</td><td>GO Process</td><td>purine-containing compound metabolic process</td><td>1.9E-33</td><td>NDUFB4|UQCRC1|MTHFD1|AHCY|OGDH|PDHX|GAPDH|DNPH1|ATP6V1B1|ENO1|GDA|PMPCB|NDUFA2|NDUFA10|ATP5B|ATP6V0A1|SDHA|NDUFA9|UQCRC2|PFKL|ATP6V1A|ATP6V1B2|NDUFB9|DLAT|OXSM|ATP5O|ATP5J2|HPRT1|NDUFB8|ATP5L|ATP5H|HINT1|ATP5I|PDHB|CYCS|ATP6V0A4|UQCRH|AQP1|GBAS|ACSS1|CYC1|PIPOX|COX5A|SHMT1|NDUFV1|ACSM2B|NDUFA12|UQCR10|DLST|PKLR|PFKM|STOML2|ATP5C1|MPC1|MT-CO1|PNP|OPA1|MT-CO2|ATP1B1|ATP5F1|AHCYL1|OGDHL|ALDOB|SUCLA2|ACOT9|PDHA1|PFKP|AK3|NME2|AK4|AMPD3|ATP5A1|NDUFS1|ACSF2|HK1|GPX1|ABHD14B|ACSL1|COX7C|COX4I1|ACSM2A|GALK1|GLYAT</td><td>8.69E-36</td><td>GO.0072521</td><td>3.27258422</td></tr>
</table>

442	80	GO Process	purine nucleotide metabolic process	2.7E-33	NDUFB4|UQCRC1|MTHFD1|OGDH|PDHX|GAPDH|DNPH1|ATP6V1B1|ENO1|GDA|PMPCB|NDUFA2|NDUFA10|ATP5B|ATP6V0A1|SDHA|NDUFA9|UQCRC2|PFKL|ATP6V1A|ATP6V1B2|NDUFB9|DLAT|OXSM|ATP5O|ATP5J2|HPRT1|NDUFB8|ATP5L|ATP5H|HINT1|ATP5I|PDHB|CYCS|ATP6V0A4|UQCRH|AQP1|GBAS|ACSS1|CYC1|PIPOX|COX5A|NDUFV1|ACSM2B|NDUFA12|UQCR10|DLST|PKLR|PFKM|STOML2|ATP5C1|MPC1|MT-CO1|PNP|OPA1|MT-CO2|ATP1B1|ATP5F1|OGDHL|ALDOB|SUCLA2|ACOT9|PDHA1|PFKP|AK3|NME2|AK4|AMPD3|ATP5A1|NDUFS1|ACSF2|HK1|GPX1|ABHD14B|ACSL1|COX7C|COX4I1|ACSM2A|GALK1|GLYAT	1.31E-35	GO.0006163	3.25654311
4958	248	GO Component	cytosol	2.6E-18	UQCRC1|NANS|VCL|MAPK1|LGALS1|RTCB|MYH9|GSTZ1|MTHFD1|AHCY|CRYM|CTSH|DECR1|PNPO|PFN1|C1QBP|LTA4H|GAPDH|RAB35|DNPH1|SKP1|EHHADH|ATP6V1B1|SPR|ENO1|AKR7A2|GDA|TUBA4A|HSPB1|ACTN4|HRSP12|BHMT2|GSTM3|GGH|KHK|PEBP1|PSMD11|PRDX1|BBOX1|EHD2|NAPA|PSMD3|ATP6V0A1|ADD1|CALB1|PRDX5|ALDH8A1|EIF4H|SORD|SLC27A2|PFKL|SOD1|ECSIT|ABHD10|ATP6V1A|ALDH1L1|BHMT|IQGAP2|ATP6V1B2|ERLIN2|DPYS|HNMT|CCT5|OXSM|QDPR|GPD1L|HGD|DDAH1|UCHL1|HPD|ACTC1|FTCD|CAPN2|FABP1|ADH5|BDH2|CMBL|ALDH1A1|CRYL1|PRDX3|HPRT1|CKB|CCT2|HSP90B1|GPD1|HINT1|YWHAG|ADH1B|ASL|CYCS|ACO1|DPYSL2|PRKACA|RAB1B|ISCU|DAB2|GRHPR|MSRA|CRAT|TLN1|PIPOX|SHMT1|UGDH|PCK1|CALR|RHOT2|NDUFV1|PLEC|CNDP2|DHRS4|EHD3|AP2A2|NDUFA12|IDH2|RAB11B|ACAA1|PARVA|HSP90AA1|GSTA1|PKLR|TUBB4B|CYB5A|RPS2|PRDX6|EPB41L3|TPM4|PTGES2|PFKM|ANXA2|GPX4|ALDH9A1|AGK|RAC1|MSN|MYH10|DPP3|LONP1|CYB5R3|PNP|OPA1|KRT19|TGM2|AKR7A3|IARS2|LHPP|PHGDH|GSTO1|AHCYL1|FLNA|MYO6|ABCD3|GOT1|GNAS|ALDH18A1|RBP4|SCP2|CMPK1|AKR1A1|ASS1|YWHAB|RAB14|CLIC4|NIPSNAP3A|ALDOB|VARS|PCCA|PSAT1|CUBN|SLC3A2|RSU1|ACTR2|APOO|GLRX|MPV17|HBD|NQO2|AKR1C3|PFKP|AMACR|KRT18|DNM2|NME2|CNP|CORO1B|JUP|PC|NIT2|FHL1|ACTN1|DAK|MVP|QPRT|MYH11|AMPD3|ILK|MPST|HAGH|GSTP1|SEPT7|FKBP1A|CDC42|TXNRD2|ACY1|IFI30|FAH|PDIA6|SMS|ALAD|ALDH7A1|ARPC1B|IDH1|TKT|SELENBP1|CRYZ|PNPT1|NAPRT|GK|HK1|ACTA2|DDC|NDRG1|GPX1|FBP1|TOMM40|GBE1|ATPAF2|PCCB|ABHD14B|GSTA2|EPHX2|CRYAB|RAP1GAP|ECHDC1|IST1|ACADVL|VIM|MYL6|PAH|DNM1L|ACTG1|RBM8A|GALK1|TTN|SIRT5|PDLIM5|PLCG2|AP2B1|HAO2	9.67E-20	GO.0005829	1.75867002

<table>
<tr><th></th><th></th><th></th><th></th><th></th><th></th><th></th><th></th><th></th></tr>
<tr><td>3337</td><td>215</td><td>GO Component</td><td>organelle membrane</td><td>1.5E-28</td><td>RALA|NDUFB4|UQCRC1|RTCB|PGRMC1|OGDH|PPIF|SLC25A11|CTSC|SLC25A3|MLEC|GAPDH|RAB35|REEP6|ATP6V1B1|GOT2|TRAP1|CANX|PMPCB|MTX2|NDUFA2|NDUFA10|LGALS3|MRPL15|SQRDL|ADAM10|LRPPRC|ATP5B|CHCHD3|EHD2|NAPA|LRP2|ATP6V0A1|NNT|SDHA|ACAD11|VDAC1|CPT1A|MTFP1|NDUFA9|SORD|SLC27A2|UQCRC2|AFG3L2|ECSIT|TIMM44|NAT8|IQGAP2|HIGD2A|ERLIN2|NDUFB9|MRPL49|SLC25A4|UCHL1|ATP6V0D2|MRPL17|HPD|ATP5O|ATP6V0D1|FTCD|ATP5J2|COX7A1|AMN|NDUFB8|HSP90B1|LDHD|ANPEP|PDIA3|ATP5L|CA4|PRODH2|ATP5H|UGT2B7|LETM1|MRPL39|GAA|ATP5I|MRPL13|TMEM126A|CYCS|ATP6V0A4|GPD2|C19orf70|UQCRH|RAB1B|CYP4A11|AQP1|ACAD9|GBAS|DAB2|CRAT|CYC1|COX5A|CHDH|TMEM11|UGT2B17|CALR|RHOT2|NDUFV1|HSPA5|HADHB|DHRS4|EHD3|AP2A2|NDUFA12|UQCR10|SERPINA5|RAB11B|MRPL12|CYB5A|SLC25A15|SLC25A40|PTGES2|SAMM50|ALDH3A2|ANXA2|ANXA6|UGT1A9|AGK|VAT1|RAC1|STOML2|PIGR|ATP5C1|MRPL37|MPC1|CYB5R3|MT-CO1|MRPL21|OPA1|COA3|MT-CO2|MARC2|MGST3|RTN4IP1|ATP5F1|AHCYL1|USMG5|SFXN2|MYO6|GNAS|ALDH18A1|SLC25A5|PMPCA|ASS1|VDAC2|APOOL|RAB14|CISD1|CLIC4|CLIC1|CUBN|MAOB|APOO|MPV17|BPHL|HADHA|SLC25A6|CD9|FECH|SACM1L|DNM2|BDH1|PRCP|CNP|FLOT2|UMOD|MGST1|TSPO|GATM|ATP5A1|FKBP1A|CDC42|PDIA6|TF|IMMT|PCYOX1|SLC25A12|SCRN1|RDH13|NDUFS1|HIGD1A|PNPT1|GK|HK1|CKMT2|NDRG1|COX20|CKMT1A|TOMM40|BCS1L|SERPINA1|MRPS22|MME|TIMMDC1|ACSL1|NDUFA13|MRPS30|COX7C|ETFDH|VDAC3|RAP1GAP|ACADVL|PHB2|TIMM50|SLC25A10|MGAM|DNM1L|ITGB3|COX4I1|SIRT5|PHB|EPHX1|FMO1|AP2B1</td><td>4.78E-30</td><td>GO.0031090</td><td>2.78356471</td></tr>
<tr><td>4792</td><td>189</td><td>GO Component</td><td>protein-containing complex</td><td>4.7E-05</td><td>DCN|NDUFB4|UQCRC1|VCL|MAPK1|RTCB|MYH9|CTSH|OGDH|PPIF|VTN|PDHX|GAPDH|SKP1|LTF|ATP6V1B1|ENO1|TTR|GOT2|CANX|HSPB1|PMPCB|NDUFA2|ACTN4|NDUFA10|LAMA5|LGALS3|GRSF1|LAMC1|MRPL15|LRPPRC|PSMD11|ATP5B|CHCHD3|NAPA|LRP2|PSMD3|ATP6V0A1|ADD1|SDHA|VDAC1|EIF4H|NDUFA9|LUM|UQCRC2|AFG3L2|PFKL|BCKDHA|SOD1|ATP6V1A|ATP6V1B2|ERLIN2|NDUFB9|MRPL49|CCT5|DLAT|GPD1L|ATP6V0D2|MRPL17|ATP5O|ATP6V0D1|ATP5J2|FABP1|SCIN|COL1A2|COL14A1|PRDX3|AMN|NDUFB8|CCT2|HSP90B1|PPIB|ATP5L|GPD1|ATP5H|HINT1|MRPL39|ATP5I|MRPL13|PDHB|CYCS|ATP6V0A4|GPD2|C19orf70|UQCRH|PRKACA|CYC1|COX5A|BCKDHB|CALR|NDUFV1|HSPA5|AP2A2|P4HB|NDUFA12|UQCR10|SERPINA5|MRPL12|HSP90AA1|DLST|RPS2|GNAI1|SAMM50|PFKM|ANXA2|GPX4|AGK|COL18A1|RAC1|PIGR|ATP5C1|MYH10|COL4A2|MRPL37|CYB5R3|MT-CO1|MRPL21|MT-CO2|KRT19|FH|ATP1B1|ATP5F1|USMG5|FLNA|MYO6|GNAS|RBP4|SCP2|SLC25A5|VDAC2|YWHAB|APOOL|OGDHL|CLIC4|HSPG2|CLIC1|COL4A1|FLOT1|ACTR2|APOO|PDHA1|HBD|HADHA|GLDC|SLC25A6|SACM1L|DNM2|JUP|FLOT2|MVP|SNTB1|MYH11|ABAT|ILK|GSTP1|ATP5A1|SEPT7|FKBP1A|CDC42|PDIA6|TF|SSB|IMMT|PCYOX1|ARPC1B|NDUFS1|HIGD1A|PNPT1|ACTA2|TOMM40|BCS1L|MRPS22|NDUFA13|MRPS30|COX7C|ETFDH|VDAC3|PHB2|ATP1A1|TIMM50|VIM|MYL6|DNM1L|ITGB3|COX4I1|ACTG1|RBM8A|TTN|AP2B1</td><td>6.53E-06</td><td>GO.0032991</td><td>0.43316141</td></tr>
</table>

1011	116	GO Process	organophosphate metabolic process	3.2E-32	NDUFB4|UQCRC1|MAPK1|MTHFD1|OGDH|PNPO|PDHX|GAPDH|DNPH1|ATP6V1B1|ENO1|GDA|PMPCB|NDUFA2|NDUFA10|KHK|ATP5B|ATP6V0A1|NNT|SDHA|PRDX5|REXO2|NDUFA9|SORD|UQCRC2|PFKL|ATP6V1A|ATP6V1B2|NDUFB9|DLAT|OXSM|GPD1L|ATP5O|ATP5J2|ALDH1A1|CRYL1|HPRT1|NDUFB8|ATP5L|GPD1|ATP5H|DCXR|HINT1|ATP5I|PDHB|CYCS|ATP6V0A4|GPD2|UQCRH|AQP1|GBAS|ACSS1|CYC1|PIPOX|COX5A|SHMT1|NDUFV1|MDH2|ACSM2B|NDUFA12|IDH2|UQCR10|DLST|PKLR|PRDX6|PFKM|GPX4|AGK|STOML2|ATP5C1|MPC1|MT-CO1|PNP|OPA1|MT-CO2|ATP1B1|LHPP|ATP5F1|SCP2|CMPK1|AKR1A1|RAB14|OGDHL|ALDOB|SUCLA2|ACOT9|PDHA1|DUT|PFKP|AK3|SACM1L|NME2|CNP|DAK|AK4|QPRT|AMPD3|ATP5A1|IDH1|TKT|NDUFS1|NAPRT|GK|ACSF2|HK1|GPX1|FBP1|ABHD14B|ACSL1|COX7C|COX4I1|ACSM2A|GALK1|FMO1|PLCG2|GLYAT	1.61E-34	GO.0019637	3.1493495
4347	178	GO Component	endomembrane system	1.1E-05	DCN|NDUFB4|VCL|MAPK1|LGALS1|RTCB|PGRMC1|F9|CTSH|VTN|CTSC|MLEC|LTA4H|GAPDH|RAB35|LTF|REEP6|ATP6V1B1|AKR7A2|CTSD|TTR|CANX|ACTN4|NAPSA|LGALS3|LAMC1|GGH|ADAM10|LRPPRC|LYZ|PSMD11|ERP29|COTL1|SLC9A3R1|EHD2|NAPA|LRP2|PSMD3|ATP6V0A1|F13A1|VCAN|LUM|SLC27A2|PFKL|SOD1|NAT8|IQGAP2|ATP6V1B2|ERLIN2|UCHL1|ATP6V0D2|HPD|ATP6V0D1|FTCD|CAPN2|RNPEP|COL1A2|COL14A1|PRDX3|AMN|NDUFB8|CCT2|HSP90B1|PPIB|ANPEP|PDIA3|CA4|UGT2B7|GAA|GLB1|ATP6V0A4|ACO1|PRKACA|RAB1B|CYP4A11|AQP1|AOC3|DAB2|CRAT|CES2|UGT2B17|CALR|HSPA5|HADHB|FBN1|DHRS4|EHD3|BGN|AP2A2|P4HB|SERPINA5|RAB11B|ACAA1|HSP90AA1|TUBB4B|CYB5A|PRDX6|PTGES2|CTSB|ALDH3A2|ANXA2|ANXA6|UGT1A9|COL18A1|VAT1|RAC1|PIGR|GM2A|ITIH2|COL4A2|CYB5R3|PNP|TGM2|MGST3|S100A10|AHCYL1|MYO6|GNAS|ASS1|VDAC2|APOOL|RAB14|HSPG2|CLIC1|COL4A1|FLOT1|CUBN|ACTR2|ESD|APOO|POSTN|TXNDC5|CD9|SACM1L|DNM2|NME2|PRCP|JUP|NIT2|ACTN1|FLOT2|MVP|UMOD|MGST1|AMPD3|GSTP1|FKBP1A|CDC42|PDIA6|TF|ALAD|SCRN1|IDH1|AHSG|PNPT1|NAPRT|DDC|NDRG1|AOC1|SERPINA1|TGFBI|VWA1|MME|ACSL1|OCIAD2|CRYAB|RAP1GAP|IST1|ATP1A1|MGAM|METTL7A|DNM1L|NPC2|ITGB3|PHB|EPHX1|FMO1|AP2B1	1.43E-06	GO.0012505	0.4954677
274	64	GO Function	coenzyme binding	1.3E-31	AHCY|IDH3G|CRYM|DECR1|OGDH|PNPO|GAPDH|AGXT2|SPR|ACADS|GOT2|ALDH2|NNT|SDHA|ACAD11|HIBADH|NDUFA9|SORD|ACAD8|QDPR|GPD1L|BDH2|ALDH1A1|CRYL1|IDH3A|LDHD|GPD1|PRODH2|ACAD9|GRHPR|SHMT1|UGDH|CHDH|NDUFV1|IDH2|CYB5R3|MARC2|PHGDH|GOT1|SCP2|OGDHL|PCCA|ALDH1B1|MAOB|NQO2|HADHA|IDH3B|GLDC|PC|SUOX|ABAT|TXNRD2|IDH1|CRYZ|DDC|IVD|ETFDH|ACADVL|ME3|ALDH6A1|ETFA|SIRT5|FMO1|HAO2	4.43E-34	GO.0050662	3.08728952
158	51	Reactome Pathways	The citric acid (TCA) cycle and respiratory electron transport	1.9E-31	NDUFB4|UQCRC1|GSTZ1|OGDH|PDHX|TRAP1|NDUFA2|NDUFA10|LRPPRC|NNT|SDHA|NDUFA9|UQCRC2|ECSIT|NDUFB9|DLAT|ATP5O|ATP5J2|IDH3A|ATP5L|ATP5H|ATP5I|PDHB|UQCRH|ACAD9|COX5A|NDUFV1|MDH2|NDUFA12|IDH2|UQCR10|DLST|ATP5C1|MPC1|MT-CO1|MT-CO2|FH|ATP5F1|SUCLA2|PDHA1|IDH3B|PDP1|NDUFS1|COX20|TIMMDC1|NDUFA13|COX7C|ETFDH|ME3|ETFA|COX4I1	6.33E-33	HSA-1428517	3.07212464
569	86	GO Process	small molecule biosynthetic process	8.2E-31	DCN|MTHFD1|PCK2|AHCY|OGDH|PNPO|SLC25A11|LTA4H|GAPDH|DNPH1|AGXT2|SPR|ENO1|GDA|GOT2|BHMT2|MECR|VCAN|ALDH8A1|NDUFA9|SORD|SLC27A2|PFKL|BHMT|OXSM|QDPR|BDH2|HPRT1|GPD1|HINT1|ASL|GPD2|ACSS1|SHMT1|UGDH|PCK1|BCAT2|MDH2|BGN|ACSM2B|PKLR|PTGES2|PFKM|GPX4|CYB5R3|PNP|MGST3|HMGCS2|PHGDH|ABCD3|GOT1|HOGA1|ALDH18A1|RBP4|SCP2|CMPK1|AKR1A1|ASS1|OGDHL|ALDOB|PSAT1|SUCLA2|AKR1C3|DUT|PFKP|AMACR|BDH1|NME2|CNP|PC|AMPD3|ABAT|GATM|GGT5|GGT1|SLC25A12|TKT|HK1|GPX1|FBP1|EPHX2|SLC25A10|PAH|ACSM2A|GALK1|PLCG2	4.26E-33	GO.0044283	3.00872467

2460	185	GO Function	small molecule binding	1.1E-30	RALA|MAPK1|RTCB|MYH9|MTHFD1|PCK2|AHCY|IDH3G|CRYM|DECR1|OGDH|PNPO|PFN1|C1QBP|GAPDH|RAB35|AGXT2|ATP6V1B1|SPR|ACADS|GOT2|TRAP1|TUBA4A|ACTN4|HRSP12|CARS2|TINAG|KHK|PEBP1|ALDH2|ATP5B|EHD2|NNT|SDHA|ACAD11|VCAN|HIBADH|CALB1|RBP5|SORD|SLC27A2|AFG3L2|PFKL|TIMM44|ATP6V1A|MUT|ATP6V1B2|ERLIN2|DPYS|CCT5|ACAD8|QDPR|GPD1L|DDAH1|ACTC1|FTCD|FABP1|ADH5|BDH2|HSPA9|ALDH1A1|CRYL1|HPRT1|CKB|CCT2|IDH3A|HSP90B1|LDHD|TUBA1C|GPD1|PRODH2|HINT1|MRPL39|PRKACA|RAB1B|ACAD9|GRHPR|ACSS1|SHMT1|UGDH|PCK1|CHDH|RHOT2|TUFM|NDUFV1|HSPA5|EHD3|ACSM2B|IDH2|SERPINA5|RAB11B|HSP90AA1|PKLR|TUBB4B|GNAI1|PFKM|ANXA6|UGT1A9|AGK|RAC1|MYH10|LONP1|CYB5R3|PNP|OPA1|DARS2|TGM2|MARC2|IARS2|PHGDH|MYO6|ABCD3|GOT1|GNAS|ALDH18A1|RBP4|SCP2|CMPK1|ASS1|VDAC2|RAB14|OGDHL|ALDOB|VARS|PCCA|ALDH1B1|CUBN|ACTR2|MAOB|SUCLA2|HBD|NQO2|HADHA|IDH3B|PFKP|GLDC|AK3|DNM2|NME2|CNP|PC|DAK|AK4|TSPO|MYH11|ABAT|ILK|ATP5A1|SEPT7|CDC42|TXNRD2|IDH1|CRYZ|GK|ACSF2|HK1|ACTA2|DDC|CKMT2|CKMT1A|FBP1|LARS2|BCS1L|IVD|PCCB|GFM1|ACSL1|NDUFA13|ETFDH|VDAC3|ACADVL|ME3|ATP1A1|DNM1L|ALDH6A1|NPC2|ETFA|ACTG1|ACSM2A|GALK1|TTN|SARS2|SIRT5|FMO1|HAO2	4.68E-33	GO.0036094	2.99469216

14652	542	GO Process	cellular process	1.4E-30	RALA|SPATA20|DCN|NDUFB4|OXCT1|UQCRC1|NANS|VCL|MAPK1|LGALS1|RTCB|MYH9|ACO2|NID2|GSTZ1|MTHFD1|AHCY|IDH3G|PGRMC1|CRYM|CTSH|DECR1|OGDH|PPIF|PNPO|PFN1|C1QBP|NAGLU|VTN|CTSC|PDHX|MLEC|LTA4H|GAPDH|RAB35|DNPH1|AGXT2|SKP1|LTF|EHHADH|ATP6V1B1|SPR|ENO1|AKR7A2|CTSD|TTR|GDA|ACADS|PEPD|GOT2|DSTN|TRAP1|CANX|TUBA4A|VIL1|HSPB1|PMPCB|MTX2|NDUFA2|ACTN4|NDUFA10|LAMA5|NAPSA|LGALS3|GRSF1|HRSP12|APCS|DMGDH|BHMT2|GSTM3|CARS2|LAMC1|MRPL15|GGH|SQRDL|ADAM10|KHK|LRPPRC|LYZ|PEBP1|PSMD11|ALDH2|ERP29|ATP5B|COTL1|CHCHD3|SLC9A3R1|PRDX1|BBOX1|EHD2|NAPA|CD81|MECR|LRP2|PSMD3|ATP6V0A1|NNT|ADD1|F13A1|SDHA|ACAD11|VCAN|AMBP|ENPEP|VDAC1|HIBADH|CALB1|PRDX5|ALDH8A1|CPT1A|EIF4H|REXO2|MTFP1|NDUFA9|LUM|SORD|SLC27A2|RHCG|UQCRC2|AFG3L2|PFKL|BCKDHA|SOD1|BCAM|ECSIT|TIMM44|NAT8|ABHD10|ATP6V1A|ALDH1L1|AMT|BHMT|IQGAP2|MUT|ATP6V1B2|ERLIN2|LACTB2|DPYS|NDUFB9|MRPL49|HNMT|CCT5|DLAT|OXSM|ACAD8|QDPR|SLC25A4|GPD1L|HGD|DDAH1|UCHL1|ATP6V0D2|MRPL17|HPD|ATP5O|ACTC1|ATP6V0D1|FTCD|ATP5J2|COX7A1|CAPN2|RNPEP|FABP1|ADH5|BDH2|CMBL|SCIN|HSPA9|COL1A2|ALDH1A1|COL14A1|CRYL1|PRDX3|HPRT1|AMN|NDUFB8|CKB|CCT2|TPP1|IDH3A|CDH16|HSP90B1|PPIB|ANPEP|GLYATL1|PDIA3|ATP5L|TUBA1C|GPD1|PRODH2|ATP5H|ECI1|SLC23A1|DCXR|HINT1|UGT2B7|LETM1|MRPL39|GAA|ATP5I|YWHAG|MRPL13|ADH1B|GLB1|ASL|PDHB|CYCS|ATP6V0A4|GPD2|ACO1|DPYSL2|C19orf70|UQCRH|PRKACA|RAB1B|ISCU|CSRP2|CYP4A11|AQP1|AOC3|ACAD9|GBAS|DAB2|GRHPR|TSFM|MSRA|CRAT|TLN1|ACSS1|CYC1|PIPOX|COX5A|CES2|BCKDHB|SHMT1|UGDH|PCK1|CHDH|TMEM11|SLC7A8|UGT2B17|CALR|RHOT2|TUFM|NDUFV1|BCAT2|PLEC|HSPA5|HADHB|FBN1|CNDP2|DHRS4|MDH2|EHD3|BGN|ACSM2B|AP2A2|P4HB|NDUFA12|IDH2|UQCR10|SERPINA5|RAB11B|ACAA1|MRPL12|PARVA|HSP90AA1|TMLHE|DLST|GSTA1|PLS1|PKLR|TUBB4B|CYB5A|RPS2|PRDX6|CS|SLC25A15|GNAI1|EPB41L3|TPSAB1|PPA2|TPM4|PTGES2|SAMM50|CTSB|PFKM|ALDH3A2|ANXA2|GPX4|ANXA6|UGT1A9|ALDH9A1|AGK|COL18A1|VAT1|RAC1|STOML2|PIGR|ATP5C1|GM2A|ITIH2|MSN|MYH10|COL4A2|DPP4|LONP1|MRPL37|MPC1|CYB5R3|MT-CO1|PNP|MRPL21|OPA1|COA3|MT-CO2|DARS2|KRT19|TGM2|AKR7A3|FH|IBA57|MARC2|IARS2|ATP1B1|DPT|MGST3|ECHS1|S100A10|LHPP|RTN4IP1|HMGCS2|PHGDH|GSTO1|ATP5F1|AHCYL1|FLNA|MYO6|ABCD3|GOT1|HOGA1|GNAS|ALDH18A1|RBP4|SCP2|ECHDC2|SLC25A5|PMPCA|SARDH|CMPK1|AKR1A1|ASS1|VDAC2|YWHAB|APOOL|RAB14|OGDHL|CLIC4|HSPG2|ALDOB|ALDH4A1|ASPN|VARS|AUH|CLIC1|COL4A1|AGMAT|PCCA|FLOT1|PSAT1|KCTD12|ALDH1B1|CUBN|SLC3A2|RSU1|ACTR2|MAOB|SUCLA2|ESD|APOO|ACOT9|POSTN|TXNDC5|PDHA1|MT1M|GLRX|MPV17|BPHL|NQO2|AKR1C3|HADHA|IDH3B|DUT|PITRM1|PFKP|GLDC|SLC25A6|AK3|AMACR|CD9|FECH|GPX3|KRT18|SACM1L|DNM2|BDH1|DPEP1|NME2|PRCP|CNP|CORO1B|JUP|PC|SUOX|NIT2|FHL1|ACTN1|DAK|FLOT2|AK4|MVP|QPRT|UMOD|PDP1|MGST1|TSPO|MYH11|AMPD3|ABAT|GATM|ILK|MPST|HAGH|GGT5|GSTP1|ATP5A1|SEPT7|FKBP1A|CDC42|GGT1|TXNRD2|RDX|ACY1|IFI30|FAH|PDIA6|SMS|PTGR1|TST|TF|ALAD|SSB|ALDH7A1|CPVL|IMMT|PCYOX1|SLC25A12|SCRN1|ARPC1B|IDH1|RDH13|TKT|NDUFS1|SLC4A4|AHSG|HIGD1A|CRYZ|PNPT1|NAPRT|GK|ACSF2|HK1|ACTA2|DDC|CKMT2|NDRG1|COX20|C[illegible]	7.59E-33	GO.0009987	2.98507809

425	75	GO Process	purine ribonucleotide metabolic process	1.7E-30	NDUFB4|UQCRC1|OGDH|PDHX|GAPDH|ATP6V1B1|ENO1|PMPCB|NDUFA2|NDUFA10|ATP5B|ATP6V0A1|SDHA|NDUFA9|UQCRC2|PFKL|ATP6V1A|ATP6V1B2|NDUFB9|DLAT|OXSM|ATP5O|ATP5J2|HPRT1|NDUFB8|ATP5L|ATP5H|HINT1|ATP5I|PDHB|CYCS|ATP6V0A4|UQCRH|AQP1|GBAS|ACSS1|CYC1|PIPOX|COX5A|NDUFV1|ACSM2B|NDUFA12|UQCR10|DLST|PKLR|PFKM|STOML2|ATP5C1|MPC1|MT-CO1|OPA1|MT-CO2|ATP1B1|ATP5F1|OGDHL|ALDOB|SUCLA2|ACOT9|PDHA1|PFKP|AK3|NME2|AK4|AMPD3|ATP5A1|NDUFS1|ACSF2|HK1|ABHD14B|ACSL1|COX7C|COX4I1|ACSM2A|GALK1|GLYAT	9.64E-33	GO.0009150	2.97644716
440	76	GO Process	ribonucleotide metabolic process	2.2E-30	NDUFB4|UQCRC1|OGDH|PDHX|GAPDH|ATP6V1B1|ENO1|PMPCB|NDUFA2|NDUFA10|ATP5B|ATP6V0A1|SDHA|NDUFA9|UQCRC2|PFKL|ATP6V1A|ATP6V1B2|NDUFB9|DLAT|OXSM|ATP5O|ATP5J2|HPRT1|NDUFB8|ATP5L|ATP5H|HINT1|ATP5I|PDHB|CYCS|ATP6V0A4|UQCRH|AQP1|GBAS|ACSS1|CYC1|PIPOX|COX5A|NDUFV1|ACSM2B|NDUFA12|UQCR10|DLST|PKLR|PFKM|STOML2|ATP5C1|MPC1|MT-CO1|OPA1|MT-CO2|ATP1B1|ATP5F1|CMPK1|OGDHL|ALDOB|SUCLA2|ACOT9|PDHA1|PFKP|AK3|NME2|AK4|AMPD3|ATP5A1|NDUFS1|ACSF2|HK1|ABHD14B|ACSL1|COX7C|COX4I1|ACSM2A|GALK1|GLYAT	1.25E-32	GO.0009259	2.96655462
455	77	GO Process	ribose phosphate metabolic process	2.6E-30	NDUFB4|UQCRC1|OGDH|PDHX|GAPDH|ATP6V1B1|ENO1|PMPCB|NDUFA2|NDUFA10|ATP5B|ATP6V0A1|SDHA|NDUFA9|UQCRC2|PFKL|ATP6V1A|ATP6V1B2|NDUFB9|DLAT|OXSM|ATP5O|ATP5J2|HPRT1|NDUFB8|ATP5L|ATP5H|HINT1|ATP5I|PDHB|CYCS|ATP6V0A4|UQCRH|AQP1|GBAS|ACSS1|CYC1|PIPOX|COX5A|NDUFV1|ACSM2B|NDUFA12|UQCR10|DLST|PKLR|PFKM|STOML2|ATP5C1|MPC1|MT-CO1|OPA1|MT-CO2|ATP1B1|ATP5F1|CMPK1|OGDHL|ALDOB|SUCLA2|ACOT9|PDHA1|PFKP|AK3|NME2|AK4|AMPD3|ATP5A1|TKT|NDUFS1|ACSF2|HK1|ABHD14B|ACSL1|COX7C|COX4I1|ACSM2A|GALK1|GLYAT	1.58E-32	GO.0019693	2.95783961
209	55	GO Process	alpha-amino acid metabolic process	2.1E-29	GSTZ1|MTHFD1|AHCY|CRYM|AGXT2|GOT2|HRSP12|BHMT2|HIBADH|AMT|BHMT|MUT|HNMT|ACAD8|QDPR|HGD|DDAH1|HPD|FTCD|CKB|GLYATL1|PRODH2|ASL|MSRA|PIPOX|SHMT1|BCAT2|P4HB|DLST|PHGDH|AHCYL1|GOT1|HOGA1|ALDH18A1|SARDH|ASS1|ALDH4A1|AUH|AGMAT|PSAT1|GLDC|DPEP1|NIT2|GATM|MPST|GGT1|FAH|SMS|ALDH7A1|CKMT2|CKMT1A|IVD|PAH|ALDH6A1|GLYAT	1.3E-31	GO.1901605	2.86777807
1146	149	GO Component	organelle envelope	2.6E-49	NDUFB4|UQCRC1|RTCB|OGDH|PPIF|SLC25A11|SLC25A3|GAPDH|GOT2|TRAP1|PMPCB|MTX2|NDUFA2|NDUFA10|LGALS3|MRPL15|SQRDL|LRPPRC|ATP5B|CHCHD3|NNT|SDHA|ACAD11|VDAC1|CPT1A|REXO2|MTFP1|NDUFA9|SORD|UQCRC2|AFG3L2|SOD1|ECSIT|TIMM44|HIGD2A|NDUFB9|MRPL49|SLC25A4|MRPL17|ATP5O|ATP5J2|COX7A1|NDUFB8|LDHD|ATP5L|PRODH2|ATP5H|LETM1|MRPL39|ATP5I|MRPL13|TMEM126A|CYCS|GPD2|C19orf70|UQCRH|AQP1|ACAD9|GBAS|CRAT|CYC1|COX5A|CHDH|TMEM11|CALR|RHOT2|NDUFV1|HADHB|NDUFA12|UQCR10|MRPL12|CYB5A|SLC25A15|SLC25A40|SAMM50|AGK|VAT1|STOML2|ATP5C1|MRPL37|MPC1|CYB5R3|MT-CO1|MRPL21|OPA1|COA3|MT-CO2|MARC2|MGST3|RTN4IP1|ATP5F1|USMG5|SFXN2|MYO6|ALDH18A1|SLC25A5|PMPCA|ASS1|VDAC2|APOOL|CISD1|CLIC1|MAOB|APOO|MPV17|BPHL|HADHA|SLC25A6|FECH|DNM2|BDH1|CNP|SUOX|MVP|MGST1|TSPO|GATM|ATP5A1|IMMT|SLC25A12|SCRN1|RDH13|NDUFS1|HIGD1A|PNPT1|GK|HK1|CKMT2|COX20|CKMT1A|TOMM40|BCS1L|MRPS22|TIMMDC1|ACSL1|NDUFA13|MRPS30|COX7C|ETFDH|VDAC3|IST1|ACADVL|PHB2|TIMM50|SLC25A10|DNM1L|COX4I1|SIRT5|PHB	3.23E-51	GO.0031967	4.85850267

722	138	GO Component	mitochondrial envelope	5.8E-63	NDUFB4|UQCRC1|OGDH|PPIF|SLC25A11|SLC25A3|GOT2|TRAP1|PMPCB|MTX2|NDUFA2|NDUFA10|LGALS3|MRPL15|SQRDL|ATP5B|CHCHD3|NNT|SDHA|ACAD11|VDAC1|CPT1A|REXO2|MTFP1|NDUFA9|SORD|UQCRC2|AFG3L2|SOD1|ECSIT|TIMM44|HIGD2A|NDUFB9|MRPL49|SLC25A4|MRPL17|ATP5O|ATP5J2|COX7A1|NDUFB8|LDHD|ATP5L|PRODH2|ATP5H|LETM1|MRPL39|ATP5I|MRPL13|TMEM126A|CYCS|GPD2|C19orf70|UQCRH|ACAD9|GBAS|CRAT|CYC1|COX5A|CHDH|TMEM11|RHOT2|NDUFV1|HADHB|NDUFA12|UQCR10|MRPL12|CYB5A|SLC25A15|SLC25A40|SAMM50|AGK|VAT1|STOML2|ATP5C1|MRPL37|MPC1|CYB5R3|MT-CO1|MRPL21|OPA1|COA3|MT-CO2|MARC2|RTN4IP1|ATP5F1|USMG5|SFXN2|ALDH18A1|SLC25A5|PMPCA|ASS1|VDAC2|APOOL|CISD1|MAOB|APOO|MPV17|BPHL|HADHA|SLC25A6|FECH|DNM2|BDH1|CNP|SUOX|MGST1|TSPO|GATM|ATP5A1|IMMT|SLC25A12|RDH13|NDUFS1|HIGD1A|PNPT1|GK|HK1|CKMT2|COX20|CKMT1A|TOMM40|BCS1L|MRPS22|TIMMDC1|ACSL1|NDUFA13|MRPS30|COX7C|ETFDH|VDAC3|ACADVL|PHB2|TIMM50|SLC25A10|DNM1L|COX4I1|SIRT5|PHB	3.61E-65	GO.0005740	6.2236572
2318	138	GO Component	vesicle	3.6E-14	RALA|VCL|MAPK1|AHCY|PGRMC1|CTSH|NAGLU|CTSC|MLEC|LTA4H|RAB35|LTF|ATP6V1B1|CTSD|TTR|CANX|ACTN4|NAPSA|LGALS3|GGH|ADAM10|LYZ|PSMD11|ERP29|COTL1|PRDX1|EHD2|CD81|LRP2|PSMD3|ATP6V0A1|F13A1|ENPEP|PRDX5|SORD|SLC27A2|RHCG|PFKL|SOD1|BHMT|IQGAP2|ATP6V1B2|ATP6V0D2|ATP6V0D1|RNPEP|PRDX3|AMN|CCT2|TPP1|HSP90B1|PPIB|ANPEP|PDIA3|CA4|GAA|GLB1|ATP6V0A4|PRKACA|RAB1B|AQP1|AOC3|DAB2|CALR|HSPA5|EHD3|BGN|AP2A2|P4HB|SERPINA5|RAB11B|ACAA1|HSP90AA1|TUBB4B|PRDX6|PTGES2|CTSB|ANXA2|ANXA6|VAT1|RAC1|PIGR|GM2A|DPP4|CYB5R3|PNP|MYO6|GNAS|XPNPEP2|VDAC2|YWHAB|APOOL|RAB14|CLIC4|CLIC1|FLOT1|CUBN|SLC3A2|ACTR2|ESD|TXNDC5|CD9|DNM2|NME2|PRCP|CNP|JUP|NIT2|ACTN1|FLOT2|MVP|MGST1|MYH11|AMPD3|GSTP1|ACY1|PDIA6|TF|ALAD|IDH1|AHSG|NAPRT|DDC|NDRG1|AOC1|SERPINA1|MME|OCIAD2|IST1|ATP1A1|VIM|MGAM|METTL7A|DNM1L|NPC2|ITGB3|ACTG1|PHB|AP2B1	2.02E-15	GO.0031982	1.34412914
679	134	GO Component	mitochondrial membrane	1.6E-62	NDUFB4|UQCRC1|OGDH|PPIF|SLC25A11|SLC25A3|GOT2|TRAP1|PMPCB|MTX2|NDUFA2|NDUFA10|LGALS3|MRPL15|SQRDL|ATP5B|CHCHD3|NNT|SDHA|ACAD11|VDAC1|CPT1A|MTFP1|NDUFA9|SORD|UQCRC2|AFG3L2|ECSIT|TIMM44|HIGD2A|NDUFB9|MRPL49|SLC25A4|MRPL17|ATP5O|ATP5J2|COX7A1|NDUFB8|LDHD|ATP5L|PRODH2|ATP5H|LETM1|MRPL39|ATP5I|MRPL13|TMEM126A|CYCS|GPD2|C19orf70|UQCRH|ACAD9|GBAS|CRAT|CYC1|COX5A|CHDH|TMEM11|RHOT2|NDUFV1|HADHB|NDUFA12|UQCR10|MRPL12|CYB5A|SLC25A15|SLC25A40|SAMM50|AGK|VAT1|STOML2|ATP5C1|MRPL37|MPC1|CYB5R3|MT-CO1|MRPL21|OPA1|COA3|MT-CO2|MARC2|RTN4IP1|ATP5F1|USMG5|SFXN2|ALDH18A1|SLC25A5|PMPCA|ASS1|VDAC2|APOOL|CISD1|MAOB|APOO|MPV17|BPHL|HADHA|SLC25A6|FECH|DNM2|BDH1|CNP|MGST1|TSPO|GATM|ATP5A1|IMMT|SLC25A12|RDH13|NDUFS1|HIGD1A|GK|HK1|CKMT2|COX20|CKMT1A|TOMM40|BCS1L|MRPS22|TIMMDC1|ACSL1|NDUFA13|MRPS30|COX7C|ETFDH|VDAC3|ACADVL|PHB2|TIMM50|SLC25A10|DNM1L|COX4I1|SIRT5|PHB	1.22E-64	GO.0031966	6.18013429
262	59	GO Process	nucleoside monophosphate metabolic process	1.6E-28	NDUFB4|UQCRC1|OGDH|GAPDH|DNPH1|ATP6V1B1|ENO1|PMPCB|NDUFA2|NDUFA10|ATP5B|ATP6V0A1|SDHA|NDUFA9|UQCRC2|PFKL|ATP6V1A|ATP6V1B2|NDUFB9|ATP5O|ATP5J2|HPRT1|NDUFB8|ATP5L|ATP5H|ATP5I|CYCS|ATP6V0A4|UQCRH|GBAS|CYC1|COX5A|SHMT1|NDUFV1|NDUFA12|UQCR10|PKLR|PFKM|STOML2|ATP5C1|MT-CO1|MT-CO2|ATP1B1|LHPP|ATP5F1|CMPK1|OGDHL|ALDOB|DUT|PFKP|AK3|AK4|AMPD3|ATP5A1|NDUFS1|HK1|COX7C|COX4I1|GALK1	1E-30	GO.0009123	2.78013429

190	52	GO Process	ATP metabolic process	1.9E-28	NDUFB4|UQCRC1|OGDH|GAPDH|ATP6V1B1|ENO1|PMPCB|NDUFA2|NDUFA10|ATP5B|ATP6V0A1|SDHA|NDUFA9|UQCRC2|PFKL|ATP6V1A|ATP6V1B2|NDUFB9|ATP5O|ATP5J2|NDUFB8|ATP5L|ATP5H|ATP5I|CYCS|ATP6V0A4|UQCRH|GBAS|CYC1|COX5A|NDUFV1|NDUFA12|UQCR10|PKLR|PFKM|STOML2|ATP5C1|MT-CO1|MT-CO2|ATP1B1|ATP5F1|OGDHL|ALDOB|PFKP|AK4|AMPD3|ATP5A1|NDUFS1|HK1|COX7C|COX4I1|GALK1	1.25E-30	GO.0046034	2.77189666
221	55	GO Process	purine ribonucleoside triphosphate metabolic process	2.2E-28	NDUFB4|UQCRC1|OGDH|GAPDH|ATP6V1B1|ENO1|PMPCB|NDUFA2|NDUFA10|ATP5B|ATP6V0A1|SDHA|NDUFA9|UQCRC2|PFKL|ATP6V1A|ATP6V1B2|NDUFB9|ATP5O|ATP5J2|NDUFB8|ATP5L|ATP5H|ATP5I|CYCS|ATP6V0A4|UQCRH|GBAS|CYC1|COX5A|NDUFV1|NDUFA12|UQCR10|PKLR|PFKM|STOML2|ATP5C1|MT-CO1|OPA1|MT-CO2|ATP1B1|ATP5F1|OGDHL|ALDOB|PFKP|AK3|NME2|AK4|AMPD3|ATP5A1|NDUFS1|HK1|COX7C|COX4I1|GALK1	1.45E-30	GO.0009205	2.76675615
246	57	GO Process	nucleoside triphosphate metabolic process	4.2E-28	NDUFB4|UQCRC1|OGDH|GAPDH|ATP6V1B1|ENO1|PMPCB|NDUFA2|NDUFA10|ATP5B|ATP6V0A1|SDHA|NDUFA9|UQCRC2|PFKL|ATP6V1A|ATP6V1B2|NDUFB9|ATP5O|ATP5J2|NDUFB8|ATP5L|ATP5H|ATP5I|CYCS|ATP6V0A4|UQCRH|GBAS|CYC1|COX5A|NDUFV1|NDUFA12|UQCR10|PKLR|PFKM|STOML2|ATP5C1|MT-CO1|OPA1|MT-CO2|ATP1B1|ATP5F1|CMPK1|OGDHL|ALDOB|DUT|PFKP|AK3|NME2|AK4|AMPD3|ATP5A1|NDUFS1|HK1|COX7C|COX4I1|GALK1	2.93E-30	GO.0009141	2.73757179
297	61	GO Process	coenzyme metabolic process	1E-27	MTHFD1|AHCY|OGDH|PNPO|PDHX|GAPDH|SPR|ENO1|BHMT2|GGH|NNT|PRDX5|NDUFA9|PFKL|ALDH1L1|DLAT|OXSM|QDPR|GPD1L|FTCD|GPD1|SLC23A1|DCXR|PDHB|GPD2|ACSS1|PIPOX|SHMT1|MDH2|ACSM2B|IDH2|DLST|PKLR|CYB5A|PFKM|MPC1|CYB5R3|PNP|GSTO1|AHCYL1|AKR1A1|OGDHL|ALDOB|PCCA|SUCLA2|ACOT9|PDHA1|PFKP|PC|QPRT|IDH1|TKT|NAPRT|ACSF2|HK1|PCCB|ACSL1|ACSM2A|GALK1|FMO1|GLYAT	7.81E-30	GO.0006732	2.69829667
230	55	GO Process	purine ribonucleoside monophosphate metabolic process	1.1E-27	NDUFB4|UQCRC1|OGDH|GAPDH|ATP6V1B1|ENO1|PMPCB|NDUFA2|NDUFA10|ATP5B|ATP6V0A1|SDHA|NDUFA9|UQCRC2|PFKL|ATP6V1A|ATP6V1B2|NDUFB9|ATP5O|ATP5J2|HPRT1|NDUFB8|ATP5L|ATP5H|ATP5I|CYCS|ATP6V0A4|UQCRH|GBAS|CYC1|COX5A|NDUFV1|NDUFA12|UQCR10|PKLR|PFKM|STOML2|ATP5C1|MT-CO1|MT-CO2|ATP1B1|LHPP|ATP5F1|OGDHL|ALDOB|PFKP|AK3|AK4|AMPD3|ATP5A1|NDUFS1|HK1|COX7C|COX4I1|GALK1	8.14E-30	GO.0009167	2.69746941
242	56	GO Process	ribonucleoside monophosphate metabolic process	1.3E-27	NDUFB4|UQCRC1|OGDH|GAPDH|ATP6V1B1|ENO1|PMPCB|NDUFA2|NDUFA10|ATP5B|ATP6V0A1|SDHA|NDUFA9|UQCRC2|PFKL|ATP6V1A|ATP6V1B2|NDUFB9|ATP5O|ATP5J2|HPRT1|NDUFB8|ATP5L|ATP5H|ATP5I|CYCS|ATP6V0A4|UQCRH|GBAS|CYC1|COX5A|NDUFV1|NDUFA12|UQCR10|PKLR|PFKM|STOML2|ATP5C1|MT-CO1|MT-CO2|ATP1B1|LHPP|ATP5F1|CMPK1|OGDHL|ALDOB|PFKP|AK3|AK4|AMPD3|ATP5A1|NDUFS1|HK1|COX7C|COX4I1|GALK1	1.02E-29	GO.0009161	2.68996295
153	46	GO Process	cellular respiration	1.4E-26	NDUFB4|UQCRC1|ACO2|IDH3G|OGDH|PMPCB|NDUFA2|NDUFA10|NNT|SDHA|NDUFA9|UQCRC2|NDUFB9|DLAT|NDUFB8|IDH3A|GPD1|PDHB|CYCS|GPD2|ACO1|UQCRH|CYC1|COX5A|NDUFV1|MDH2|NDUFA12|IDH2|UQCR10|DLST|CS|MT-CO1|MT-CO2|FH|OGDHL|SUCLA2|PDHA1|IDH3B|SLC25A12|IDH1|NDUFS1|COX20|COX7C|ETFDH|ME3|COX4I1	1.13E-28	GO.0045333	2.58664611
343	63	GO Process	sulfur compound metabolic process	2.4E-26	DCN|GSTZ1|MTHFD1|AHCY|OGDH|PDHX|BHMT2|GSTM3|SQRDL|VCAN|LUM|SOD1|NAT8|BHMT|MUT|DLAT|OXSM|HSPA9|GLB1|PDHB|ISCU|MSRA|ACSS1|PIPOX|CNDP2|BGN|ACSM2B|DLST|GSTA1|MPC1|IBA57|MGST3|PHGDH|GSTO1|AHCYL1|AKR1A1|PCCA|SUCLA2|ESD|ACOT9|PDHA1|DPEP1|PC|SUOX|MGST1|MPST|HAGH|GGT5|GSTP1|GGT1|SMS|TST|PCYOX1|IDH1|ACSF2|GPX1|PCCB|ABHD14B|GSTA2|ACSL1|SLC25A10|ACSM2A|GLYAT	2.07E-28	GO.0006790	2.56143937

<table>
<tr><td>424</td><td>69</td><td>GO Process</td><td>mitochondrion organization</td><td>3.3E-26</td><td>NDUFB4|PPIF|PMPCB|MTX2|NDUFA2|NDUFA10|ATP5B|CHCHD3|MTFP1|NDUFA9|UQCRC2|AFG3L2|ECSIT|TIMM44|NDUFB9|SLC25A4|MRPL17|ATP5O|ATP5J2|PRDX3|NDUFB8|ATP5L|ATP5H|LETM1|ATP5I|YWHAG|CYCS|C19orf70|ACAD9|TMEM11|RHOT2|NDUFV1|NDUFA12|UQCR10|HSP90AA1|SAMM50|AGK|STOML2|ATP5C1|LONP1|OPA1|COA3|ATP5F1|SLC25A5|PMPCA|YWHAB|APOOL|APOO|MPV17|PITRM1|SLC25A6|DNM2|CNP|TSPO|ATP5A1|IMMT|NDUFS1|PNPT1|COX20|TOMM40|BCS1L|TIMMDC1|SSBP1|NDUFA13|PHB2|TIMM50|DNM1L|SIRT5|PHB</td><td>2.85E-28</td><td>GO.0007005</td><td>2.54828041</td></tr>
<tr><td>2226</td><td>130</td><td>GO Component</td><td>cytoplasmic vesicle</td><td>1.1E-12</td><td>RALA|VCL|MAPK1|AHCY|PGRMC1|CTSH|CTSC|MLEC|LTA4H|RAB35|LTF|ATP6V1B1|CTSD|TTR|CANX|ACTN4|NAPSA|LGALS3|GGH|ADAM10|LYZ|PSMD11|ERP29|COTL1|PRDX1|EHD2|LRP2|PSMD3|ATP6V0A1|F13A1|ENPEP|PRDX5|SLC27A2|RHCG|PFKL|SOD1|IQGAP2|ATP6V1B2|ATP6V0D2|ATP6V0D1|RNPEP|PRDX3|AMN|CCT2|TPP1|HSP90B1|PPIB|ANPEP|PDIA3|CA4|GAA|GLB1|ATP6V0A4|PRKACA|RAB1B|AOC3|DAB2|CALR|HSPA5|EHD3|BGN|AP2A2|P4HB|SERPINA5|RAB11B|ACAA1|HSP90AA1|TUBB4B|PRDX6|PTGES2|CTSB|ANXA2|ANXA6|VAT1|RAC1|PIGR|GM2A|DPP4|CYB5R3|PNP|MYO6|GNAS|VDAC2|YWHAB|APOOL|RAB14|CLIC4|FLOT1|CUBN|SLC3A2|ACTR2|ESD|TXNDC5|CD9|DNM2|NME2|PRCP|CNP|JUP|NIT2|ACTN1|FLOT2|MVP|MGST1|MYH11|AMPD3|GSTP1|PDIA6|TF|ALAD|IDH1|AHSG|NAPRT|DDC|NDRG1|AOC1|SERPINA1|MME|OCIAD2|IST1|ATP1A1|VIM|MGAM|METTL7A|DNM1L|NPC2|ITGB3|ACTG1|PHB|AP2B1</td><td>6.8E-14</td><td>GO.0031410</td><td>1.1950782</td></tr>
<tr><td>1083</td><td>109</td><td>GO Process</td><td>carbohydrate derivative metabolic process</td><td>7.8E-26</td><td>DCN|NDUFB4|UQCRC1|NANS|AHCY|OGDH|NAGLU|PDHX|GAPDH|ATP6V1B1|ENO1|AKR7A2|PMPCB|NDUFA2|NDUFA10|KHK|ATP5B|ATP6V0A1|SDHA|VCAN|NDUFA9|LUM|SORD|UQCRC2|PFKL|ABHD10|ATP6V1A|ATP6V1B2|DPYS|NDUFB9|DLAT|OXSM|GPD1L|ATP5O|ATP5J2|ALDH1A1|CRYL1|HPRT1|NDUFB8|ATP5L|GPD1|ATP5H|DCXR|HINT1|ATP5I|GLB1|PDHB|CYCS|ATP6V0A4|GPD2|UQCRH|AQP1|GBAS|ACSS1|CYC1|PIPOX|COX5A|SHMT1|UGDH|NDUFV1|BGN|ACSM2B|NDUFA12|UQCR10|DLST|PKLR|PFKM|STOML2|ATP5C1|GM2A|ITIH2|MPC1|MT-CO1|PNP|OPA1|MT-CO2|ATP1B1|ATP5F1|AHCYL1|CMPK1|AKR1A1|OGDHL|HSPG2|ALDOB|SUCLA2|ACOT9|PDHA1|AKR1C3|DUT|PFKP|AK3|NME2|DAK|AK4|AMPD3|ATP5A1|TKT|NDUFS1|GK|ACSF2|HK1|FBP1|ABHD14B|ACSL1|COX7C|COX4I1|ACSM2A|GALK1|GLYAT</td><td>6.94E-28</td><td>GO.1901135</td><td>2.5107349</td></tr>
<tr><td>217</td><td>51</td><td>GO Process</td><td>energy derivation by oxidation of organic compounds</td><td>2.3E-25</td><td>NDUFB4|UQCRC1|ACO2|IDH3G|OGDH|PMPCB|NDUFA2|NDUFA10|NNT|SDHA|NDUFA9|UQCRC2|NDUFB9|DLAT|NDUFB8|IDH3A|GPD1|GAA|PDHB|CYCS|GPD2|ACO1|UQCRH|CYC1|COX5A|NDUFV1|MDH2|NDUFA12|IDH2|UQCR10|DLST|CS|PFKM|MT-CO1|MT-CO2|FH|GNAS|OGDHL|SUCLA2|PDHA1|IDH3B|SLC25A12|IDH1|NDUFS1|COX20|GBE1|COX7C|ETFDH|ACADVL|ME3|COX4I1</td><td>2.09E-27</td><td>GO.0015980</td><td>2.4636388</td></tr>
<tr><td>1370</td><td>121</td><td>GO Process</td><td>organonitrogen compound biosynthetic process</td><td>2.8E-24</td><td>DCN|GSTZ1|MTHFD1|AHCY|OGDH|PNPO|PDHX|GAPDH|AGXT2|SPR|ENO1|GOT2|BHMT2|GSTM3|CARS2|MRPL15|ATP5B|BBOX1|ATP6V0A1|VCAN|EIF4H|LUM|PFKL|BHMT|MRPL49|DLAT|QDPR|MRPL17|ATP5O|ATP5J2|BDH2|HPRT1|ATP5L|ATP5H|MRPL39|ATP5I|MRPL13|ASL|PDHB|ATP6V0A4|AQP1|GBAS|TSFM|ACSS1|CYC1|SHMT1|UGDH|CHDH|TUFM|BCAT2|CNDP2|BGN|IDH2|MRPL12|TMLHE|GSTA1|PKLR|RPS2|SLC25A15|PPA2|PFKM|ALDH3A2|ALDH9A1|AGK|STOML2|ATP5C1|MRPL37|MPC1|PNP|MRPL21|DARS2|IBA57|IARS2|MGST3|PHGDH|GSTO1|ATP5F1|GOT1|ALDH18A1|CMPK1|AKR1A1|ASS1|OGDHL|HSPG2|ALDOB|VARS|AGMAT|PSAT1|SUCLA2|ESD|PDHA1|DUT|PFKP|FECH|NME2|QPRT|MGST1|TSPO|AMPD3|ABAT|GATM|HAGH|GGT5|GSTP1|ATP5A1|GGT1|SMS|ALAD|ALDH7A1|NAPRT|HK1|DDC|LARS2|MRPS22|GFM1|GSTA2|ACSL1|MRPS30|PAH|GALK1|SARS2</td><td>2.61E-26</td><td>GO.1901566</td><td>2.35497509</td></tr>
</table>

169	45	GO Process	electron transport chain	3.5E-24	NDUFB4|UQCRC1|AKR7A2|PMPCB|NDUFA2|NDUFA10|DMGDH|ALDH2|SDHA|NDUFA9|NDUFB9|QDPR|COX7A1|ADH5|NDUFB8|GPD1|CYCS|GPD2|UQCRH|CYC1|COX5A|UGDH|NDUFV1|NDUFA12|UQCR10|CYB5A|PTGES2|MT-CO1|MT-CO2|AKR7A3|PHGDH|AKR1A1|ALDH4A1|MAOB|GLRX|NQO2|IDH3B|GLDC|TXNRD2|SLC25A12|NDUFS1|COX7C|ETFDH|ETFA|COX4I1	3.3E-26	GO.0022900	2.34571746
131	40	KEGG Pathways	Oxidative phosphorylation	2.6E-23	NDUFB4|UQCRC1|ATP6V1B1|NDUFA2|NDUFA10|ATP5B|ATP6V0A1|SDHA|NDUFA9|UQCRC2|ATP6V1A|ATP6V1B2|NDUFB9|ATP6V0D2|ATP5O|ATP6V0D1|ATP5J2|COX7A1|NDUFB8|ATP5L|ATP5H|ATP5I|ATP6V0A4|UQCRH|CYC1|COX5A|NDUFV1|NDUFA12|UQCR10|PPA2|ATP5C1|MT-CO1|MT-CO2|LHPP|ATP5F1|ATP5A1|NDUFS1|NDUFA13|COX7C|COX4I1	2.8E-25	hsa00190	2.25783961
2097	153	GO Function	nucleotide binding	4.6E-23	RALA|MAPK1|RTCB|MYH9|MTHFD1|PCK2|AHCY|IDH3G|CRYM|DECR1|PNPO|PFN1|GAPDH|RAB35|ATP6V1B1|SPR|ACADS|TRAP1|TUBA4A|CARS2|TINAG|KHK|PEBP1|ALDH2|ATP5B|EHD2|NNT|SDHA|ACAD11|HIBADH|SORD|SLC27A2|AFG3L2|PFKL|TIMM44|ATP6V1A|ATP6V1B2|CCT5|ACAD8|QDPR|GPD1L|ACTC1|BDH2|HSPA9|ALDH1A1|CRYL1|HPRT1|CKB|CCT2|IDH3A|HSP90B1|LDHD|TUBA1C|GPD1|PRODH2|HINT1|MRPL39|PRKACA|RAB1B|ACAD9|GRHPR|ACSS1|UGDH|PCK1|CHDH|RHOT2|TUFM|NDUFV1|HSPA5|EHD3|ACSM2B|IDH2|RAB11B|HSP90AA1|PKLR|TUBB4B|GNAI1|PFKM|ANXA6|AGK|RAC1|MYH10|LONP1|CYB5R3|OPA1|DARS2|TGM2|IARS2|PHGDH|MYO6|ABCD3|GNAS|ALDH18A1|SCP2|CMPK1|ASS1|VDAC2|RAB14|VARS|PCCA|ALDH1B1|ACTR2|MAOB|SUCLA2|NQO2|HADHA|IDH3B|PFKP|AK3|DNM2|NME2|CNP|PC|DAK|AK4|MYH11|ILK|ATP5A1|SEPT7|CDC42|TXNRD2|IDH1|CRYZ|GK|ACSF2|HK1|ACTA2|CKMT2|CKMT1A|FBP1|LARS2|BCS1L|IVD|PCCB|GFM1|ACSL1|NDUFA13|ETFDH|VDAC3|ACADVL|ME3|ATP1A1|DNM1L|ALDH6A1|ETFA|ACTG1|ACSM2A|GALK1|TTN|SARS2|SIRT5|FMO1|HAO2	2.26E-25	GO.0000166	2.23400838
124	39	GO Process	antibiotic metabolic process	4.7E-23	ACO2|IDH3G|OGDH|AKR7A2|ALDH2|PRDX1|NNT|SDHA|PRDX5|SOD1|DLAT|FTCD|ADH5|ALDH1A1|PRDX3|IDH3A|ADH1B|PDHB|ACO1|ACSS1|MDH2|IDH2|DLST|PRDX6|CS|FH|OGDHL|ALDH1B1|MAOB|SUCLA2|ESD|PDHA1|AKR1C3|IDH3B|GPX3|DPEP1|IDH1|GPX1|ME3	4.55E-25	GO.0016999	2.2326058

4130	233	GO Process	transport	5.8E-23	RALA|VCL|MAPK1|MYH9|PGRMC1|F9|CRYM|CTSH|PPIF|SLC25A11|VTN|CTSC|SLC25A3|MLEC|LTA4H|RAB35|LTF|EHHADH|ATP6V1B1|CTSD|TTR|GOT2|CANX|TUBA4A|VIL1|HSPB1|PMPCB|MTX2|ACTN4|LGALS3|TINAG|GGH|ADAM10|LRPPRC|LYZ|PSMD11|ERP29|ATP5B|COTL1|SLC9A3R1|EHD2|NAPA|CD81|LRP2|PSMD3|ATP6V0A1|NNT|ADD1|F13A1|AMBP|VDAC1|CPT1A|NDUFA9|SLC27A2|RHCG|UQCRC2|AFG3L2|PFKL|SOD1|TIMM44|TINAGL1|ATP6V1A|IQGAP2|ATP6V1B2|ERLIN2|CCT5|SLC25A4|UCHL1|ATP6V0D2|ATP5O|ATP6V0D1|ATP5J2|COX7A1|FABP1|SCIN|HSPA9|AMN|CCT2|HSP90B1|ANPEP|ATP5L|CA4|TUBA1C|ATP5H|SLC23A1|LETM1|GAA|ATP5I|YWHAG|GLB1|ATP6V0A4|ACO1|DPYSL2|RAB1B|AQP1|DAB2|CRAT|TLN1|CYC1|PIPOX|COX5A|SLC7A8|CALR|RHOT2|HSPA5|DHRS4|EHD3|AP2A2|SLC5A2|SERPINA5|RAB11B|ACAA1|HSP90AA1|PLS1|TUBB4B|CYB5A|RPS2|PRDX6|SLC25A15|SLC25A40|PTGES2|SAMM50|CTSB|ANXA2|ANXA6|AGK|VAT1|RAC1|STOML2|PIGR|ATP5C1|GM2A|MYH10|MPC1|CYB5R3|MT-CO1|PNP|OPA1|MT-CO2|TGM2|ATP1B1|S100A10|ATP5F1|AHCYL1|FLNA|SFXN2|MYO6|ABCD3|GNAS|RBP4|SCP2|SLC25A5|PMPCA|VDAC2|YWHAB|APOOL|RAB14|CLIC4|HSPG2|CLIC1|FLOT1|CUBN|SLC3A2|ACTR2|APOO|TXNDC5|MPV17|HBD|AKR1C3|PITRM1|SLC25A6|AMACR|CD9|KRT18|DNM2|NME2|PRCP|CNP|JUP|NIT2|ACTN1|MVP|MGST1|TSPO|AMPD3|GSTP1|ATP5A1|FKBP1A|CDC42|PDIA6|TST|TF|ALAD|SSB|PCYOX1|SLC25A12|SCRN1|ARPC1B|IDH1|SLC4A4|AHSG|PDZK1|SELENBP1|PNPT1|NAPRT|DDC|TOMM40|AOC1|SERPINA1|MME|ACSL1|NDUFA13|COX7C|VDAC3|EPHX2|IST1|PHB2|ATP1A1|TIMM50|SLC25A10|MGAM|METTL7A|DNM1L|NPC2|ITGB3|COX4I1|ACTG1|RBM8A|TTN|PHB|PLCG2|AP2B1|HAO2	5.69E-25	GO.0006810	2.22380722
185	45	GO Process	cellular modified amino acid metabolic process	8E-23	GSTZ1|MTHFD1|AHCY|CRYM|GOT2|DMGDH|BHMT2|GSTM3|GGH|BBOX1|CPT1A|SOD1|NAT8|ALDH1L1|BHMT|FTCD|CKB|CRAT|PIPOX|SHMT1|CHDH|CNDP2|P4HB|TMLHE|GSTA1|ALDH9A1|GSTO1|AHCYL1|HOGA1|SARDH|ASS1|ALDH4A1|DPEP1|GATM|HAGH|GGT5|GSTP1|GGT1|ALDH7A1|PCYOX1|IDH1|CKMT2|CKMT1A|GPX1|GSTA2	8.05E-25	GO.0006575	2.20958256
71	32	GO Process	aerobic respiration	1.2E-22	UQCRC1|ACO2|IDH3G|OGDH|PMPCB|NNT|SDHA|UQCRC2|DLAT|IDH3A|PDHB|CYCS|ACO1|UQCRH|COX5A|MDH2|IDH2|UQCR10|DLST|CS|MT-CO1|MT-CO2|FH|OGDHL|SUCLA2|PDHA1|IDH3B|IDH1|COX20|COX7C|ME3|COX4I1	1.2E-24	GO.0009060	2.1928118

<table>
<tr><td>8808</td><td>383</td><td>GO Process</td><td>primary metabolic process</td><td>1.9E-22</td><td>DCN|NDUFB4|UQCRC1|NANS|MAPK1|LGALS1|RTCB|MYH9|ACO2|GSTZ1|MTHFD1|PCK2|AHCY|IDH3G|F9|CRYM|CTSH|DECR1|OGDH|PPIF|SLC25A11|C1QBP|CTSC|PDHX|MLEC|LTA4H|GAPDH|DNPH1|AGXT2|SKP1|LTF|EHHADH|ATP6V1B1|ENO1|AKR7A2|CTSD|TTR|GDA|ACADS|PEPD|GOT2|PMPCB|NDUFA2|NDUFA10|NAPSA|LGALS3|GRSF1|HRSP12|APCS|BHMT2|CARS2|LAMC1|TINAG|MRPL15|GGH|ADAM10|KHK|LRPPRC|LYZ|PEBP1|PSMD11|ALDH2|ATP5B|CHCHD3|MECR|LRP2|PSMD3|ATP6V0A1|NNT|ADD1|F13A1|SDHA|ACAD11|VCAN|AMBP|ENPEP|HIBADH|CALB1|PRDX5|ALDH8A1|CPT1A|EIF4H|REXO2|NDUFA9|SORD|SLC27A2|UQCRC2|AFG3L2|PFKL|BCKDHA|TINAGL1|NAT8|ABHD10|ATP6V1A|AMT|BHMT|MUT|ATP6V1B2|ERLIN2|LACTB2|DPYS|NDUFB9|MRPL49|HNMT|DLAT|OXSM|ACAD8|QDPR|GPD1L|HGD|DDAH1|UCHL1|MRPL17|HPD|ATP5O|FTCD|ATP5J2|CAPN2|RNPEP|FABP1|ADH5|BDH2|ALDH1A1|CRYL1|PRDX3|HPRT1|NDUFB8|CKB|TPP1|IDH3A|HSP90B1|PPIB|ANPEP|GLYATL1|PDIA3|ATP5L|GPD1|PRODH2|ATP5H|ECI1|SLC23A1|DCXR|HINT1|UGT2B7|MRPL39|GAA|ATP5I|MRPL13|GLB1|ASL|PDHB|CYCS|ATP6V0A4|GPD2|ACO1|DPYSL2|UQCRH|PRKACA|RAB1B|ISCU|CYP4A11|AQP1|ACAD9|GBAS|TSFM|MSRA|CRAT|ACSS1|CYC1|PIPOX|COX5A|CES2|BCKDHB|SHMT1|UGDH|PCK1|SLC7A8|UGT2B17|CALR|TUFM|NDUFV1|BCAT2|HSPA5|HADHB|FBN1|CNDP2|DHRS4|MDH2|BGN|ACSM2B|AP2A2|P4HB|SLC5A2|NDUFA12|IDH2|UQCR10|RAB11B|ACAA1|MRPL12|HSP90AA1|DLST|GSTA1|PKLR|CYB5A|RPS2|PRDX6|CS|TPSAB1|PPA2|PTGES2|CTSB|PFKM|ALDH3A2|ANXA2|GPX4|UGT1A9|AGK|RAC1|STOML2|ATP5C1|GM2A|ITIH2|COL4A2|DPP3|DPP4|LONP1|MRPL37|MPC1|CYB5R3|MT-CO1|PNP|MRPL21|OPA1|MT-CO2|DARS2|TGM2|FH|IARS2|C4BPA|ATP1B1|MGST3|ECHS1|LHPP|HMGCS2|PHGDH|GSTO1|ATP5F1|AHCYL1|FLNA|ABCD3|GOT1|HOGA1|GNAS|XPNPEP2|ALDH18A1|RBP4|SCP2|ECHDC2|PMPCA|SARDH|CMPK1|AKR1A1|ASS1|YWHAB|RAB14|OGDHL|HSPG2|ALDOB|ALDH4A1|VARS|AUH|AGMAT|PSAT1|ALDH1B1|CUBN|SLC3A2|SUCLA2|ACOT9|PDHA1|GLRX|BPHL|AKR1C3|HADHA|IDH3B|DUT|PITRM1|PFKP|GLDC|AK3|AMACR|SACM1L|DPEP1|NME2|PRCP|CNP|PC|NIT2|DAK|AK4|QPRT|PDP1|TSPO|AMPD3|ABAT|GATM|ILK|MPST|GGT5|GSTP1|ATP5A1|FKBP1A|CDC42|GGT1|ACY1|FAH|PDIA6|SMS|PTGR1|TST|TF|SSB|ALDH7A1|CPVL|PCYOX1|SLC25A12|SCRN1|IDH1|RDH13|TKT|NDUFS1|AHSG|PNPT1|NAPRT|GK|ACSF2|HK1|DDC|CKMT2|CKMT1A|GPX1|FBP1|LARS2|GBE1|SERPINA1|TGFBI|VWA1|MRPS22|IVD|MME|PCCB|GFM1|SSBP1|ABHD14B|ACSL1|MRPS30|COX7C|ETFDH|EPHX2|CRYAB|ECHDC1|ACADVL|ME3|PHB2|TIMM50|SLC25A10|MGAM|PAH|ALDH6A1|NPC2|ETFA|COX4I1|ACSM2A|RBM8A|GALK1|TTN|SARS2|SIRT5|PHB|FMO1|PLCG2|AP2B1|HAO2|GLYAT</td><td>1.95E-24</td><td>GO.0044238</td><td>2.17281584</td></tr>
<tr><td>114</td><td>37</td><td>GO Process</td><td>cellular amino acid catabolic process</td><td>2.9E-22</td><td>GSTZ1|AHCY|CRYM|AGXT2|GOT2|HRSP12|HIBADH|BCKDHA|AMT|HNMT|ACAD8|QDPR|HGD|DDAH1|HPD|FTCD|PRODH2|PIPOX|BCKDHB|SHMT1|BCAT2|DLST|GOT1|HOGA1|SARDH|ALDH4A1|AUH|GLDC|ABAT|MPST|FAH|TST|ALDH7A1|PCYOX1|IVD|PAH|ALDH6A1</td><td>3.09E-24</td><td>GO.0009063</td><td>2.15346171</td></tr>
</table>

4248	235	GO Process	establishment of localization	4.1E-22	RALA|VCL|MAPK1|MYH9|PGRMC1|F9|CRYM|CTSH|PPIF|SLC25A11|VTN|CTSC|SLC25A3|MLEC|LTA4H|RAB35|LTF|EHHADH|ATP6V1B1|CTSD|TTR|GOT2|CANX|TUBA4A|VIL1|HSPB1|PMPCB|MTX2|ACTN4|LGALS3|TINAG|GGH|ADAM10|LRPPRC|LYZ|PSMD11|ERP29|ATP5B|COTL1|SLC9A3R1|EHD2|NAPA|CD81|LRP2|PSMD3|ATP6V0A1|NNT|ADD1|F13A1|AMBP|VDAC1|CPT1A|NDUFA9|SLC27A2|RHCG|UQCRC2|AFG3L2|PFKL|SOD1|TIMM44|TINAGL1|ATP6V1A|IQGAP2|ATP6V1B2|ERLIN2|CCT5|SLC25A4|UCHL1|ATP6V0D2|ATP5O|ATP6V0D1|ATP5J2|COX7A1|FABP1|SCIN|HSPA9|AMN|CCT2|HSP90B1|ANPEP|ATP5L|CA4|TUBA1C|ATP5H|SLC23A1|LETM1|GAA|ATP5I|YWHAG|GLB1|ATP6V0A4|ACO1|DPYSL2|RAB1B|AQP1|DAB2|CRAT|TLN1|CYC1|PIPOX|COX5A|SLC7A8|CALR|RHOT2|HSPA5|DHRS4|EHD3|AP2A2|SLC5A2|SERPINA5|RAB11B|ACAA1|HSP90AA1|PLS1|TUBB4B|CYB5A|RPS2|PRDX6|SLC25A15|SLC25A40|PTGES2|SAMM50|CTSB|ANXA2|ANXA6|AGK|VAT1|RAC1|STOML2|PIGR|ATP5C1|GM2A|MYH10|MPC1|CYB5R3|MT-CO1|PNP|OPA1|MT-CO2|TGM2|ATP1B1|S100A10|ATP5F1|AHCYL1|FLNA|SFXN2|MYO6|ABCD3|GNAS|RBP4|SCP2|SLC25A5|PMPCA|VDAC2|YWHAB|APOOL|RAB14|CLIC4|HSPG2|CLIC1|FLOT1|CUBN|SLC3A2|ACTR2|APOO|TXNDC5|MPV17|HBD|AKR1C3|PITRM1|SLC25A6|AMACR|CD9|KRT18|DNM2|NME2|PRCP|CNP|JUP|NIT2|ACTN1|MVP|MGST1|TSPO|AMPD3|GSTP1|ATP5A1|FKBP1A|CDC42|RDX|PDIA6|TST|TF|ALAD|SSB|PCYOX1|SLC25A12|SCRN1|ARPC1B|IDH1|SLC4A4|AHSG|PDZK1|SELENBP1|PNPT1|NAPRT|HK1|DDC|TOMM40|AOC1|SERPINA1|MME|ACSL1|NDUFA13|COX7C|VDAC3|EPHX2|IST1|PHB2|ATP1A1|TIMM50|SLC25A10|MGAM|METTL7A|DNM1L|NPC2|ITGB3|COX4I1|ACTG1|RBM8A|TTN|PHB|PLCG2|AP2B1|HAO2	4.43E-24	GO.0051234	2.13872161
198	45	GO Process	monosaccharide metabolic process	8E-22	PCK2|SLC25A11|GAPDH|ENO1|GOT2|KHK|CPT1A|SORD|PFKL|ABHD10|DLAT|ALDH1A1|CRYL1|GPD1|SLC23A1|DCXR|UGT2B7|GAA|GLB1|PDHB|GPD2|PCK1|UGT2B17|FBN1|MDH2|PKLR|CYB5A|PFKM|UGT1A9|CYB5R3|GSTO1|GOT1|RBP4|AKR1A1|ALDOB|PDHA1|PFKP|PC|DAK|SLC25A12|TKT|HK1|FBP1|SLC25A10|GALK1	8.82E-24	GO.0005996	2.10958256
142	39	KEGG Pathways	Parkinson's disease	2E-21	NDUFB4|UQCRC1|PPIF|NDUFA2|NDUFA10|ATP5B|SDHA|VDAC1|NDUFA9|UQCRC2|NDUFB9|SLC25A4|UCHL1|ATP5O|COX7A1|NDUFB8|ATP5H|CYCS|UQCRH|PRKACA|CYC1|COX5A|NDUFV1|NDUFA12|UQCR10|GNAI1|ATP5C1|MT-CO1|MT-CO2|ATP5F1|SLC25A5|VDAC2|SLC25A6|ATP5A1|NDUFS1|NDUFA13|COX7C|VDAC3|COX4I1	2.86E-23	hsa05012	2.0692504
106	35	GO Process	monocarboxylic acid catabolic process	3.1E-21	DECR1|AGXT2|EHHADH|ACADS|MECR|ACAD11|CPT1A|SORD|SLC27A2|BDH2|CRYL1|ECI1|DCXR|CYP4A11|CRAT|HADHB|ACAA1|ALDH3A2|ECHS1|ABCD3|HOGA1|SCP2|ECHDC2|AKR1A1|AUH|HADHA|AMACR|ABAT|IVD|PCCB|ETFDH|ECHDC1|ACADVL|ETFA|HAO2	3.41E-23	GO.0072329	2.05157002
457	64	GO Process	carbohydrate metabolic process	3.8E-21	NANS|PCK2|IDH3G|OGDH|SLC25A11|MLEC|GAPDH|ENO1|AKR7A2|GOT2|KHK|ALDH2|CPT1A|SORD|PFKL|ABHD10|DLAT|GPD1L|ALDH1A1|CRYL1|IDH3A|GPD1|SLC23A1|DCXR|UGT2B7|GAA|GLB1|PDHB|GPD2|PCK1|UGT2B17|FBN1|MDH2|SLC5A2|IDH2|PKLR|CYB5A|CS|PFKM|UGT1A9|GM2A|CYB5R3|GSTO1|GOT1|RBP4|AKR1A1|OGDHL|ALDOB|ALDH1B1|SLC3A2|PDHA1|PFKP|PC|DAK|SLC25A12|IDH1|TKT|GK|HK1|FBP1|GBE1|SLC25A10|MGAM|GALK1	4.3E-23	GO.0005975	2.04236586

463	124	GO Component	mitochondrial matrix	2.5E-70	OXCT1|ACO2|GSTZ1|PCK2|IDH3G|DECR1|OGDH|PPIF|C1QBP|PDHX|AGXT2|ACADS|GOT2|TRAP1|PMPCB|NDUFA10|GRSF1|HRSP12|DMGDH|CARS2|MRPL15|LRPPRC|ALDH2|ATP5B|MECR|VDAC1|HIBADH|PRDX5|REXO2|NDUFA9|BCKDHA|SOD1|TIMM44|ABHD10|AMT|MUT|LACTB2|MRPL49|DLAT|ACAD8|MRPL17|HSPA9|PRDX3|NDUFB8|IDH3A|ECI1|MRPL39|MRPL13|PDHB|ISCU|TSFM|ACSS1|BCKDHB|TUFM|BCAT2|HADHB|MDH2|ACSM2B|IDH2|MRPL12|TMLHE|DLST|CS|PPA2|ATP5C1|LONP1|MRPL37|MRPL21|DARS2|FH|IBA57|IARS2|ECHS1|HMGCS2|ATP5F1|HOGA1|SLC25A5|PMPCA|SARDH|VDAC2|OGDHL|ALDH4A1|AUH|PCCA|ALDH1B1|SUCLA2|ACOT9|PDHA1|HADHA|IDH3B|PITRM1|GLDC|AK3|FECH|BDH1|PC|SUOX|AK4|PDP1|ABAT|MPST|HAGH|ATP5A1|TXNRD2|TST|ALDH7A1|NDUFS1|ACSF2|GPX1|LARS2|MRPS22|IVD|PCCB|GFM1|SSBP1|ETFDH|ACADVL|ME3|ALDH6A1|ETFA|ACSM2A|SARS2|SIRT5|GLYAT	1.17E-72	GO.0005759	6.95985995
311	53	GO Process	carboxylic acid biosynthetic process	9.4E-21	DCN|MTHFD1|AHCY|OGDH|LTA4H|GAPDH|AGXT2|ENO1|GOT2|BHMT2|MECR|VCAN|ALDH8A1|SLC27A2|PFKL|BHMT|OXSM|ASL|ACSS1|SHMT1|UGDH|BCAT2|BGN|ACSM2B|PKLR|PTGES2|PFKM|GPX4|MGST3|PHGDH|ABCD3|GOT1|HOGA1|ALDH18A1|SCP2|AKR1A1|ASS1|OGDHL|ALDOB|PSAT1|SUCLA2|AKR1C3|PFKP|AMACR|ABAT|GATM|GGT5|GGT1|HK1|EPHX2|PAH|ACSM2A|GALK1	1.09E-22	GO.0046394	2.00264104
103	34	GO Function	electron transfer activity	2.2E-20	UQCRC1|AKR7A2|DMGDH|ALDH2|SDHA|QDPR|COX7A1|ADH5|CYCS|UQCRH|CYC1|COX5A|UGDH|NDUFA12|UQCR10|CYB5A|PTGES2|MT-CO1|MT-CO2|AKR7A3|PHGDH|AKR1A1|ALDH4A1|MAOB|GLRX|NQO2|IDH3B|GLDC|TXNRD2|NDUFS1|COX7C|ETFDH|ETFA|COX4I1	1.46E-22	GO.0009055	1.96556077
35	8	KEGG Pathways	Alanine, aspartate and glutamate metabolism	0.00015	AGXT2|GOT2|ASL|GOT1|ASS1|ALDH4A1|NIT2|ABAT	2.59E-05	hsa00250	0.38239087
223	45	GO Process	mitochondrial transport	4.8E-20	PPIF|SLC25A11|SLC25A3|PMPCB|MTX2|ATP5B|CPT1A|UQCRC2|AFG3L2|TIMM44|SLC25A4|ATP5O|ATP5J2|ATP5L|ATP5H|LETM1|ATP5I|YWHAG|CYC1|RHOT2|HSP90AA1|SLC25A15|SLC25A40|SAMM50|AGK|STOML2|ATP5C1|MPC1|OPA1|ATP5F1|SLC25A5|PMPCA|YWHAB|PITRM1|SLC25A6|CNP|TSPO|ATP5A1|TST|SLC25A12|PNPT1|TOMM40|NDUFA13|TIMM50|SLC25A10	5.75E-22	GO.0006839	1.93178549
100	33	GO Process	oxidative phosphorylation	5.1E-20	NDUFB4|UQCRC1|PMPCB|NDUFA2|NDUFA10|ATP5B|SDHA|NDUFA9|UQCRC2|NDUFB9|ATP5O|ATP5J2|NDUFB8|ATP5L|ATP5H|ATP5I|CYCS|UQCRH|GBAS|CYC1|COX5A|NDUFV1|NDUFA12|UQCR10|STOML2|ATP5C1|MT-CO1|MT-CO2|ATP5F1|ATP5A1|NDUFS1|COX7C|COX4I1	6.25E-22	GO.0006119	1.92890369
135	37	GO Function	oxidoreductase activity, acting on CH-OH group of donors	5.6E-20	IDH3G|EHHADH|SPR|AKR7A2|HIBADH|SORD|GPD1L|ADH5|BDH2|CRYL1|IDH3A|LDHD|GPD1|DCXR|ADH1B|GPD2|GRHPR|UGDH|CHDH|HADHB|DHRS4|MDH2|IDH2|CYB5A|ALDH3A2|AKR7A3|PHGDH|AKR1A1|AKR1C3|HADHA|IDH3B|BDH1|PTGR1|IDH1|RDH13|ME3|HAO2	4.14E-22	GO.0016614	1.92533658

<table>
<tr><td>3559</td><td>202</td><td>GO Process</td><td>regulation of biological quality</td><td>2.6E-19</td><td>DCN|OXCT1|MAPK1|LGALS1|MYH9|MTHFD1|F9|CRYM|CTSH|PPIF|PFN1|C1QBP|VTN|GAPDH|AGXT2|SKP1|LTF|ATP6V1B1|SPR|TTR|GOT2|DSTN|VIL1|HSPB1|NAPSA|HRSP12|DMGDH|ATP5B|SLC9A3R1|PRDX1|EHD2|NAPA|CD81|ATP6V0A1|NNT|ADD1|F13A1|ENPEP|CALB1|PRDX5|ALDH8A1|CPT1A|RHCG|AFG3L2|PFKL|SOD1|ATP6V1A|AMT|BHMT|IQGAP2|DPYS|HNMT|CCT5|SLC25A4|GPD1L|DDAH1|ATP6V0D2|ATP6V0D1|RNPEP|ADH5|BDH2|SCIN|HSPA9|COL1A2|ALDH1A1|PRDX3|CKB|CCT2|TPP1|HSP90B1|PPIB|ANPEP|PDIA3|UGT2B7|LETM1|GAA|YWHAG|ATP6V0A4|ACO1|DPYSL2|PRKACA|ISCU|CYP4A11|AQP1|TLN1|SHMT1|PCK1|CHDH|SLC7A8|CALR|RHOT2|BCAT2|HSPA5|FBN1|DHRS4|EHD3|P4HB|SERPINA5|RAB11B|PARVA|HSP90AA1|PLS1|PRDX6|EPB41L3|PPA2|PTGES2|PFKM|ANXA2|ANXA6|UGT1A9|ALDH9A1|RAC1|STOML2|GM2A|PBLD|MSN|MYH10|DPP4|OPA1|MT-CO2|TGM2|FH|IARS2|ATP1B1|PHGDH|GSTO1|FLNA|SFXN2|GOT1|GNAS|RBP4|SCP2|SLC25A5|SARDH|ASS1|YWHAB|CLIC4|VARS|CLIC1|FLOT1|CUBN|ACTR2|MAOB|POSTN|TXNDC5|GLRX|MPV17|HBD|AKR1C3|GLDC|SLC25A6|AK3|CD9|DNM2|PRCP|CNP|CORO1B|JUP|FHL1|FLOT2|UMOD|TSPO|AMPD3|ABAT|ILK|GSTP1|SEPT7|FKBP1A|CDC42|TXNRD2|RDX|PDIA6|TF|ALDH7A1|IMMT|ARPC1B|RDH13|NDUFS1|SLC4A4|HK1|ACTA2|DDC|GPX1|LARS2|SERPINA1|MME|EPHX2|CRYAB|IST1|ACADVL|PHB2|ATP1A1|VIM|PAH|DNM1L|NPC2|ITGB3|ACSM2A|PHB|PDLIM5|PLCG2|GLYAT</td><td>3.17E-21</td><td>GO.0065008</td><td>1.85900669</td></tr>
<tr><td>193</td><td>41</td><td>KEGG Pathways</td><td>Huntington's disease</td><td>3.8E-19</td><td>NDUFB4|UQCRC1|PPIF|NDUFA2|NDUFA10|ATP5B|SDHA|VDAC1|NDUFA9|UQCRC2|SOD1|NDUFB9|SLC25A4|ATP5O|COX7A1|NDUFB8|ATP5H|CYCS|UQCRH|CYC1|COX5A|NDUFV1|AP2A2|NDUFA12|UQCR10|ATP5C1|MT-CO1|MT-CO2|TGM2|ATP5F1|SLC25A5|VDAC2|SLC25A6|ATP5A1|NDUFS1|GPX1|NDUFA13|COX7C|VDAC3|COX4I1|AP2B1</td><td>7.99E-21</td><td>hsa05016</td><td>1.84236586</td></tr>
<tr><td>691</td><td>75</td><td>GO Process</td><td>regulated exocytosis</td><td>5.4E-19</td><td>VCL|MAPK1|PGRMC1|CTSH|CTSC|MLEC|LTA4H|LTF|CTSD|TTR|TUBA4A|ACTN4|LGALS3|GGH|ADAM10|LYZ|PSMD11|COTL1|PSMD3|ATP6V0A1|F13A1|SLC27A2|PFKL|SOD1|IQGAP2|SCIN|CCT2|ANPEP|GAA|GLB1|TLN1|AP2A2|RAB11B|ACAA1|HSP90AA1|TUBB4B|PRDX6|PTGES2|CTSB|ANXA2|VAT1|RAC1|PIGR|GM2A|CYB5R3|PNP|FLNA|APOOL|RAB14|ACTR2|TXNDC5|CD9|NME2|PRCP|JUP|NIT2|ACTN1|MVP|MGST1|AMPD3|GSTP1|TF|ALAD|IDH1|AHSG|NAPRT|AOC1|SERPINA1|MME|IST1|MGAM|METTL7A|NPC2|ITGB3|TTN</td><td>6.78E-21</td><td>GO.0045055</td><td>1.82676062</td></tr>
<tr><td>732</td><td>77</td><td>GO Process</td><td>cellular amide metabolic process</td><td>8.4E-19</td><td>GSTZ1|MTHFD1|CTSH|OGDH|PDHX|LTA4H|GSTM3|CARS2|MRPL15|GGH|ENPEP|EIF4H|SOD1|NAT8|ALDH1L1|MRPL49|DLAT|OXSM|MRPL17|FTCD|RNPEP|BDH2|TPP1|ANPEP|MRPL39|MRPL13|ASL|PDHB|TSFM|ACSS1|PIPOX|SHMT1|TUFM|CNDP2|ACSM2B|MRPL12|HSP90AA1|DLST|GSTA1|RPS2|SLC25A15|PPA2|AGK|GM2A|MRPL37|MPC1|MRPL21|DARS2|IARS2|GSTO1|ASS1|VARS|PCCA|SUCLA2|ACOT9|PDHA1|DPEP1|PC|NIT2|HAGH|GGT5|GSTP1|GGT1|IDH1|ACSF2|GPX1|LARS2|MRPS22|MME|PCCB|GFM1|GSTA2|ACSL1|MRPS30|ACSM2A|SARS2|GLYAT</td><td>1.06E-20</td><td>GO.0043603</td><td>1.80783135</td></tr>
<tr><td>72</td><td>28</td><td>KEGG Pathways</td><td>Biosynthesis of amino acids</td><td>9.7E-19</td><td>ACO2|IDH3G|GAPDH|ENO1|GOT2|PFKL|IDH3A|ASL|ACO1|SHMT1|BCAT2|IDH2|PKLR|CS|PFKM|PHGDH|GOT1|ALDH18A1|ASS1|ALDOB|PSAT1|IDH3B|PFKP|PC|ACY1|IDH1|TKT|PAH</td><td>2.4E-20</td><td>hsa01230</td><td>1.80136762</td></tr>
<tr><td>98</td><td>31</td><td>GO Process</td><td>alpha-amino acid catabolic process</td><td>2.4E-18</td><td>GSTZ1|AHCY|CRYM|AGXT2|GOT2|HRSP12|HIBADH|AMT|HNMT|ACAD8|QDPR|HGD|DDAH1|HPD|FTCD|PRODH2|PIPOX|SHMT1|BCAT2|DLST|GOT1|HOGA1|SARDH|ALDH4A1|AUH|GLDC|FAH|ALDH7A1|IVD|PAH|ALDH6A1</td><td>3.12E-20</td><td>GO.1901606</td><td>1.7617983</td></tr>
</table>

<table>
<tr><td>2505</td><td>118</td><td>GO Component</td><td>extracellular region</td><td>2.4E-06</td><td>SPATA20|VCL|MAPK1|NID2|F9|CTSH|C1QBP|NAGLU|VTN|CTSC|LTA4H|RAB35|LTF|CTSD|TTR|TUBA4A|ACTN4|LAMA5|NAPSA|LGALS3|APCS|GSTM3|LAMC1|TINAG|GGH|LYZ|PSMD11|COTL1|EHD2|CD81|PSMD3|F13A1|AMBP|LUM|SORD|PFKL|SOD1|TINAGL1|BHMT|COL1A2|COL14A1|AMN|CKB|CCT2|HSP90B1|ANPEP|PDIA3|CA4|GLB1|PRKACA|AQP1|TLN1|CALR|FBN1|BGN|P4HB|SERPINA5|ACAA1|HSP90AA1|TUBB4B|PRDX6|TPSAB1|PTGES2|CTSB|ANXA2|COL18A1|VAT1|PIGR|GM2A|ITIH2|COL4A2|DPP4|CYB5R3|PNP|C4BPA|FLNA|GNAS|XPNPEP2|RBP4|APOOL|HSPG2|ASPN|CLIC1|COL4A1|CUBN|ACTR2|APOO|POSTN|TXNDC5|CD9|GPX3|DPEP1|NME2|CNP|JUP|NIT2|ACTN1|MVP|AMPD3|GSTP1|ACY1|IFI30|PDIA6|TF|ALAD|PCYOX1|IDH1|AHSG|NAPRT|AOC1|SERPINA1|TGFBI|VWA1|IST1|METTL7A|NPC2|ACTG1|TTN</td><td>2.91E-07</td><td>GO.0005576</td><td>0.56252517</td></tr>
<tr><td>57</td><td>26</td><td>GO Function</td><td>NAD binding</td><td>2.8E-18</td><td>AHCY|IDH3G|GAPDH|ALDH2|NNT|HIBADH|SORD|QDPR|GPD1L|BDH2|ALDH1A1|CRYL1|IDH3A|GPD1|GRHPR|UGDH|NDUFV1|IDH2|CYB5R3|PHGDH|ALDH1B1|HADHA|IDH3B|IDH1|ME3|SIRT5</td><td>2.31E-20</td><td>GO.0051287</td><td>1.7552842</td></tr>
<tr><td>294</td><td>48</td><td>GO Process</td><td>fatty acid metabolic process</td><td>4.5E-18</td><td>DECR1|LTA4H|EHHADH|ACADS|MECR|ACAD11|CPT1A|SLC27A2|OXSM|BDH2|CRYL1|ECI1|CYP4A11|ACAD9|CRAT|ACSS1|CES2|HADHB|ACSM2B|ACAA1|GSTA1|PTGES2|ALDH3A2|GPX4|ECHS1|ABCD3|SCP2|ECHDC2|AUH|AKR1C3|HADHA|AMACR|GGT5|GSTP1|GGT1|PTGR1|ACSF2|GPX1|IVD|PCCB|ACSL1|ETFDH|EPHX2|ECHDC1|ACADVL|ETFA|ACSM2A|HAO2</td><td>5.9E-20</td><td>GO.0006631</td><td>1.73467875</td></tr>
<tr><td>774</td><td>78</td><td>GO Process</td><td>exocytosis</td><td>4.6E-18</td><td>RALA|VCL|MAPK1|PGRMC1|CTSH|CTSC|MLEC|LTA4H|LTF|CTSD|TTR|TUBA4A|ACTN4|LGALS3|GGH|ADAM10|LYZ|PSMD11|COTL1|PSMD3|ATP6V0A1|F13A1|SLC27A2|PFKL|SOD1|IQGAP2|SCIN|CCT2|ANPEP|GAA|GLB1|TLN1|AP2A2|RAB11B|ACAA1|HSP90AA1|TUBB4B|PRDX6|PTGES2|CTSB|ANXA2|VAT1|RAC1|PIGR|GM2A|MYH10|CYB5R3|PNP|FLNA|APOOL|RAB14|ACTR2|TXNDC5|CD9|NME2|PRCP|JUP|NIT2|ACTN1|MVP|MGST1|AMPD3|GSTP1|TF|ALAD|SCRN1|IDH1|AHSG|NAPRT|AOC1|SERPINA1|MME|IST1|MGAM|METTL7A|NPC2|ITGB3|TTN</td><td>6.05E-20</td><td>GO.0006887</td><td>1.73419886</td></tr>
<tr><td>485</td><td>61</td><td>GO Process</td><td>neutrophil degranulation</td><td>4.6E-18</td><td>VCL|MAPK1|PGRMC1|CTSH|CTSC|MLEC|LTA4H|LTF|CTSD|TTR|LGALS3|GGH|ADAM10|LYZ|PSMD11|COTL1|PSMD3|ATP6V0A1|SLC27A2|PFKL|IQGAP2|CCT2|ANPEP|GAA|GLB1|AP2A2|ACAA1|HSP90AA1|TUBB4B|PRDX6|PTGES2|CTSB|ANXA2|VAT1|RAC1|PIGR|GM2A|CYB5R3|PNP|RAB14|ACTR2|TXNDC5|NME2|PRCP|JUP|NIT2|MVP|MGST1|AMPD3|GSTP1|ALAD|IDH1|AHSG|NAPRT|AOC1|SERPINA1|MME|IST1|MGAM|METTL7A|NPC2</td><td>6.22E-20</td><td>GO.0043312</td><td>1.73372422</td></tr>
<tr><td>27</td><td>9</td><td>KEGG Pathways</td><td>Ascorbate and aldarate metabolism</td><td>4.8E-06</td><td>ALDH2|UGT2B7|UGDH|UGT2B17|ALDH3A2|UGT1A9|ALDH9A1|ALDH1B1|ALDH7A1</td><td>5.76E-07</td><td>hsa00053</td><td>0.53196645</td></tr>
<tr><td>2086</td><td>140</td><td>GO Process</td><td>phosphorus metabolic process</td><td>5E-18</td><td>NDUFB4|UQCRC1|NANS|MAPK1|MTHFD1|CTSH|OGDH|PNPO|PDHX|GAPDH|DNPH1|SKP1|ATP6V1B1|ENO1|GDA|PMPCB|NDUFA2|NDUFA10|ADAM10|KHK|PEBP1|ATP5B|ATP6V0A1|NNT|SDHA|PRDX5|REXO2|NDUFA9|SORD|UQCRC2|PFKL|ATP6V1A|ATP6V1B2|ERLIN2|NDUFB9|DLAT|OXSM|GPD1L|ATP5O|ATP5J2|ALDH1A1|CRYL1|HPRT1|NDUFB8|CKB|ATP5L|GPD1|ATP5H|DCXR|HINT1|ATP5I|PDHB|CYCS|ATP6V0A4|GPD2|UQCRH|PRKACA|AQP1|GBAS|ACSS1|CYC1|PIPOX|COX5A|SHMT1|UGDH|NDUFV1|MDH2|ACSM2B|NDUFA12|IDH2|UQCR10|HSP90AA1|DLST|PKLR|PRDX6|PPA2|PFKM|GPX4|AGK|RAC1|STOML2|ATP5C1|MPC1|MT-CO1|PNP|OPA1|MT-CO2|ATP1B1|LHPP|ATP5F1|ALDH18A1|SCP2|CMPK1|AKR1A1|YWHAB|RAB14|OGDHL|ALDOB|SUCLA2|ACOT9|PDHA1|DUT|PFKP|AK3|SACM1L|NME2|CNP|DAK|AK4|QPRT|PDP1|AMPD3|ILK|ATP5A1|CDC42|IDH1|TKT|NDUFS1|NAPRT|GK|ACSF2|HK1|CKMT2|CKMT1A|GPX1|FBP1|ABHD14B|ACSL1|COX7C|EPHX2|CRYAB|ATP1A1|TIMM50|COX4I1|ACSM2A|GALK1|TTN|FMO1|PLCG2|GLYAT</td><td>6.89E-20</td><td>GO.0006793</td><td>1.7298432</td></tr>
</table>

<table>
<tr><td>958</td><td>87</td><td>GO Process</td><td>organonitrogen compound catabolic process</td><td>1.2E-17</td><td>DCN|GSTZ1|AHCY|CRYM|CTSH|NAGLU|CTSC|LTA4H|DNPH1|AGXT2|SKP1|CTSD|GDA|GOT2|NAPSA|HRSP12|DMGDH|PSMD11|PSMD3|VCAN|AMBP|ENPEP|HIBADH|LUM|BCKDHA|ALDH1L1|AMT|BHMT|ERLIN2|DPYS|HNMT|ACAD8|QDPR|HGD|DDAH1|UCHL1|HPD|FTCD|CAPN2|RNPEP|HPRT1|TPP1|HSP90B1|ANPEP|PRODH2|HINT1|GLB1|PIPOX|BCKDHB|SHMT1|CHDH|BCAT2|HSPA5|BGN|AP2A2|HSP90AA1|DLST|CTSB|ANXA2|GM2A|LONP1|PNP|GOT1|HOGA1|SARDH|HSPG2|ALDH4A1|AUH|MAOB|DUT|GLDC|QPRT|AMPD3|ABAT|MPST|GGT5|GGT1|FAH|TST|ALDH7A1|CPVL|PCYOX1|GPX1|IVD|PAH|ALDH6A1|AP2B1</td><td>1.64E-19</td><td>GO.1901565</td><td>1.6935542</td></tr>
<tr><td>2065</td><td>138</td><td>GO Process</td><td>phosphate-containing compound metabolic process</td><td>1.4E-17</td><td>NDUFB4|UQCRC1|MAPK1|MTHFD1|CTSH|OGDH|PNPO|PDHX|GAPDH|DNPH1|SKP1|ATP6V1B1|ENO1|GDA|PMPCB|NDUFA2|NDUFA10|ADAM10|KHK|PEBP1|ATP5B|ATP6V0A1|NNT|SDHA|PRDX5|REXO2|NDUFA9|SORD|UQCRC2|PFKL|ATP6V1A|ATP6V1B2|ERLIN2|NDUFB9|DLAT|OXSM|GPD1L|ATP5O|ATP5J2|ALDH1A1|CRYL1|HPRT1|NDUFB8|CKB|ATP5L|GPD1|ATP5H|DCXR|HINT1|ATP5I|PDHB|CYCS|ATP6V0A4|GPD2|UQCRH|PRKACA|AQP1|GBAS|ACSS1|CYC1|PIPOX|COX5A|SHMT1|NDUFV1|MDH2|ACSM2B|NDUFA12|IDH2|UQCR10|HSP90AA1|DLST|PKLR|PRDX6|PPA2|PFKM|GPX4|AGK|RAC1|STOML2|ATP5C1|MPC1|MT-CO1|PNP|OPA1|MT-CO2|ATP1B1|LHPP|ATP5F1|ALDH18A1|SCP2|CMPK1|AKR1A1|YWHAB|RAB14|OGDHL|ALDOB|SUCLA2|ACOT9|PDHA1|DUT|PFKP|AK3|SACM1L|NME2|CNP|DAK|AK4|QPRT|PDP1|AMPD3|ILK|ATP5A1|CDC42|IDH1|TKT|NDUFS1|NAPRT|GK|ACSF2|HK1|CKMT2|CKMT1A|GPX1|FBP1|ABHD14B|ACSL1|COX7C|EPHX2|CRYAB|ATP1A1|TIMM50|COX4I1|ACSM2A|GALK1|TTN|FMO1|PLCG2|GLYAT</td><td>1.98E-19</td><td>GO.0006796</td><td>1.68632794</td></tr>
<tr><td>293</td><td>47</td><td>GO Process</td><td>nucleoside phosphate biosynthetic process</td><td>1.9E-17</td><td>MAPK1|MTHFD1|OGDH|PDHX|GAPDH|ENO1|ATP5B|ATP6V0A1|PFKL|DLAT|ATP5O|ATP5J2|HPRT1|ATP5L|ATP5H|ATP5I|PDHB|ATP6V0A4|AQP1|GBAS|ACSS1|CYC1|SHMT1|IDH2|PKLR|PFKM|STOML2|ATP5C1|MPC1|PNP|LHPP|ATP5F1|CMPK1|OGDHL|ALDOB|PDHA1|DUT|PFKP|NME2|AK4|QPRT|AMPD3|ATP5A1|NAPRT|HK1|ACSL1|GALK1</td><td>2.75E-19</td><td>GO.1901293</td><td>1.67328283</td></tr>
<tr><td>2696</td><td>164</td><td>GO Function</td><td>anion binding</td><td>2E-17</td><td>RALA|MAPK1|RTCB|MYH9|MTHFD1|PCK2|IDH3G|DECR1|OGDH|PNPO|PFN1|C1QBP|VTN|CTSC|RAB35|AGXT2|LTF|ATP6V1B1|ACADS|GOT2|TRAP1|TUBA4A|VIL1|HRSP12|GSTM3|CARS2|KHK|PEBP1|ATP5B|EHD2|SDHA|ACAD11|VCAN|SLC27A2|AFG3L2|PFKL|TIMM44|ATP6V1A|IQGAP2|ATP6V1B2|DPYS|CCT5|ACAD8|QDPR|DDAH1|ACTC1|FTCD|FABP1|ADH5|SCIN|HSPA9|CRYL1|CKB|CCT2|HSP90B1|LDHD|TUBA1C|PRODH2|PRKACA|RAB1B|ACAD9|GRHPR|TLN1|ACSS1|SHMT1|PCK1|CHDH|RHOT2|TUFM|NDUFV1|HSPA5|FBN1|EHD3|ACSM2B|SERPINA5|RAB11B|HSP90AA1|PKLR|TUBB4B|GNAI1|PTGES2|PFKM|ANXA2|ANXA6|UGT1A9|AGK|RAC1|STOML2|MYH10|LONP1|CYB5R3|PNP|OPA1|DARS2|TGM2|MARC2|IARS2|MYO6|ABCD3|GOT1|GNAS|ALDH18A1|SCP2|CMPK1|ASS1|RAB14|OGDHL|VARS|PCCA|ACTR2|MAOB|SUCLA2|POSTN|NQO2|HADHA|PFKP|GLDC|AK3|DNM2|DPEP1|NME2|PC|DAK|AK4|MGST1|MYH11|ABAT|ILK|GSTP1|ATP5A1|SEPT7|CDC42|TXNRD2|CRYZ|GK|ACSF2|HK1|ACTA2|DDC|CKMT2|CKMT1A|FBP1|LARS2|AOC1|BCS1L|IVD|PCCB|GFM1|ACSL1|NDUFA13|ETFDH|ACADVL|ATP1A1|DNM1L|ALDH6A1|ETFA|ACTG1|ACSM2A|GALK1|TTN|SARS2|SIRT5|FMO1|HAO2</td><td>1.81E-19</td><td>GO.0043168</td><td>1.67011469</td></tr>
<tr><td>218</td><td>41</td><td>GO Process</td><td>cofactor biosynthetic process</td><td>2.5E-17</td><td>MTHFD1|OGDH|PNPO|PDHX|GAPDH|SPR|ENO1|NDUFA9|PFKL|SOD1|DLAT|QDPR|BDH2|PDHB|ACSS1|CNDP2|IDH2|PKLR|PFKM|MPC1|PNP|IBA57|AKR1A1|OGDHL|ALDOB|PSAT1|MAOB|SUCLA2|PDHA1|PFKP|FECH|QPRT|TSPO|HAGH|GGT5|GGT1|ALAD|NAPRT|HK1|ACSL1|GALK1</td><td>3.86E-19</td><td>GO.0051188</td><td>1.65968795</td></tr>
</table>

1754	124	GO Function	identical protein binding	3.4E-17	OXCT1|MAPK1|LGALS1|MYH9|GSTZ1|AHCY|CRYM|PNPO|VTN|CTSC|GAPDH|DNPH1|AGXT2|ENO1|TTR|GOT2|VIL1|HSPB1|ACTN4|HRSP12|APCS|GSTM3|ADAM10|LYZ|ERP29|PRDX1|BBOX1|EHD2|ADD1|AMBP|VDAC1|CPT1A|SORD|RHCG|PFKL|SOD1|MUT|DLAT|QDPR|GPD1L|HGD|ADH5|COL1A2|CRYL1|PRDX3|HPRT1|PDIA3|GPD1|DCXR|YWHAG|GLB1|ASL|DPYSL2|AQP1|AOC3|GRHPR|SHMT1|FBN1|HSP90AA1|PRDX6|TPM4|PFKM|ALDH3A2|ANXA2|GPX4|ANXA6|UGT1A9|COL18A1|PBLD|DPP4|DARS2|AKR7A3|S100A10|LHPP|AHCYL1|FLNA|MYO6|ABCD3|HOGA1|ALDH18A1|ASS1|YWHAB|CISD1|ALDOB|ALDH4A1|KCTD12|CUBN|MAOB|ESD|NQO2|PFKP|GLDC|CORO1B|JUP|PC|ACTN1|MVP|QPRT|MGST1|ABAT|MPST|SEPT7|CDC42|RDX|ACY1|ALAD|SLC25A12|IDH1|TKT|SLC4A4|HK1|FBP1|AOC1|SERPINA1|VWA1|EPHX2|CRYAB|RAP1GAP|IST1|VIM|DNM1L|ITGB3|ACTG1|TTN	3.39E-19	GO.0042802	1.64659739
118	32	GO Function	oxidoreductase activity, acting on the CH-OH group of donors, NAD or NADP as acceptor	3.4E-17	IDH3G|EHHADH|SPR|AKR7A2|HIBADH|SORD|GPD1L|ADH5|BDH2|CRYL1|IDH3A|GPD1|DCXR|ADH1B|GRHPR|UGDH|HADHB|DHRS4|MDH2|IDH2|CYB5A|AKR7A3|PHGDH|AKR1A1|AKR1C3|HADHA|IDH3B|BDH1|PTGR1|IDH1|RDH13|ME3	3.59E-19	GO.0016616	1.64659739
84	28	GO Process	fatty acid catabolic process	4.4E-17	DECR1|EHHADH|ACADS|MECR|ACAD11|CPT1A|SLC27A2|BDH2|ECI1|CYP4A11|CRAT|HADHB|ACAA1|ALDH3A2|ECHS1|ABCD3|SCP2|ECHDC2|AUH|HADHA|AMACR|IVD|PCCB|ETFDH|ECHDC1|ACADVL|ETFA|HAO2	6.8E-19	GO.0009062	1.63615107
154	35	GO Process	hexose metabolic process	6E-17	PCK2|SLC25A11|GAPDH|ENO1|GOT2|KHK|CPT1A|SORD|PFKL|DLAT|ALDH1A1|GPD1|DCXR|GAA|GLB1|PDHB|GPD2|PCK1|FBN1|MDH2|PKLR|PFKM|GOT1|RBP4|AKR1A1|ALDOB|PDHA1|PFKP|PC|DAK|SLC25A12|HK1|FBP1|SLC25A10|GALK1	9.46E-19	GO.0019318	1.62232988
113	31	GO Process	pyridine-containing compound metabolic process	6.2E-17	OGDH|PNPO|PDHX|GAPDH|ENO1|NNT|PRDX5|PFKL|GPD1L|GPD1|DCXR|PDHB|GPD2|MDH2|IDH2|PKLR|PFKM|MPC1|PNP|OGDHL|ALDOB|PSAT1|PDHA1|PFKP|QPRT|IDH1|TKT|NAPRT|HK1|GALK1|FMO1	9.85E-19	GO.0072524	1.62111249
291	46	GO Process	nucleotide biosynthetic process	6.9E-17	MAPK1|MTHFD1|OGDH|PDHX|GAPDH|ENO1|ATP5B|ATP6V0A1|PFKL|DLAT|ATP5O|ATP5J2|HPRT1|ATP5L|ATP5H|ATP5I|PDHB|ATP6V0A4|AQP1|GBAS|ACSS1|CYC1|SHMT1|IDH2|PKLR|PFKM|STOML2|ATP5C1|MPC1|PNP|ATP5F1|CMPK1|OGDHL|ALDOB|PDHA1|DUT|PFKP|NME2|AK4|QPRT|AMPD3|ATP5A1|NAPRT|HK1|ACSL1|GALK1	1.12E-18	GO.0009165	1.61598939

5233	254	GO Process	localization	7.7E-17	RALA|VCL|MAPK1|MYH9|PGRMC1|F9|CRYM|CTSH|OGDH|PPIF|SLC25A11|VTN|CTSC|SLC25A3|MLEC|LTA4H|RAB35|SKP1|LTF|EHHADH|ATP6V1B1|CTSD|TTR|GOT2|CANX|TUBA4A|VIL1|HSPB1|PMPCB|MTX2|ACTN4|LAMA5|LGALS3|LAMC1|TINAG|GGH|ADAM10|LRPPRC|LYZ|PSMD11|ERP29|ATP5B|COTL1|SLC9A3R1|EHD2|NAPA|CD81|LRP2|PSMD3|ATP6V0A1|NNT|ADD1|F13A1|VCAN|AMBP|ENPEP|VDAC1|CPT1A|NDUFA9|SORD|SLC27A2|RHCG|UQCRC2|AFG3L2|PFKL|SOD1|TIMM44|TINAGL1|ATP6V1A|IQGAP2|ATP6V1B2|ERLIN2|CCT5|SLC25A4|UCHL1|ATP6V0D2|ATP5O|ATP6V0D1|ATP5J2|COX7A1|FABP1|SCIN|HSPA9|COL1A2|AMN|CCT2|HSP90B1|ANPEP|ATP5L|CA4|TUBA1C|ATP5H|SLC23A1|LETM1|GAA|ATP5I|YWHAG|GLB1|ATP6V0A4|ACO1|DPYSL2|PRKACA|RAB1B|AQP1|DAB2|CRAT|TLN1|CYC1|PIPOX|COX5A|SLC7A8|CALR|RHOT2|HSPA5|FBN1|DHRS4|EHD3|AP2A2|SLC5A2|SERPINA5|RAB11B|ACAA1|PARVA|HSP90AA1|PLS1|TUBB4B|CYB5A|RPS2|PRDX6|SLC25A15|EPB41L3|SLC25A40|PTGES2|SAMM50|CTSB|ANXA2|ANXA6|AGK|VAT1|RAC1|STOML2|PIGR|ATP5C1|GM2A|MSN|MYH10|DPP4|MPC1|CYB5R3|MT-CO1|PNP|OPA1|MT-CO2|TGM2|ATP1B1|S100A10|ATP5F1|AHCYL1|FLNA|SFXN2|MYO6|ABCD3|GNAS|RBP4|SCP2|SLC25A5|PMPCA|VDAC2|YWHAB|APOOL|RAB14|CLIC4|HSPG2|CLIC1|FLOT1|CUBN|SLC3A2|ACTR2|APOO|TXNDC5|MPV17|HBD|AKR1C3|PITRM1|SLC25A6|AMACR|CD9|KRT18|DNM2|NME2|PRCP|CNP|CORO1B|JUP|NIT2|ACTN1|FLOT2|MVP|UMOD|MGST1|TSPO|AMPD3|ILK|GSTP1|ATP5A1|FKBP1A|CDC42|RDX|PDIA6|TST|TF|ALAD|SSB|PCYOX1|SLC25A12|SCRN1|ARPC1B|IDH1|SLC4A4|AHSG|PDZK1|SELENBP1|PNPT1|NAPRT|HK1|DDC|GPX1|TOMM40|AOC1|SERPINA1|MME|ACSL1|NDUFA13|COX7C|VDAC3|EPHX2|IST1|PHB2|ATP1A1|TIMM50|SLC25A10|MGAM|METTL7A|DNM1L|NPC2|ITGB3|COX4I1|ACTG1|RBM8A|TTN|PHB|PLCG2|AP2B1|HAO2	1.28E-18	GO.0051179	1.61146388
56	24	GO Process	fatty acid beta-oxidation	1.2E-16	DECR1|EHHADH|ACADS|MECR|ACAD11|CPT1A|SLC27A2|BDH2|ECI1|CRAT|HADHB|ACAA1|ECHS1|ABCD3|SCP2|ECHDC2|AUH|HADHA|AMACR|IVD|ETFDH|ECHDC1|ACADVL|ETFA	2.11E-18	GO.0006635	1.59065783
206	39	GO Process	purine-containing compound biosynthetic process	1.4E-16	MTHFD1|OGDH|PDHX|GAPDH|ENO1|ATP5B|ATP6V0A1|PFKL|DLAT|ATP5O|ATP5J2|HPRT1|ATP5L|ATP5H|ATP5I|PDHB|ATP6V0A4|AQP1|GBAS|ACSS1|CYC1|SHMT1|PKLR|PFKM|STOML2|ATP5C1|MPC1|PNP|ATP5F1|OGDHL|ALDOB|PDHA1|PFKP|NME2|AMPD3|ATP5A1|HK1|ACSL1|GALK1	2.38E-18	GO.0072522	1.58569852
64	25	GO Process	cellular aldehyde metabolic process	1.4E-16	PNPO|AGXT2|AKR7A2|GOT2|KHK|ALDH8A1|ADH5|ALDH1A1|PRODH2|GRHPR|IDH2|ALDH3A2|ALDH9A1|AKR7A3|HOGA1|AKR1A1|ALDOB|ALDH4A1|ESD|AKR1C3|DAK|HAGH|ALDH7A1|IDH1|TKT	2.42E-18	GO.0006081	1.58569852
513	108	GO Component	organelle inner membrane	5.5E-52	NDUFB4|UQCRC1|PPIF|SLC25A11|SLC25A3|GOT2|TRAP1|PMPCB|NDUFA2|NDUFA10|LGALS3|MRPL15|SQRDL|LRPPRC|ATP5B|CHCHD3|NNT|SDHA|ACAD11|MTFP1|NDUFA9|UQCRC2|AFG3L2|ECSIT|TIMM44|HIGD2A|NDUFB9|MRPL49|SLC25A4|MRPL17|ATP5O|ATP5J2|COX7A1|NDUFB8|LDHD|ATP5L|PRODH2|ATP5H|LETM1|MRPL39|ATP5I|MRPL13|TMEM126A|CYCS|GPD2|C19orf70|UQCRH|ACAD9|CRAT|CYC1|COX5A|CHDH|TMEM11|NDUFV1|HADHB|NDUFA12|UQCR10|MRPL12|SLC25A15|SLC25A40|AGK|STOML2|ATP5C1|MRPL37|MPC1|MT-CO1|MRPL21|OPA1|COA3|MT-CO2|ATP5F1|USMG5|ALDH18A1|SLC25A5|PMPCA|APOOL|APOO|MPV17|HADHA|SLC25A6|FECH|BDH1|CNP|GATM|ATP5A1|IMMT|SLC25A12|RDH13|NDUFS1|HIGD1A|CKMT2|COX20|CKMT1A|TOMM40|BCS1L|MRPS22|TIMMDC1|NDUFA13|MRPS30|COX7C|ETFDH|ACADVL|PHB2|TIMM50|SLC25A10|COX4I1|SIRT5|PHB	6.04E-54	GO.0019866	5.12564902

456	107	GO Component	mitochondrial inner membrane	2.1E-55	NDUFB4|UQCRC1|PPIF|SLC25A11|SLC25A3|GOT2|TRAP1|PMPCB|NDUFA2|NDUFA10|LGALS3|MRPL15|SQRDL|ATP5B|CHCHD3|NNT|SDHA|ACAD11|MTFP1|NDUFA9|UQCRC2|AFG3L2|ECSIT|TIMM44|HIGD2A|NDUFB9|MRPL49|SLC25A4|MRPL17|ATP5O|ATP5J2|COX7A1|NDUFB8|LDHD|ATP5L|PRODH2|ATP5H|LETM1|MRPL39|ATP5I|MRPL13|TMEM126A|CYCS|GPD2|C19orf70|UQCRH|ACAD9|CRAT|CYC1|COX5A|CHDH|TMEM11|NDUFV1|HADHB|NDUFA12|UQCR10|MRPL12|SLC25A15|SLC25A40|AGK|STOML2|ATP5C1|MRPL37|MPC1|MT-CO1|MRPL21|OPA1|COA3|MT-CO2|ATP5F1|USMG5|ALDH18A1|SLC25A5|PMPCA|APOOL|APOO|MPV17|HADHA|SLC25A6|FECH|BDH1|CNP|GATM|ATP5A1|IMMT|SLC25A12|RDH13|NDUFS1|HIGD1A|CKMT2|COX20|CKMT1A|TOMM40|BCS1L|MRPS22|TIMMDC1|NDUFA13|MRPS30|COX7C|ETFDH|ACADVL|PHB2|TIMM50|SLC25A10|COX4I1|SIRT5|PHB	1.98E-57	GO.0005743	5.46736641
119	31	GO Process	nucleoside monophosphate biosynthetic process	2E-16	OGDH|GAPDH|ENO1|ATP5B|ATP6V0A1|PFKL|ATP5O|ATP5J2|HPRT1|ATP5L|ATP5H|ATP5I|ATP6V0A4|GBAS|CYC1|SHMT1|PKLR|PFKM|STOML2|ATP5C1|LHPP|ATP5F1|CMPK1|OGDHL|ALDOB|DUT|PFKP|AMPD3|ATP5A1|HK1|GALK1	3.46E-18	GO.0009124	1.57055338
1554	92	GO Component	whole membrane	4.3E-09	VCL|MAPK1|PGRMC1|MLEC|RAB35|ATP6V1B1|CTSD|MTX2|LGALS3|ADAM10|LRPPRC|SLC9A3R1|EHD2|NAPA|LRP2|ATP6V0A1|VDAC1|CPT1A|SLC27A2|IQGAP2|ERLIN2|ATP6V0D2|ATP6V0D1|CAPN2|AMN|ANPEP|PDIA3|CA4|GAA|ATP6V0A4|PRKACA|RAB1B|GBAS|DAB2|CALR|RHOT2|HADHB|EHD3|AP2A2|SERPINA5|RAB11B|CYB5A|GNAI1|SAMM50|ALDH3A2|ANXA2|ANXA6|AGK|VAT1|RAC1|STOML2|PIGR|DPP4|CYB5R3|OPA1|MARC2|ATP1B1|RTN4IP1|MYO6|SLC25A5|ASS1|VDAC2|RAB14|CISD1|FLOT1|CUBN|MAOB|MPV17|BPHL|CD9|DNM2|PRCP|CNP|FLOT2|MGST1|TSPO|TF|PCYOX1|PDZK1|GK|HK1|NDRG1|TOMM40|MME|ACSL1|VDAC3|PHB2|ATP1A1|MGAM|DNM1L|ITGB3|AP2B1	3.51E-10	GO.0098805	0.83625103
1950	90	GO Component	bounding membrane of organelle	0.00014	PGRMC1|CTSC|MLEC|RAB35|ATP6V1B1|MTX2|LGALS3|ADAM10|LRPPRC|EHD2|NAPA|LRP2|ATP6V0A1|VDAC1|CPT1A|SLC27A2|NAT8|IQGAP2|ATP6V0D2|HPD|ATP6V0D1|FTCD|AMN|ANPEP|PDIA3|CA4|GAA|ATP6V0A4|RAB1B|GBAS|DAB2|CALR|RHOT2|HADHB|EHD3|AP2A2|SERPINA5|RAB11B|CYB5A|PTGES2|SAMM50|ALDH3A2|ANXA2|ANXA6|AGK|VAT1|RAC1|PIGR|CYB5R3|OPA1|MARC2|RTN4IP1|MYO6|GNAS|ASS1|VDAC2|RAB14|CISD1|CUBN|MAOB|APOO|MPV17|BPHL|CD9|SACM1L|DNM2|PRCP|CNP|FLOT2|UMOD|MGST1|TSPO|FKBP1A|CDC42|TF|PCYOX1|GK|HK1|NDRG1|TOMM40|SERPINA1|MME|ACSL1|VDAC3|RAP1GAP|PHB2|MGAM|DNM1L|ITGB3|AP2B1	2.13E-05	GO.0098588	0.3853872
32	20	GO Process	tricarboxylic acid cycle	3E-16	ACO2|IDH3G|OGDH|NNT|SDHA|DLAT|IDH3A|PDHB|ACO1|MDH2|IDH2|DLST|CS|FH|OGDHL|SUCLA2|PDHA1|IDH3B|IDH1|ME3	5.37E-18	GO.0006099	1.55199931
75	26	GO Process	fatty acid oxidation	3.1E-16	DECR1|EHHADH|ACADS|MECR|ACAD11|CPT1A|SLC27A2|BDH2|ECI1|CRAT|HADHB|ACAA1|ALDH3A2|ECHS1|ABCD3|SCP2|ECHDC2|AUH|HADHA|AMACR|IVD|ETFDH|ECHDC1|ACADVL|ETFA|HAO2	5.56E-18	GO.0019395	1.55086383
113	30	GO Process	glucose metabolic process	4.3E-16	PCK2|SLC25A11|GAPDH|ENO1|GOT2|CPT1A|SORD|PFKL|DLAT|GPD1|DCXR|GAA|PDHB|GPD2|PCK1|FBN1|MDH2|PKLR|PFKM|GOT1|RBP4|AKR1A1|ALDOB|PDHA1|PFKP|PC|SLC25A12|HK1|FBP1|SLC25A10	7.79E-18	GO.0006006	1.53716111
40	21	GO Process	tricarboxylic acid metabolic process	6.3E-16	ACO2|IDH3G|OGDH|NNT|SDHA|DLAT|IDH3A|PDHB|ACO1|MDH2|IDH2|DLST|CS|FH|ASS1|OGDHL|SUCLA2|PDHA1|IDH3B|IDH1|ME3	1.16E-17	GO.0072350	1.520412
193	37	GO Process	purine nucleotide biosynthetic process	6.7E-16	MTHFD1|OGDH|PDHX|GAPDH|ENO1|ATP5B|ATP6V0A1|PFKL|DLAT|ATP5O|ATP5J2|HPRT1|ATP5L|ATP5H|ATP5I|PDHB|ATP6V0A4|AQP1|GBAS|ACSS1|CYC1|PKLR|PFKM|STOML2|ATP5C1|MPC1|ATP5F1|OGDHL|ALDOB|PDHA1|PFKP|NME2|AMPD3|ATP5A1|HK1|ACSL1|GALK1	1.26E-17	GO.0006164	1.51713401

948	83	GO Component	secretory vesicle	2.3E-16	VCL|MAPK1|PGRMC1|CTSH|CTSC|MLEC|LTA4H|RAB35|LTF|ATP6V1B1|CTSD|TTR|ACTN4|NAPSA|LGALS3|GGH|ADAM10|LYZ|PSMD11|COTL1|PSMD3|ATP6V0A1|F13A1|SLC27A2|PFKL|SOD1|IQGAP2|ATP6V0D1|RNPEP|CCT2|ANPEP|CA4|GAA|GLB1|PRKACA|CALR|AP2A2|SERPINA5|RAB11B|ACAA1|HSP90AA1|TUBB4B|PRDX6|PTGES2|CTSB|ANXA2|VAT1|RAC1|PIGR|GM2A|CYB5R3|PNP|VDAC2|APOOL|RAB14|ACTR2|TXNDC5|CD9|NME2|PRCP|JUP|NIT2|ACTN1|FLOT2|MVP|MGST1|AMPD3|GSTP1|TF|ALAD|IDH1|AHSG|NAPRT|DDC|AOC1|SERPINA1|MME|IST1|MGAM|METTL7A|DNM1L|NPC2|ITGB3	9.57E-18	GO.0099503	1.56420652
107	29	GO Process	nicotinamide nucleotide metabolic process	9.2E-16	OGDH|PDHX|GAPDH|ENO1|NNT|PRDX5|PFKL|GPD1L|GPD1|DCXR|PDHB|GPD2|MDH2|IDH2|PKLR|PFKM|MPC1|PNP|OGDHL|ALDOB|PDHA1|PFKP|QPRT|IDH1|TKT|NAPRT|HK1|GALK1|FMO1	1.74E-17	GO.0046496	1.50371573
98	28	GO Process	purine ribonucleoside monophosphate biosynthetic process	1E-15	OGDH|GAPDH|ENO1|ATP5B|ATP6V0A1|PFKL|ATP5O|ATP5J2|HPRT1|ATP5L|ATP5H|ATP5I|ATP6V0A4|GBAS|CYC1|PKLR|PFKM|STOML2|ATP5C1|LHPP|ATP5F1|OGDHL|ALDOB|PFKP|AMPD3|ATP5A1|HK1|GALK1	1.98E-17	GO.0009168	1.49913998
574	62	GO Process	myeloid leukocyte activation	1.4E-15	VCL|MAPK1|PGRMC1|CTSH|CTSC|MLEC|LTA4H|LTF|CTSD|TTR|LGALS3|GGH|ADAM10|LYZ|PSMD11|COTL1|PSMD3|ATP6V0A1|SLC27A2|PFKL|IQGAP2|CCT2|ANPEP|GAA|GLB1|AP2A2|ACAA1|HSP90AA1|TUBB4B|PRDX6|PTGES2|CTSB|ANXA2|VAT1|RAC1|PIGR|GM2A|CYB5R3|PNP|RAB14|ACTR2|TXNDC5|NME2|PRCP|JUP|NIT2|MVP|MGST1|AMPD3|GSTP1|ALAD|IDH1|AHSG|NAPRT|NDRG1|AOC1|SERPINA1|MME|IST1|MGAM|METTL7A|NPC2	2.71E-17	GO.0002274	1.48601209
36	20	GO Process	citrate metabolic process	1.5E-15	ACO2|IDH3G|OGDH|NNT|SDHA|DLAT|IDH3A|PDHB|ACO1|MDH2|IDH2|DLST|CS|FH|OGDHL|SUCLA2|PDHA1|IDH3B|IDH1|ME3	3.01E-17	GO.0006101	1.48210231
110	29	GO Process	ribonucleoside monophosphate biosynthetic process	1.6E-15	OGDH|GAPDH|ENO1|ATP5B|ATP6V0A1|PFKL|ATP5O|ATP5J2|HPRT1|ATP5L|ATP5H|ATP5I|ATP6V0A4|GBAS|CYC1|PKLR|PFKM|STOML2|ATP5C1|LHPP|ATP5F1|CMPK1|OGDHL|ALDOB|PFKP|AMPD3|ATP5A1|HK1|GALK1	3.25E-17	GO.0009156	1.4790485
1969	80	GO Component	cell projection	0.0102	MAPK1|MYH9|AHCY|PGRMC1|RAB35|REEP6|ATP6V1B1|CANX|VIL1|HSPB1|ACTN4|GSTM3|SLC9A3R1|LRP2|CALB1|SORD|SOD1|ATP6V1A|IQGAP2|ATP6V1B2|HNMT|QDPR|UCHL1|ACTC1|ATP6V0D1|CAPN2|SCIN|AMN|CKB|CA4|DCXR|ATP6V0A4|PRKACA|AQP1|AOC3|ACAD9|TLN1|EHD3|HSP90AA1|EPB41L3|PFKM|ANXA2|RAC1|MSN|MYH10|DPP4|OPA1|FLNA|MYO6|GOT1|GNAS|ASS1|CLIC4|FLOT1|CUBN|ACTR2|DNM2|DPEP1|NME2|CNP|CORO1B|ACTN1|FLOT2|UMOD|ABAT|ILK|MPST|SEPT7|CDC42|RDX|PDZK1|ACTA2|DDC|MME|CRYAB|PHB2|VIM|ITGB3|RBM8A|PDLIM5	0.0026	GO.0042995	0.19913998
632	65	GO Process	leukocyte mediated immunity	2E-15	VCL|MAPK1|PGRMC1|CTSH|C1QBP|CTSC|MLEC|LTA4H|LTF|CTSD|TTR|LGALS3|GGH|ADAM10|LYZ|PSMD11|COTL1|PRDX1|PSMD3|ATP6V0A1|SLC27A2|PFKL|IQGAP2|HPRT1|CCT2|ANPEP|GAA|GLB1|AP2A2|ACAA1|HSP90AA1|TUBB4B|PRDX6|PTGES2|CTSB|ANXA2|VAT1|RAC1|PIGR|GM2A|CYB5R3|PNP|C4BPA|RAB14|ACTR2|TXNDC5|NME2|PRCP|JUP|NIT2|MVP|MGST1|AMPD3|GSTP1|ALAD|IDH1|AHSG|NAPRT|AOC1|SERPINA1|MME|IST1|MGAM|METTL7A|NPC2	4.03E-17	GO.0002443	1.47011469
168	34	KEGG Pathways	Alzheimer's disease	2E-15	NDUFB4|UQCRC1|MAPK1|GAPDH|NDUFA2|NDUFA10|ADAM10|ATP5B|SDHA|NDUFA9|UQCRC2|NDUFB9|ATP5O|COX7A1|CAPN2|NDUFB8|ATP5H|CYCS|UQCRH|CYC1|COX5A|NDUFV1|NDUFA12|UQCR10|ATP5C1|MT-CO1|MT-CO2|ATP5F1|ATP5A1|NDUFS1|MME|NDUFA13|COX7C|COX4I1	6.47E-17	hsa05010	1.4692504
189	36	GO Process	purine ribonucleotide biosynthetic process	2.1E-15	OGDH|PDHX|GAPDH|ENO1|ATP5B|ATP6V0A1|PFKL|DLAT|ATP5O|ATP5J2|HPRT1|ATP5L|ATP5H|ATP5I|PDHB|ATP6V0A4|AQP1|GBAS|ACSS1|CYC1|PKLR|PFKM|STOML2|ATP5C1|MPC1|ATP5F1|OGDHL|ALDOB|PDHA1|PFKP|NME2|AMPD3|ATP5A1|HK1|ACSL1|GALK1	4.19E-17	GO.0009152	1.46882461

202	37	GO Process	ribonucleotide biosynthetic process	2.3E-15	OGDH|PDHX|GAPDH|ENO1|ATP5B|ATP6V0A1|PFKL|DLAT|ATP5O|ATP5J2|HPRT1|ATP5L|ATP5H|ATP5I|PDHB|ATP6V0A4|AQP1|GBAS|ACSS1|CYC1|PKLR|PFKM|STOML2|ATP5C1|MPC1|ATP5F1|CMPK1|OGDHL|ALDOB|PDHA1|PFKP|NME2|AMPD3|ATP5A1|HK1|ACSL1|GALK1	4.64E-17	GO.0009260	1.46478175
75	25	GO Process	ATP biosynthetic process	2.5E-15	OGDH|GAPDH|ENO1|ATP5B|ATP6V0A1|PFKL|ATP5O|ATP5J2|ATP5L|ATP5H|ATP5I|ATP6V0A4|GBAS|CYC1|PKLR|PFKM|STOML2|ATP5C1|ATP5F1|OGDHL|ALDOB|PFKP|ATP5A1|HK1|GALK1	5.22E-17	GO.0006754	1.460206
123	30	GO Process	mitochondrial membrane organization	2.7E-15	PPIF|ATP5B|CHCHD3|AFG3L2|SLC25A4|ATP5O|ATP5J2|ATP5L|ATP5H|LETM1|ATP5I|YWHAG|C19orf70|RHOT2|HSP90AA1|SAMM50|AGK|ATP5C1|OPA1|ATP5F1|SLC25A5|YWHAB|APOOL|APOO|SLC25A6|CNP|ATP5A1|IMMT|NDUFA13|TIMM50	5.7E-17	GO.0007006	1.45670307
103	28	GO Process	nucleoside triphosphate biosynthetic process	2.8E-15	OGDH|GAPDH|ENO1|ATP5B|ATP6V0A1|PFKL|ATP5O|ATP5J2|ATP5L|ATP5H|ATP5I|ATP6V0A4|GBAS|CYC1|PKLR|PFKM|STOML2|ATP5C1|ATP5F1|CMPK1|OGDHL|ALDOB|PFKP|NME2|AK4|ATP5A1|HK1|GALK1	5.88E-17	GO.0009142	1.45575202
124	30	GO Process	oxidoreduction coenzyme metabolic process	3.2E-15	OGDH|PDHX|GAPDH|ENO1|NNT|PRDX5|NDUFA9|PFKL|GPD1L|GPD1|DCXR|PDHB|GPD2|MDH2|IDH2|PKLR|PFKM|MPC1|PNP|OGDHL|ALDOB|PDHA1|PFKP|QPRT|IDH1|TKT|NAPRT|HK1|GALK1|FMO1	6.89E-17	GO.0006733	1.44921441
86	26	GO Process	purine ribonucleoside triphosphate biosynthetic process	4.1E-15	OGDH|GAPDH|ENO1|ATP5B|ATP6V0A1|PFKL|ATP5O|ATP5J2|ATP5L|ATP5H|ATP5I|ATP6V0A4|GBAS|CYC1|PKLR|PFKM|STOML2|ATP5C1|ATP5F1|OGDHL|ALDOB|PFKP|NME2|ATP5A1|HK1|GALK1	8.89E-17	GO.0009206	1.43893398
64	23	Reactome Pathways	Mitochondrial protein import	4.9E-15	ACO2|IDH3G|PMPCB|MTX2|ATP5B|CHCHD3|VDAC1|TIMM44|SLC25A4|HSPA9|NDUFB8|LDHD|CYC1|CS|SAMM50|PMPCA|PITRM1|SLC25A6|ATP5A1|SLC25A12|TOMM40|BCS1L|TIMM50	2.45E-16	HSA-1268020	1.43098039
577	61	GO Process	organophosphate biosynthetic process	5.7E-15	MAPK1|MTHFD1|OGDH|PNPO|PDHX|GAPDH|ENO1|ATP5B|ATP6V0A1|SORD|PFKL|DLAT|GPD1L|ATP5O|ATP5J2|CRYL1|HPRT1|ATP5L|GPD1|ATP5H|DCXR|ATP5I|PDHB|ATP6V0A4|AQP1|GBAS|ACSS1|CYC1|SHMT1|IDH2|PKLR|PFKM|AGK|STOML2|ATP5C1|MPC1|PNP|LHPP|ATP5F1|SCP2|CMPK1|AKR1A1|RAB14|OGDHL|ALDOB|PDHA1|DUT|PFKP|SACM1L|NME2|AK4|QPRT|AMPD3|ATP5A1|TKT|NAPRT|GK|HK1|ACSL1|GALK1|PLCG2	1.26E-16	GO.0090407	1.42471836
616	63	GO Process	leukocyte activation involved in immune response	7.3E-15	VCL|MAPK1|LGALS1|PGRMC1|CTSH|CTSC|MLEC|LTA4H|LTF|CTSD|TTR|LGALS3|GGH|ADAM10|LYZ|PSMD11|COTL1|PSMD3|ATP6V0A1|SLC27A2|PFKL|IQGAP2|CCT2|ANPEP|GAA|GLB1|AP2A2|ACAA1|HSP90AA1|TUBB4B|PRDX6|PTGES2|CTSB|ANXA2|VAT1|RAC1|PIGR|GM2A|CYB5R3|PNP|RAB14|ACTR2|TXNDC5|NME2|PRCP|JUP|NIT2|MVP|MGST1|AMPD3|GSTP1|ALAD|IDH1|AHSG|NAPRT|AOC1|SERPINA1|MME|IST1|MGAM|METTL7A|NPC2|PLCG2	1.64E-16	GO.0002366	1.4134896
959	81	GO Process	secretion by cell	7.6E-15	RALA|VCL|MAPK1|PGRMC1|CTSH|CTSC|MLEC|LTA4H|LTF|CTSD|TTR|CANX|TUBA4A|ACTN4|LGALS3|GGH|ADAM10|LYZ|PSMD11|ERP29|COTL1|PSMD3|ATP6V0A1|F13A1|SLC27A2|PFKL|SOD1|IQGAP2|SCIN|CCT2|ANPEP|GAA|GLB1|TLN1|AP2A2|RAB11B|ACAA1|HSP90AA1|TUBB4B|PRDX6|PTGES2|CTSB|ANXA2|VAT1|RAC1|PIGR|GM2A|MYH10|CYB5R3|PNP|FLNA|GNAS|APOOL|RAB14|ACTR2|TXNDC5|CD9|NME2|PRCP|JUP|NIT2|ACTN1|MVP|MGST1|AMPD3|GSTP1|TF|ALAD|SCRN1|IDH1|AHSG|NAPRT|AOC1|SERPINA1|MME|IST1|MGAM|METTL7A|NPC2|ITGB3|TTN	1.7E-16	GO.0032940	1.4122053
228	38	KEGG Pathways	Thermogenesis	7.7E-15	NDUFB4|UQCRC1|NDUFA2|NDUFA10|ATP5B|SDHA|CPT1A|NDUFA9|UQCRC2|NDUFB9|ATP5O|ATP5J2|COX7A1|NDUFB8|ATP5L|ATP5H|ATP5I|UQCRH|PRKACA|CYC1|COX5A|NDUFV1|NDUFA12|UQCR10|ATP5C1|MT-CO1|COA3|MT-CO2|ATP5F1|GNAS|ATP5A1|NDUFS1|COX20|ACSL1|NDUFA13|COX7C|COX4I1|ACTG1	2.71E-16	hsa04714	1.41146388
41	20	GO Process	inner mitochondrial membrane organization	9.2E-15	ATP5B|CHCHD3|AFG3L2|ATP5O|ATP5J2|ATP5L|ATP5H|LETM1|ATP5I|C19orf70|SAMM50|AGK|ATP5C1|OPA1|ATP5F1|APOOL|APOO|ATP5A1|IMMT|NDUFA13	2.09E-16	GO.0007007	1.40376307

1900	79	GO Component	plasma membrane bounded cell projection	0.0063	MAPK1|MYH9|AHCY|PGRMC1|RAB35|REEP6|ATP6V1B1|CANX|VIL1|HSPB1|ACTN4|GSTM3|SLC9A3R1|LRP2|CALB1|SORD|SOD1|ATP6V1A|IQGAP2|ATP6V1B2|HNMT|QDPR|UCHL1|ACTC1|ATP6V0D1|CAPN2|AMN|CKB|CA4|DCXR|ATP6V0A4|PRKACA|AQP1|AOC3|ACAD9|TLN1|EHD3|HSP90AA1|EPB41L3|PFKM|ANXA2|RAC1|MSN|MYH10|DPP4|OPA1|FLNA|MYO6|GOT1|GNAS|ASS1|CLIC4|FLOT1|CUBN|ACTR2|DNM2|DPEP1|NME2|CNP|CORO1B|ACTN1|FLOT2|UMOD|ABAT|ILK|MPST|SEPT7|CDC42|RDX|PDZK1|ACTA2|DDC|MME|CRYAB|PHB2|VIM|ITGB3|RBM8A|PDLIM5	0.0015	GO.0120025	0.22006595
167	33	GO Process	cellular lipid catabolic process	1.5E-14	DECR1|EHHADH|ACADS|MECR|ACAD11|CPT1A|SLC27A2|FABP1|BDH2|ECI1|CYP4A11|CRAT|HADHB|ACAA1|PRDX6|ALDH3A2|GM2A|ECHS1|ABCD3|SCP2|ECHDC2|AUH|AKR1C3|HADHA|AMACR|IVD|PCCB|ETFDH|ECHDC1|ACADVL|ETFA|PLCG2|HAO2	3.44E-16	GO.0044242	1.38268137
1796	78	GO Component	endoplasmic reticulum	0.0023	LGALS1|RTCB|PGRMC1|F9|VTN|CTSC|MLEC|REEP6|CANX|LAMC1|ADAM10|ERP29|LRP2|VCAN|SLC27A2|NAT8|ERLIN2|UCHL1|HPD|FTCD|CAPN2|COL1A2|COL14A1|NDUFB8|HSP90B1|PPIB|PDIA3|CA4|UGT2B7|ACO1|RAB1B|CYP4A11|AOC3|CRAT|CES2|UGT2B17|CALR|HSPA5|HADHB|FBN1|DHRS4|P4HB|CYB5A|ALDH3A2|UGT1A9|COL18A1|RAC1|ITIH2|COL4A2|CYB5R3|TGM2|MGST3|S100A10|AHCYL1|ASS1|RAB14|COL4A1|CUBN|ESD|APOO|TXNDC5|SACM1L|MGST1|FKBP1A|CDC42|PDIA6|TF|AHSG|PNPT1|SERPINA1|VWA1|ACSL1|ATP1A1|METTL7A|DNM1L|NPC2|EPHX1|FMO1	0.0005	GO.0005783	0.26382722
83	25	GO Process	mitochondrial transmembrane transport	1.6E-14	ATP5B|CPT1A|AFG3L2|TIMM44|ATP5O|ATP5J2|ATP5L|ATP5H|LETM1|ATP5I|CYC1|SLC25A15|AGK|STOML2|ATP5C1|MPC1|OPA1|ATP5F1|ATP5A1|TST|SLC25A12|PNPT1|TOMM40|NDUFA13|TIMM50	3.76E-16	GO.1990542	1.379588
41	20	GO Function	oxidoreductase activity, acting on the aldehyde or oxo group of donors	1.8E-14	OGDH|GAPDH|ALDH2|ALDH8A1|BCKDHA|ALDH1L1|ADH5|ALDH1A1|PDHB|BCKDHB|ALDH3A2|ALDH9A1|ALDH18A1|OGDHL|ALDH4A1|ALDH1B1|PDHA1|AKR1C3|ALDH7A1|ALDH6A1	2.09E-16	GO.0016903	1.37423214
28	11	KEGG Pathways	Butanoate metabolism	9.3E-08	OXCT1|EHHADH|ACADS|BDH2|ACSM2B|ECHS1|HMGCS2|HADHA|BDH1|ABAT|ACSM2A	7.84E-09	hsa00650	0.70338583
30	21	KEGG Pathways	Citrate cycle (TCA cycle)	5E-18	ACO2|PCK2|IDH3G|OGDH|SDHA|DLAT|IDH3A|PDHB|ACO1|PCK1|MDH2|IDH2|DLST|CS|FH|OGDHL|SUCLA2|PDHA1|IDH3B|PC|IDH1	1.42E-19	hsa00020	1.7298432
1070	85	GO Process	secretion	3.4E-14	RALA|VCL|MAPK1|PGRMC1|CTSH|CTSC|MLEC|LTA4H|LTF|CTSD|TTR|GOT2|CANX|TUBA4A|ACTN4|LGALS3|GGH|ADAM10|LYZ|PSMD11|ERP29|COTL1|SLC9A3R1|PSMD3|ATP6V0A1|F13A1|SLC27A2|PFKL|SOD1|IQGAP2|SCIN|CCT2|ANPEP|GAA|GLB1|AQP1|TLN1|AP2A2|RAB11B|ACAA1|HSP90AA1|TUBB4B|PRDX6|PTGES2|CTSB|ANXA2|VAT1|RAC1|PIGR|GM2A|MYH10|CYB5R3|PNP|MT-CO2|FLNA|GNAS|APOOL|RAB14|ACTR2|TXNDC5|CD9|NME2|PRCP|JUP|NIT2|ACTN1|MVP|MGST1|AMPD3|GSTP1|TF|ALAD|SCRN1|IDH1|AHSG|NAPRT|AOC1|SERPINA1|MME|IST1|MGAM|METTL7A|NPC2|ITGB3|TTN	7.95E-16	GO.0046903	1.34749552
828	77	GO Component	secretory granule	1.9E-16	VCL|MAPK1|PGRMC1|CTSH|CTSC|MLEC|LTA4H|LTF|CTSD|TTR|ACTN4|NAPSA|LGALS3|GGH|ADAM10|LYZ|PSMD11|COTL1|PSMD3|ATP6V0A1|F13A1|SLC27A2|PFKL|SOD1|IQGAP2|RNPEP|CCT2|ANPEP|CA4|GAA|GLB1|PRKACA|CALR|AP2A2|SERPINA5|ACAA1|HSP90AA1|TUBB4B|PRDX6|PTGES2|CTSB|ANXA2|VAT1|RAC1|PIGR|GM2A|CYB5R3|PNP|VDAC2|APOOL|RAB14|ACTR2|TXNDC5|CD9|NME2|PRCP|JUP|NIT2|ACTN1|FLOT2|MVP|MGST1|AMPD3|GSTP1|TF|ALAD|IDH1|AHSG|NAPRT|AOC1|SERPINA1|MME|IST1|MGAM|METTL7A|NPC2|ITGB3	7.57E-18	GO.0030141	1.57281584
48	20	KEGG Pathways	Arginine and proline metabolism	5E-14	GOT2|ALDH2|CKB|PRODH2|CNDP2|ALDH3A2|ALDH9A1|GOT1|HOGA1|ALDH18A1|ALDH4A1|AGMAT|ALDH1B1|MAOB|GATM|SMS|ALDH7A1|CKMT2|CKMT1A|AOC1	2.28E-15	hsa00330	1.33036436

4153	207	GO Process	response to chemical	6.2E-14	RALA|DCN|OXCT1|UQCRC1|MAPK1|LGALS1|GSTZ1|AHCY|CTSH|PLGRKT|PPIF|CTSC|GAPDH|RAB35|SKP1|ATP6V1B1|AKR7A2|GOT2|CANX|VIL1|HSPB1|LAMA5|LGALS3|HRSP12|GSTM3|MRPL15|GGH|ADAM10|KHK|PRDX1|CD81|LRP2|ATP6V0A1|NNT|ADD1|F13A1|CALB1|PRDX5|CPT1A|LUM|SORD|PFKL|SOD1|NAT8|ATP6V1A|ATP6V1B2|ERLIN2|QDPR|SLC25A4|ATP6V0D2|ACTC1|ATP6V0D1|CAPN2|FABP1|ADH5|CMBL|HSPA9|COL1A2|PRDX3|HPRT1|AMN|TPP1|HSP90B1|GPD1|SLC23A1|YWHAG|GLB1|CYCS|ATP6V0A4|ACO1|DPYSL2|PRKACA|AQP1|AOC3|TLN1|CYC1|CES2|SHMT1|PCK1|SLC7A8|CALR|TUFM|BCAT2|HSPA5|FBN1|BGN|ACSM2B|P4HB|RAB11B|PARVA|HSP90AA1|GSTA1|PKLR|CYB5A|PRDX6|SLC25A15|GNAI1|CTSB|ANXA2|GPX4|UGT1A9|COL18A1|RAC1|PIGR|MSN|MYH10|COL4A2|LONP1|MPC1|MT-CO1|PNP|OPA1|AKR7A3|MARC2|MGST3|S100A10|PHGDH|GSTO1|AHCYL1|MYO6|ABCD3|GOT1|GNAS|RBP4|SLC25A5|ASS1|RAB14|CLIC4|ASPN|COL4A1|FLOT1|CUBN|SLC3A2|ACTR2|MAOB|ESD|POSTN|MT1M|MPV17|HBD|BPHL|NQO2|AKR1C3|HADHA|PFKP|GLDC|SLC25A6|CD9|FECH|GPX3|KRT18|DNM2|DPEP1|CNP|CORO1B|JUP|AK4|MGST1|TSPO|ABAT|ILK|MPST|GSTP1|CDC42|GGT1|TXNRD2|RDX|ACY1|IFI30|PDIA6|PTGR1|TF|ALAD|SLC25A12|ARPC1B|IDH1|PDZK1|HIGD1A|CRYZ|PNPT1|DDC|NDRG1|GPX1|FBP1|AOC1|MME|GSTA2|ACSL1|NDUFA13|EPHX2|CRYAB|RAP1GAP|ACADVL|PHB2|ATP1A1|VIM|SLC25A10|DNM1L|ITGB3|COX4I1|ACTG1|TTN|PHB|EPHX1|FMO1|PLCG2|GLYAT	1.47E-15	GO.0042221	1.32104193
1047	77	GO Component	membrane protein complex	1.4E-11	NDUFB4|UQCRC1|PPIF|VTN|ATP6V1B1|PMPCB|NDUFA2|NDUFA10|ATP5B|CHCHD3|NAPA|ATP6V0A1|SDHA|VDAC1|NDUFA9|UQCRC2|AFG3L2|ATP6V1A|ATP6V1B2|NDUFB9|ATP6V0D2|ATP5O|ATP6V0D1|ATP5J2|NDUFB8|ATP5L|ATP5H|ATP5I|ATP6V0A4|C19orf70|UQCRH|PRKACA|CYC1|COX5A|CALR|NDUFV1|AP2A2|NDUFA12|UQCR10|GNAI1|SAMM50|AGK|ATP5C1|MT-CO1|MT-CO2|KRT19|ATP1B1|ATP5F1|USMG5|GNAS|VDAC2|APOOL|CLIC4|HSPG2|CLIC1|FLOT1|APOO|SLC25A6|SACM1L|JUP|FLOT2|SNTB1|ATP5A1|FKBP1A|TF|IMMT|NDUFS1|TOMM40|BCS1L|NDUFA13|COX7C|VDAC3|ATP1A1|TIMM50|ITGB3|COX4I1|AP2B1	8.81E-13	GO.0098796	1.08601209
251	61	GO Component	mitochondrial protein complex	1.1E-31	NDUFB4|UQCRC1|PPIF|PMPCB|NDUFA2|NDUFA10|MRPL15|ATP5B|CHCHD3|SDHA|VDAC1|NDUFA9|UQCRC2|AFG3L2|BCKDHA|NDUFB9|MRPL49|DLAT|MRPL17|ATP5O|ATP5J2|NDUFB8|ATP5L|ATP5H|MRPL39|ATP5I|MRPL13|C19orf70|UQCRH|CYC1|COX5A|BCKDHB|NDUFV1|NDUFA12|UQCR10|MRPL12|SAMM50|AGK|ATP5C1|MRPL37|MT-CO1|MRPL21|MT-CO2|ATP5F1|USMG5|APOOL|APOO|HADHA|SLC25A6|ATP5A1|IMMT|NDUFS1|PNPT1|TOMM40|BCS1L|MRPS22|NDUFA13|COX7C|ETFDH|TIMM50|COX4I1	2.47E-33	GO.0098798	3.09746941
96	25	GO Process	respiratory electron transport chain	2.7E-13	NDUFB4|UQCRC1|PMPCB|NDUFA2|NDUFA10|SDHA|NDUFA9|NDUFB9|NDUFB8|GPD1|CYCS|GPD2|UQCRH|CYC1|COX5A|NDUFV1|NDUFA12|UQCR10|MT-CO1|MT-CO2|SLC25A12|NDUFS1|COX7C|ETFDH|COX4I1	6.45E-15	GO.0022904	1.25718652
1699	111	GO Process	vesicle-mediated transport	3E-13	RALA|VCL|MAPK1|MYH9|PGRMC1|F9|CTSH|VTN|CTSC|MLEC|LTA4H|RAB35|LTF|CTSD|TTR|CANX|TUBA4A|ACTN4|LGALS3|TINAG|GGH|ADAM10|LYZ|PSMD11|COTL1|EHD2|NAPA|CD81|LRP2|PSMD3|ATP6V0A1|F13A1|AMBP|SLC27A2|PFKL|SOD1|TINAGL1|IQGAP2|SCIN|AMN|CCT2|HSP90B1|ANPEP|GAA|GLB1|DPYSL2|RAB1B|DAB2|TLN1|CALR|EHD3|AP2A2|RAB11B|ACAA1|HSP90AA1|TUBB4B|PRDX6|PTGES2|CTSB|ANXA2|VAT1|RAC1|PIGR|GM2A|MYH10|CYB5R3|PNP|TGM2|S100A10|FLNA|MYO6|APOOL|RAB14|HSPG2|CUBN|ACTR2|TXNDC5|CD9|KRT18|DNM2|NME2|PRCP|JUP|NIT2|ACTN1|MVP|MGST1|AMPD3|GSTP1|CDC42|PDIA6|TF|ALAD|SCRN1|ARPC1B|IDH1|AHSG|NAPRT|AOC1|SERPINA1|MME|IST1|MGAM|METTL7A|DNM1L|NPC2|ITGB3|ACTG1|TTN|PLCG2|AP2B1	7.3E-15	GO.0016192	1.25228787

97	25	GO Process	dicarboxylic acid metabolic process	3.2E-13	MTHFD1|OGDH|GOT2|SDHA|ALDH1L1|FTCD|PRODH2|GRHPR|SHMT1|PCK1|MDH2|IDH2|DLST|FH|GOT1|HOGA1|ALDH18A1|ASS1|ALDH4A1|SUCLA2|NIT2|QPRT|GGT1|IDH1|ME3	7.9E-15	GO.0043648	1.2493495
8349	340	GO Process	nitrogen compound metabolic process	3.2E-13	DCN|NDUFB4|UQCRC1|NANS|MAPK1|LGALS1|RTCB|MYH9|GSTZ1|MTHFD1|AHCY|F9|CRYM|CTSH|OGDH|PPIF|PNPO|C1QBP|NAGLU|CTSC|PDHX|LTA4H|GAPDH|DNPH1|AGXT2|SKP1|LTF|EHHADH|ATP6V1B1|SPR|ENO1|AKR7A2|CTSD|TTR|GDA|PEPD|GOT2|PMPCB|NDUFA2|NDUFA10|NAPSA|LGALS3|GRSF1|HRSP12|APCS|DMGDH|BHMT2|GSTM3|CARS2|LAMC1|TINAG|MRPL15|GGH|ADAM10|LRPPRC|LYZ|PEBP1|PSMD11|ATP5B|CHCHD3|BBOX1|PSMD3|ATP6V0A1|NNT|ADD1|F13A1|SDHA|VCAN|AMBP|ENPEP|HIBADH|CALB1|PRDX5|CPT1A|EIF4H|REXO2|NDUFA9|LUM|UQCRC2|AFG3L2|PFKL|BCKDHA|SOD1|TINAGL1|NAT8|ATP6V1A|ALDH1L1|AMT|BHMT|MUT|ATP6V1B2|ERLIN2|LACTB2|DPYS|NDUFB9|MRPL49|HNMT|DLAT|OXSM|ACAD8|QDPR|GPD1L|HGD|DDAH1|UCHL1|MRPL17|HPD|ATP5O|FTCD|ATP5J2|CAPN2|RNPEP|ADH5|BDH2|PRDX3|HPRT1|AMN|NDUFB8|CKB|TPP1|HSP90B1|PPIB|ANPEP|GLYATL1|PDIA3|ATP5L|GPD1|PRODH2|ATP5H|SLC23A1|DCXR|HINT1|MRPL39|ATP5I|MRPL13|GLB1|ASL|PDHB|CYCS|ATP6V0A4|GPD2|DPYSL2|UQCRH|PRKACA|RAB1B|ISCU|AQP1|AOC3|GBAS|GRHPR|TSFM|MSRA|CRAT|ACSS1|CYC1|PIPOX|COX5A|BCKDHB|SHMT1|UGDH|PCK1|CHDH|SLC7A8|CALR|TUFM|NDUFV1|BCAT2|HSPA5|FBN1|CNDP2|MDH2|BGN|ACSM2B|AP2A2|P4HB|NDUFA12|IDH2|UQCR10|RAB11B|MRPL12|HSP90AA1|TMLHE|DLST|GSTA1|PKLR|RPS2|SLC25A15|TPSAB1|PPA2|CTSB|PFKM|ALDH3A2|ANXA2|ALDH9A1|AGK|RAC1|STOML2|ATP5C1|GM2A|ITIH2|COL4A2|DPP3|DPP4|LONP1|MRPL37|MPC1|MT-CO1|PNP|MRPL21|OPA1|MT-CO2|DARS2|TGM2|IBA57|MARC2|IARS2|C4BPA|ATP1B1|MGST3|LHPP|PHGDH|GSTO1|ATP5F1|AHCYL1|FLNA|GOT1|HOGA1|GNAS|XPNPEP2|ALDH18A1|PMPCA|SARDH|CMPK1|AKR1A1|ASS1|YWHAB|OGDHL|HSPG2|ALDOB|ALDH4A1|VARS|AUH|AGMAT|PCCA|PSAT1|CUBN|MAOB|SUCLA2|ESD|ACOT9|PDHA1|GLRX|BPHL|AKR1C3|DUT|PITRM1|PFKP|GLDC|AK3|FECH|DPEP1|NME2|PRCP|CNP|PC|SUOX|NIT2|AK4|QPRT|PDP1|MGST1|TSPO|AMPD3|ABAT|GATM|ILK|MPST|HAGH|GGT5|GSTP1|ATP5A1|FKBP1A|CDC42|GGT1|ACY1|FAH|PDIA6|SMS|TST|TF|ALAD|SSB|ALDH7A1|CPVL|PCYOX1|SCRN1|IDH1|TKT|NDUFS1|AHSG|PNPT1|NAPRT|ACSF2|HK1|DDC|CKMT2|CKMT1A|GPX1|LARS2|AOC1|SERPINA1|TGFBI|VWA1|MRPS22|IVD|MME|PCCB|GFM1|SSBP1|ABHD14B|GSTA2|ACSL1|MRPS30|COX7C|CRYAB|PHB2|TIMM50|PAH|ALDH6A1|COX4I1|ACSM2A|RBM8A|GALK1|TTN|SARS2|SIRT5|PHB|FMO1|AP2B1|GLYAT	7.86E-15	GO.0006807	1.2493495
31	17	GO Process	cristae formation	3.5E-13	ATP5B|CHCHD3|AFG3L2|ATP5O|ATP5J2|ATP5L|ATP5H|LETM1|ATP5I|C19orf70|SAMM50|ATP5C1|ATP5F1|APOOL|APOO|ATP5A1|IMMT	8.69E-15	GO.0042407	1.24571746
113	26	GO Process	drug catabolic process	9.1E-13	OXCT1|GSTZ1|CTSH|HRSP12|ALDH2|PRDX1|PRDX5|AMT|DPYS|QDPR|HGD|HPD|PRDX3|PIPOX|DLST|PRDX6|ALDH1B1|MAOB|GLDC|GPX3|BDH1|QPRT|AMPD3|FAH|GPX1|PAH	2.28E-14	GO.0042737	1.20409586
1061	60	GO Component	plasma membrane region	1.8E-05	RALA|MAPK1|MYH9|RAB35|ATP6V1B1|SLC9A3R1|EHD2|CD81|LRP2|ENPEP|RHCG|ATP6V1A|ATP6V0D2|ATP6V0D1|AMN|CDH16|CA4|SLC23A1|ATP6V0A4|PRKACA|CYP4A11|AQP1|TLN1|SLC7A8|EHD3|HSP90AA1|EPB41L3|PFKM|ANXA2|RAC1|GM2A|MSN|MYH10|DPP4|KRT19|ATP1B1|AHCYL1|MYO6|GNAS|AKR1A1|FLOT1|KCTD12|CUBN|SLC3A2|CD9|DNM2|DPEP1|JUP|FLOT2|UMOD|SEPT7|RDX|TF|SLC4A4|PDZK1|CRYAB|ATP1A1|MGAM|ITGB3|PDLIM5	2.35E-06	GO.0098590	0.47447275

425	48	GO Process	organic hydroxy compound metabolic process	1.1E-12	ACO2|IDH3G|CRYM|PNPO|SPR|AKR7A2|TTR|ALDH2|LRP2|SORD|SLC27A2|ERLIN2|QDPR|ADH5|ALDH1A1|HPRT1|GPD1|ADH1B|GPD2|CYP4A11|ACSS1|PCK1|DHRS4|IDH2|ACAA1|ALDH3A2|CYB5R3|HMGCS2|GOT1|RBP4|SCP2|PSAT1|ALDH1B1|CUBN|MAOB|AKR1C3|IDH3B|AMACR|DAK|HAGH|IDH1|RDH13|GK|DDC|PAH|NPC2|GALK1|PLCG2	2.87E-14	GO.1901615	1.19469216
137	28	GO Process	proton transmembrane transport	1.2E-12	SLC25A3|ATP6V1B1|ATP5B|ATP6V0A1|NNT|ATP6V1A|ATP6V1B2|ATP6V0D2|ATP5O|ATP6V0D1|ATP5J2|COX7A1|ATP5L|ATP5H|LETM1|ATP5I|ATP6V0A4|CYC1|COX5A|CYB5A|STOML2|ATP5C1|MT-CO1|MT-CO2|ATP5F1|ATP5A1|COX7C|COX4I1	2.99E-14	GO.1902600	1.1928118
104	25	GO Process	carbohydrate catabolic process	1.2E-12	OGDH|GAPDH|ENO1|KHK|SORD|PFKL|ABHD10|ALDH1A1|CRYL1|DCXR|GAA|GLB1|GPD2|PKLR|PFKM|GM2A|AKR1A1|OGDHL|ALDOB|PFKP|DAK|GK|HK1|MGAM|GALK1	3.08E-14	GO.0016052	1.19208188
66	21	GO Process	pyruvate metabolic process	1.4E-12	OGDH|PDHX|GAPDH|ENO1|PFKL|DLAT|PDHB|PCK1|PKLR|PFKM|MPC1|HOGA1|OGDHL|ALDOB|PDHA1|PFKP|PC|HAGH|HK1|ME3|GALK1	3.6E-14	GO.0006090	1.18569852
61	9	KEGG Pathways	Arachidonic acid metabolism	0.00085	LTA4H|CYP4A11|PTGES2|AKR1C3|GPX3|GGT5|GGT1|GPX1|EPHX2	0.00017	hsa00590	0.30705811
108	25	GO Process	xenobiotic metabolic process	2.5E-12	AKR7A2|GSTM3|CMBL|AOC3|CES2|ACSM2B|UGT1A9|AKR7A3|MGST3|PHGDH|GSTO1|BPHL|NQO2|DPEP1|MGST1|GSTP1|GGT1|ACY1|CRYZ|AOC1|ACSL1|EPHX2|EPHX1|FMO1|GLYAT	6.44E-14	GO.0006805	1.1607303
6066	266	GO Function	ion binding	4.6E-12	RALA|UQCRC1|MAPK1|RTCB|MYH9|ACO2|NID2|MTHFD1|PCK2|IDH3G|F9|DECR1|OGDH|PNPO|PFN1|C1QBP|VTN|CTSC|LTA4H|RAB35|AGXT2|LTF|ATP6V1B1|ENO1|GDA|ACADS|PEPD|GOT2|TRAP1|CANX|TUBA4A|VIL1|PMPCB|ACTN4|HRSP12|APCS|BHMT2|GSTM3|CARS2|ADAM10|KHK|PEBP1|ATP5B|BBOX1|EHD2|LRP2|F13A1|SDHA|ACAD11|VCAN|ENPEP|CALB1|SORD|SLC27A2|UQCRC2|AFG3L2|PFKL|BCKDHA|SOD1|TIMM44|ATP6V1A|BHMT|IQGAP2|MUT|ATP6V1B2|LACTB2|DPYS|CCT5|ACAD8|QDPR|HGD|DDAH1|HPD|ACTC1|FTCD|CAPN2|RNPEP|FABP1|ADH5|SCIN|HSPA9|COL1A2|CRYL1|HPRT1|CKB|CCT2|TPP1|IDH3A|CDH16|HSP90B1|LDHD|ANPEP|CA4|TUBA1C|PRODH2|LETM1|ADH1B|CYCS|GPD2|ACO1|PRKACA|RAB1B|ISCU|CSRP2|CYP4A11|AOC3|ACAD9|GRHPR|TLN1|ACSS1|CYC1|COX5A|SHMT1|PCK1|CHDH|CALR|RHOT2|TUFM|NDUFV1|HSPA5|FBN1|CNDP2|EHD3|ACSM2B|C11orf54|IDH2|SERPINA5|RAB11B|HSP90AA1|TMLHE|PLS1|PKLR|TUBB4B|CYB5A|GNAI1|PPA2|TPM4|PTGES2|PFKM|ANXA2|ANXA6|UGT1A9|AGK|COL18A1|VAT1|RAC1|STOML2|MYH10|DPP3|LONP1|CYB5R3|MT-CO1|PNP|OPA1|MT-CO2|DARS2|TGM2|MARC2|IARS2|CSRP1|S100A10|LHPP|RTN4IP1|MYO6|ABCD3|GOT1|GNAS|XPNPEP2|ALDH18A1|SCP2|PMPCA|CMPK1|ASS1|RAB14|CISD1|OGDHL|HSPG2|ASPN|VARS|AGMAT|PCCA|CUBN|ACTR2|MAOB|SUCLA2|POSTN|MT1M|HBD|NQO2|HADHA|IDH3B|DUT|PITRM1|PFKP|GLDC|AK3|FECH|DNM2|DPEP1|NME2|PC|SUOX|FHL1|ACTN1|DAK|AK4|UMOD|PDP1|MGST1|MYH11|AMPD3|ABAT|ILK|HAGH|GSTP1|ATP5A1|SEPT7|CDC42|TXNRD2|ACY1|FAH|TF|ALAD|SLC25A12|IDH1|TKT|NDUFS1|CRYZ|GK|ACSF2|HK1|ACTA2|DDC|CKMT2|CKMT1A|FBP1|LARS2|GBE1|AOC1|BCS1L|IVD|MME|PCCB|GFM1|ACSL1|NDUFA13|ETFDH|EPHX2|CRYAB|ACADVL|ME3|ATP1A1|MYL6|PAH|DNM1L|ALDH6A1|ETFA|ACTG1|ACSM2A|GALK1|TTN|SARS2|SIRT5|PDLIM5|FMO1|HAO2	5.63E-14	GO.0043167	1.13419886
172	30	GO Process	sulfur compound biosynthetic process	5.3E-12	DCN|GSTZ1|MTHFD1|AHCY|PDHX|BHMT2|GSTM3|VCAN|LUM|BHMT|DLAT|PDHB|ACSS1|CNDP2|BGN|GSTA1|MPC1|MGST3|GSTO1|AKR1A1|ESD|PDHA1|MGST1|MPST|HAGH|GGT5|GSTP1|GGT1|GSTA2|ACSL1	1.4E-13	GO.0044272	1.12740884
55	19	GO Process	monosaccharide biosynthetic process	6.7E-12	PCK2|SLC25A11|GAPDH|ENO1|GOT2|SORD|GPD1|GPD2|PCK1|MDH2|GOT1|RBP4|AKR1A1|ALDOB|PC|SLC25A12|TKT|FBP1|SLC25A10	1.78E-13	GO.0046364	1.11713401

31	16	GO Function	oxidoreductase activity, acting on the aldehyde or oxo group of donors, NAD or NADP as acceptor	8.4E-12	OGDH|GAPDH|ALDH2|ALDH8A1|ALDH1L1|ADH5|ALDH1A1|ALDH3A2|ALDH9A1|ALDH18A1|ALDH4A1|ALDH1B1|PDHA1|AKR1C3|ALDH7A1|ALDH6A1	1.11E-13	GO.0016620	1.1076756
2448	138	GO Function	hydrolase activity	8.6E-12	RALA|UQCRC1|MYH9|GSTZ1|MTHFD1|AHCY|F9|CTSH|ABHD11|NAGLU|CTSC|LTA4H|RAB35|DNPH1|LTF|ATP6V1B1|AKR7A2|CTSD|GDA|HDHD3|PEPD|TUBA4A|PMPCB|NAPSA|HRSP12|TINAG|GGH|ADAM10|LRPPRC|LYZ|ATP5B|EHD2|ATP6V0A1|ENPEP|EIF4H|REXO2|UQCRC2|AFG3L2|TINAGL1|ABHD10|ATP6V1A|MUT|ATP6V1B2|LACTB2|DPYS|DDAH1|UCHL1|ATP6V0D2|ATP5O|ACTC1|ATP6V0D1|CAPN2|RNPEP|CMBL|TPP1|ANPEP|PDIA3|TUBA1C|HINT1|GAA|GLB1|ATP6V0A4|DPYSL2|RAB1B|CES2|RHOT2|TUFM|HSPA5|CNDP2|C11orf54|RAB11B|HSP90AA1|TUBB4B|PRDX6|GNAI1|TPSAB1|PPA2|CTSB|RAC1|ATP5C1|GM2A|MYH10|DPP3|DPP4|LONP1|OPA1|IARS2|ATP1B1|LHPP|MYO6|ABCD3|GNAS|XPNPEP2|PMPCA|RAB14|VARS|AGMAT|ESD|ACOT9|BPHL|DUT|PITRM1|SACM1L|DNM2|DPEP1|PRCP|CNP|NIT2|PDP1|MYH11|AMPD3|HAGH|GGT5|ATP5A1|CDC42|GGT1|ACY1|FAH|CPVL|PCYOX1|SCRN1|PNPT1|FBP1|LARS2|GBE1|MME|GFM1|ABHD14B|EPHX2|RAP1GAP|ATP1A1|TIMM50|MYL6|MGAM|DNM1L|SIRT5|EPHX1|PLCG2	1.21E-13	GO.0016787	1.10660068
894	71	GO Process	leukocyte activation	9.7E-12	VCL|MAPK1|LGALS1|MYH9|PGRMC1|CTSH|CTSC|MLEC|LTA4H|LTF|CTSD|TTR|LGALS3|GGH|ADAM10|LYZ|PSMD11|COTL1|PRDX1|PSMD3|ATP6V0A1|SLC27A2|PFKL|IQGAP2|HPRT1|CCT2|ANPEP|GAA|GLB1|AP2A2|ACAA1|HSP90AA1|TUBB4B|PRDX6|PTGES2|CTSB|ANXA2|VAT1|RAC1|STOML2|PIGR|GM2A|MSN|DPP4|CYB5R3|PNP|RAB14|ACTR2|TXNDC5|NME2|PRCP|JUP|NIT2|MVP|MGST1|AMPD3|GSTP1|FKBP1A|ALAD|IDH1|AHSG|NAPRT|NDRG1|AOC1|SERPINA1|MME|IST1|MGAM|METTL7A|NPC2|PLCG2	2.57E-13	GO.0045321	1.10145735
157	58	GO Component	myelin sheath	3.1E-38	RALA|UQCRC1|ACO2|SLC25A3|GOT2|CANX|NDUFA10|ATP5B|PRDX1|NAPA|SDHA|UQCRC2|SOD1|ATP6V1A|ATP6V1B2|CCT5|DLAT|SLC25A4|UCHL1|HSPA9|PRDX3|CKB|CCT2|IDH3A|PDIA3|ATP5H|YWHAG|COX5A|TUFM|HSPA5|MDH2|EHD3|HSP90AA1|DLST|TUBB4B|ANXA2|ATP5C1|MSN|ATP1B1|PHGDH|ATP5F1|SLC25A5|ASS1|VDAC2|SUCLA2|PDHA1|CNP|ATP5A1|RDX|IMMT|SLC25A12|TKT|NDUFS1|NDRG1|CRYAB|ATP1A1|ACTG1|PHB	5.28E-40	GO.0043209	3.75100415
175	30	GO Function	lyase activity	1.4E-11	ACO2|PCK2|EHHADH|ENO1|BCKDHA|FTCD|CA4|ECI1|ASL|ACO1|SHMT1|PCK1|HADHB|PTGES2|FH|MGST3|ECHS1|GOT1|HOGA1|ECHDC2|ALDOB|AUH|HADHA|GLDC|FECH|DAK|ALAD|DDC|ECHDC1|ME3	2.08E-13	GO.0016829	1.0853872
78	21	GO Process	mitochondrial ATP synthesis coupled electron transport	2.1E-11	NDUFB4|UQCRC1|PMPCB|NDUFA2|NDUFA10|SDHA|NDUFA9|NDUFB9|NDUFB8|CYCS|UQCRH|CYC1|COX5A|NDUFV1|NDUFA12|UQCR10|MT-CO1|MT-CO2|NDUFS1|COX7C|COX4I1	5.56E-13	GO.0042775	1.06840297
157	28	GO Process	cellular response to xenobiotic stimulus	2.1E-11	AKR7A2|GSTM3|CMBL|AQP1|AOC3|CES2|ACSM2B|UGT1A9|AKR7A3|MGST3|PHGDH|GSTO1|ASS1|BPHL|NQO2|FECH|DPEP1|MGST1|GSTP1|GGT1|ACY1|CRYZ|AOC1|ACSL1|EPHX2|EPHX1|FMO1|GLYAT	5.7E-13	GO.0071466	1.06757175
1192	84	GO Process	lipid metabolic process	2.2E-11	DECR1|LTA4H|EHHADH|TTR|ACADS|ATP5B|MECR|LRP2|ACAD11|ALDH8A1|CPT1A|SLC27A2|ERLIN2|OXSM|ACAD8|GPD1L|FABP1|ADH5|BDH2|ALDH1A1|CRYL1|TPP1|GPD1|ECI1|UGT2B7|GLB1|CYP4A11|ACAD9|CRAT|ACSS1|CES2|PCK1|UGT2B17|HADHB|DHRS4|ACSM2B|ACAA1|GSTA1|PRDX6|PTGES2|ALDH3A2|GPX4|UGT1A9|AGK|GM2A|CYB5R3|MGST3|ECHS1|HMGCS2|ABCD3|RBP4|SCP2|ECHDC2|RAB14|HSPG2|AUH|CUBN|AKR1C3|HADHA|AMACR|SACM1L|PC|TSPO|GGT5|GSTP1|ATP5A1|GGT1|PTGR1|RDH13|GK|ACSF2|GPX1|IVD|PCCB|ACSL1|ETFDH|EPHX2|ECHDC1|ACADVL|NPC2|ETFA|ACSM2A|PLCG2|HAO2	6.05E-13	GO.0006629	1.06516951

468	48	GO Process	response to toxic substance	2.7E-11	OXCT1|MAPK1|GSTZ1|PPIF|GOT2|GSTM3|GGH|PRDX1|NNT|PRDX5|SOD1|ACTC1|FABP1|ADH5|PRDX3|HPRT1|SLC23A1|AQP1|SLC7A8|TUFM|GSTA1|PRDX6|GPX4|MARC2|MGST3|GSTO1|RBP4|ASS1|ACTR2|MAOB|ESD|BPHL|FECH|GPX3|DNM2|CNP|MGST1|ABAT|MPST|GSTP1|TXNRD2|ALAD|DDC|GPX1|EPHX2|CRYAB|EPHX1|GLYAT	7.38E-13	GO.0009636	1.05734887
1024	76	GO Process	cell activation	3E-11	VCL|MAPK1|LGALS1|MYH9|PGRMC1|CTSH|CTSC|MLEC|LTA4H|LTF|CTSD|TTR|LGALS3|GGH|ADAM10|LYZ|PSMD11|COTL1|PRDX1|PSMD3|ATP6V0A1|SLC27A2|PFKL|IQGAP2|COL1A2|HPRT1|CCT2|ANPEP|GAA|GLB1|AP2A2|ACAA1|HSP90AA1|TUBB4B|PRDX6|PTGES2|CTSB|ANXA2|VAT1|RAC1|STOML2|PIGR|GM2A|MSN|DPP4|CYB5R3|PNP|FLNA|GNAS|RAB14|ACTR2|TXNDC5|CD9|NME2|PRCP|JUP|NIT2|MVP|MGST1|AMPD3|GSTP1|FKBP1A|ALAD|IDH1|AHSG|NAPRT|NDRG1|AOC1|SERPINA1|MME|IST1|MGAM|METTL7A|NPC2|ITGB3|PLCG2	8.27E-13	GO.0001775	1.05272436
2672	144	GO Process	cellular response to chemical stimulus	3E-11	RALA|DCN|MAPK1|LGALS1|GSTZ1|CTSH|PPIF|GAPDH|RAB35|SKP1|ATP6V1B1|AKR7A2|CANX|VIL1|HSPB1|LAMA5|LGALS3|GSTM3|MRPL15|PRDX1|ATP6V0A1|NNT|ADD1|F13A1|CALB1|PRDX5|CPT1A|SOD1|ATP6V1A|ATP6V1B2|QDPR|ATP6V0D2|ATP6V0D1|CAPN2|FABP1|ADH5|CMBL|HSPA9|COL1A2|PRDX3|TPP1|HSP90B1|GPD1|YWHAG|CYCS|ATP6V0A4|PRKACA|AQP1|AOC3|TLN1|CES2|SHMT1|PCK1|CALR|BCAT2|HSPA5|FBN1|BGN|ACSM2B|P4HB|RAB11B|PARVA|HSP90AA1|GSTA1|PKLR|PRDX6|GNAI1|CTSB|ANXA2|GPX4|UGT1A9|RAC1|MSN|COL4A2|LONP1|MPC1|OPA1|AKR7A3|MGST3|PHGDH|GSTO1|AHCYL1|GOT1|GNAS|SLC25A5|ASS1|RAB14|CLIC4|ASPN|COL4A1|FLOT1|ACTR2|ESD|POSTN|MT1M|MPV17|BPHL|NQO2|AKR1C3|PFKP|GLDC|FECH|GPX3|KRT18|DNM2|DPEP1|CORO1B|JUP|MGST1|TSPO|ILK|GSTP1|CDC42|GGT1|TXNRD2|RDX|ACY1|IFI30|PDIA6|TF|ALAD|HIGD1A|CRYZ|PNPT1|DDC|NDRG1|GPX1|FBP1|AOC1|MME|GSTA2|ACSL1|NDUFA13|EPHX2|ACADVL|PHB2|ATP1A1|VIM|DNM1L|ACTG1|PHB|EPHX1|FMO1|GLYAT	8.43E-13	GO.0070887	1.05214335
200	31	GO Process	monocarboxylic acid biosynthetic process	3.1E-11	DCN|OGDH|LTA4H|GAPDH|ENO1|MECR|VCAN|ALDH8A1|SLC27A2|PFKL|OXSM|ACSS1|BGN|ACSM2B|PKLR|PTGES2|PFKM|GPX4|ABCD3|HOGA1|SCP2|OGDHL|ALDOB|AKR1C3|PFKP|AMACR|ABAT|HK1|EPHX2|ACSM2A|GALK1	8.8E-13	GO.0072330	1.05058454
120	25	GO Function	proton transmembrane transporter activity	3.2E-11	SLC25A3|ATP6V1B1|ATP5B|ATP6V0A1|NNT|ATP6V1A|ATP6V1B2|ATP6V0D2|ATP5O|ATP6V0D1|COX7A1|ATP5L|ATP5H|LETM1|ATP5I|ATP6V0A4|COX5A|CYB5A|ATP5C1|MT-CO1|MT-CO2|ATP5F1|ATP5A1|COX7C|COX4I1	5.01E-13	GO.0015078	1.04962093
20	5	KEGG Pathways	Arginine biosynthesis	0.0029	GOT2|ASL|GOT1|ASS1|ACY1	0.00063	hsa00220	0.2537602
682	55	GO Component	vacuole	1.2E-09	DCN|MAPK1|CTSH|NAGLU|CTSC|ATP6V1B1|CTSD|TTR|NAPSA|GGH|LYZ|NAPA|LRP2|ATP6V0A1|VCAN|LUM|SOD1|ATP6V0D2|ATP6V0D1|CAPN2|CCT2|TPP1|GAA|GLB1|ATP6V0A4|DAB2|BGN|AP2A2|HSP90AA1|TUBB4B|PRDX6|PTGES2|CTSB|ANXA2|ANXA6|VAT1|PIGR|GM2A|CYB5R3|MYO6|GOT1|ASS1|RAB14|HSPG2|CUBN|ACTR2|TXNDC5|PRCP|MGST1|IFI30|PCYOX1|NAPRT|IST1|NPC2|AP2B1	9.23E-11	GO.0005773	0.8924453
1295	53	GO Component	catalytic complex	0.0396	NDUFB4|UQCRC1|OGDH|PDHX|SKP1|ENO1|HSPB1|PMPCB|NDUFA2|NDUFA10|PSMD11|PSMD3|SDHA|NDUFA9|UQCRC2|AFG3L2|PFKL|BCKDHA|NDUFB9|DLAT|GPD1L|NDUFB8|GPD1|HINT1|PDHB|CYCS|GPD2|UQCRH|PRKACA|CYC1|BCKDHB|NDUFV1|P4HB|NDUFA12|UQCR10|DLST|GNAI1|PFKM|MT-CO1|ATP1B1|MYO6|GNAS|OGDHL|PDHA1|GLDC|ABAT|NDUFS1|PNPT1|BCS1L|NDUFA13|ETFDH|ATP1A1|RBM8A	0.012	GO.1902494	0.14023048

582	51	GO Component	lysosome	4.1E-10	DCN|MAPK1|CTSH|NAGLU|CTSC|CTSD|TTR|NAPSA|GGH|LYZ|LRP2|ATP6V0A1|VCAN|LUM|SOD1|ATP6V0D2|CAPN2|CCT2|TPP1|GAA|GLB1|DAB2|BGN|AP2A2|HSP90AA1|TUBB4B|PRDX6|PTGES2|CTSB|ANXA2|ANXA6|VAT1|PIGR|GM2A|CYB5R3|MYO6|GOT1|ASS1|RAB14|HSPG2|CUBN|ACTR2|TXNDC5|PRCP|MGST1|IFI30|PCYOX1|NAPRT|IST1|NPC2|AP2B1	3.05E-11	GO.0005764	0.93893398
262	35	GO Process	response to xenobiotic stimulus	5E-11	AKR7A2|GOT2|GSTM3|SOD1|CMBL|HPRT1|AQP1|AOC3|CES2|HSPA5|ACSM2B|UGT1A9|AKR7A3|MGST3|PHGDH|GSTO1|ASS1|BPHL|NQO2|FECH|DNM2|DPEP1|MGST1|ABAT|GSTP1|GGT1|ACY1|CRYZ|GPX1|AOC1|ACSL1|EPHX2|EPHX1|FMO1|GLYAT	1.4E-12	GO.0009410	1.03053948
1134	50	GO Component	extracellular space	0.0145	F9|CTSH|C1QBP|NAGLU|VTN|LTF|CTSD|LAMA5|NAPSA|APCS|LAMC1|TINAG|GGH|LYZ|CD81|SORD|SOD1|TINAGL1|BHMT|COL1A2|AMN|CKB|ANPEP|PDIA3|CA4|AQP1|CALR|FBN1|SERPINA5|CTSB|ANXA2|PIGR|C4BPA|XPNPEP2|RBP4|CLIC1|CUBN|APOO|POSTN|CD9|GPX3|DPEP1|CNP|ACY1|PDIA6|PCYOX1|AOC1|SERPINA1|TGFBI|ACTG1	0.0039	GO.0005615	0.1838632
50	17	KEGG Pathways	Glutathione metabolism	7.4E-11	GSTM3|NAT8|ANPEP|IDH2|GSTA1|GPX4|MGST3|GSTO1|GPX3|MGST1|GGT5|GSTP1|GGT1|SMS|IDH1|GPX1|GSTA2	4.16E-12	hsa00480	1.01325325
167	28	GO Process	coenzyme biosynthetic process	7.5E-11	MTHFD1|OGDH|PNPO|PDHX|GAPDH|SPR|ENO1|NDUFA9|PFKL|DLAT|QDPR|PDHB|ACSS1|IDH2|PKLR|PFKM|MPC1|PNP|AKR1A1|OGDHL|ALDOB|PDHA1|PFKP|QPRT|NAPRT|HK1|ACSL1|GALK1	2.14E-12	GO.0009108	1.01260984
340	48	GO Component	cytoplasmic vesicle lumen	2.6E-16	VCL|MAPK1|CTSH|CTSC|LTF|CTSD|TTR|ACTN4|GGH|LYZ|PSMD11|COTL1|PSMD3|F13A1|PFKL|CCT2|HSP90B1|GLB1|CALR|ACAA1|HSP90AA1|TUBB4B|PRDX6|PTGES2|ANXA2|VAT1|GM2A|CYB5R3|PNP|APOOL|ACTR2|TXNDC5|NME2|JUP|NIT2|ACTN1|MVP|AMPD3|GSTP1|TF|ALAD|IDH1|AHSG|NAPRT|AOC1|SERPINA1|IST1|NPC2	1.15E-17	GO.0060205	1.55783961
149	26	KEGG Pathways	Non-alcoholic fatty liver disease (NAFLD)	9.9E-11	NDUFB4|UQCRC1|NDUFA2|NDUFA10|SDHA|NDUFA9|UQCRC2|NDUFB9|COX7A1|NDUFB8|CYCS|UQCRH|CYC1|COX5A|NDUFV1|NDUFA12|UQCR10|PKLR|RAC1|MT-CO1|MT-CO2|CDC42|NDUFS1|NDUFA13|COX7C|COX4I1	5.97E-12	hsa04932	1.00026136
946	71	GO Process	cellular lipid metabolic process	1.1E-10	DECR1|LTA4H|EHHADH|TTR|ACADS|MECR|LRP2|ACAD11|ALDH8A1|CPT1A|SLC27A2|OXSM|GPD1L|FABP1|ADH5|BDH2|ALDH1A1|CRYL1|GPD1|ECI1|GLB1|CYP4A11|ACAD9|CRAT|ACSS1|CES2|PCK1|HADHB|DHRS4|ACSM2B|ACAA1|GSTA1|PRDX6|PTGES2|ALDH3A2|GPX4|UGT1A9|AGK|GM2A|ECHS1|HMGCS2|ABCD3|RBP4|SCP2|ECHDC2|RAB14|HSPG2|AUH|AKR1C3|HADHA|AMACR|SACM1L|GGT5|GSTP1|GGT1|PTGR1|RDH13|GK|ACSF2|GPX1|IVD|PCCB|ACSL1|ETFDH|EPHX2|ECHDC1|ACADVL|ETFA|ACSM2A|PLCG2|HAO2	3.15E-12	GO.0044255	0.99625735
49	17	GO Process	hexose biosynthetic process	1.1E-10	PCK2|SLC25A11|GAPDH|ENO1|GOT2|SORD|GPD1|GPD2|PCK1|MDH2|GOT1|RBP4|ALDOB|PC|SLC25A12|FBP1|SLC25A10	3.19E-12	GO.0019319	0.99586073
927	70	GO Process	immune effector process	1.2E-10	VCL|MAPK1|LGALS1|PGRMC1|CTSH|C1QBP|CTSC|MLEC|LTA4H|LTF|CTSD|TTR|LGALS3|GGH|ADAM10|LYZ|PSMD11|COTL1|PRDX1|PSMD3|ATP6V0A1|SLC27A2|PFKL|IQGAP2|HPRT1|CCT2|ANPEP|GAA|GLB1|AP2A2|ACAA1|HSP90AA1|TUBB4B|PRDX6|PTGES2|CTSB|ANXA2|VAT1|RAC1|PIGR|GM2A|CYB5R3|PNP|C4BPA|RAB14|ACTR2|TXNDC5|NME2|PRCP|JUP|NIT2|MVP|MGST1|AMPD3|GSTP1|CDC42|ALAD|ARPC1B|IDH1|AHSG|NAPRT|AOC1|SERPINA1|MME|IST1|MGAM|METTL7A|NPC2|ACTG1|PLCG2	3.5E-12	GO.0002252	0.99208188
17	12	KEGG Pathways	2-Oxocarboxylic acid metabolism	1.4E-10	ACO2|IDH3G|GOT2|IDH3A|ACO1|BCAT2|IDH2|CS|GOT1|IDH3B|ACY1|IDH1	9.07E-12	hsa01210	0.9844664
729	60	GO Process	membrane organization	1.9E-10	RALA|MYH9|PPIF|CTSC|ATP5B|CHCHD3|EHD2|NAPA|LRP2|AFG3L2|SOD1|SLC25A4|ATP5O|ATP5J2|ATP5L|ATP5H|LETM1|ATP5I|YWHAG|C19orf70|RAB1B|DAB2|CALR|RHOT2|AP2A2|SERPINA5|HSP90AA1|EPB41L3|SAMM50|ANXA2|AGK|STOML2|ATP5C1|MYH10|OPA1|ATP1B1|S100A10|ATP5F1|SLC25A5|YWHAB|APOOL|FLOT1|ACTR2|APOO|SLC25A6|CD9|DNM2|CNP|FLOT2|ATP5A1|CDC42|TF|IMMT|NDRG1|SERPINA1|NDUFA13|TIMM50|DNM1L|ACTG1|AP2B1	5.54E-12	GO.0061024	0.97235382

31	14	KEGG Pathways	beta-Alanine metabolism	2.4E-10	EHHADH|ALDH2|DPYS|AOC3|CNDP2|ALDH3A2|ALDH9A1|ECHS1|ALDH1B1|HADHA|ABAT|SMS|ALDH7A1|ALDH6A1	1.58E-11	hsa00410	0.96289321
323	46	GO Component	secretory granule lumen	9E-16	VCL|MAPK1|CTSH|CTSC|LTF|CTSD|TTR|ACTN4|GGH|LYZ|PSMD11|COTL1|PSMD3|F13A1|PFKL|CCT2|GLB1|ACAA1|HSP90AA1|TUBB4B|PRDX6|PTGES2|ANXA2|VAT1|GM2A|CYB5R3|PNP|APOOL|ACTR2|TXNDC5|NME2|JUP|NIT2|ACTN1|MVP|AMPD3|GSTP1|TF|ALAD|IDH1|AHSG|NAPRT|AOC1|SERPINA1|IST1|NPC2	4.18E-17	GO.0034774	1.5047692
11878	429	GO Function	binding	4.1E-10	RALA|DCN|OXCT1|UQCRC1|VCL|MAPK1|LGALS1|RTCB|MYH9|ACO2|NID2|GSTZ1|MTHFD1|PCK2|AHCY|IDH3G|PGRMC1|F9|CRYM|CTSH|DECR1|OGDH|PPIF|PNPO|PFN1|C1QBP|VTN|CTSC|SLC25A3|MLEC|LTA4H|GAPDH|RAB35|DNPH1|AGXT2|SKP1|LTF|EHHADH|ATP6V1B1|SPR|ENO1|TTR|GDA|ACADS|PEPD|GOT2|DSTN|TRAP1|CANX|TUBA4A|VIL1|HSPB1|PMPCB|ACTN4|LAMA5|LGALS3|GRSF1|HRSP12|APCS|BHMT2|GSTM3|CARS2|TINAG|SQRDL|ADAM10|KHK|LRPPRC|LYZ|PEBP1|ALDH2|ERP29|ATP5B|COTL1|CHCHD3|SLC9A3R1|PRDX1|BBOX1|EHD2|NAPA|CD81|LRP2|ATP6V0A1|NNT|ADD1|F13A1|SDHA|ACAD11|VCAN|AMBP|ENPEP|VDAC1|HIBADH|CALB1|PRDX5|CPT1A|EIF4H|REXO2|NDUFA9|RBP5|LUM|SORD|SLC27A2|RHCG|UQCRC2|AFG3L2|PFKL|BCKDHA|SOD1|BCAM|TIMM44|TINAGL1|ATP6V1A|BHMT|IQGAP2|MUT|ATP6V1B2|ERLIN2|LACTB2|DPYS|CCT5|DLAT|ACAD8|QDPR|GPD1L|HGD|DDAH1|UCHL1|MRPL17|HPD|ATP5O|ACTC1|ATP6V0D1|FTCD|CAPN2|RNPEP|FABP1|ADH5|BDH2|SCIN|HSPA9|COL1A2|ALDH1A1|COL14A1|CRYL1|PRDX3|HPRT1|AMN|CKB|CCT2|TPP1|IDH3A|CDH16|HSP90B1|PPIB|LDHD|ANPEP|PDIA3|CA4|TUBA1C|GPD1|PRODH2|DCXR|HINT1|LETM1|MRPL39|GAA|YWHAG|MRPL13|ADH1B|GLB1|ASL|CYCS|ATP6V0A4|GPD2|ACO1|DPYSL2|PRKACA|RAB1B|ISCU|CSRP2|CYP4A11|AQP1|AOC3|ACAD9|DAB2|GRHPR|TSFM|CRAT|TLN1|ACSS1|CYC1|PIPOX|COX5A|SHMT1|UGDH|PCK1|CHDH|SLC7A8|CALR|RHOT2|TUFM|NDUFV1|PLEC|HSPA5|FBN1|CNDP2|DHRS4|MDH2|EHD3|BGN|ACSM2B|AP2A2|P4HB|C11orf54|IDH2|SERPINA5|RAB11B|MRPL12|PARVA|HSP90AA1|TMLHE|PLS1|PKLR|TUBB4B|CYB5A|RPS2|PRDX6|GNAI1|EPB41L3|PPA2|TPM4|PTGES2|CTSB|PFKM|ALDH3A2|ANXA2|GPX4|ANXA6|UGT1A9|AGK|COL18A1|VAT1|RAC1|STOML2|PBLD|MSN|MYH10|DPP3|DPP4|LONP1|CYB5R3|MT-CO1|PNP|OPA1|MT-CO2|DARS2|KRT19|TGM2|AKR7A3|MARC2|IARS2|CSRP1|ATP1B1|S100A10|LHPP|RTN4IP1|PHGDH|AHCYL1|FLNA|MYO6|ABCD3|GOT1|HOGA1|GNAS|XPNPEP2|ALDH18A1|RBP4|SCP2|SLC25A5|PMPCA|CMPK1|ASS1|VDAC2|YWHAB|RAB14|CISD1|OGDHL|HSPG2|ALDOB|ALDH4A1|ASPN|VARS|AUH|COL4A1|AGMAT|PCCA|FLOT1|KCTD12|ALDH1B1|CUBN|SLC3A2|ACTR2|MAOB|SUCLA2|ESD|POSTN|MT1M|GLRX|HBD|NQO2|HADHA|IDH3B|DUT|PITRM1|PFKP|GLDC|AK3|AMACR|CD9|FECH|GPX3|KRT18|DNM2|DPEP1|NME2|CNP|CORO1B|JUP|PC|SUOX|FHL1|ACTN1|DAK|FLOT2|AK4|MVP|QPRT|SNTB1|UMOD|PDP1|MGST1|TSPO|MYH11|AMPD3|ABAT|ILK|MPST|HAGH|GSTP1|ATP5A1|SEPT7|FKBP1A|CDC42|TXNRD2|RDX|ACY1|FAH|TST|TF|ALAD|SSB|SLC25A12|ARPC1B|IDH1|TKT|NDUFS1|SLC4A4|PDZK1|SELENBP1|CRYZ|PNPT1|GK|ACSF2|HK1|ACTA2|DDC|CKMT2|NDRG1|CKMT1A|GPX1|FBP1|LARS2|GBE1|AOC1|BCS1L|SERPINA1|TGFBI|VWA1|IVD|MME|PCCB|GFM1|SSBP1|ACSL1|NDUFA13|ETFDH|VDAC3|EPHX2|TAGLN|CRYAB|RAP1GAP|IST1|ACADVL|ME3|PHB2|ATP1A1|TIMM50|VIM|MYL6|MGAM|PAH|DNM1L|ALDH6A1|NPC2|ETFA|ITGB3|ACTG1|ACSM2A|RBM8A|GALK1|TTN|SARS2|SIRT5|PHB|PDLIM5|FMO1|PLCG2|AP2B1|HAO2	6.83E-12	GO.0005488	0.93829997
46	16	GO Process	gluconeogenesis	4.6E-10	PCK2|SLC25A11|GAPDH|ENO1|GOT2|GPD1|GPD2|PCK1|MDH2|GOT1|RBP4|ALDOB|PC|SLC25A12|FBP1|SLC25A10	1.35E-11	GO.0006094	0.93400838

55	17	GO Process	aspartate family amino acid metabolic process	4.9E-10	MTHFD1|AHCY|CRYM|GOT2|HRSP12|BHMT2|BHMT|MSRA|PIPOX|DLST|PHGDH|AHCYL1|GOT1|ASS1|NIT2|SMS|ALDH7A1	1.47E-11	GO.0009066	0.93062731
96	21	GO Process	detoxification	5.6E-10	GSTZ1|GSTM3|PRDX1|NNT|PRDX5|SOD1|FABP1|ADH5|PRDX3|GSTA1|PRDX6|GPX4|MARC2|MGST3|GSTO1|ESD|GPX3|MGST1|GSTP1|TXNRD2|GPX1	1.67E-11	GO.0098754	0.92525882
290	35	GO Process	alcohol metabolic process	6.2E-10	ACO2|IDH3G|SPR|AKR7A2|TTR|ALDH2|SORD|ERLIN2|QDPR|ADH5|ALDH1A1|GPD1|ADH1B|GPD2|ACSS1|PCK1|DHRS4|IDH2|ALDH3A2|CYB5R3|HMGCS2|GOT1|RBP4|SCP2|ALDH1B1|CUBN|AKR1C3|IDH3B|DAK|IDH1|RDH13|GK|NPC2|GALK1|PLCG2	1.88E-11	GO.0006066	0.9205512
87	20	GO Process	neurotransmitter metabolic process	7.8E-10	AGXT2|SPR|HRSP12|DMGDH|AMT|BHMT|DPYS|HNMT|SHMT1|CHDH|ALDH9A1|PHGDH|SARDH|MAOB|GLDC|ABAT|ALDH7A1|DDC|PAH|GLYAT	2.35E-11	GO.0042133	0.91106983
33	9	KEGG Pathways	Fructose and mannose metabolism	1.7E-05	KHK|SORD|PFKL|PFKM|ALDOB|PFKP|DAK|HK1|FBP1	2.34E-06	hsa00051	0.47594508
5163	226	GO Process	cellular component organization	1E-09	RALA|DCN|NDUFB4|VCL|MAPK1|MYH9|NID2|DECR1|PPIF|PFN1|C1QBP|NAGLU|VTN|CTSC|GAPDH|RAB35|SKP1|EHHADH|TTR|DSTN|TUBA4A|VIL1|PMPCB|MTX2|NDUFA2|ACTN4|NDUFA10|LAMA5|APCS|LAMC1|MRPL15|ADAM10|PSMD11|ATP5B|CHCHD3|SLC9A3R1|EHD2|NAPA|LRP2|ATP6V0A1|ADD1|VCAN|CPT1A|EIF4H|MTFP1|NDUFA9|LUM|SLC27A2|UQCRC2|AFG3L2|PFKL|SOD1|ECSIT|TIMM44|IQGAP2|DPYS|NDUFB9|MRPL49|SLC25A4|UCHL1|MRPL17|ATP5O|ACTC1|ATP6V0D1|ATP5J2|SCIN|HSPA9|COL1A2|COL14A1|PRDX3|HPRT1|NDUFB8|CCT2|TPP1|HSP90B1|ATP5L|TUBA1C|ATP5H|DCXR|LETM1|MRPL39|GAA|ATP5I|YWHAG|MRPL13|CYCS|ATP6V0A4|DPYSL2|C19orf70|PRKACA|RAB1B|ISCU|AQP1|ACAD9|DAB2|GRHPR|CRAT|TLN1|PIPOX|SHMT1|TMEM11|CALR|RHOT2|NDUFV1|PLEC|FBN1|DHRS4|EHD3|BGN|AP2A2|P4HB|NDUFA12|UQCR10|SERPINA5|ACAA1|MRPL12|PARVA|HSP90AA1|PLS1|TUBB4B|EPB41L3|TPSAB1|TPM4|SAMM50|PFKM|ANXA2|GPX4|ANXA6|AGK|COL18A1|RAC1|STOML2|ATP5C1|MSN|MYH10|COL4A2|LONP1|MRPL37|MRPL21|OPA1|COA3|KRT19|TGM2|IBA57|ATP1B1|DPT|S100A10|PHGDH|ATP5F1|FLNA|ABCD3|SCP2|SLC25A5|PMPCA|YWHAB|APOOL|RAB14|CLIC4|HSPG2|ALDOB|COL4A1|FLOT1|KCTD12|ACTR2|APOO|POSTN|MPV17|PITRM1|SLC25A6|AMACR|CD9|GPX3|KRT18|DNM2|CNP|CORO1B|JUP|ACTN1|FLOT2|QPRT|MGST1|TSPO|MYH11|ILK|ATP5A1|SEPT7|FKBP1A|CDC42|RDX|TF|ALAD|IMMT|ARPC1B|IDH1|NDUFS1|CRYZ|PNPT1|NDRG1|COX20|FBP1|TOMM40|BCS1L|SERPINA1|TGFBI|VWA1|ATPAF2|MRPS22|TIMMDC1|SSBP1|NDUFA13|MRPS30|EPHX2|CRYAB|RAP1GAP|IST1|PHB2|TIMM50|VIM|DNM1L|ITGB3|ACTG1|TTN|SIRT5|PHB|AP2B1|HAO2	3.09E-11	GO.0016043	0.89956786
880	43	GO Component	supramolecular polymer	0.0053	DCN|VCL|ENO1|TUBA4A|HSPB1|ACTN4|LRPPRC|LUM|IQGAP2|CCT5|ACTC1|COL1A2|CCT2|TUBA1C|DCXR|PLEC|FBN1|PARVA|PLS1|TUBB4B|TPM4|COL4A2|KRT19|FLNA|MYO6|COL4A1|KRT18|DNM2|CNP|CORO1B|JUP|ACTN1|MYH11|ILK|FKBP1A|RMDN1|ACTA2|NDRG1|CRYAB|VIM|DNM1L|ACTG1|TTN	0.0012	GO.0099081	0.22757241
59	17	GO Process	monosaccharide catabolic process	1.2E-09	GAPDH|ENO1|KHK|SORD|PFKL|ALDH1A1|CRYL1|DCXR|GLB1|PKLR|PFKM|AKR1A1|ALDOB|PFKP|DAK|HK1|GALK1	3.72E-11	GO.0046365	0.89208188
968	70	GO Function	protein-containing complex binding	1.3E-09	DCN|UQCRC1|MYH9|NID2|C1QBP|VTN|SLC25A3|ATP6V1B1|DSTN|VIL1|ACTN4|LAMA5|LGALS3|ADAM10|SLC9A3R1|NAPA|CD81|ADD1|AMBP|EIF4H|NDUFA9|LUM|UQCRC2|IQGAP2|ATP6V0D1|SCIN|COL14A1|PPIB|LETM1|TLN1|CALR|HSPA5|FBN1|P4HB|PLS1|GNAI1|TPM4|CTSB|MYH10|KRT19|FLNA|MYO6|GNAS|YWHAB|HSPG2|ASPN|HADHA|PFKP|CD9|DNM2|CORO1B|ACTN1|UMOD|MYH11|ILK|FKBP1A|ALAD|ARPC1B|PDZK1|AOC1|TGFBI|TAGLN|CRYAB|IST1|TIMM50|VIM|DNM1L|ITGB3|TTN|AP2B1	2.21E-11	GO.0044877	0.889279
90	20	GO Process	cellular detoxification	1.3E-09	GSTZ1|GSTM3|PRDX1|NNT|PRDX5|SOD1|FABP1|ADH5|PRDX3|GSTA1|PRDX6|GPX4|MGST3|GSTO1|ESD|GPX3|MGST1|GSTP1|TXNRD2|GPX1	3.98E-11	GO.1990748	0.889279

27	13	GO Process	ATP synthesis coupled proton transport	1.5E-09	ATP5B|ATP6V0A1|ATP5O|ATP5J2|ATP5L|ATP5H|ATP5I|ATP6V0A4|CYC1|STOML2|ATP5C1|ATP5F1|ATP5A1	4.53E-11	GO.0015986	0.8838632
497	46	GO Process	peptide metabolic process	1.8E-09	GSTZ1|CTSH|LTA4H|GSTM3|CARS2|MRPL15|ENPEP|EIF4H|SOD1|NAT8|MRPL49|MRPL17|RNPEP|BDH2|TPP1|ANPEP|MRPL39|MRPL13|TSFM|TUFM|CNDP2|MRPL12|HSP90AA1|GSTA1|RPS2|PPA2|MRPL37|MRPL21|DARS2|IARS2|GSTO1|VARS|DPEP1|HAGH|GGT5|GSTP1|GGT1|IDH1|GPX1|LARS2|MRPS22|MME|GFM1|GSTA2|MRPS30|SARS2	5.64E-11	GO.0006518	0.874958
2163	119	GO Function	carbohydrate derivative binding	2.4E-09	RALA|DCN|MAPK1|RTCB|MYH9|MTHFD1|PCK2|IDH3G|PNPO|C1QBP|VTN|RAB35|LTF|ATP6V1B1|TRAP1|TUBA4A|VIL1|ACTN4|CARS2|KHK|PEBP1|ATP5B|EHD2|VCAN|SLC27A2|AFG3L2|PFKL|TIMM44|ATP6V1A|ATP6V1B2|CCT5|ACTC1|HSPA9|CKB|CCT2|HSP90B1|TUBA1C|GLB1|PRKACA|RAB1B|ACSS1|PCK1|RHOT2|TUFM|NDUFV1|HSPA5|FBN1|EHD3|BGN|ACSM2B|SERPINA5|RAB11B|HSP90AA1|PKLR|TUBB4B|GNAI1|CTSB|PFKM|ANXA6|AGK|RAC1|MYH10|LONP1|CYB5R3|PNP|OPA1|DARS2|TGM2|IARS2|MYO6|ABCD3|GNAS|ALDH18A1|SCP2|CMPK1|ASS1|RAB14|VARS|PCCA|ACTR2|SUCLA2|POSTN|HADHA|PFKP|AK3|DNM2|DPEP1|NME2|PC|DAK|AK4|MYH11|ILK|ATP5A1|SEPT7|CDC42|GK|ACSF2|HK1|ACTA2|CKMT2|CKMT1A|FBP1|LARS2|AOC1|BCS1L|PCCB|GFM1|ACSL1|NDUFA13|ATP1A1|DNM1L|ALDH6A1|ACTG1|ACSM2A|GALK1|TTN|SARS2|HAO2	4.41E-11	GO.0097367	0.86143937
128	42	GO Component	inner mitochondrial membrane protein complex	4.7E-26	NDUFB4|UQCRC1|PMPCB|NDUFA2|NDUFA10|ATP5B|CHCHD3|SDHA|NDUFA9|UQCRC2|AFG3L2|NDUFB9|ATP5O|ATP5J2|NDUFB8|ATP5L|ATP5H|ATP5I|C19orf70|UQCRH|CYC1|COX5A|NDUFV1|NDUFA12|UQCR10|AGK|ATP5C1|MT-CO1|MT-CO2|ATP5F1|USMG5|APOOL|APOO|SLC25A6|ATP5A1|IMMT|NDUFS1|BCS1L|NDUFA13|COX7C|TIMM50|COX4I1	1.62E-27	GO.0098800	2.53242217
31	8	KEGG Pathways	Galactose metabolism	7.8E-05	PFKL|GAA|GLB1|PFKM|PFKP|HK1|MGAM|GALK1	1.21E-05	hsa00052	0.41079054
625	52	GO Process	carbohydrate derivative biosynthetic process	3.5E-09	DCN|NANS|OGDH|PDHX|GAPDH|ENO1|ATP5B|ATP6V0A1|VCAN|LUM|SORD|PFKL|DLAT|ATP5O|ATP5J2|CRYL1|HPRT1|ATP5L|ATP5H|DCXR|ATP5I|PDHB|ATP6V0A4|AQP1|GBAS|ACSS1|CYC1|SHMT1|UGDH|BGN|PKLR|PFKM|STOML2|ATP5C1|MPC1|ATP5F1|CMPK1|AKR1A1|OGDHL|HSPG2|ALDOB|PDHA1|DUT|PFKP|NME2|AMPD3|ATP5A1|TKT|GK|HK1|ACSL1|GALK1	1.11E-10	GO.1901137	0.84584208
17	11	GO Process	mitochondrial ATP synthesis coupled proton transport	4.2E-09	ATP5B|ATP5O|ATP5J2|ATP5L|ATP5H|ATP5I|CYC1|STOML2|ATP5C1|ATP5F1|ATP5A1	1.34E-10	GO.0042776	0.83788237
873	41	GO Component	supramolecular fiber	0.0121	DCN|VCL|ENO1|TUBA4A|HSPB1|ACTN4|LRPPRC|LUM|IQGAP2|CCT5|ACTC1|COL1A2|CCT2|TUBA1C|DCXR|PLEC|FBN1|PARVA|PLS1|TUBB4B|TPM4|KRT19|FLNA|MYO6|KRT18|DNM2|CNP|CORO1B|JUP|ACTN1|MYH11|ILK|FKBP1A|RMDN1|ACTA2|NDRG1|CRYAB|VIM|DNM1L|ACTG1|TTN	0.0032	GO.0099512	0.19172146
670	40	GO Component	perinuclear region of cytoplasm	0.00024	GAPDH|ACTN4|ADAM10|LRPPRC|SLC9A3R1|EHD2|PRDX5|EIF4H|CAPN2|HSP90B1|PPIB|CA4|GLB1|PRKACA|CALR|EHD3|HSP90AA1|PRDX6|PTGES2|CTSB|ANXA2|ANXA6|MSN|FLNA|MYO6|GNAS|YWHAB|RAB14|CLIC4|CLIC1|MT1M|KRT18|DNM2|CNP|CORO1B|FLOT2|MVP|TF|NDRG1|DNM1L	3.97E-05	GO.0048471	0.36197888
81	18	KEGG Pathways	Peroxisome	4.9E-09	EHHADH|PRDX1|PRDX5|SLC27A2|SOD1|CRAT|PIPOX|DHRS4|IDH2|ACAA1|ABCD3|SCP2|MPV17|AMACR|IDH1|ACSL1|EPHX2|HAO2	3.78E-10	hsa04146	0.83133637
724	38	GO Component	cytoplasmic vesicle membrane	0.0032	RALA|PGRMC1|MLEC|RAB35|ATP6V1B1|LGALS3|ADAM10|LRP2|ATP6V0A1|SLC27A2|IQGAP2|ATP6V0D2|ATP6V0D1|ANPEP|CA4|GAA|ATP6V0A4|DAB2|CALR|AP2A2|SERPINA5|RAB11B|RAC1|PIGR|MYO6|RAB14|CLIC4|CD9|DNM2|PRCP|FLOT2|MGST1|TF|MME|MGAM|DNM1L|ITGB3|AP2B1	0.00069	GO.0030659	0.249485

77	18	GO Process	cellular amino acid biosynthetic process	5.8E-09	MTHFD1|AHCY|AGXT2|GOT2|BHMT2|BHMT|ASL|SHMT1|BCAT2|PHGDH|GOT1|ALDH18A1|ASS1|PSAT1|ABAT|GATM|GGT1|PAH	1.86E-10	GO.0008652	0.82388242
101	20	GO Process	nucleotide catabolic process	7.4E-09	OGDH|GAPDH|DNPH1|ENO1|GDA|PFKL|HPRT1|HINT1|PKLR|PFKM|PNP|OGDHL|ALDOB|DUT|PFKP|CNP|AMPD3|HK1|GPX1|GALK1	2.39E-10	GO.0009166	0.81331222
152	24	GO Process	organophosphate catabolic process	7.5E-09	OGDH|GAPDH|DNPH1|ENO1|GDA|PFKL|GPD1L|HPRT1|GPD1|HINT1|PKLR|PRDX6|PFKM|PNP|OGDHL|ALDOB|DUT|PFKP|CNP|AMPD3|HK1|GPX1|GALK1|PLCG2	2.43E-10	GO.0046434	0.81278437
370	37	GO Component	apical part of cell	7.7E-09	REEP6|ATP6V1B1|SLC9A3R1|LRP2|ENPEP|RHCG|ATP6V1A|ATP6V0D2|ATP6V0D1|FABP1|AMN|CA4|SLC23A1|ATP6V0A4|CYP4A11|AQP1|PFKM|GM2A|MSN|DPP4|ATP1B1|AHCYL1|MYO6|GNAS|AKR1A1|CLIC4|CUBN|SLC3A2|CD9|DPEP1|UMOD|MGST1|RDX|TF|PDZK1|ATP1A1|MGAM	6.57E-10	GO.0045177	0.81163386
690	36	GO Component	cell surface	0.0046	RALA|LGALS1|C1QBP|LTF|ENO1|GOT2|ADAM10|ERP29|ATP5B|LRP2|AMBP|ENPEP|BCAM|IQGAP2|ANPEP|PDIA3|CA4|AOC3|TLN1|CALR|HSPA5|BGN|P4HB|SERPINA5|ANXA2|MSN|DPP4|CLIC4|CUBN|SLC3A2|CD9|TF|CRYAB|PHB2|ITGB3|PHB	0.0011	GO.0009986	0.23372422
60	16	GO Process	alpha-amino acid biosynthetic process	1.1E-08	MTHFD1|AHCY|AGXT2|GOT2|BHMT2|BHMT|ASL|SHMT1|BCAT2|PHGDH|GOT1|ALDH18A1|ASS1|PSAT1|GATM|GGT1	3.69E-10	GO.1901607	0.79469216
491	44	GO Process	response to inorganic substance	1.2E-08	MAPK1|PPIF|GGH|KHK|PRDX1|ADD1|SORD|SOD1|QDPR|FABP1|PRDX3|ACO1|AQP1|CALR|HSPA5|PKLR|CYB5A|COL18A1|LONP1|MT-CO1|AHCYL1|GOT1|ASS1|CLIC4|MAOB|MT1M|AKR1C3|CD9|FECH|DNM2|DPEP1|TSPO|ABAT|TXNRD2|TF|ALAD|SLC25A12|NDRG1|GPX1|FBP1|AOC1|CRYAB|ACTG1|TTN	3.86E-10	GO.0010035	0.79318141
34	13	GO Process	NADH metabolic process	1.3E-08	OGDH|GAPDH|ENO1|PFKL|GPD1L|GPD1|GPD2|MDH2|PKLR|PFKM|ALDOB|PFKP|HK1	4.4E-10	GO.0006734	0.78761484
830	61	GO Function	protein homodimerization activity	1.4E-08	OXCT1|LGALS1|MYH9|GSTZ1|CRYM|PNPO|DNPH1|ENO1|GOT2|VIL1|HSPB1|ACTN4|HRSP12|GSTM3|ADAM10|ERP29|ADD1|AMBP|SOD1|MUT|QDPR|GPD1L|ADH5|CRYL1|HPRT1|GPD1|GLB1|AOC3|GRHPR|SHMT1|HSP90AA1|PRDX6|TPM4|PFKM|ALDH3A2|ANXA6|UGT1A9|DPP4|DARS2|S100A10|LHPP|FLNA|ABCD3|HOGA1|CUBN|MAOB|NQO2|GLDC|JUP|ACTN1|QPRT|MGST1|ABAT|RDX|IDH1|TKT|AOC1|EPHX2|CRYAB|RAP1GAP|DNM1L	2.68E-10	GO.0042803	0.78507809
5342	227	GO Process	cellular component organization or biogenesis	1.4E-08	RALA|DCN|NDUFB4|VCL|MAPK1|MYH9|NID2|DECR1|PPIF|PFN1|C1QBP|NAGLU|VTN|CTSC|GAPDH|RAB35|SKP1|EHHADH|TTR|DSTN|TUBA4A|VIL1|PMPCB|MTX2|NDUFA2|ACTN4|NDUFA10|LAMA5|APCS|LAMC1|MRPL15|ADAM10|PSMD11|ATP5B|CHCHD3|SLC9A3R1|EHD2|NAPA|LRP2|ATP6V0A1|ADD1|VCAN|CPT1A|EIF4H|MTFP1|NDUFA9|LUM|SLC27A2|UQCRC2|AFG3L2|PFKL|SOD1|ECSIT|TIMM44|IQGAP2|DPYS|NDUFB9|MRPL49|SLC25A4|UCHL1|MRPL17|ATP5O|ACTC1|ATP6V0D1|ATP5J2|SCIN|HSPA9|COL1A2|COL14A1|PRDX3|HPRT1|NDUFB8|CCT2|TPP1|HSP90B1|ATP5L|TUBA1C|ATP5H|DCXR|LETM1|MRPL39|GAA|ATP5I|YWHAG|MRPL13|CYCS|ATP6V0A4|DPYSL2|C19orf70|PRKACA|RAB1B|ISCU|AQP1|ACAD9|DAB2|GRHPR|CRAT|TLN1|PIPOX|SHMT1|TMEM11|CALR|RHOT2|NDUFV1|PLEC|FBN1|DHRS4|EHD3|BGN|AP2A2|P4HB|NDUFA12|UQCR10|SERPINA5|ACAA1|MRPL12|PARVA|HSP90AA1|PLS1|TUBB4B|RPS2|EPB41L3|TPSAB1|TPM4|SAMM50|PFKM|ANXA2|GPX4|ANXA6|AGK|COL18A1|RAC1|STOML2|ATP5C1|MSN|MYH10|COL4A2|LONP1|MRPL37|MRPL21|OPA1|COA3|KRT19|TGM2|IBA57|ATP1B1|DPT|S100A10|PHGDH|ATP5F1|FLNA|ABCD3|SCP2|SLC25A5|PMPCA|YWHAB|APOOL|RAB14|CLIC4|HSPG2|ALDOB|COL4A1|FLOT1|KCTD12|ACTR2|APOO|POSTN|MPV17|PITRM1|SLC25A6|AMACR|CD9|GPX3|KRT18|DNM2|CNP|CORO1B|JUP|ACTN1|FLOT2|QPRT|MGST1|TSPO|MYH11|ILK|ATP5A1|SEPT7|FKBP1A|CDC42|RDX|TF|ALAD|IMMT|ARPC1B|IDH1|NDUFS1|CRYZ|PNPT1|NDRG1|COX20|FBP1|TOMM40|BCS1L|SERPINA1|TGFBI|VWA1|ATPAF2|MRPS22|TIMMDC1|SSBP1|NDUFA13|MRPS30|EPHX2|CRYAB|RAP1GAP|IST1|PHB2|TIMM50|VIM|DNM1L|ITGB3|ACTG1|TTN|SIRT5|PHB|AP2B1|HAO2	4.73E-10	GO.0071840	0.78477117

72	17	GO Process	pyridine-containing compound biosynthetic process	1.6E-08	OGDH|PNPO|GAPDH|ENO1|PFKL|IDH2|PKLR|PFKM|PNP|OGDHL|ALDOB|PSAT1|PFKP|QPRT|NAPRT|HK1|GALK1	5.22E-10	GO.0072525	0.78096683
52	15	GO Process	glutathione metabolic process	1.6E-08	GSTZ1|GSTM3|SOD1|NAT8|CNDP2|GSTA1|GSTO1|DPEP1|HAGH|GGT5|GSTP1|GGT1|IDH1|GPX1|GSTA2	5.24E-10	GO.0006749	0.78096683
120	21	GO Process	carbohydrate biosynthetic process	1.8E-08	NANS|PCK2|SLC25A11|GAPDH|ENO1|GOT2|SORD|GPD1|GPD2|PCK1|MDH2|GOT1|RBP4|AKR1A1|ALDOB|PC|SLC25A12|TKT|FBP1|GBE1|SLC25A10	6.24E-10	GO.0016051	0.77375489
103	34	GO Component	oxidoreductase complex	4.1E-21	NDUFB4|UQCRC1|OGDH|PDHX|PMPCB|NDUFA2|NDUFA10|SDHA|NDUFA9|UQCRC2|BCKDHA|NDUFB9|DLAT|GPD1L|NDUFB8|GPD1|PDHB|GPD2|UQCRH|CYC1|BCKDHB|NDUFV1|P4HB|NDUFA12|UQCR10|DLST|MT-CO1|OGDHL|PDHA1|GLDC|NDUFS1|BCS1L|NDUFA13|ETFDH	1.46E-22	GO.1990204	2.03882767
1427	86	GO Process	response to oxygen-containing compound	1.9E-08	DCN|OXCT1|MAPK1|LGALS1|CTSH|PPIF|ATP6V1B1|GOT2|GGH|KHK|PRDX1|ATP6V0A1|PRDX5|CPT1A|PFKL|SOD1|ATP6V1A|ATP6V1B2|QDPR|ATP6V0D2|ACTC1|ATP6V0D1|CAPN2|FABP1|ADH5|COL1A2|PRDX3|HSP90B1|GPD1|YWHAG|GLB1|ATP6V0A4|PRKACA|AQP1|CYC1|SHMT1|PCK1|CALR|TUFM|HSPA5|FBN1|RAB11B|PKLR|GNAI1|COL18A1|MSN|OPA1|AHCYL1|GOT1|GNAS|RBP4|ASS1|COL4A1|ACTR2|MAOB|ESD|POSTN|MPV17|AKR1C3|HADHA|GLDC|CD9|FECH|GPX3|DNM2|DPEP1|CNP|JUP|MGST1|TSPO|ABAT|GSTP1|TXNRD2|ALAD|ARPC1B|PNPT1|GPX1|AOC1|ACSL1|NDUFA13|CRYAB|PHB2|ATP1A1|DNM1L|FMO1|PLCG2	6.46E-10	GO.1901700	0.77258422
1710	98	GO Function	drug binding	2.2E-08	MAPK1|RTCB|MYH9|MTHFD1|IDH3G|PPIF|PNPO|AGXT2|ATP6V1B1|GOT2|TRAP1|CARS2|KHK|PEBP1|ATP5B|EHD2|LRP2|SLC27A2|AFG3L2|PFKL|TIMM44|ATP6V1A|MUT|ATP6V1B2|DPYS|CCT5|ATP5O|ACTC1|FTCD|FABP1|HSPA9|CKB|CCT2|HSP90B1|PPIB|PRKACA|ACSS1|SHMT1|HSPA5|EHD3|ACSM2B|HSP90AA1|PKLR|PFKM|AGK|MYH10|LONP1|CYB5R3|PNP|DARS2|MARC2|IARS2|MYO6|ABCD3|GOT1|ALDH18A1|CMPK1|ASS1|VARS|PCCA|CUBN|ACTR2|SUCLA2|HBD|NQO2|PFKP|GLDC|AK3|NME2|PC|DAK|AK4|MYH11|ABAT|ILK|GSTP1|ATP5A1|FKBP1A|GK|ACSF2|HK1|ACTA2|DDC|CKMT2|CKMT1A|FBP1|LARS2|AOC1|BCS1L|PCCB|ACSL1|NDUFA13|ATP1A1|ACTG1|ACSM2A|GALK1|TTN|SARS2	4.42E-10	GO.0008144	0.76516951
432	34	GO Component	actin cytoskeleton	5.4E-06	VCL|MYH9|DSTN|VIL1|ACTN4|SLC9A3R1|ADD1|IQGAP2|ACTC1|CAPN2|SCIN|MSRA|PLS1|TPM4|MYH10|KRT19|FLNA|MYO6|CLIC4|ACTR2|CORO1B|JUP|ACTN1|MYH11|ILK|SEPT7|RDX|ARPC1B|ACTA2|CRYAB|MYL6|ACTG1|TTN|PDLIM5	6.78E-07	GO.0015629	0.52692177
900	63	GO Process	response to drug	2.4E-08	OXCT1|MAPK1|LGALS1|PPIF|GOT2|GGH|LRP2|CPT1A|SORD|SOD1|NAT8|QDPR|SLC25A4|ACTC1|FABP1|PRDX3|HPRT1|AMN|HSP90B1|AQP1|CALR|TUFM|HSPA5|PKLR|SLC25A15|GNAI1|COL18A1|PNP|OPA1|S100A10|MYO6|ABCD3|GNAS|RBP4|SLC25A5|ASS1|CUBN|ACTR2|MAOB|HBD|HADHA|SLC25A6|FECH|DNM2|DPEP1|JUP|AK4|MGST1|TSPO|ABAT|GSTP1|PTGR1|ALAD|SLC25A12|PDZK1|DDC|GPX1|FBP1|AOC1|ACSL1|CRYAB|ATP1A1|SLC25A10	8.28E-10	GO.0042493	0.76197888
106	20	GO Function	exopeptidase activity	2.4E-08	CTSH|LTA4H|PEPD|GGH|ENPEP|UCHL1|RNPEP|TPP1|ANPEP|CNDP2|DPP3|DPP4|XPNPEP2|DPEP1|PRCP|GGT5|GGT1|CPVL|SCRN1|MME	5.03E-10	GO.0008238	0.76126102

7824	304	GO Process	response to stimulus	2.5E-08	RALA|DCN|NDUFB4|OXCT1|UQCRC1|VCL|MAPK1|LGALS1|MYH9|ACO2|GSTZ1|AHCY|PGRMC1|F9|CTSH|PLGRKT|PPIF|PFN1|C1QBP|VTN|CTSC|MLEC|LTA4H|GAPDH|RAB35|SKP1|LTF|REEP6|ATP6V1B1|ENO1|AKR7A2|CTSD|TTR|GOT2|TRAP1|CANX|VIL1|HSPB1|ACTN4|LAMA5|LGALS3|HRSP12|APCS|GSTM3|TINAG|MRPL15|GGH|ADAM10|KHK|LYZ|PEBP1|PSMD11|COTL1|SLC9A3R1|PRDX1|EHD2|CD81|LRP2|PSMD3|ATP6V0A1|NNT|ADD1|F13A1|CALB1|PRDX5|CPT1A|MTFP1|LUM|SORD|SLC27A2|AFG3L2|PFKL|SOD1|BCAM|ECSIT|TINAGL1|NAT8|ATP6V1A|IQGAP2|ATP6V1B2|ERLIN2|CCT5|QDPR|SLC25A4|DDAH1|UCHL1|ATP6V0D2|ACTC1|ATP6V0D1|CAPN2|FABP1|ADH5|CMBL|HSPA9|COL1A2|PRDX3|HPRT1|AMN|CCT2|TPP1|HSP90B1|ANPEP|PDIA3|GPD1|SLC23A1|HINT1|GAA|YWHAG|GLB1|CYCS|ATP6V0A4|ACO1|DPYSL2|PRKACA|AQP1|AOC3|DAB2|MSRA|TLN1|CYC1|CES2|SHMT1|PCK1|SLC7A8|CALR|RHOT2|TUFM|BCAT2|HSPA5|FBN1|EHD3|BGN|ACSM2B|AP2A2|P4HB|NDUFA12|SERPINA5|RAB11B|ACAA1|PARVA|HSP90AA1|GSTA1|PKLR|TUBB4B|CYB5A|PRDX6|SLC25A15|GNAI1|TPSAB1|PTGES2|CTSB|ANXA2|GPX4|ANXA6|UGT1A9|COL18A1|VAT1|RAC1|STOML2|PIGR|GM2A|MSN|MYH10|COL4A2|DPP4|LONP1|MPC1|CYB5R3|MT-CO1|PNP|OPA1|MT-CO2|KRT19|TGM2|AKR7A3|MARC2|C4BPA|ATP1B1|MGST3|S100A10|PHGDH|GSTO1|AHCYL1|FLNA|MYO6|ABCD3|GOT1|GNAS|RBP4|SLC25A5|ASS1|YWHAB|RAB14|CLIC4|HSPG2|ASPN|CLIC1|COL4A1|FLOT1|CUBN|SLC3A2|RSU1|ACTR2|MAOB|ESD|POSTN|TXNDC5|MT1M|MPV17|HBD|BPHL|NQO2|AKR1C3|HADHA|PFKP|GLDC|SLC25A6|AK3|CD9|FECH|GPX3|KRT18|DNM2|DPEP1|NME2|PRCP|CNP|CORO1B|JUP|NIT2|DAK|AK4|MVP|UMOD|MGST1|TSPO|AMPD3|ABAT|GATM|ILK|MPST|GGT5|GSTP1|FKBP1A|CDC42|GGT1|TXNRD2|RDX|ACY1|IFI30|PDIA6|PTGR1|TF|ALAD|SLC25A12|ARPC1B|IDH1|RDH13|AHSG|PDZK1|HIGD1A|CRYZ|PNPT1|NAPRT|ACTA2|DDC|NDRG1|GPX1|FBP1|AOC1|SERPINA1|TGFBI|VWA1|MME|GSTA2|ACSL1|NDUFA13|ETFDH|VDAC3|EPHX2|CRYAB|RAP1GAP|IST1|ACADVL|PHB2|ATP1A1|TIMM50|VIM|SLC25A10|MGAM|METTL7A|DNM1L|NPC2|ITGB3|COX4I1|ACTG1|TTN|SIRT5|PHB|EPHX1|FMO1|PLCG2|AP2B1|GLYAT	8.61E-10	GO.0050896	0.76055483
1292	80	GO Process	ion transport	2.5E-08	SLC25A11|SLC25A3|LTF|ATP6V1B1|GOT2|PMPCB|ATP5B|SLC9A3R1|LRP2|ATP6V0A1|NNT|VDAC1|CPT1A|NDUFA9|SLC27A2|RHCG|AFG3L2|ATP6V1A|ATP6V1B2|SLC25A4|ATP6V0D2|ATP5O|ATP6V0D1|ATP5J2|COX7A1|FABP1|ATP5L|CA4|ATP5H|SLC23A1|LETM1|ATP5I|ATP6V0A4|AQP1|CYC1|COX5A|SLC7A8|SLC5A2|RAB11B|CYB5A|SLC25A15|ANXA6|STOML2|ATP5C1|MPC1|MT-CO1|OPA1|MT-CO2|ATP1B1|ATP5F1|SFXN2|ABCD3|SCP2|SLC25A5|PMPCA|VDAC2|CLIC4|CLIC1|SLC3A2|SLC25A6|DNM2|TSPO|ATP5A1|FKBP1A|TF|PCYOX1|SLC25A12|SLC4A4|PDZK1|TOMM40|ACSL1|COX7C|VDAC3|PHB2|ATP1A1|SLC25A10|NPC2|COX4I1|PHB|PLCG2	8.69E-10	GO.0006811	0.76038007
339	35	GO Process	response to metal ion	2.5E-08	MAPK1|PPIF|GGH|KHK|ADD1|SORD|SOD1|QDPR|ACO1|AQP1|CALR|HSPA5|PKLR|CYB5A|LONP1|MT-CO1|AHCYL1|GOT1|ASS1|CLIC4|MAOB|MT1M|AKR1C3|FECH|DPEP1|TSPO|ABAT|TF|ALAD|SLC25A12|NDRG1|FBP1|AOC1|ACTG1|TTN	8.92E-10	GO.0010038	0.75951663
44	12	KEGG Pathways	Cysteine and methionine metabolism	4.7E-07	AHCY|AGXT2|GOT2|BHMT2|BHMT|BCAT2|MDH2|AHCYL1|GOT1|MPST|SMS|TST	4.82E-08	hsa00270	0.63279021
66	16	GO Process	NAD metabolic process	3.4E-08	OGDH|GAPDH|ENO1|PFKL|GPD1L|GPD1|GPD2|MDH2|PKLR|PFKM|PNP|ALDOB|PFKP|QPRT|NAPRT|HK1	1.21E-09	GO.0019674	0.74634416
172	32	GO Component	vacuolar lumen	7.5E-14	DCN|MAPK1|NAGLU|CTSC|CTSD|TTR|GGH|LYZ|VCAN|LUM|CCT2|TPP1|GAA|GLB1|BGN|HSP90AA1|TUBB4B|PRDX6|PTGES2|CTSB|ANXA2|VAT1|GM2A|CYB5R3|HSPG2|CUBN|ACTR2|TXNDC5|IFI30|NAPRT|IST1|NPC2	4.33E-15	GO.0005775	1.31237822

109	20	GO Function	oxidoreductase activity, acting on NAD(P)H	3.6E-08	NDUFB4|DECR1|NDUFA2|NDUFA10|NNT|NDUFA9|ECSIT|NDUFB9|PRDX3|NDUFB8|DCXR|NDUFV1|DHRS4|NDUFA12|CYB5R3|AKR1C3|TXNRD2|NDUFS1|CRYZ|NDUFA13	7.73E-10	GO.0016651	0.74436975
1235	77	GO Process	transmembrane transport	4E-08	SLC25A11|SLC25A3|ATP6V1B1|PMPCB|ATP5B|LRP2|ATP6V0A1|NNT|ADD1|VDAC1|CPT1A|SLC27A2|RHCG|AFG3L2|TIMM44|ATP6V1A|ATP6V1B2|ERLIN2|SLC25A4|ATP6V0D2|ATP5O|ATP6V0D1|ATP5J2|COX7A1|ATP5L|ATP5H|SLC23A1|LETM1|ATP5I|ATP6V0A4|AQP1|CYC1|COX5A|SLC7A8|SLC5A2|CYB5A|SLC25A15|SLC25A40|ANXA6|AGK|STOML2|ATP5C1|MPC1|MT-CO1|OPA1|MT-CO2|ATP1B1|ATP5F1|SFXN2|ABCD3|RBP4|SLC25A5|PMPCA|VDAC2|CLIC4|CLIC1|SLC3A2|SLC25A6|ATP5A1|FKBP1A|TST|TF|PCYOX1|SLC25A12|SLC4A4|PNPT1|TOMM40|NDUFA13|COX7C|VDAC3|PHB2|ATP1A1|TIMM50|SLC25A10|COX4I1|PHB|PLCG2	1.41E-09	GO.0055085	0.74023048
299	32	GO Component	endoplasmic reticulum lumen	2.3E-08	LGALS1|F9|VTN|CTSC|CANX|LAMC1|ADAM10|ERP29|VCAN|SLC27A2|COL1A2|COL14A1|HSP90B1|PPIB|PDIA3|CES2|CALR|HSPA5|FBN1|P4HB|COL18A1|ITIH2|COL4A2|COL4A1|ESD|TXNDC5|PDIA6|TF|AHSG|SERPINA1|VWA1|FMO1	2.14E-09	GO.0005788	0.76401645
245	29	GO Process	lipid modification	4.3E-08	DECR1|EHHADH|ACADS|MECR|ACAD11|CPT1A|SLC27A2|BDH2|ECI1|CRAT|HADHB|ACAA1|ALDH3A2|AGK|ECHS1|ABCD3|SCP2|ECHDC2|AUH|HADHA|AMACR|SACM1L|IVD|ETFDH|EPHX2|ECHDC1|ACADVL|ETFA|HAO2	1.55E-09	GO.0030258	0.73635121
307	32	GO Component	apical plasma membrane	4E-08	ATP6V1B1|SLC9A3R1|LRP2|ENPEP|RHCG|ATP6V1A|ATP6V0D2|ATP6V0D1|AMN|CA4|SLC23A1|ATP6V0A4|CYP4A11|AQP1|PFKM|GM2A|MSN|DPP4|ATP1B1|AHCYL1|GNAS|AKR1A1|CUBN|SLC3A2|CD9|DPEP1|UMOD|RDX|TF|PDZK1|ATP1A1|MGAM	3.85E-09	GO.0016324	0.73990271
76	17	GO Function	flavin adenine dinucleotide binding	4.8E-08	ACADS|SDHA|ACAD11|ACAD8|LDHD|PRODH2|ACAD9|CHDH|CYB5R3|MAOB|NQO2|TXNRD2|IVD|ETFDH|ACADVL|ETFA|FMO1	1.07E-09	GO.0050660	0.73187588
1868	103	GO Function	ribonucleotide binding	4.8E-08	RALA|MAPK1|RTCB|MYH9|MTHFD1|PCK2|IDH3G|PNPO|RAB35|ATP6V1B1|TRAP1|TUBA4A|CARS2|KHK|PEBP1|ATP5B|EHD2|SLC27A2|AFG3L2|PFKL|TIMM44|ATP6V1A|ATP6V1B2|CCT5|ACTC1|HSPA9|CKB|CCT2|HSP90B1|TUBA1C|PRKACA|RAB1B|ACSS1|PCK1|RHOT2|TUFM|NDUFV1|HSPA5|EHD3|ACSM2B|RAB11B|HSP90AA1|PKLR|TUBB4B|GNAI1|PFKM|ANXA6|AGK|RAC1|MYH10|LONP1|CYB5R3|OPA1|DARS2|TGM2|IARS2|MYO6|ABCD3|GNAS|ALDH18A1|SCP2|CMPK1|ASS1|RAB14|VARS|PCCA|ACTR2|SUCLA2|HADHA|PFKP|AK3|DNM2|NME2|PC|DAK|AK4|MYH11|ILK|ATP5A1|SEPT7|CDC42|GK|ACSF2|HK1|ACTA2|CKMT2|CKMT1A|FBP1|LARS2|BCS1L|PCCB|GFM1|ACSL1|NDUFA13|ATP1A1|DNM1L|ALDH6A1|ACTG1|ACSM2A|GALK1|TTN|SARS2|HAO2	1.1E-09	GO.0032553	0.73187588
39	13	GO Process	sulfur amino acid metabolic process	4.9E-08	MTHFD1|AHCY|BHMT2|BHMT|MUT|MSRA|AHCYL1|DPEP1|MPST|GGT1|SMS|TST|PCYOX1	1.74E-09	GO.0000096	0.73133637
17	10	GO Process	amino-acid betaine metabolic process	5.2E-08	DMGDH|BBOX1|CPT1A|BHMT|CRAT|SHMT1|CHDH|TMLHE|ALDH9A1|ALDH7A1	1.87E-09	GO.0006577	0.72848326
2180	114	GO Process	cellular localization	6.1E-08	RALA|VCL|MYH9|F9|CTSC|RAB35|SKP1|LTF|EHHADH|CANX|TUBA4A|HSPB1|PMPCB|MTX2|ACTN4|LAMA5|LRPPRC|ERP29|ATP5B|SLC9A3R1|EHD2|NAPA|CD81|CPT1A|SLC27A2|UQCRC2|SOD1|TIMM44|TINAGL1|UCHL1|ATP5O|ATP5J2|SCIN|HSPA9|AMN|HSP90B1|ATP5L|TUBA1C|ATP5H|LETM1|GAA|ATP5I|YWHAG|PRKACA|RAB1B|CRAT|TLN1|CYC1|PIPOX|CALR|RHOT2|HSPA5|DHRS4|EHD3|AP2A2|RAB11B|ACAA1|HSP90AA1|TUBB4B|RPS2|EPB41L3|SAMM50|ANXA2|AGK|RAC1|STOML2|PIGR|ATP5C1|MYH10|OPA1|ATP1B1|S100A10|ATP5F1|AHCYL1|FLNA|MYO6|ABCD3|GNAS|SCP2|PMPCA|YWHAB|RAB14|FLOT1|ACTR2|MPV17|AKR1C3|PITRM1|SLC25A6|AMACR|KRT18|DNM2|CORO1B|JUP|FLOT2|TSPO|ATP5A1|CDC42|RDX|SSB|IDH1|HK1|DDC|TOMM40|SERPINA1|NDUFA13|EPHX2|PHB2|TIMM50|DNM1L|NPC2|RBM8A|PLCG2|AP2B1|HAO2	2.21E-09	GO.0051641	0.72146702

<table>
<tr><th></th><th></th><th></th><th></th><th></th><th></th><th></th><th></th><th></th></tr>
<tr><td>1491</td><td>87</td><td>GO Process</td><td>homeostatic process</td><td>6.2E-08</td><td>DCN|MAPK1|LGALS1|MTHFD1|CTSH|SKP1|LTF|ATP6V1B1|NAPSA|ATP5B|SLC9A3R1|PRDX1|ATP6V0A1|NNT|ADD1|CALB1|PRDX5|RHCG|AFG3L2|SOD1|ATP6V1A|GPD1L|ATP6V0D2|ATP6V0D1|BDH2|HSPA9|PRDX3|CKB|TPP1|HSP90B1|PDIA3|LETM1|GAA|ATP6V0A4|ACO1|PRKACA|ISCU|CYP4A11|AQP1|PCK1|SLC7A8|CALR|RHOT2|FBN1|P4HB|RAB11B|HSP90AA1|PRDX6|PTGES2|PFKM|ANXA6|RAC1|STOML2|PBLD|OPA1|TGM2|FH|ATP1B1|GSTO1|SFXN2|GOT1|GNAS|RBP4|CLIC4|CUBN|TXNDC5|GLRX|MPV17|PRCP|FHL1|UMOD|TSPO|AMPD3|GSTP1|TXNRD2|PDIA6|TF|IMMT|SLC4A4|HK1|GPX1|EPHX2|ACADVL|ATP1A1|NPC2|ACSM2A|PLCG2</td><td>2.24E-09</td><td>GO.0042592</td><td>0.72111249</td></tr>
<tr><td>40</td><td>13</td><td>GO Process</td><td>serine family amino acid metabolic process</td><td>6.2E-08</td><td>MTHFD1|AHCY|AGXT2|HRSP12|AMT|SHMT1|PHGDH|AHCYL1|PSAT1|GLDC|MPST|GGT1|GLYAT</td><td>2.25E-09</td><td>GO.0009069</td><td>0.72111249</td></tr>
<tr><td>484</td><td>42</td><td>GO Process</td><td>organic cyclic compound catabolic process</td><td>6.4E-08</td><td>GSTZ1|AHCY|OGDH|GAPDH|DNPH1|ENO1|GDA|AMBP|PFKL|ALDH1L1|DPYS|HNMT|QDPR|HGD|HPD|FTCD|HPRT1|PRODH2|HINT1|PKLR|RPS2|PFKM|PNP|OGDHL|ALDOB|ALDH4A1|MAOB|DUT|PFKP|CNP|QPRT|AMPD3|FAH|SSB|PNPT1|HK1|GPX1|EPHX2|PAH|ALDH6A1|RBM8A|GALK1</td><td>2.38E-09</td><td>GO.1901361</td><td>0.71911141</td></tr>
<tr><td>995</td><td>66</td><td>GO Process</td><td>ion transmembrane transport</td><td>6.7E-08</td><td>SLC25A11|SLC25A3|ATP6V1B1|PMPCB|ATP5B|LRP2|ATP6V0A1|NNT|VDAC1|CPT1A|SLC27A2|RHCG|AFG3L2|ATP6V1A|ATP6V1B2|SLC25A4|ATP6V0D2|ATP5O|ATP6V0D1|ATP5J2|COX7A1|ATP5L|ATP5H|SLC23A1|LETM1|ATP5I|ATP6V0A4|AQP1|CYC1|COX5A|SLC7A8|SLC5A2|CYB5A|SLC25A15|ANXA6|STOML2|ATP5C1|MPC1|MT-CO1|OPA1|MT-CO2|ATP1B1|ATP5F1|SFXN2|ABCD3|SLC25A5|PMPCA|VDAC2|CLIC4|CLIC1|SLC3A2|SLC25A6|ATP5A1|FKBP1A|TF|PCYOX1|SLC25A12|SLC4A4|COX7C|VDAC3|PHB2|ATP1A1|SLC25A10|COX4I1|PHB|PLCG2</td><td>2.47E-09</td><td>GO.0034220</td><td>0.71771784</td></tr>
<tr><td>1770</td><td>98</td><td>GO Process</td><td>protein-containing complex subunit organization</td><td>6.7E-08</td><td>NDUFB4|DECR1|C1QBP|VTN|CTSC|SKP1|DSTN|VIL1|NDUFA2|NDUFA10|APCS|LAMC1|MRPL15|PSMD11|SLC9A3R1|NAPA|ATP6V0A1|CPT1A|EIF4H|NDUFA9|LUM|PFKL|ECSIT|DPYS|NDUFB9|MRPL49|MRPL17|COL1A2|COL14A1|HPRT1|NDUFB8|CCT2|DCXR|LETM1|MRPL39|MRPL13|ATP6V0A4|PRKACA|RAB1B|ACAD9|GRHPR|TLN1|SHMT1|CALR|NDUFV1|DHRS4|EHD3|P4HB|NDUFA12|UQCR10|MRPL12|HSP90AA1|SAMM50|PFKM|ANXA2|GPX4|ANXA6|RAC1|STOML2|LONP1|MRPL37|MRPL21|OPA1|COA3|TGM2|DPT|S100A10|YWHAB|ALDOB|FLOT1|KCTD12|GPX3|DNM2|JUP|QPRT|MGST1|MYH11|ILK|SEPT7|FKBP1A|ALAD|NDUFS1|CRYZ|PNPT1|COX20|FBP1|BCS1L|SERPINA1|ATPAF2|MRPS22|TIMMDC1|NDUFA13|MRPS30|CRYAB|IST1|DNM1L|TTN|AP2B1</td><td>2.49E-09</td><td>GO.0043933</td><td>0.71745739</td></tr>
<tr><td>1865</td><td>102</td><td>GO Function</td><td>purine nucleotide binding</td><td>8.6E-08</td><td>RALA|MAPK1|RTCB|MYH9|MTHFD1|PCK2|AHCY|IDH3G|PFN1|RAB35|ATP6V1B1|TRAP1|TUBA4A|CARS2|KHK|PEBP1|ATP5B|EHD2|SLC27A2|AFG3L2|PFKL|TIMM44|ATP6V1A|ATP6V1B2|CCT5|ACTC1|HSPA9|CKB|CCT2|HSP90B1|TUBA1C|PRKACA|RAB1B|ACSS1|PCK1|RHOT2|TUFM|HSPA5|EHD3|ACSM2B|RAB11B|HSP90AA1|PKLR|TUBB4B|GNAI1|PFKM|ANXA6|AGK|RAC1|MYH10|LONP1|CYB5R3|OPA1|DARS2|TGM2|IARS2|MYO6|ABCD3|GNAS|ALDH18A1|SCP2|CMPK1|ASS1|RAB14|VARS|PCCA|ACTR2|SUCLA2|HADHA|PFKP|AK3|DNM2|NME2|PC|DAK|AK4|MYH11|ILK|ATP5A1|SEPT7|CDC42|GK|ACSF2|HK1|ACTA2|CKMT2|CKMT1A|FBP1|LARS2|BCS1L|PCCB|GFM1|ACSL1|NDUFA13|ATP1A1|DNM1L|ALDH6A1|ACTG1|ACSM2A|GALK1|TTN|SARS2</td><td>2.05E-09</td><td>GO.0017076</td><td>0.70675262</td></tr>
<tr><td>203</td><td>30</td><td>GO Component</td><td>organelle outer membrane</td><td>8.5E-11</td><td>MTX2|LRPPRC|VDAC1|CPT1A|GBAS|RHOT2|HADHB|CYB5A|SAMM50|AGK|VAT1|CYB5R3|OPA1|MARC2|RTN4IP1|ASS1|VDAC2|CISD1|MAOB|BPHL|CNP|MGST1|TSPO|GK|HK1|TOMM40|ACSL1|VDAC3|PHB2|DNM1L</td><td>6.05E-12</td><td>GO.0031968</td><td>1.00726296</td></tr>
<tr><td>68</td><td>23</td><td>KEGG Pathways</td><td>Glycolysis / Gluconeogenesis</td><td>1.9E-14</td><td>PCK2|GAPDH|ENO1|ALDH2|PFKL|DLAT|ADH5|ADH1B|PDHB|ACSS1|PCK1|PKLR|PFKM|ALDH3A2|ALDH9A1|AKR1A1|ALDOB|ALDH1B1|PDHA1|PFKP|ALDH7A1|HK1|FBP1</td><td>7.23E-16</td><td>hsa00010</td><td>1.37304871</td></tr>
</table>

127	29	GO Component	peroxisome	1.6E-14	CRYM|EHHADH|HRSP12|ACAD11|PRDX5|SLC27A2|SOD1|FABP1|GRHPR|CRAT|PIPOX|DHRS4|IDH2|ACAA1|ALDH3A2|MARC2|ABCD3|SCP2|MPV17|AMACR|MGST1|IDH1|TKT|AOC1|ACSL1|EPHX2|VIM|DNM1L|HAO2	8.45E-16	GO.0005777	1.379588
149	22	GO Process	nucleobase-containing small molecule biosynthetic process	1.1E-07	OGDH|GAPDH|DNPH1|ENO1|GDA|PFKL|HPRT1|HINT1|PKLR|PFKM|PNP|CMPK1|OGDHL|ALDOB|DUT|PFKP|NME2|CNP|AMPD3|HK1|GPX1|GALK1	4.14E-09	GO.0034404	0.6954677
1390	82	GO Process	intracellular transport	1.2E-07	F9|CTSC|RAB35|EHHADH|HSPB1|PMPCB|MTX2|ACTN4|LRPPRC|ERP29|ATP5B|SLC9A3R1|EHD2|NAPA|CPT1A|SLC27A2|UQCRC2|SOD1|TIMM44|TINAGL1|UCHL1|ATP5O|ATP5J2|HSPA9|HSP90B1|ATP5L|TUBA1C|ATP5H|ATP5I|YWHAG|RAB1B|CRAT|CYC1|PIPOX|CALR|RHOT2|DHRS4|EHD3|AP2A2|RAB11B|ACAA1|HSP90AA1|RPS2|SAMM50|AGK|STOML2|ATP5C1|MYH10|OPA1|ATP1B1|ATP5F1|AHCYL1|MYO6|ABCD3|GNAS|SCP2|PMPCA|YWHAB|RAB14|ACTR2|MPV17|AKR1C3|PITRM1|SLC25A6|AMACR|DNM2|TSPO|ATP5A1|CDC42|SSB|IDH1|TOMM40|SERPINA1|NDUFA13|EPHX2|PHB2|TIMM50|DNM1L|NPC2|RBM8A|AP2B1|HAO2	4.36E-09	GO.0046907	0.6935542
181	29	GO Component	mitochondrial outer membrane	3.6E-11	MTX2|VDAC1|CPT1A|GBAS|RHOT2|HADHB|CYB5A|SAMM50|AGK|VAT1|CYB5R3|OPA1|MARC2|RTN4IP1|ASS1|VDAC2|CISD1|MAOB|BPHL|CNP|MGST1|TSPO|GK|HK1|TOMM40|ACSL1|VDAC3|PHB2|DNM1L	2.34E-12	GO.0005741	1.0446117
98	18	GO Process	mitochondrial respiratory chain complex assembly	1.4E-07	NDUFB4|NDUFA2|NDUFA10|NDUFA9|ECSIT|NDUFB9|NDUFB8|ACAD9|NDUFV1|NDUFA12|UQCR10|SAMM50|COA3|NDUFS1|COX20|BCS1L|TIMMDC1|NDUFA13	5.38E-09	GO.0033108	0.68477117
86	17	GO Process	cellular oxidant detoxification	1.4E-07	GSTZ1|PRDX1|NNT|PRDX5|SOD1|FABP1|PRDX3|GSTA1|PRDX6|GPX4|MGST3|GSTO1|GPX3|MGST1|GSTP1|TXNRD2|GPX1	5.46E-09	GO.0098869	0.68416375
186	29	GO Component	ficolin-1-rich granule	6.1E-11	VCL|MAPK1|CTSH|LTA4H|CTSD|LGALS3|PSMD11|COTL1|PSMD3|ATP6V0A1|PFKL|GAA|GLB1|AP2A2|HSP90AA1|CTSB|RAC1|PNP|ACTR2|NME2|PRCP|JUP|MVP|AMPD3|GSTP1|ALAD|IDH1|SERPINA1|MGAM	4.25E-12	GO.0101002	1.02160964
93	28	GO Component	respirasome	1.6E-16	NDUFB4|UQCRC1|PMPCB|NDUFA2|NDUFA10|NNT|SDHA|NDUFA9|UQCRC2|HIGD2A|NDUFB9|COX7A1|NDUFB8|CYCS|UQCRH|CYC1|COX5A|NDUFV1|NDUFA12|UQCR10|MT-CO1|MT-CO2|NDUFS1|HIGD1A|BCS1L|NDUFA13|COX7C|COX4I1	6.28E-18	GO.0070469	1.5790485
8	8	GO Function	enoyl-CoA hydratase activity	1.8E-07	EHHADH|ECI1|HADHB|ECHS1|ECHDC2|AUH|HADHA|ECHDC1	4.54E-09	GO.0004300	0.67375489
70	15	KEGG Pathways	Metabolism of xenobiotics by cytochrome P450	1.9E-07	AKR7A2|GSTM3|ADH5|UGT2B7|ADH1B|UGT2B17|GSTA1|UGT1A9|AKR7A3|MGST3|GSTO1|MGST1|GSTP1|GSTA2|EPHX1	1.69E-08	hsa00980	0.67235382
39	16	KEGG Pathways	Glycine, serine and threonine metabolism	3.3E-11	AGXT2|DMGDH|AMT|BHMT|AOC3|GRHPR|PIPOX|SHMT1|CHDH|PHGDH|SARDH|PSAT1|MAOB|GLDC|GATM|ALDH7A1	1.77E-12	hsa00260	1.04762535
23	10	KEGG Pathways	Histidine metabolism	1.9E-07	ALDH2|HNMT|FTCD|CNDP2|ALDH3A2|ALDH9A1|ALDH1B1|MAOB|ALDH7A1|AOC1	1.76E-08	hsa00340	0.67235382
88	17	GO Process	acyl-CoA metabolic process	1.9E-07	OGDH|PDHX|DLAT|OXSM|PDHB|ACSS1|PIPOX|ACSM2B|DLST|MPC1|SUCLA2|ACOT9|PDHA1|ACSF2|ACSL1|ACSM2A|GLYAT	7.38E-09	GO.0006637	0.67144427
28	11	GO Process	cellular modified amino acid catabolic process	2E-07	AHCY|GOT2|DMGDH|ALDH1L1|BHMT|HOGA1|SARDH|ALDH4A1|GGT5|GGT1|PCYOX1	7.84E-09	GO.0042219	0.6692504
9	8	GO Process	glyoxylate metabolic process	2.2E-07	AGXT2|GOT2|PRODH2|GRHPR|IDH2|HOGA1|ALDH4A1|IDH1	8.36E-09	GO.0046487	0.66655462
21	10	GO Process	glutathione derivative biosynthetic process	2.3E-07	GSTZ1|GSTM3|GSTA1|MGST3|GSTO1|AKR1A1|ESD|MGST1|GSTP1|GSTA2	8.88E-09	GO.1901687	0.66420652

21	10	GO Process	neurotransmitter catabolic process	2.3E-07	DMGDH|AMT|BHMT|HNMT|CHDH|SARDH|MAOB|GLDC|ABAT|ALDH7A1	8.88E-09	GO.0042135	0.66420652
1853	100	GO Function	purine ribonucleotide binding	2.3E-07	RALA|MAPK1|RTCB|MYH9|MTHFD1|PCK2|IDH3G|RAB35|ATP6V1B1|TRAP1|TUBA4A|CARS2|KHK|PEBP1|ATP5B|EHD2|SLC27A2|AFG3L2|PFKL|TIMM44|ATP6V1A|ATP6V1B2|CCT5|ACTC1|HSPA9|CKB|CCT2|HSP90B1|TUBA1C|PRKACA|RAB1B|ACSS1|PCK1|RHOT2|TUFM|HSPA5|EHD3|ACSM2B|RAB11B|HSP90AA1|PKLR|TUBB4B|GNAI1|PFKM|ANXA6|AGK|RAC1|MYH10|LONP1|CYB5R3|OPA1|DARS2|TGM2|IARS2|MYO6|ABCD3|GNAS|ALDH18A1|SCP2|CMPK1|ASS1|RAB14|VARS|PCCA|ACTR2|SUCLA2|HADHA|PFKP|AK3|DNM2|NME2|PC|DAK|AK4|MYH11|ILK|ATP5A1|SEPT7|CDC42|GK|ACSF2|HK1|ACTA2|CKMT2|CKMT1A|FBP1|LARS2|BCS1L|PCCB|GFM1|ACSL1|NDUFA13|ATP1A1|DNM1L|ALDH6A1|ACTG1|ACSM2A|GALK1|TTN|SARS2	5.92E-09	GO.0032555	0.6636388
56	14	GO Process	nucleoside diphosphate phosphorylation	2.4E-07	OGDH|GAPDH|ENO1|PFKL|PKLR|PFKM|CMPK1|OGDHL|ALDOB|PFKP|NME2|AK4|HK1|GALK1	9.46E-09	GO.0006165	0.66216021
806	56	GO Process	cellular homeostasis	2.5E-07	MAPK1|LGALS1|SKP1|LTF|ATP5B|SLC9A3R1|PRDX1|ATP6V0A1|NNT|ADD1|CALB1|PRDX5|RHCG|AFG3L2|SOD1|ATP6V1A|ATP6V0D2|ATP6V0D1|PRDX3|CKB|HSP90B1|PDIA3|LETM1|GAA|ATP6V0A4|ACO1|PRKACA|ISCU|AQP1|CALR|RHOT2|P4HB|RAB11B|PRDX6|PTGES2|PFKM|ANXA6|STOML2|OPA1|TGM2|ATP1B1|GSTO1|GOT1|CLIC4|TXNDC5|GLRX|TXNRD2|PDIA6|TF|IMMT|SLC4A4|HK1|GPX1|EPHX2|ATP1A1|PLCG2	9.9E-09	GO.0019725	0.66038007
2815	135	GO Process	response to organic substance	2.6E-07	RALA|DCN|OXCT1|UQCRC1|MAPK1|LGALS1|CTSH|CTSC|GAPDH|RAB35|SKP1|ATP6V1B1|GOT2|CANX|VIL1|HSPB1|LAMA5|HRSP12|MRPL15|GGH|ADAM10|KHK|CD81|ATP6V0A1|ADD1|F13A1|CALB1|CPT1A|LUM|SORD|PFKL|SOD1|ATP6V1A|ATP6V1B2|ERLIN2|QDPR|ATP6V0D2|ACTC1|ATP6V0D1|CAPN2|ADH5|HSPA9|COL1A2|PRDX3|HPRT1|TPP1|HSP90B1|GPD1|YWHAG|GLB1|ATP6V0A4|PRKACA|AQP1|TLN1|CYC1|SHMT1|PCK1|CALR|TUFM|BCAT2|HSPA5|FBN1|BGN|P4HB|RAB11B|HSP90AA1|PKLR|GNAI1|CTSB|ANXA2|MSN|COL4A2|LONP1|MPC1|OPA1|MGST3|GSTO1|AHCYL1|ABCD3|GOT1|GNAS|RBP4|SLC25A5|ASS1|RAB14|ASPN|COL4A1|FLOT1|SLC3A2|ACTR2|MAOB|ESD|POSTN|AKR1C3|HADHA|PFKP|GLDC|CD9|FECH|GPX3|KRT18|DNM2|CNP|CORO1B|JUP|MGST1|TSPO|ABAT|ILK|GSTP1|CDC42|RDX|IFI30|PDIA6|ALAD|ARPC1B|IDH1|PNPT1|DDC|AOC1|MME|GSTA2|ACSL1|NDUFA13|CRYAB|ACADVL|PHB2|ATP1A1|VIM|DNM1L|ACTG1|PHB|EPHX1|FMO1|PLCG2	1.03E-08	GO.0010033	0.65900669
453	39	GO Process	aromatic compound catabolic process	2.7E-07	GSTZ1|AHCY|OGDH|GAPDH|DNPH1|ENO1|GDA|AMBP|PFKL|ALDH1L1|DPYS|HNMT|QDPR|HGD|HPD|FTCD|HPRT1|HINT1|PKLR|RPS2|PFKM|PNP|OGDHL|ALDOB|MAOB|DUT|PFKP|CNP|AMPD3|FAH|SSB|PNPT1|HK1|GPX1|EPHX2|PAH|RBM8A|GALK1|EPHX1	1.1E-08	GO.0019439	0.65622494
55	14	GO Function	hydro-lyase activity	2.9E-07	ACO2|EHHADH|ENO1|CA4|ECI1|ACO1|HADHB|FH|ECHS1|ECHDC2|AUH|HADHA|ALAD|ECHDC1	7.77E-09	GO.0016836	0.65316527
68	15	GO Process	nicotinamide nucleotide biosynthetic process	3E-07	OGDH|GAPDH|ENO1|PFKL|IDH2|PKLR|PFKM|PNP|OGDHL|ALDOB|PFKP|QPRT|NAPRT|HK1|GALK1	1.21E-08	GO.0019359	0.65243288

2040	106	GO Process	organic substance transport	3.2E-07	MYH9|SLC25A11|RAB35|EHHADH|ATP6V1B1|GOT2|CANX|VIL1|HSPB1|PMPCB|MTX2|ACTN4|LRPPRC|ERP29|SLC9A3R1|NAPA|LRP2|ATP6V0A1|CPT1A|SLC27A2|RHCG|UQCRC2|TIMM44|ATP6V1A|ATP6V1B2|SLC25A4|ATP6V0D2|ATP6V0D1|FABP1|HSPA9|AMN|HSP90B1|CA4|SLC23A1|YWHAG|ATP6V0A4|RAB1B|AQP1|DAB2|CRAT|PIPOX|SLC7A8|CALR|DHRS4|EHD3|AP2A2|SLC5A2|SERPINA5|RAB11B|ACAA1|HSP90AA1|PLS1|RPS2|SLC25A15|SAMM50|AGK|GM2A|MPC1|PNP|ATP1B1|AHCYL1|MYO6|ABCD3|GNAS|RBP4|SCP2|SLC25A5|PMPCA|YWHAB|RAB14|FLOT1|CUBN|SLC3A2|APOO|MPV17|AKR1C3|PITRM1|SLC25A6|AMACR|KRT18|DNM2|MVP|TSPO|TST|TF|SSB|SLC25A12|IDH1|SLC4A4|PDZK1|SELENBP1|PNPT1|DDC|TOMM40|ACSL1|NDUFA13|VDAC3|EPHX2|IST1|PHB2|TIMM50|SLC25A10|NPC2|RBM8A|AP2B1|HAO2	1.29E-08	GO.0071702	0.64975729
58	14	GO Process	protein targeting to mitochondrion	3.4E-07	PMPCB|MTX2|UQCRC2|TIMM44|HSP90AA1|SAMM50|AGK|PMPCA|PITRM1|SLC25A6|TSPO|TOMM40|NDUFA13|TIMM50	1.39E-08	GO.0006626	0.64698003
69	15	GO Process	primary alcohol metabolic process	3.5E-07	AKR7A2|TTR|ALDH2|ADH5|ALDH1A1|GPD1|ADH1B|GPD2|ACSS1|DHRS4|ALDH3A2|RBP4|ALDH1B1|AKR1C3|RDH13	1.43E-08	GO.0034308	0.64596705
69	15	GO Process	glutamine family amino acid metabolic process	3.5E-07	GOT2|DDAH1|FTCD|GLYATL1|PRODH2|ASL|PHGDH|GOT1|ALDH18A1|ASS1|ALDH4A1|AGMAT|NIT2|GGT1|FAH	1.43E-08	GO.0009064	0.64596705
81	16	GO Process	water-soluble vitamin metabolic process	3.7E-07	MTHFD1|PNPO|ALDH1L1|MUT|AMN|SLC23A1|SHMT1|CYB5A|CYB5R3|GSTO1|AKR1A1|PCCA|PSAT1|CUBN|PC|PCCB	1.55E-08	GO.0006767	0.64294571
15	9	GO Function	aldehyde dehydrogenase (NAD) activity	4E-07	ALDH2|ALDH1L1|ADH5|ALDH1A1|ALDH3A2|ALDH9A1|ALDH4A1|ALDH1B1|ALDH7A1	1.09E-08	GO.0004029	0.63990271
70	15	GO Process	establishment of protein localization to mitochondrion	4E-07	PMPCB|MTX2|UQCRC2|TIMM44|HSP90AA1|SAMM50|AGK|PMPCA|PITRM1|SLC25A6|TSPO|HK1|TOMM40|NDUFA13|TIMM50	1.69E-08	GO.0072655	0.63936186
79	16	GO Function	antioxidant activity	4.1E-07	GSTZ1|PRDX1|PRDX5|SOD1|FABP1|PRDX3|GSTA1|PRDX6|GPX4|MGST3|GSTO1|GPX3|MGST1|GSTP1|TXNRD2|GPX1	1.14E-08	GO.0016209	0.6391474
49	13	GO Process	hexose catabolic process	4.2E-07	GAPDH|ENO1|KHK|PFKL|ALDH1A1|GLB1|PKLR|PFKM|ALDOB|PFKP|DAK|HK1|GALK1	1.75E-08	GO.0019320	0.63798639
76	15	KEGG Pathways	Drug metabolism - other enzymes	4.5E-07	GSTM3|DPYS|HPRT1|UGT2B7|CES2|UGT2B17|GSTA1|UGT1A9|MGST3|GSTO1|DUT|NME2|MGST1|GSTP1|GSTA2	4.41E-08	hsa00983	0.635164
59	14	KEGG Pathways	Lysine degradation	1.9E-07	OGDH|EHHADH|ALDH2|BBOX1|PIPOX|TMLHE|DLST|ALDH3A2|ALDH9A1|ECHS1|OGDHL|ALDH1B1|HADHA|ALDH7A1	1.67E-08	hsa00310	0.67235382
58	14	GO Function	oxidoreductase activity, acting on NAD(P)H, quinone or similar compound as acceptor	4.8E-07	NDUFB4|NDUFA2|NDUFA10|NDUFA9|NDUFB9|NDUFB8|DCXR|NDUFV1|DHRS4|NDUFA12|AKR1C3|NDUFS1|CRYZ|NDUFA13	1.39E-08	GO.0016655	0.63178549
66	14	KEGG Pathways	Drug metabolism - cytochrome P450	5.4E-07	GSTM3|ADH5|UGT2B7|ADH1B|UGT2B17|GSTA1|UGT1A9|MGST3|GSTO1|MAOB|MGST1|GSTP1|GSTA2|FMO1	5.67E-08	hsa00982	0.62716462
105	28	GO Component	melanosome	1.9E-15	AHCY|RAB35|CTSD|CANX|GGH|ERP29|PRDX1|ATP6V0A1|ATP6V1B2|TPP1|HSP90B1|PPIB|PDIA3|HSPA5|P4HB|HSP90AA1|CTSB|ANXA2|ANXA6|RAC1|YWHAB|FLOT1|SLC3A2|CNP|MYH11|PDIA6|ATP1A1|ITGB3	8.95E-17	GO.0042470	1.47304871
371	27	GO Component	cell leading edge	0.00022	MYH9|VIL1|SLC9A3R1|IQGAP2|ATP6V1B2|ACTC1|TLN1|HSP90AA1|EPB41L3|ANXA2|RAC1|MYH10|DPP4|MYO6|GNAS|FLOT1|ACTR2|DNM2|NME2|CORO1B|ACTN1|FLOT2|ILK|RDX|ACTA2|VIM|ITGB3	3.47E-05	GO.0031252	0.36575773

41	12	GO Process	cellular modified amino acid biosynthetic process	5.9E-07	MTHFD1|BBOX1|SHMT1|CHDH|CNDP2|TMLHE|ALDH9A1|GATM|HAGH|GGT5|GGT1|ALDH7A1	2.49E-08	GO.0042398	0.62298847
17	8	KEGG Pathways	Phenylalanine metabolism	2.6E-06	GOT2|HPD|AOC3|GOT1|MAOB|DDC|PAH|GLYAT	3.04E-07	hsa00360	0.55850267
5126	212	GO Process	cellular nitrogen compound metabolic process	6.6E-07	NDUFB4|UQCRC1|NANS|MAPK1|RTCB|GSTZ1|MTHFD1|AHCY|CTSH|OGDH|PNPO|C1QBP|PDHX|LTA4H|GAPDH|DNPH1|LTF|ATP6V1B1|SPR|ENO1|GDA|PMPCB|NDUFA2|NDUFA10|LGALS3|GRSF1|HRSP12|DMGDH|GSTM3|CARS2|MRPL15|GGH|LRPPRC|ATP5B|CHCHD3|BBOX1|ATP6V0A1|NNT|SDHA|AMBP|ENPEP|PRDX5|CPT1A|EIF4H|REXO2|NDUFA9|UQCRC2|PFKL|SOD1|NAT8|ATP6V1A|ALDH1L1|BHMT|ATP6V1B2|LACTB2|DPYS|NDUFB9|MRPL49|HNMT|DLAT|OXSM|ACAD8|QDPR|GPD1L|MRPL17|ATP5O|FTCD|ATP5J2|RNPEP|BDH2|HPRT1|NDUFB8|TPP1|ANPEP|ATP5L|GPD1|ATP5H|SLC23A1|DCXR|HINT1|MRPL39|ATP5I|MRPL13|ASL|PDHB|CYCS|ATP6V0A4|GPD2|DPYSL2|UQCRH|PRKACA|AQP1|GBAS|GRHPR|TSFM|CRAT|ACSS1|CYC1|PIPOX|COX5A|SHMT1|UGDH|CHDH|TUFM|NDUFV1|CNDP2|MDH2|ACSM2B|NDUFA12|IDH2|UQCR10|MRPL12|HSP90AA1|TMLHE|DLST|GSTA1|PKLR|RPS2|SLC25A15|PPA2|PFKM|ALDH9A1|AGK|STOML2|ATP5C1|GM2A|COL4A2|LONP1|MRPL37|MPC1|MT-CO1|PNP|MRPL21|OPA1|MT-CO2|DARS2|IBA57|IARS2|ATP1B1|LHPP|GSTO1|ATP5F1|AHCYL1|FLNA|GNAS|SARDH|CMPK1|ASS1|OGDHL|ALDOB|VARS|AGMAT|PCCA|PSAT1|SUCLA2|ACOT9|PDHA1|GLRX|DUT|PFKP|AK3|FECH|DPEP1|NME2|CNP|PC|NIT2|AK4|QPRT|TSPO|AMPD3|MPST|HAGH|GGT5|GSTP1|ATP5A1|GGT1|SMS|TST|ALAD|SSB|ALDH7A1|IDH1|TKT|NDUFS1|PNPT1|NAPRT|ACSF2|HK1|DDC|GPX1|LARS2|MRPS22|MME|PCCB|GFM1|SSBP1|ABHD14B|GSTA2|ACSL1|MRPS30|COX7C|PHB2|ALDH6A1|COX4I1|ACSM2A|RBM8A|GALK1|SARS2|PHB|FMO1|GLYAT	2.8E-08	GO.0034641	0.61811146
40	12	GO Function	peroxidase activity	6.7E-07	GSTZ1|PRDX1|PRDX5|PRDX3|GSTA1|PRDX6|GPX4|MGST3|GPX3|MGST1|GSTP1|GPX1	1.98E-08	GO.0004601	0.61771784
42	12	GO Process	pyruvate biosynthetic process	7.3E-07	OGDH|GAPDH|ENO1|PFKL|PKLR|PFKM|HOGA1|OGDHL|ALDOB|PFKP|HK1|GALK1	3.12E-08	GO.0042866	0.61360826
61	14	GO Function	oxidoreductase activity, acting on a sulfur group of donors	7.9E-07	SQRDL|PRDX3|PDIA3|MSRA|P4HB|PTGES2|GSTO1|GLRX|SUOX|TXNRD2|IFI30|PDIA6|PCYOX1|SELENBP1	2.41E-08	GO.0016667	0.61034738
1616	88	GO Process	establishment of localization in cell	9.1E-07	MYH9|F9|CTSC|RAB35|LTF|EHHADH|CANX|HSPB1|PMPCB|MTX2|ACTN4|LRPPRC|ERP29|ATP5B|SLC9A3R1|EHD2|NAPA|CPT1A|SLC27A2|UQCRC2|SOD1|TIMM44|TINAGL1|UCHL1|ATP5O|ATP5J2|HSPA9|HSP90B1|ATP5L|TUBA1C|ATP5H|LETM1|ATP5I|YWHAG|RAB1B|CRAT|CYC1|PIPOX|CALR|RHOT2|DHRS4|EHD3|AP2A2|RAB11B|ACAA1|HSP90AA1|RPS2|SAMM50|AGK|STOML2|ATP5C1|MYH10|OPA1|ATP1B1|ATP5F1|AHCYL1|MYO6|ABCD3|GNAS|SCP2|PMPCA|YWHAB|RAB14|ACTR2|MPV17|AKR1C3|PITRM1|SLC25A6|AMACR|DNM2|TSPO|ATP5A1|CDC42|SSB|IDH1|DDC|TOMM40|SERPINA1|NDUFA13|EPHX2|PHB2|TIMM50|DNM1L|NPC2|RBM8A|PLCG2|AP2B1|HAO2	3.91E-08	GO.0051649	0.60419142
437	37	GO Process	monovalent inorganic cation transport	9.1E-07	SLC25A3|ATP6V1B1|ATP5B|SLC9A3R1|ATP6V0A1|NNT|NDUFA9|ATP6V1A|ATP6V1B2|ATP6V0D2|ATP5O|ATP6V0D1|ATP5J2|COX7A1|ATP5L|ATP5H|SLC23A1|LETM1|ATP5I|ATP6V0A4|AQP1|CYC1|COX5A|SLC5A2|CYB5A|STOML2|ATP5C1|MT-CO1|MT-CO2|ATP1B1|ATP5F1|SLC3A2|ATP5A1|SLC4A4|COX7C|ATP1A1|COX4I1	3.93E-08	GO.0015672	0.60409586
44	12	GO Process	ADP metabolic process	1.1E-06	OGDH|GAPDH|ENO1|PFKL|PKLR|PFKM|OGDHL|ALDOB|PFKP|AMPD3|HK1|GALK1	4.82E-08	GO.0046031	0.5954677
441	37	GO Process	cellular nitrogen compound catabolic process	1.1E-06	AHCY|OGDH|GAPDH|DNPH1|ENO1|GDA|DMGDH|AMBP|PFKL|ALDH1L1|BHMT|DPYS|HNMT|FTCD|HPRT1|HINT1|PKLR|RPS2|PFKM|PNP|OGDHL|ALDOB|DUT|PFKP|DPEP1|CNP|QPRT|AMPD3|MPST|TST|SSB|PNPT1|HK1|GPX1|ALDH6A1|RBM8A|GALK1	4.88E-08	GO.0044270	0.5954677

65	14	GO Process	mitochondrial respiratory chain complex I assembly	1.1E-06	NDUFB4|NDUFA2|NDUFA10|NDUFA9|ECSIT|NDUFB9|NDUFB8|ACAD9|NDUFV1|NDUFA12|NDUFS1|BCS1L|TIMMDC1|NDUFA13	4.81E-08	GO.0032981	0.5954677
1794	95	GO Function	purine ribonucleoside triphosphate binding	1.1E-06	RALA|MAPK1|RTCB|MYH9|MTHFD1|PCK2|IDH3G|RAB35|ATP6V1B1|TRAP1|TUBA4A|CARS2|KHK|PEBP1|ATP5B|EHD2|SLC27A2|AFG3L2|PFKL|TIMM44|ATP6V1A|ATP6V1B2|CCT5|ACTC1|HSPA9|CKB|CCT2|HSP90B1|TUBA1C|PRKACA|RAB1B|ACSS1|PCK1|RHOT2|TUFM|HSPA5|EHD3|ACSM2B|RAB11B|HSP90AA1|PKLR|TUBB4B|GNAI1|PFKM|ANXA6|AGK|RAC1|MYH10|LONP1|OPA1|DARS2|TGM2|IARS2|MYO6|ABCD3|GNAS|ALDH18A1|CMPK1|ASS1|RAB14|VARS|PCCA|ACTR2|SUCLA2|PFKP|AK3|DNM2|NME2|PC|DAK|AK4|MYH11|ILK|ATP5A1|SEPT7|CDC42|GK|ACSF2|HK1|ACTA2|CKMT2|CKMT1A|LARS2|BCS1L|PCCB|GFM1|ACSL1|NDUFA13|ATP1A1|DNM1L|ACTG1|ACSM2A|GALK1|TTN|SARS2	3.68E-08	GO.0035639	0.59430951
155	21	GO Function	ligase activity	1.1E-06	RTCB|MTHFD1|CARS2|SLC27A2|UCHL1|ACSS1|ACSM2B|DARS2|IARS2|ASS1|VARS|PCCA|SUCLA2|PC|NAPRT|ACSF2|LARS2|PCCB|ACSL1|ACSM2A|SARS2	3.58E-08	GO.0016874	0.59430951
144	20	GO Process	cellular carbohydrate metabolic process	1.2E-06	KHK|SORD|PFKL|ABHD10|GAA|GLB1|GPD2|PCK1|IDH2|PFKM|GOT1|PFKP|DAK|IDH1|GK|HK1|FBP1|GBE1|MGAM|GALK1	5.23E-08	GO.0044262	0.5928118
19	9	GO Function	glutathione peroxidase activity	1.5E-06	GSTZ1|GSTA1|PRDX6|GPX4|MGST3|GPX3|MGST1|GSTP1|GPX1	5.18E-08	GO.0004602	0.58153086
275	26	GO Component	endocytic vesicle	5.1E-06	RAB35|LTF|LRP2|ATP6V0A1|ATP6V0D2|ATP6V0D1|AMN|HSP90B1|PDIA3|ATP6V0A4|CALR|EHD3|AP2A2|RAB11B|HSP90AA1|DPP4|MYO6|RAB14|CUBN|CD9|DNM2|FLOT2|TF|VIM|ACTG1|AP2B1	6.35E-07	GO.0030139	0.52915791
27	10	GO Function	oxidoreductase activity, acting on the CH-NH group of donors	1.7E-06	MTHFD1|CRYM|DMGDH|ALDH1L1|QDPR|PRODH2|PIPOX|SARDH|ALDH4A1|ETFDH	5.97E-08	GO.0016645	0.57644716
1514	83	GO Process	protein-containing complex assembly	1.7E-06	NDUFB4|DECR1|C1QBP|VTN|CTSC|SKP1|VIL1|NDUFA2|NDUFA10|APCS|LAMC1|PSMD11|SLC9A3R1|NAPA|ATP6V0A1|CPT1A|EIF4H|NDUFA9|PFKL|ECSIT|DPYS|NDUFB9|COL1A2|HPRT1|NDUFB8|CCT2|DCXR|LETM1|ATP6V0A4|PRKACA|RAB1B|ACAD9|GRHPR|TLN1|SHMT1|CALR|NDUFV1|DHRS4|EHD3|P4HB|NDUFA12|UQCR10|HSP90AA1|SAMM50|PFKM|ANXA2|GPX4|ANXA6|RAC1|STOML2|LONP1|OPA1|COA3|TGM2|S100A10|YWHAB|ALDOB|FLOT1|KCTD12|GPX3|DNM2|JUP|QPRT|MGST1|MYH11|ILK|SEPT7|FKBP1A|ALAD|NDUFS1|CRYZ|PNPT1|COX20|FBP1|BCS1L|SERPINA1|ATPAF2|TIMMDC1|NDUFA13|CRYAB|DNM1L|TTN|AP2B1	7.71E-08	GO.0065003	0.57594508
80	15	GO Process	nucleoside diphosphate metabolic process	1.8E-06	OGDH|GAPDH|ENO1|PFKL|PKLR|PFKM|CMPK1|OGDHL|ALDOB|PFKP|NME2|AK4|AMPD3|HK1|GALK1	7.98E-08	GO.0009132	0.5747147
195	23	GO Process	cellular response to toxic substance	1.8E-06	GSTZ1|PPIF|GSTM3|PRDX1|NNT|PRDX5|SOD1|FABP1|ADH5|PRDX3|AQP1|GSTA1|PRDX6|GPX4|MGST3|GSTO1|ESD|GPX3|DNM2|MGST1|GSTP1|TXNRD2|GPX1	8.15E-08	GO.0097237	0.57399286
495	39	GO Process	amide biosynthetic process	2.2E-06	MTHFD1|PDHX|CARS2|MRPL15|EIF4H|MRPL49|DLAT|MRPL17|BDH2|MRPL39|MRPL13|ASL|PDHB|TSFM|ACSS1|TUFM|CNDP2|MRPL12|RPS2|SLC25A15|PPA2|AGK|MRPL37|MPC1|MRPL21|DARS2|IARS2|ASS1|VARS|PDHA1|HAGH|GGT5|GGT1|LARS2|MRPS22|GFM1|ACSL1|MRPS30|SARS2	9.89E-08	GO.0043604	0.56575773
76	14	KEGG Pathways	Cardiac muscle contraction	2.3E-06	UQCRC1|UQCRC2|ACTC1|COX7A1|UQCRH|CYC1|COX5A|UQCR10|MT-CO1|MT-CO2|ATP1B1|COX7C|ATP1A1|COX4I1	2.61E-07	hsa04260	0.5636388
38	11	GO Process	glycolytic process	2.3E-06	OGDH|GAPDH|ENO1|PFKL|PKLR|PFKM|OGDHL|ALDOB|PFKP|HK1|GALK1	1.05E-07	GO.0006096	0.56326441

85	25	GO Component	mitochondrial respirasome	1.2E-14	NDUFB4|UQCRC1|PMPCB|NDUFA2|NDUFA10|NNT|SDHA|NDUFA9|UQCRC2|NDUFB9|COX7A1|NDUFB8|UQCRH|CYC1|COX5A|NDUFV1|NDUFA12|UQCR10|MT-CO1|MT-CO2|NDUFS1|BCS1L|NDUFA13|COX7C|COX4I1	5.98E-16	GO.0005746	1.39318141
3131	142	GO Process	organelle organization	2.4E-06	RALA|NDUFB4|MYH9|PPIF|PFN1|C1QBP|NAGLU|CTSC|GAPDH|SKP1|EHHADH|DSTN|TUBA4A|VIL1|PMPCB|MTX2|NDUFA2|ACTN4|NDUFA10|LAMA5|ATP5B|CHCHD3|SLC9A3R1|EHD2|NAPA|ADD1|MTFP1|NDUFA9|SLC27A2|UQCRC2|AFG3L2|SOD1|ECSIT|TIMM44|IQGAP2|NDUFB9|SLC25A4|MRPL17|ATP5O|ACTC1|ATP6V0D1|ATP5J2|SCIN|PRDX3|NDUFB8|TPP1|HSP90B1|ATP5L|TUBA1C|ATP5H|LETM1|GAA|ATP5I|YWHAG|CYCS|DPYSL2|C19orf70|PRKACA|RAB1B|AQP1|ACAD9|CRAT|TLN1|PIPOX|TMEM11|CALR|RHOT2|NDUFV1|DHRS4|EHD3|NDUFA12|UQCR10|ACAA1|PARVA|HSP90AA1|PLS1|TUBB4B|EPB41L3|TPM4|SAMM50|ANXA2|AGK|RAC1|STOML2|ATP5C1|MSN|MYH10|LONP1|OPA1|COA3|KRT19|S100A10|ATP5F1|FLNA|ABCD3|SCP2|SLC25A5|PMPCA|YWHAB|APOOL|RAB14|ACTR2|APOO|MPV17|PITRM1|SLC25A6|AMACR|KRT18|DNM2|CNP|CORO1B|JUP|ACTN1|TSPO|MYH11|ATP5A1|SEPT7|CDC42|IMMT|ARPC1B|IDH1|NDUFS1|PNPT1|COX20|TOMM40|BCS1L|SERPINA1|TIMMDC1|SSBP1|NDUFA13|EPHX2|CRYAB|IST1|PHB2|TIMM50|VIM|DNM1L|ACTG1|TTN|SIRT5|PHB|HAO2	1.1E-07	GO.0006996	0.56143937
5	3	KEGG Pathways	Phenylalanine, tyrosine and tryptophan biosynthesis	0.0049	GOT2|GOT1|PAH	0.0012	hsa00400	0.23098039
135	19	GO Function	vitamin binding	2.6E-06	OGDH|PNPO|AGXT2|GOT2|CALB1|RBP5|MUT|FTCD|SHMT1|MARC2|GOT1|RBP4|OGDHL|PCCA|CUBN|GLDC|PC|ABAT|DDC	9.34E-08	GO.0019842	0.55800443
123	18	GO Process	purine nucleoside bisphosphate metabolic process	2.6E-06	OGDH|PDHX|DLAT|OXSM|PDHB|ACSS1|PIPOX|ACSM2B|DLST|MPC1|SUCLA2|ACOT9|PDHA1|ACSF2|ABHD14B|ACSL1|ACSM2A|GLYAT	1.2E-07	GO.0034032	0.55783961
123	18	GO Process	ribonucleoside bisphosphate metabolic process	2.6E-06	OGDH|PDHX|DLAT|OXSM|PDHB|ACSS1|PIPOX|ACSM2B|DLST|MPC1|SUCLA2|ACOT9|PDHA1|ACSF2|ABHD14B|ACSL1|ACSM2A|GLYAT	1.2E-07	GO.0033875	0.55783961
58	13	GO Function	oxidoreductase activity, acting on the CH-CH group of donors	2.7E-06	DECR1|ACADS|MECR|SDHA|ACAD11|ACAD8|BDH2|ACAD9|ACAA1|AKR1C3|PTGR1|IVD|ACADVL	9.69E-08	GO.0016627	0.55734887
995	61	GO Process	chemical homeostasis	3.2E-06	DCN|MAPK1|LGALS1|CTSH|SKP1|LTF|ATP6V1B1|NAPSA|ATP5B|SLC9A3R1|ATP6V0A1|NNT|CALB1|RHCG|AFG3L2|SOD1|ATP6V1A|GPD1L|ATP6V0D2|ATP6V0D1|BDH2|CKB|HSP90B1|LETM1|ATP6V0A4|ACO1|PRKACA|ISCU|CYP4A11|AQP1|PCK1|SLC7A8|CALR|FBN1|RAB11B|PFKM|ANXA6|STOML2|OPA1|TGM2|ATP1B1|GSTO1|SFXN2|GOT1|GNAS|RBP4|CLIC4|PRCP|FHL1|UMOD|TSPO|GSTP1|TF|IMMT|SLC4A4|HK1|EPHX2|ATP1A1|NPC2|ACSM2A|PLCG2	1.49E-07	GO.0048878	0.54921441
30	10	GO Function	ATPase activity, coupled to transmembrane movement of ions, rotational mechanism	3.6E-06	ATP5B|ATP6V0A1|ATP6V1A|ATP6V1B2|ATP6V0D2|ATP5O|ATP6V0D1|ATP6V0A4|ATP5C1|ATP5A1	1.34E-07	GO.0044769	0.54412914

679	47	GO Function	structural molecule activity	4.6E-06	VCL|TUBA4A|LAMC1|MRPL15|PSMD11|CHCHD3|SLC9A3R1|ADD1|VCAN|LUM|TINAGL1|MRPL49|MRPL17|COL1A2|COL14A1|TUBA1C|MRPL13|ISCU|TLN1|PLEC|FBN1|BGN|MRPL12|PLS1|TUBB4B|EPB41L3|TPM4|MSN|COL4A2|MRPL37|MRPL21|KRT19|COL4A1|ACTR2|KRT18|JUP|SNTB1|MYH11|SEPT7|ARPC1B|MRPS22|MRPS30|CRYAB|VIM|MYL6|ACTG1|TTN	1.8E-07	GO.0005198	0.5333482
618	44	GO Process	inorganic cation transmembrane transport	4.7E-06	SLC25A3|ATP6V1B1|PMPCB|ATP5B|ATP6V0A1|NNT|AFG3L2|ATP6V1A|ATP6V1B2|ATP6V0D2|ATP5O|ATP6V0D1|ATP5J2|COX7A1|ATP5L|ATP5H|SLC23A1|LETM1|ATP5I|ATP6V0A4|AQP1|CYC1|COX5A|SLC5A2|CYB5A|STOML2|ATP5C1|MT-CO1|OPA1|MT-CO2|ATP1B1|ATP5F1|PMPCA|SLC3A2|ATP5A1|FKBP1A|TF|SLC4A4|COX7C|PHB2|ATP1A1|COX4I1|PHB|PLCG2	2.18E-07	GO.0098662	0.53288272
866	55	GO Process	cation transport	4.8E-06	SLC25A3|LTF|ATP6V1B1|PMPCB|ATP5B|SLC9A3R1|LRP2|ATP6V0A1|NNT|NDUFA9|RHCG|AFG3L2|ATP6V1A|ATP6V1B2|ATP6V0D2|ATP5O|ATP6V0D1|ATP5J2|COX7A1|ATP5L|ATP5H|SLC23A1|LETM1|ATP5I|ATP6V0A4|AQP1|CYC1|COX5A|SLC7A8|SLC5A2|RAB11B|CYB5A|SLC25A15|ANXA6|STOML2|ATP5C1|MT-CO1|OPA1|MT-CO2|ATP1B1|ATP5F1|PMPCA|SLC3A2|DNM2|ATP5A1|FKBP1A|TF|SLC4A4|PDZK1|COX7C|PHB2|ATP1A1|COX4I1|PHB|PLCG2	2.22E-07	GO.0006812	0.5322393
28	13	KEGG Pathways	Glyoxylate and dicarboxylate metabolism	9.1E-10	ACO2|AMT|MUT|ACO1|GRHPR|SHMT1|MDH2|CS|HOGA1|PCCA|GLDC|PCCB|HAO2	6.46E-11	hsa00630	0.90390538
23	9	GO Function	transferase activity, transferring nitrogenous groups	4.8E-06	GAPDH|AGXT2|GOT2|AMT|BCAT2|GOT1|PSAT1|ABAT|GATM	1.9E-07	GO.0016769	0.53187588
707	48	GO Process	inorganic ion transmembrane transport	4.8E-06	SLC25A3|ATP6V1B1|PMPCB|ATP5B|ATP6V0A1|NNT|AFG3L2|ATP6V1A|ATP6V1B2|ATP6V0D2|ATP5O|ATP6V0D1|ATP5J2|COX7A1|ATP5L|ATP5H|SLC23A1|LETM1|ATP5I|ATP6V0A4|AQP1|CYC1|COX5A|SLC5A2|CYB5A|STOML2|ATP5C1|MT-CO1|OPA1|MT-CO2|ATP1B1|ATP5F1|PMPCA|CLIC4|CLIC1|SLC3A2|ATP5A1|FKBP1A|TF|PCYOX1|SLC4A4|COX7C|PHB2|ATP1A1|SLC25A10|COX4I1|PHB|PLCG2	2.26E-07	GO.0098660	0.5316953
402	25	GO Component	cytoplasmic region	0.0029	VCL|MAPK1|MYH9|PFN1|ENO1|DSTN|CANX|HSPB1|ACTN4|SOD1|UCHL1|CAPN2|FABP1|SCIN|PLS1|GNAI1|ANXA2|MYH10|OPA1|KRT19|FLNA|MYO6|ACTR2|SEPT7|RDX	0.00063	GO.0099568	0.2537602
42	11	GO Process	antibiotic catabolic process	5.3E-06	ALDH2|PRDX1|PRDX5|ADH5|PRDX3|PRDX6|ALDH1B1|ESD|AKR1C3|GPX3|GPX1	2.48E-07	GO.0017001	0.52781894
79	23	GO Component	respiratory chain complex	1.8E-13	NDUFB4|UQCRC1|PMPCB|NDUFA2|NDUFA10|SDHA|NDUFA9|UQCRC2|NDUFB9|NDUFB8|UQCRH|CYC1|COX5A|NDUFV1|NDUFA12|UQCR10|MT-CO1|MT-CO2|NDUFS1|BCS1L|NDUFA13|COX7C|COX4I1	1.06E-14	GO.0098803	1.27447275
1690	88	GO Process	nitrogen compound transport	5.5E-06	MYH9|RAB35|EHHADH|ATP6V1B1|CANX|HSPB1|PMPCB|MTX2|ACTN4|LRPPRC|ERP29|SLC9A3R1|NAPA|LRP2|ATP6V0A1|SLC27A2|RHCG|UQCRC2|TIMM44|ATP6V1A|ATP6V1B2|SLC25A4|ATP6V0D2|ATP6V0D1|HSPA9|AMN|HSP90B1|SLC23A1|YWHAG|ATP6V0A4|RAB1B|AQP1|DAB2|CRAT|PIPOX|SLC7A8|CALR|DHRS4|EHD3|AP2A2|RAB11B|ACAA1|HSP90AA1|RPS2|SLC25A15|SAMM50|AGK|PNP|ATP1B1|AHCYL1|MYO6|GNAS|SCP2|SLC25A5|PMPCA|YWHAB|RAB14|FLOT1|CUBN|SLC3A2|MPV17|AKR1C3|PITRM1|SLC25A6|AMACR|KRT18|DNM2|MVP|TSPO|TST|TF|SSB|SLC25A12|IDH1|PDZK1|SELENBP1|PNPT1|DDC|TOMM40|NDUFA13|VDAC3|EPHX2|IST1|PHB2|TIMM50|RBM8A|AP2B1|HAO2	2.59E-07	GO.0071705	0.52612194
318	29	GO Process	protein targeting	6.1E-06	EHHADH|PMPCB|MTX2|SLC27A2|UQCRC2|TIMM44|YWHAG|CRAT|PIPOX|DHRS4|ACAA1|HSP90AA1|RPS2|SAMM50|AGK|SCP2|PMPCA|YWHAB|MPV17|PITRM1|SLC25A6|AMACR|TSPO|IDH1|TOMM40|NDUFA13|EPHX2|TIMM50|HAO2	2.89E-07	GO.0006605	0.52160964

1236	70	GO Process	phosphorylation	6.2E-06	NDUFB4|UQCRC1|MAPK1|CTSH|OGDH|GAPDH|SKP1|ENO1|PMPCB|NDUFA2|NDUFA10|ADAM10|KHK|PEBP1|ATP5B|SDHA|NDUFA9|UQCRC2|PFKL|ERLIN2|NDUFB9|ATP5O|ATP5J2|NDUFB8|CKB|ATP5L|ATP5H|ATP5I|CYCS|UQCRH|PRKACA|GBAS|CYC1|COX5A|NDUFV1|NDUFA12|UQCR10|HSP90AA1|PKLR|PFKM|AGK|RAC1|STOML2|ATP5C1|MT-CO1|MT-CO2|ATP5F1|ALDH18A1|CMPK1|YWHAB|OGDHL|ALDOB|PFKP|AK3|NME2|DAK|AK4|ILK|ATP5A1|CDC42|NDUFS1|GK|HK1|CKMT2|CKMT1A|COX7C|CRYAB|COX4I1|GALK1|TTN	2.96E-07	GO.0016310	0.52083094
117	17	GO Process	vitamin metabolic process	6.2E-06	MTHFD1|PNPO|LRP2|ALDH1L1|MUT|AMN|SLC23A1|SHMT1|CYB5A|CYB5R3|GSTO1|AKR1A1|PCCA|PSAT1|CUBN|PC|PCCB	2.95E-07	GO.0006766	0.52083094
48	11	KEGG Pathways	Vibrio cholerae infection	6.3E-06	ATP6V1B1|ATP6V0A1|ATP6V1A|ATP6V1B2|ATP6V0D2|ATP6V0D1|ATP6V0A4|PRKACA|GNAS|ACTG1|PLCG2	7.79E-07	hsa05110	0.52006595
44	18	KEGG Pathways	Fatty acid degradation	1.5E-12	EHHADH|ACADS|ALDH2|CPT1A|ADH5|ECI1|ADH1B|CYP4A11|HADHB|ACAA1|ALDH3A2|ALDH9A1|ECHS1|ALDH1B1|HADHA|ALDH7A1|ACSL1|ACADVL	7.44E-14	hsa00071	1.18239087
2343	112	GO Process	cellular component assembly	6.5E-06	NDUFB4|VCL|DECR1|C1QBP|VTN|CTSC|SKP1|TUBA4A|VIL1|NDUFA2|ACTN4|NDUFA10|LAMA5|APCS|LAMC1|PSMD11|SLC9A3R1|NAPA|ATP6V0A1|ADD1|CPT1A|EIF4H|NDUFA9|PFKL|ECSIT|DPYS|NDUFB9|ACTC1|ATP6V0D1|HSPA9|COL1A2|HPRT1|NDUFB8|CCT2|HSP90B1|DCXR|LETM1|YWHAG|ATP6V0A4|PRKACA|RAB1B|ISCU|ACAD9|GRHPR|TLN1|SHMT1|CALR|NDUFV1|PLEC|DHRS4|EHD3|P4HB|NDUFA12|UQCR10|PARVA|HSP90AA1|PLS1|TUBB4B|EPB41L3|SAMM50|PFKM|ANXA2|GPX4|ANXA6|RAC1|STOML2|MYH10|LONP1|OPA1|COA3|KRT19|TGM2|IBA57|S100A10|FLNA|YWHAB|ALDOB|FLOT1|KCTD12|ACTR2|CD9|GPX3|DNM2|CORO1B|JUP|ACTN1|FLOT2|QPRT|MGST1|MYH11|ILK|SEPT7|FKBP1A|CDC42|RDX|ALAD|NDUFS1|CRYZ|PNPT1|COX20|FBP1|BCS1L|SERPINA1|ATPAF2|TIMMDC1|NDUFA13|CRYAB|IST1|DNM1L|ACTG1|TTN|AP2B1	3.14E-07	GO.0022607	0.51844223
148	19	GO Process	fatty acid derivative metabolic process	7E-06	OXCT1|LTA4H|BDH2|CYP4A11|CES2|PTGES2|GPX4|MGST3|HMGCS2|AKR1C3|BDH1|DPEP1|GGT5|GGT1|PTGR1|GPX1|ACSL1|EPHX2|ACSM2A	3.39E-07	GO.1901568	0.51536629
440	35	GO Process	heterocycle catabolic process	7.6E-06	AHCY|OGDH|GAPDH|DNPH1|ENO1|GDA|AMBP|PFKL|ALDH1L1|DPYS|HNMT|FTCD|HPRT1|PRODH2|HINT1|PKLR|RPS2|PFKM|PNP|OGDHL|ALDOB|ALDH4A1|DUT|PFKP|DPEP1|CNP|QPRT|AMPD3|SSB|PNPT1|HK1|GPX1|ALDH6A1|RBM8A|GALK1	3.68E-07	GO.0046700	0.51191864
79	14	GO Process	peroxisome organization	8.2E-06	EHHADH|SLC27A2|CRAT|PIPOX|DHRS4|ACAA1|ABCD3|SCP2|MPV17|AMACR|IDH1|EPHX2|DNM1L|HAO2	3.97E-07	GO.0007031	0.50888424
18	8	GO Process	branched-chain amino acid catabolic process	8.8E-06	HIBADH|BCKDHA|ACAD8|BCKDHB|BCAT2|AUH|IVD|ALDH6A1	4.28E-07	GO.0009083	0.50574959
26	9	GO Process	glycolytic process through glucose-6-phosphate	9E-06	GAPDH|ENO1|PFKL|PKLR|PFKM|ALDOB|PFKP|HK1|GALK1	4.43E-07	GO.0061620	0.50438316
44	11	GO Function	aminopeptidase activity	9.1E-06	CTSH|LTA4H|PEPD|ENPEP|RNPEP|ANPEP|DPP3|DPP4|XPNPEP2|DPEP1|PRCP	3.7E-07	GO.0004177	0.50390538
143	23	GO Component	cluster of actin-based cell projections	5E-09	VCL|MYH9|VIL1|ACTN4|SLC9A3R1|LRP2|ENPEP|SCIN|AMN|CA4|DCXR|ATP6V0A4|AQP1|PLEC|PLS1|MYH10|CLIC1|CUBN|ACTN1|RDX|PDZK1|MME|MYL6	4.17E-10	GO.0098862	0.83036436
68	13	GO Process	cell redox homeostasis	9.8E-06	PRDX1|NNT|PRDX5|PRDX3|PDIA3|P4HB|PRDX6|PTGES2|TXNDC5|GLRX|TXNRD2|PDIA6|GPX1	4.82E-07	GO.0045454	0.50109954
155	23	GO Component	azurophil granule	1.9E-08	MAPK1|CTSC|TTR|GGH|LYZ|CCT2|GAA|GLB1|TUBB4B|PRDX6|PTGES2|ANXA2|VAT1|PIGR|GM2A|CYB5R3|ACTR2|TXNDC5|PRCP|MGST1|NAPRT|IST1|NPC2	1.69E-09	GO.0042582	0.77281584
76	13	KEGG Pathways	Chemical carcinogenesis	1.1E-05	GSTM3|ADH5|UGT2B7|ADH1B|UGT2B17|GSTA1|UGT1A9|MGST3|GSTO1|MGST1|GSTP1|GSTA2|EPHX1	1.46E-06	hsa05204	0.4950782

36	10	GO Process	transferrin transport	1.1E-05	ATP6V1B1|ATP6V0A1|ATP6V1A|ATP6V1B2|ATP6V0D2|ATP6V0D1|ATP6V0A4|RAB11B|DNM2|TF	5.54E-07	GO.0033572	0.4950782
70	13	GO Process	peroxisomal transport	1.3E-05	EHHADH|SLC27A2|CRAT|PIPOX|DHRS4|ACAA1|ABCD3|SCP2|MPV17|AMACR|IDH1|EPHX2|HAO2	6.44E-07	GO.0043574	0.489279
493	37	GO Process	actin filament-based process	1.3E-05	RALA|MYH9|PFN1|DSTN|VIL1|ACTN4|SLC9A3R1|EHD2|ADD1|IQGAP2|GPD1L|ACTC1|SCIN|HSP90B1|AQP1|TLN1|CALR|PARVA|PLS1|EPB41L3|TPM4|RAC1|MYH10|KRT19|FLNA|MYO6|ACTR2|CORO1B|ACTN1|MYH11|CDC42|ARPC1B|ATP1A1|VIM|MYL6|ACTG1|TTN	6.4E-07	GO.0030029	0.489279
295	27	GO Process	regulation of neurotransmitter levels	1.4E-05	AGXT2|SPR|HRSP12|DMGDH|NAPA|AMT|BHMT|DPYS|HNMT|DDAH1|SHMT1|CHDH|HSP90AA1|ALDH9A1|PHGDH|SARDH|ASS1|MAOB|GLDC|DNM2|TSPO|ABAT|ALDH7A1|DDC|PAH|DNM1L|GLYAT	6.95E-07	GO.0001505	0.48632794
373	31	GO Process	response to oxidative stress	1.4E-05	NDUFB4|MAPK1|PPIF|PRDX1|PRDX5|SOD1|FABP1|PRDX3|CYCS|AQP1|MSRA|NDUFA12|PRDX6|GPX4|LONP1|MT-CO1|MPV17|AKR1C3|GPX3|DNM2|DPEP1|MGST1|GSTP1|TXNRD2|ALAD|IDH1|PNPT1|NAPRT|GPX1|ETFDH|CRYAB	7.29E-07	GO.0006979	0.48416375
296	27	GO Process	extracellular matrix organization	1.5E-05	DCN|NID2|VTN|TTR|LAMA5|LAMC1|ADAM10|VCAN|LUM|COL1A2|COL14A1|FBN1|BGN|TPSAB1|ANXA2|COL18A1|COL4A2|DPT|HSPG2|COL4A1|FLOT1|POSTN|MPV17|MYH11|TGFBI|VWA1|ITGB3	7.38E-07	GO.0030198	0.4838632
187	21	GO Function	carboxylic acid binding	1.5E-05	C1QBP|GOT2|HRSP12|VCAN|DPYS|DDAH1|FTCD|FABP1|ADH5|GRHPR|SHMT1|PCK1|SERPINA5|UGT1A9|GOT1|SCP2|ASS1|PCCA|GLDC|PC|DDC	6.21E-07	GO.0031406	0.48239087
390	32	GO Function	monovalent inorganic cation transmembrane transporter activity	1.5E-05	SLC25A3|ATP6V1B1|ATP5B|ATP6V0A1|NNT|ATP6V1A|ATP6V1B2|ATP6V0D2|ATP5O|ATP6V0D1|COX7A1|ATP5L|ATP5H|SLC23A1|LETM1|ATP5I|ATP6V0A4|AQP1|COX5A|SLC5A2|CYB5A|ATP5C1|MT-CO1|MT-CO2|ATP1B1|ATP5F1|SLC3A2|ATP5A1|SLC4A4|COX7C|ATP1A1|COX4I1	6.27E-07	GO.0015077	0.48239087
7	6	GO Process	amino-acid betaine biosynthetic process	1.5E-05	BBOX1|SHMT1|CHDH|TMLHE|ALDH9A1|ALDH7A1	7.78E-07	GO.0006578	0.48181564
720	47	GO Process	cation transmembrane transport	1.7E-05	SLC25A3|ATP6V1B1|PMPCB|ATP5B|ATP6V0A1|NNT|RHCG|AFG3L2|ATP6V1A|ATP6V1B2|ATP6V0D2|ATP5O|ATP6V0D1|ATP5J2|COX7A1|ATP5L|ATP5H|SLC23A1|LETM1|ATP5I|ATP6V0A4|AQP1|CYC1|COX5A|SLC7A8|SLC5A2|CYB5A|SLC25A15|STOML2|ATP5C1|MT-CO1|OPA1|MT-CO2|ATP1B1|ATP5F1|PMPCA|SLC3A2|ATP5A1|FKBP1A|TF|SLC4A4|COX7C|PHB2|ATP1A1|COX4I1|PHB|PLCG2	8.64E-07	GO.0098655	0.47746907
34	8	KEGG Pathways	Pentose and glucuronate interconversions	0.00013	SORD|CRYL1|DCXR|UGT2B7|UGDH|UGT2B17|UGT1A9|AKR1A1	2.16E-05	hsa00040	0.38860566
97	22	GO Component	brush border	4.4E-11	VCL|MYH9|VIL1|ACTN4|SLC9A3R1|LRP2|ENPEP|SCIN|AMN|CA4|DCXR|ATP6V0A4|AQP1|PLEC|PLS1|MYH10|CLIC1|CUBN|ACTN1|PDZK1|MME|MYL6	2.95E-12	GO.0005903	1.03545777
339	29	GO Process	extracellular structure organization	1.9E-05	DCN|NID2|VTN|TTR|LAMA5|LAMC1|ADAM10|VCAN|LUM|COL1A2|COL14A1|PRKACA|FBN1|BGN|P4HB|TPSAB1|ANXA2|COL18A1|COL4A2|DPT|HSPG2|COL4A1|FLOT1|POSTN|MPV17|MYH11|TGFBI|VWA1|ITGB3	9.71E-07	GO.0043062	0.47258422
39	10	GO Process	trivalent inorganic cation transport	2E-05	ATP6V1B1|ATP6V0A1|ATP6V1A|ATP6V1B2|ATP6V0D2|ATP6V0D1|ATP6V0A4|RAB11B|DNM2|TF	1.03E-06	GO.0072512	0.47011469
21	8	GO Process	mitochondrial calcium ion transmembrane transport	2.1E-05	PMPCB|AFG3L2|LETM1|STOML2|OPA1|PMPCA|PHB2|PHB	1.09E-06	GO.0006851	0.46798537

<table>
<tr><td>2268</td><td>107</td><td>GO Process</td><td>macromolecule localization</td><td>2.1E-05</td><td>VCL|MYH9|RAB35|SKP1|EHHADH|ATP6V1B1|GOT2|CANX|HSPB1|PMPCB|MTX2|ACTN4|LAMA5|LRPPRC|ERP29|SLC9A3R1|EHD2|NAPA|CD81|LRP2|ATP6V0A1|CPT1A|SLC27A2|UQCRC2|TIMM44|ATP6V1A|ATP6V1B2|ATP6V0D2|ATP6V0D1|FABP1|SCIN|HSPA9|AMN|CCT2|HSP90B1|YWHAG|ATP6V0A4|RAB1B|DAB2|CRAT|TLN1|PIPOX|CALR|HSPA5|FBN1|DHRS4|EHD3|AP2A2|SERPINA5|RAB11B|ACAA1|HSP90AA1|RPS2|EPB41L3|SAMM50|ANXA2|AGK|STOML2|PIGR|GM2A|PNP|ATP1B1|S100A10|AHCYL1|FLNA|MYO6|ABCD3|GNAS|RBP4|SCP2|PMPCA|YWHAB|RAB14|FLOT1|CUBN|APOO|MPV17|AKR1C3|PITRM1|SLC25A6|AMACR|KRT18|DNM2|CORO1B|JUP|FLOT2|MVP|TSPO|RDX|TST|TF|SSB|IDH1|SELENBP1|PNPT1|HK1|TOMM40|ACSL1|NDUFA13|EPHX2|IST1|PHB2|TIMM50|NPC2|RBM8A|AP2B1|HAO2</td><td>1.12E-06</td><td>GO.0033036</td><td>0.46695862</td></tr>
<tr><td>61</td><td>12</td><td>GO Function</td><td>cation-transporting ATPase activity</td><td>2.3E-05</td><td>ATP5B|ATP6V0A1|ATP6V1A|ATP6V1B2|ATP6V0D2|ATP5O|ATP6V0D1|ATP6V0A4|ATP5C1|ATP1B1|ATP5A1|ATP1A1</td><td>1.02E-06</td><td>GO.0019829</td><td>0.46326441</td></tr>
<tr><td>51</td><td>11</td><td>GO Process</td><td>NAD biosynthetic process</td><td>2.5E-05</td><td>GAPDH|ENO1|PFKL|PKLR|PFKM|PNP|ALDOB|PFKP|QPRT|NAPRT|HK1</td><td>1.31E-06</td><td>GO.0009435</td><td>0.46038007</td></tr>
<tr><td>214</td><td>22</td><td>GO Process</td><td>protein folding</td><td>2.5E-05</td><td>PPIF|MLEC|TRAP1|CANX|HSPB1|APCS|ERP29|CCT5|HSPA9|CCT2|HSP90B1|PPIB|PDIA3|CALR|HSPA5|P4HB|HSP90AA1|GNAI1|TXNDC5|FKBP1A|PDIA6|CRYAB</td><td>1.31E-06</td><td>GO.0006457</td><td>0.46038007</td></tr>
<tr><td>125</td><td>22</td><td>GO Component</td><td>ficolin-1-rich granule lumen</td><td>2.8E-09</td><td>VCL|MAPK1|CTSH|LTA4H|CTSD|PSMD11|COTL1|PSMD3|PFKL|GLB1|HSP90AA1|CTSB|PNP|ACTR2|NME2|JUP|MVP|AMPD3|GSTP1|ALAD|IDH1|SERPINA1</td><td>2.21E-10</td><td>GO.1904813</td><td>0.85543958</td></tr>
<tr><td>5382</td><td>212</td><td>GO Function</td><td>organic cyclic compound binding</td><td>2.7E-05</td><td>RALA|MAPK1|RTCB|MYH9|MTHFD1|PCK2|AHCY|IDH3G|PGRMC1|CRYM|DECR1|OGDH|PNPO|PFN1|C1QBP|GAPDH|RAB35|AGXT2|LTF|ATP6V1B1|SPR|ENO1|ACADS|GOT2|TRAP1|TUBA4A|ACTN4|GRSF1|CARS2|TINAG|KHK|LRPPRC|PEBP1|ALDH2|ATP5B|EHD2|NNT|SDHA|ACAD11|AMBP|HIBADH|CALB1|PRDX5|EIF4H|REXO2|SORD|SLC27A2|AFG3L2|PFKL|TIMM44|ATP6V1A|MUT|ATP6V1B2|ERLIN2|LACTB2|DPYS|CCT5|ACAD8|QDPR|GPD1L|ACTC1|FTCD|BDH2|HSPA9|ALDH1A1|CRYL1|HPRT1|CKB|CCT2|IDH3A|HSP90B1|LDHD|TUBA1C|GPD1|PRODH2|HINT1|MRPL39|MRPL13|CYCS|ACO1|PRKACA|RAB1B|CYP4A11|ACAD9|GRHPR|TSFM|ACSS1|CYC1|SHMT1|UGDH|PCK1|CHDH|CALR|RHOT2|TUFM|NDUFV1|HSPA5|EHD3|ACSM2B|IDH2|RAB11B|MRPL12|HSP90AA1|PKLR|TUBB4B|CYB5A|RPS2|GNAI1|PTGES2|PFKM|ANXA6|AGK|RAC1|MSN|MYH10|LONP1|CYB5R3|MT-CO1|PNP|OPA1|DARS2|TGM2|MARC2|IARS2|PHGDH|AHCYL1|MYO6|ABCD3|GOT1|GNAS|ALDH18A1|SCP2|CMPK1|ASS1|VDAC2|RAB14|OGDHL|VARS|AUH|PCCA|ALDH1B1|CUBN|SLC3A2|ACTR2|MAOB|SUCLA2|HBD|NQO2|HADHA|IDH3B|PFKP|GLDC|AK3|DNM2|NME2|CNP|PC|SUOX|ACTN1|DAK|AK4|TSPO|MYH11|ABAT|ILK|ATP5A1|SEPT7|FKBP1A|CDC42|TXNRD2|TST|SSB|IDH1|CRYZ|PNPT1|GK|ACSF2|HK1|ACTA2|DDC|CKMT2|CKMT1A|FBP1|LARS2|BCS1L|IVD|PCCB|GFM1|SSBP1|ACSL1|NDUFA13|ETFDH|VDAC3|ACADVL|ME3|ATP1A1|TIMM50|VIM|DNM1L|ALDH6A1|NPC2|ETFA|ACTG1|ACSM2A|RBM8A|GALK1|TTN|SARS2|SIRT5|PHB|FMO1|HAO2</td><td>1.24E-06</td><td>GO.0097159</td><td>0.45734887</td></tr>
<tr><td>283</td><td>22</td><td>GO Component</td><td>extracellular matrix</td><td>0.00044</td><td>NID2|VTN|LAMA5|LAMC1|TINAG|LUM|SOD1|COL1A2|COL14A1|CALR|FBN1|BGN|ANXA2|COL18A1|COL4A2|HSPG2|ASPN|COL4A1|POSTN|AHSG|TGFBI|VWA1</td><td>7.46E-05</td><td>GO.0031012</td><td>0.33565473</td></tr>
<tr><td>8</td><td>6</td><td>GO Function</td><td>oxidoreductase activity, acting on the aldehyde or oxo group of donors, disulfide as acceptor</td><td>2.8E-05</td><td>OGDH|BCKDHA|PDHB|BCKDHB|OGDHL|PDHA1</td><td>1.33E-06</td><td>GO.0016624</td><td>0.45482136</td></tr>
</table>

1524	79	GO Function	adenyl nucleotide binding	2.9E-05	MAPK1|RTCB|MYH9|MTHFD1|AHCY|IDH3G|PFN1|ATP6V1B1|TRAP1|CARS2|KHK|PEBP1|ATP5B|EHD2|SLC27A2|AFG3L2|PFKL|TIMM44|ATP6V1A|ATP6V1B2|CCT5|ACTC1|HSPA9|CKB|CCT2|HSP90B1|PRKACA|ACSS1|HSPA5|EHD3|ACSM2B|HSP90AA1|PKLR|PFKM|AGK|MYH10|LONP1|CYB5R3|DARS2|IARS2|MYO6|ABCD3|ALDH18A1|SCP2|CMPK1|ASS1|VARS|PCCA|ACTR2|SUCLA2|HADHA|PFKP|AK3|NME2|PC|DAK|AK4|MYH11|ILK|ATP5A1|GK|ACSF2|HK1|ACTA2|CKMT2|CKMT1A|FBP1|LARS2|BCS1L|PCCB|ACSL1|NDUFA13|ATP1A1|ALDH6A1|ACTG1|ACSM2A|GALK1|TTN|SARS2	1.38E-06	GO.0030554	0.45421181
52	11	GO Process	cofactor catabolic process	2.9E-05	AHCY|PRDX1|AMBP|PRDX5|ALDH1L1|PRDX3|PRDX6|GPX3|GGT5|GGT1|GPX1	1.55E-06	GO.0051187	0.4536107
6607	250	GO Function	protein binding	3.4E-05	RALA|DCN|OXCT1|UQCRC1|VCL|MAPK1|LGALS1|RTCB|MYH9|GSTZ1|AHCY|CRYM|OGDH|PNPO|PFN1|C1QBP|VTN|CTSC|MLEC|GAPDH|DNPH1|AGXT2|SKP1|EHHADH|ENO1|TTR|GOT2|DSTN|TRAP1|CANX|TUBA4A|VIL1|HSPB1|ACTN4|LAMA5|LGALS3|HRSP12|APCS|GSTM3|ADAM10|LRPPRC|LYZ|PEBP1|ERP29|ATP5B|COTL1|CHCHD3|SLC9A3R1|PRDX1|BBOX1|EHD2|NAPA|CD81|LRP2|ATP6V0A1|ADD1|AMBP|VDAC1|PRDX5|CPT1A|SORD|SLC27A2|RHCG|AFG3L2|PFKL|SOD1|BCAM|TIMM44|TINAGL1|IQGAP2|MUT|ERLIN2|DPYS|CCT5|DLAT|QDPR|GPD1L|HGD|UCHL1|MRPL17|ACTC1|FTCD|CAPN2|ADH5|SCIN|HSPA9|COL1A2|COL14A1|CRYL1|PRDX3|HPRT1|AMN|CKB|CCT2|HSP90B1|PPIB|PDIA3|GPD1|DCXR|HINT1|YWHAG|GLB1|ASL|ATP6V0A4|DPYSL2|PRKACA|ISCU|AQP1|AOC3|DAB2|GRHPR|CRAT|TLN1|PIPOX|SHMT1|CALR|PLEC|HSPA5|FBN1|DHRS4|MDH2|AP2A2|P4HB|SERPINA5|RAB11B|PARVA|HSP90AA1|PLS1|TUBB4B|CYB5A|RPS2|PRDX6|GNAI1|EPB41L3|TPM4|CTSB|PFKM|ALDH3A2|ANXA2|GPX4|ANXA6|UGT1A9|COL18A1|RAC1|STOML2|PBLD|MSN|MYH10|DPP4|LONP1|OPA1|DARS2|TGM2|AKR7A3|ATP1B1|S100A10|LHPP|AHCYL1|FLNA|MYO6|ABCD3|HOGA1|GNAS|ALDH18A1|RBP4|SCP2|SLC25A5|ASS1|YWHAB|RAB14|CISD1|HSPG2|ALDOB|ALDH4A1|COL4A1|PCCA|FLOT1|KCTD12|CUBN|ACTR2|MAOB|ESD|GLRX|NQO2|PFKP|GLDC|AMACR|CD9|GPX3|KRT18|DNM2|CORO1B|JUP|PC|FHL1|ACTN1|FLOT2|MVP|QPRT|SNTB1|MGST1|TSPO|MYH11|ABAT|ILK|MPST|GSTP1|ATP5A1|SEPT7|FKBP1A|CDC42|RDX|ACY1|TF|ALAD|SLC25A12|ARPC1B|IDH1|TKT|SLC4A4|PDZK1|HK1|ACTA2|DDC|NDRG1|GPX1|FBP1|AOC1|SERPINA1|TGFBI|VWA1|EPHX2|TAGLN|CRYAB|RAP1GAP|IST1|PHB2|ATP1A1|TIMM50|VIM|DNM1L|NPC2|ITGB3|ACTG1|TTN|PHB|PDLIM5|PLCG2|AP2B1|HAO2	1.63E-06	GO.0005515	0.44749552
72	12	KEGG Pathways	PPAR signaling pathway	3.4E-05	PCK2|EHHADH|CPT1A|SLC27A2|FABP1|PCK1|ACAA1|HMGCS2|SCP2|ILK|GK|ACSL1	4.7E-06	hsa03320	0.44672456
53	11	GO Process	sulfur compound catabolic process	3.4E-05	DCN|AHCY|VCAN|LUM|GLB1|BGN|MPST|GGT5|GGT1|TST|PCYOX1	1.82E-06	GO.0044273	0.44659739
298	22	GO Component	secretory granule membrane	0.00083	PGRMC1|MLEC|LGALS3|ADAM10|ATP6V0A1|SLC27A2|IQGAP2|ANPEP|CA4|GAA|AP2A2|SERPINA5|RAC1|PIGR|RAB14|CD9|PRCP|FLOT2|MGST1|MME|MGAM|ITGB3	0.00015	GO.0030667	0.30809219
591	40	Reactome Pathways	Hemostasis	3.8E-05	VCL|MAPK1|F9|PFN1|C1QBP|TUBA4A|ACTN4|EHD2|F13A1|SOD1|COL1A2|TUBA1C|PRKACA|TLN1|SLC7A8|HSPA5|EHD3|SERPINA5|TUBB4B|GNAI1|ANXA2|RAC1|ATP1B1|S100A10|FLNA|GNAS|APOOL|SLC3A2|HBD|AK3|CD9|PRCP|ACTN1|CDC42|TF|AHSG|SERPINA1|ITGB3|TTN|PLCG2	2.55E-06	HSA-109582	0.44168012
609	41	GO Process	import into cell	4E-05	MAPK1|MYH9|VTN|LTF|CANX|TINAG|EHD2|CD81|LRP2|AMBP|SLC27A2|TINAGL1|AMN|HSP90B1|DPYSL2|DAB2|CALR|AP2A2|HSP90AA1|RAC1|TGM2|ATP1B1|MYO6|HSPG2|CUBN|SLC3A2|ACTR2|TXNDC5|CD9|DNM2|CDC42|PDIA6|ARPC1B|AHSG|ACSL1|ATP1A1|DNM1L|ITGB3|ACTG1|PLCG2|AP2B1	2.16E-06	GO.0098657	0.43936186
61	11	KEGG Pathways	Synaptic vesicle cycle	4.3E-05	ATP6V1B1|NAPA|ATP6V0A1|ATP6V1A|ATP6V1B2|ATP6V0D2|ATP6V0D1|ATP6V0A4|AP2A2|DNM2|AP2B1	6.01E-06	hsa04721	0.43716111

396	31	GO Process	establishment of protein localization to organelle	4.4E-05	EHHADH|PMPCB|MTX2|SLC27A2|UQCRC2|TIMM44|CRAT|PIPOX|DHRS4|ACAA1|HSP90AA1|RPS2|SAMM50|AGK|SCP2|PMPCA|MPV17|AKR1C3|PITRM1|SLC25A6|AMACR|TSPO|IDH1|HK1|TOMM40|NDUFA13|EPHX2|PHB2|TIMM50|AP2B1|HAO2	2.34E-06	GO.0072594	0.43605135
988	57	GO Process	response to nitrogen compound	4.5E-05	UQCRC1|MAPK1|ATP6V1B1|GOT2|GSTM3|GGH|KHK|CD81|ATP6V0A1|SOD1|ATP6V1A|ATP6V1B2|ERLIN2|QDPR|ATP6V0D2|ATP6V0D1|CAPN2|COL1A2|HPRT1|HSP90B1|GPD1|YWHAG|ATP6V0A4|PRKACA|AQP1|CYC1|SHMT1|PCK1|HSPA5|FBN1|PKLR|GNAI1|OPA1|MARC2|MGST3|AHCYL1|GOT1|GNAS|ASS1|COL4A1|FLOT1|SLC3A2|ACTR2|HADHA|GLDC|CD9|DNM2|DPEP1|JUP|MGST1|TSPO|ABAT|GSTP1|ALAD|PNPT1|DDC|AOC1	2.4E-06	GO.1901698	0.43506651
24	8	GO Process	glucuronate metabolic process	4.6E-05	SORD|ABHD10|CRYL1|DCXR|UGT2B7|UGT2B17|UGT1A9|AKR1A1	2.47E-06	GO.0019585	0.43391345
418	32	GO Process	actin cytoskeleton organization	4.6E-05	RALA|MYH9|PFN1|DSTN|VIL1|ACTN4|SLC9A3R1|EHD2|ADD1|IQGAP2|ACTC1|SCIN|HSP90B1|AQP1|TLN1|CALR|PARVA|PLS1|EPB41L3|TPM4|RAC1|MYH10|KRT19|FLNA|ACTR2|CORO1B|ACTN1|MYH11|CDC42|ARPC1B|ACTG1|TTN	2.5E-06	GO.0030036	0.43381873
16	7	GO Process	glycine metabolic process	4.6E-05	AGXT2|HRSP12|AMT|SHMT1|PHGDH|GLDC|GLYAT	2.51E-06	GO.0006544	0.43372422
330	22	GO Component	intrinsic component of organelle membrane	0.0026	RAB35|CANX|CHCHD3|CPT1A|SLC27A2|AFG3L2|SLC25A4|C19orf70|TMEM11|CALR|RHOT2|HSPA5|RAB11B|AGK|MPC1|COA3|APOOL|APOO|SACM1L|IMMT|TOMM40|ETFDH	0.00056	GO.0031300	0.25850267
5305	208	GO Function	heterocyclic compound binding	4.8E-05	RALA|MAPK1|RTCB|MYH9|MTHFD1|PCK2|AHCY|IDH3G|PGRMC1|CRYM|DECR1|OGDH|PNPO|PFN1|C1QBP|GAPDH|RAB35|AGXT2|LTF|ATP6V1B1|SPR|ENO1|ACADS|GOT2|TRAP1|TUBA4A|ACTN4|GRSF1|CARS2|TINAG|KHK|LRPPRC|PEBP1|ALDH2|ATP5B|EHD2|NNT|SDHA|ACAD11|AMBP|HIBADH|PRDX5|EIF4H|REXO2|SORD|SLC27A2|AFG3L2|PFKL|TIMM44|ATP6V1A|MUT|ATP6V1B2|LACTB2|DPYS|CCT5|ACAD8|QDPR|GPD1L|ACTC1|FTCD|BDH2|HSPA9|ALDH1A1|CRYL1|HPRT1|CKB|CCT2|IDH3A|HSP90B1|LDHD|TUBA1C|GPD1|PRODH2|HINT1|MRPL39|MRPL13|CYCS|ACO1|PRKACA|RAB1B|CYP4A11|ACAD9|GRHPR|TSFM|ACSS1|CYC1|SHMT1|UGDH|PCK1|CHDH|CALR|RHOT2|TUFM|NDUFV1|HSPA5|EHD3|ACSM2B|IDH2|RAB11B|MRPL12|HSP90AA1|PKLR|TUBB4B|CYB5A|RPS2|GNAI1|PTGES2|PFKM|ANXA6|AGK|RAC1|MSN|MYH10|LONP1|CYB5R3|MT-CO1|PNP|OPA1|DARS2|TGM2|MARC2|IARS2|PHGDH|AHCYL1|MYO6|ABCD3|GOT1|GNAS|ALDH18A1|SCP2|CMPK1|ASS1|VDAC2|RAB14|OGDHL|VARS|AUH|PCCA|ALDH1B1|CUBN|SLC3A2|ACTR2|MAOB|SUCLA2|HBD|NQO2|HADHA|IDH3B|PFKP|GLDC|AK3|DNM2|NME2|CNP|PC|SUOX|ACTN1|DAK|AK4|MYH11|ABAT|ILK|ATP5A1|SEPT7|FKBP1A|CDC42|TXNRD2|TST|SSB|IDH1|CRYZ|PNPT1|GK|ACSF2|HK1|ACTA2|DDC|CKMT2|CKMT1A|FBP1|LARS2|BCS1L|IVD|PCCB|GFM1|SSBP1|ACSL1|NDUFA13|ETFDH|VDAC3|ACADVL|ME3|ATP1A1|TIMM50|VIM|DNM1L|ALDH6A1|ETFA|ACTG1|ACSM2A|RBM8A|GALK1|TTN|SARS2|SIRT5|PHB|FMO1|HAO2	2.36E-06	GO.1901363	0.4322393
896	53	GO Process	cellular response to oxygen-containing compound	5.1E-05	MAPK1|LGALS1|PPIF|ATP6V1B1|PRDX1|ATP6V0A1|PRDX5|CPT1A|SOD1|ATP6V1A|ATP6V1B2|ATP6V0D2|ATP6V0D1|CAPN2|FABP1|ADH5|COL1A2|PRDX3|HSP90B1|GPD1|YWHAG|ATP6V0A4|PRKACA|AQP1|SHMT1|PCK1|HSPA5|FBN1|RAB11B|PKLR|GNAI1|MSN|OPA1|AHCYL1|GOT1|GNAS|ASS1|COL4A1|ACTR2|ESD|MPV17|AKR1C3|FECH|DNM2|DPEP1|JUP|MGST1|TSPO|GSTP1|AOC1|NDUFA13|PHB2|DNM1L	2.8E-06	GO.1901701	0.4294992
68	12	GO Process	protein targeting to peroxisome	5.1E-05	EHHADH|SLC27A2|CRAT|PIPOX|DHRS4|ACAA1|SCP2|MPV17|AMACR|IDH1|EPHX2|HAO2	2.78E-06	GO.0006625	0.4294992
317	21	GO Component	cell projection membrane	0.0036	RAB35|SLC9A3R1|LRP2|AMN|CA4|ATP6V0A4|AQP1|TLN1|EHD3|HSP90AA1|EPB41L3|RAC1|MSN|DPP4|MYO6|CUBN|DNM2|DPEP1|UMOD|PDZK1|ITGB3	0.0008	GO.0031253	0.24436975
90	13	KEGG Pathways	Protein digestion and absorption	5.3E-05	COL1A2|COL14A1|SLC7A8|COL18A1|COL4A2|DPP4|ATP1B1|XPNPEP2|COL4A1|SLC3A2|PRCP|MME|ATP1A1	7.73E-06	hsa04974	0.42724587

300	20	GO Component	membrane raft	0.0044	VCL|MAPK1|CTSD|SLC9A3R1|EHD2|VDAC1|ERLIN2|CAPN2|PRKACA|GNAI1|ANXA2|STOML2|DPP4|ATP1B1|SLC25A5|VDAC2|FLOT1|FLOT2|PDZK1|ATP1A1	0.00098	GO.0045121	0.23565473
25	8	GO Process	canonical glycolysis	5.7E-05	GAPDH|ENO1|PFKL|PKLR|PFKM|ALDOB|PFKP|HK1	3.19E-06	GO.0061621	0.42433639
876	52	GO Process	response to organonitrogen compound	5.7E-05	UQCRC1|MAPK1|ATP6V1B1|GOT2|GGH|KHK|CD81|ATP6V0A1|SOD1|ATP6V1A|ATP6V1B2|ERLIN2|QDPR|ATP6V0D2|ATP6V0D1|CAPN2|COL1A2|HPRT1|HSP90B1|GPD1|YWHAG|ATP6V0A4|PRKACA|AQP1|CYC1|SHMT1|PCK1|HSPA5|FBN1|PKLR|GNAI1|OPA1|MGST3|AHCYL1|GOT1|GNAS|ASS1|COL4A1|ACTR2|HADHA|GLDC|CD9|DNM2|JUP|MGST1|TSPO|ABAT|GSTP1|ALAD|PNPT1|DDC|AOC1	3.17E-06	GO.0010243	0.42433639
1560	79	GO Process	immune response	5.7E-05	VCL|MAPK1|LGALS1|AHCY|PGRMC1|CTSH|C1QBP|VTN|CTSC|MLEC|LTA4H|GAPDH|LTF|CTSD|TTR|LGALS3|APCS|TINAG|GGH|ADAM10|LYZ|PSMD11|COTL1|PRDX1|PSMD3|ATP6V0A1|SLC27A2|PFKL|ECSIT|TINAGL1|IQGAP2|HPRT1|CCT2|ANPEP|GAA|GLB1|AP2A2|ACAA1|HSP90AA1|TUBB4B|PRDX6|PTGES2|CTSB|ANXA2|VAT1|RAC1|PIGR|GM2A|CYB5R3|PNP|C4BPA|ASS1|RAB14|ACTR2|TXNDC5|NME2|PRCP|JUP|NIT2|DAK|MVP|MGST1|AMPD3|GSTP1|IFI30|ALAD|IDH1|AHSG|NAPRT|AOC1|SERPINA1|MME|IST1|VIM|MGAM|METTL7A|NPC2|ACTG1|PLCG2	3.2E-06	GO.0006955	0.42433639
25	8	GO Process	NADH regeneration	5.7E-05	GAPDH|ENO1|PFKL|PKLR|PFKM|ALDOB|PFKP|HK1	3.19E-06	GO.0006735	0.42433639
310	20	GO Component	integral component of organelle membrane	0.0059	CANX|CHCHD3|CPT1A|SLC27A2|AFG3L2|SLC25A4|C19orf70|TMEM11|CALR|RHOT2|HSPA5|AGK|MPC1|COA3|APOOL|APOO|SACM1L|IMMT|TOMM40|ETFDH	0.0014	GO.0031301	0.2229148
383	30	GO Process	supramolecular fiber organization	6E-05	DSTN|VIL1|ACTN4|ADD1|LUM|IQGAP2|ACTC1|SCIN|COL1A2|COL14A1|HSP90B1|PLS1|TPM4|ANXA2|RAC1|MYH10|KRT19|DPT|FLNA|ACTR2|CORO1B|ACTN1|MYH11|ILK|FKBP1A|CDC42|ARPC1B|VIM|ACTG1|TTN	3.37E-06	GO.0097435	0.4225483
10	6	GO Process	aldehyde catabolic process	6E-05	AGXT2|ADH5|HOGA1|AKR1A1|ESD|HAGH	3.37E-06	GO.0046185	0.4225483
158	18	GO Function	actin filament binding	6.3E-05	MYH9|DSTN|VIL1|ACTN4|ADD1|IQGAP2|SCIN|TLN1|PLS1|TPM4|MYH10|FLNA|MYO6|CORO1B|ACTN1|MYH11|TAGLN|TTN	3.25E-06	GO.0051015	0.42027325
10	6	GO Function	hydrolase activity, acting on ether bonds	6.3E-05	AHCY|LTA4H|AKR7A2|RNPEP|EPHX2|EPHX1	3.37E-06	GO.0016801	0.42027325
45	10	GO Function	NADH dehydrogenase (ubiquinone) activity	6.3E-05	NDUFB4|NDUFA2|NDUFA10|NDUFA9|NDUFB9|NDUFB8|NDUFV1|NDUFA12|NDUFS1|NDUFA13	3.15E-06	GO.0008137	0.42027325
25	8	GO Function	glutathione transferase activity	6.3E-05	GSTZ1|GSTM3|GSTA1|MGST3|GSTO1|MGST1|GSTP1|GSTA2	3.19E-06	GO.0004364	0.42027325
25	8	GO Function	aldo-keto reductase (NADP) activity	6.3E-05	SPR|AKR7A2|DHRS4|CYB5A|AKR7A3|AKR1A1|AKR1C3|RDH13	3.19E-06	GO.0004033	0.42027325
46	10	GO Function	NADP binding	6.8E-05	CRYM|DECR1|GAPDH|SPR|NNT|QDPR|GRHPR|IDH1|CRYZ|FMO1	3.74E-06	GO.0050661	0.41706962
1514	77	GO Function	adenyl ribonucleotide binding	6.8E-05	MAPK1|RTCB|MYH9|MTHFD1|IDH3G|ATP6V1B1|TRAP1|CARS2|KHK|PEBP1|ATP5B|EHD2|SLC27A2|AFG3L2|PFKL|TIMM44|ATP6V1A|ATP6V1B2|CCT5|ACTC1|HSPA9|CKB|CCT2|HSP90B1|PRKACA|ACSS1|HSPA5|EHD3|ACSM2B|HSP90AA1|PKLR|PFKM|AGK|MYH10|LONP1|CYB5R3|DARS2|IARS2|MYO6|ABCD3|ALDH18A1|SCP2|CMPK1|ASS1|VARS|PCCA|ACTR2|SUCLA2|HADHA|PFKP|AK3|NME2|PC|DAK|AK4|MYH11|ILK|ATP5A1|GK|ACSF2|HK1|ACTA2|CKMT2|CKMT1A|FBP1|LARS2|BCS1L|PCCB|ACSL1|NDUFA13|ATP1A1|ALDH6A1|ACTG1|ACSM2A|GALK1|TTN|SARS2	3.73E-06	GO.0032559	0.41706962
212	21	GO Function	transferase activity, transferring acyl groups	6.8E-05	PDHX|F13A1|CPT1A|NAT8|DLAT|OXSM|GLYATL1|CRAT|HADHB|ACSM2B|ACAA1|DLST|CS|TGM2|HMGCS2|SCP2|HADHA|GGT5|GGT1|ACSM2A|GLYAT	3.89E-06	GO.0016746	0.41706962

882	52	GO Function	cytoskeletal protein binding	6.8E-05	RALA|VCL|RTCB|MYH9|PFN1|GAPDH|DSTN|VIL1|ACTN4|LRPPRC|COTL1|SLC9A3R1|ADD1|RHCG|IQGAP2|CCT5|ACTC1|FTCD|CAPN2|SCIN|DPYSL2|TLN1|PLEC|RAB11B|PARVA|HSP90AA1|PLS1|EPB41L3|TPM4|ANXA2|MSN|MYH10|OPA1|FLNA|MYO6|RAB14|ALDOB|ACTR2|DNM2|CORO1B|ACTN1|SNTB1|MYH11|RDX|ARPC1B|NDRG1|TAGLN|CRYAB|ATP1A1|DNM1L|TTN|PDLIM5	3.82E-06	GO.0008092	0.41706962
123	15	KEGG Pathways	Lysosome	6.9E-05	CTSH|NAGLU|CTSC|CTSD|NAPSA|ATP6V0A1|ATP6V0D2|ATP6V0D1|TPP1|GAA|GLB1|ATP6V0A4|CTSB|GM2A|NPC2	1.02E-05	hsa04142	0.41643094
603	40	GO Function	peptidase activity, acting on L-amino acid peptides	6.9E-05	UQCRC1|F9|CTSH|CTSC|LTA4H|LTF|CTSD|PEPD|PMPCB|NAPSA|TINAG|GGH|ADAM10|ENPEP|UQCRC2|AFG3L2|TINAGL1|UCHL1|CAPN2|RNPEP|TPP1|ANPEP|PDIA3|CNDP2|TPSAB1|CTSB|DPP3|DPP4|LONP1|XPNPEP2|PMPCA|PITRM1|DPEP1|PRCP|GGT5|GGT1|ACY1|CPVL|SCRN1|MME	4.03E-06	GO.0070011	0.41630433
884	52	GO Function	ion transmembrane transporter activity	6.9E-05	SLC25A11|SLC25A3|ATP6V1B1|ATP5B|ATP6V0A1|NNT|VDAC1|SLC27A2|RHCG|ATP6V1A|ATP6V1B2|SLC25A4|ATP6V0D2|ATP5O|ATP6V0D1|COX7A1|ATP5L|ATP5H|SLC23A1|LETM1|ATP5I|ATP6V0A4|AQP1|COX5A|SLC7A8|SLC5A2|CYB5A|SLC25A15|ANXA6|ATP5C1|MPC1|MT-CO1|MT-CO2|ATP1B1|ATP5F1|SFXN2|SLC25A5|VDAC2|CLIC4|CLIC1|SLC3A2|SLC25A6|ATP5A1|TF|PCYOX1|SLC25A12|SLC4A4|COX7C|VDAC3|ATP1A1|SLC25A10|COX4I1	4.06E-06	GO.0015075	0.41630433
5	5	GO Function	glyceraldehyde-3-phosphate dehydrogenase (NAD+) (non-phosphorylating) activity	7E-05	ALDH2|ALDH3A2|ALDH9A1|ALDH1B1|ALDH7A1	4.24E-06	GO.0043878	0.41524273
5	5	GO Function	isocitrate dehydrogenase activity	7E-05	IDH3G|IDH3A|IDH2|IDH3B|IDH1	4.24E-06	GO.0004448	0.41524273
5	5	GO Process	isocitrate metabolic process	7.5E-05	ACO2|IDH3G|IDH2|IDH3B|IDH1	4.24E-06	GO.0006102	0.41278437
92	19	GO Component	azurophil granule lumen	4.4E-09	MAPK1|CTSC|TTR|GGH|LYZ|CCT2|GLB1|TUBB4B|PRDX6|PTGES2|ANXA2|VAT1|GM2A|CYB5R3|ACTR2|TXNDC5|NAPRT|IST1|NPC2	3.62E-10	GO.0035578	0.83575355
192	19	GO Component	actin-based cell projection	7.6E-05	ATP6V1B1|VIL1|SLC9A3R1|ATP6V1A|IQGAP2|ATP6V1B2|ACTC1|DCXR|AOC3|MSN|MYO6|CLIC4|DPEP1|CNP|CDC42|RDX|PDZK1|ACTA2|ITGB3	1.13E-05	GO.0098858	0.41169066
66	11	KEGG Pathways	Epithelial cell signaling in Helicobacter pylori infection	7.7E-05	ATP6V1B1|ADAM10|ATP6V0A1|ATP6V1A|ATP6V1B2|ATP6V0D2|ATP6V0D1|ATP6V0A4|RAC1|CDC42|PLCG2	1.17E-05	hsa05120	0.41140737
30	6	KEGG Pathways	Pentose phosphate pathway	0.0024	PFKL|PFKM|ALDOB|PFKP|TKT|FBP1	0.0005	hsa00030	0.26197888
179	19	GO Process	ammonium ion metabolic process	7.9E-05	AKR7A2|DMGDH|BBOX1|CPT1A|BHMT|HNMT|HPRT1|CRAT|SHMT1|CHDH|TMLHE|ALDH9A1|SARDH|AGMAT|MAOB|AKR1C3|SMS|ALDH7A1|DDC	4.49E-06	GO.0097164	0.41040253
228	19	GO Component	contractile fiber	0.00056	VCL|ENO1|HSPB1|ACTN4|ACTC1|PLEC|PARVA|TPM4|KRT19|FLNA|JUP|ACTN1|MYH11|ILK|FKBP1A|ACTA2|CRYAB|ACTG1|TTN	9.84E-05	GO.0043292	0.3251812
11	6	GO Process	erythrose 4-phosphate/phosphoenolpyruvate family amino acid metabolic process	8.9E-05	GSTZ1|QDPR|HGD|HPD|FAH|PAH	5.09E-06	GO.1902221	0.4051587

11	6	GO Process	L-phenylalanine catabolic process	8.9E-05	GSTZ1|QDPR|HGD|HPD|FAH|PAH	5.09E-06	GO.0006559	0.4051587
11	6	GO Process	L-phenylalanine metabolic process	8.9E-05	GSTZ1|QDPR|HGD|HPD|FAH|PAH	5.09E-06	GO.0006558	0.4051587
27	8	GO Process	acetyl-CoA metabolic process	8.9E-05	PDHX|DLAT|PDHB|ACSS1|PIPOX|DLST|MPC1|PDHA1	5.13E-06	GO.0006084	0.4051587
60	11	GO Process	amine metabolic process	9E-05	DMGDH|BHMT|HNMT|AOC3|CHDH|SARDH|AGMAT|SMS|ALDH7A1|DDC|AOC1	5.23E-06	GO.0009308	0.40472076
1223	65	GO Function	transporter activity	9.8E-05	SLC25A11|SLC25A3|ATP6V1B1|ATP5B|LRP2|ATP6V0A1|NNT|VDAC1|SLC27A2|RHCG|ATP6V1A|ATP6V1B2|SLC25A4|ATP6V0D2|ATP5O|ATP6V0D1|ATP5J2|COX7A1|FABP1|ATP5L|ATP5H|SLC23A1|LETM1|ATP5I|ATP6V0A4|AQP1|COX5A|SLC7A8|SLC5A2|CYB5A|SLC25A15|SLC25A40|ANXA6|ATP5C1|GM2A|MPC1|MT-CO1|MT-CO2|ATP1B1|ATP5F1|SFXN2|ABCD3|RBP4|SCP2|SLC25A5|VDAC2|CLIC4|CLIC1|CUBN|SLC3A2|SLC25A6|TSPO|ATP5A1|TF|PCYOX1|SLC25A12|SLC4A4|PDZK1|TOMM40|COX7C|VDAC3|ATP1A1|SLC25A10|NPC2|COX4I1	6.13E-06	GO.0005215	0.40096611
1301	68	GO Function	protein dimerization activity	9.8E-05	OXCT1|LGALS1|MYH9|GSTZ1|CRYM|PNPO|DNPH1|ENO1|TTR|GOT2|VIL1|HSPB1|ACTN4|HRSP12|GSTM3|ADAM10|ERP29|ADD1|AMBP|PRDX5|SOD1|MUT|QDPR|GPD1L|CAPN2|ADH5|CRYL1|HPRT1|GPD1|GLB1|AOC3|GRHPR|SHMT1|P4HB|HSP90AA1|PRDX6|TPM4|PFKM|ALDH3A2|ANXA6|UGT1A9|DPP4|DARS2|S100A10|LHPP|FLNA|ABCD3|HOGA1|RBP4|FLOT1|CUBN|MAOB|NQO2|GLDC|JUP|ACTN1|FLOT2|QPRT|MGST1|ABAT|RDX|IDH1|TKT|AOC1|EPHX2|CRYAB|RAP1GAP|DNM1L	6.25E-06	GO.0046983	0.40070049
197	19	KEGG Pathways	Focal adhesion	9.9E-05	VCL|MAPK1|VTN|ACTN4|LAMA5|LAMC1|CAPN2|COL1A2|TLN1|PARVA|RAC1|COL4A2|FLNA|COL4A1|ACTN1|ILK|CDC42|ITGB3|ACTG1	1.57E-05	hsa04510	0.40048037
230	19	GO Component	cell cortex	0.00062	VCL|MYH9|PFN1|ENO1|DSTN|ACTN4|CAPN2|FABP1|SCIN|PLS1|GNAI1|ANXA2|MYH10|KRT19|FLNA|MYO6|ACTR2|SEPT7|RDX	0.00011	GO.0005938	0.32076083
23	7	KEGG Pathways	Proximal tubule bicarbonate reclamation	0.00011	PCK2|CA4|AQP1|PCK1|ATP1B1|SLC4A4|ATP1A1	1.75E-05	hsa04964	0.39586073
708	44	GO Process	ion homeostasis	0.00011	DCN|MAPK1|SKP1|LTF|ATP6V1B1|ATP5B|SLC9A3R1|ATP6V0A1|CALB1|RHCG|AFG3L2|SOD1|ATP6V1A|GPD1L|ATP6V0D2|ATP6V0D1|BDH2|CKB|HSP90B1|LETM1|ATP6V0A4|ACO1|PRKACA|ISCU|CYP4A11|SLC7A8|CALR|ANXA6|STOML2|TGM2|ATP1B1|GSTO1|SFXN2|GOT1|CLIC4|FHL1|UMOD|TSPO|TF|IMMT|SLC4A4|EPHX2|ATP1A1|PLCG2	6.4E-06	GO.0050801	0.39586073
1353	70	GO Process	response to endogenous stimulus	0.00011	OXCT1|MAPK1|CTSH|RAB35|ATP6V1B1|GOT2|VIL1|GGH|KHK|CD81|ATP6V0A1|SORD|SOD1|ATP6V1A|ATP6V1B2|QDPR|ATP6V0D2|ATP6V0D1|CAPN2|COL1A2|HSP90B1|GPD1|YWHAG|GLB1|ATP6V0A4|PRKACA|AQP1|CYC1|SHMT1|PCK1|CALR|HSPA5|FBN1|PKLR|GNAI1|CTSB|ANXA2|MSN|COL4A2|LONP1|OPA1|AHCYL1|GOT1|GNAS|ASS1|RAB14|COL4A1|ACTR2|MAOB|POSTN|AKR1C3|HADHA|CD9|FECH|DNM2|CORO1B|JUP|TSPO|GSTP1|RDX|ALAD|ARPC1B|IDH1|PNPT1|DDC|AOC1|ACSL1|CRYAB|ATP1A1|PHB	6.27E-06	GO.0009719	0.39586073
28	8	GO Process	aromatic amino acid family catabolic process	0.00011	GSTZ1|HNMT|QDPR|HGD|HPD|FTCD|FAH|PAH	6.44E-06	GO.0009074	0.39586073
6	5	GO Function	epoxide hydrolase activity	0.00012	LTA4H|AKR7A2|RNPEP|EPHX2|EPHX1	7.59E-06	GO.0004301	0.39208188
104	14	GO Process	long-chain fatty acid metabolic process	0.00012	LTA4H|CPT1A|SLC27A2|CYP4A11|ACAD9|ACAA1|GSTA1|PTGES2|GPX4|SCP2|AKR1C3|GSTP1|ACSL1|EPHX2	7.24E-06	GO.0001676	0.39208188
106	14	GO Function	unfolded protein binding	0.00013	TRAP1|CANX|APCS|AFG3L2|CCT5|HSPA9|CCT2|HSP90B1|PPIB|CALR|HSPA5|HSP90AA1|TUBB4B|CRYAB	8.82E-06	GO.0051082	0.38860566

51	10	GO Function	extracellular matrix binding	0.00013	DCN|LGALS1|VTN|LGALS3|BCAM|TINAGL1|BGN|ANXA2|TGFBI|ITGB3	8.33E-06	GO.0050840	0.38860566
51	10	GO Function	carbon-carbon lyase activity	0.00013	PCK2|BCKDHA|SHMT1|PCK1|GOT1|HOGA1|ALDOB|DDC|ECHDC1|ME3	8.33E-06	GO.0016830	0.38860566
381	29	GO Function	nucleoside binding	0.00013	RALA|PCK2|RAB35|TUBA4A|ACTN4|EHD2|TUBA1C|RAB1B|PCK1|RHOT2|TUFM|EHD3|RAB11B|TUBB4B|GNAI1|ANXA6|RAC1|PNP|OPA1|TGM2|GNAS|RAB14|AK3|DNM2|AK4|SEPT7|CDC42|GFM1|DNM1L	8.17E-06	GO.0001882	0.38860566
32	13	KEGG Pathways	Propanoate metabolism	3.3E-09	EHHADH|BCKDHA|MUT|ACSS1|BCKDHB|ECHS1|PCCA|SUCLA2|HADHA|ABAT|PCCB|ECHDC1|ALDH6A1	2.41E-10	hsa00640	0.84881166
1467	74	GO Process	establishment of protein localization	0.00013	MYH9|RAB35|EHHADH|ATP6V1B1|CANX|HSPB1|PMPCB|MTX2|ACTN4|ERP29|NAPA|LRP2|ATP6V0A1|SLC27A2|UQCRC2|TIMM44|ATP6V1A|ATP6V1B2|ATP6V0D2|ATP6V0D1|HSPA9|AMN|HSP90B1|YWHAG|ATP6V0A4|RAB1B|DAB2|CRAT|PIPOX|CALR|DHRS4|EHD3|AP2A2|RAB11B|ACAA1|HSP90AA1|RPS2|SAMM50|AGK|PNP|ATP1B1|AHCYL1|FLNA|MYO6|GNAS|SCP2|PMPCA|YWHAB|RAB14|CUBN|MPV17|AKR1C3|PITRM1|SLC25A6|AMACR|KRT18|DNM2|MVP|TSPO|RDX|TF|SSB|IDH1|SELENBP1|HK1|TOMM40|NDUFA13|EPHX2|IST1|PHB2|TIMM50|RBM8A|AP2B1|HAO2	7.76E-06	GO.0045184	0.38860566
6	5	GO Process	choline catabolic process	0.00013	DMGDH|BHMT|CHDH|SARDH|ALDH7A1	7.59E-06	GO.0042426	0.38860566
20	7	GO Process	alditol metabolic process	0.00013	SORD|GPD2|PCK1|GOT1|DAK|GK|GALK1	8.23E-06	GO.0019400	0.38860566
29	8	GO Process	energy coupled proton transmembrane transport, against electrochemical gradient	0.00013	ATP6V1B1|ATP6V0A1|ATP6V1A|ATP6V1B2|ATP6V0D2|ATP6V0D1|ATP6V0A4|MT-CO1	8.01E-06	GO.0015988	0.38860566
1416	72	GO Process	peptide transport	0.00013	MYH9|RAB35|EHHADH|ATP6V1B1|CANX|HSPB1|PMPCB|MTX2|ACTN4|ERP29|SLC9A3R1|NAPA|LRP2|ATP6V0A1|SLC27A2|UQCRC2|TIMM44|ATP6V1A|ATP6V1B2|ATP6V0D2|ATP6V0D1|HSPA9|AMN|HSP90B1|YWHAG|ATP6V0A4|RAB1B|DAB2|CRAT|PIPOX|CALR|DHRS4|EHD3|AP2A2|RAB11B|ACAA1|HSP90AA1|RPS2|SAMM50|AGK|PNP|ATP1B1|AHCYL1|MYO6|GNAS|SCP2|PMPCA|YWHAB|RAB14|CUBN|MPV17|AKR1C3|PITRM1|SLC25A6|AMACR|KRT18|DNM2|MVP|TSPO|TF|SSB|IDH1|SELENBP1|TOMM40|NDUFA13|EPHX2|IST1|PHB2|TIMM50|RBM8A|AP2B1|HAO2	8.06E-06	GO.0015833	0.38860566
1391	71	GO Process	protein transport	0.00013	MYH9|RAB35|EHHADH|ATP6V1B1|CANX|HSPB1|PMPCB|MTX2|ACTN4|ERP29|NAPA|LRP2|ATP6V0A1|SLC27A2|UQCRC2|TIMM44|ATP6V1A|ATP6V1B2|ATP6V0D2|ATP6V0D1|HSPA9|AMN|HSP90B1|YWHAG|ATP6V0A4|RAB1B|DAB2|CRAT|PIPOX|CALR|DHRS4|EHD3|AP2A2|RAB11B|ACAA1|HSP90AA1|RPS2|SAMM50|AGK|PNP|ATP1B1|AHCYL1|MYO6|GNAS|SCP2|PMPCA|YWHAB|RAB14|CUBN|MPV17|AKR1C3|PITRM1|SLC25A6|AMACR|KRT18|DNM2|MVP|TSPO|TF|SSB|IDH1|SELENBP1|TOMM40|NDUFA13|EPHX2|IST1|PHB2|TIMM50|RBM8A|AP2B1|HAO2	8.29E-06	GO.0015031	0.38860566
39	9	GO Process	aromatic amino acid family metabolic process	0.00013	GSTZ1|MTHFD1|HNMT|QDPR|HGD|HPD|FTCD|FAH|PAH	7.55E-06	GO.0009072	0.38860566

4714	186	GO Process	biosynthetic process	0.00013	DCN|NANS|MAPK1|GSTZ1|MTHFD1|PCK2|AHCY|OGDH|PNPO|SLC25A11|C1QBP|PDHX|LTA4H|GAPDH|DNPH1|AGXT2|LTF|SPR|ENO1|GDA|GOT2|BHMT2|GSTM3|CARS2|MRPL15|LRPPRC|ATP5B|CHCHD3|BBOX1|MECR|ATP6V0A1|VCAN|ALDH8A1|EIF4H|NDUFA9|LUM|SORD|SLC27A2|PFKL|SOD1|ALDH1L1|BHMT|MRPL49|DLAT|OXSM|ACAD8|QDPR|GPD1L|MRPL17|ATP5O|ATP5J2|BDH2|CRYL1|HPRT1|ATP5L|GPD1|ATP5H|DCXR|HINT1|MRPL39|ATP5I|MRPL13|ASL|PDHB|ATP6V0A4|GPD2|AQP1|GBAS|TSFM|ACSS1|CYC1|SHMT1|UGDH|PCK1|CHDH|TUFM|BCAT2|CNDP2|MDH2|BGN|ACSM2B|IDH2|MRPL12|HSP90AA1|TMLHE|GSTA1|PKLR|RPS2|SLC25A15|PPA2|PTGES2|PFKM|ALDH3A2|GPX4|ALDH9A1|AGK|STOML2|ATP5C1|PBLD|COL4A2|MRPL37|MPC1|CYB5R3|PNP|MRPL21|DARS2|IBA57|IARS2|MGST3|LHPP|HMGCS2|PHGDH|GSTO1|ATP5F1|FLNA|ABCD3|GOT1|HOGA1|ALDH18A1|RBP4|SCP2|CMPK1|AKR1A1|ASS1|RAB14|OGDHL|HSPG2|ALDOB|VARS|AGMAT|PSAT1|MAOB|SUCLA2|ESD|PDHA1|AKR1C3|DUT|PFKP|AMACR|FECH|SACM1L|BDH1|NME2|CNP|PC|AK4|QPRT|MGST1|TSPO|AMPD3|ABAT|GATM|MPST|HAGH|GGT5|GSTP1|ATP5A1|GGT1|SMS|ALAD|ALDH7A1|SLC25A12|TKT|NAPRT|GK|HK1|DDC|GPX1|FBP1|LARS2|GBE1|MRPS22|GFM1|SSBP1|GSTA2|ACSL1|MRPS30|EPHX2|PHB2|SLC25A10|PAH|ACSM2A|GALK1|SARS2|PHB|PLCG2	7.92E-06	GO.0009058	0.38860566
510	35	GO Process	endocytosis	0.00013	MAPK1|MYH9|VTN|CANX|TINAG|EHD2|CD81|LRP2|AMBP|TINAGL1|AMN|HSP90B1|DPYSL2|DAB2|CALR|AP2A2|HSP90AA1|RAC1|TGM2|MYO6|HSPG2|CUBN|ACTR2|TXNDC5|CD9|DNM2|CDC42|PDIA6|ARPC1B|AHSG|DNM1L|ITGB3|ACTG1|PLCG2|AP2B1	8.22E-06	GO.0006897	0.38860566
20	7	GO Process	methionine metabolic process	0.00013	MTHFD1|AHCY|BHMT2|BHMT|MSRA|AHCYL1|SMS	8.23E-06	GO.0006555	0.38860566
12	6	GO Process	mitochondrial electron transport, ubiquinol to cytochrome c	0.00013	UQCRC1|PMPCB|CYCS|UQCRH|CYC1|UQCR10	7.45E-06	GO.0006122	0.38860566
12	6	GO Process	ethanol oxidation	0.00013	ALDH2|ADH5|ALDH1A1|ADH1B|ACSS1|ALDH1B1	7.45E-06	GO.0006069	0.38860566
2370	107	GO Process	immune system process	0.00013	VCL|MAPK1|LGALS1|MYH9|MTHFD1|AHCY|PGRMC1|CTSH|C1QBP|VTN|CTSC|MLEC|LTA4H|GAPDH|RAB35|LTF|CTSD|TTR|CANX|LGALS3|APCS|TINAG|GGH|ADAM10|LYZ|PSMD11|COTL1|PRDX1|PSMD3|ATP6V0A1|ADD1|SLC27A2|PFKL|SOD1|ECSIT|TINAGL1|IQGAP2|HSPA9|COL1A2|PRDX3|HPRT1|CCT2|HSP90B1|ANPEP|PDIA3|GAA|GLB1|PRKACA|SLC7A8|CALR|AP2A2|ACAA1|HSP90AA1|TUBB4B|PRDX6|PTGES2|CTSB|ANXA2|VAT1|RAC1|STOML2|PIGR|GM2A|MSN|DPP4|CYB5R3|PNP|C4BPA|ATP1B1|ASS1|RAB14|SLC3A2|ACTR2|TXNDC5|DNM2|NME2|PRCP|JUP|NIT2|ACTN1|DAK|MVP|UMOD|MGST1|AMPD3|GSTP1|FKBP1A|CDC42|IFI30|ALAD|ARPC1B|IDH1|AHSG|NAPRT|NDRG1|AOC1|SERPINA1|MME|IST1|VIM|MGAM|METTL7A|NPC2|ITGB3|ACTG1|PLCG2|AP2B1	7.7E-06	GO.0002376	0.38860566
164	18	GO Component	tertiary granule	3.8E-05	CTSH|LTA4H|LTF|CTSD|LGALS3|GGH|ADAM10|LYZ|ATP6V0A1|GAA|AP2A2|RAC1|RAB14|PRCP|NIT2|IDH1|MGAM|METTL7A	5.23E-06	GO.0070820	0.44225082
122	15	GO Function	integrin binding	0.00014	VTN|ACTN4|LAMA5|ADAM10|CD81|TLN1|CALR|FBN1|P4HB|HSPG2|CD9|ACTN1|ILK|TGFBI|ITGB3	9.3E-06	GO.0005178	0.3853872
512	35	GO Process	protein complex oligomerization	0.00014	DECR1|CPT1A|PFKL|DPYS|COL1A2|HPRT1|DCXR|LETM1|GRHPR|SHMT1|DHRS4|EHD3|PFKM|ANXA2|ANXA6|STOML2|LONP1|OPA1|TGM2|S100A10|YWHAB|FLOT1|KCTD12|GPX3|JUP|QPRT|MGST1|ILK|SEPT7|ALAD|CRYZ|PNPT1|FBP1|CRYAB|DNM1L	8.9E-06	GO.0051259	0.3853872
21	7	GO Function	proton-transporting ATPase activity, rotational mechanism	0.00015	ATP5B|ATP6V0A1|ATP6V1A|ATP6V1B2|ATP6V0D2|ATP6V0D1|ATP6V0A4	1.07E-05	GO.0046961	0.38239087
21	7	GO Function	transaminase activity	0.00015	AGXT2|GOT2|AMT|BCAT2|GOT1|PSAT1|ABAT	1.07E-05	GO.0008483	0.38239087

40	13	KEGG Pathways	Tryptophan metabolism	2.8E-08	OGDH|EHHADH|ALDH2|ALDH3A2|ALDH9A1|ECHS1|OGDHL|ALDH1B1|MAOB|HADHA|ALDH7A1|DDC|AOC1	2.25E-09	hsa00380	0.75575202
25	7	KEGG Pathways	Collecting duct acid secretion	0.00016	ATP6V1B1|ATP6V0A1|ATP6V1A|ATP6V1B2|ATP6V0D2|ATP6V0D1|ATP6V0A4	2.75E-05	hsa04966	0.379588
73	11	KEGG Pathways	Thyroid hormone synthesis	0.00016	TTR|CANX|LRP2|HSP90B1|PRKACA|HSPA5|ATP1B1|GNAS|GPX3|GPX1|ATP1A1	2.71E-05	hsa04918	0.379588
2556	113	GO Process	cellular component biogenesis	0.00016	NDUFB4|VCL|DECR1|C1QBP|VTN|CTSC|SKP1|TUBA4A|VIL1|NDUFA2|ACTN4|NDUFA10|LAMA5|APCS|LAMC1|PSMD11|SLC9A3R1|NAPA|ATP6V0A1|ADD1|CPT1A|EIF4H|NDUFA9|PFKL|ECSIT|DPYS|NDUFB9|ACTC1|ATP6V0D1|HSPA9|COL1A2|HPRT1|NDUFB8|CCT2|HSP90B1|DCXR|LETM1|YWHAG|ATP6V0A4|PRKACA|RAB1B|ISCU|ACAD9|GRHPR|TLN1|SHMT1|CALR|NDUFV1|PLEC|DHRS4|EHD3|P4HB|NDUFA12|UQCR10|PARVA|HSP90AA1|PLS1|TUBB4B|RPS2|EPB41L3|SAMM50|PFKM|ANXA2|GPX4|ANXA6|RAC1|STOML2|MYH10|LONP1|OPA1|COA3|KRT19|TGM2|IBA57|S100A10|FLNA|YWHAB|ALDOB|FLOT1|KCTD12|ACTR2|CD9|GPX3|DNM2|CORO1B|JUP|ACTN1|FLOT2|QPRT|MGST1|MYH11|ILK|SEPT7|FKBP1A|CDC42|RDX|ALAD|NDUFS1|CRYZ|PNPT1|COX20|FBP1|BCS1L|SERPINA1|ATPAF2|TIMMDC1|NDUFA13|CRYAB|IST1|DNM1L|ACTG1|TTN|AP2B1	1.01E-05	GO.0044085	0.379588
1966	92	GO Process	protein localization	0.00016	VCL|MYH9|RAB35|SKP1|EHHADH|ATP6V1B1|CANX|HSPB1|PMPCB|MTX2|ACTN4|LAMA5|ERP29|SLC9A3R1|EHD2|NAPA|CD81|LRP2|ATP6V0A1|SLC27A2|UQCRC2|TIMM44|ATP6V1A|ATP6V1B2|ATP6V0D2|ATP6V0D1|SCIN|HSPA9|AMN|HSP90B1|YWHAG|ATP6V0A4|RAB1B|DAB2|CRAT|TLN1|PIPOX|CALR|HSPA5|FBN1|DHRS4|EHD3|AP2A2|RAB11B|ACAA1|HSP90AA1|RPS2|EPB41L3|SAMM50|ANXA2|AGK|PIGR|PNP|ATP1B1|S100A10|AHCYL1|FLNA|MYO6|GNAS|SCP2|PMPCA|YWHAB|RAB14|FLOT1|CUBN|MPV17|AKR1C3|PITRM1|SLC25A6|AMACR|KRT18|DNM2|CORO1B|JUP|FLOT2|MVP|TSPO|RDX|TF|SSB|IDH1|SELENBP1|HK1|TOMM40|NDUFA13|EPHX2|IST1|PHB2|TIMM50|RBM8A|AP2B1|HAO2	1.01E-05	GO.0008104	0.379588
311	18	GO Component	vacuolar membrane	0.024	ATP6V1B1|NAPA|ATP6V0A1|ATP6V0D2|ATP6V0D1|GAA|ATP6V0A4|DAB2|AP2A2|ANXA2|ANXA6|PIGR|MYO6|CUBN|PRCP|MGST1|PCYOX1|AP2B1	0.0069	GO.0005774	0.16197888
305	25	GO Process	response to antibiotic	0.00017	OXCT1|PPIF|GOT2|GGH|SOD1|ACTC1|FABP1|PRDX3|AQP1|AOC3|TUFM|HSPA5|HSP90AA1|GNAI1|OPA1|RBP4|ACTR2|MAOB|FECH|ABAT|GSTP1|ALAD|GPX1|AOC1|CRYAB	1.07E-05	GO.0046677	0.37695511
13	6	GO Process	carnitine metabolic process	0.00017	BBOX1|CPT1A|CRAT|SHMT1|TMLHE|ALDH9A1	1.06E-05	GO.0009437	0.37695511
21	7	GO Process	aspartate family amino acid catabolic process	0.00017	CRYM|GOT2|HRSP12|PIPOX|DLST|GOT1|ALDH7A1	1.07E-05	GO.0009068	0.37695511
13	6	GO Process	acetyl-CoA biosynthetic process	0.00017	PDHX|DLAT|PDHB|ACSS1|MPC1|PDHA1	1.06E-05	GO.0006085	0.37695511
49	17	GO Component	proton-transporting two-sector ATPase complex	4.7E-11	ATP6V1B1|ATP5B|ATP6V0A1|ATP6V1A|ATP6V1B2|ATP6V0D2|ATP5O|ATP6V0D1|ATP5J2|ATP5L|ATP5H|ATP5I|ATP6V0A4|ATP5C1|ATP5F1|USMG5|ATP5A1	3.19E-12	GO.0016469	1.03316141
1049	57	GO Function	transmembrane transporter activity	0.00018	SLC25A11|SLC25A3|ATP6V1B1|ATP5B|ATP6V0A1|NNT|VDAC1|SLC27A2|RHCG|ATP6V1A|ATP6V1B2|SLC25A4|ATP6V0D2|ATP5O|ATP6V0D1|ATP5J2|COX7A1|ATP5L|ATP5H|SLC23A1|LETM1|ATP5I|ATP6V0A4|AQP1|COX5A|SLC7A8|SLC5A2|CYB5A|SLC25A15|SLC25A40|ANXA6|ATP5C1|MPC1|MT-CO1|MT-CO2|ATP1B1|ATP5F1|SFXN2|ABCD3|RBP4|SLC25A5|VDAC2|CLIC4|CLIC1|SLC3A2|SLC25A6|ATP5A1|TF|PCYOX1|SLC25A12|SLC4A4|TOMM40|COX7C|VDAC3|ATP1A1|SLC25A10|COX4I1	0.000013	GO.0022857	0.37447275

413	30	GO Function	actin binding	0.00018	VCL|MYH9|PFN1|DSTN|VIL1|ACTN4|COTL1|ADD1|IQGAP2|SCIN|TLN1|PLEC|PARVA|PLS1|EPB41L3|TPM4|MSN|MYH10|FLNA|MYO6|ACTR2|CORO1B|ACTN1|SNTB1|MYH11|RDX|ARPC1B|TAGLN|TTN|PDLIM5	1.33E-05	GO.0003779	0.37447275
175	18	GO Process	carbohydrate derivative catabolic process	0.00019	DCN|AHCY|NAGLU|VCAN|LUM|ABHD10|DPYS|GPD1L|HPRT1|GPD1|HINT1|GLB1|BGN|GM2A|PNP|HSPG2|DUT|AMPD3	1.18E-05	GO.1901136	0.37212464
31	8	GO Process	peptide catabolic process	0.00019	CTSH|LTA4H|ENPEP|RNPEP|TPP1|ANPEP|GGT5|GGT1	1.21E-05	GO.0043171	0.37212464
31	8	GO Process	hydrogen peroxide metabolic process	0.00019	PRDX1|PRDX5|SOD1|PRDX3|PRDX6|MAOB|GPX3|GPX1	1.21E-05	GO.0042743	0.37212464
55	10	GO Function	transferase activity, transferring alkyl or aryl (other than methyl) groups	0.0002	NANS|GSTZ1|GSTM3|GSTA1|MGST3|GSTO1|MGST1|GSTP1|SMS|GSTA2	1.49E-05	GO.0016765	0.369897
110	14	GO Process	mitochondrial translation	0.0002	MRPL15|MRPL49|MRPL17|MRPL39|MRPL13|TSFM|MRPL12|MRPL37|MRPL21|DARS2|LARS2|MRPS22|GFM1|MRPS30	1.29E-05	GO.0032543	0.369897
832	48	GO Function	inorganic molecular entity transmembrane transporter activity	0.00021	SLC25A11|SLC25A3|ATP6V1B1|ATP5B|ATP6V0A1|NNT|VDAC1|SLC27A2|ATP6V1A|ATP6V1B2|ATP6V0D2|ATP5O|ATP6V0D1|COX7A1|ATP5L|ATP5H|SLC23A1|LETM1|ATP5I|ATP6V0A4|AQP1|COX5A|SLC7A8|SLC5A2|CYB5A|SLC25A15|ANXA6|ATP5C1|MPC1|MT-CO1|MT-CO2|ATP1B1|ATP5F1|RBP4|VDAC2|CLIC4|CLIC1|SLC3A2|ATP5A1|TF|PCYOX1|SLC25A12|SLC4A4|COX7C|VDAC3|ATP1A1|SLC25A10|COX4I1	1.56E-05	GO.0015318	0.36777807
94	17	GO Component	lysosomal lumen	1.6E-07	DCN|NAGLU|CTSD|VCAN|LUM|TPP1|GAA|GLB1|BGN|HSP90AA1|CTSB|GM2A|HSPG2|CUBN|TXNDC5|IFI30|NPC2	1.75E-08	GO.0043202	0.67931741
213	17	GO Component	basolateral plasma membrane	0.0018	ATP6V1B1|CD81|RHCG|CDH16|SLC23A1|AQP1|SLC7A8|ANXA2|GM2A|MSN|ATP1B1|FLOT1|FLOT2|UMOD|TF|SLC4A4|ATP1A1	0.00036	GO.0016323	0.27447275
216	17	GO Component	myofibril	0.002	VCL|ENO1|HSPB1|ACTN4|ACTC1|PLEC|PARVA|TPM4|KRT19|FLNA|JUP|ACTN1|ILK|FKBP1A|CRYAB|ACTG1|TTN	0.00042	GO.0030016	0.269897
180	18	GO Function	metallopeptidase activity	0.00022	UQCRC1|LTA4H|PEPD|PMPCB|ADAM10|ENPEP|UQCRC2|AFG3L2|RNPEP|ANPEP|CNDP2|DPP3|XPNPEP2|PMPCA|PITRM1|DPEP1|ACY1|MME	1.68E-05	GO.0008237	0.36575773
23	7	GO Function	proton-exporting ATPase activity, phosphorylative mechanism	0.00023	ATP5B|ATP6V0A1|ATP6V1A|ATP6V1B2|ATP6V0D2|ATP6V0D1|ATP6V0A4	1.75E-05	GO.0008553	0.36382722
23	7	GO Function	cytochrome-c oxidase activity	0.00023	COX7A1|COX5A|CYB5A|MT-CO1|MT-CO2|COX7C|COX4I1	1.75E-05	GO.0004129	0.36382722
252	17	GO Component	adherens junction	0.0081	VCL|MAPK1|MYH9|ADD1|REXO2|PLEC|PARVA|FLOT1|RSU1|JUP|ACTN1|FLOT2|ILK|RDX|NDRG1|ITGB3|ACTG1	0.002	GO.0005912	0.2091515
10	5	KEGG Pathways	Sulfur metabolism	0.00025	SQRDL|CYCS|SUOX|MPST|TST	4.48E-05	hsa00920	0.360206
1367	69	GO Process	cellular protein localization	0.00025	VCL|RAB35|SKP1|EHHADH|HSPB1|PMPCB|MTX2|LAMA5|ERP29|SLC9A3R1|EHD2|NAPA|CD81|SLC27A2|UQCRC2|TIMM44|SCIN|HSPA9|AMN|HSP90B1|YWHAG|CRAT|TLN1|PIPOX|CALR|HSPA5|DHRS4|EHD3|AP2A2|RAB11B|ACAA1|HSP90AA1|RPS2|EPB41L3|SAMM50|ANXA2|AGK|PIGR|ATP1B1|S100A10|AHCYL1|FLNA|MYO6|SCP2|PMPCA|YWHAB|FLOT1|MPV17|AKR1C3|PITRM1|SLC25A6|AMACR|KRT18|CORO1B|JUP|FLOT2|TSPO|RDX|SSB|IDH1|HK1|TOMM40|NDUFA13|EPHX2|PHB2|TIMM50|RBM8A|AP2B1|HAO2	1.59E-05	GO.0034613	0.360206

129	15	GO Process	platelet degranulation	0.00026	VCL|TUBA4A|ACTN4|F13A1|SOD1|TLN1|FLNA|APOOL|CD9|ACTN1|TF|AHSG|SERPINA1|ITGB3|TTN	0.000017	GO.0002576	0.35850267
80	16	GO Component	extracellular exosome	1.2E-07	NAGLU|CD81|SORD|BHMT|ANPEP|CA4|AQP1|SERPINA5|ANXA2|XPNPEP2|CLIC1|CUBN|CD9|ACY1|AOC1|ACTG1	1.33E-08	GO.0070062	0.69065783
819	47	GO Function	pyrophosphatase activity	0.00027	RALA|MYH9|RAB35|TUBA4A|ATP5B|ATP6V0A1|EIF4H|ATP6V1A|MUT|ATP6V1B2|ATP6V0D2|ATP5O|ACTC1|ATP6V0D1|TUBA1C|ATP6V0A4|RAB1B|RHOT2|TUFM|HSPA5|RAB11B|HSP90AA1|TUBB4B|GNAI1|PPA2|RAC1|ATP5C1|MYH10|LONP1|OPA1|ATP1B1|LHPP|MYO6|ABCD3|GNAS|RAB14|DUT|DNM2|MYH11|ATP5A1|CDC42|PCYOX1|GFM1|RAP1GAP|ATP1A1|MYL6|DNM1L	2.17E-05	GO.0016462	0.35686362
485	33	GO Process	cellular response to organonitrogen compound	0.00027	MAPK1|ATP6V1B1|ATP6V0A1|SOD1|ATP6V1A|ATP6V1B2|ATP6V0D2|ATP6V0D1|CAPN2|COL1A2|HSP90B1|GPD1|YWHAG|ATP6V0A4|PRKACA|AQP1|SHMT1|PCK1|HSPA5|FBN1|PKLR|OPA1|AHCYL1|GOT1|GNAS|ASS1|COL4A1|ACTR2|DNM2|JUP|GSTP1|DDC|AOC1	1.75E-05	GO.0071417	0.35686362
1462	72	GO Function	ATP binding	0.00028	MAPK1|RTCB|MYH9|MTHFD1|IDH3G|ATP6V1B1|TRAP1|CARS2|KHK|PEBP1|ATP5B|EHD2|SLC27A2|AFG3L2|PFKL|TIMM44|ATP6V1A|ATP6V1B2|CCT5|ACTC1|HSPA9|CKB|CCT2|HSP90B1|PRKACA|ACSS1|HSPA5|EHD3|ACSM2B|HSP90AA1|PKLR|PFKM|AGK|MYH10|LONP1|DARS2|IARS2|MYO6|ABCD3|ALDH18A1|CMPK1|ASS1|VARS|PCCA|ACTR2|SUCLA2|PFKP|AK3|NME2|PC|DAK|AK4|MYH11|ILK|ATP5A1|GK|ACSF2|HK1|ACTA2|CKMT2|CKMT1A|LARS2|BCS1L|PCCB|ACSL1|NDUFA13|ATP1A1|ACTG1|ACSM2A|GALK1|TTN|SARS2	2.24E-05	GO.0005524	0.3552842
33	8	GO Process	pteridine-containing compound metabolic process	0.00028	MTHFD1|SPR|GGH|ALDH1L1|QDPR|FTCD|PIPOX|SHMT1	1.79E-05	GO.0042558	0.3552842
57	10	GO Process	tetrapyrrole metabolic process	0.0003	AMBP|MUT|BDH2|AMN|IBA57|CUBN|SUCLA2|FECH|TSPO|ALAD	1.96E-05	GO.0033013	0.35228787
99	13	GO Process	icosanoid metabolic process	0.0003	LTA4H|CYP4A11|CES2|PTGES2|GPX4|MGST3|AKR1C3|DPEP1|GGT5|GGT1|PTGR1|GPX1|EPHX2	1.94E-05	GO.0006690	0.35228787
15	6	GO Process	fatty acid beta-oxidation using acyl-CoA dehydrogenase	0.00031	ACADS|ACAD11|IVD|ETFDH|ACADVL|ETFA	2.02E-05	GO.0033539	0.35086383
15	6	GO Process	mitochondrial electron transport, cytochrome c to oxygen	0.00031	CYCS|COX5A|MT-CO1|MT-CO2|COX7C|COX4I1	2.02E-05	GO.0006123	0.35086383
8	5	GO Process	acetyl-CoA biosynthetic process from pyruvate	0.00031	PDHX|DLAT|PDHB|MPC1|PDHA1	2.02E-05	GO.0006086	0.35086383
15	6	GO Process	fructose metabolic process	0.00031	KHK|SORD|ALDH1A1|ALDOB|DAK|FBP1	2.02E-05	GO.0006000	0.35086383
118	14	GO Function	ATPase-coupled transmembrane transporter activity	0.00032	ATP5B|ATP6V0A1|ATP6V1A|ATP6V1B2|ATP6V0D2|ATP5O|ATP6V0D1|ATP6V0A4|ATP5C1|ATP1B1|ABCD3|ATP5A1|PCYOX1|ATP1A1	2.63E-05	GO.0042626	0.349485
366	27	GO Function	GTP binding	0.00032	RALA|PCK2|RAB35|TUBA4A|EHD2|TUBA1C|RAB1B|PCK1|RHOT2|TUFM|EHD3|RAB11B|TUBB4B|GNAI1|ANXA6|RAC1|OPA1|TGM2|GNAS|RAB14|AK3|DNM2|AK4|SEPT7|CDC42|GFM1|DNM1L	2.79E-05	GO.0005525	0.349485

16	6	GO Function	acyl-CoA dehydrogenase activity	0.00032	ACADS|ACAD11|ACAD8|ACAD9|IVD|ACADVL	2.72E-05	GO.0003995	0.349485
145	15	KEGG Pathways	Phagosome	0.00032	ATP6V1B1|CANX|TUBA4A|ATP6V0A1|ATP6V1A|ATP6V1B2|ATP6V0D2|ATP6V0D1|TUBA1C|ATP6V0A4|CALR|TUBB4B|RAC1|ITGB3|ACTG1	5.87E-05	hsa04145	0.349485
71	11	GO Process	iron ion transport	0.00032	LTF|ATP6V1B1|ATP6V0A1|ATP6V1A|ATP6V1B2|ATP6V0D2|ATP6V0D1|ATP6V0A4|RAB11B|DNM2|TF	2.15E-05	GO.0006826	0.349485
82	16	GO Component	microvillus	1.6E-07	ATP6V1B1|VIL1|SLC9A3R1|ATP6V1A|IQGAP2|ATP6V1B2|DCXR|AOC3|MSN|MYO6|CLIC4|DPEP1|CNP|RDX|PDZK1|ITGB3	1.8E-08	GO.0005902	0.67878124
144	16	GO Component	collagen-containing extracellular matrix	1E-04	NID2|VTN|LAMA5|LAMC1|TINAG|LUM|COL1A2|COL14A1|FBN1|ANXA2|COL18A1|COL4A2|HSPG2|COL4A1|TGFBI|VWA1	1.51E-05	GO.0062023	0.40008695
169	17	GO Process	regulation of reactive oxygen species metabolic process	0.0004	AGXT2|TRAP1|NNT|SOD1|DDAH1|HSP90AA1|RAC1|MT-CO2|ASS1|MPV17|AKR1C3|DNM2|PRCP|TSPO|GSTP1|CRYAB|SIRT5	2.67E-05	GO.2000377	0.339794
4656	181	GO Process	organic substance biosynthetic process	0.00041	DCN|NANS|MAPK1|GSTZ1|MTHFD1|PCK2|AHCY|OGDH|PNPO|SLC25A11|C1QBP|PDHX|LTA4H|GAPDH|DNPH1|AGXT2|LTF|SPR|ENO1|GDA|GOT2|BHMT2|GSTM3|CARS2|MRPL15|LRPPRC|ATP5B|CHCHD3|BBOX1|MECR|ATP6V0A1|VCAN|ALDH8A1|EIF4H|NDUFA9|LUM|SORD|SLC27A2|PFKL|BHMT|MRPL49|DLAT|OXSM|ACAD8|QDPR|GPD1L|MRPL17|ATP5O|ATP5J2|BDH2|CRYL1|HPRT1|ATP5L|GPD1|ATP5H|DCXR|HINT1|MRPL39|ATP5I|MRPL13|ASL|PDHB|ATP6V0A4|GPD2|AQP1|GBAS|TSFM|ACSS1|CYC1|SHMT1|UGDH|PCK1|CHDH|TUFM|BCAT2|CNDP2|MDH2|BGN|ACSM2B|IDH2|MRPL12|HSP90AA1|TMLHE|GSTA1|PKLR|RPS2|SLC25A15|PPA2|PTGES2|PFKM|ALDH3A2|GPX4|ALDH9A1|AGK|STOML2|ATP5C1|COL4A2|MRPL37|MPC1|CYB5R3|PNP|MRPL21|DARS2|IBA57|IARS2|MGST3|LHPP|HMGCS2|PHGDH|GSTO1|ATP5F1|FLNA|ABCD3|GOT1|HOGA1|ALDH18A1|RBP4|SCP2|CMPK1|AKR1A1|ASS1|RAB14|OGDHL|HSPG2|ALDOB|VARS|AGMAT|PSAT1|SUCLA2|ESD|PDHA1|AKR1C3|DUT|PFKP|AMACR|FECH|SACM1L|BDH1|NME2|CNP|PC|AK4|QPRT|MGST1|TSPO|AMPD3|ABAT|GATM|HAGH|GGT5|GSTP1|ATP5A1|GGT1|SMS|ALAD|ALDH7A1|SLC25A12|TKT|NAPRT|GK|HK1|DDC|GPX1|FBP1|LARS2|GBE1|MRPS22|GFM1|SSBP1|GSTA2|ACSL1|MRPS30|EPHX2|PHB2|SLC25A10|PAH|ACSM2A|GALK1|SARS2|PHB|PLCG2	2.73E-05	GO.1901576	0.33872161
47	9	GO Process	regulation of substrate adhesion-dependent cell spreading	0.00041	C1QBP|ACTN4|CALR|RAC1|S100A10|FLNA|POSTN|DNM2|CDC42	2.78E-05	GO.1900024	0.33872161
47	9	GO Process	interleukin-12-mediated signaling pathway	0.00041	RALA|SOD1|HSPA9|P4HB|ANXA2|MSN|GSTO1|CDC42|GSTA2	2.78E-05	GO.0035722	0.33872161
167	16	KEGG Pathways	Tight junction	0.00042	MYH9|TUBA4A|ACTN4|SLC9A3R1|TUBA1C|PRKACA|RAC1|MSN|MYH10|ACTR2|ACTN1|MYH11|CDC42|RDX|MYL6|ACTG1	0.000078	hsa04530	0.33767507
103	13	GO Process	reactive oxygen species metabolic process	0.00042	SPR|PRDX1|NNT|PRDX5|SOD1|PRDX3|PRDX6|MAOB|GPX3|NDUFS1|GPX1|NDUFA13|EPHX2	2.83E-05	GO.0072593	0.33767507
854	48	GO Process	response to hormone	0.00043	OXCT1|CTSH|ATP6V1B1|GOT2|GGH|KHK|ATP6V0A1|SORD|ATP6V1A|ATP6V1B2|QDPR|ATP6V0D2|ATP6V0D1|YWHAG|GLB1|ATP6V0A4|PRKACA|AQP1|CYC1|PCK1|CALR|FBN1|PKLR|GNAI1|CTSB|ANXA2|MSN|LONP1|AHCYL1|GOT1|GNAS|ASS1|MAOB|POSTN|AKR1C3|HADHA|FECH|TSPO|GSTP1|RDX|ALAD|ARPC1B|IDH1|PNPT1|ACSL1|CRYAB|ATP1A1|PHB	2.92E-05	GO.0009725	0.33665315

185	16	GO Component	lamellipodium	0.0012	VIL1|IQGAP2|ACTC1|RAC1|MYH10|DPP4|FLOT1|ACTR2|DNM2|NME2|CORO1B|FLOT2|ILK|RDX|ACTA2|ITGB3	0.00023	GO.0030027	0.29208188
9	5	GO Process	nucleobase transport	0.00045	SLC25A4|SLC23A1|SLC25A5|SLC25A6|VDAC3	3.06E-05	GO.0015851	0.33467875
354	26	GO Function	active transmembrane transporter activity	0.00047	SLC25A11|SLC25A3|ATP5B|ATP6V0A1|ATP6V1A|ATP6V1B2|SLC25A4|ATP6V0D2|ATP5O|ATP6V0D1|SLC23A1|LETM1|ATP6V0A4|SLC7A8|SLC5A2|ATP5C1|ATP1B1|ABCD3|SLC25A5|SLC3A2|SLC25A6|ATP5A1|PCYOX1|SLC4A4|ATP1A1|SLC25A10	4.19E-05	GO.0022804	0.33279021
137	15	GO Process	mitochondrial gene expression	0.00047	MRPL15|MRPL49|MRPL17|MRPL39|MRPL13|TSFM|MRPL12|MRPL37|MRPL21|DARS2|PNPT1|LARS2|MRPS22|GFM1|MRPS30	3.23E-05	GO.0140053	0.33279021
89	12	GO Process	mitochondrial translational elongation	0.00047	MRPL15|MRPL49|MRPL17|MRPL39|MRPL13|TSFM|MRPL12|MRPL37|MRPL21|MRPS22|GFM1|MRPS30	3.19E-05	GO.0070125	0.33279021
53	15	GO Component	peroxisomal matrix	7.7E-09	CRYM|EHHADH|PRDX5|FABP1|GRHPR|CRAT|PIPOX|DHRS4|ACAA1|ABCD3|SCP2|AMACR|IDH1|EPHX2|HAO2	6.54E-10	GO.0005782	0.81163386
124	14	GO Function	ATPase activity, coupled to movement of substances	0.00048	ATP5B|ATP6V0A1|ATP6V1A|ATP6V1B2|ATP6V0D2|ATP5O|ATP6V0D1|ATP6V0A4|ATP5C1|ATP1B1|ABCD3|ATP5A1|PCYOX1|ATP1A1	4.33E-05	GO.0043492	0.33187588
26	7	GO Process	carbohydrate phosphorylation	0.00049	KHK|PFKL|PFKM|PFKP|DAK|HK1|GALK1	3.41E-05	GO.0046835	0.33098039
26	7	GO Process	cellular carbohydrate catabolic process	0.00049	SORD|ABHD10|GAA|GPD2|PFKM|GK|MGAM	3.41E-05	GO.0044275	0.33098039
105	13	GO Process	actin filament-based movement	0.00049	MYH9|VIL1|ACTN4|GPD1L|ACTC1|PARVA|TPM4|MYH10|MYO6|ATP1A1|VIM|MYL6|TTN	0.000034	GO.0030048	0.33098039
27	7	Reactome Pathways	ROS, RNS production in phagocytes	0.0005	ATP6V1B1|ATP6V0A1|ATP6V1A|ATP6V1B2|ATP6V0D2|ATP6V0D1|ATP6V0A4	4.19E-05	HSA-1222556	0.330103
67	15	GO Component	integral component of mitochondrial membrane	9.9E-08	CHCHD3|CPT1A|AFG3L2|SLC25A4|C19orf70|TMEM11|RHOT2|AGK|MPC1|COA3|APOOL|APOO|IMMT|TOMM40|ETFDH	1.02E-08	GO.0032592	0.70043648
643	39	GO Process	inorganic ion homeostasis	0.00052	MAPK1|SKP1|LTF|ATP6V1B1|ATP5B|SLC9A3R1|ATP6V0A1|CALB1|RHCG|AFG3L2|SOD1|ATP6V1A|ATP6V0D2|ATP6V0D1|BDH2|CKB|HSP90B1|LETM1|ATP6V0A4|ACO1|PRKACA|ISCU|CYP4A11|SLC7A8|CALR|ANXA6|STOML2|TGM2|ATP1B1|GSTO1|SFXN2|GOT1|CLIC4|TF|IMMT|SLC4A4|EPHX2|ATP1A1|PLCG2	3.59E-05	GO.0098771	0.32839967
17	6	GO Process	serine family amino acid biosynthetic process	0.00052	MTHFD1|AGXT2|SHMT1|PHGDH|PSAT1|GGT1	3.58E-05	GO.0009070	0.32839967
156	16	GO Process	protein localization to plasma membrane	0.00053	LAMA5|SLC9A3R1|EHD2|AMN|EHD3|EPB41L3|ANXA2|ATP1B1|S100A10|FLNA|SCP2|FLOT1|KRT18|JUP|FLOT2|RDX	3.69E-05	GO.0072659	0.32757241
62	10	GO Process	pigment metabolic process	0.00053	MTHFD1|AMBP|BDH2|HPRT1|SHMT1|UGT1A9|IBA57|FECH|TSPO|ALAD	3.74E-05	GO.0042440	0.32757241

4963	190	GO Process	organic cyclic compound metabolic process	0.00054	NDUFB4|UQCRC1|NANS|MAPK1|RTCB|GSTZ1|MTHFD1|AHCY|CRYM|OGDH|PNPO|C1QBP|PDHX|GAPDH|DNPH1|LTF|ATP6V1B1|SPR|ENO1|AKR7A2|GDA|GOT2|PMPCB|NDUFA2|NDUFA10|LGALS3|GRSF1|HRSP12|GSTM3|CARS2|GGH|LRPPRC|ATP5B|CHCHD3|LRP2|ATP6V0A1|NNT|SDHA|AMBP|PRDX5|REXO2|NDUFA9|SLC27A2|UQCRC2|PFKL|ATP6V1A|ALDH1L1|MUT|ATP6V1B2|ERLIN2|LACTB2|DPYS|NDUFB9|HNMT|DLAT|OXSM|ACAD8|QDPR|GPD1L|HGD|HPD|ATP5O|FTCD|ATP5J2|BDH2|HPRT1|AMN|NDUFB8|ATP5L|GPD1|PRODH2|ATP5H|SLC23A1|DCXR|HINT1|UGT2B7|ATP5I|PDHB|CYCS|ATP6V0A4|GPD2|DPYSL2|UQCRH|PRKACA|AQP1|GBAS|ACSS1|CYC1|PIPOX|COX5A|SHMT1|UGDH|UGT2B17|NDUFV1|DHRS4|MDH2|ACSM2B|NDUFA12|IDH2|UQCR10|ACAA1|MRPL12|HSP90AA1|DLST|PKLR|RPS2|PPA2|PFKM|STOML2|ATP5C1|COL4A2|LONP1|MPC1|CYB5R3|MT-CO1|PNP|OPA1|MT-CO2|DARS2|IBA57|IARS2|ATP1B1|LHPP|HMGCS2|ATP5F1|AHCYL1|FLNA|GNAS|ALDH18A1|SCP2|CMPK1|OGDHL|ALDOB|ALDH4A1|VARS|PCCA|PSAT1|CUBN|MAOB|SUCLA2|ACOT9|PDHA1|GLRX|AKR1C3|DUT|PFKP|AK3|AMACR|FECH|NME2|CNP|PC|AK4|QPRT|TSPO|AMPD3|ATP5A1|FAH|ALAD|SSB|IDH1|TKT|NDUFS1|PNPT1|NAPRT|ACSF2|HK1|DDC|GPX1|LARS2|MME|PCCB|SSBP1|ABHD14B|ACSL1|COX7C|EPHX2|PHB2|PAH|ALDH6A1|NPC2|COX4I1|ACSM2A|RBM8A|GALK1|SARS2|PHB|EPHX1|FMO1|GLYAT	3.81E-05	GO.1901360	0.32676062
164	15	GO Component	ruffle	0.0011	MYH9|VIL1|SLC9A3R1|ATP6V1B2|TLN1|HSP90AA1|ANXA2|RAC1|MYO6|GNAS|DNM2|NME2|ACTN1|RDX|ITGB3	0.00021	GO.0001726	0.29586073
778	44	GO Function	nucleoside-triphosphatase activity	0.0006	RALA|MYH9|RAB35|TUBA4A|ATP5B|ATP6V0A1|EIF4H|ATP6V1A|MUT|ATP6V1B2|ATP6V0D2|ATP5O|ACTC1|ATP6V0D1|TUBA1C|ATP6V0A4|RAB1B|RHOT2|TUFM|HSPA5|RAB11B|HSP90AA1|TUBB4B|GNAI1|RAC1|ATP5C1|MYH10|LONP1|OPA1|ATP1B1|MYO6|ABCD3|GNAS|RAB14|DNM2|MYH11|ATP5A1|CDC42|PCYOX1|GFM1|RAP1GAP|ATP1A1|MYL6|DNM1L	5.51E-05	GO.0017111	0.32218487
4	4	GO Process	adenine transport	0.0006	SLC25A4|SLC25A5|SLC25A6|VDAC3	4.26E-05	GO.0015853	0.32218487
50	9	GO Process	cellular biogenic amine metabolic process	0.0006	DMGDH|BHMT|HNMT|CHDH|SARDH|AGMAT|SMS|ALDH7A1|DDC	4.28E-05	GO.0006576	0.32218487
38	8	GO Process	nucleobase metabolic process	0.00061	MAPK1|MTHFD1|GDA|DPYS|HPRT1|SHMT1|CMPK1|ALDH6A1	4.31E-05	GO.0009112	0.32146702
195	14	GO Component	sarcomere	0.0113	ENO1|HSPB1|ACTN4|ACTC1|PARVA|TPM4|KRT19|FLNA|JUP|ACTN1|ILK|FKBP1A|CRYAB|TTN	0.0029	GO.0030017	0.19469216
10	5	GO Process	mitochondrial protein processing	0.00063	PMPCB|UQCRC2|AFG3L2|STOML2|PMPCA	4.48E-05	GO.0034982	0.32006595
200	18	GO Function	cell adhesion molecule binding	0.00065	VTN|ACTN4|LAMA5|ADAM10|CD81|TLN1|CALR|FBN1|P4HB|MSN|HSPG2|CD9|JUP|ACTN1|ILK|NDRG1|TGFBI|ITGB3	6.03E-05	GO.0050839	0.31870866
18	6	GO Process	2-oxoglutarate metabolic process	0.00065	OGDH|GOT2|IDH2|DLST|GOT1|IDH1	4.66E-05	GO.0006103	0.31870866
93	12	GO Process	diterpenoid metabolic process	0.00066	TTR|LRP2|ALDH8A1|ADH5|ALDH1A1|DHRS4|ALDH3A2|UGT1A9|RBP4|HSPG2|AKR1C3|RDH13	4.71E-05	GO.0016101	0.31804561
321	24	GO Function	amide binding	0.00067	PPIF|LTA4H|GSTM3|ENPEP|FTCD|RNPEP|TPP1|PPIB|ANPEP|SLC7A8|CALR|PTGES2|SCP2|PCCA|NQO2|HADHA|PC|MGST1|GSTP1|MME|CRYAB|PHB2|ALDH6A1|AP2B1	6.32E-05	GO.0033218	0.31739252
53	9	GO Function	pyridoxal phosphate binding	0.00067	PNPO|AGXT2|GOT2|SHMT1|MARC2|GOT1|GLDC|ABAT|DDC	0.000064	GO.0030170	0.31739252
147	15	GO Function	isomerase activity	0.0007	GSTZ1|PPIF|EHHADH|MUT|PPIB|PDIA3|ECI1|P4HB|PTGES2|PBLD|TXNDC5|AMACR|FKBP1A|PDIA6|ITGB3	6.77E-05	GO.0016853	0.3154902

25	4	KEGG Pathways	Fatty acid elongation	0.0285	MECR|HADHB|ECHS1|HADHA	0.009	hsa00062	0.15451551
30	7	GO Function	acid-thiol ligase activity	0.00076	SLC27A2|ACSS1|ACSM2B|SUCLA2|ACSF2|ACSL1|ACSM2A	7.44E-05	GO.0016878	0.31191864
20	6	GO Function	C-acyltransferase activity	0.00077	HADHB|ACSM2B|ACAA1|SCP2|HADHA|ACSM2A	0.000076	GO.0016408	0.31135093
162	16	GO Process	cellular response to metal ion	0.00078	MAPK1|PPIF|ADD1|SOD1|AQP1|CALR|HSPA5|CLIC4|MT1M|AKR1C3|DPEP1|TSPO|TF|ALAD|FBP1|AOC1	0.000056	GO.0071248	0.31079054
43	13	GO Component	mitochondrial nucleoid	4.8E-08	GRSF1|LRPPRC|ATP5B|VDAC1|HSPA9|TUFM|HADHB|LONP1|SLC25A5|VDAC2|HADHA|SSBP1|ACADVL	4.67E-09	GO.0042645	0.73214816
47	8	KEGG Pathways	Endocrine and other factor-regulated calcium reabsorption	0.00083	CALB1|PRKACA|AP2A2|ATP1B1|GNAS|DNM2|ATP1A1|AP2B1	0.00016	hsa04961	0.30809219
200	18	GO Process	actin filament organization	0.00084	DSTN|VIL1|ACTN4|ADD1|IQGAP2|ACTC1|SCIN|HSP90B1|PLS1|TPM4|RAC1|FLNA|ACTR2|CORO1B|ACTN1|CDC42|ARPC1B|TTN	6.03E-05	GO.0007015	0.30757207
48	11	KEGG Pathways	Fatty acid metabolism	6.3E-06	EHHADH|ACADS|MECR|CPT1A|OXSM|HADHB|ACAA1|ECHS1|HADHA|ACSL1|ACADVL	7.79E-07	hsa01212	0.52006595
29	7	GO Process	leukotriene metabolic process	0.00085	LTA4H|CYP4A11|MGST3|DPEP1|GGT5|GGT1|PTGR1	6.18E-05	GO.0006691	0.30705811
11	5	GO Process	L-ascorbic acid metabolic process	0.00088	SLC23A1|CYB5A|CYB5R3|GSTO1|AKR1A1	6.37E-05	GO.0019852	0.30555173
11	5	GO Process	oxaloacetate metabolic process	0.00088	GOT2|PCK1|MDH2|GOT1|NIT2	6.37E-05	GO.0006107	0.30555173
386	27	GO Process	peptide biosynthetic process	0.0009	CARS2|MRPL15|EIF4H|MRPL49|MRPL17|BDH2|MRPL39|MRPL13|TSFM|TUFM|CNDP2|MRPL12|RPS2|PPA2|MRPL37|MRPL21|DARS2|IARS2|VARS|HAGH|GGT5|GGT1|LARS2|MRPS22|GFM1|MRPS30|SARS2	6.53E-05	GO.0043043	0.30457575
568	35	GO Process	cellular response to nitrogen compound	0.00092	MAPK1|ATP6V1B1|ATP6V0A1|SOD1|ATP6V1A|ATP6V1B2|ATP6V0D2|ATP6V0D1|CAPN2|COL1A2|HSP90B1|GPD1|YWHAG|ATP6V0A4|PRKACA|AQP1|SHMT1|PCK1|HSPA5|FBN1|PKLR|OPA1|AHCYL1|GOT1|GNAS|ASS1|COL4A1|FLOT1|ACTR2|DNM2|DPEP1|JUP|GSTP1|DDC|AOC1	0.000067	GO.1901699	0.30362122
21	6	GO Function	carbohydrate kinase activity	0.00096	KHK|PFKL|PFKM|PFKP|HK1|GALK1	9.54E-05	GO.0019200	0.30177288
21	6	GO Function	oxidoreductase activity, acting on the CH-NH2 group of donors	0.00096	CRYM|PNPO|AOC3|MAOB|GLDC|AOC1	9.54E-05	GO.0016638	0.30177288
222	19	GO Process	cellular response to oxidative stress	0.00097	MAPK1|PPIF|PRDX1|PRDX5|SOD1|FABP1|PRDX3|CYCS|AQP1|LONP1|MPV17|AKR1C3|GPX3|DNM2|DPEP1|MGST1|TXNRD2|PNPT1|GPX1	0.000071	GO.0034599	0.30132283
91	13	GO Component	basement membrane	6E-05	NID2|VTN|LAMA5|LAMC1|TINAG|FBN1|ANXA2|COL18A1|COL4A2|HSPG2|COL4A1|TGFBI|VWA1	8.61E-06	GO.0005604	0.4225483
159	13	GO Component	specific granule	0.0059	VCL|PGRMC1|MLEC|LTF|CTSD|GGH|ADAM10|LYZ|SLC27A2|ACAA1|JUP|NIT2|AOC1	0.0014	GO.0042581	0.2229148
185	17	GO Process	cellular response to inorganic substance	0.001	MAPK1|PPIF|ADD1|SOD1|AQP1|CALR|HSPA5|CLIC4|MT1M|AKR1C3|DNM2|DPEP1|TSPO|TF|ALAD|FBP1|AOC1	7.58E-05	GO.0071241	0.3

5	4	GO Process	fructose catabolic process to hydroxyacetone phosphate and glyceraldehyde-3-phosphate	0.001	KHK|ALDH1A1|ALDOB|DAK	7.49E-05	GO.0061624	0.3
68	10	GO Process	regulation of mitochondrial membrane potential	0.001	DCN|NNT|SOD1|PRDX3|PPA2|STOML2|GOT1|CLIC1|TSPO|NDUFS1	7.52E-05	GO.0051881	0.3
5	4	GO Process	carnitine biosynthetic process	0.001	BBOX1|SHMT1|TMLHE|ALDH9A1	7.49E-05	GO.0045329	0.3
20	6	GO Process	hydrogen peroxide catabolic process	0.001	PRDX1|PRDX5|PRDX3|PRDX6|GPX3|GPX1	0.000076	GO.0042744	0.3
5	4	GO Process	glucuronate catabolic process to xylulose 5-phosphate	0.001	SORD|CRYL1|DCXR|AKR1A1	7.49E-05	GO.0019640	0.3
5	4	GO Process	response to aluminum ion	0.001	QDPR|LONP1|MAOB|ALAD	7.49E-05	GO.0010044	0.3
30	7	GO Process	NADP metabolic process	0.001	NNT|PRDX5|DCXR|IDH2|IDH1|TKT|FMO1	7.44E-05	GO.0006739	0.3
5	4	GO Process	tyrosine catabolic process	0.001	GSTZ1|HGD|HPD|FAH	7.49E-05	GO.0006572	0.3
30	7	GO Process	glutamate metabolic process	0.001	GOT2|FTCD|PRODH2|GOT1|ALDH18A1|ALDH4A1|GGT1	7.44E-05	GO.0006536	0.3
201	13	GO Component	ribosome	0.03	CANX|MRPL15|MRPL49|MRPL17|MRPL39|MRPL13|MRPL12|RPS2|MRPL37|MRPL21|PNPT1|MRPS22|MRPS30	0.009	GO.0005840	0.15228787
33	12	GO Component	cytochrome complex	3.5E-08	UQCRC1|PMPCB|UQCRC2|UQCRH|CYC1|COX5A|UQCR10|MT-CO1|MT-CO2|BCS1L|COX7C|COX4I1	3.31E-09	GO.0070069	0.74571746
94	11	KEGG Pathways	Amoebiasis	0.0011	VCL|HSPB1|ACTN4|LAMA5|LAMC1|COL1A2|PRKACA|COL4A2|GNAS|COL4A1|ACTN1	0.00021	hsa05146	0.29586073
150	15	GO Process	maintenance of location	0.0011	SKP1|SCIN|HSP90B1|GAA|TLN1|CALR|HSPA5|FBN1|EPB41L3|GM2A|FLNA|YWHAB|JUP|TSPO|HK1	8.34E-05	GO.0051235	0.29586073
69	10	GO Process	cellular ketone metabolic process	0.0011	OXCT1|AKR7A2|GOT2|NDUFA9|GPD1|GPD2|DHRS4|SCP2|AKR1C3|HAGH	8.39E-05	GO.0042180	0.29586073
116	13	GO Process	translational elongation	0.0011	MRPL15|MRPL49|MRPL17|MRPL39|MRPL13|TSFM|TUFM|MRPL12|MRPL37|MRPL21|MRPS22|GFM1|MRPS30	8.64E-05	GO.0006414	0.29586073
79	12	GO Component	mitochondrial intermembrane space	7.5E-05	TRAP1|REXO2|SOD1|CYCS|AGK|STOML2|OPA1|SUOX|GATM|NDUFS1|PNPT1|SIRT5	1.09E-05	GO.0005758	0.41260984
113	12	GO Component	endoplasmic reticulum-Golgi intermediate compartment	0.0013	CTSC|NAT8|FTCD|ANPEP|CA4|RAB1B|CALR|HSPA5|P4HB|PDIA6|SERPINA1|IST1	0.00025	GO.0005793	0.28860566
6	4	GO Function	porin activity	0.0012	VDAC1|VDAC2|TOMM40|VDAC3	0.00012	GO.0015288	0.29208188
13	5	GO Function	peptide disulfide oxidoreductase activity	0.0012	PDIA3|P4HB|GSTO1|GLRX|PDIA6	0.00012	GO.0015037	0.29208188

73	10	GO Function	extracellular matrix structural constituent	0.0012	LAMC1|VCAN|LUM|TINAGL1|COL1A2|COL14A1|FBN1|BGN|COL4A2|COL4A1	0.00013	GO.0005201	0.29208188
873	47	GO Process	response to organic cyclic compound	0.0012	MAPK1|LGALS1|GOT2|CPT1A|LUM|SOD1|HSP90B1|GPD1|GLB1|PRKACA|AQP1|SHMT1|PCK1|CALR|HSPA5|PKLR|GNAI1|MSN|OPA1|ABCD3|GOT1|GNAS|ASS1|FLOT1|SLC3A2|ACTR2|MAOB|POSTN|AKR1C3|GLDC|FECH|DNM2|JUP|TSPO|ABAT|GSTP1|ALAD|ARPC1B|IDH1|PNPT1|AOC1|ACSL1|CRYAB|ATP1A1|DNM1L|PHB|EPHX1	9.47E-05	GO.0014070	0.29208188
122	12	GO Component	phagocytic vesicle	0.0023	LTF|ATP6V0A1|ATP6V0D2|ATP6V0D1|PDIA3|ATP6V0A4|CALR|RAB11B|RAB14|DNM2|VIM|ACTG1	0.00048	GO.0045335	0.26382722
629	37	GO Process	cation homeostasis	0.0013	MAPK1|SKP1|LTF|ATP6V1B1|ATP5B|ATP6V0A1|CALB1|RHCG|AFG3L2|SOD1|ATP6V1A|ATP6V0D2|ATP6V0D1|BDH2|HSP90B1|LETM1|ATP6V0A4|ACO1|PRKACA|ISCU|CYP4A11|SLC7A8|CALR|ANXA6|STOML2|TGM2|ATP1B1|GSTO1|SFXN2|GOT1|CLIC4|TF|IMMT|SLC4A4|EPHX2|ATP1A1|PLCG2	0.0001	GO.0055080	0.28860566
135	14	GO Process	cell junction assembly	0.0013	VCL|ACTN4|LAMA5|LAMC1|TLN1|PLEC|EPB41L3|FLNA|CD9|JUP|ACTN1|ILK|CDC42|ACTG1	0.0001	GO.0034329	0.28860566
209	18	GO Process	receptor-mediated endocytosis	0.0013	VTN|CANX|TINAG|CD81|LRP2|AMBP|TINAGL1|AMN|HSP90B1|DAB2|CALR|AP2A2|HSP90AA1|HSPG2|CUBN|CD9|DNM2|AP2B1	0.0001	GO.0006898	0.28860566
122	12	GO Component	sarcolemma	0.0023	VCL|GOT2|AQP1|PLEC|BGN|ANXA2|KRT19|ATP1B1|FLOT1|SNTB1|RDX|ATP1A1	0.00048	GO.0042383	0.26382722
165	12	GO Component	midbody	0.0182	RALA|HSP90B1|HSPA5|GNAI1|ANXA2|MYH10|CLIC4|DNM2|SEPT7|CDC42|RDX|IST1	0.0051	GO.0030496	0.17399286
34	7	GO Function	carboxy-lyase activity	0.0014	PCK2|BCKDHA|PCK1|GOT1|DDC|ECHDC1|ME3	0.00015	GO.0016831	0.2853872
106	12	GO Function	structural constituent of cytoskeleton	0.0014	TUBA4A|TUBA1C|TLN1|PLS1|TUBB4B|EPB41L3|MSN|KRT19|ACTR2|ARPC1B|VIM|ACTG1	0.00015	GO.0005200	0.2853872
32	7	GO Process	positive regulation of substrate adhesion-dependent cell spreading	0.0014	C1QBP|CALR|RAC1|S100A10|FLNA|DNM2|CDC42	0.00011	GO.1900026	0.2853872
119	13	GO Process	monovalent inorganic cation homeostasis	0.0014	MAPK1|ATP6V1B1|ATP5B|ATP6V0A1|RHCG|ATP6V0D2|ATP6V0D1|ATP6V0A4|CYP4A11|ATP1B1|CLIC4|SLC4A4|ATP1A1	0.00011	GO.0055067	0.2853872
44	8	GO Process	mitochondrial electron transport, NADH to ubiquinone	0.0014	NDUFB4|NDUFA2|NDUFA10|NDUFA9|NDUFB9|NDUFB8|NDUFV1|NDUFS1	0.00011	GO.0006120	0.2853872
36	11	GO Component	integral component of mitochondrial inner membrane	5.7E-07	CHCHD3|AFG3L2|C19orf70|TMEM11|AGK|MPC1|COA3|APOOL|APOO|IMMT|ETFDH	6.62E-08	GO.0031305	0.62456517
47	8	GO Function	intramolecular oxidoreductase activity	0.0015	EHHADH|PDIA3|ECI1|P4HB|PTGES2|TXNDC5|PDIA6|ITGB3	0.00016	GO.0016860	0.28239087
133	13	KEGG Pathways	Fluid shear stress and atherosclerosis	0.0015	GSTM3|HSP90B1|HSP90AA1|GSTA1|RAC1|MGST3|GSTO1|ASS1|MGST1|GSTP1|GSTA2|ITGB3|ACTG1	0.0003	hsa05418	0.28239087
6	4	GO Process	mitochondrial acetyl-CoA biosynthetic process from pyruvate	0.0015	PDHX|PDHB|MPC1|PDHA1	0.00012	GO.0061732	0.28239087
13	5	GO Process	homocysteine metabolic process	0.0015	MTHFD1|AHCY|MUT|DPEP1|MPST	0.00012	GO.0050667	0.28239087

87	11	GO Process	retinoid metabolic process	0.0015	TTR|LRP2|ALDH8A1|ADH5|ALDH1A1|DHRS4|UGT1A9|RBP4|HSPG2|AKR1C3|RDH13	0.00011	GO.0001523	0.28239087
57	11	GO Component	actin filament bundle	2.6E-05	MYH9|VIL1|ACTN4|PLS1|TPM4|MYH10|CORO1B|ACTN1|ILK|SEPT7|CRYAB	3.38E-06	GO.0032432	0.459176
63	11	GO Component	clathrin-coated pit	5.5E-05	RAB35|LRP2|AMN|DAB2|AP2A2|MYO6|CUBN|DNM2|TF|DNM1L|AP2B1	7.89E-06	GO.0005905	0.42580609
134	11	GO Component	focal adhesion	0.012	VCL|MAPK1|MYH9|REXO2|PLEC|PARVA|RSU1|ACTN1|ILK|ITGB3|ACTG1	0.0031	GO.0005925	0.19208188
62	9	GO Function	iron-sulfur cluster binding	0.0018	ACO2|ACO1|ISCU|NDUFV1|CISD1|FECH|ABAT|NDUFS1|ETFDH	0.00019	GO.0051536	0.27447275
4170	160	GO Function	cation binding	0.0018	UQCRC1|RTCB|ACO2|NID2|PCK2|IDH3G|F9|OGDH|LTA4H|LTF|ENO1|GDA|PEPD|CANX|VIL1|PMPCB|ACTN4|HRSP12|APCS|BHMT2|CARS2|ADAM10|BBOX1|EHD2|LRP2|F13A1|VCAN|ENPEP|CALB1|SORD|UQCRC2|AFG3L2|PFKL|BCKDHA|SOD1|BHMT|MUT|LACTB2|DPYS|HGD|DDAH1|HPD|CAPN2|RNPEP|ADH5|SCIN|COL1A2|HPRT1|TPP1|IDH3A|CDH16|HSP90B1|ANPEP|CA4|LETM1|ADH1B|CYCS|GPD2|ACO1|PRKACA|ISCU|CSRP2|CYP4A11|AOC3|ACSS1|CYC1|COX5A|SHMT1|PCK1|CALR|RHOT2|NDUFV1|HSPA5|FBN1|CNDP2|EHD3|ACSM2B|C11orf54|IDH2|SERPINA5|TMLHE|PLS1|PKLR|CYB5A|GNAI1|PPA2|TPM4|PFKM|ANXA2|ANXA6|COL18A1|VAT1|DPP3|CYB5R3|MT-CO1|OPA1|MT-CO2|TGM2|MARC2|CSRP1|S100A10|LHPP|RTN4IP1|GNAS|XPNPEP2|PMPCA|CISD1|OGDHL|HSPG2|ASPN|AGMAT|PCCA|CUBN|SUCLA2|POSTN|MT1M|HBD|NQO2|IDH3B|DUT|PITRM1|PFKP|GLDC|FECH|DPEP1|NME2|PC|SUOX|FHL1|ACTN1|DAK|UMOD|PDP1|AMPD3|ABAT|HAGH|ACY1|FAH|TF|ALAD|SLC25A12|IDH1|TKT|NDUFS1|CRYZ|FBP1|GBE1|AOC1|MME|ETFDH|EPHX2|CRYAB|ME3|ATP1A1|MYL6|PAH|ACSM2A|TTN|SIRT5|PDLIM5	0.00019	GO.0043169	0.27447275
665	38	GO Process	cellular chemical homeostasis	0.0018	MAPK1|LGALS1|SKP1|LTF|ATP5B|SLC9A3R1|ATP6V0A1|CALB1|RHCG|AFG3L2|SOD1|ATP6V1A|ATP6V0D2|ATP6V0D1|CKB|HSP90B1|LETM1|ATP6V0A4|ACO1|PRKACA|ISCU|CALR|RAB11B|ANXA6|STOML2|OPA1|TGM2|ATP1B1|GSTO1|GOT1|CLIC4|TF|IMMT|SLC4A4|HK1|EPHX2|ATP1A1|PLCG2	0.00015	GO.0055082	0.27447275
362	25	GO Process	response to peptide hormone	0.0018	ATP6V1B1|GOT2|GGH|KHK|ATP6V0A1|ATP6V1A|ATP6V1B2|QDPR|ATP6V0D2|ATP6V0D1|YWHAG|ATP6V0A4|PRKACA|CYC1|PCK1|FBN1|PKLR|GNAI1|AHCYL1|GOT1|GNAS|ASS1|HADHA|GSTP1|PNPT1	0.00015	GO.0043434	0.27447275
23	6	GO Process	cellular biogenic amine catabolic process	0.0018	DMGDH|BHMT|HNMT|CHDH|SARDH|ALDH7A1	0.00015	GO.0042402	0.27447275
90	11	GO Process	unsaturated fatty acid metabolic process	0.0019	CYP4A11|CES2|ACAA1|GSTA1|PTGES2|SCP2|AKR1C3|GSTP1|PTGR1|ACSL1|EPHX2	0.00015	GO.0033559	0.27212464
123	13	GO Process	isoprenoid metabolic process	0.0019	TTR|LRP2|ALDH8A1|ADH5|ALDH1A1|DHRS4|ALDH3A2|UGT1A9|HMGCS2|RBP4|HSPG2|AKR1C3|RDH13	0.00015	GO.0006720	0.27212464
136	11	GO Component	I band	0.013	HSPB1|ACTN4|ACTC1|PARVA|KRT19|FLNA|JUP|ACTN1|FKBP1A|CRYAB|TTN	0.0034	GO.0031674	0.18860566
137	11	GO Component	cell-substrate adherens junction	0.0136	VCL|MAPK1|MYH9|REXO2|PLEC|PARVA|RSU1|ACTN1|ILK|ITGB3|ACTG1	0.0036	GO.0005924	0.18664611
70	9	KEGG Pathways	Platinum drug resistance	0.002	MAPK1|GSTM3|CYCS|GSTA1|MGST3|GSTO1|MGST1|GSTP1|GSTA2	0.00042	hsa01524	0.269897
321	23	GO Process	response to oxygen levels	0.002	AHCY|ACTN4|ATP6V1A|ATP6V0D1|CAPN2|FABP1|HSP90B1|AQP1|PCK1|P4HB|PKLR|DPP4|LONP1|OPA1|ATP1B1|POSTN|ABAT|ALAD|HIGD1A|NDRG1|CRYAB|PHB2|DNM1L	0.00016	GO.0070482	0.269897

14	5	GO Process	protein folding in endoplasmic reticulum	0.002	CANX|HSP90B1|PDIA3|CALR|HSPA5	0.00016	GO.0034975	0.269897
14	5	GO Process	cysteine metabolic process	0.002	MTHFD1|AHCY|AHCYL1|MPST|GGT1	0.00016	GO.0006534	0.269897
21	10	GO Component	mitochondrial proton-transporting ATP synthase complex	8.8E-08	ATP5B|ATP5O|ATP5J2|ATP5L|ATP5H|ATP5I|ATP5C1|ATP5F1|USMG5|ATP5A1	8.88E-09	GO.0005753	0.70565055
161	15	GO Process	organic hydroxy compound biosynthetic process	0.0021	PNPO|SPR|SLC27A2|QDPR|PCK1|CYB5R3|HMGCS2|GOT1|SCP2|PSAT1|AKR1C3|AMACR|DDC|PAH|PLCG2	0.00017	GO.1901617	0.26777807
720	40	GO Process	cellular component morphogenesis	0.0021	VCL|MAPK1|MYH9|LAMA5|LAMC1|SLC9A3R1|LRP2|ADD1|AFG3L2|SOD1|UCHL1|ACTC1|HPRT1|DPYSL2|PARVA|HSP90AA1|EPB41L3|COL18A1|RAC1|MYH10|OPA1|KRT19|FLNA|CLIC4|FLOT1|POSTN|CD9|DNM2|CNP|ACTN1|MYH11|ILK|CDC42|PNPT1|SSBP1|RAP1GAP|DNM1L|ITGB3|ACTG1|TTN	0.00017	GO.0032989	0.26777807
143	14	GO Process	glycosyl compound metabolic process	0.0022	AHCY|AKR7A2|ABHD10|DPYS|HPRT1|PNP|OPA1|AHCYL1|CMPK1|AKR1C3|AK3|NME2|AK4|AMPD3	0.00017	GO.1901657	0.26575773
24	6	GO Process	one-carbon metabolic process	0.0022	MTHFD1|AHCY|ALDH1L1|FTCD|SHMT1|AHCYL1	0.00018	GO.0006730	0.26575773
25	6	Reactome Pathways	Mitochondrial biogenesis	0.0022	C19orf70|TMEM11|APOO|IMMT|SSBP1|SIRT5	0.00022	HSA-1592230	0.26575773
46	10	GO Component	mitochondrial respiratory chain complex I	2.8E-05	NDUFB4|NDUFA2|NDUFA10|NDUFA9|NDUFB9|NDUFB8|NDUFV1|NDUFA12|NDUFS1|NDUFA13	3.74E-06	GO.0005747	0.4552842
77	10	GO Component	cortical cytoskeleton	0.001	VCL|MYH9|DSTN|ACTN4|CAPN2|PLS1|KRT19|FLNA|ACTR2|RDX	0.00019	GO.0030863	0.3
122	10	GO Component	Z disc	0.0171	HSPB1|ACTN4|PARVA|KRT19|FLNA|JUP|ACTN1|FKBP1A|CRYAB|TTN	0.0047	GO.0030018	0.17670039
2219	95	GO Process	cellular response to organic substance	0.0023	RALA|DCN|MAPK1|LGALS1|CTSH|GAPDH|RAB35|SKP1|ATP6V1B1|CANX|VIL1|HSPB1|LAMA5|MRPL15|ATP6V0A1|ADD1|F13A1|CALB1|CPT1A|SOD1|ATP6V1A|ATP6V1B2|ATP6V0D2|ATP6V0D1|CAPN2|HSPA9|COL1A2|TPP1|HSP90B1|GPD1|YWHAG|ATP6V0A4|PRKACA|AQP1|TLN1|SHMT1|PCK1|CALR|BCAT2|HSPA5|FBN1|BGN|P4HB|RAB11B|HSP90AA1|PKLR|GNAI1|CTSB|ANXA2|MSN|COL4A2|MPC1|OPA1|GSTO1|AHCYL1|GOT1|GNAS|SLC25A5|ASS1|RAB14|ASPN|COL4A1|FLOT1|ACTR2|POSTN|AKR1C3|PFKP|GLDC|FECH|KRT18|DNM2|CORO1B|JUP|TSPO|ILK|GSTP1|CDC42|RDX|IFI30|PDIA6|ALAD|PNPT1|DDC|AOC1|MME|GSTA2|ACSL1|NDUFA13|ACADVL|PHB2|ATP1A1|VIM|DNM1L|ACTG1|PHB	0.00018	GO.0071310	0.26382722
649	37	GO Process	protein localization to organelle	0.0023	RAB35|SKP1|EHHADH|PMPCB|MTX2|CD81|SLC27A2|UQCRC2|TIMM44|CRAT|PIPOX|CALR|HSPA5|DHRS4|ACAA1|HSP90AA1|RPS2|SAMM50|AGK|SCP2|PMPCA|MPV17|AKR1C3|PITRM1|SLC25A6|AMACR|TSPO|SSB|IDH1|HK1|TOMM40|NDUFA13|EPHX2|PHB2|TIMM50|AP2B1|HAO2	0.00019	GO.0033365	0.26382722
7	4	GO Process	glycerol-3-phosphate metabolic process	0.0023	GPD1L|GPD1|GPD2|GK	0.00019	GO.0006072	0.26382722
26	6	GO Function	CoA-ligase activity	0.0024	SLC27A2|ACSS1|ACSM2B|ACSF2|ACSL1|ACSM2A	0.00026	GO.0016405	0.26197888
16	5	GO Function	alcohol dehydrogenase (NADP+) activity	0.0024	AKR7A2|DHRS4|AKR1A1|AKR1C3|RDH13	0.00026	GO.0008106	0.26197888

39	19	KEGG Pathways	Pyruvate metabolism	2.9E-14	PCK2|ALDH2|DLAT|LDHD|PDHB|GRHPR|ACSS1|PCK1|MDH2|PKLR|ALDH3A2|ALDH9A1|FH|ALDH1B1|PDHA1|PC|HAGH|ALDH7A1|ME3	1.21E-15	hsa00620	1.35451551
36	7	GO Process	porphyrin-containing compound metabolic process	0.0024	AMBP|BDH2|IBA57|SUCLA2|FECH|TSPO|ALAD	0.0002	GO.0006778	0.26197888
23	9	GO Component	proton-transporting two-sector ATPase complex, proton-transporting domain	1.6E-06	ATP6V0A1|ATP6V0D2|ATP6V0D1|ATP5J2|ATP5L|ATP5H|ATP5I|ATP6V0A4|ATP5F1	1.9E-07	GO.0033177	0.58041003
8	4	GO Function	peroxiredoxin activity	0.0025	PRDX1|PRDX5|PRDX3|PRDX6	0.00027	GO.0051920	0.260206
8	4	GO Function	nucleobase transmembrane transporter activity	0.0025	SLC25A4|SLC23A1|SLC25A5|SLC25A6	0.00027	GO.0015205	0.260206
10	4	KEGG Pathways	Synthesis and degradation of ketone bodies	0.0025	OXCT1|BDH2|HMGCS2|BDH1	0.00053	hsa00072	0.260206
94	11	GO Process	positive regulation of reactive oxygen species metabolic process	0.0025	AGXT2|NNT|SOD1|DDAH1|HSP90AA1|MT-CO2|ASS1|AKR1C3|DNM2|TSPO|GSTP1	0.00021	GO.2000379	0.260206
78	10	GO Process	protein homotetramerization	0.0025	DECR1|PFKL|DPYS|HPRT1|DCXR|SHMT1|GPX3|CRYZ|FBP1|DNM1L	0.00021	GO.0051289	0.260206
394	26	GO Process	nucleobase-containing compound catabolic process	0.0025	AHCY|OGDH|GAPDH|DNPH1|ENO1|GDA|PFKL|DPYS|HPRT1|HINT1|PKLR|RPS2|PFKM|PNP|OGDHL|ALDOB|DUT|PFKP|CNP|AMPD3|SSB|PNPT1|HK1|GPX1|RBM8A|GALK1	0.00021	GO.0034655	0.260206
15	5	GO Process	fatty acid beta-oxidation using acyl-CoA oxidase	0.0025	EHHADH|CRAT|ACAA1|SCP2|AMACR	0.00021	GO.0033540	0.260206
63	9	GO Process	response to activity	0.0025	OXCT1|UQCRC1|GOT2|MTFP1|PCK1|COL4A2|OPA1|POSTN|ALAD	0.00021	GO.0014823	0.260206
55	9	GO Component	tertiary granule lumen	0.00048	CTSH|LTA4H|LTF|CTSD|GGH|LYZ|NIT2|IDH1|METTL7A	8.25E-05	GO.1904724	0.33187588
1051	52	GO Function	transition metal ion binding	0.0026	UQCRC1|ACO2|LTA4H|LTF|GDA|PEPD|PMPCB|HRSP12|BHMT2|BBOX1|ENPEP|CALB1|SORD|UQCRC2|AFG3L2|SOD1|BHMT|LACTB2|DPYS|RNPEP|ADH5|ANPEP|CA4|ADH1B|PRKACA|ISCU|CYP4A11|AOC3|PCK1|CALR|C11orf54|TMLHE|VAT1|DPP3|MT-CO2|MARC2|CSRP1|RTN4IP1|PMPCA|MT1M|NQO2|PITRM1|FECH|DPEP1|SUOX|TF|ALAD|CRYZ|AOC1|MME|PAH|SIRT5	0.0003	GO.0046914	0.25850267
584	34	GO Process	cellular ion homeostasis	0.0028	MAPK1|SKP1|LTF|ATP5B|SLC9A3R1|ATP6V0A1|CALB1|RHCG|AFG3L2|SOD1|ATP6V1A|ATP6V0D2|ATP6V0D1|CKB|HSP90B1|LETM1|ATP6V0A4|ACO1|PRKACA|ISCU|CALR|ANXA6|STOML2|TGM2|ATP1B1|GSTO1|GOT1|CLIC4|TF|IMMT|SLC4A4|EPHX2|ATP1A1|PLCG2	0.00023	GO.0006873	0.2552842
62	9	GO Component	specific granule lumen	0.001	VCL|LTF|CTSD|GGH|LYZ|ACAA1|JUP|NIT2|AOC1	0.00019	GO.0035580	0.3
17	5	GO Function	oxidoreductase activity, acting on the CH-NH group of donors, NAD or NADP as acceptor	0.0029	MTHFD1|CRYM|ALDH1L1|QDPR|ALDH4A1	0.00033	GO.0016646	0.2537602

205	16	KEGG Pathways	Regulation of actin cytoskeleton	0.0029	VCL|MAPK1|MYH9|PFN1|ACTN4|IQGAP2|SCIN|RAC1|MSN|MYH10|ACTN1|CDC42|RDX|ARPC1B|ITGB3|ACTG1	0.00066	hsa04810	0.2537602
20	5	KEGG Pathways	One carbon pool by folate	0.0029	MTHFD1|ALDH1L1|AMT|FTCD|SHMT1	0.00063	hsa00670	0.2537602
36	11	KEGG Pathways	Tyrosine metabolism	6E-07	GSTZ1|GOT2|HGD|HPD|ADH5|ADH1B|AOC3|GOT1|MAOB|FAH|DDC	6.62E-08	hsa00350	0.62189631
288	21	GO Process	response to hypoxia	0.0029	AHCY|ACTN4|CAPN2|FABP1|HSP90B1|AQP1|PCK1|P4HB|PKLR|DPP4|LONP1|OPA1|ATP1B1|POSTN|ABAT|ALAD|HIGD1A|NDRG1|CRYAB|PHB2|DNM1L	0.00025	GO.0001666	0.2537602
17	5	Reactome Pathways	tRNA Aminoacylation	0.0029	CARS2|DARS2|IARS2|LARS2|SARS2	0.00033	HSA-379724	0.2537602
96	11	GO Process	maintenance of protein location	0.003	SKP1|SCIN|TLN1|HSPA5|FBN1|EPB41L3|FLNA|YWHAB|JUP|TSPO|HK1	0.00025	GO.0045185	0.25228787
836	44	GO Process	intracellular protein transport	0.003	EHHADH|HSPB1|PMPCB|MTX2|ERP29|NAPA|SLC27A2|UQCRC2|TIMM44|HSPA9|HSP90B1|YWHAG|CRAT|PIPOX|CALR|DHRS4|AP2A2|ACAA1|HSP90AA1|RPS2|SAMM50|AGK|ATP1B1|AHCYL1|MYO6|SCP2|PMPCA|YWHAB|MPV17|AKR1C3|PITRM1|SLC25A6|AMACR|TSPO|SSB|IDH1|TOMM40|NDUFA13|EPHX2|PHB2|TIMM50|RBM8A|AP2B1|HAO2	0.00025	GO.0006886	0.25228787
51	8	GO Process	purine-containing compound catabolic process	0.0031	AHCY|DNPH1|GDA|HPRT1|HINT1|PNP|AMPD3|GPX1	0.00026	GO.0072523	0.25086383
131	13	GO Process	cellular protein complex disassembly	0.0031	DSTN|VIL1|MRPL15|NAPA|MRPL49|MRPL17|MRPL39|MRPL13|MRPL12|MRPL37|MRPL21|MRPS22|MRPS30	0.00026	GO.0043624	0.25086383
16	5	GO Process	nonribosomal peptide biosynthetic process	0.0031	BDH2|CNDP2|HAGH|GGT5|GGT1	0.00026	GO.0019184	0.25086383
63	9	GO Component	actomyosin	0.0011	MYH9|ACTN4|ACTC1|TPM4|MYH10|CORO1B|ACTN1|ILK|SEPT7	0.00021	GO.0042641	0.29586073
312	22	GO Process	protein homooligomerization	0.0032	DECR1|CPT1A|PFKL|DPYS|HPRT1|DCXR|LETM1|SHMT1|EHD3|ANXA6|LONP1|TGM2|FLOT1|KCTD12|GPX3|MGST1|ALAD|CRYZ|PNPT1|FBP1|CRYAB|DNM1L	0.00027	GO.0051260	0.249485
148	13	KEGG Pathways	Retrograde endocannabinoid signaling	0.0033	NDUFB4|MAPK1|NDUFA2|NDUFA10|NDUFA9|NDUFB9|NDUFB8|PRKACA|NDUFV1|NDUFA12|GNAI1|NDUFS1|NDUFA13	0.00076	hsa04723	0.24814861
86	9	GO Component	mitochondrial ribosome	0.0066	MRPL15|MRPL49|MRPL17|MRPL39|MRPL13|MRPL12|MRPL37|MRPL21|MRPS22	0.0016	GO.0005761	0.21804561
9	4	GO Function	3-hydroxyacyl-CoA dehydrogenase activity	0.0034	EHHADH|CRYL1|HADHB|HADHA	0.00039	GO.0003857	0.24685211
189	16	GO Process	response to reactive oxygen species	0.0034	MAPK1|PPIF|PRDX1|PRDX5|SOD1|FABP1|PRDX3|AQP1|MPV17|AKR1C3|DNM2|DPEP1|GSTP1|TXNRD2|GPX1|CRYAB	0.00029	GO.0000302	0.24685211
652	36	GO Function	cation transmembrane transporter activity	0.0035	SLC25A3|ATP6V1B1|ATP5B|ATP6V0A1|NNT|RHCG|ATP6V1A|ATP6V1B2|ATP6V0D2|ATP5O|ATP6V0D1|COX7A1|ATP5L|ATP5H|SLC23A1|LETM1|ATP5I|ATP6V0A4|AQP1|COX5A|SLC7A8|SLC5A2|CYB5A|SLC25A15|ATP5C1|MT-CO1|MT-CO2|ATP1B1|ATP5F1|SLC3A2|ATP5A1|TF|SLC4A4|COX7C|ATP1A1|COX4I1	0.0004	GO.0008324	0.2455932
82	10	GO Process	fatty acid derivative biosynthetic process	0.0035	LTA4H|BDH2|PTGES2|MGST3|HMGCS2|AKR1C3|BDH1|GGT5|GGT1|ACSL1	0.0003	GO.1901570	0.2455932
52	8	GO Process	secondary metabolic process	0.0035	AKR7A2|SQRDL|BDH2|GSTO1|AKR1C3|MPST|DDC|FMO1	0.0003	GO.0019748	0.2455932

91	9	GO Component	platelet alpha granule	0.0091	ACTN4|F13A1|SERPINA5|APOOL|CD9|ACTN1|AHSG|SERPINA1|ITGB3	0.0023	GO.0031091	0.20409586
196	16	GO Function	magnesium ion binding	0.0036	IDH3G|ENO1|HPRT1|IDH3A|PRKACA|PCK1|IDH2|PKLR|GNAI1|PPA2|OPA1|LHPP|IDH3B|DUT|IDH1|EPHX2	0.00042	GO.0000287	0.24436975
27	6	GO Process	glutamine family amino acid catabolic process	0.0036	GOT2|DDAH1|PRODH2|GOT1|ALDH4A1|FAH	0.00031	GO.0009065	0.24436975
27	6	GO Process	folic acid-containing compound metabolic process	0.0036	MTHFD1|GGH|ALDH1L1|FTCD|PIPOX|SHMT1	0.00031	GO.0006760	0.24436975
3	3	GO Function	adenine transmembrane transporter activity	0.0037	SLC25A4|SLC25A5|SLC25A6	0.00044	GO.0015207	0.24317983
3	3	GO Function	ATP:ADP antiporter activity	0.0037	SLC25A4|SLC25A5|SLC25A6	0.00044	GO.0005471	0.24317983
3	3	GO Function	isocitrate dehydrogenase (NAD+) activity	0.0037	IDH3G|IDH3A|IDH3B	0.00044	GO.0004449	0.24317983
3	3	GO Function	6-phosphofructokinase activity	0.0037	PFKL|PFKM|PFKP	0.00044	GO.0003872	0.24317983
62	8	KEGG Pathways	Retinol metabolism	0.0037	ADH5|ALDH1A1|UGT2B7|ADH1B|CYP4A11|UGT2B17|DHRS4|UGT1A9	0.00086	hsa00830	0.24317983
134	13	GO Process	response to ethanol	0.0037	OXCT1|GOT2|GGH|SOD1|ACTC1|TUFM|RBP4|ACTR2|MAOB|FECH|ABAT|GSTP1|ALAD	0.00032	GO.0045471	0.24317983
71	9	GO Function	modified amino acid binding	0.0038	GSTM3|MUT|FTCD|SCIN|TLN1|PTGES2|DPEP1|MGST1|GSTP1	0.00046	GO.0072341	0.24202164
83	10	GO Process	iron ion homeostasis	0.0038	SKP1|LTF|SOD1|ATP6V1A|ATP6V0D1|BDH2|ACO1|ISCU|SFXN2|TF	0.00033	GO.0055072	0.24202164
17	5	GO Process	arginine metabolic process	0.0038	DDAH1|ASL|ASS1|AGMAT|FAH	0.00033	GO.0006525	0.24202164
17	5	GO Process	glycerol metabolic process	0.0038	GPD2|PCK1|GOT1|DAK|GK	0.00033	GO.0006071	0.24202164
17	5	GO Process	sulfur amino acid biosynthetic process	0.0038	MTHFD1|AHCY|BHMT2|BHMT|GGT1	0.00033	GO.0000097	0.24202164
524	31	GO Process	anion transport	0.0039	SLC25A11|GOT2|SLC9A3R1|LRP2|VDAC1|CPT1A|SLC27A2|SLC25A4|FABP1|CA4|SLC23A1|AQP1|SLC7A8|SLC25A15|MPC1|ABCD3|SCP2|SLC25A5|VDAC2|CLIC4|CLIC1|SLC3A2|SLC25A6|TSPO|PCYOX1|SLC25A12|SLC4A4|ACSL1|VDAC3|SLC25A10|NPC2	0.00034	GO.0006820	0.24089354
362	24	GO Process	translation	0.0039	CARS2|MRPL15|EIF4H|MRPL49|MRPL17|MRPL39|MRPL13|TSFM|TUFM|MRPL12|RPS2|PPA2|MRPL37|MRPL21|DARS2|IARS2|VARS|GGT5|GGT1|LARS2|MRPS22|GFM1|MRPS30|SARS2	0.00034	GO.0006412	0.24089354
233	18	GO Process	response to alcohol	0.004	OXCT1|GOT2|GGH|SOD1|ACTC1|GLB1|TUFM|GNAI1|GNAS|RBP4|ACTR2|MAOB|AKR1C3|FECH|JUP|ABAT|GSTP1|ALAD	0.00035	GO.0097305	0.239794
283	20	GO Function	GTPase activity	0.0041	RALA|RAB35|TUBA4A|MUT|TUBA1C|RAB1B|RHOT2|TUFM|RAB11B|TUBB4B|GNAI1|RAC1|OPA1|GNAS|RAB14|DNM2|CDC42|GFM1|RAP1GAP|DNM1L	0.0005	GO.0003924	0.23872161

4567	171	GO Process	cellular biosynthetic process	0.0041	DCN|NANS|MAPK1|GSTZ1|MTHFD1|AHCY|OGDH|PNPO|C1QBP|PDHX|LTA4H|GAPDH|DNPH1|AGXT2|LTF|SPR|ENO1|GDA|GOT2|BHMT2|GSTM3|CARS2|MRPL15|LRPPRC|ATP5B|CHCHD3|BBOX1|MECR|ATP6V0A1|VCAN|ALDH8A1|EIF4H|NDUFA9|LUM|SLC27A2|PFKL|SOD1|BHMT|MRPL49|DLAT|OXSM|ACAD8|QDPR|GPD1L|MRPL17|ATP5O|ATP5J2|BDH2|HPRT1|ATP5L|GPD1|ATP5H|HINT1|MRPL39|ATP5I|MRPL13|ASL|PDHB|ATP6V0A4|AQP1|GBAS|TSFM|ACSS1|CYC1|SHMT1|UGDH|PCK1|CHDH|TUFM|BCAT2|CNDP2|BGN|ACSM2B|IDH2|MRPL12|HSP90AA1|TMLHE|GSTA1|PKLR|RPS2|SLC25A15|PPA2|PTGES2|PFKM|ALDH3A2|GPX4|ALDH9A1|AGK|STOML2|ATP5C1|COL4A2|MRPL37|MPC1|PNP|MRPL21|DARS2|IBA57|IARS2|MGST3|LHPP|HMGCS2|PHGDH|GSTO1|ATP5F1|FLNA|ABCD3|GOT1|HOGA1|ALDH18A1|SCP2|CMPK1|AKR1A1|ASS1|RAB14|OGDHL|ALDOB|VARS|AGMAT|PSAT1|MAOB|SUCLA2|ESD|PDHA1|AKR1C3|DUT|PFKP|AMACR|FECH|SACM1L|BDH1|NME2|CNP|AK4|QPRT|MGST1|TSPO|AMPD3|ABAT|GATM|MPST|HAGH|GGT5|GSTP1|ATP5A1|GGT1|SMS|ALAD|ALDH7A1|TKT|NAPRT|GK|HK1|DDC|GPX1|FBP1|LARS2|GBE1|MRPS22|GFM1|SSBP1|GSTA2|ACSL1|MRPS30|EPHX2|PHB2|PAH|ACSM2A|GALK1|SARS2|PHB|PLCG2	0.00036	GO.0044249	0.23872161
57	8	GO Function	monocarboxylic acid binding	0.0042	HRSP12|FABP1|ADH5|SERPINA5|UGT1A9|SCP2|PCCA|PC	0.00052	GO.0033293	0.23767507
43	7	GO Function	disulfide oxidoreductase activity	0.0043	PDIA3|P4HB|PTGES2|GSTO1|GLRX|TXNRD2|PDIA6	0.00052	GO.0015036	0.23665315
96	9	GO Component	filopodium	0.0123	VIL1|SLC9A3R1|IQGAP2|ACTC1|MSN|CDC42|RDX|ACTA2|ITGB3	0.0032	GO.0030175	0.19100949
9	4	GO Process	valine metabolic process	0.0044	HIBADH|ACAD8|BCAT2|ALDH6A1	0.00039	GO.0006573	0.23565473
9	4	GO Process	fructose 6-phosphate metabolic process	0.0044	PFKL|PFKM|PFKP|FBP1	0.00039	GO.0006002	0.23565473
41	7	GO Process	acyl-CoA biosynthetic process	0.0045	PDHX|DLAT|PDHB|ACSS1|MPC1|PDHA1|ACSL1	0.00041	GO.0071616	0.23467875
102	11	GO Process	polyol metabolic process	0.0045	SPR|SORD|QDPR|GPD2|PCK1|GOT1|SCP2|DAK|GK|GALK1|PLCG2	0.0004	GO.0019751	0.23467875
13	8	GO Component	mitochondrial respiratory chain complex III	5.6E-07	UQCRC1|PMPCB|UQCRC2|UQCRH|CYC1|UQCR10|MT-CO1|BCS1L	6.32E-08	GO.0005750	0.62541448
18	5	GO Process	tetrahydrofolate metabolic process	0.0046	MTHFD1|ALDH1L1|FTCD|PIPOX|SHMT1	0.00042	GO.0046653	0.23372422
50	8	GO Component	stress fiber	0.0012	MYH9|ACTN4|TPM4|MYH10|CORO1B|ACTN1|ILK|SEPT7	0.00023	GO.0001725	0.29208188
70	9	GO Process	purine ribonucleoside metabolic process	0.0047	AHCY|HPRT1|PNP|OPA1|AHCYL1|AK3|NME2|AK4|AMPD3	0.00042	GO.0046128	0.23279021
65	8	KEGG Pathways	Central carbon metabolism in cancer	0.0048	MAPK1|PFKL|PDHB|PFKM|PDHA1|PFKP|IDH1|HK1	0.0011	hsa05230	0.23187588

<table>
<tr><td>4087</td><td>154</td><td>GO Function</td><td>metal ion binding</td><td>0.0049</td><td>UQCRC1|RTCB|ACO2|NID2|PCK2|IDH3G|F9|OGDH|LTA4H|LTF|ENO1|GDA|PEPD|CANX|VIL1|PMPCB|ACTN4|HRSP12|APCS|BHMT2|CARS2|ADAM10|BBOX1|EHD2|LRP2|F13A1|VCAN|ENPEP|CALB1|SORD|UQCRC2|AFG3L2|PFKL|BCKDHA|SOD1|BHMT|MUT|LACTB2|DPYS|HGD|DDAH1|HPD|CAPN2|RNPEP|ADH5|SCIN|COL1A2|HPRT1|TPP1|IDH3A|CDH16|HSP90B1|ANPEP|CA4|LETM1|ADH1B|CYCS|GPD2|ACO1|PRKACA|ISCU|CSRP2|CYP4A11|AOC3|CYC1|COX5A|PCK1|CALR|RHOT2|NDUFV1|HSPA5|FBN1|CNDP2|EHD3|ACSM2B|C11orf54|IDH2|TMLHE|PLS1|PKLR|CYB5A|GNAI1|PPA2|TPM4|PFKM|ANXA2|ANXA6|COL18A1|VAT1|DPP3|MT-CO1|OPA1|MT-CO2|TGM2|MARC2|CSRP1|S100A10|LHPP|RTN4IP1|GNAS|XPNPEP2|PMPCA|CISD1|OGDHL|HSPG2|ASPN|AGMAT|PCCA|CUBN|SUCLA2|POSTN|MT1M|HBD|NQO2|IDH3B|DUT|PITRM1|PFKP|FECH|DPEP1|NME2|PC|SUOX|FHL1|ACTN1|DAK|UMOD|PDP1|AMPD3|ABAT|HAGH|ACY1|FAH|TF|ALAD|SLC25A12|IDH1|TKT|NDUFS1|CRYZ|FBP1|AOC1|MME|ETFDH|EPHX2|CRYAB|ME3|ATP1A1|MYL6|PAH|ACSM2A|TTN|SIRT5|PDLIM5</td><td>0.00061</td><td>GO.0046872</td><td>0.23098039</td></tr>
<tr><td>48</td><td>26</td><td>KEGG Pathways</td><td>Valine, leucine and isoleucine degradation</td><td>4.6E-20</td><td>OXCT1|AGXT2|EHHADH|ACADS|ALDH2|HIBADH|BCKDHA|MUT|ACAD8|BCKDHB|BCAT2|HADHB|ACAA1|ALDH3A2|ALDH9A1|ECHS1|HMGCS2|AUH|PCCA|ALDH1B1|HADHA|ABAT|ALDH7A1|IVD|PCCB|ALDH6A1</td><td>8.03E-22</td><td>hsa00280</td><td>1.93419886</td></tr>
<tr><td>177</td><td>15</td><td>GO Process</td><td>regulation of blood pressure</td><td>0.0049</td><td>ENPEP|SOD1|DDAH1|RNPEP|ADH5|COL1A2|ANPEP|CYP4A11|POSTN|PRCP|ABAT|ACTA2|MME|EPHX2|ATP1A1</td><td>0.00044</td><td>GO.0008217</td><td>0.23098039</td></tr>
<tr><td>3</td><td>3</td><td>GO Process</td><td>NADH oxidation</td><td>0.0049</td><td>GPD1|GPD2|ALDOB</td><td>0.00044</td><td>GO.0006116</td><td>0.23098039</td></tr>
<tr><td>3</td><td>3</td><td>GO Process</td><td>fumarate metabolic process</td><td>0.0049</td><td>GOT2|FH|GOT1</td><td>0.00044</td><td>GO.0006106</td><td>0.23098039</td></tr>
<tr><td>20</td><td>5</td><td>GO Function</td><td>metalloaminopeptidase activity</td><td>0.005</td><td>LTA4H|ENPEP|RNPEP|ANPEP|XPNPEP2</td><td>0.00063</td><td>GO.0070006</td><td>0.230103</td></tr>
<tr><td>20</td><td>5</td><td>GO Function</td><td>protein disulfide isomerase activity</td><td>0.005</td><td>PDIA3|P4HB|TXNDC5|PDIA6|ITGB3</td><td>0.00063</td><td>GO.0003756</td><td>0.230103</td></tr>
<tr><td>52</td><td>8</td><td>GO Component</td><td>brush border membrane</td><td>0.0015</td><td>SLC9A3R1|LRP2|AMN|CA4|ATP6V0A4|AQP1|CUBN|PDZK1</td><td>0.0003</td><td>GO.0031526</td><td>0.28239087</td></tr>
<tr><td>42</td><td>7</td><td>GO Process</td><td>neurotransmitter biosynthetic process</td><td>0.0051</td><td>AGXT2|SPR|SHMT1|ALDH9A1|ABAT|DDC|PAH</td><td>0.00046</td><td>GO.0042136</td><td>0.22924298</td></tr>
<tr><td>104</td><td>11</td><td>GO Process</td><td>fatty acid biosynthetic process</td><td>0.0052</td><td>LTA4H|MECR|OXSM|ACSS1|ACSM2B|PTGES2|GPX4|ABCD3|AKR1C3|EPHX2|ACSM2A</td><td>0.00047</td><td>GO.0006633</td><td>0.22839967</td></tr>
<tr><td>53</td><td>8</td><td>GO Component</td><td>cortical actin cytoskeleton</td><td>0.0017</td><td>VCL|MYH9|DSTN|CAPN2|PLS1|KRT19|ACTR2|RDX</td><td>0.00033</td><td>GO.0030864</td><td>0.27695511</td></tr>
<tr><td>11</td><td>4</td><td>GO Function</td><td>glutathione binding</td><td>0.0055</td><td>GSTM3|PTGES2|MGST1|GSTP1</td><td>0.00071</td><td>GO.0043295</td><td>0.22596373</td></tr>
<tr><td>270</td><td>19</td><td>GO Function</td><td>carbohydrate binding</td><td>0.0055</td><td>LGALS1|VTN|MLEC|CANX|LGALS3|APCS|TINAG|VCAN|SORD|PFKL|TINAGL1|GAA|CALR|PFKM|ALDOB|HK1|FBP1|MGAM|GALK1</td><td>0.00071</td><td>GO.0030246</td><td>0.22596373</td></tr>
<tr><td>598</td><td>33</td><td>GO Function</td><td>inorganic cation transmembrane transporter activity</td><td>0.0055</td><td>SLC25A3|ATP6V1B1|ATP5B|ATP6V0A1|NNT|ATP6V1A|ATP6V1B2|ATP6V0D2|ATP5O|ATP6V0D1|COX7A1|ATP5L|ATP5H|SLC23A1|LETM1|ATP5I|ATP6V0A4|AQP1|COX5A|SLC5A2|CYB5A|ATP5C1|MT-CO1|MT-CO2|ATP1B1|ATP5F1|SLC3A2|ATP5A1|TF|SLC4A4|COX7C|ATP1A1|COX4I1</td><td>0.0007</td><td>GO.0022890</td><td>0.22596373</td></tr>
<tr><td>11</td><td>4</td><td>GO Function</td><td>dipeptidyl-peptidase activity</td><td>0.0055</td><td>DPP3|DPP4|DPEP1|PRCP</td><td>0.00071</td><td>GO.0008239</td><td>0.22596373</td></tr>
<tr><td>30</td><td>6</td><td>GO Process</td><td>retinol metabolic process</td><td>0.0055</td><td>TTR|ALDH1A1|DHRS4|RBP4|AKR1C3|RDH13</td><td>0.0005</td><td>GO.0042572</td><td>0.22596373</td></tr>
<tr><td>30</td><td>6</td><td>GO Process</td><td>heme metabolic process</td><td>0.0055</td><td>AMBP|BDH2|IBA57|FECH|TSPO|ALAD</td><td>0.0005</td><td>GO.0042168</td><td>0.22596373</td></tr>
</table>

30	6	GO Process	cortical actin cytoskeleton organization	0.0055	VIL1|EHD2|TLN1|CALR|PLS1|EPB41L3	0.0005	GO.0030866	0.22596373
30	6	GO Process	positive regulation of sodium ion transport	0.0055	ACTN4|GPD1L|ATP1B1|AHCYL1|GLRX|DNM2	0.0005	GO.0010765	0.22596373
892	45	GO Process	positive regulation of transport	0.0056	OXCT1|MAPK1|VTN|GAPDH|ACTN4|LGALS3|EHD2|GPD1L|SCIN|YWHAG|PRKACA|CYP4A11|AQP1|GBAS|DAB2|CALR|EHD3|PFKM|ANXA2|MSN|MYH10|OPA1|ATP1B1|GSTO1|AHCYL1|FLNA|RBP4|SCP2|YWHAB|FLOT1|POSTN|GLRX|DNM2|JUP|FHL1|TSPO|ABAT|RDX|TF|AHSG|PDZK1|DNM1L|TTN|PLCG2|AP2B1	0.00051	GO.0051050	0.2251812
19	5	GO Process	glyceraldehyde-3-phosphate metabolic process	0.0056	KHK|ALDH1A1|ALDOB|DAK|TKT	0.00051	GO.0019682	0.2251812
84	9	KEGG Pathways	Salmonella infection	0.0057	MAPK1|MYH9|PFN1|RAC1|MYH10|FLNA|CDC42|ARPC1B|ACTG1	0.0014	hsa05132	0.22441251
142	13	GO Process	protein processing	0.0058	UQCRC1|F9|CTSH|PMPCB|ADAM10|ENPEP|UQCRC2|AFG3L2|CAPN2|STOML2|PMPCA|GGT1|MME	0.00053	GO.0016485	0.2236572
10	4	GO Process	lysine catabolic process	0.0058	CRYM|PIPOX|DLST|ALDH7A1	0.00053	GO.0006554	0.2236572
10	4	GO Process	membrane raft assembly	0.0058	ANXA2|S100A10|FLOT1|FLOT2	0.00053	GO.0001765	0.2236572
272	19	Reactome Pathways	Innate Immune System	0.0058	PGRMC1|MLEC|LGALS3|GGH|LYZ|COTL1|CCT2|ANPEP|PRDX6|VAT1|PIGR|TXNDC5|NIT2|MVP|MME|IST1|MGAM|METTL7A|NPC2	0.00078	HSA-168249	0.2236572
53	8	GO Component	mitochondrial large ribosomal subunit	0.0017	MRPL15|MRPL49|MRPL17|MRPL39|MRPL13|MRPL12|MRPL37|MRPL21	0.00033	GO.0005762	0.27695511
74	8	GO Component	recycling endosome membrane	0.0095	RAB35|EHD2|PDIA3|EHD3|RAB11B|RAC1|RAB14|NDRG1	0.0024	GO.0055038	0.20222764
124	12	GO Process	cellular response to reactive oxygen species	0.0059	MAPK1|PPIF|PRDX1|PRDX5|SOD1|FABP1|PRDX3|AQP1|MPV17|AKR1C3|DNM2|DPEP1	0.00055	GO.0034614	0.2229148
33	6	GO Function	ADP binding	0.006	MYH9|MYH10|LONP1|CYB5R3|MYO6|ATP1A1	0.00079	GO.0043531	0.22218487
61	8	GO Function	collagen binding	0.006	DCN|NID2|VTN|LUM|COL14A1|CTSB|ASPN|TGFBI	0.00078	GO.0005518	0.22218487
202	16	GO Process	protein maturation	0.0062	UQCRC1|F9|CTSH|PMPCB|ADAM10|ENPEP|UQCRC2|AFG3L2|CAPN2|ISCU|CALR|STOML2|PMPCA|FKBP1A|GGT1|MME	0.00057	GO.0051604	0.22076083
100	8	GO Component	clathrin-coated vesicle membrane	0.0411	RAB35|LRP2|DAB2|AP2A2|MYO6|CD9|TF|AP2B1	0.0125	GO.0030665	0.13861582
53	7	KEGG Pathways	Pathogenic Escherichia coli infection	0.0063	TUBA4A|TUBA1C|TUBB4B|KRT18|CDC42|ARPC1B|ACTG1	0.0016	hsa05130	0.22006595
161	13	KEGG Pathways	Protein processing in endoplasmic reticulum	0.0063	SKP1|CANX|ERP29|CAPN2|HSP90B1|PDIA3|CALR|HSPA5|P4HB|HSP90AA1|TXNDC5|PDIA6|CRYAB	0.0016	hsa04141	0.22006595
44	7	GO Process	purine nucleotide catabolic process	0.0064	DNPH1|GDA|HPRT1|HINT1|PNP|AMPD3|GPX1	0.00059	GO.0006195	0.219382
288	20	GO Process	blood coagulation	0.0065	MAPK1|F9|C1QBP|EHD2|F13A1|COL1A2|PRKACA|EHD3|SERPINA5|RAC1|FLNA|GNAS|HBD|AK3|CD9|PRCP|CDC42|SERPINA1|ITGB3|PLCG2	0.00061	GO.0007596	0.21870866
101	8	GO Component	Golgi lumen	0.0431	DCN|F9|VTN|VCAN|LUM|BGN|HSPG2|UMOD	0.0131	GO.0005796	0.13655227

26	5	KEGG Pathways	Folate biosynthesis	0.0068	SPR|GGH|QDPR|AKR1C3|PAH	0.0017	hsa00790	0.21674911
91	10	GO Process	mitochondrial translational termination	0.0068	MRPL15|MRPL49|MRPL17|MRPL39|MRPL13|MRPL12|MRPL37|MRPL21|MRPS22|MRPS30	0.00064	GO.0070126	0.21674911
91	10	GO Process	regulation of pH	0.0068	MAPK1|ATP6V1B1|ATP5B|ATP6V0A1|RHCG|ATP6V0D2|ATP6V0D1|ATP6V0A4|CLIC4|SLC4A4	0.00064	GO.0006885	0.21674911
25	7	GO Component	proton-transporting V-type ATPase complex	0.00018	ATP6V1B1|ATP6V0A1|ATP6V1A|ATP6V1B2|ATP6V0D2|ATP6V0D1|ATP6V0A4	2.75E-05	GO.0033176	0.37447275
31	7	GO Component	smooth endoplasmic reticulum	0.00051	PGRMC1|CANX|ERP29|FTCD|PPIB|CALR|HSPA5	0.000089	GO.0005790	0.32924298
109	11	GO Process	cellular hormone metabolic process	0.0071	TTR|ALDH8A1|ALDH1A1|UGT2B7|DHRS4|UGT1A9|RBP4|SCP2|AKR1C3|TSPO|RDH13	0.00067	GO.0034754	0.21487417
109	11	GO Process	positive regulation of cell-substrate adhesion	0.0071	C1QBP|VTN|CALR|RAC1|S100A10|FLNA|RSU1|DNM2|JUP|ILK|CDC42	0.00067	GO.0010811	0.21487417
234	17	GO Function	sulfur compound binding	0.0073	OGDH|VTN|LTF|GSTM3|FBN1|SERPINA5|PTGES2|SCP2|OGDHL|PCCA|POSTN|HADHA|PC|MGST1|GSTP1|AOC1|ALDH6A1	0.00097	GO.1901681	0.21366771
40	6	KEGG Pathways	Ferroptosis	0.0074	GPX4|VDAC2|SLC3A2|TF|ACSL1|VDAC3	0.0019	hsa04216	0.21307683
166	14	GO Process	protein stabilization	0.0075	PFN1|GAPDH|CCT5|CCT2|PPIB|CALR|HSP90AA1|ATP1B1|FLNA|FLOT1|FLOT2|CRYAB|PHB2|PHB	0.00071	GO.0050821	0.21249387
11	4	GO Process	cellular ketone body metabolic process	0.0075	OXCT1|BDH2|HMGCS2|BDH1	0.00071	GO.0046950	0.21249387
186	15	GO Process	hormone metabolic process	0.0075	CRYM|TTR|ENPEP|ALDH8A1|ALDH1A1|UGT2B7|DHRS4|UGT1A9|ALDH9A1|RBP4|SCP2|AKR1C3|TSPO|RDH13|MME	0.00071	GO.0042445	0.21249387
11	4	GO Process	xenobiotic catabolic process	0.0075	GSTM3|GSTO1|CRYZ|ACSL1	0.00071	GO.0042178	0.21249387
11	4	GO Process	creatine metabolic process	0.0075	CKB|GATM|CKMT2|CKMT1A	0.00071	GO.0006600	0.21249387
4	3	GO Process	valine catabolic process	0.0078	HIBADH|ACAD8|ALDH6A1	0.00075	GO.0006574	0.21079054
129	12	GO Process	positive regulation of endocytosis	0.0079	VTN|ACTN4|DAB2|CALR|ANXA2|FLOT1|DNM2|TF|AHSG|DNM1L|PLCG2|AP2B1	0.00076	GO.0045807	0.21023729
832	42	GO Process	cellular protein-containing complex assembly	0.008	NDUFB4|C1QBP|VTN|VIL1|NDUFA2|NDUFA10|APCS|PSMD11|ATP6V0A1|EIF4H|NDUFA9|ECSIT|NDUFB9|NDUFB8|CCT2|ATP6V0A4|ACAD9|CALR|NDUFV1|NDUFA12|UQCR10|HSP90AA1|SAMM50|GPX4|RAC1|LONP1|OPA1|COA3|ALDOB|DNM2|MYH11|FKBP1A|NDUFS1|COX20|BCS1L|ATPAF2|TIMMDC1|NDUFA13|CRYAB|DNM1L|TTN|AP2B1	0.00077	GO.0034622	0.209691
61	7	GO Component	ficolin-1-rich granule membrane	0.0124	LGALS3|ATP6V0A1|GAA|AP2A2|RAC1|PRCP|MGAM	0.0033	GO.0101003	0.19065783
1626	71	GO Process	tissue development	0.0081	RALA|DCN|OXCT1|VCL|MAPK1|MTHFD1|CTSH|PFN1|VTN|DNPH1|VIL1|LAMA5|LGALS3|GRSF1|GSTM3|LAMC1|SLC9A3R1|LRP2|VDAC1|CALB1|CPT1A|LUM|RHCG|SOD1|ACTC1|BDH2|TPP1|GAA|PRKACA|DAB2|CALR|GSTA1|CTSB|ALDH3A2|COL18A1|MSN|MYH10|COL4A2|KRT19|TGM2|PHGDH|FLNA|GNAS|RBP4|CLIC4|ASPN|COL4A1|POSTN|AKR1C3|KRT18|JUP|FLOT2|UMOD|MYH11|ILK|FKBP1A|CDC42|RDX|TST|ACTA2|GPX1|TGFBI|GSTA2|TAGLN|ACADVL|PHB2|VIM|MYL6|ITGB3|TTN|PDLIM5	0.00079	GO.0009888	0.2091515

251	18	GO Process	regulation of protein stability	0.0082	MAPK1|CTSH|PFN1|GAPDH|CD81|CCT5|CCT2|PPIB|CALR|HSP90AA1|ATP1B1|FLNA|FLOT1|FLOT2|TF|CRYAB|PHB2|PHB	0.0008	GO.0031647	0.20861861
47	7	GO Process	pigment biosynthetic process	0.0087	MTHFD1|HPRT1|SHMT1|IBA57|FECH|TSPO|ALAD	0.00084	GO.0046148	0.20604807
13	4	GO Function	NADPH binding	0.0088	DECR1|QDPR|GRHPR|CRYZ	0.0012	GO.0070402	0.20555173
5	3	GO Function	fructose binding	0.0088	PFKL|PFKM|ALDOB	0.0012	GO.0070061	0.20555173
13	4	GO Function	proton-transporting ATP synthase activity, rotational mechanism	0.0088	ATP5B|ATP5O|ATP5C1|ATP5A1	0.0012	GO.0046933	0.20555173
5	3	GO Function	thioredoxin peroxidase activity	0.0088	PRDX1|PRDX5|PRDX3	0.0012	GO.0008379	0.20555173
13	4	GO Function	omega peptidase activity	0.0088	GGH|UCHL1|GGT5|GGT1	0.0012	GO.0008242	0.20555173
5	3	GO Function	long-chain-acyl-CoA dehydrogenase activity	0.0088	ACAD11|ACAD9|ACADVL	0.0012	GO.0004466	0.20555173
62	8	GO Process	organic hydroxy compound catabolic process	0.0088	ALDH2|SORD|GPD2|CYP4A11|ALDH1B1|MAOB|AKR1C3|GK	0.00086	GO.1901616	0.20555173
67	7	GO Component	cell-cell contact zone	0.0185	VCL|ATP1B1|FLOT1|JUP|ACTN1|FLOT2|ATP1A1	0.0052	GO.0044291	0.17328283
34	6	GO Process	pH reduction	0.0093	ATP6V1B1|ATP6V0A1|ATP6V0D2|ATP6V0D1|ATP6V0A4|CLIC4	0.0009	GO.0045851	0.20315171
22	5	GO Process	toxin metabolic process	0.0093	SQRDL|GSTO1|MPST|DDC|FMO1	0.00091	GO.0009404	0.20315171
22	5	GO Process	aspartate family amino acid biosynthetic process	0.0093	MTHFD1|GOT2|BHMT2|BHMT|GOT1	0.00091	GO.0009067	0.20315171
22	5	GO Process	vacuolar acidification	0.0093	ATP6V0A1|ATP6V0D2|ATP6V0D1|ATP6V0A4|CLIC4	0.00091	GO.0007035	0.20315171
22	5	GO Process	mitochondrial genome maintenance	0.0093	SLC25A4|MRPL17|LONP1|OPA1|MPV17	0.00091	GO.0000002	0.20315171
138	12	GO Function	tetrapyrrole binding	0.0094	PGRMC1|AMBP|MUT|CYCS|CYP4A11|CYC1|CYB5A|PTGES2|MT-CO1|CUBN|HBD|SUOX	0.0013	GO.0046906	0.20268721
12	4	GO Process	ketone body metabolic process	0.0094	OXCT1|BDH2|HMGCS2|BDH1	0.00092	GO.1902224	0.20268721
67	7	GO Component	actin filament	0.0185	ACTC1|PLS1|TPM4|FLNA|MYO6|CORO1B|ACTG1	0.0052	GO.0005884	0.17328283
700	36	GO Function	calcium ion binding	0.0096	NID2|F9|CANX|VIL1|ACTN4|APCS|EHD2|LRP2|VCAN|CALB1|CAPN2|SCIN|CDH16|HSP90B1|LETM1|GPD2|AOC3|CALR|RHOT2|HSPA5|FBN1|EHD3|PLS1|TPM4|ANXA2|ANXA6|S100A10|HSPG2|ASPN|CUBN|ACTN1|UMOD|SLC25A12|AOC1|MYL6|TTN	0.0013	GO.0005509	0.20177288
63	8	GO Process	chaperone-mediated protein folding	0.0096	TRAP1|CANX|HSPB1|CCT5|CCT2|PPIB|CALR|FKBP1A	0.00094	GO.0061077	0.20177288
255	18	GO Process	aging	0.0096	DCN|CTSC|CANX|SOD1|PCK1|CALR|COL4A2|LONP1|MT-CO1|OPA1|ASS1|CNP|TSPO|ABAT|ILK|DDC|MME|CRYAB	0.00094	GO.0007568	0.20177288
48	7	GO Process	tRNA aminoacylation for protein translation	0.0096	CARS2|PPA2|DARS2|IARS2|VARS|LARS2|SARS2	0.00094	GO.0006418	0.20177288

213	16	GO Process	response to insulin	0.0098	ATP6V1B1|GOT2|GGH|KHK|ATP6V0A1|ATP6V1A|ATP6V1B2|ATP6V0D2|ATP6V0D1|YWHAG|ATP6V0A4|PCK1|PKLR|GOT1|HADHA|GSTP1	0.00097	GO.0032868	0.20087739
3267	126	GO Process	response to stress	0.0098	DCN|NDUFB4|OXCT1|MAPK1|LGALS1|ACO2|AHCY|F9|PLGRKT|PPIF|C1QBP|GAPDH|SKP1|LTF|TRAP1|HSPB1|ACTN4|LGALS3|APCS|GSTM3|LYZ|COTL1|PRDX1|EHD2|ADD1|F13A1|PRDX5|SORD|SOD1|ECSIT|ERLIN2|UCHL1|ATP6V0D1|CAPN2|FABP1|ADH5|COL1A2|PRDX3|TPP1|HSP90B1|PDIA3|CYCS|PRKACA|AQP1|AOC3|MSRA|TLN1|PCK1|CALR|HSPA5|EHD3|P4HB|NDUFA12|SERPINA5|HSP90AA1|PKLR|TUBB4B|PRDX6|TPSAB1|GPX4|COL18A1|RAC1|STOML2|MYH10|DPP4|LONP1|MT-CO1|OPA1|MT-CO2|C4BPA|ATP1B1|FLNA|MYO6|GOT1|GNAS|ASS1|RAB14|HSPG2|FLOT1|ACTR2|POSTN|TXNDC5|MPV17|HBD|AKR1C3|AK3|CD9|GPX3|DNM2|DPEP1|PRCP|CORO1B|DAK|UMOD|MGST1|TSPO|ABAT|GGT5|GSTP1|CDC42|TXNRD2|IFI30|PDIA6|ALAD|IDH1|AHSG|HIGD1A|PNPT1|NAPRT|NDRG1|GPX1|AOC1|SERPINA1|VWA1|ETFDH|VDAC3|EPHX2|CRYAB|ACADVL|PHB2|VIM|DNM1L|ITGB3|ACTG1|FMO1|PLCG2	0.00097	GO.0006950	0.20087739
179	14	GO Function	transferase activity, transferring acyl groups other than amino-acyl groups	0.0099	CPT1A|NAT8|DLAT|OXSM|GLYATL1|CRAT|HADHB|ACSM2B|ACAA1|DLST|SCP2|HADHA|ACSM2A|GLYAT	0.0014	GO.0016747	0.20043648
323	21	GO Process	response to acid chemical	0.01	CTSH|CPT1A|CAPN2|COL1A2|AQP1|SHMT1|PCK1|COL18A1|OPA1|GNAS|RBP4|ASS1|COL4A1|AKR1C3|GLDC|CD9|GSTP1|ALAD|ACSL1|NDUFA13|PHB2	0.001	GO.0001101	0.2
71	7	GO Component	phagocytic vesicle membrane	0.024	ATP6V0A1|ATP6V0D2|ATP6V0D1|ATP6V0A4|CALR|RAB11B|DNM2	0.007	GO.0030670	0.16197888
64	8	GO Process	maintenance of protein location in cell	0.0103	SKP1|SCIN|TLN1|HSPA5|EPB41L3|JUP|TSPO|HK1	0.001	GO.0032507	0.19871628
14	4	GO Function	phosphotransferase activity, nitrogenous group as acceptor	0.0105	CKB|NME2|CKMT2|CKMT1A	0.0015	GO.0016775	0.19788107
2250	91	GO Function	transferase activity	0.0105	OXCT1|NANS|MAPK1|GSTZ1|PDHX|GAPDH|AGXT2|SKP1|GOT2|BHMT2|GSTM3|KHK|F13A1|CPT1A|PFKL|NAT8|ALDH1L1|AMT|BHMT|ERLIN2|HNMT|DLAT|OXSM|FTCD|HPRT1|CKB|GLYATL1|UGT2B7|PRKACA|ISCU|CRAT|SHMT1|UGT2B17|BCAT2|HADHB|ACSM2B|ACAA1|HSP90AA1|DLST|GSTA1|PKLR|CS|PFKM|UGT1A9|AGK|RAC1|PNP|TGM2|IBA57|MGST3|HMGCS2|GSTO1|GOT1|ALDH18A1|SCP2|CMPK1|COMTD1|PSAT1|HADHA|PFKP|AK3|NME2|DAK|AK4|QPRT|MGST1|ABAT|GATM|ILK|MPST|GGT5|GSTP1|CDC42|GGT1|SMS|TST|TKT|PNPT1|NAPRT|GK|HK1|CKMT2|CKMT1A|GBE1|GSTA2|METTL7A|ACSM2A|GALK1|TTN|SIRT5|GLYAT	0.0015	GO.0016740	0.19788107
14	4	GO Function	opsonin binding	0.0105	C1QBP|APCS|CALR|PHB	0.0015	GO.0001846	0.19788107
953	46	GO Process	cellular response to cytokine stimulus	0.0107	RALA|DCN|MAPK1|GAPDH|SKP1|CANX|LAMA5|MRPL15|F13A1|SOD1|HSPA9|COL1A2|HSP90B1|GPD1|PRKACA|SHMT1|PCK1|BCAT2|HSPA5|BGN|P4HB|HSP90AA1|ANXA2|MSN|MPC1|GSTO1|SLC25A5|ASS1|ASPN|ACTR2|POSTN|PFKP|GLDC|KRT18|ILK|CDC42|IFI30|ALAD|PNPT1|MME|GSTA2|ACSL1|NDUFA13|VIM|ACTG1|PHB	0.0011	GO.0071345	0.19706162
23	5	GO Process	mitochondrial calcium ion homeostasis	0.0107	AFG3L2|LETM1|ANXA6|TGM2|IMMT	0.0011	GO.0051560	0.19706162
98	10	GO Process	cellular monovalent inorganic cation homeostasis	0.0107	MAPK1|ATP5B|ATP6V0A1|ATP6V0D2|ATP6V0D1|ATP6V0A4|ATP1B1|CLIC4|SLC4A4|ATP1A1	0.0011	GO.0030004	0.19706162

442	26	GO Process	regulation of cell morphogenesis	0.0107	MYH9|C1QBP|VIL1|ACTN4|SLC9A3R1|DPYSL2|CALR|PARVA|EPB41L3|RAC1|MSN|MYH10|OPA1|S100A10|FLNA|ACTR2|POSTN|DNM2|CORO1B|ILK|SEPT7|CDC42|RDX|IST1|DNM1L|PDLIM5	0.0011	GO.0022604	0.19706162
98	10	GO Process	ribonucleoside metabolic process	0.0107	AHCY|HPRT1|PNP|OPA1|AHCYL1|CMPK1|AK3|NME2|AK4|AMPD3	0.0011	GO.0009119	0.19706162
23	5	GO Process	porphyrin-containing compound biosynthetic process	0.0107	IBA57|SUCLA2|FECH|TSPO|ALAD	0.0011	GO.0006779	0.19706162
195	14	KEGG Pathways	Proteoglycans in cancer	0.0111	DCN|MAPK1|VTN|LUM|PRKACA|RAC1|MSN|FLNA|HSPG2|CDC42|RDX|ITGB3|ACTG1|PLCG2	0.0029	hsa05205	0.1954677
44	6	KEGG Pathways	Aminoacyl-tRNA biosynthesis	0.0111	CARS2|DARS2|IARS2|VARS|LARS2|SARS2	0.0029	hsa00970	0.1954677
16	6	GO Component	mitochondrial respiratory chain complex IV	0.00017	UQCRC2|COX5A|MT-CO1|MT-CO2|COX7C|COX4I1	2.72E-05	GO.0005751	0.37695511
13	4	GO Process	positive regulation of sodium ion transmembrane transporter activity	0.0115	ACTN4|ATP1B1|GLRX|DNM2	0.0012	GO.2000651	0.19393022
13	4	GO Process	nitrogen cycle metabolic process	0.0115	ASL|SLC25A15|ASS1|SUOX	0.0012	GO.0071941	0.19393022
36	6	GO Process	cellular metabolic compound salvage	0.0115	BHMT2|BHMT|HPRT1|PNP|AMPD3|NAPRT	0.0012	GO.0043094	0.19393022
13	4	GO Process	4-hydroxyproline metabolic process	0.0115	GOT2|P4HB|HOGA1|ALDH4A1	0.0012	GO.0019471	0.19393022
5	3	GO Process	4-hydroxyproline catabolic process	0.0115	GOT2|HOGA1|ALDH4A1	0.0012	GO.0019470	0.19393022
50	7	GO Process	negative regulation of mitochondrion organization	0.0115	PPIF|VAT1|OPA1|SLC25A5|TSPO|GPX1|DNM1L	0.0012	GO.0010823	0.19393022
136	12	GO Process	positive regulation of cell morphogenesis involved in differentiation	0.0115	C1QBP|CALR|RAC1|OPA1|S100A10|FLNA|ACTR2|DNM2|ILK|CDC42|IST1|DNM1L	0.0012	GO.0010770	0.19393022
5	3	GO Process	succinyl-CoA metabolic process	0.0115	OGDH|DLST|SUCLA2	0.0012	GO.0006104	0.19393022
283	19	GO Process	multicellular organismal homeostasis	0.0117	CTSH|NAPSA|ADD1|PRDX5|SOD1|TPP1|PRKACA|CYP4A11|AQP1|RAC1|PBLD|FH|GNAS|RBP4|CUBN|PRCP|AMPD3|GPX1|ACADVL	0.0012	GO.0048871	0.19318141
12	6	GO Component	tricarboxylic acid cycle enzyme complex	5.3E-05	OGDH|BCKDHA|BCKDHB|DLST|FH|OGDHL	7.45E-06	GO.0045239	0.42790143
26	5	GO Function	oxidoreductase activity, acting on the CH-CH group of donors, NAD or NADP as acceptor	0.012	DECR1|MECR|BDH2|AKR1C3|PTGR1	0.0017	GO.0016628	0.19208188

6	3	GO Function	primary amine oxidase activity	0.012	AOC3|MAOB|AOC1	0.0017	GO.0008131	0.19208188
6	3	GO Function	creatine kinase activity	0.012	CKB|CKMT2|CKMT1A	0.0017	GO.0004111	0.19208188
6	3	GO Function	acetyl-CoA C-acyltransferase activity	0.012	HADHB|ACAA1|HADHA	0.0017	GO.0003988	0.19208188
66	8	GO Process	cellular iron ion homeostasis	0.012	SKP1|LTF|SOD1|ATP6V1A|ATP6V0D1|ACO1|ISCU|TF	0.0012	GO.0006879	0.19208188
17	6	GO Component	proton-transporting two-sector ATPase complex, catalytic domain	0.00022	ATP6V1B1|ATP5B|ATP6V1A|ATP6V1B2|ATP5C1|ATP5A1	3.58E-05	GO.0033178	0.36575773
570	31	GO Process	cellular cation homeostasis	0.0121	MAPK1|SKP1|LTF|ATP5B|ATP6V0A1|CALB1|AFG3L2|SOD1|ATP6V1A|ATP6V0D2|ATP6V0D1|HSP90B1|LETM1|ATP6V0A4|ACO1|PRKACA|ISCU|CALR|ANXA6|STOML2|TGM2|ATP1B1|GSTO1|GOT1|CLIC4|TF|IMMT|SLC4A4|EPHX2|ATP1A1|PLCG2	0.0012	GO.0030003	0.19172146
157	13	GO Process	protein tetramerization	0.0122	DECR1|PFKL|DPYS|HPRT1|DCXR|SHMT1|DHRS4|ANXA2|S100A10|GPX3|CRYZ|FBP1|DNM1L	0.0013	GO.0051262	0.19136402
17	6	GO Component	pseudopodium	0.00022	MAPK1|ACTN4|CAPN2|MSN|CNP|ACTN1	3.58E-05	GO.0031143	0.36575773
353	22	GO Process	anion transmembrane transport	0.0123	SLC25A11|LRP2|VDAC1|CPT1A|SLC27A2|SLC25A4|SLC23A1|SLC7A8|SLC25A15|MPC1|ABCD3|SLC25A5|VDAC2|CLIC4|CLIC1|SLC3A2|SLC25A6|PCYOX1|SLC25A12|SLC4A4|VDAC3|SLC25A10	0.0013	GO.0098656	0.19100949
7	5	GO Component	MICOS complex	8.5E-05	CHCHD3|C19orf70|APOOL|APOO|IMMT	1.27E-05	GO.0061617	0.40700704
15	4	GO Function	dipeptidase activity	0.0124	PEPD|CNDP2|DPEP1|SCRN1	0.0018	GO.0016805	0.19065783
83	9	GO Process	maintenance of location in cell	0.0124	SKP1|SCIN|GAA|TLN1|HSPA5|EPB41L3|JUP|TSPO|HK1	0.0013	GO.0051651	0.19065783
37	6	GO Process	response to glucagon	0.0128	QDPR|PRKACA|CYC1|PCK1|GNAS|ASS1	0.0013	GO.0033762	0.189279
10	5	GO Component	dihydrolipoyl dehydrogenase complex	0.00027	OGDH|BCKDHA|BCKDHB|DLST|OGDHL	4.48E-05	GO.0045240	0.35686362
242	17	GO Process	negative regulation of endopeptidase activity	0.013	VTN|GAPDH|LTF|VIL1|PEBP1|AMBP|PRDX5|FABP1|PRDX3|AQP1|SERPINA5|ITIH2|DPEP1|AHSG|GPX1|SERPINA1|CRYAB	0.0014	GO.0010951	0.18860566
84	9	GO Process	actin-mediated cell contraction	0.0132	VIL1|GPD1L|ACTC1|PARVA|TPM4|ATP1A1|VIM|MYL6|TTN	0.0014	GO.0070252	0.18794261
650	34	GO Process	symbiont process	0.0132	RALA|MAPK1|C1QBP|GAPDH|LTF|APCS|CD81|AMBP|VDAC1|EIF4H|SLC25A4|PPIB|ANPEP|RAB1B|AQP1|TLN1|AP2A2|CTSB|ANXA2|RAC1|DPP4|KRT19|SLC25A5|YWHAB|SLC25A6|KRT18|PC|CDC42|SSB|GPX1|IST1|VIM|ITGB3|AP2B1	0.0014	GO.0044403	0.18794261
120	11	GO Process	nucleoside metabolic process	0.0132	AHCY|DPYS|HPRT1|PNP|OPA1|AHCYL1|CMPK1|AK3|NME2|AK4|AMPD3	0.0014	GO.0009116	0.18794261
159	13	GO Process	regulation of intrinsic apoptotic signaling pathway	0.0133	PPIF|ENO1|TRAP1|HSPB1|ERP29|SLC9A3R1|SOD1|P4HB|OPA1|VDAC2|GPX1|NDUFA13|DNM1L	0.0014	GO.2001242	0.18761484
98	9	KEGG Pathways	HIF-1 signaling pathway	0.0135	MAPK1|GAPDH|ENO1|PFKL|PDHB|PDHA1|TF|HK1|PLCG2	0.0036	hsa04066	0.18696662

11	5	GO Component	endoplasmic reticulum chaperone complex	0.00038	HSP90B1|PPIB|HSPA5|P4HB|PDIA6	6.37E-05	GO.0034663	0.34202164
52	7	GO Process	IRE1-mediated unfolded protein response	0.0136	ADD1|ATP6V0D1|TPP1|TLN1|HSPA5|PDIA6|ACADVL	0.0014	GO.0036498	0.18664611
25	5	GO Process	positive regulation of lamellipodium organization	0.014	VIL1|RAC1|DNM2|CORO1B|CDC42	0.0015	GO.1902745	0.1853872
14	4	GO Process	establishment of protein localization to mitochondrial membrane	0.014	HSP90AA1|SAMM50|AGK|NDUFA13	0.0015	GO.0090151	0.1853872
14	4	GO Process	glutathione biosynthetic process	0.014	CNDP2|HAGH|GGT5|GGT1	0.0015	GO.0006750	0.1853872
63	7	KEGG Pathways	Shigellosis	0.0141	VCL|MAPK1|PFN1|RAC1|CDC42|ARPC1B|ACTG1	0.0038	hsa05131	0.18507809
38	6	GO Process	substrate adhesion-dependent cell spreading	0.0141	LAMA5|LAMC1|PARVA|RAC1|ILK|ITGB3	0.0015	GO.0034446	0.18507809
11	5	GO Component	mitochondrial proton-transporting ATP synthase complex, coupling factor F(o)	0.00038	ATP5J2|ATP5L|ATP5H|ATP5I|ATP5F1	6.37E-05	GO.0000276	0.34202164
16	5	GO Component	vacuolar proton-transporting V-type ATPase complex	0.0014	ATP6V1B1|ATP6V0A1|ATP6V0D2|ATP6V0D1|ATP6V0A4	0.00026	GO.0016471	0.2853872
18	5	GO Component	pore complex	0.002	PPIF|VDAC1|VDAC2|TOMM40|VDAC3	0.00042	GO.0046930	0.269897
41	6	GO Function	4 iron, 4 sulfur cluster binding	0.0143	ACO2|ACO1|ISCU|NDUFV1|NDUFS1|ETFDH	0.0021	GO.0051539	0.1844664
1052	49	GO Process	response to abiotic stimulus	0.0143	DCN|AHCY|REEP6|ACTN4|SORD|SOD1|ATP6V1A|ATP6V0D1|CAPN2|FABP1|HSP90B1|PRKACA|AQP1|PCK1|HSPA5|P4HB|RAB11B|HSP90AA1|PKLR|COL18A1|RAC1|DPP4|LONP1|MT-CO1|OPA1|MT-CO2|ATP1B1|GOT1|POSTN|CD9|FECH|DNM2|JUP|TSPO|ABAT|ALAD|RDH13|HIGD1A|NDRG1|GPX1|MME|CRYAB|ACADVL|PHB2|ATP1A1|DNM1L|ACTG1|TTN|FMO1	0.0015	GO.0009628	0.1844664
245	17	GO Process	cellular response to peptide hormone stimulus	0.0144	ATP6V1B1|ATP6V0A1|ATP6V1A|ATP6V1B2|ATP6V0D2|ATP6V0D1|YWHAG|ATP6V0A4|PRKACA|PCK1|FBN1|PKLR|AHCYL1|GOT1|GNAS|ASS1|GSTP1	0.0015	GO.0071375	0.18416375
23	5	GO Component	microvillus membrane	0.0047	SLC9A3R1|MSN|DPEP1|PDZK1|ITGB3	0.0011	GO.0031528	0.23279021
81	8	KEGG Pathways	ECM-receptor interaction	0.0145	VTN|LAMA5|LAMC1|COL1A2|COL4A2|HSPG2|COL4A1|ITGB3	0.004	hsa04512	0.1838632
100	9	KEGG Pathways	Glucagon signaling pathway	0.0147	PCK2|CPT1A|PFKL|PDHB|PRKACA|PCK1|GNAS|PDHA1|FBP1	0.0041	hsa04922	0.18326827

224	16	GO Process	regulation of ion transmembrane transporter activity	0.0148	PPIF|ACTN4|SLC9A3R1|GPD1L|PRKACA|GBAS|EHD3|ATP1B1|GSTO1|AHCYL1|GLRX|DNM2|FHL1|FKBP1A|PDZK1|PLCG2	0.0016	GO.0032412	0.18297383
16	4	GO Function	quinone binding	0.015	SQRDL|AOC3|AOC1|ETFDH	0.0022	GO.0048038	0.18239087
16	4	GO Function	transferase activity, transferring amino-acyl groups	0.015	F13A1|TGM2|GGT5|GGT1	0.0022	GO.0016755	0.18239087
16	4	GO Function	oxidoreductase activity, acting on the CH-NH2 group of donors, oxygen as acceptor	0.015	PNPO|AOC3|MAOB|AOC1	0.0022	GO.0016641	0.18239087
1055	49	GO Process	epithelium development	0.015	RALA|VCL|MAPK1|MTHFD1|CTSH|PFN1|DNPH1|VIL1|LAMA5|LGALS3|GRSF1|GSTM3|SLC9A3R1|LRP2|VDAC1|CALB1|CPT1A|RHCG|SOD1|BDH2|TPP1|PRKACA|DAB2|GSTA1|CTSB|COL18A1|MSN|KRT19|TGM2|PHGDH|FLNA|GNAS|CLIC4|COL4A1|AKR1C3|KRT18|JUP|UMOD|ILK|CDC42|RDX|TST|ACTA2|GPX1|GSTA2|TAGLN|ACADVL|PHB2|VIM	0.0016	GO.0060429	0.18239087
291	19	GO Process	muscle system process	0.0151	VCL|SOD1|GPD1L|ACTC1|GAA|TLN1|TPM4|ATP1B1|SNTB1|MYH11|GATM|ACTA2|CKMT2|CRYAB|ATP1A1|VIM|MYL6|TTN|PDLIM5	0.0016	GO.0003012	0.18210231
2197	88	GO Function	enzyme binding	0.0152	RALA|UQCRC1|VCL|MAPK1|PFN1|C1QBP|CTSC|MLEC|EHHADH|ENO1|GOT2|TRAP1|TUBA4A|HSPB1|GSTM3|ADAM10|LRPPRC|PEBP1|COTL1|CHCHD3|SLC9A3R1|ATP6V0A1|VDAC1|SLC27A2|PFKL|SOD1|IQGAP2|ERLIN2|UCHL1|HSPA9|COL1A2|PRDX3|CKB|CCT2|HSP90B1|PPIB|HINT1|YWHAG|ATP6V0A4|PRKACA|CALR|HSPA5|AP2A2|P4HB|SERPINA5|HSP90AA1|CYB5A|RPS2|PRDX6|PFKM|ANXA2|UGT1A9|RAC1|STOML2|MSN|DPP4|LONP1|OPA1|ATP1B1|FLNA|SLC25A5|YWHAB|ALDOB|PCCA|FLOT1|GLDC|DNM2|JUP|FLOT2|MVP|ABAT|ILK|GSTP1|CDC42|RDX|ACTA2|DDC|NDRG1|SERPINA1|RAP1GAP|ATP1A1|DNM1L|NPC2|ITGB3|ACTG1|TTN|PHB|PDLIM5	0.0023	GO.0019899	0.18181564
57	7	GO Function	amino acid binding	0.0152	GOT2|DPYS|DDAH1|SHMT1|ASS1|GLDC|DDC	0.0023	GO.0016597	0.18181564
57	7	GO Function	metalloexopeptidase activity	0.0152	LTA4H|PEPD|ENPEP|RNPEP|ANPEP|XPNPEP2|DPEP1	0.0023	GO.0008235	0.18181564
33	5	KEGG Pathways	Prion diseases	0.0153	MAPK1|LAMC1|SOD1|PRKACA|HSPA5	0.0044	hsa05020	0.18153086
48	6	KEGG Pathways	Cholesterol metabolism	0.0153	LRP2|VDAC1|VDAC2|TSPO|VDAC3|NPC2	0.0043	hsa04979	0.18153086
8	4	GO Component	pyruvate dehydrogenase complex	0.0014	PDHX|DLAT|PDHB|PDHA1	0.00027	GO.0045254	0.2853872
7	3	GO Function	thyroid hormone binding	0.0156	CRYM|CTSH|TTR	0.0024	GO.0070324	0.18068754
7	3	GO Function	alditol:NADP+ 1-oxidoreductase activity	0.0156	AKR7A2|AKR1A1|AKR1C3	0.0024	GO.0004032	0.18068754
7	3	GO Function	retinal dehydrogenase activity	0.0156	ALDH8A1|ALDH1A1|AKR1C3	0.0024	GO.0001758	0.18068754
6	3	GO Process	purine nucleobase transmembrane transport	0.016	SLC25A4|SLC25A5|SLC25A6	0.0017	GO.1904823	0.179588

6	3	GO Process	sulfide oxidation, using sulfide:quinone oxidoreductase	0.016	SQRDL|SUOX|SLC25A10	0.0017	GO.0070221	0.179588
483	27	GO Process	regulation of body fluid levels	0.016	MAPK1|F9|C1QBP|VTN|GOT2|EHD2|F13A1|COL1A2|PRKACA|CYP4A11|AQP1|EHD3|SERPINA5|ANXA2|RAC1|MT-CO2|FLNA|GNAS|HBD|AK3|CD9|PRCP|ABAT|CDC42|SERPINA1|ITGB3|PLCG2	0.0017	GO.0050878	0.179588
6	3	GO Process	nucleobase catabolic process	0.016	GDA|DPYS|ALDH6A1	0.0017	GO.0046113	0.179588
6	3	GO Process	positive regulation of dopamine metabolic process	0.016	HPRT1|MAOB|ABAT	0.0017	GO.0045964	0.179588
6	3	GO Process	alditol catabolic process	0.016	SORD|GPD2|GK	0.0017	GO.0019405	0.179588
6	3	GO Process	protein processing involved in protein targeting to mitochondrion	0.016	PMPCB|UQCRC2|PMPCA	0.0017	GO.0006627	0.179588
399	23	GO Function	endopeptidase activity	0.0164	UQCRC1|F9|CTSH|CTSC|LTF|CTSD|PMPCB|NAPSA|TINAG|ADAM10|UQCRC2|AFG3L2|UCHL1|CAPN2|TPP1|PDIA3|TPSAB1|CTSB|DPP4|LONP1|PMPCA|PITRM1|MME	0.0026	GO.0004175	0.17851562
43	6	GO Function	aminoacyl-tRNA ligase activity	0.0167	CARS2|DARS2|IARS2|VARS|LARS2|SARS2	0.0026	GO.0004812	0.17772835
15	4	GO Process	proton-transporting two-sector ATPase complex assembly	0.0167	ATP6V0A1|ATP6V0A4|ALDOB|ATPAF2	0.0018	GO.0070071	0.17772835
953	45	GO Process	cytoskeleton organization	0.0167	RALA|MYH9|PFN1|GAPDH|DSTN|TUBA4A|VIL1|ACTN4|LAMA5|SLC9A3R1|EHD2|ADD1|SOD1|IQGAP2|ACTC1|SCIN|HSP90B1|TUBA1C|DPYSL2|AQP1|TLN1|CALR|PARVA|PLS1|TUBB4B|EPB41L3|TPM4|RAC1|MSN|MYH10|KRT19|FLNA|ACTR2|KRT18|CNP|CORO1B|JUP|ACTN1|MYH11|CDC42|ARPC1B|CRYAB|VIM|ACTG1|TTN	0.0018	GO.0007010	0.17772835
66	7	KEGG Pathways	Antigen processing and presentation	0.0168	CANX|PDIA3|CALR|HSPA5|HSP90AA1|CTSB|IFI30	0.0049	hsa04612	0.17746907
185	14	GO Process	phagocytosis	0.0168	MAPK1|MYH9|HSP90AA1|RAC1|TGM2|ACTR2|TXNDC5|DNM2|CDC42|PDIA6|ARPC1B|ITGB3|ACTG1|PLCG2	0.0018	GO.0006909	0.17746907
10	4	GO Component	proton-transporting V-type ATPase, V0 domain	0.0025	ATP6V0A1|ATP6V0D2|ATP6V0D1|ATP6V0A4	0.00053	GO.0033179	0.260206
13	4	GO Component	uropod	0.0051	MYH9|MSN|FLOT1|FLOT2	0.0012	GO.0001931	0.22924298
71	8	GO Process	regulation of mitochondrial membrane permeability	0.0171	PPIF|SLC25A4|YWHAG|RHOT2|SLC25A5|YWHAB|SLC25A6|CNP	0.0019	GO.0046902	0.17670039
511	28	GO Process	regulation of hormone levels	0.0172	OXCT1|CRYM|TTR|ENPEP|ALDH8A1|CPT1A|PFKL|SLC25A4|ALDH1A1|UGT2B7|BCAT2|DHRS4|RAB11B|PFKM|UGT1A9|ALDH9A1|DPP4|GNAS|RBP4|SCP2|SLC25A5|AKR1C3|SLC25A6|TSPO|ABAT|RDH13|MME|ATP1A1	0.0019	GO.0010817	0.17644716
229	16	GO Process	regulation of endocytosis	0.0176	VTN|ACTN4|LGALS3|DAB2|CALR|ANXA2|RAC1|C4BPA|RAB14|FLOT1|DNM2|TF|AHSG|DNM1L|PLCG2|AP2B1	0.002	GO.0030100	0.17544873

414	24	GO Process	organic anion transport	0.0177	SLC25A11|GOT2|SLC9A3R1|LRP2|CPT1A|SLC27A2|SLC25A4|FABP1|CA4|SLC23A1|AQP1|SLC7A8|SLC25A15|MPC1|ABCD3|SCP2|SLC25A5|SLC3A2|SLC25A6|SLC25A12|SLC4A4|ACSL1|SLC25A10|NPC2	0.002	GO.0015711	0.17520267
208	15	GO Process	response to nutrient	0.018	OXCT1|AHCY|NNT|SOD1|HSP90B1|PKLR|OPA1|ASS1|POSTN|AKR1C3|TSPO|GSTP1|ALAD|ACSL1|COX4I1	0.002	GO.0007584	0.17447275
19	4	GO Component	complex of collagen trimers	0.0143	LUM|COL1A2|COL4A2|COL4A1	0.0038	GO.0098644	0.1844664
252	17	GO Process	positive regulation of ion transport	0.0182	ACTN4|LGALS3|GPD1L|CYP4A11|GBAS|EHD3|ATP1B1|GSTO1|AHCYL1|FLNA|GLRX|DNM2|FHL1|TSPO|ABAT|PDZK1|PLCG2	0.002	GO.0043270	0.17399286
19	4	GO Component	endocytic vesicle lumen	0.0143	LTF|HSP90B1|CALR|HSP90AA1	0.0038	GO.0071682	0.1844664
19	4	GO Component	costamere	0.0143	VCL|PLEC|KRT19|ILK	0.0038	GO.0043034	0.1844664
3	3	GO Component	glycerol-3-phosphate dehydrogenase complex	0.0021	GPD1L|GPD1|GPD2	0.00044	GO.0009331	0.26777807
56	7	GO Process	response to cadmium ion	0.0189	MAPK1|SORD|SOD1|CYB5A|GOT1|AKR1C3|ALAD	0.0021	GO.0046686	0.17235382
41	6	GO Process	icosanoid biosynthetic process	0.0189	LTA4H|PTGES2|MGST3|AKR1C3|GGT5|GGT1	0.0021	GO.0046456	0.17235382
41	6	GO Process	polyol biosynthetic process	0.0189	SPR|QDPR|PCK1|GOT1|SCP2|PLCG2	0.0021	GO.0046173	0.17235382
73	8	GO Process	Fc-gamma receptor signaling pathway involved in phagocytosis	0.0196	MAPK1|HSP90AA1|RAC1|ACTR2|CDC42|ARPC1B|ACTG1|PLCG2	0.0022	GO.0038096	0.17077439
189	14	GO Process	regulation of cell-substrate adhesion	0.0196	LGALS1|C1QBP|VTN|ACTN4|CALR|RAC1|S100A10|FLNA|RSU1|POSTN|DNM2|JUP|ILK|CDC42	0.0022	GO.0010810	0.17077439
16	4	GO Process	mitochondrial fusion	0.0197	CHCHD3|AFG3L2|STOML2|OPA1	0.0022	GO.0008053	0.17055338
148	12	GO Process	regulation of mitochondrion organization	0.0198	DCN|PPIF|VDAC1|YWHAG|VAT1|STOML2|OPA1|SLC25A5|YWHAB|TSPO|GPX1|DNM1L	0.0023	GO.0010821	0.17033348

<table>
<tr><td>4754</td><td>171</td><td>GO Process</td><td>cellular aromatic compound metabolic process</td><td>0.0198</td><td>NDUFB4|UQCRC1|NANS|MAPK1|RTCB|GSTZ1|MTHFD1|AHCY|CRYM|OGDH|PNPO|C1QBP|PDHX|GAPDH|DNPH1|LTF|ATP6V1B1|SPR|ENO1|AKR7A2|GDA|GOT2|PMPCB|NDUFA2|NDUFA10|LGALS3|GRSF1|HRSP12|GSTM3|CARS2|GGH|LRPPRC|ATP5B|CHCHD3|ATP6V0A1|NNT|SDHA|AMBP|PRDX5|REXO2|NDUFA9|UQCRC2|PFKL|ATP6V1A|ALDH1L1|MUT|ATP6V1B2|LACTB2|DPYS|NDUFB9|HNMT|DLAT|OXSM|ACAD8|QDPR|GPD1L|HGD|HPD|ATP5O|FTCD|ATP5J2|BDH2|HPRT1|AMN|NDUFB8|ATP5L|GPD1|ATP5H|SLC23A1|DCXR|HINT1|ATP5I|PDHB|CYCS|ATP6V0A4|GPD2|DPYSL2|UQCRH|PRKACA|AQP1|GBAS|ACSS1|CYC1|PIPOX|COX5A|SHMT1|UGDH|NDUFV1|MDH2|ACSM2B|NDUFA12|IDH2|UQCR10|MRPL12|HSP90AA1|DLST|PKLR|RPS2|PPA2|PFKM|STOML2|ATP5C1|COL4A2|LONP1|MPC1|MT-CO1|PNP|OPA1|MT-CO2|DARS2|IBA57|IARS2|ATP1B1|LHPP|ATP5F1|AHCYL1|FLNA|GNAS|CMPK1|OGDHL|ALDOB|VARS|PSAT1|CUBN|MAOB|SUCLA2|ACOT9|PDHA1|GLRX|AKR1C3|DUT|PFKP|AK3|FECH|NME2|CNP|AK4|QPRT|TSPO|AMPD3|ATP5A1|FAH|ALAD|SSB|IDH1|TKT|NDUFS1|PNPT1|NAPRT|ACSF2|HK1|DDC|GPX1|LARS2|SSBP1|ABHD14B|ACSL1|COX7C|EPHX2|PHB2|PAH|ALDH6A1|COX4I1|ACSM2A|RBM8A|GALK1|SARS2|PHB|EPHX1|FMO1|GLYAT</td><td>0.0023</td><td>GO.0006725</td><td>0.17033348</td></tr>
<tr><td>2524</td><td>99</td><td>GO Process</td><td>regulation of localization</td><td>0.02</td><td>RALA|DCN|OXCT1|VCL|MAPK1|CTSH|PPIF|PFN1|C1QBP|VTN|GAPDH|REEP6|VIL1|HSPB1|ACTN4|LAMA5|LGALS3|ADAM10|ERP29|ATP5B|SLC9A3R1|EHD2|NAPA|CD81|CPT1A|PFKL|CCT5|SLC25A4|GPD1L|SCIN|CCT2|LETM1|YWHAG|PRKACA|CYP4A11|AQP1|GBAS|DAB2|CALR|HSPA5|EHD3|IDH2|RAB11B|HSP90AA1|PLS1|GNAI1|PFKM|ANXA2|COL18A1|RAC1|PBLD|MSN|MYH10|DPP4|OPA1|C4BPA|ATP1B1|GSTO1|AHCYL1|FLNA|MYO6|GNAS|RBP4|SCP2|SLC25A5|YWHAB|RAB14|CLIC4|CLIC1|FLOT1|MAOB|POSTN|GLRX|SLC25A6|CD9|DNM2|DPEP1|PRCP|CORO1B|JUP|FHL1|ACTN1|TSPO|ABAT|ILK|GSTP1|ATP5A1|FKBP1A|RDX|TF|AHSG|PDZK1|CRYAB|ATP1A1|DNM1L|ITGB3|TTN|PLCG2|AP2B1</td><td>0.0023</td><td>GO.0032879</td><td>0.169897</td></tr>
<tr><td>724</td><td>36</td><td>GO Process</td><td>interspecies interaction between organisms</td><td>0.0201</td><td>RALA|MAPK1|C1QBP|GAPDH|LTF|LGALS3|APCS|LYZ|CD81|AMBP|VDAC1|EIF4H|SLC25A4|PPIB|ANPEP|RAB1B|AQP1|TLN1|AP2A2|CTSB|ANXA2|RAC1|DPP4|KRT19|SLC25A5|YWHAB|SLC25A6|KRT18|PC|CDC42|SSB|GPX1|IST1|VIM|ITGB3|AP2B1</td><td>0.0023</td><td>GO.0044419</td><td>0.16968039</td></tr>
<tr><td>91</td><td>9</td><td>GO Process</td><td>positive regulation of ion transmembrane transporter activity</td><td>0.0201</td><td>ACTN4|GBAS|EHD3|ATP1B1|GSTO1|GLRX|DNM2|PDZK1|PLCG2</td><td>0.0023</td><td>GO.0032414</td><td>0.16968039</td></tr>
<tr><td>28</td><td>5</td><td>GO Process</td><td>response to copper ion</td><td>0.0202</td><td>SORD|SOD1|AQP1|MT-CO1|AOC1</td><td>0.0023</td><td>GO.0046688</td><td>0.16946486</td></tr>
<tr><td>28</td><td>5</td><td>GO Process</td><td>nucleobase-containing small molecule interconversion</td><td>0.0202</td><td>CMPK1|GLRX|DUT|NME2|AK4</td><td>0.0023</td><td>GO.0015949</td><td>0.16946486</td></tr>
<tr><td>8</td><td>3</td><td>GO Function</td><td>ligase activity, forming carbon-carbon bonds</td><td>0.0203</td><td>PCCA|PC|PCCB</td><td>0.0032</td><td>GO.0016885</td><td>0.1692504</td></tr>
<tr><td>8</td><td>3</td><td>GO Function</td><td>hydroxymethyl-, formyl- and related transferase activity</td><td>0.0203</td><td>ALDH1L1|FTCD|SHMT1</td><td>0.0032</td><td>GO.0016742</td><td>0.1692504</td></tr>
<tr><td>8</td><td>3</td><td>GO Function</td><td>malate dehydrogenase activity</td><td>0.0203</td><td>MDH2|PHGDH|ME3</td><td>0.0032</td><td>GO.0016615</td><td>0.1692504</td></tr>
</table>

45	6	GO Function	structural constituent of muscle	0.0203	PLEC|TPM4|KRT19|MYH11|MYL6|TTN	0.0032	GO.0008307	0.1692504
8	3	GO Function	ubiquinol-cytochrome-c reductase activity	0.0203	UQCRC1|UQCRH|UQCR10	0.0032	GO.0008121	0.1692504
8	3	GO Function	acyl-CoA ligase activity	0.0203	ACSM2B|ACSF2|ACSM2A	0.0032	GO.0003996	0.1692504
8	3	GO Function	complement component C1q binding	0.0203	C1QBP|APCS|CALR	0.0032	GO.0001849	0.1692504
831	40	GO Process	anatomical structure formation involved in morphogenesis	0.0203	RALA|MAPK1|MYH9|MTHFD1|PFN1|VTN|LAMA5|ATP5B|LRP2|ENPEP|CALB1|ACTC1|CAPN2|ANPEP|PRKACA|PARVA|EPB41L3|ANXA2|COL18A1|MYH10|COL4A2|KRT19|TGM2|GNAS|RBP4|CLIC4|HSPG2|COL4A1|CD9|PRCP|ACTN1|MYH11|ILK|FKBP1A|RDH13|GPX1|TGFBI|ITGB3|ACTG1|TTN	0.0023	GO.0048646	0.1692504
7	3	GO Process	fructose 1,6-bisphosphate metabolic process	0.0209	PFKL|ALDOB|FBP1	0.0024	GO.0030388	0.16798537
7	3	GO Process	response to selenium ion	0.0209	MAOB|ALAD|GPX1	0.0024	GO.0010269	0.16798537
373	22	GO Process	blood circulation	0.0209	ENPEP|SOD1|GPD1L|DDAH1|ACTC1|RNPEP|ADH5|COL1A2|ANPEP|GAA|CYP4A11|CYB5R3|ATP1B1|POSTN|PRCP|ABAT|ACTA2|GPX1|MME|EPHX2|ATP1A1|TTN	0.0024	GO.0008015	0.16798537
7	3	GO Process	biotin metabolic process	0.0209	PCCA|PC|PCCB	0.0024	GO.0006768	0.16798537
7	3	GO Process	leucine metabolic process	0.0209	BCAT2|AUH|IVD	0.0024	GO.0006551	0.16798537
7	3	GO Process	dynamin family protein polymerization involved in mitochondrial fission	0.0209	OPA1|DNM2|DNM1L	0.0024	GO.0003374	0.16798537
7	3	GO Process	citrulline metabolic process	0.0209	DDAH1|ALDH18A1|ASS1	0.0024	GO.0000052	0.16798537
649	33	GO Process	epithelial cell differentiation	0.0211	DNPH1|VIL1|LAMA5|LGALS3|GSTM3|SLC9A3R1|VDAC1|CPT1A|RHCG|SOD1|BDH2|TPP1|DAB2|GSTA1|CTSB|COL18A1|MSN|KRT19|FLNA|CLIC4|COL4A1|AKR1C3|KRT18|JUP|CDC42|RDX|TST|ACTA2|GPX1|GSTA2|TAGLN|ACADVL|VIM	0.0025	GO.0030855	0.16757175
92	9	GO Process	cellular component assembly involved in morphogenesis	0.0211	ACTC1|EPB41L3|MYH10|KRT19|CD9|MYH11|ILK|ACTG1|TTN	0.0025	GO.0010927	0.16757175
547	29	GO Process	response to wounding	0.0215	DCN|MAPK1|LGALS1|F9|C1QBP|EHD2|F13A1|SOD1|COL1A2|PRKACA|EHD3|SERPINA5|RAC1|MYH10|FLNA|GNAS|POSTN|HBD|AK3|CD9|PRCP|CORO1B|TSPO|CDC42|GPX1|SERPINA1|PHB2|ITGB3|PLCG2	0.0025	GO.0009611	0.16675615
75	8	GO Process	interaction with symbiont	0.022	GAPDH|LTF|APCS|PPIB|AQP1|ANXA2|PC|GPX1	0.0026	GO.0051702	0.16575773
43	6	GO Process	endothelial cell development	0.0224	GSTM3|COL18A1|MSN|CLIC4|RDX|GPX1	0.0026	GO.0001885	0.1649752
29	5	GO Process	actin nucleation	0.0225	VIL1|IQGAP2|SCIN|ACTR2|ARPC1B	0.0027	GO.0045010	0.16478175
19	4	GO Function	adrenergic receptor binding	0.023	C1QBP|SLC9A3R1|UCHL1|GNAS	0.0038	GO.0031690	0.16382722

71	7	KEGG Pathways	Bile secretion	0.0236	PRKACA|AQP1|ATP1B1|GNAS|SLC4A4|ATP1A1|EPHX1	0.007	hsa04976	0.1627088
23	4	KEGG Pathways	Renin-angiotensin system	0.0236	ENPEP|ANPEP|PRCP|MME	0.0069	hsa04614	0.1627088
71	7	KEGG Pathways	Adherens junction	0.0236	VCL|MAPK1|ACTN4|RAC1|ACTN1|CDC42|ACTG1	0.007	hsa04520	0.1627088
840	40	GO Process	regulation of catabolic process	0.0237	DCN|CTSC|GAPDH|ATP6V1B1|HSPB1|LRPPRC|CD81|LRP2|PSMD3|ATP6V0A1|NNT|VDAC1|CPT1A|ATP6V1A|ATP6V1B2|UCHL1|ATP6V0D2|ATP6V0D1|FABP1|GPD1|PRKACA|RAB1B|DAB2|HSP90AA1|ANXA2|MSN|C4BPA|FLNA|YWHAB|TSPO|RDX|ALAD|IDH1|PNPT1|GPX1|FBP1|NDUFA13|VIM|DNM1L|PHB	0.0028	GO.0009894	0.16252517
4	3	GO Component	terminal web	0.0034	VCL|PLS1|KRT19	0.00075	GO.1990357	0.24685211
6	3	GO Component	proton-transporting ATP synthase complex, catalytic core F(1)	0.007	ATP5B|ATP5C1|ATP5A1	0.0017	GO.0045261	0.2154902
515	26	KEGG Pathways	Pathways in cancer	0.0243	RALA|MAPK1|SKP1|LAMA5|GSTM3|LAMC1|HSP90B1|CYCS|PRKACA|HSP90AA1|GSTA1|GNAI1|RAC1|COL4A2|FH|MGST3|GSTO1|GNAS|COL4A1|JUP|MGST1|GSTP1|CDC42|TXNRD2|GSTA2|PLCG2	0.0074	hsa05200	0.16143937
72	7	KEGG Pathways	Bacterial invasion of epithelial cells	0.0243	VCL|RAC1|DNM2|ILK|CDC42|ARPC1B|ACTG1	0.0075	hsa05100	0.16143937
153	12	GO Process	cellular response to insulin stimulus	0.0245	ATP6V1B1|ATP6V0A1|ATP6V1A|ATP6V1B2|ATP6V0D2|ATP6V0D1|YWHAG|ATP6V0A4|PCK1|PKLR|GOT1|GSTP1	0.0029	GO.0032869	0.16108339
100	9	GO Function	chaperone binding	0.0246	OGDH|CTSC|ERP29|LRP2|SOD1|TIMM44|CALR|HSPA5|ATP1A1	0.0041	GO.0051087	0.16090649
9	3	GO Function	NADP-retinol dehydrogenase activity	0.0252	DHRS4|AKR1C3|RDH13	0.0042	GO.0052650	0.15985995
9	3	GO Function	aldehyde-lyase activity	0.0252	SHMT1|HOGA1|ALDOB	0.0042	GO.0016832	0.15985995
9	3	GO Function	oxidoreductase activity, acting on a sulfur group of donors, oxygen as acceptor	0.0252	SUOX|PCYOX1|SELENBP1	0.0042	GO.0016670	0.15985995
9	3	GO Function	C4-dicarboxylate transmembrane transporter activity	0.0252	SLC25A11|SLC25A12|SLC25A10	0.0042	GO.0015556	0.15985995
82	8	GO Function	antiporter activity	0.0252	SLC25A11|SLC25A4|LETM1|SLC7A8|SLC25A5|SLC3A2|SLC25A6|SLC25A10	0.0043	GO.0015297	0.15985995
77	8	GO Process	regulation of reactive oxygen species biosynthetic process	0.0253	AGXT2|TRAP1|DDAH1|HSP90AA1|MT-CO2|ASS1|DNM2|TSPO	0.003	GO.1903426	0.15968795
30	5	GO Process	regulation of oxidative stress-induced intrinsic apoptotic signaling pathway	0.0253	TRAP1|HSPB1|SOD1|P4HB|GPX1	0.003	GO.1902175	0.15968795

1035	47	GO Process	response to cytokine	0.0253	RALA|DCN|MAPK1|GAPDH|SKP1|CANX|LAMA5|MRPL15|ADAM10|F13A1|SOD1|HSPA9|COL1A2|HSP90B1|GPD1|PRKACA|SHMT1|PCK1|BCAT2|HSPA5|BGN|P4HB|HSP90AA1|ANXA2|MSN|MPC1|GSTO1|SLC25A5|ASS1|ASPN|ACTR2|POSTN|PFKP|GLDC|KRT18|ILK|CDC42|IFI30|ALAD|PNPT1|MME|GSTA2|ACSL1|NDUFA13|VIM|ACTG1|PHB	0.003	GO.0034097	0.15968795
555	29	GO Process	metal ion homeostasis	0.0256	SKP1|LTF|ATP6V1B1|CALB1|AFG3L2|SOD1|ATP6V1A|ATP6V0D1|BDH2|HSP90B1|LETM1|ACO1|PRKACA|ISCU|CYP4A11|SLC7A8|CALR|ANXA6|STOML2|TGM2|ATP1B1|GSTO1|SFXN2|GOT1|TF|IMMT|EPHX2|ATP1A1|PLCG2	0.0031	GO.0055065	0.159176
20	4	GO Function	complement binding	0.0257	C1QBP|APCS|CALR|PHB	0.0045	GO.0001848	0.15900669
405	23	GO Process	protein localization to membrane	0.0257	LAMA5|SLC9A3R1|EHD2|AMN|EHD3|RAB11B|HSP90AA1|RPS2|EPB41L3|SAMM50|ANXA2|AGK|PIGR|ATP1B1|S100A10|FLNA|SCP2|FLOT1|KRT18|JUP|FLOT2|RDX|NDUFA13	0.0031	GO.0072657	0.15900669
1516	64	GO Process	regulation of programmed cell death	0.0257	LGALS1|CTSH|PPIF|C1QBP|CTSC|GAPDH|LTF|ENO1|TRAP1|VIL1|HSPB1|ACTN4|LGALS3|ERP29|SLC9A3R1|NNT|PRDX5|SOD1|NAT8|HIGD2A|SLC25A4|ACTC1|FABP1|SCIN|HSPA9|PRDX3|HSP90B1|PDIA3|YWHAG|CYCS|AQP1|DAB2|CALR|HSPA5|P4HB|CTSB|GPX4|COL18A1|OPA1|TGM2|FLNA|SLC25A5|VDAC2|YWHAB|TXNDC5|AKR1C3|KRT18|DNM2|DPEP1|NME2|ACTN1|TSPO|ILK|GSTP1|HIGD1A|NDRG1|GPX1|NDUFA13|CRYAB|PHB2|DNM1L|SIRT5|PHB|PLCG2	0.0031	GO.0043067	0.15900669
112	9	KEGG Pathways	Leukocyte transendothelial migration	0.026	VCL|ACTN4|GNAI1|RAC1|MSN|ACTN1|CDC42|ACTG1|PLCG2	0.0081	hsa04670	0.15850267
505	27	GO Process	cellular response to organic cyclic compound	0.026	MAPK1|LGALS1|SOD1|HSP90B1|GPD1|PRKACA|AQP1|SHMT1|PCK1|CALR|HSPA5|PKLR|GNAI1|MSN|GNAS|ASS1|FLOT1|ACTR2|AKR1C3|FECH|DNM2|JUP|GSTP1|AOC1|ATP1A1|DNM1L|PHB	0.0031	GO.0071407	0.15850267
6	3	GO Component	oxoglutarate dehydrogenase complex	0.007	OGDH|DLST|OGDHL	0.0017	GO.0045252	0.2154902
9	3	GO Component	proton-transporting V-type ATPase, V1 domain	0.0154	ATP6V1B1|ATP6V1A|ATP6V1B2	0.0042	GO.0033180	0.18124793
10	3	GO Component	fascia adherens	0.0189	VCL|JUP|ACTN1	0.0054	GO.0005916	0.17235382
2	2	GO Component	6-phosphofructokinase complex	0.0171	PFKL|PFKM	0.0047	GO.0005945	0.17670039
155	12	GO Process	drug transport	0.0265	LRP2|SLC25A4|AMN|AQP1|SLC25A15|SLC25A5|CUBN|HBD|SLC25A6|SLC25A12|PDZK1|SLC25A10	0.0032	GO.0015893	0.15767541
155	12	GO Process	tissue homeostasis	0.0265	CTSH|NAPSA|ADD1|PRDX5|SOD1|TPP1|RAC1|PBLD|FH|GNAS|RBP4|CUBN	0.0032	GO.0001894	0.15767541
8	3	GO Process	positive regulation of early endosome to late endosome transport	0.0267	DAB2|MSN|RDX	0.0032	GO.2000643	0.15734887
18	4	GO Process	regulation of early endosome to late endosome transport	0.0267	MAPK1|DAB2|MSN|RDX	0.0032	GO.2000641	0.15734887
18	4	GO Process	transepithelial transport	0.0267	RHCG|SLC23A1|AQP1|AHCYL1	0.0032	GO.0070633	0.15734887
8	3	GO Process	actin filament severing	0.0267	DSTN|VIL1|SCIN	0.0032	GO.0051014	0.15734887

18	4	GO Process	mitochondrion transport along microtubule	0.0267	LRPPRC|UCHL1|RHOT2|OPA1	0.0032	GO.0047497	0.15734887
8	3	GO Process	purine ribonucleoside catabolic process	0.0267	AHCY|HPRT1|PNP	0.0032	GO.0046130	0.15734887
18	4	GO Process	nucleobase biosynthetic process	0.0267	MTHFD1|HPRT1|SHMT1|CMPK1	0.0032	GO.0046112	0.15734887
18	4	GO Process	dicarboxylic acid catabolic process	0.0267	GOT2|ALDH1L1|GOT1|QPRT	0.0032	GO.0043649	0.15734887
18	4	GO Process	leukotriene biosynthetic process	0.0267	LTA4H|MGST3|GGT5|GGT1	0.0032	GO.0019370	0.15734887
8	3	GO Process	skeletal myofibril assembly	0.0267	ACTC1|MYH11|TTN	0.0032	GO.0014866	0.15734887
18	4	GO Process	pyrimidine nucleobase metabolic process	0.0267	MAPK1|DPYS|CMPK1|ALDH6A1	0.0032	GO.0006206	0.15734887
8	3	GO Process	malate metabolic process	0.0267	MDH2|FH|ME3	0.0032	GO.0006108	0.15734887
2	2	GO Function	oxidoreductase activity, acting on the CH-NH group of donors, flavin as acceptor	0.0269	DMGDH|SARDH	0.0047	GO.0046997	0.15702477
2	2	GO Function	nucleoside triphosphate adenylate kinase activity	0.0269	AK3|AK4	0.0047	GO.0046899	0.15702477
2	2	GO Function	electron transporter, transferring electrons from CoQH2-cytochrome c reductase complex and cytochrome c oxidase complex activity	0.0269	CYCS|CYC1	0.0047	GO.0045155	0.15702477
2	2	GO Function	propionyl-CoA carboxylase activity	0.0269	PCCA|PCCB	0.0047	GO.0004658	0.15702477
2	2	GO Function	phosphoenolpyruvate carboxykinase (GTP) activity	0.0269	PCK2|PCK1	0.0047	GO.0004613	0.15702477
2	2	GO Function	isocitrate dehydrogenase (NADP+) activity	0.0269	IDH2|IDH1	0.0047	GO.0004450	0.15702477
2	2	GO Function	glycerol-3-phosphate dehydrogenase (quinone) activity	0.0269	GPD1|GPD2	0.0047	GO.0004368	0.15702477

2	2	GO Function	glycerol-3-phosphate dehydrogenase [NAD+] activity	0.0269	GPD1L|GPD1	0.0047	GO.0004367	0.15702477
2	2	GO Function	dihydropyrimidinase activity	0.0269	DPYS|DPYSL2	0.0047	GO.0004157	0.15702477
2	2	GO Function	aconitate hydratase activity	0.0269	ACO2|ACO1	0.0047	GO.0003994	0.15702477
2	2	GO Function	3-methyl-2-oxobutanoate dehydrogenase (2-methylpropanoyl-transferring) activity	0.0269	BCKDHA|BCKDHB	0.0047	GO.0003863	0.15702477
2	2	GO Function	3-hydroxybutyrate dehydrogenase activity	0.0269	BDH2|BDH1	0.0047	GO.0003858	0.15702477
2	2	GO Function	alpha-ketoacid dehydrogenase activity	0.0269	BCKDHA|BCKDHB	0.0047	GO.0003826	0.15702477
2	2	GO Function	leukotriene-C(4) hydrolase	0.0269	GGT5|GGT1	0.0047	GO.0002951	0.15702477
2	2	GO Function	peptidyltransferase activity	0.0269	GGT5|GGT1	0.0047	GO.0000048	0.15702477
21	4	GO Function	2 iron, 2 sulfur cluster binding	0.0275	ISCU|CISD1|FECH|NDUFS1	0.0052	GO.0051537	0.15606673
21	4	GO Function	fatty acid ligase activity	0.0275	SLC27A2|ACSM2B|ACSL1|ACSM2A	0.0052	GO.0015645	0.15606673
85	8	GO Function	cysteine-type endopeptidase activity	0.0275	CTSH|CTSC|CTSD|TINAG|UCHL1|CAPN2|PDIA3|CTSB	0.0052	GO.0004197	0.15606673
10	3	GO Function	vinculin binding	0.0281	RTCB|TLN1|ACTN1	0.0054	GO.0017166	0.15512937
1992	80	GO Process	anatomical structure morphogenesis	0.0281	RALA|DCN|VCL|MAPK1|MYH9|MTHFD1|CTSH|PFN1|NAGLU|VTN|LTF|ATP6V1B1|LAMA5|GRSF1|LAMC1|ATP5B|SLC9A3R1|LRP2|ADD1|ENPEP|CALB1|AFG3L2|SOD1|UCHL1|ACTC1|CAPN2|COL1A2|HPRT1|ANPEP|GAA|DPYSL2|PRKACA|AQP1|UGDH|HSPA5|FBN1|PARVA|HSP90AA1|EPB41L3|ANXA2|COL18A1|RAC1|MSN|MYH10|COL4A2|OPA1|KRT19|TGM2|FLNA|GNAS|RBP4|CLIC4|HSPG2|COL4A1|FLOT1|POSTN|CD9|KRT18|DNM2|PRCP|CNP|FHL1|ACTN1|MYH11|ILK|FKBP1A|CDC42|RDH13|PNPT1|ACTA2|GPX1|TGFBI|SSBP1|CRYAB|RAP1GAP|PHB2|DNM1L|ITGB3|ACTG1|TTN	0.0035	GO.0009653	0.15512937
244	16	GO Process	muscle contraction	0.0284	VCL|GPD1L|ACTC1|GAA|TLN1|TPM4|ATP1B1|SNTB1|MYH11|ACTA2|CKMT2|CRYAB|ATP1A1|VIM|MYL6|TTN	0.0035	GO.0006936	0.15466817
135	10	KEGG Pathways	Apoptosis	0.0285	MAPK1|CTSH|CTSC|CTSD|TUBA4A|CAPN2|TUBA1C|CYCS|CTSB|ACTG1	0.009	hsa04210	0.15451551
59	9	KEGG Pathways	Glycerolipid metabolism	0.0007	ALDH2|ALDH3A2|ALDH9A1|AGK|AKR1A1|ALDH1B1|DAK|ALDH7A1|GK	0.00013	hsa00561	0.3154902
62	7	GO Process	glycosaminoglycan catabolic process	0.0285	DCN|NAGLU|VCAN|LUM|GLB1|BGN|HSPG2	0.0035	GO.0006027	0.15451551
46	6	GO Process	cofactor transport	0.0287	SLC25A11|SLC9A3R1|LRP2|AMN|SLC23A1|CUBN	0.0036	GO.0051181	0.15421181

46	6	GO Process	alcohol catabolic process	0.0287	ALDH2|SORD|GPD2|ALDH1B1|AKR1C3|GK	0.0036	GO.0046164	0.15421181
211	14	GO Function	peptidase regulator activity	0.0289	CTSH|CTSC|GAPDH|LTF|VIL1|PEBP1|AMBP|PRDX5|PRDX3|SERPINA5|ITIH2|DPEP1|AHSG|SERPINA1	0.0056	GO.0061134	0.15391022
1732	71	GO Process	regulation of transport	0.0295	RALA|OXCT1|MAPK1|PPIF|VTN|GAPDH|REEP6|ACTN4|LGALS3|ERP29|SLC9A3R1|EHD2|NAPA|CPT1A|PFKL|SLC25A4|GPD1L|SCIN|YWHAG|PRKACA|CYP4A11|AQP1|GBAS|DAB2|CALR|EHD3|IDH2|RAB11B|PFKM|ANXA2|RAC1|MSN|MYH10|DPP4|OPA1|C4BPA|ATP1B1|GSTO1|AHCYL1|FLNA|MYO6|GNAS|RBP4|SCP2|SLC25A5|YWHAB|RAB14|CLIC4|CLIC1|FLOT1|MAOB|POSTN|GLRX|SLC25A6|DNM2|JUP|FHL1|TSPO|ABAT|FKBP1A|RDX|TF|AHSG|PDZK1|CRYAB|ATP1A1|DNM1L|ITGB3|TTN|PLCG2|AP2B1	0.0037	GO.0051049	0.1530178
461	25	GO Process	wound healing	0.0295	DCN|MAPK1|F9|C1QBP|EHD2|F13A1|COL1A2|PRKACA|EHD3|SERPINA5|RAC1|MYH10|FLNA|GNAS|POSTN|HBD|AK3|CD9|PRCP|CORO1B|CDC42|GPX1|SERPINA1|ITGB3|PLCG2	0.0037	GO.0042060	0.1530178
4716	168	GO Process	heterocycle metabolic process	0.0299	NDUFB4|UQCRC1|NANS|MAPK1|RTCB|MTHFD1|AHCY|OGDH|PNPO|C1QBP|PDHX|GAPDH|DNPH1|LTF|ATP6V1B1|SPR|ENO1|GDA|PMPCB|NDUFA2|NDUFA10|LGALS3|GRSF1|HRSP12|CARS2|GGH|LRPPRC|ATP5B|CHCHD3|ATP6V0A1|NNT|SDHA|AMBP|PRDX5|REXO2|NDUFA9|UQCRC2|PFKL|ATP6V1A|ALDH1L1|MUT|ATP6V1B2|LACTB2|DPYS|NDUFB9|HNMT|DLAT|OXSM|ACAD8|QDPR|GPD1L|ATP5O|FTCD|ATP5J2|BDH2|HPRT1|AMN|NDUFB8|ATP5L|GPD1|PRODH2|ATP5H|SLC23A1|DCXR|HINT1|ATP5I|PDHB|CYCS|ATP6V0A4|GPD2|DPYSL2|UQCRH|PRKACA|AQP1|GBAS|ACSS1|CYC1|PIPOX|COX5A|SHMT1|UGDH|NDUFV1|MDH2|ACSM2B|NDUFA12|IDH2|UQCR10|MRPL12|HSP90AA1|DLST|PKLR|RPS2|PPA2|PFKM|STOML2|ATP5C1|COL4A2|LONP1|MPC1|MT-CO1|PNP|OPA1|MT-CO2|DARS2|IBA57|IARS2|ATP1B1|LHPP|ATP5F1|AHCYL1|FLNA|GNAS|ALDH18A1|CMPK1|OGDHL|ALDOB|ALDH4A1|VARS|PCCA|PSAT1|CUBN|SUCLA2|ACOT9|PDHA1|GLRX|DUT|PFKP|AK3|FECH|DPEP1|NME2|CNP|PC|AK4|QPRT|TSPO|AMPD3|ATP5A1|ALAD|SSB|IDH1|TKT|NDUFS1|PNPT1|NAPRT|ACSF2|HK1|DDC|GPX1|LARS2|MME|PCCB|SSBP1|ABHD14B|ACSL1|COX7C|EPHX2|PHB2|ALDH6A1|COX4I1|ACSM2A|RBM8A|GALK1|SARS2|PHB|EPHX1|FMO1|GLYAT	0.0037	GO.0046483	0.15243288
3	2	GO Component	myosin II filament	0.0265	MYH9|MYH10	0.0077	GO.0097513	0.15767541
19	4	GO Process	cellular glucuronidation	0.0304	ABHD10|UGT2B7|UGT2B17|UGT1A9	0.0038	GO.0052695	0.15171264
19	4	GO Process	mitochondrial fission	0.0304	MTFP1|OPA1|DNM2|DNM1L	0.0038	GO.0000266	0.15171264
32	5	GO Process	GTP metabolic process	0.0307	OPA1|AK3|NME2|AK4|AMPD3	0.0039	GO.0046039	0.15128616
47	6	GO Process	regulation of sodium ion transmembrane transporter activity	0.0311	ACTN4|SLC9A3R1|GPD1L|ATP1B1|GLRX|DNM2	0.0039	GO.2000649	0.15072396
1106	49	GO Process	cellular response to endogenous stimulus	0.0311	MAPK1|CTSH|RAB35|ATP6V1B1|VIL1|ATP6V0A1|SOD1|ATP6V1A|ATP6V1B2|ATP6V0D2|ATP6V0D1|CAPN2|COL1A2|HSP90B1|GPD1|YWHAG|ATP6V0A4|PRKACA|AQP1|SHMT1|PCK1|CALR|HSPA5|FBN1|PKLR|CTSB|MSN|COL4A2|OPA1|AHCYL1|GOT1|GNAS|ASS1|RAB14|COL4A1|ACTR2|POSTN|AKR1C3|FECH|DNM2|CORO1B|JUP|GSTP1|RDX|DDC|AOC1|ACSL1|ATP1A1|PHB	0.0039	GO.0071495	0.15072396
169	12	GO Function	endopeptidase inhibitor activity	0.0312	GAPDH|LTF|VIL1|PEBP1|AMBP|PRDX5|PRDX3|SERPINA5|ITIH2|DPEP1|AHSG|SERPINA1	0.0061	GO.0004866	0.15058454
81	8	GO Process	heart contraction	0.0315	SOD1|GPD1L|ACTC1|GAA|ATP1B1|GPX1|ATP1A1|TTN	0.004	GO.0060047	0.15016894

81	8	GO Process	regulation of receptor-mediated endocytosis	0.0315	VTN|DAB2|ANXA2|RAC1|FLOT1|DNM2|TF|PLCG2	0.004	GO.0048259	0.15016894
119	10	GO Process	secondary alcohol metabolic process	0.0316	ACO2|IDH3G|ERLIN2|IDH2|CYB5R3|HMGCS2|CUBN|IDH3B|IDH1|NPC2	0.004	GO.1902652	0.15003129
88	8	GO Function	steroid binding	0.0323	PGRMC1|CALB1|ERLIN2|ANXA6|SCP2|TSPO|ATP1A1|NPC2	0.0063	GO.0005496	0.14907975
160	11	KEGG Pathways	cGMP-PKG signaling pathway	0.0325	MAPK1|PPIF|VDAC1|SLC25A4|GNAI1|ATP1B1|SLC25A5|VDAC2|SLC25A6|VDAC3|ATP1A1	0.0104	hsa04022	0.14881166
128	10	GO Function	heme binding	0.0329	PGRMC1|AMBP|CYCS|CYP4A11|CYC1|CYB5A|PTGES2|MT-CO1|HBD|SUOX	0.0065	GO.0020037	0.14828041
183	12	KEGG Pathways	Viral carcinogenesis	0.0329	MAPK1|ACTN4|ATP6V0D2|ATP6V0D1|SCIN|YWHAG|PRKACA|RAC1|YWHAB|ACTN1|CDC42|VDAC3	0.0107	hsa05203	0.14828041
42	5	KEGG Pathways	Porphyrin and chlorophyll metabolism	0.0329	UGT2B7|UGT2B17|UGT1A9|FECH|ALAD	0.0108	hsa00860	0.14828041
182	13	GO Process	striated muscle cell differentiation	0.0329	MYH9|AFG3L2|UCHL1|ACTC1|CAPN2|CALR|MYH10|KRT19|MYH11|GPX1|ACTG1|TTN|PDLIM5	0.0042	GO.0051146	0.14828041
9	3	GO Process	protein localization to membrane raft	0.0332	ATP1B1|FLOT1|FLOT2	0.0042	GO.1903044	0.14788619
9	3	GO Process	ketone body biosynthetic process	0.0332	BDH2|HMGCS2|BDH1	0.0042	GO.0046951	0.14788619
9	3	GO Process	water-soluble vitamin biosynthetic process	0.0332	PNPO|AKR1A1|PSAT1	0.0042	GO.0042364	0.14788619
9	3	GO Process	ATF6-mediated unfolded protein response	0.0332	HSP90B1|CALR|HSPA5	0.0042	GO.0036500	0.14788619
9	3	GO Process	tetrahydrofolate interconversion	0.0332	MTHFD1|FTCD|SHMT1	0.0042	GO.0035999	0.14788619
9	3	GO Process	ADP transport	0.0332	SLC25A4|SLC25A5|SLC25A6	0.0042	GO.0015866	0.14788619
9	3	GO Process	regulation of mitochondrial fusion	0.0332	VAT1|OPA1|DNM1L	0.0042	GO.0010635	0.14788619
82	8	GO Process	regulation of sodium ion transport	0.0332	ACTN4|SLC9A3R1|GPD1L|ATP1B1|AHCYL1|GLRX|DNM2|ATP1A1	0.0043	GO.0002028	0.14788619
48	6	GO Process	guanosine-containing compound metabolic process	0.0335	HPRT1|OPA1|AK3|NME2|AK4|AMPD3	0.0043	GO.1901068	0.14749552
48	6	GO Process	actin filament bundle assembly	0.0335	ACTN4|ADD1|HSP90B1|PLS1|CORO1B|ACTN1	0.0043	GO.0051017	0.14749552
11	3	GO Function	actin-dependent ATPase activity	0.0341	MYH9|MYH10|MYL6	0.0067	GO.0030898	0.14672456
11	3	GO Function	AMP binding	0.0341	ACSS1|CYB5R3|FBP1	0.0067	GO.0016208	0.14672456
20	4	GO Process	mitochondrion morphogenesis	0.0345	OPA1|PNPT1|SSBP1|DNM1L	0.0045	GO.0070584	0.14621809
121	10	GO Process	striated muscle cell development	0.0345	AFG3L2|UCHL1|ACTC1|MYH10|KRT19|MYH11|GPX1|ACTG1|TTN|PDLIM5	0.0045	GO.0055002	0.14621809
20	4	GO Process	heme biosynthetic process	0.0345	IBA57|FECH|TSPO|ALAD	0.0045	GO.0006783	0.14621809

83	8	GO Process	regulation of purine nucleotide metabolic process	0.035	GSTZ1|PPIF|ENO1|GPD1|MT-CO2|PDP1|TSPO|FBP1	0.0046	GO.1900542	0.1455932
83	8	GO Process	regulation of cellular pH	0.035	MAPK1|ATP5B|ATP6V0A1|ATP6V0D2|ATP6V0D1|ATP6V0A4|CLIC4|SLC4A4	0.0046	GO.0030641	0.1455932
163	11	KEGG Pathways	Hepatocellular carcinoma	0.0355	MAPK1|GSTM3|GSTA1|MGST3|GSTO1|MGST1|GSTP1|TXNRD2|GSTA2|ACTG1|PLCG2	0.0118	hsa05225	0.14497716
336	19	GO Function	anion transmembrane transporter activity	0.0356	SLC25A11|VDAC1|SLC27A2|SLC25A4|SLC23A1|SLC7A8|SLC25A15|MPC1|SLC25A5|VDAC2|CLIC4|CLIC1|SLC3A2|SLC25A6|PCYOX1|SLC25A12|SLC4A4|VDAC3|SLC25A10	0.0071	GO.0008509	0.144855
90	8	GO Function	hormone binding	0.0357	CRYM|CTSH|TTR|ALDH1A1|CALR|NQO2|TSPO|ATP1A1	0.0071	GO.0042562	0.14473318
120	9	KEGG Pathways	AMPK signaling pathway	0.0358	PCK2|CPT1A|PFKL|PCK1|RAB11B|PFKM|RAB14|PFKP|FBP1	0.012	hsa04152	0.1446117
2	2	GO Process	protein localization to bicellular tight junction	0.036	ACTN4|FLNA	0.0047	GO.1902396	0.14436975
2	2	GO Process	glycolytic process through glucose-1-phosphate	0.036	PFKM|GALK1	0.0047	GO.0061622	0.14436975
252	16	GO Process	carboxylic acid transport	0.036	SLC25A11|GOT2|SLC9A3R1|LRP2|CPT1A|SLC27A2|FABP1|SLC23A1|SLC7A8|SLC25A15|MPC1|ABCD3|SLC3A2|SLC25A12|ACSL1|SLC25A10	0.0047	GO.0046942	0.14436975
2	2	GO Process	formaldehyde catabolic process	0.036	ADH5|ESD	0.0047	GO.0046294	0.14436975
2	2	GO Process	glycerol-3-phosphate catabolic process	0.036	GPD1L|GPD1	0.0047	GO.0046168	0.14436975
2	2	GO Process	protein unfolding	0.036	ERP29|HSP90AA1	0.0047	GO.0043335	0.14436975
2	2	GO Process	regulation of isoprenoid metabolic process	0.036	AKR1C3|NPC2	0.0047	GO.0019747	0.14436975
2	2	GO Process	glutamate catabolic process to 2-oxoglutarate	0.036	GOT2|GOT1	0.0047	GO.0019551	0.14436975
2	2	GO Process	glutamate catabolic process to aspartate	0.036	GOT2|GOT1	0.0047	GO.0019550	0.14436975
2	2	GO Process	glycine betaine biosynthetic process from choline	0.036	CHDH|ALDH7A1	0.0047	GO.0019285	0.14436975
122	10	GO Process	regulation of cellular ketone metabolic process	0.036	GSTZ1|CPT1A|BHMT|ERLIN2|FABP1|UGT1A9|AKR1C3|PDP1|ACADVL|SIRT5	0.0047	GO.0010565	0.14436975
2	2	GO Process	cyanate catabolic process	0.036	MPST|TST	0.0047	GO.0009440	0.14436975
2	2	GO Process	pyridoxine biosynthetic process	0.036	PNPO|PSAT1	0.0047	GO.0008615	0.14436975
2	2	GO Process	glycerophosphate shuttle	0.036	GPD1|GPD2	0.0047	GO.0006127	0.14436975
2	2	GO Process	glyoxylate cycle	0.036	IDH2|IDH1	0.0047	GO.0006097	0.14436975

103	9	GO Process	endoplasmic reticulum unfolded protein response	0.0364	ADD1|ATP6V0D1|TPP1|HSP90B1|TLN1|CALR|HSPA5|PDIA6|ACADVL	0.0049	GO.0030968	0.14388986
1638	67	GO Process	regulation of cell death	0.0371	LGALS1|CTSH|PPIF|C1QBP|CTSC|GAPDH|LTF|ENO1|TRAP1|VIL1|HSPB1|ACTN4|LGALS3|ERP29|SLC9A3R1|NNT|PRDX5|SOD1|NAT8|HIGD2A|SLC25A4|ACTC1|FABP1|SCIN|HSPA9|PRDX3|HSP90B1|PDIA3|YWHAG|CYCS|AQP1|DAB2|CALR|HSPA5|P4HB|CTSB|GPX4|COL18A1|OPA1|MT-CO2|TGM2|FLNA|SLC25A5|VDAC2|YWHAB|HSPG2|TXNDC5|AKR1C3|KRT18|DNM2|DPEP1|NME2|ACTN1|TSPO|ILK|GSTP1|HIGD1A|NDRG1|GPX1|NDUFA13|CRYAB|PHB2|DNM1L|SIRT5|PHB|PLCG2|AP2B1	0.005	GO.0010941	0.14306261
3	2	GO Component	alphav-beta3 integrin-vitronectin complex	0.0265	VTN|ITGB3	0.0077	GO.0071062	0.15767541
50	6	GO Process	positive regulation of receptor-mediated endocytosis	0.0383	VTN|DAB2|ANXA2|DNM2|TF|PLCG2	0.0052	GO.0048260	0.14168012
21	4	GO Process	reactive nitrogen species metabolic process	0.0385	SPR|PRDX5|MARC2|SUOX	0.0052	GO.2001057	0.14145393
21	4	GO Process	protein insertion into mitochondrial membrane	0.0385	HSP90AA1|SAMM50|AGK|NDUFA13	0.0052	GO.0051204	0.14145393
873	40	GO Process	negative regulation of programmed cell death	0.0385	CTSH|PPIF|LTF|ENO1|TRAP1|VIL1|HSPB1|LGALS3|NNT|PRDX5|SOD1|NAT8|HIGD2A|SLC25A4|ACTC1|FABP1|HSPA9|PRDX3|HSP90B1|AQP1|DAB2|HSPA5|GPX4|OPA1|FLNA|SLC25A5|VDAC2|TXNDC5|KRT18|DPEP1|NME2|ILK|GSTP1|HIGD1A|GPX1|NDUFA13|CRYAB|PHB2|SIRT5|PLCG2	0.0052	GO.0043069	0.14145393
21	4	GO Process	purine nucleobase metabolic process	0.0385	MTHFD1|GDA|HPRT1|SHMT1	0.0052	GO.0006144	0.14145393
3	2	GO Function	medium-chain-acyl-CoA dehydrogenase activity	0.0386	ACAD11|ACAD9	0.0077	GO.0070991	0.14134127
3	2	GO Function	3 iron, 4 sulfur cluster binding	0.0386	ACO2|ACO1	0.0077	GO.0051538	0.14134127
3	2	GO Function	methylumbelliferyl-acetate deacetylase activity	0.0386	CES2|ESD	0.0077	GO.0047374	0.14134127
3	2	GO Function	maltose alpha-glucosidase activity	0.0386	GAA|MGAM	0.0077	GO.0032450	0.14134127
3	2	GO Function	oxidoreductase activity, acting on the CH-OH group of donors, oxygen as acceptor	0.0386	ALDH3A2|HAO2	0.0077	GO.0016899	0.14134127
392	21	GO Function	ATPase activity	0.0386	MYH9|ATP5B|ATP6V0A1|ATP6V1A|ATP6V1B2|ATP6V0D2|ATP5O|ACTC1|ATP6V0D1|ATP6V0A4|HSPA5|HSP90AA1|ATP5C1|MYH10|LONP1|ATP1B1|ABCD3|ATP5A1|PCYOX1|ATP1A1|MYL6	0.0084	GO.0016887	0.14134127

3	2	GO Function	oxidoreductase activity, acting on a sulfur group of donors, quinone or similar compound as acceptor	0.0386	SQRDL|GSTO1	0.0077	GO.0016672	0.14134127
3	2	GO Function	long-chain-3-hydroxyacyl-CoA dehydrogenase activity	0.0386	HADHB|HADHA	0.0077	GO.0016509	0.14134127
3	2	GO Function	long-chain-enoyl-CoA hydratase activity	0.0386	EHHADH|HADHA	0.0077	GO.0016508	0.14134127
3	2	GO Function	malate transmembrane transporter activity	0.0386	SLC25A11|SLC25A10	0.0077	GO.0015140	0.14134127
3	2	GO Function	protein kinase C inhibitor activity	0.0386	HSPB1|YWHAG	0.0077	GO.0008426	0.14134127
3	2	GO Function	thiosulfate sulfurtransferase activity	0.0386	MPST|TST	0.0077	GO.0004792	0.14134127
3	2	GO Function	pyruvate dehydrogenase (acetyl-transferring) activity	0.0386	PDHB|PDHA1	0.0077	GO.0004739	0.14134127
3	2	GO Function	oxoglutarate dehydrogenase (succinyl-transferring) activity	0.0386	OGDH|OGDHL	0.0077	GO.0004591	0.14134127
3	2	GO Function	nicotinate-nucleotide diphosphorylase (carboxylating) activity	0.0386	QPRT|NAPRT	0.0077	GO.0004514	0.14134127
3	2	GO Function	dodecenoyl-CoA delta-isomerase activity	0.0386	EHHADH|ECI1	0.0077	GO.0004165	0.14134127
3	2	GO Function	L-aspartate:2-oxoglutarate aminotransferase activity	0.0386	GOT2|GOT1	0.0077	GO.0004069	0.14134127
198	13	GO Function	mRNA binding	0.0386	C1QBP|GRSF1|CCT5|MRPL13|ACO1|SHMT1|CALR|RPS2|MYH10|AUH|SSB|CRYZ|RBM8A	0.008	GO.0003729	0.14134127
12	3	GO Function	aminoacyl-tRNA editing activity	0.0386	IARS2|VARS|LARS2	0.0082	GO.0002161	0.14134127
270	16	GO Function	peptide binding	0.0392	PPIF|LTA4H|GSTM3|ENPEP|RNPEP|TPP1|PPIB|ANPEP|SLC7A8|CALR|PTGES2|MGST1|GSTP1|MME|CRYAB|AP2B1	0.0086	GO.0042277	0.14067139
10	3	GO Process	fatty acid derivative catabolic process	0.0395	OXCT1|CYP4A11|BDH1	0.0054	GO.1901569	0.14034029

10	3	GO Process	vacuolar proton-transporting V-type ATPase complex assembly	0.0395	ATP6V0A1|ATP6V0A4|ALDOB	0.0054	GO.0070072	0.14034029
10	3	GO Process	proline metabolic process	0.0395	PRODH2|ALDH18A1|ALDH4A1	0.0054	GO.0006560	0.14034029
10	3	GO Process	histidine metabolic process	0.0395	MTHFD1|HNMT|FTCD	0.0054	GO.0006547	0.14034029
10	3	GO Process	T cell mediated cytotoxicity	0.0395	CTSH|CTSC|HPRT1	0.0054	GO.0001913	0.14034029
3	2	GO Component	laminin-11 complex	0.0265	LAMA5|LAMC1	0.0077	GO.0043260	0.15767541
35	5	GO Process	cellular response to catecholamine stimulus	0.0396	MAPK1|PRKACA|PKLR|GNAS|DNM2	0.0054	GO.0071870	0.14023048
25	4	GO Function	protein folding chaperone	0.0405	HSPB1|CCT5|CCT2|CALR	0.009	GO.0044183	0.1392545
123	9	KEGG Pathways	Platelet activation	0.0407	MAPK1|COL1A2|PRKACA|TLN1|GNAI1|GNAS|ITGB3|ACTG1|PLCG2	0.0138	hsa04611	0.13904056
68	7	GO Process	digestive system process	0.0408	VIL1|FABP1|ACO1|AQP1|PLS1|PBLD|RBP4	0.0056	GO.0022600	0.13893398
51	6	GO Process	positive regulation of reactive oxygen species biosynthetic process	0.0409	AGXT2|DDAH1|HSP90AA1|MT-CO2|ASS1|DNM2	0.0057	GO.1903428	0.13882767
3	2	GO Component	laminin-10 complex	0.0265	LAMA5|LAMC1	0.0077	GO.0043259	0.15767541
961	43	GO Process	regulation of anatomical structure morphogenesis	0.0413	DCN|MYH9|CTSH|PFN1|C1QBP|VIL1|HSPB1|ACTN4|SLC9A3R1|EHD2|DDAH1|DPYSL2|AQP1|DAB2|CALR|AP2A2|PARVA|EPB41L3|VAT1|RAC1|MSN|MYH10|COL4A2|OPA1|S100A10|FLNA|HSPG2|ASPN|FLOT1|ACTR2|POSTN|DNM2|CORO1B|JUP|ILK|SEPT7|CDC42|RDX|IST1|PHB2|DNM1L|PDLIM5|AP2B1	0.0057	GO.0022603	0.13840499
57	6	GO Function	cysteine-type endopeptidase inhibitor activity	0.0414	LTF|VIL1|PRDX5|PRDX3|DPEP1|AHSG	0.0092	GO.0004869	0.13829997
743	35	GO Process	regulation of cellular catabolic process	0.0423	DCN|CTSC|GAPDH|ATP6V1B1|HSPB1|LRPPRC|CD81|LRP2|ATP6V0A1|NNT|VDAC1|CPT1A|ATP6V1A|ATP6V1B2|UCHL1|ATP6V0D2|ATP6V0D1|FABP1|GPD1|PRKACA|RAB1B|DAB2|HSP90AA1|ANXA2|MSN|YWHAB|TSPO|RDX|ALAD|IDH1|PNPT1|GPX1|FBP1|VIM|DNM1L	0.0059	GO.0031329	0.13736596
825	38	GO Process	response to lipid	0.0426	DCN|MAPK1|CTSH|HRSP12|CPT1A|ADH5|PRDX3|GLB1|AQP1|PCK1|CALR|GNAI1|MSN|GOT1|GNAS|RBP4|ASS1|MAOB|POSTN|AKR1C3|GLDC|FECH|CNP|MGST1|TSPO|GSTP1|ALAD|ARPC1B|IDH1|ACSL1|NDUFA13|CRYAB|PHB2|ATP1A1|DNM1L|PHB|FMO1|PLCG2	0.0059	GO.0033993	0.13705904
4	2	GO Component	mitochondrial permeability transition pore complex	0.0379	PPIF|VDAC1	0.0114	GO.0005757	0.14213608

664	32	GO Process	metal ion transport	0.0433	LTF|ATP6V1B1|PMPCB|SLC9A3R1|LRP2|ATP6V0A1|NDUFA9|AFG3L2|ATP6V1A|ATP6V1B2|ATP6V0D2|ATP6V0D1|SLC23A1|LETM1|ATP6V0A4|AQP1|SLC5A2|RAB11B|ANXA6|STOML2|OPA1|ATP1B1|PMPCA|SLC3A2|DNM2|FKBP1A|TF|SLC4A4|PHB2|ATP1A1|PHB|PLCG2	0.0061	GO.0030001	0.13635121
22	4	GO Process	regulation of necrotic cell death	0.0433	PPIF|SLC25A4|MT-CO2|TSPO	0.006	GO.0010939	0.13635121
52	6	GO Process	response to zinc ion	0.044	GGH|KHK|ASS1|MT1M|TSPO|ALAD	0.0062	GO.0010043	0.13565473
26	4	GO Function	cysteine-type endopeptidase inhibitor activity involved in apoptotic process	0.0452	VIL1|PRDX5|PRDX3|DPEP1	0.0101	GO.0043027	0.13448616
800	36	GO Function	zinc ion binding	0.0452	UQCRC1|LTA4H|GDA|PMPCB|BHMT2|BBOX1|ENPEP|CALB1|SORD|UQCRC2|AFG3L2|SOD1|BHMT|LACTB2|DPYS|RNPEP|ADH5|ANPEP|CA4|ADH1B|CALR|C11orf54|VAT1|DPP3|CSRP1|RTN4IP1|PMPCA|MT1M|NQO2|PITRM1|DPEP1|ALAD|CRYZ|AOC1|MME|SIRT5	0.0101	GO.0008270	0.13448616
170	12	GO Process	carboxylic acid transmembrane transport	0.0452	SLC25A11|LRP2|CPT1A|SLC27A2|SLC23A1|SLC7A8|SLC25A15|MPC1|ABCD3|SLC3A2|SLC25A12|SLC25A10	0.0063	GO.1905039	0.13448616
84	7	KEGG Pathways	Rheumatoid arthritis	0.0455	ATP6V1B1|ATP6V0A1|ATP6V1A|ATP6V1B2|ATP6V0D2|ATP6V0D1|ATP6V0A4	0.0156	hsa05323	0.13419886
30	4	KEGG Pathways	Nicotinate and nicotinamide metabolism	0.0455	NNT|PNP|QPRT|NAPRT	0.0156	hsa00760	0.13419886
333	19	GO Process	regulation of cellular component size	0.0461	PFN1|DSTN|VIL1|SLC9A3R1|ADD1|IQGAP2|SCIN|DPYSL2|AQP1|PLS1|RAC1|MSN|ACTR2|DNM2|CORO1B|ILK|RDX|ARPC1B|IST1	0.0065	GO.0032535	0.13362991
70	7	GO Process	regulation of plasma lipoprotein particle levels	0.0462	AMN|PRKACA|AP2A2|P4HB|CUBN|NPC2|AP2B1	0.0065	GO.0097006	0.1335358
59	6	GO Function	GDP binding	0.0473	RALA|RAB35|PCK1|RAB11B|GNAI1|RAB14	0.0107	GO.0019003	0.13251389
411	22	Reactome Pathways	Metabolism of proteins	0.0476	NANS|NAPSA|APCS|MRPL15|CALB1|NAT8|MRPL49|UCHL1|MRPL17|MRPL39|MRPL13|TSFM|MSRA|TUFM|MRPL12|MRPL37|MRPL21|UMOD|TGFBI|MRPS22|GFM1|MRPS30	0.0071	HSA-392499	0.1322393
37	5	GO Process	toxin transport	0.0477	ATP6V0A1|CCT5|CCT2|SLC7A8|HSPA5	0.0067	GO.1901998	0.13214816
129	10	GO Process	response to calcium ion	0.0477	PPIF|ADD1|HSPA5|AHCYL1|CLIC4|AKR1C3|DPEP1|SLC25A12|ACTG1|TTN	0.0068	GO.0051592	0.13214816
310	18	GO Process	cellular response to drug	0.0477	MAPK1|PPIF|SOD1|QDPR|FABP1|HSP90B1|AQP1|HSPA5|GNAI1|OPA1|ASS1|FECH|DNM2|DPEP1|JUP|DDC|FBP1|AOC1	0.0067	GO.0035690	0.13214816
11	3	GO Process	ATP transport	0.0477	SLC25A4|SLC25A5|SLC25A6	0.0067	GO.0015867	0.13214816
11	3	GO Process	protein kinase A signaling	0.0477	FBN1|RDX|TTN	0.0067	GO.0010737	0.13214816
11	3	GO Process	deoxyribonucleoside monophosphate metabolic process	0.0477	DNPH1|SHMT1|DUT	0.0067	GO.0009162	0.13214816
11	3	GO Process	nucleoside monophosphate catabolic process	0.0477	DNPH1|HPRT1|AMPD3	0.0067	GO.0009125	0.13214816

11	3	GO Process	serine family amino acid catabolic process	0.0477	AMT|SHMT1|GLDC	0.0067	GO.0009071	0.13214816
11	3	GO Process	L-serine metabolic process	0.0477	SHMT1|PHGDH|PSAT1	0.0067	GO.0006563	0.13214816
11	3	GO Process	aspartate metabolic process	0.0477	GOT2|GOT1|ASS1	0.0067	GO.0006531	0.13214816
11	3	GO Process	disaccharide metabolic process	0.0477	GAA|FBP1|MGAM	0.0067	GO.0005984	0.13214816
11	3	GO Process	urea cycle	0.0477	ASL|SLC25A15|ASS1	0.0067	GO.0000050	0.13214816
263	16	GO Process	regulation of cell morphogenesis involved in differentiation	0.048	C1QBP|ACTN4|DPYSL2|CALR|RAC1|OPA1|S100A10|FLNA|ACTR2|POSTN|DNM2|ILK|CDC42|IST1|DNM1L|PDLIM5	0.0069	GO.0010769	0.13187588
23	4	GO Process	modulation by host of viral process	0.0482	LTF|APCS|PPIB|PC	0.0069	GO.0044788	0.1316953
23	4	GO Process	response to muscle activity	0.0482	GOT2|MTFP1|OPA1|POSTN	0.0069	GO.0014850	0.1316953
71	7	GO Process	modification by host of symbiont morphology or physiology	0.0485	GAPDH|LTF|APCS|PPIB|AQP1|ANXA2|PC	0.007	GO.0051851	0.13142583
71	7	GO Process	regulation of cofactor metabolic process	0.0485	GSTZ1|NNT|GPD1|RAC1|MT-CO2|PDP1|FBP1	0.007	GO.0051193	0.13142583
71	7	GO Process	endothelial cell differentiation	0.0485	LAMA5|GSTM3|COL18A1|MSN|CLIC4|RDX|GPX1	0.007	GO.0045446	0.13142583
753	35	GO Process	regulation of cell migration	0.0492	DCN|VCL|MAPK1|CTSH|PFN1|C1QBP|VTN|VIL1|HSPB1|ACTN4|LAMA5|LGALS3|ADAM10|ATP5B|SLC9A3R1|DAB2|CALR|HSPA5|IDH2|COL18A1|RAC1|PBLD|MSN|FLNA|CLIC4|POSTN|DPEP1|PRCP|CORO1B|JUP|ILK|GSTP1|ATP5A1|RDX|ITGB3	0.0071	GO.0030334	0.13080349
