# Supplementary material for: Proteomic analysis of chromophobe renal cell carcinoma and benign renal oncocytoma biopsies reveals shared metabolic dysregulation
Source: Clin Proteomics. 2023 Nov 28;20:54. doi: 10.1186/s12014-023-09443-8 (PMC10683195; doi:10.1186/s12014-023-09443-8)
Supplement: Supplementary file 4 — Additional file 4: Figure S1. TPA concentration values (pmol/mg of tissue) of respiratory chain complex I (CI), II (CII) and III (CIII)proteins. Statistical analysis was performed using pairwise Mann Whitney test (**p ≤ 0.001; ***p ≤ 0.0001; ****p ≤ 0.00001). Figure S2. TPA concentration values (pmol/mg of tissue) of respiratory chain complex IV (CIV) and V (CV) proteins. Statistical analysis was performed using pairwise Mann Whitney test (**p ≤ 0.001; ***p ≤ 0.0001;****p ≤ 0.00001). [file 12014_2023_9443_MOESM4_ESM.pdf]

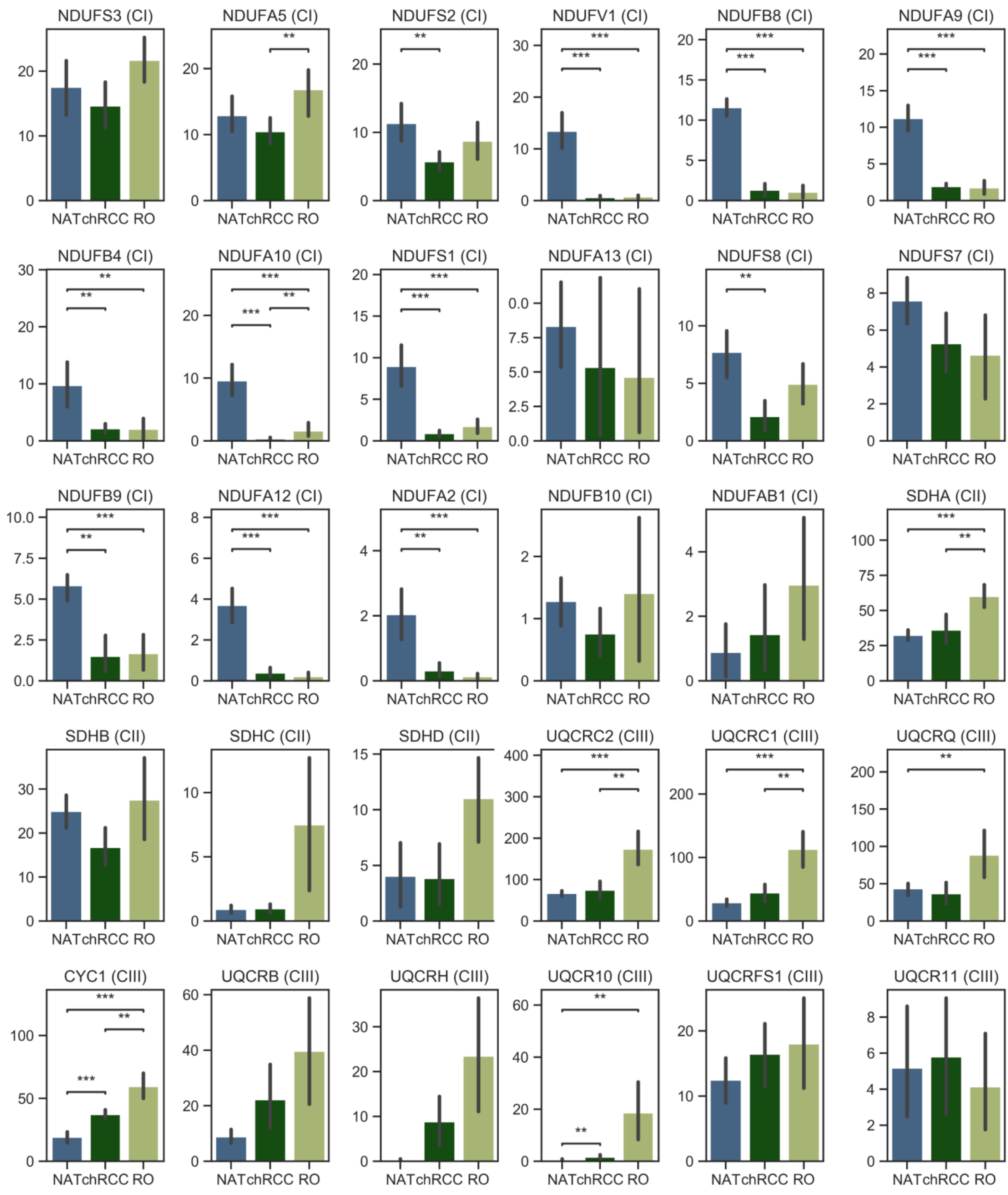

Additional file 4: Fig. S1 – TPA concentration values (pmol/mg of tissue) of respiratory chain complex I (CI), II (CII) and III (CIII) proteins. Statistical analysis was performed using pairwise Mann Whitney test (**p ≤ 0.001; ***p ≤ 0.0001; ****p ≤ 0.00001).

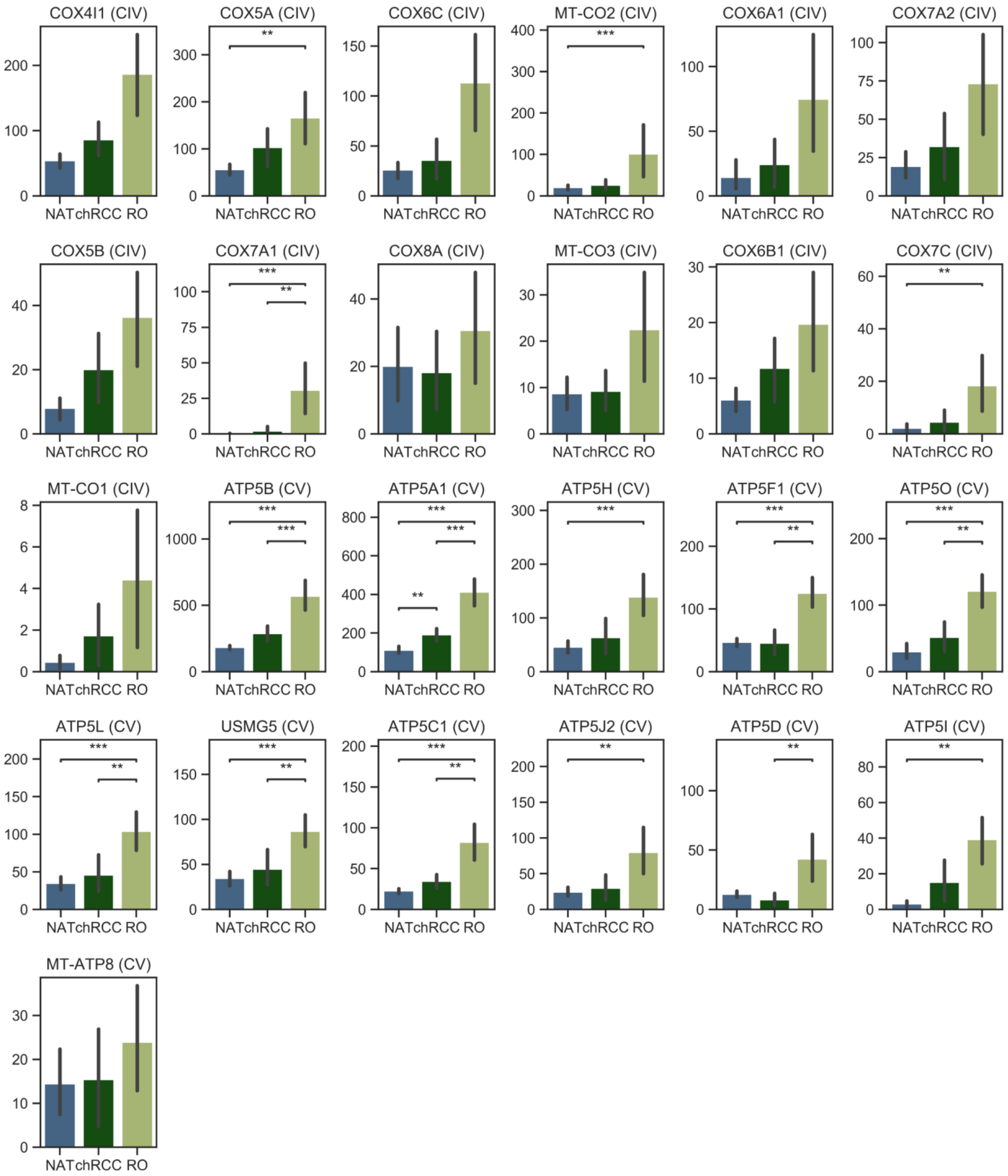

Additional file 4: Fig. S2. – TPA concentration values (pmol/mg of tissue) of respiratory complex IV (CIV) and V (CV) proteins. Statistical analysis was performed using pairwise Mann Whitney test (**p ≤ 0.001; ***p ≤ 0.0001; ****p ≤ 0.00001).
